# Antigen-encapsulating host extracellular vesicles derived from *Salmonella*-infected cells stimulate pathogen-specific Th1-type responses in vivo

**DOI:** 10.1371/journal.ppat.1009465

**Published:** 2021-05-06

**Authors:** Winnie W. Hui, Lisa E. Emerson, Beata Clapp, Austin E. Sheppe, Jatin Sharma, Johanna del Castillo, Mark Ou, Gustavo H. B. Maegawa, Carol Hoffman, Joseph Larkin, III, David W. Pascual, Mariola J. Ferraro

**Affiliations:** 1 Department of Microbiology and Cell Science, Institute of Food and Agricultural Sciences, University of Florida, Gainesville, Florida, United States of America; 2 Department of Medicine, College of Medicine, University of Florida, Gainesville, Florida, United States of America; 3 Department of Infectious Diseases & Immunology, College of Veterinary Medicine, University of Florida, Gainesville, Florida, United States of America; 4 Department of Pediatrics, College of Medicine, University of Florida, Gainesville, Florida, United States of America; University of Pittsburgh, UNITED STATES

## Abstract

*Salmonella* Typhimurium is a causative agent of nontyphoidal salmonellosis, for which there is a lack of a clinically approved vaccine in humans. As an intracellular pathogen, *Salmonella* impacts many cellular pathways. However, the intercellular communication mechanism facilitated by host-derived small extracellular vesicles (EVs), such as exosomes, is an overlooked aspect of the host responses to this infection. We used a comprehensive proteome-based network analysis of exosomes derived from *Salmonella*-infected macrophages to identify host molecules that are trafficked via these EVs. This analysis predicted that the host-derived small EVs generated during macrophage infection stimulate macrophages and promote activation of T helper 1 (Th1) cells. We identified that exosomes generated during infection contain *Salmonella* proteins, including unique antigens previously shown to stimulate protective immune responses against *Salmonella* in murine studies. Furthermore, we showed that host EVs formed upon infection stimulate a mucosal immune response against *Salmonella* infection when delivered intranasally to BALB/c mice, a route of antigen administration known to initiate mucosal immunity. Specifically, the administration of these vesicles to animals stimulated the production of anti-*Salmonella* IgG antibodies, such as anti-OmpA antibodies. Exosomes also stimulated antigen-specific cell-mediated immunity. In particular, splenic mononuclear cells isolated from mice administered with exosomes derived from *Salmonella*-infected antigen-presenting cells increased CD4+ T cells secreting Th1-type cytokines in response to *Salmonella* antigens. These results demonstrate that small EVs, formed during infection, contribute to Th1 cell bias in the anti-*Salmonella* responses. Collectively, this study helps to unravel the role of host-derived small EVs as vehicles transmitting antigens to induce Th1-type immunity against Gram-negative bacteria. Understanding the EV-mediated defense mechanisms will allow the development of future approaches to combat bacterial infections.

## Author summary

*Salmonella* Typhimurium is a causative agent of nontyphoidal salmonellosis, and we still lack a clinically approved vaccine against this infection. The design of efficacious vaccines against *Salmonella* can benefit from studying overlooked aspects of host immune responses to this pathogen. The cell-to-cell communication facilitated by nano-sized extracellular vesicles (EVs) can affect the immune response. Our earlier work showed that *Salmonella* infection leads to the generation of immunogenic small EVs by macrophages. Here, we performed a proteomic and metabolomic analysis of the EV cargo, which indicated that EVs generated by *Salmonella*-infected macrophages stimulate macrophages and promote T-helper 1 (Th1) cell activation. We confirmed in vivo that mucosal administration of these EVs promotes a mucosal immune response against *Salmonella* infection in BALB/c mice. EVs from infected macrophages increased the CD4+ T cells secreting IL-2, IFN-γ, and TNF-α in response to *Salmonella* antigens, contributing to Th1 cell bias. These EVs not only stimulated antigen-specific cell-mediated immunity, but also contributed to anti-*Salmonella* antibody production, which included IgGs recognizing bacterial proteins present in EVs. Our study unraveled a novel role of EVs as vehicles transmitting antigens to induce Th1-type immunity against Gram-negative bacteria, thus supporting the future development of preventative approaches to combat *Salmonella* infection.

## Introduction

The high incidence of multidrug-resistant salmonellae and invasive nontyphoidal salmonellae (NTS) highlights the need to develop preventative and therapeutic strategies to control these infections. There is still no efficacious subunit vaccine against NTS *Salmonella* in humans [1,2] that stimulates cellular and humoral adaptive immune responses since both are required for *Salmonella* clearance [3,4]. Pathogen-specific CD4^+^ Th1 cells are essential in driving protective immunity against *S*. *enterica*, and these cells secrete IFN-γ to activate macrophages for the elimination of the intracellular bacteria [5]. Although there are multiple regulatory levels of host immunity, small extracellular vesicles (EVs), such as exosomes, represent a promising defense line against pathogens [6–16], but their role in immunity against bacterial infections remains mostly unknown. Exosomes are nano-sized EVs ranging from 30 nm to 120 nm in size [17], and characterized by the surface rich in tetraspanins (CD9, CD63, CD81) and specific biogenesis [18]. The early endosomes mature into late endosomes, further generating intraluminal vesicles (ILVs) by endosomal membrane invagination. These ILVs are liberated into the extracellular milieu as exosomes by the fusion of MVBs with the plasma membrane [19]. Exosomes carry cargo such as proteins, metabolites, miRNA, and lipids [20–22], and these biological contents can be transmitted to the target cells even at a significant distance since exosomes can travel in physiological fluids [23]. The various functions of host-derived small EVs have not yet been well understood in the context of bacterial infections. Cells infected with *Mycobacterium tuberculosis* produce exosomes containing mycobacterial antigens, inducing protective immune responses in the vaccinated animals [12]. Exosomes can also serve as decoys to capture bacterial toxins, as shown for Gram-positive pathogen *Staphylococcus aureus* [24]. These vesicles potentially support both innate and adaptive responses to pathogens, yet it is unknown whether exosomes affect immune defenses directed towards gram-negative infections by eliciting humoral or T cell-mediated responses against these bacterial pathogens.

In this study, we identified protein and metabolite cargo of small EVs formed during *Salmonella* infection, demonstrating that exosomes derived from *Salmonella*-infected macrophages transmit bacterial antigens and other molecules that lead to M1 polarization of macrophages and cause an increase in PGE2 biosynthesis. Exosomes derived from infected macrophages were delivered intranasally to BALB/c mice to study whether exosomes can lead to a stimulation of mucosal immunity at a location distal to the place of injection. The intranasal route of EV delivery was used for several reasons. First, *Salmonella* Typhimurium causes systemic infection in mice, and second, the nasal route is less destructive to the administered antigen and elicits robust systemic and mucosal immunity, therefore providing an easier model for the studies of mucosal immunity [25,26]. Exosomes generated by infected macrophages indeed stimulated alveolar APCs in the lung mucosa within 24 hours post-administration, specifically leading to increased subsets of DCs, such as CD11b+ DCs in the mucosal tissues. Exosomes transmitting the immunogenic cargo were also capable of stimulating mucosal immunity in the murine NTS model by inducing antigen-specific Th1-type responses against *Salmonella*. In addition, exosomes also generated a robust anti-*Salmonella* humoral response in BALB/c mice shown by the production of anti-*Salmonella* IgGs, such as anti-OmpA IgG. In summary, exosomes stimulate the innate and adaptive responses targeting intracellular pathogens, such as *Salmonella*. An improved understanding of host EV’s function in immune responses against bacterial pathogens will provide a conceptual framework for the development of preventative or therapeutic strategies against *Salmonella* and improve our knowledge of the host-pathogen battle in response to *Salmonella* infection.

## Results

### The protein cargo of small EVs derived from *Salmonella*-infected macrophages

**Fig 1:**
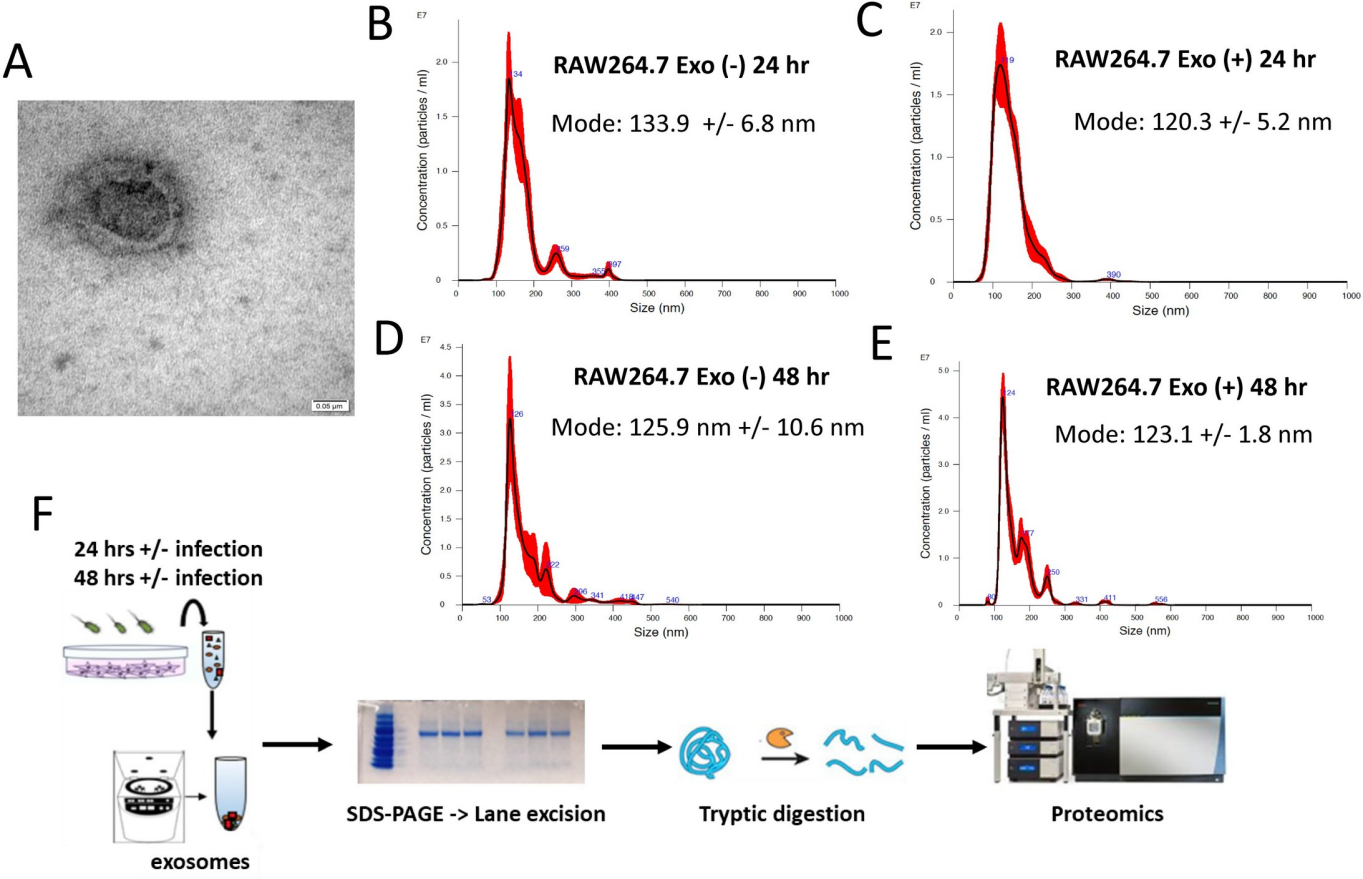
**(A**). **Morphology of exosomes derived from infected macrophages.** Exosomes derived from *S*. Typhimurium-infected RAW264.7 macrophages were isolated by differential ultracentrifugation described previously [6] and visualized under TEM. **(B)**-**(E). NanoSight Tracking Analysis of exosomes derived from RAW264.7 macrophages left uninfected and infected with *S*. Typhimurium.** Exosomes were isolated from RAW264.7 macrophages, which were left uninfected or infected with wild type *S*. Typhimurium (UK-1) for (B-D) 24 or (E-F) 48 hours. **(F). Schematic of proteomic analysis of exosome content.** Exosomes derived from *S*. Typhimurium-infected RAW264.7 macrophages (24 and 48 hpi) were isolated by differential ultracentrifugation as described previously [6]. Equal protein samples in triplicates were subjected to protein extraction and separation by SDS-PAGE. Protein bands excised from each lane were reduced, alkylated, and digested with trypsin. Peptide pools were desalted by C18 columns and analyzed by liquid chromatography-Orbitrap Fusion tandem mass spectrometry.

Our previous study revealed that small EVs released from *Salmonella* Typhimurium-infected macrophages led to a TLR4-dependent release of TNF-α from naïve macrophages treated with these vesicles [6]. The treatment of EVs with Protease A indicated that vesicular proteins are crucial to the proinflammatory function of these EVs upon infection [6]. We performed shotgun proteomic analysis of exosomes released from RAW264.7 macrophages upon *S.* Typhimurium infection at 24 and 48 hours, compared to uninfected cells. This proteomics approach was used to identify host and bacterial proteins carried by these host-derived EVs. The EVs had a donut-like shape when visualized by the electron microscopy (**Fig 1A**) and a size consistent with the characteristics of exosomes (**Fig 1B–1E**).

**Fig 2:**
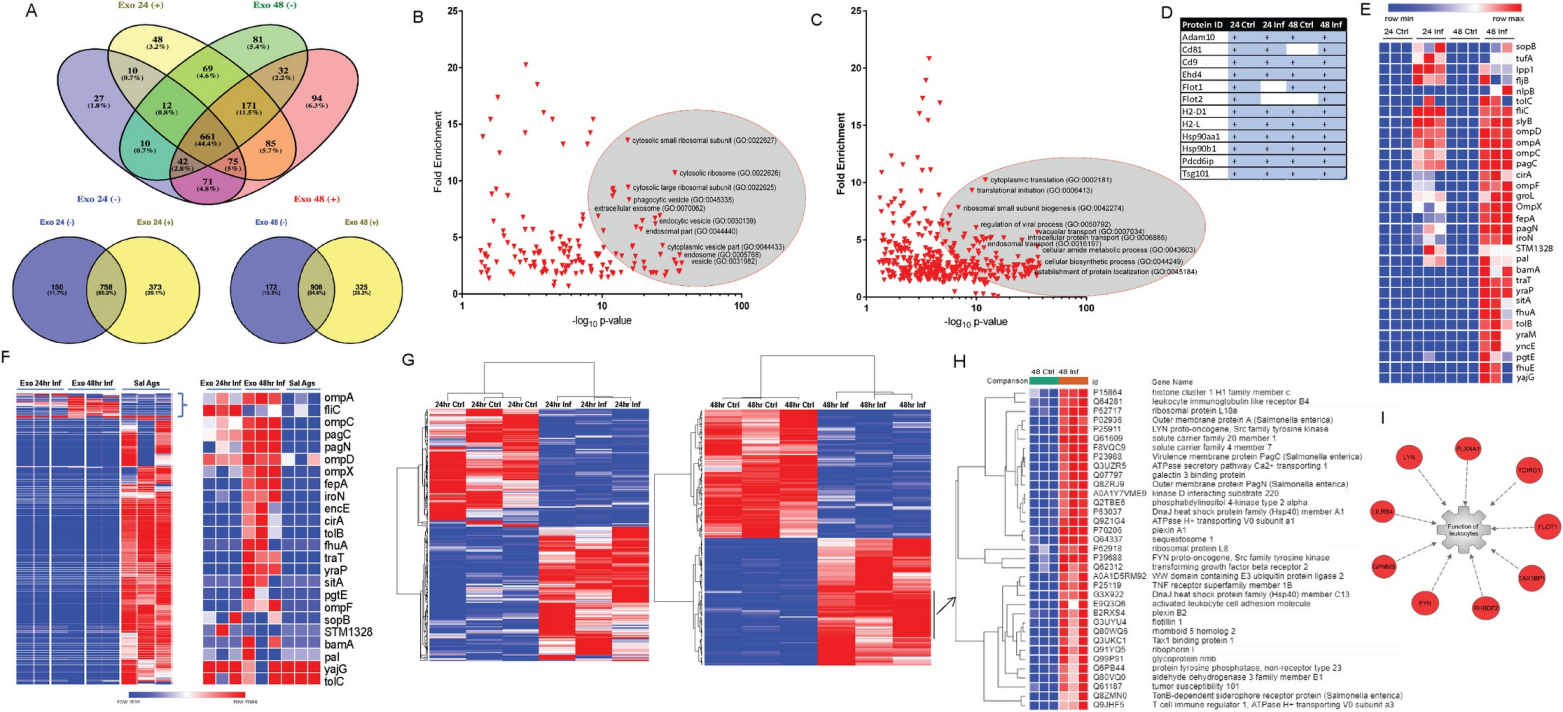
Proteomic analysis of exosomes isolated from *S*. Typhimurium-infected macrophages. **(A).** Venn Diagrams of the number of proteins identified in exosomes derived from macrophages infected for 24 [Exo 24 (+)] and 48 hours [Exo 48(+)] with *S*. Typhimurium in comparison to exosomes from uninfected cells [Exo 24 (-)] and Exo 48(-)] isolated at the same time points. The graphs show confidently identified proteins in at least two out of three replicates. (**B-C**). Scatterplot of GO terms associated with exosomal proteins identified at 48 hpi in at least two replicates. GO terms were analyzed using the PANTHER overrepresentation test. GO terms defining subcellular localization (B) and biological function (C) are both shown. **(D).** Identified exosomal markers in each category of samples. **(E).** Heat map of *S*. Typhimurium antigens identified in exosomes derived from macrophages infected for 24 (24 Inf) and 48 hours (48 Inf) with *S*. Typhimurium in comparison to exosomes from uninfected cells (24 Ctrl and 48 Ctrl) isolated at the same time points. **(F).** Heat map of *S*. Typhimurium antigens identified in exosomes at 24 hpi and 48 hpi compared in relative intensities to the proteins identified in the total *Salmonella* antigen preparation (Sal Ags). **(G).** Heat map of hierarchical clustering analysis of exosomal proteins, which were differentially expressed in exosomes from 24 and 48-hour infection meeting fold change of -2/+2. Proteins, which were not significantly altered upon infection, are not included in the graph. **(H-I).** Hierarchical clustering analysis of exosomal proteins identified in exosomes 48 hpi. Ingenuity Pathway Analysis was used to analyze one of the specific sub-clusters [indicated with an arrow in **(F)**]. The most significant represented pathway of this sub-cluster was related to the function of leukocytes. The red color indicated the upregulation of the molecules at 48 hpi in comparison to control.

By using high-throughput proteomic and metabolomic analysis (**Figs 1F and S4**), we confidently identified 1661 host and bacterial proteins, amongst which 661 proteins were identified in all the samples (**Fig 2A**). GO enrichment analysis identified that the subcellular localization of the identified proteins was related to vesicles, extracellular exosomes, phagocytic vesicles, and endocytic vesicles (**Fig 2B**). The biological processes represented by these proteins were related to translation, vacuolar transport, intracellular protein transport, endosomal protein transport, and biosynthetic processes (**Fig 2C**). Exosomal markers and general markers of EVs [27,28], were confidently identified in the vesicles obtained from infected and uninfected macrophages (**Fig 2D**).

Specifically, the analyzed EVs contained EHD-4, ADAM10, and TSG101 proteins [27], which are found explicitly in small EVs (exosomes). We also identified CD9, MHCII, Flotillin-1, CD81, and CD63 [27], and these markers are often found in the tetraspanin-positive EVs (**Fig 2D**). Finally, exosomes derived from infected cells contained *Salmonella* proteins (**Fig 2E**), including the SopB virulence factor [29]. SopB is an immunogenic protein, and the SopB DNA vaccine has shown promising results in the protection study [30]. Known immunogenic proteins were also identified, such as OmpA, OmpC, OmpD, IroN, FepA, CirA, or OmpF [31–33]. Several bacterial proteins were specifically enriched in the analyzed EVs compared with the proteomic analysis of the total *Salmonella* antigens (**Figs 2E, 2F and S1A–S1C**). While IroN, TraT, OmpC, PagC, OmpA, FepA, or CirA were confidently identified in EVs isolated from infected macrophages, these proteins were in the minority in the *Salmonella* antigen preparation or were completely not detectable by the proteomic analysis due to their relatively lower abundance in the bacterial lysate. Next, to understand the potential immunogenicity of exosome-enclosed *Salmonella* proteins, we analyzed the epitopes most likely displayed on MHC II from the list of identified proteins (**S2 Fig**) by IEDB (Immune Epitope Database and Analysis Resource) prediction tools for T cell epitopes. From the list of exosome-enclosed proteins, 84 candidate antigenic peptides were identified with IC_50_< 500 and percentile rank <1, suggesting that the identified *Salmonella* proteins have characteristics enabling their MHC II display, although these peptides would have to be synthesized and tested by using vaccinated animals to establish whether the cell-mediated immunity is due to these predicted epitopes.

The quantitative mass spectrometry analysis revealed that there were 373 exosomal proteins with the abundance altered at 24 hpi (hours post-infection) in comparison to uninfected control, and 325 proteins at 48 hpi (Fisher’s test p<0.05, fold change more significant than -2/+2, **Figs 2G, S1D, S1G and S1 and S2 Tables**). There were also differences in the protein content of exosomes formed at 24 hpi compared to 48 hpi (**S3 Table**). Unsupervised hierarchical clustering analysis of the differentially regulated proteins in infected versus uninfected EVs identified distinct clusters of exosomal proteins upregulated in EVs from infected cells (**Fig 2G and 2H**), such as the cluster containing proteins modulating the leukocyte function (FLOT1, FYN, GPNMB, LILRB4, LYN, PLXNA1, RHBDF2, TAX1BP1, TCIRG1; **Fig 2G and 2I**). The exosomal proteins with a protein level differentially regulated by infection were analyzed by pathway analysis tools to identify the mechanisms explaining up-or down-regulation of the identified proteins. The differentially regulated exosomal proteins at 48 hpi were involved in such processes as cellular function and maintenance (endocytosis), inflammatory responses, cell-to-cell signaling, and interaction (phagocytosis), as well as lymphoid tissue structure and development, or purine nucleotides de novo biosynthesis (**S3A and S3B Fig and S4 Table**). The host EVs produced during infection were enriched in proteins related to the clathrin-mediated endocytosis (**S3C Fig and S5 Table**), consistent with the previous studies [34–37]. We also used a metabolomics approach to identify metabolites within the same groups of exosomes and confidently quantified over 20 exosomal metabolites (**S4 Fig**). Apart from the proteins, exosomes also contained specific metabolites, such as citrulline, adenosine, AMP, GMP, and others, confirming some of the significantly enriched pathways detected by the proteomics (**S4A and S4B Fig)**.

### Exosomes from infected macrophages stimulate macrophage polarization and the COX2 pathway in target cells

**Fig 3:**
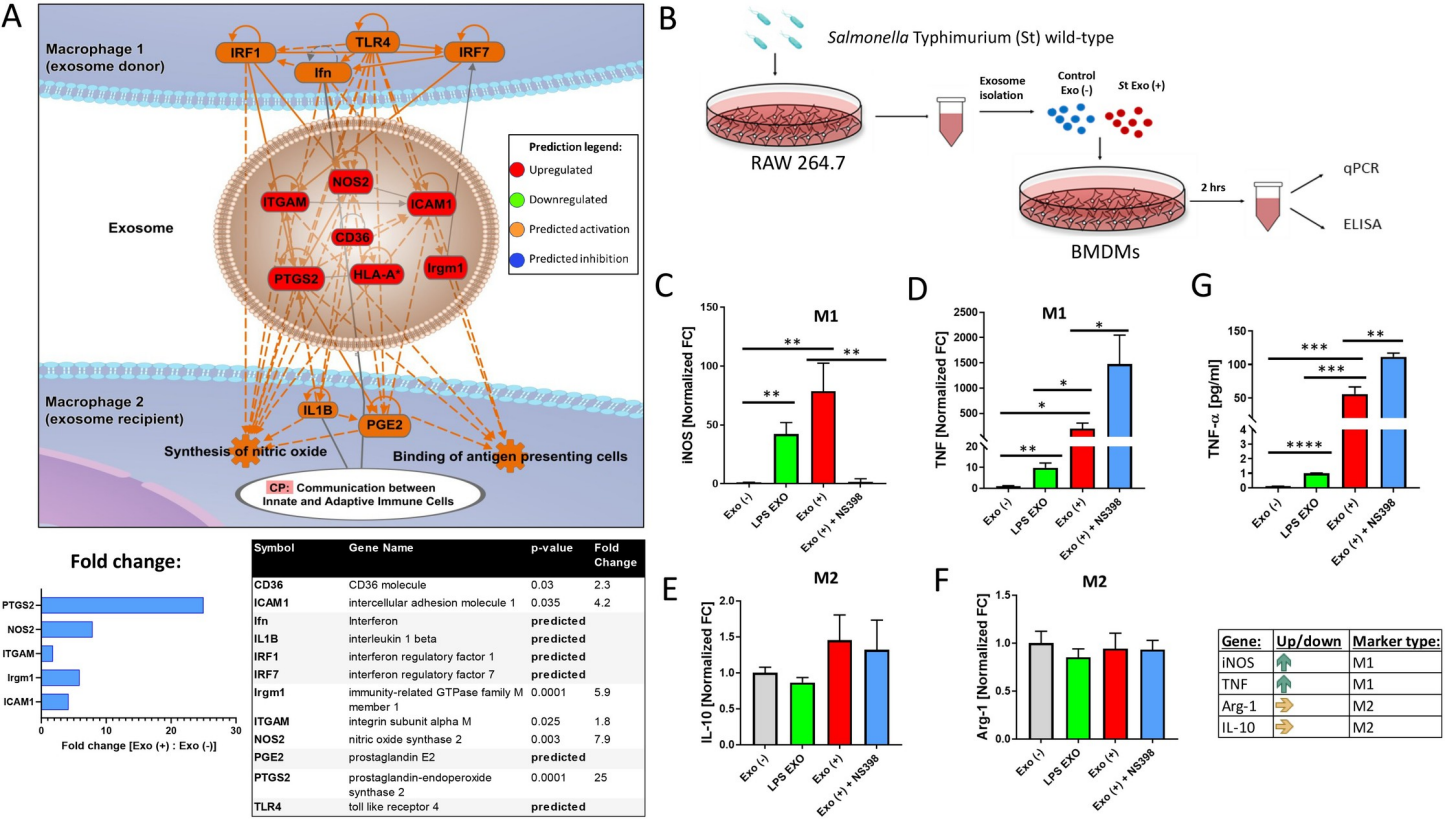
Predicted activation of pathways identified by network analysis. The exosomal proteins which were differentially regulated upon infection (48 hpi) were analyzed by Ingenuity Pathway Analysis software to understand the function of these proteins. **(A).** Network analysis identified predicted upstream TLR4 receptor activation and downstream activation of PGE2, nitric oxide synthesis, and binding of antigen-presenting cells. The red color of molecules indicated upregulation, and the orange color indicated predicted up-regulation. The table and graph that show the fold change (fold change was calculated between the abundance of proteins in exosomes derived from infected macrophages relative to proteins from exosomes derived from uninfected cells) are included. **(B)-(F).** The naïve BMDMs isolated from BALB/c mice were treated with exosomes obtained from *Salmonella*-infected or uninfected RAW264.7 macrophages or macrophages treated with LPS. Cell culture supernatant and cells were collected for ELISA-based analysis and qRT-PCR analysis, respectively. The cells were alternatively pre-treated with a COX-2 inhibitor (NS-398) to test whether the activation of macrophages happens downstream of this molecule predicted by proteomics (**Fig 3A**). The polarization markers for M1 **(C, D)** and alternative M2 phenotype **(E, F)** were analyzed by qPCR. Moreover, TNF-α release from treated naïve BMDM was also tested. Samples were normalized to GAPDH, and the relative abundance for each transcript is displayed. A t-test was used for statistical analysis (n = 3). *P*-values were indicated as follows: * p≤ 0.05; ** p ≤ 0.01; *** p≤ 0.001; **** p ≤ 0.0001.

The upstream pathway analysis identified the TLR4 receptor as a possible regulator responsible for increased NOS2, PTGS2, ICAM1, IRGM1, and other molecules in exosomes derived from infected macrophages (**Fig 3A**), consistent with the results of our prior study [6]. Based on the vesicular content (PTGS2, NOS2, Irgm1, and others, see **Fig 3A**), exosomes derived from the infected macrophages were predicted to stimulate the M1 polarization in standby macrophages, which were treated with these vesicles. The M1 macrophage polarization can be shown using markers such as iNOS and TNF-α, while M2 polarization by IL-10 and Arg-1 upregulation (reviewed in [38]). We tested the hypothesis that exosomes generated by macrophages during infection affect macrophage polarization by using the primary BMDM model. The naïve BMDMs were treated with exosomes obtained from infected or uninfected RAW264.7 macrophages. The cells were alternatively pre-treated with a COX-2 inhibitor (NS-398) to test whether the iNOS signaling pathway is induced by EVs from infected cells via the COX-2 enzyme, as predicted by our bioinformatic model (**Fig 3A**). Exosomes obtained from LPS-treated macrophages were used to understand whether any of these effects can be attributable to exosomes affected by a molecule that typically upregulates proinflammatory responses such as TNF-α. Exosomes isolated from *S.* Typhimurium-infected cells (24 hpi) stimulated the transcription of M1 polarization markers (iNOS or TNF-α) in BMDMs treated with these EVs, while the transcripts of M2 markers (Arg-1 or IL-10) were not affected by these EVs. The iNOS was regulated by exosomes from infected macrophages in a COX-2-dependent manner (**Fig 3C–3F)**, as we previously predicted (**Fig 3A**). Other M1 targets affected in BMDMs treated with EVs obtained from infected human THP-1 macrophages, which were used as an alternative model, were IL-1β, IL-12p40, and SOCS3, while other M2 markers remained either unchanged (MSR1), or down-regulated (MRC1) (**S5 Fig**). The release of prostaglandin E2 (PGE2) or COX2 transcription, a key enzyme responsible for PGE2 generation, was also increased in macrophages treated with exosomes derived from infected THP-1 cells, which dependent on the COX-2 activity, which can be inhibited pharmacologically (**S5 Fig**). This experiment confirmed our prediction that EVs from infected macrophages could upregulate PGE2 in the target cells (**Fig 3A**). Moreover, EVs obtained from LPS-treated cells led to an increase in the iNOS transcription to a similar degree as in cells treated with EVs from infected cells (**Fig 3C**), but the effect of EVs derived from LPS treatment was approximately 20-fold lower on TNF-α transcription (**Fig 3D**), and 50-fold lower on TNF-α secretion (**Fig 3G**). This observation suggests that the effects of EVs derived from infected cells are more complex than just the unspecific effect of LPS present in these exosomes [6]. While testing EVs from other infection models would be interesting, it is difficult to find a comparable infection model to *Salmonella* Typhimurium due to its unique pathogenesis.

### The function of exosomes derived from *Salmonella*-infected cells in the Th1 cell differentiation pathway

**Fig 4:**
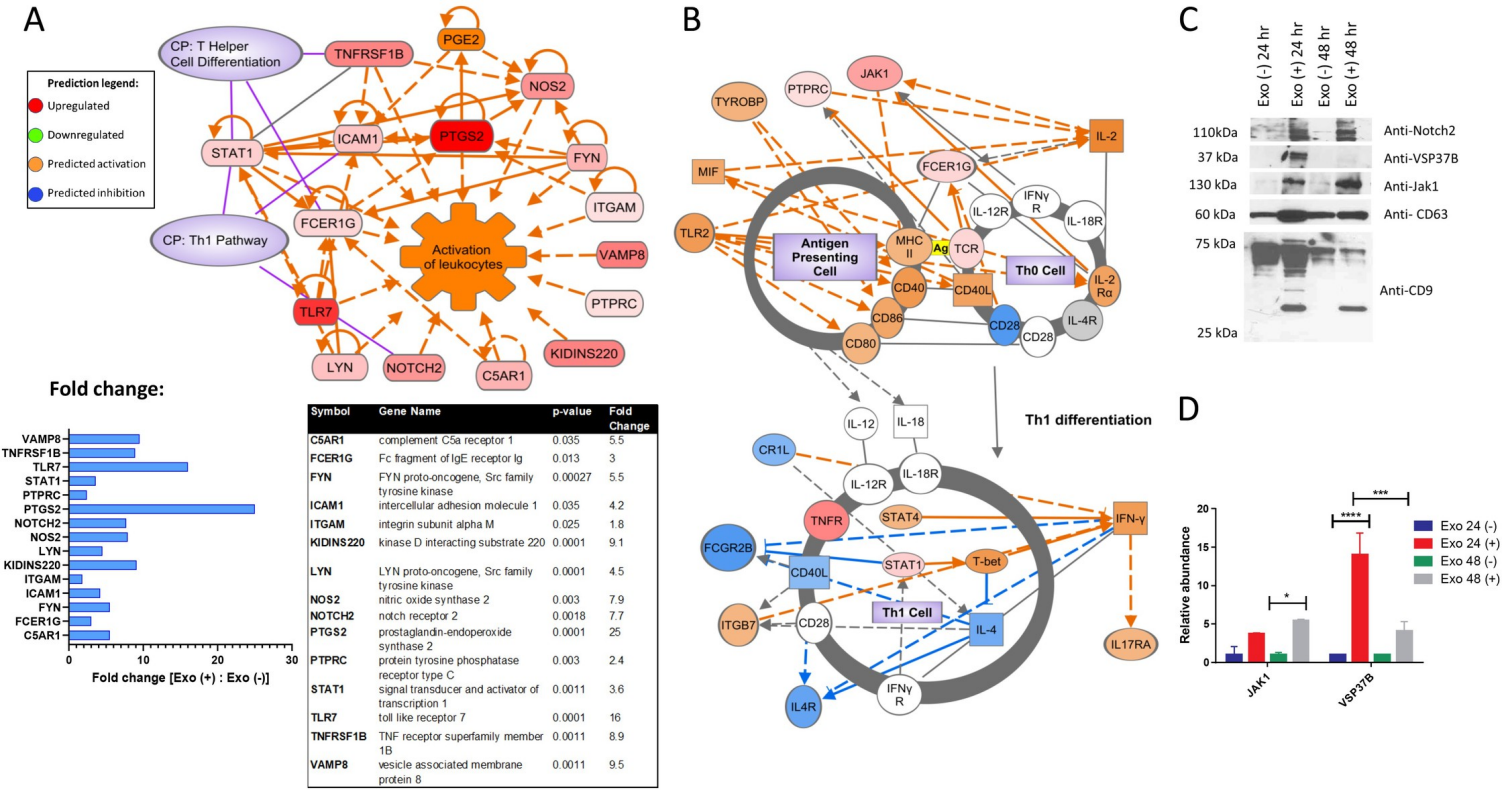
Predicted activation of pathways identified by network analysis. The exosomal proteins which were differentially regulated upon infection (48 hpi) were analyzed by Ingenuity Pathway Analysis software to understand the function of these proteins. **(A).** Network analysis of the exosomal proteins significantly altered by *S*. Typhimurium infection was linked to canonical pathways (CP) and predicted to cause activation of the leukocytes. The table and graph that show the fold change (fold change was calculated between the abundance of proteins in exosomes derived from infected macrophages relative to proteins from exosomes derived from uninfected cells) are included. **(B).** The canonical pathway (’TH1 differentiation’) of proteins identified as differentially regulated in exosomes at 48 hpi was by Ingenuity Pathway Analysis. The color scheme is explained in Fig 4A. **(C)-(D).** Validation of proteins identified by proteomics with a significant difference in the fold change. Validation was done by using an equal protein amount of exosomes collected from two different time points of infection (24 hpi and 48 hpi) by using western blot analysis. CD63 and CD9 exosome markers were also analyzed. The protein bands were then analyzed by ImageJ. Data are represented as mean ± SEM. A t-test was used for statistical analysis, and the p-values are displayed (n = 3). *P*-values were indicated as follows: * p≤ 0.05; ** p ≤ 0.01; *** p≤ 0.001; **** p ≤ 0.0001.

The top upregulated biological function of proteins in exosomes from infected cells compared to control was related to the Th1 cell differentiation pathway (**Fig 4B**). The identified molecules within the Th1 cell differentiation pathway were examined, where JAK1, PTPRC, TYROBP, and FCER1G proteins carried by exosomes were predicted to activate APCs (**Fig 4C**), followed by Th1 cell differentiation and IFN-γ release from Th1 cells in downstream pathways.

The presence of select exosomal proteins belonging to these pathways, including JAK1 and NOTCH2, were validated in exosomes by western blotting (**Fig 4C and 4D**). Inducible NOS2 was identified in exosomes from infected macrophages as well. NOS2 is a molecule involved in eliminating pathogens and has been previously identified in exosomes obtained from nasal mucosa [10]. STAT1 has also been identified in exosomes, and its presence in EVs could partially explain the activation of the naïve APCs exposed to these exosomes [39], such as an increase in RANTES [6].

### Biodistribution of exosomes delivered intranasally to BALB/c mice

The immunogenicity of exosomes from infected cells and predicted effect of exosomes on Th1 cell differentiation were tested *in vivo* in BALB/c mice. We used an intranasal (IN) mode of delivery of exosomes, which was selected based on the past studies demonstrating that IN immunization with *Salmonella* antigens is less destructive to the antigens than some other routes and that this route elicits robust systemic and mucosal immunity [25,26,40,41]. As an example, IN administration of the NTS vaccine consisting of microbubbles containing Ags has been successful in delivering the Ags to pulmonary DCs and inducing local and distant anti-*Salmonella* immune responses via the mucosal immune system [41]. While in this study, we did not aim to test exosomes generated by infected macrophages as a vaccine candidate, we were interested in the effects these EVs have on the stimulation of innate responses and mucosal pathogen-specific adaptive responses *in vivo*.

**Fig 5:**
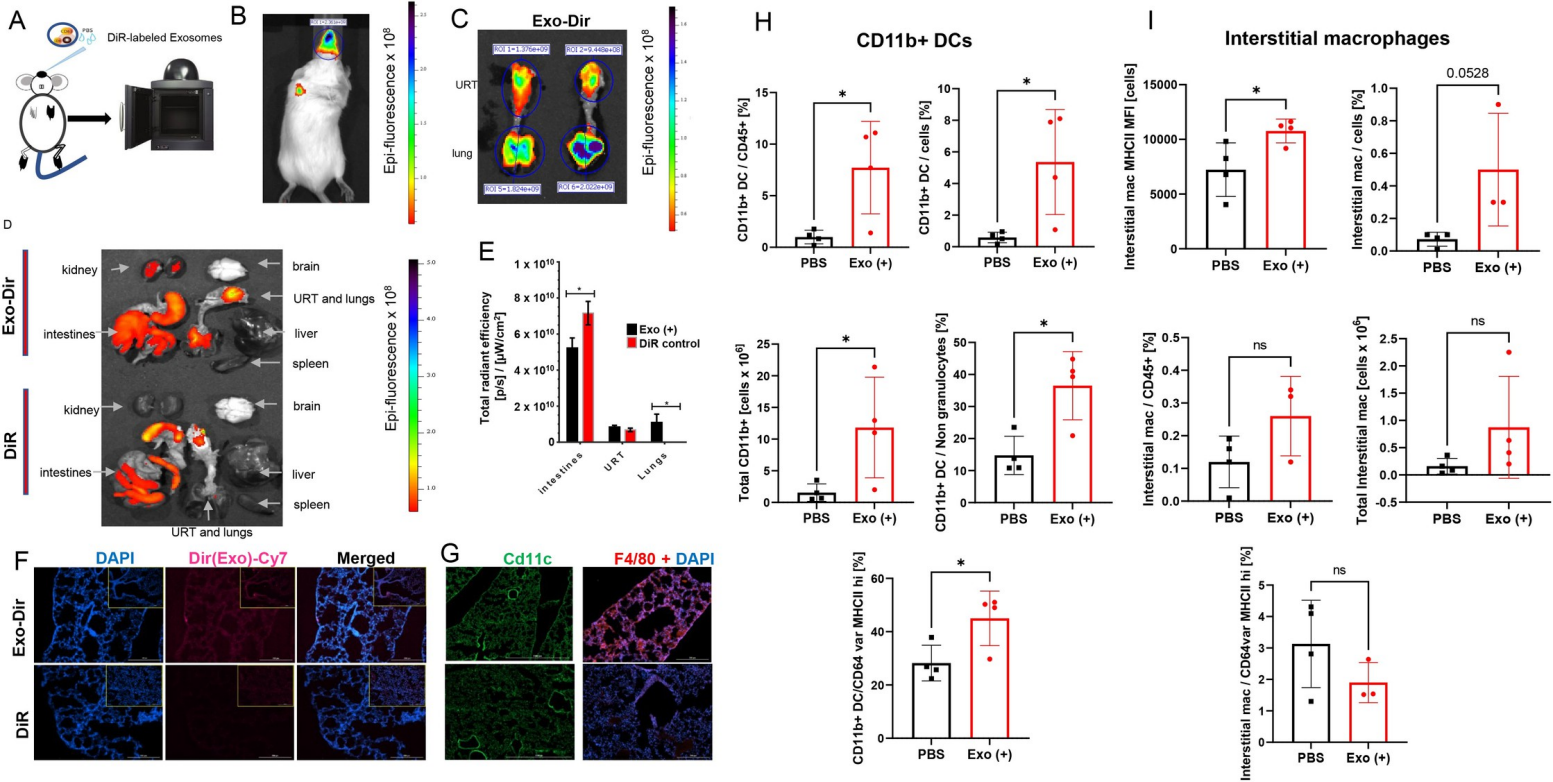
Biodistribution of exosomes derived from *Salmonella*-infected murine macrophages (RAW264.7 cells) administered intranasally to BALB/c mice. **(A)-(B).** Exosomes derived from *Salmonella*-infected macrophages were labeled with DiR (Exo-DiR), followed by washes to remove the free dye. A dose consisting of 20 μg exosomes was administered intranasally (I.N.) to female BALB/c mice. At 24-hours post-administration, mice were anesthetized and imaged under IVIS Spectrum In Vivo System (Perkin-Elmer, USA). The whole-body image is shown, demonstrating a successful intranasal delivery. **(C)-(E)** Mice (B) were euthanized, and individual organs were harvested and subjected to IVIS analysis. Exosomes in the upper respiratory (URT) and lungs were evident as fluorescence of DiR-labeled exosomes **(**Exo-DiR; **C, D).** For comparison, control mice received free DiR dye, which was also administered IN, and these animals underwent the same analysis (DiR). Other organs from control animals are also shown for comparison **(D)**. The total radiant efficacy was measured in intestines, URT, and lungs to quantify the Exo-DiR in individual organs (or free DiR dye). **(F).** Lung sections of mice immunized with Exo-DiR from *S*. Typhimurium-infected macrophages show exosomes in the lung regions, which are absent in the lungs of control animals receiving free DiR dye. DAPI staining was used to stain nuclei. **(G).** Lung sections of mice immunized with Exo-DiR from infected macrophages show the antigen-presenting cells’ infiltration to the pulmonary mucosa. Lung sections from mice were stained with anti-CD11c (FITC) or anti-F8/40 (APC) markers. **(H)-(I). Graphs showing myeloid cell populations in the lungs of BALB/c mice treated with exosomes derived from *Salmonella*-infected macrophages or PBS control.** Exosomes were isolated from *Salmonella*-infected macrophages by ultracentrifugation and stored in -80°C after further use. A dose consisting of 40 μg of exosomes was administered I.N. to female BALB/c mice. At 24-hours post-administration, mice were euthanized, and lungs collected for cell subpopulation analysis. Graphs show the percentage and numbers of CD11b^+^ DCs populations **(H)** or interstitial macrophages and their MHCII expression **(I)**. Data on graphs are represented as mean ± SD. The *p*-values were determined by t-test, and indicated as follows (n≥3): * p≤ 0.05; ** p ≤ 0.01; *** p≤ 0.001; **** p ≤ 0.0001.

The exosomes were labeled with DiR lipophilic dye to determine whether these vesicles can penetrate the nasal mucosa (**Fig 5A and 5B**). The exosomes were detectable in the nasal mucosa and lungs 24 hours post-application, predicted to promote mucosal immunity (**Fig 5C**). Moreover, exosomes were also present in the intestine and kidneys, while similarly delivered DiR dye control migrated primarily to intestines and upper respiratory tract (URT), where it was initially delivered (**Fig 5D and 5E**). The spleen and liver did not contain exosomes 24 hours post-application (**Fig 5D**), suggesting that the effects of exosomes on the initial innate response were local to the respiratory system. The specific migration of exosomes to lungs compared to free DiR dye (**Fig 5C–5E**) was confirmed by immunofluorescence (**Fig 5F**). Next, immunofluorescent staining was performed to visualize the presence of murine pulmonary macrophages and CD11c-positive DCs 24 hours post-administration of exosomes derived from *S*. Typhimurium-infected macrophages. Lung sections were stained with an anti-CD11c mAb, a DC marker, and an anti-F4/80 mAb to distinguish macrophages. Lung sections derived from mice administered 20 μg of exosomes derived from *S*. Typhimurium-infected macrophages showed increased infiltration of CD11c^+^ cells as well as F4/80^+^ macrophages (**Fig 5G**). These positive-staining cells were proximal to the airway mucus membranes of mice dosed with exosomes derived from infected cells, compared to control mice given the free DiR dye.

We next determined whether EVs from infected macrophages stimulate specific subsets of pulmonary innate immune cells, in which studies we focused on macrophages and DCs. In this model, the lung tissue from animals administered with EVs obtained from infected macrophages was evaluated 24 hours post-delivery (**S6 Fig**). Lungs of mice administered with exosomes generated by infected macrophages led to an increase in specific DC subpopulations. Specifically, subsets of CD11b^+^ DCs cells were significantly increased in proportion to other cell types, including Cd11b^+^ DCs cells expressing high MHC II levels (**Fig 5J**). Also, CD103^+^ DCs CD45^+^ cells were significantly increased upon EV delivery (**S7 Fig**). Pulmonary macrophages were also affected by the exosomes generated by infected macrophages. Interestingly, while the population of interstitial macrophages and specifically MHC^+^ interstitial macrophages was slightly upregulated in animals treated with EVs (**Fig 5I**), the alveolar macrophages were overall not significantly affected at the tested treatment and time point (**S7 Fig**). These results suggest that EVs derived from infected innate immune cells result in the infiltration of specific DCs and macrophages into the alveolar mucosa.

### Analysis of Th1-type responses stimulated by exosomes in vivo

**Fig 6:**
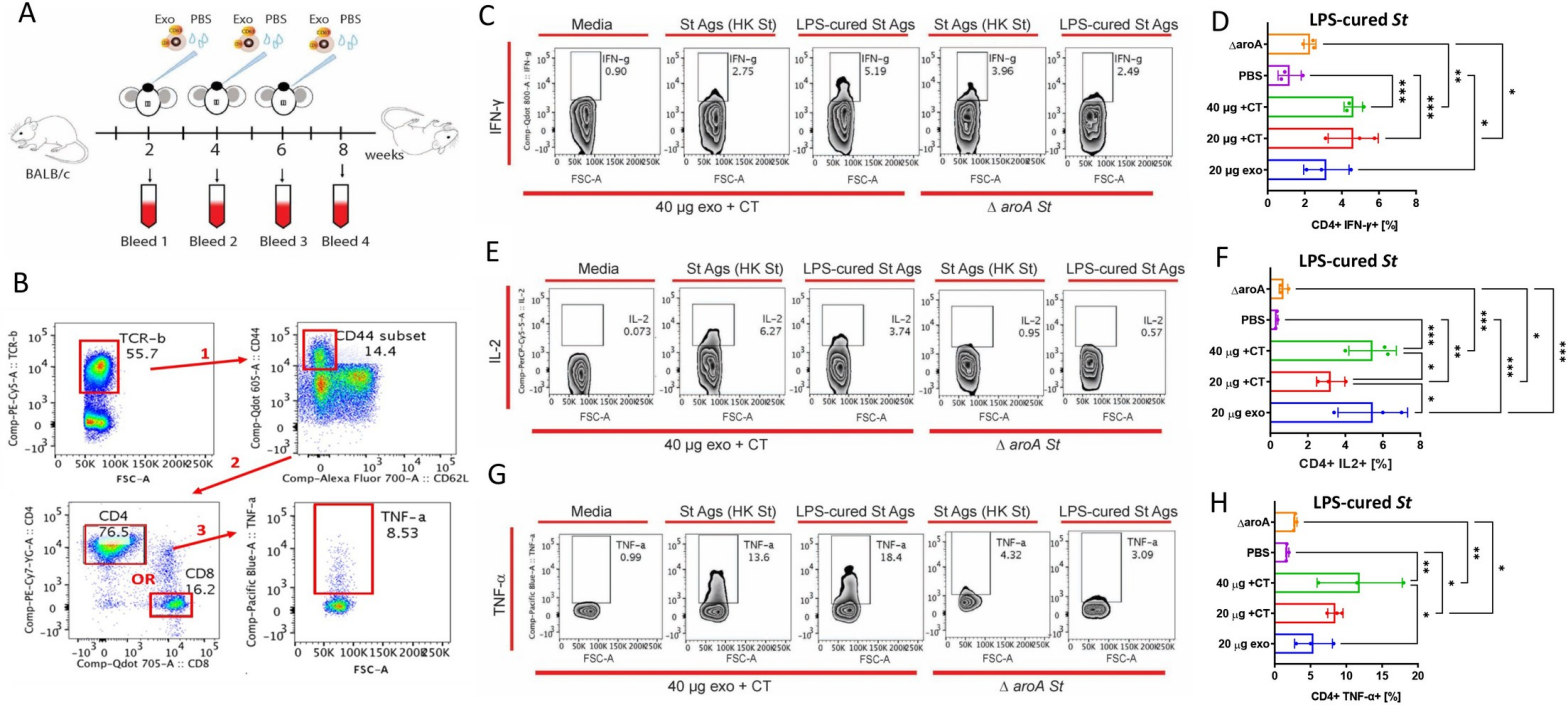
Exosomes derived from *Salmonella*-infected murine macrophages stimulate CD4^+^ T lymphocytes *in vivo*. **(A).** A schematic of the experiment. Mice (5/group) were IN immunized with 20- μg or 40- μg doses of exosomes derived from *Salmonella*-infected macrophages (Exo) in the presence or absence of cholera toxin adjuvant (CT) or MPL adjuvant. Other mice were immunized with live Δ*aroA S*. Typhimurium vaccine (χ9909). Two booster immunizations were administered in 2-week increments**. (B).** Splenocytes obtained from mice administered with exosomes, PBS, or Δ*aroA S*. Typhimurium were re-stimulated with media (control), whole *Salmonella* antigens, or LPS-cured *Salmonella* antigens [see **(A)** for regimen]. Splenocytes were stained as described in the individual figures below. FACS analyzed pools of indicated CD4+ or CD8+ cells producing specific cytokines in the following manner: memory T-lymphocytes were distinguished from naïve T-lymphocytes by gating for effector T-lymphocyte phenotype (TCR-β^+^ CD44^+^ CD62L^-^). Next, effector T-lymphocytes are characterized phenotypically by high expression levels of CD44+ hi and CD62 lo, in which we subsequently gated for CD4+ (or CD8+) specific T-lymphocytes and then CD4+ (or CD8+) T-lymphocytes secreting appropriate cytokines as indicated on figures. **(C)-(H).** Mice were exposed to conditions indicated above **(A)**. FACS analysis **(B)** of CD4+ cells was performed on splenic mononuclear cells treated with heat-killed *Salmonella* Ags, LPS-cured *Salmonella* Ags, or media (control). Cells were stained intracellularly for IFN-γ **(C, D)**, IL-2 **(E, F)**, and TNF-α **(G, H)**. Zebra plots show individual analyses (**C, E, F**), where the antigen used for cell re-stimulation is indicated on the top of the graphs while the source of splenocytes is indicated on the bottom of the figures. **(D), (F), (H).** The summary graphs showing the percentage of CD4+ cells that express IFN-γ, IL-2, or TNF-α upon restimulation with *Salmonella* Ags devoid of LPS. Data on graphs are represented as mean ± SD. The *p*-values were determined by ANOVA and indicated as follows (n = 3): * p≤ 0.05; ** p ≤ 0.01; *** p≤ 0.001; **** p ≤ 0.0001.

We next examined whether exosomes generated by infected cells stimulate antigen-specific CD4^+^ or CD8^+^ T cell responses. Following antigen re-stimulation of T cells with *Salmonella* antigen to test for pathogen-specific responses, intracellular staining for IFN-γ, TNF-*a*, IL-2, IL-10, and IL-17 was done in these cell subsets. IFN-γ was chosen as one of the cytokines, as this molecule is induced in mice in a *Salmonella* model, which directly correlates with immune protection with this live vaccine [42]. Doses containing 20-μg (~0.75 x 10^10^ exosomes) or 40-μg (~1.5 x 10^10^ exosomes) exosomes derived from infected macrophages were delivered by the IN route in the presence or absence of adjuvant (monophosphoryl lipid A, MPL; or mucosal adjuvant, cholera toxin, CT) to 8-wk female BALB/c mice (3–5 mice per group). Two booster administrations of exosomes were done at two-week intervals over eight weeks (**Fig 6A**). PBS and Δ*aroA* attenuated *Salmonella* strain were used as controls, although the results with Δ*aroA Salmonell*a had a different delivery route (oral route) than exosomes (intranasal route). The splenocytes were collected and incubated with media (control) or with *Salmonella* antigens (Ags), including LPS-cured Ags [43]. The percentages of Ag-specific CD4^+^ T and CD8^+^ T cells producing specific cytokines were evaluated by flow cytometry. T cells were gated for TCR-ß expression and further subdivided into naïve and effector T cell phenotypes based on the expression of CD44 and CD62L. Naïve T cells are CD44^lo^, whereas effector or memory T cells are CD44^hi^. The population of CD44^hi^ T cells was further examined for CD62L^lo^ expression, representing activated effector T cells, as opposed to CD62L^hi^ CD44^hi^ T cells, representing resting effector T cells. Activated effector T cells were measured for both CD4^+^ and CD8^+^ T cells (**Fig 6B**). Percentages of Ag-specific CD4^+^ T and CD8^+^ T cells producing IFN-γ, IL-2, TNF-α (**Figs 6 and S8–S11**) or IL-10, and IL-17 (**S8 and S9 Figs**; IL-10 was not shown as it was not detected) were measured.

Strikingly, BALB/c mice administered with exosomes stimulated recall responses against *Salmonella* Ags, in some cases more significantly than live Δ*aroA Salmonella* strain. Different delivery routes have to be noted for exosomes and Δ*aroA*. Therefore, direct comparison is not possible. Splenic lymphocytes from mice administered with exosomes derived from infected macrophages and/or with CT adjuvant had a significant population of IFN-γ CD4^+^ T cells compared to PBS control when re-stimulated with *Salmonella* Ags (**Figs 6C, 6D and S8**). Similarly, in comparison to the PBS control, the population of IL-2-producing CD4^+^ T cells was also significantly increased with splenic mononuclear cells obtained from mice vaccinated with exosomes in the presence or absence of CT (**Figs 6E, 6F and S8 Fig**). Splenic mononuclear cells obtained from mice immunized with exosomes in the presence or absence of CT had a statistically significant increase in the percentage of TNF-a-producing CD4^+^ T cells (**Figs 6G, 6H and S7**). Finally, an increase in IL-17- and IFN-γ-producing CD8^+^ T or IL-17-producing CD4^+^ and CD8^+^ cells was identified in mice groups administered with exosomes (**S8C and S8D Fig**).

CT adjuvant was used in some treatments in conjunction with exosome treatment to stimulate the basal immune response. CT did not have a statistically significant effect on the increase of CD4^+^ TNF-α ^+^ cells or CD4^+^ IFN-γ^+^ cells compared to exosome treatment alone, but it leads to a rise in CD4^+^ IL-2^+^ cells (**Fig 6**). Finally, we ensured that the activation of these cells was not due to mitogen-mediated effects since replacement of *Salmonella* Ags with media did not lead to significant upregulation of CD4^+^ IFN-γ^+^ cells amongst the splenocytes obtained from mice treated with exosomes in comparison to PBS control (**S9 Fig**).

Overall, these data show that exosomes might play immunomodulatory functions since splenic mononuclear cells isolated from mice immunized with exosomes derived from *Salmonella*-infected antigen-presenting cells produce a similar, if not higher, percentage of CD4^+^ T cells secreting Th1-type cytokines compared to the Δ*aroA Salmonella* strain in response to *Salmonella* Ags. The effects of the exosomes on the memory phase were not tested in this study because we were focused on the immediate impact of exosomes on the immune responses in the effector phase. We conclude that the recall response of mice administered with exosomes derived from *Salmonella*-infected macrophages shows a pathogen-specific Th1 cell bias against *Salmonella* antigens, thus validating our earlier prediction model (**Fig 4A and 4B**).

### Exosomes derived from infected macrophages stimulate the production of anti-*Salmonella* antibodies in mice

**Fig 7:**
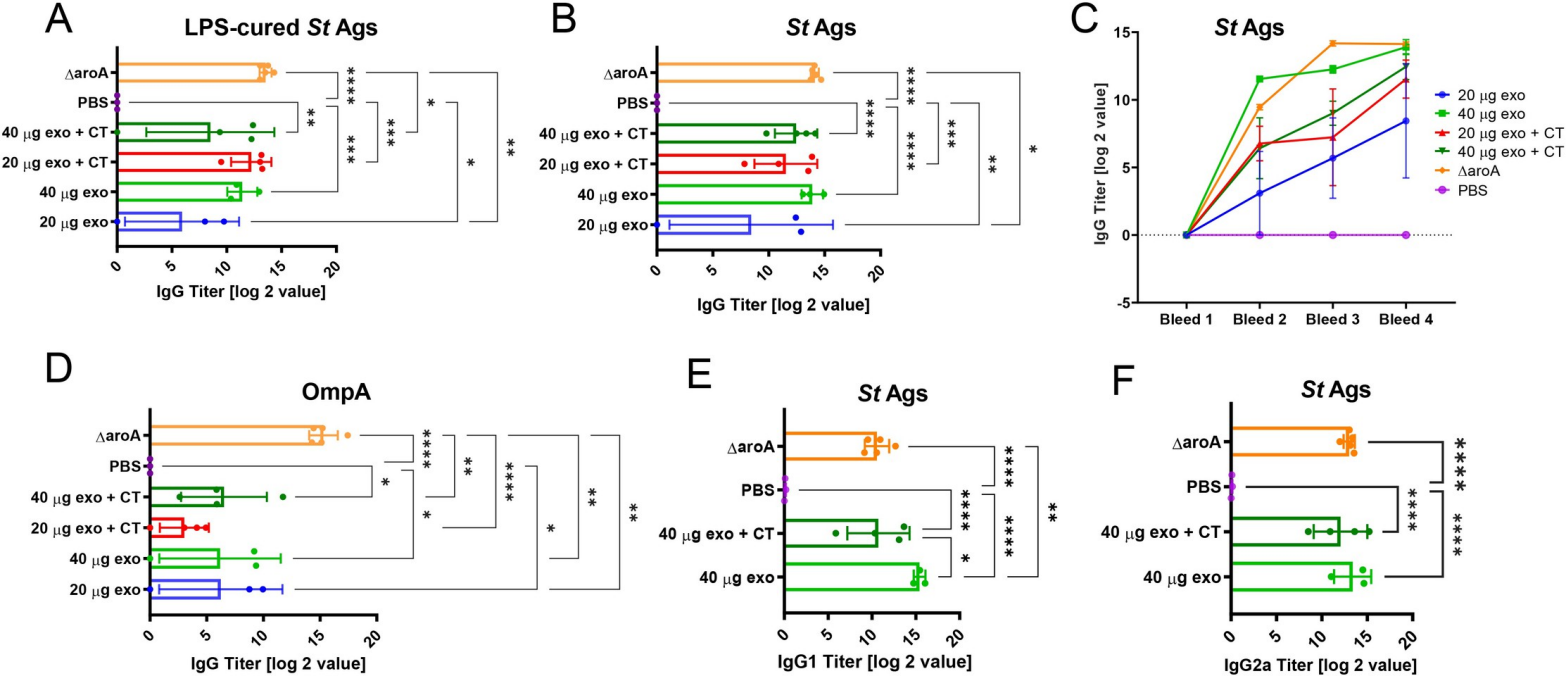
Exosomes derived from *Salmonella*-infected murine macrophages induce the production of *Salmonella*-specific antibodies in vivo. **(A-C).** Serum from mice was obtained one week after the first booster (see **Fig 6A**). The anti-*Salmonella* antibodies in sera were evaluated by ELISA by using LPS-cured antigens (A), whole *Salmonella* antigens (B), or OmpA recombinant protein (C). Titer shows serial dilutions of sera (measured at OD_415_ = 0.2), indicating the presence of anti-*Salmonella* antibodies. **(D).** The titer of serum from mice from all bleeds against whole *Salmonell*a antigens (Ags). **(E-F).** The antibody titer of serum from mice at bleed 4 (see **Fig 5A**) showing different isotypes of IgG detected in serum. For all graphs, the data are represented as mean ± SEM. The *p*-values were determined by one-way ANOVA. The *p*-values were indicated as follows: * p≤ 0.05; ** p ≤ 0.01; *** p≤ 0.001; **** p ≤ 0.0001.

Sera acquired from mice immunized with exosomes derived from *S*. Typhimurium-infected macrophages, PBS (negative control), or Δ*aroA S*. Typhimurium (positive control) were analyzed for the presence of anti-*Salmonella* antibodies, including total IgG, IgG subclasses IgG1 and IgG2a, and IgM. The titers of antibodies against whole *Salmonella* Ags, LPS-detoxified *Salmonella* Ags, and recombinant OmpA were evaluated by ELISA, followed by Four Parameter Logistic curve fitting (**Figs 7 and S12**). Mice treated with exosomes with or without CT exhibited significant antibody responses against whole *Salmonella* Ags and LPS-detoxified *Salmonella* Ags compared to the live attenuated vaccine strain. Administration of 40 μg of EVs resulted in the same terminal antibody titer as for the Δ*aroA S*. Typhimurium vaccine, despite a different delivery route for EVs (intranasal route) and Δ*aroA S*. Typhimurium (oral route) (**Fig 7A–7D**).

Interestingly, the antibody production in mice administered with exosomes was not skewed towards anti-LPS responses since the titers of antibodies against the LPS-cured *Salmonella* Ags were comparable to titers obtained with the Δ*aroA S*. Typhimurium vaccine (**Figs 7A and 7B and S8A**). Moreover, the IgGs against LPS-cured *Salmonella* Ags in these mice were comparable to the antibodies against non-LPS-cured *Salmonella* Ags (**Fig 7**). The presence of antibodies against recombinant OmpA was also examined in the sera since OmpA is a highly immunodominant (but not protective) Ag in mice [31]. Mice immunized with exosomes had a significantly lower level of antibodies against OmpA compared to the live vaccine strain (**Figs 7D, S8B and S8C)**, although the total amount of antibodies against whole *Salmonella* Ag was similar between mice treated with exosomes and Δ*aroA S*. Typhimurium (**Fig 7A–7C**).

Finally, we analyzed immunoglobulin subtypes in sera of animals administered with exosomes and attenuated *Salmonella* strain. Anti-*Salmonella* IgM antibodies were not detected at measurable levels in the first bleed, which could be due to isotype switching occurring before the first bleed. In terms of different isotypes of IgGs, both IgG1 and IgG21 antibodies were robustly present in all animal groups immunized with exosomes with or without the CT adjuvant. IgG1 antibodies were significantly higher in mice immunized with EVs derived from infected macrophages than the live Δ*aroA S*. Typhimurium vaccine (**Fig 7E and 7F**). However, there were no differences in the anti-*Salmonella* IgG2a antibody levels between mice immunized with exosomes or the attenuated *Salmonella* strain (**Fig 7E and 7F**).

In summary, the EVs derived from infected macrophages generate a unique antibody response in animals, which has a lower antibody titer against OmpA than animals vaccinated with the live attenuated strain (**Fig 7D**). This observation could be explained by the fact that exosomes deliver a unique set of Ags compared to the live Δ*aroA S*. Typhimurium vaccine (**Fig 2F**). Based on a proteomic experiment comparing the Ags present in the exosomes from infected cells (24 and 48 hpi) to the total *Salmonella* Ags, the exosomes deliver a uniquely composed set of Ags, which are not the most abundant *Salmonella* proteins present in the Ag preparation. Further studies are needed to determine the antibody responses against other specific Ags in mice administered with exosomes derived from infected macrophages.

## Discussion

An effective NTS vaccine must induce the antibody responses via Th1 cell support for adequate clearance of bacteria [3,4], and the majority of tested NTS vaccines lack adequate antigen-specific antibody responses and antigen-specific T cell responses. For example, stimulation of humoral immunity by NTS leads to the production of antibodies, which predominantly include non-protective antibodies [31,44]. Therefore, the protective NTS vaccine design will be greatly assisted by an improved understanding of previously overlooked aspects of host immune mechanisms. One of the new mechanisms currently explored as a route of antigen trafficking by immune cells is the exosome-based trafficking of antigens. Exosomes are eukaryotic nano-sized extracellular vesicles, which serve as mediators of intracellular responses and, as such, are expected to contribute to the defense mechanisms against pathogens [6–14]. The extracellular signals produced by immune cells are critical in promoting effective host responses against bacterial pathogens and enable communication between the infected and the uninfected cells [45]. The minuscule size of exosomes and the molecules decorating their surface is one of the reasons why these vesicles are efficiently taken up by cells by such mechanisms as clathrin- and caveolae-mediated endocytosis [46,47]. Exosomes encapsulate and transmit biological cargo to target cells, including proteins, metabolites, miRNAs, and lipids [20–22]. Antigens (Ags) are possible exosomal contents, and Ag-containing exosomes were shown to trigger an anti-tumor immune response [48]. Moreover, infected host cells can "smuggle" antigens to local DCs, which has been demonstrated for the acid-fast bacterium, *M*. *tuberculosis* [14,49]. Exosomes potentially support both innate and adaptive responses to pathogens, but the effect of exosomes and their encapsulated cargo on specific humoral or T cell-mediated responses against gram-negative pathogens is entirely unknown. Our group has previously shown that *S*. Typhimurium-infected macrophages and DCs produce exosomes, which function as "molecular telephones" in promoting the innate immune responses, triggering the TLR4-dependent release of TNF-α from naive macrophages and DCs, but also the secretion of T cell-attracting cytokines [6]. While these functions of exosomes on innate immune responses could be explained by the presence of LPS in exosomes during infection [6,50], several lines of evidence suggest that the protein component of exosomes is crucial in the stimulation of naïve cells [6]. We hypothesized that *Salmonella* proteins and host factors are trafficked to exosomes and transmitted to the recipient cells to stimulate innate and *Salmonella*-specific adaptive immunity.

Here, we demonstrated that exosomes derived from *S*. Typhimurium infected-macrophages carry multiple proteins involved in proinflammatory functions, trafficked by exosomes upon infection. One of the protein functions expected to be altered in cells treated with exosomes was iNOS upregulation, which is involved in the antibacterial macrophage function. Indeed, in vitro data showed that exosomes lead to the M1 polarization in BMDMs treated with these vesicles, which are, for instance, characterized by increased production of iNOS. We also showed that EVs from infected macrophages stimulate specific subsets of pulmonary innate immune cells delivered in a mucosal manner. In particular, exosomes from infected cells stimulated APCs such as CD11b^+^ DCs cells, particularly the cells expressing high MHC II levels and subsets of CD103^+^ CD45^+^ DCs. CD103+ DCs are of particular importance as this cell subset is equipped with specific abilities to tackle both the uptake, transportation, and presentation of bacterial antigens, including salmonellae antigens [51]. These results suggest that EVs derived from infected innate immune cells result in infiltration of specific DCs in the alveolar mucosa, and future studies should establish whether these EVs also assist in the antigen uptake by DCs.

The pathway analysis of exosomal proteins also generated the hypothesis that exosomes from infected macrophages activate adaptive Th1 cell-biased immunity. Infection with NTS serovars is known to stimulate an enhanced Th1-type bias by CD4^+^ or CD8^+^ T cells associated with increased production of proinflammatory cytokines IFN-γ, IL-18, IL-12, IL-15, and TNF-α [52]. Hence, we studied whether exosomes derived from infected cells and delivered to BALB/c mice stimulate bias towards *Salmonella*-specific Th1 or Th2 cell responses. Previous studies indicated that small EVs, mostly consisting of exosomes, support Th1 cell responses, while large EVs support Th2-type responses [27,53,54]. Our data confirm that after intranasal administration, exosomes localize to the alveolar mucosa and stimulate CD4^+^ T cell-mediated IFN-γ, IL-2, or TNF-α release, and that these responses are directed against *Salmonella* antigens, based on the re-stimulation experiments. These data suggest that exosomes derived from *Salmonella*-infected cells prompt a Th1-type response and increase *Salmonella*-specific CD4^+^ T cell responses. In addition to cell-mediated immunity necessary for intracellular control of infection [55], *Salmonella* induces antibody responses via Th1 cell support to target extracellular bacteria [3,4]. The production of antibodies to control *Salmonella* infection is essential for bacterial clearance, but in NTS immunization with attenuated strains, these antibodies are predominantly non-protective as they might be targeting "decoy" antigens [31,44]. Therefore, it is essential to identify new mechanisms for the trafficking of protective antigens in immune cells. Therefore, it is essential to identify new mechanisms for the trafficking of protective antigens in immune cells. In this study, we identified immunogenic pathogen-encoded proteins in exosomes formed during infection and observed enrichment of such proteins as OmpA, OmpC, OmpD, IroN, FepA, CirA, or OmpF in these exosomes. OmpA is highly immunogenic [31], while IroN, CirA [32], and OmpD [33] confer protective immunity in a murine model of salmonellosis. The enrichment of IroN and CirA in exosomes is particularly intriguing since *in vivo* expression level of these proteins is typically beyond the detection level. Moreover, antibodies against IroN and CirA are often not readily detected due to their low abundance [32,56]. Consistent with these observations, exosomes also contributed to the increased production of anti-*Salmonella* antibodies. Antibody response to soluble protein and membrane protein Ags can induce IgG1, whereas bacterial capsular polysaccharide antigens can induce IgG2a. Both soluble and membrane proteins are in exosomes based on our data, and previous findings indicate that exosomes contain LPS, a polysaccharide Ag [6]. Antibodies against OmpA were present in animals administered with exosomes, although other anti-*Salmonella* antibodies were more represented than anti-OmpA, based on the comparison of the antibody titers. Because protective antigens (CirA, IroN) were identified as cargo of exosomes, likely, antibodies against these proteins are also stimulated by exosomes, which should be tested in the future. In summary, exosomes contribute to the humoral responses against *Salmonella* in the murine model of NTS.

To summarize, proinflammatory exosomes released from antigen-presenting cells upon *Salmonella* Typhimurium infection stimulate innate and adaptive immune responses, specifically triggering Ag-specific IFN-γ- or TNF-α-producing CD4^+^ T cells, and accumulation of specific DC population in the mucosal region. The same exosomes transmit antigens and induce the production of anti-*Salmonella* antibodies in BALB/c mice administered with these exosomes. This new mechanism of exosome-mediated stimulation of adaptive immunity could find broader importance in other Gram-negative infections and explain better how intracellular infections lead to stimulation of cell-based responses via extracellular vesicles.

## Methods

### Cell and bacterial culture

RAW264.7 murine macrophage cells (ATCC# TIB-71, ATCC, USA) were cultured in DMEM media supplemented with 10% fetal bovine serum (FBS) and 100 μg/ml Penicillin/Streptomycin (Life Technologies Inc., USA). THP-1 cells were cultured, as described previously [57]. The BMDMs have been obtained and cultured as previously described [6]. The overnight cultures of *Salmonella enterica* serovar Typhimurium, strain UK-1 χ3761 (wild-type), and χ9909 (Δ*aroA* vaccine strain) [58] were cultivated at 37°C in lysogeny broth (LB) media, shaking. Overnight bacterial cultures were diluted in new LB media in a 1:80 ratio to reach the optical density at 600 nm (OD_600_) of 0.05 and grown until OD_600_ was 0.50, followed by a wash in PBS before bacterial cultures were used for infections.

### Infection conditions and exosome isolation

Macrophage cells were washed with phosphate-buffered saline (PBS) and incubated in incomplete growth media containing no FBS or antibiotics for 60 minutes before infection with wild-type UK-1 strain of *S*. Typhimurium (χ3761) at a multiplicity of infection (MOI) of 5:1. The overnight culture of χ3761 was diluted as described above and cultivated at 37°C until it reached the mid-logarithmic phase. 2 hours after infection, culture media were replaced with media containing gentamicin (100 μg/mL) for 1 hour, and thereafter replaced with media containing gentamicin (20 μg/mL) and exosome-free heat-inactivated FBS for the remaining time of infection. We chose the time points (0-, 24-, and 48-hours post-infection; hpi) based on our previous study [6]. Exosomes were isolated from the cell culture media by an ultracentrifugation method as described [59]. For more information, see ***S1 Text*.**

LPS-stimulated exosomes were collected by washing RAW264.7 macrophages with PBS and incubating cells for 60 minutes before treatment in media containing no FBS or antibiotics. After 60 minutes of incubation, cells were treated with 1 μg/mL of LPS and incubated in DMEM without FBS or antibiotics for 24 hours. Exosomes were isolated from the cell culture media using the ultracentrifugation method as described [58].

### Transmission electron microscopy; Nanosight tracking analysis; isolation of exosomal proteins; in-gel digestion and mass spectrometry analysis; proteomic data analysis; pathway, function, and network analysis; Western blotting

Please refer to ***S1 Text*** for details.

### In vitro stimulation assays

The PMA stimulated THP-1 or primary BMDM-derived macrophages were treated with exosomes obtained from infected (24 and 48 hpi) or uninfected THP-1 or RAW264.7 macrophages, as indicated in the figures. Several biological replicates, as stated, were pre-treated (or not) for one hour with 20 μg/mL polymyxin B. Alternatively, the cells were pre-treated with the selective 10 μM COX-2 inhibitor NS398 [100 μM], or 10 μM MEK inhibitor (PD184161) for one hour. The cells, as well as cell culture supernatant, were collected for downstream analyses. Concentrations of PGE2 eicosanoid, as well as IL-1β and TNFα, were determined by commercial ELISA kits (Cayman chemical and R&D Systems, respectively).

### qRT-PCR transcript analysis

The transcription of IL-1β, COX-2, IL-12p40, SOCS3, MSR1 andMRC1 was analyzed by qPCR, as we described previously [57]. The transcription of murine iNOS and Arg-1 were analyzed by using the primers described previously [60], while transcripts of TNF, IL-10 were performed utilizing the following primer sequences: TNF-Forward:CCTGTAGCCCACGTCGTAGC; TNF-Reverse:AGCAATGACTCCAAAGTAGACC; IL-10 Forward: TGCACTACCAAAGCCACAAAGCAG; IL-10 Reverse:TCAGTAAGAGCAGGCAGCATAGCA. Briefly, RNA was extracted from cells using a Qiagen RNeasy Mini Plus extraction kit. Next, cDNA was generated using Maxima First Strand cDNA synthesis kit (Thermo Fisher), and expression of genes was measured by using two-step quantitative real-time polymerase chain reaction (RT-qPCR), performed on a Stratagene MXP3005 using SYBRGreen reagents (Bio-Rad). Primer sequences were validated by melt-curve analysis as previously described [55].

### *In vivo* murine experiments

BALB/c 6- to 8-week old female mice (Charles River Laboratories, Inc., Worcester, MA) were used for the *in vivo* experiments. Mice were nasally vaccinated on days 0, 14, and 28 with 20 or 40 μg of exosomes derived from RAW264.7 cells infected with wild-type UK-1 *S*. Typhimurium (χ3761; exosomes were collected at two times points, 24–48 hpi, and pooled, n = 8 for each sample type). In some animals, we also included 2.5 μg of cholera toxin (CT; List Biologicals) or MPLA as an adjuvant (n = 8). Other animals were immunized with the live attenuated vaccine (n = 8) at 5 x 10e9 CFU, or PBS negative control (n = 8). The vaccine (positive control) used in this work was auxotrophic *S*. Typhimurium UK-1 Δ*aroA*21419 (Δ*aroA*) [58] attenuated strain (a generous gift from Dr. Roy Curtiss III, University of Florida). Each mouse was anesthetized and given 30 μl of PBS-suspended exosomes or PBS using a micropipette to administer dropwise into the external nares of mice. For the *S*. Typhimurium UK-1 Δ*aroA*21419 (Δ*aroA*), which was given orally, the actual vaccine viable inoculum CFUs were confirmed by serial dilution tests on agar.

To monitor and track the exosomes obtained from infected RAW264.7 macrophages and administered intranasally (IN) to mice, freshly purified exosomes were labeled with DiR [DiIC18(7) (1,1’-Dioctadecyl-3,3,3’,3’-Tetramethylindotricarbocyanine Iodide)], followed by removal of the free dye by using another PBS wash and finally size-exclusion spin columns. A dose consisting of 20 μg exosomes was administered IN to female BALB/c mice. For the biodistribution of DiR-labeled exosomes 15 minutes, 2, and 24-hours post-administration, mice were anesthetized and imaged under IVIS Spectrum In Vivo System (PerkinElmer, MA, USA). Live Image 4.2 Software was used for analysis. For consistency, the same instrument settings were used to measure any background fluorescence, which was negative (not shown). Furthermore, organs were harvested from the animals in (n = 2 animals) and imaged under the same IVIS System described above. Live (isoflurane-sedated) mice were imaged, or alternatively, the animals were sacrificed, and the organs were harvested before analysis. For the postmortem perfusion experiment, the mice were sedated, and the vascular system was flushed by transcardial perfusion for 5 minutes, and the right atrium was immediately perforated. The left ventricle (LV) was infused with 10 mL of PBS at 1.5 ml/min rate. After, the LV was infused with 10 mL of paraformaldehyde (PFA) 4% in PBS (pH 7.2) at 1.5 mL/min flow rate. The outflow liquid, the liver was monitored during the procedure to assure successful perfusion. After 6.5 minutes of perfusion, the organs were harvested and analyzed. The live mice or the harvested organs were imaged for 2–3 seconds (excitation 745 nm, emission 820 nm). The experimental procedures were compliant with institutional policies for animal health and well-being. The animal protocol was approved by the Institutional Animal Care Use Committee (IACUC), a federally mandated committee that oversees the University of Florida animal program, facilities, and procedures. Animals were maintained at the University of Florida Animal Care Services in individual cages under HEPA-filtered barrier conditions.

### Immunohistochemistry

Please refer to ***S1 Text*** for details.

### Flow cytometry analysis of macrophages and DCs

Exosomes were obtained from *Salmonella*-infected macrophages as described above and stored in -80°C. BALB/c mice were administered PBS or exosomes (n = 4). At 24 hours post-administration, the animals were sacrificed and dissected. Lung was placed on a strainer and minced into smaller portions with scissors, followed by incubation at 37°C (in 10% CO2 incubator) for 1 hour in the digestion buffer consisting of 0.5 mg/mL Collagenase D and 50 U/mL DNAse I. The samples were disturbed every 10 minutes by shaking to dissociate any formed clumps. After incubation, lung tissue was passed through the cell strainer, and cells were collected in media, followed by spinning at 500 x g for 5 mins at 4°C. Next, the red blood cells were lysed in 1mL of TAC red blood cell lysis buffer for 5 mins at room temperature. The samples were further diluted to 15mL with cold PBS and spun down at 500 x g for 5 min at 4°C. The cells were counted; stained with markers for CD45-BV605 (30-F11), CD11b-PB (M1/70), CD11c-PE (N418), CD64-APC-Cy7 (X54-5/7.1), CD24-FITC (M1/69), and MHCII-PE-Cy5 (M5/114.15.2). The gating scheme was used as described previously [61] (**S6 Fig**). Fluorescence reads were acquired on LSR Fortessa flow cytometer (BD Biosciences) with BD FACSDiva software. All samples were analyzed using FlowJo v10 software (Tree Star) and Graph Pad Prism 9. The data were analyzed for outliers by Grubbs’ test, and the *p*-values were determined by an unpaired t-test.

### T cell culture

Cells were harvested from the spleen, head and neck lymph nodes (HNLNs; submandibular, parotid, and superficial cervical lymph nodes) of BALB/c mice and cultured in RMPI media supplemented with 100 μg/mL Penicillin/Streptomycin, 10% fetal bovine serum (Atlanta Biologicals, GA), 10 mM HEPES buffer, 10 mM nonessential amino acids, 10 mM sodium pyruvate (Invitrogen-Life Technologies, Grand Island, NY). Cells were pelleted at 1700 x g for 10 min; resuspended in 10 ml ACK red blood cell lysis buffer (0.15 M NH_4_Cl, 10 mM KHCO_3_, 0.1 mM Na_2_EDTA) for 5 min; pelleted and washed with PBS, and then resuspended in CM and enumerated. Red blood cells were removed from the culture by the addition of ACK (Ammonium-Chloride-Potassium) lysis buffer. Lymphocytes were plated for culture at 10^6^ cells, naïve cells. Cultured lymphocytes were stimulated overnight with 1 x 10^9^ CFUs (colony forming units) of heat-inactivated *S*. Typhimurium, LPS-cured (detoxified), and sonicated lysates of *S*. Typhimurium (χ3761), and subsequently re-stimulated with PMA/ionomycin and blocked with Brefeldin A for intracellular staining analysis. LPS-cured *Salmonella* Ags prepared as described [43], and sonicated lysates (Ag preparations) were prepared from stationary overnight cultures of *S*. Typhimurium x3761 grown in LB at 37°C based on previous protocols [62]. Please refer to ***S1 Text*** for details on antigen preparation.

### Flow cytometry analysis of T cell subsets

To measure intracellular IFN-γ, TNF-α, IL-2, IL-4, and IL-17 expression levels, splenic lymphocytes from BALB/c mice nasally vaccinated with exosomes (exo) derived from infected macrophages, *S*. Typhimurium UK-1 Δ*aroA*21419 attenuated vaccine strain (Δ*aroA*), or PBS were evaluated 14 days after last vaccine dose. For intracellular IFN-γ, TNF-α, IL-2, and IL-17 detection, splenic lymphocytes from individual mice were stimulated in vitro with 10^9^ CFUs of wt *S*. *Typhimurium* [58] for 12 hours followed by treatment with 25 ng/ml PMA and 1 μg/ml ionomycin and simultaneous treatment with 10 μg/ml brefeldin A (BioVision, Milpitas, CA) during the last 6 hours of culture. Cells were then stained with T cell mAbs (PEcy7-anti-CD4, BV711-anti-CD8) but also with PE-Cy5-anti-TCR-β, BV605-anti-CD44, and AF700-anti-CD62L (eBioscience, San Diego, CA), washed, and then were fixed and permeabilized using Fixation/Permeabilization solution (BD Cytofix/Cytoperm) and stained with QD800-labeled anti-IFN-γ, PB-anti-TNF-γ, Cy-anti-IL-2, PE-anti-IL-4 (eBioscience). Fluorescence reads were acquired on LSR Fortessa flow cytometer (BD Biosciences) with BD FACSDiva software. All samples were analyzed using FlowJo software (Tree Star) and Graph Pad Prism 6, where *p*-values were determined by a one-way ANOVA test.

### Antibody response analysis

Mouse antibody titers in sera were determined by sandwich ELISA. Briefly, Nunc MaxiSorp 96 well plates (Thermo Fisher Scientific, Waltham, MA) were coated with 2 μg protein antigen (*S*. Typhimurium x3761 lysate or recombinant OmpA) per well and incubated overnight. Blocking was by 1% bovine serum albumin incubated at 37°C for 2 hours. Wells were washed between steps three times with PBS containing 0.2% Tween-20 then twice with PBS. Serial dilutions of sera were incubated in wells overnight at 4°C. The secondary antibody was goat anti-mouse IgG, human adsorbed-HRP (Southern Biotech, Birmingham, AL), and incubated at 37°C for 90 minutes. 2,2′-Azino-bis(3-ethylbenzthiazoline-6-sulfonic acid (ABTS) substrate (Life Technologies, Frederick, MD) was used for colorimetric determination of titer by reading absorbance at 415 nm in Cytation 3 plate reader (Biotek, USA). Endpoint titers were defined as the reciprocal of the dilution giving absorbance 0.1 U above negative control. The data were analyzed by Graph Pad Prism 6, where *p*-values were determined by the ANOVA test.

### Statistics

The parametric and nonparametric statistical analyses of the data were performed using Prism 8.0 (GraphPad Software Inc., La Jolla, CA, USA). We used the nonparametric Kruskal-Wallis test, followed by the Sidak post-test for all *p*-values (significance *p <* .*05*). All results are expressed as mean+/-SD. The histograms and graphs were produced using Prism software.

## Supporting information

S1 Text Supplementary methods. (DOCX)

S1 Fig Related to Fig 2. **(A)-(C). *Salmonella* proteins enclosed in exosomes derived from macrophages at 24- and 48-hours post-infection in comparison to total *Salmonella* Ags. A.** Graph showing normalized spectral count for *Salmonella* proteins in exosomes derived from infected macrophages (24 hrs and 48 hrs infection time points; S1 and S2 Tables, respectively) in comparison to proteins identified in *Salmonella* Ag preparation. **B.** Graph showing normalized spectral count for *Salmonella* proteins in exosomes derived from infected macrophages (24 hrs and 48 hrs infection time points; S1 and S2 Tables, respectively). **C**. Graph showing normalized spectral count for select *Salmonella* proteins in exosomes derived from infected macrophages (24 hrs and 48 hrs infection time points; S1 and S2 Tables) in comparison to proteins identified in *Salmonella* Ag preparation. **(D)-(E). Quality of proteomic data analysis related to changes in exosomal proteins upon infection.** Spectral quantification of proteins from exosomes derived from infected macrophages in comparison to exosomes from uninfected cells for each time point of infection (24 hrs and 48 hrs; S1 and S2 Tables) identified proteins with a significant abundance in fold change (Fisher’s test *p*<0.05, fold change more significant than -2/+2). A scatterplot representing proteins with various fold change is shown for each time point**. (F)-(G).** Principle Component Analysis (PCA) of Proteomic Analysis of Exosomes derived from macrophages infected with *S*. Typhimurium at 24 hours and 48 hours, each one in comparison to exosomes derived from uninfected control cells.
(TIF)

S2 Fig Related to Fig 2. **Number of predicted MHC II-binding epitopes for identified Ags in exosomes.** The full FASTA sequences of bacterial proteins detected in exosomes by proteomics were analyzed by the MHC class II prediction tool (The Immune Epitope Database, IEDB) for 15-mer peptides. The consensus binding prediction algorithm analyzed the H2-IAd, H2-IEd, H2-IAb alleles to predict epitopes bound by class II MHC. Epitopes with a percentile rank <1% were considered as binders with IC_50_ values < 500.
(TIF)

S3 Fig Related to Fig 2. **(A)-(C). Canonical pathways of identified exosomal proteins affected by S. Typhimurium infection at 48 hpi.** Ingenuity Pathway Analysis (IPA) software was used to analyze the exosomal proteins with differential regulation from *S*. Typhimurium infection (48 hpi). **(A).** Heat map of affected downstream biological processes based on the identified exosomal proteins significantly increased or decreased in infection. **(B).** Canonical pathways represented by exosomal proteins differentially regulated upon infection were analyzed by IPA utilizing Hochberg-Bonferroni multiple testing corrections. **(C).** Clathrin- and caveolae-mediated endocytosis signaling pathways were identified as one of the top altered canonical pathways identified based on the abundance of exosomal proteins significantly altered upon *S*. Typhimurium infection. The included Prediction Legend explains the color scheme.
(TIF)

S4 Fig Related to Fig 2. **Metabolites carried in exosomes from *S*. Typhimurium-infected macrophages. (A)-(B).** Principle Component Analysis (PCA) of metabolomic analysis of exosomes derived from RAW264.7 macrophages infected with *S*. Typhimurium at 24 hours and 48 hours, each one in comparison to exosomes derived from uninfected control cells. PCA graphs show the data from the positive and negative data set, including unknowns. **(C)-(D).** Heatmap of the top 50 metabolites identified by the ANOVA test. **(E).** Statistically significant changes in metabolites identified in exosomes derived from infected (48 hpi) RAW264.7 macrophages in comparison to control macrophages. Human Metabolome CIDs are included for each metabolite, as well as the experimental fold change (Expr Fold Change). All changes were statistically significant per the ANOVA test. **(F).** Ingenuity Pathway Analysis was used to identify the canonical pathway of proteins with altered abundance in exosomes generated upon infected cells (See **Fig 2**), based on which information the down- and up-regulation of specific metabolites were also identified (blue and orange molecules). The experimentally validated metabolites by the metabolomics approach were indicated as graphs (green colors) and indicated with red arrows.
(TIF)

S5 Fig Related to Fig 6. **Effect of exosomes isolated from *Salmonella*-infected macrophages on the secretion of PGE2 and IL-1β or M1/M2 markers. (A-C).** Human THP-1 macrophages were treated with exosomes obtained from *Salmonella*-infected or uninfected THP-1 macrophages in the presence or absence of COX2 or MEK inhibitors that regulate PGE2. Cell culture supernatant was analyzed for secreted PGE2 or IL-1β by using commercial ELISA assays. **(D-F).** BMDMs derived from BALB/c wild-type mice were treated with exosomes isolated from THP-1 macrophages. The polarization markers for M1 and M2 phenotype were analyzed by qPCR. A t-test was used for statistical analysis (n = 3). *P*-values were indicated as follows: * p≤ 0.05; ** p ≤ 0.01; *** p≤ 0.001; **** p ≤ 0.0001.
(TIF)

S6 Fig Related to Fig 5H and 5I. **Gating strategy for myeloid cells in the lung.** Cells were gated on high FSC-A to include myeloid cells. The leukocyte population was captured with the CD45+ gate. Further gating was done for alveolar macrophages, CD103+ dendritic cells, interstitial macrophages, and CD11b+ dendritic cells, as depicted.
(TIF)

S7 Fig Related to Fig 5H and 5I. **Myeloid cell populations in the lungs of BALB/c mice treated with exosomes derived from *Salmonella*- infected macrophages or PBS control.** Exosomes were isolated from *Salmonella*-infected macrophages by ultracentrifugation and stored in -80°C after further use. A dose consisting of 40 μg of exosomes was administered I.N. to female BALB/c mice. At 24-hours post-administration mice were euthanized and lungs collected for cell subpopulation analysis. Percentage and numbers of alveolar macrophages and 1083+ DCs is shown as well as their MHCII expression. Data on graphs are represented as mean ± SD. The *p*-values were determined by t-test, and indicated as follows (n = 4): * p≤ 0.05; ** p ≤ 0.01; *** p≤ 0.001; **** p ≤ 0.0001.
(TIF)

S8 Fig Related to Fig 6. **Analysis of stimulatory effects of exosomes on CD4+ and CD8+ T-lymphocytes in the spleen.** Splenocytes obtained from mice immunized with 40 μg dose exosomes (with and without MPL), PBS, or Δ*aroA S*. Typhimurium were re-stimulated with media (control), whole *Salmonella* antigens, or LPS-cured *Salmonella* antigens. Splenocytes were stained intracellularly for IFN-γ **(A, B)** or IL-17 **(C, D)**. Pools of indicated CD4+ or CD8+ T cells producing IFN-γ or IL-17 were analyzed by FACS. Memory T-lymphocytes were distinguished from naïve T-lymphocytes by gating for effector T-lymphocyte phenotype. Effector T-lymphocytes are characterized phenotypically by high expression levels of CD44+ hi and CD62 lo, in which we subsequently gated for CD4+ specific T-lymphocytes and then CD4+ T-lymphocytes secreting appropriate cytokines as indicated on figures. The results were analyzed by t-test (GraphPad prism), and the stars indicate the *p*-values of 0.05 or smaller in comparison with PBS-immunized mice (n = 3).
(TIF)

S9 Fig Related to Fig 6. **Analysis of stimulatory effects of exosomes on CD4+ T-lymphocytes in the spleen.** Splenocytes obtained from mice immunized with 40 μg dose exosomes, PBS, or Δ*aroA S*. Typhimurium were re-stimulated with media (control), or *Salmonella* antigens. Splenocytes were stained intracellularly for IFN-γ. Pools of indicated CD4+ T cells producing IFN-γ were analyzed by FACS like in S8 Fig. The results were analyzed by two-way ANOVA (GraphPad Prism 9) for statistical significance, where the p-values are indicated as follows: * p≤ 0.05; ** p ≤ 0.01; *** p≤ 0.001; **** p ≤ 0.0001.
(TIF)

S10 Fig Related to Fig 6. **Examples of individual plots of FACS analysis of the TNF-α- and IFN-γ-producing CD4+ T lymphocytes from the spleen of mice immunized with exosomes derived from infected macrophages. (A)-(C).** FACS analysis of T lymphocytes from the spleen of mice immunized with exosomes at the doses indicated on figures (20 μg, 40 μg, 20 μg with CT adjuvant, 40 μg with CT adjuvant, or PBS control) as well as mice immunized with live attenuated *Salmonella* vaccine (Δ*aroA S*. Typhimurium). Exosomes were derived from RAW264.7 macrophages infected with *S*. Typhimurium (pooled 24–48 hpi). Lymphocytes from the spleen were isolated and re-stimulated with Heat-killed *S*. Typhimurium (St Ags (HK St), LPS-detoxified *S*. Typhimurium antigens (LPS-cured St Ags), or a media control, and CD4+ T lymphocytes were analyzed for the secretion of TNF-α- and IFN-γ. The gating for analysis was done as in **Fig 6**.
(TIF)

S11 Fig Related to Fig 6. **Examples of individual plots of FACS analysis of IL-2-producing CD4+ T lymphocytes from the spleen of mice immunized with exosomes derived from infected macrophages. (A)-(F).** FACS analysis of T lymphocytes from the spleen of mice immunized with exosomes at the doses indicated on figures (20 μg, 40 μg, 20 μg with CT adjuvant, 40 μg with CT adjuvant, or PBS control) as well as mice immunized with live attenuated *Salmonella* vaccine (Δ*aroA S*. Typhimurium). Exosomes were derived from RAW264.7 macrophages infected with *S*. Typhimurium (pooled 24–48 hpi). Lymphocytes from the spleen were isolated and re-stimulated with Heat-killed *S*. Typhimurium (St Ags (HK St), LPS-detoxified *S*. Typhimurium antigens (LPS-cured St Ags), or a media control, and CD4+ T lymphocytes were analyzed for the secretion of IL-2. The gating for analysis was performed as shown in **Fig 6**.
(TIF)

S12 Fig Related to Fig 7. **Exosomes derived from *S*. Typhimurium-infected macrophages induce the production of *Salmonella*-specific antibodies**. Production of antibodies by mice immunized with exosomes with or without adjuvant (CT), PBS, or aroA-deficient S. Typhimurium. The analyzed antibodies were against **(A)** LPS-cured *S*. Typhimurium and (**B-C)** Outer Membrane Protein A (OmpA), all analyzed for bleed 3 and 4 (**Fig 5**).
(TIF)

S1 Table Spectral quantification of host proteins from exosomes from infected macrophages (24 hpi) in comparison to exosomes from uninfected macrophages. Calculated fold change was based on the normalized spectral values, and p-values were calculated by using Fisher’s test. ID, Symbol, Entrez Gene Name as well as predicted protein type and targeting drugs have been shown (Ingenuity Pathway Analysis, Qiagen).
(PDF)

S2 Table Spectral quantification of host proteins from exosomes from infected macrophages (48 hpi) in comparison to exosomes from uninfected macrophages. Calculated fold change was based on the normalized spectral values, and p-values were calculated by using Fisher’s test. ID, Symbol, Entrez Gene Name as well as predicted protein type and targeting drugs have been shown (Ingenuity Pathway Analysis, IPA, Qiagen).
(PDF)

S3 Table Spectral quantification of host proteins from exosomes from macrophages infected for 24 hpi in comparison to 48 hpi. Calculated fold change was based on the normalized spectral values (48 hpi in comparison to 24 hpi as control), and p-values were calculated by using Fisher’s test. ID, Symbol, and Entrez Gene Name have been shown (Ingenuity Pathway Analysis, IPA, Qiagen). Only significant values are shown for differentially regulated proteins.
(PDF)

S4 Table Biological functions of exosomal proteins affected by infection 48 hpi. The exosomal proteins identified by proteomics with a protein level differentially regulated by infection (48 hpi) were analyzed by Ingenuity Pathway Analysis software to identify the downstream processes that these proteins regulate. P-value and prediction of the activated state, as well as activation z-score of each function, are shown. Molecules involved in each pathway related to a particular process, as well as the number of proteins identified in our differentially expressed datasets, are also shown.
(PDF)

S5 Table Exosomal proteins affected by infection 48 hpi related to endocytosis. The exosomal proteins identified by proteomics with a protein level differentially regulated by infection (48 hpi) were analyzed by Ingenuity Pathway Analysis software to identify the canonical functions that these proteins regulate. Identified proteins related to endocytosis are shown in the table. Symbol, Entrez Gene Name, Protein Accession number, Experimental p-value, Fold Change, Protein Type(s), Mapped Entrez Gene ID for Human and Mouse are shown for each protein.
(PDF)
