# Supplementary material for: Antigen-encapsulating host extracellular vesicles derived from Salmonella-infected cells stimulate pathogen-specific Th1-type responses in vivo
Source: PLoS Pathog. 2021 May 6;17(5):e1009465. doi: 10.1371/journal.ppat.1009465 (PMC8101724; doi:10.1371/journal.ppat.1009465)
Supplement: S1 Text — (DOCX) [file ppat.1009465.s001.docx]

**Supporting information**

**Methods**

*Exosome isolation*

After infection for the indicated times, cell culture supernatant containing secreted EVs was collected, resuspended in PBS containing protease inhibitor cocktail (EDTA-free; Roche, USA) and filtered through a 0.22-micron polyethersulfone (PES) filter. The samples were centrifuged sequentially at the following conditions: 10 min at 500 × g, 10 min at 2,000 × g, and 40 min at 16,000 × g to remove cellular debris and bacteria. The supernatant was collected into a new vial and spun for 180 min at 100,000 × g by using SW 32 Ti rotor and Optima XPN ultracentrifuge (Beckman, USA). The supernatant was removed, and the pellets containing exosomes were washed by PBS, and additional centrifugation was carried out at 100,000 × g. The supernatant was removed from the exosome pellet, resuspended in sterile PBS containing protease inhibitor cocktail (Roche, USA). Exosomes were analyzed by using Nanoparticle Tracking Analysis (NTA, NanoSight LM10) to establish concentration and the hydrodynamic diameter of exosomes. Samples containing exosomes were diluted in PBS to reach a concentration of 1.0 × 10^8^ to 9.0 × 10^8^ particles/ml and measured as we did previously [1].

Transmission Electron Microscopy

For electron microscopy analysis of THP-1 and RAW 264.7 derived exosomes, the vesicle pellet was resuspended in 4% paraformaldehyde in PBS, 15 uL sample was placed on formvar and carbon-coated grid. After 10 min of coating, a piece of filter paper was used to eliminate excess fluid from the grid, and a drop of 2% aqueous uranyl acetate was applied to the grid. After 20 seconds, the excess stain was removed with filter paper, and the grid was dried for 1 hour before viewing by the JEOL JEM1230 Transmission Electron Microscope at 80kv.

Nanosight Tracking Analysis

The concentration and size/intensity of exosomes were analyzed by NanoSight LM10. The PBS used to dilute the exosome samples was also analyzed in the NanoSight to ensure that the solution is not contaminated. Extracellular vesicles were diluted in PBS to reach a concentration of 1.0-9.0 × 10^8^ particles/mL. Once the desired concentration was reached, the sample was injected into the sample chamber of the NanoSight, and particle size distribution was obtained by using Nanoparticle Tracking Analysis (NTA), where the mean square displacement of scattering species that cross the path of a sheet laser was measured. Next, the hydrodynamic diameter of objects is calculated by using the Stokes-Einstein equation. Measurement of a large number of scatters yields direct measurements of the hydrodynamic distribution and concentration of particulates in the sample. Data were analyzed and graphed in IGOR Pro 7 (WaveMetrics Inc.). We verified that the fractions do not contain bacteria by culturing the samples in cell culture media.

**Isolation of exosomal proteins, in-gel digestion, and mass spectrometry analysis**

Exosomes were isolated from uninfected macrophages, and *S.* Typhimurium-infected RAW 264.7 macrophages [multiplicity of infection (MOI) was 5:1, and the samples were collected 0, 24, and 48 hours post-infection (hpi)]. Exosomes (triplicate samples) were lysed by NP-40 lysis buffer (0.5% NP-40, 0.15 M NaCl, 0.02 M CaCl_2_ * H2O, 0.05 M Tris, pH7.4), and subsequently boiled at 98ºC for 5 minutes. Protein concentration was determined by bicinchoninic acid (BCA) assay. An equal amount of protein 25 µg per sample was used, and three replicates of exosomes per sample type were separated by sodium dodecyl sulfate-polyacrylamide gel electrophoresis (SDS-PAGE). The entire lane per sample was excised by scalpel and diced into one mm^2^-cubes, followed by in-gel trypsin digestion performed exactly as we did previously [2]. The peptide samples were then analyzed by 250-mm Ultrahigh-Performance Liquid Chromatography (UHPLC) coupled to Orbitrap Fusion mass spectrometer (Thermo Scientific). The liquid chromatography was performed using the Thermo EASY nano-LC system, where 20-mm C16 pre-column (Thermo Scientific) was used to rid of impurities, after which the samples were separated by using a reversed-phase C18 analytical column with the 100Å pore (Thermo Scientific, Acclaim PepMap 100 C18 LC Column). For the chromatography, the following solvents were used: solvent A (0.1% formic acid), 2–40% solvent B (80% acetonitrile, 0.1% formic acid), and 105-minute acetonitrile gradient was used was the separation phase, followed by 14 minutes wash with 98% solvent B, and finally column equilibration with 2% solvent A. The LC system was interfaced directly with Orbitrap Fusion mass spectrometer (Thermo Scientific). MS data were acquired at 120K resolution by Orbitrap detector at a scan range of 350-2000 m/z. For MS/MS analysis, ions were isolated by a quadrupole, prioritizing the most intense ions and injecting ions for all available parallelizable time. Once ions were selected for fragmentation, the masses of these precursor ions were then excluded for 36 s. Fragmentation was done by using collision-induced dissociation (CID), at a collision energy of 35% and activation time of 10 ms, where the AGC target of 10000. The MS/MS data were detected by the ion trap.

**Proteomic data analysis**

Tandem mass spectra were extracted, charge state deconvoluted, and deisotoped by Proteome Discoverer (Thermo Scientific) version 2.1. All MS/MS samples were analyzed using Sequest (Thermo Fisher Scientific, version 2.1.1.21) and X! Tandem (version CYCLONE 2010.12.01.1). Sequest was set up to search FASTA mouse, and *Salmonella* Typhimurium Uniprot database containing a common list of contaminants database (56,604 entries) assuming the digestion enzyme trypsin. Sequest and X! Tandem were both searched with a fragment ion mass tolerance of 1.00 Da and a parent ion tolerance of 10.0 ppm (parts-per-million). Carbamidomethyl of cysteine was specified in Sequest and X! Tandem as a fixed modification. Deamidation of asparagine, oxidation of methionine and acetyl of the n-terminus were specified in Sequest as variable modifications. Scaffold (version 8.1, Proteome Software Inc.) was used to validate MS/MS-based peptide and protein identifications. Peptide identifications were accepted if they were established at greater than 95.0% probability by the Scaffold Local FDR (false discovery rate) algorithm. Protein identifications were accepted if they could be established at greater than 95.0% probability and contained at least two identified peptides. Protein probabilities were assigned by the Protein Prophet algorithm. Proteins that contained similar peptides and could not be differentiated based on MS/MS analysis alone were grouped to protein groups, and only the top hit was reported. The reported peptide false discovery rate (FDR) was 0.35%, and the protein FDR was 3.3%.

The Venn diagrams, including protein numbers in each sample category, were constructed in Venny 2.0 (BioinfoGP, CSIC) and the graphs included the protein numbers for hits that appeared in two out of three replicates in each sample type. Gene ontology (GO) terms in proteins identified in at least two replicates were analyzed by using PANTHER Overrepresentation Test (Released 2018-11-13), where GO Ontology database (Released 2019-01-01) related to GO cellular component was used. Fisher’s Exact test with Bonferroni correction for multiple testing was used to calculate the *p*-value, which was then converted to negative Log_10_ value.

The weighted spectral count was used for protein quantification. The spectral counts of peptides were normalized based on the total count in each run. The fold change was calculated from the spectral count of proteins from exosomes derived from infected cells in comparison to exosomes from uninfected cells. A Fisher exact test was used to calculate statistical significance and a P-value of <0.05 indicated proteins with statistically significant changes in abundance. To visualize the protein abundance by using heat maps, we used Morpheus (<https://software.broadinstitute.org/morpheus/>). The Principal Component Analysis (PCA) was done by ClustVis [3].

**Analysis of predicted epitopes by MHC class II prediction tool (The Immune Epitope Database, IEDB).** The full FASTA sequences of *Salmonella* proteins detected in exosomes by proteomics were analyzed by MHC class II prediction tool (IEDB) for 15-mer peptides The following alleles were analyzed: H2-IAd, H2-IEd, H2-IAb. The consensus binding prediction algorithm was used to predict epitopes bound by class II MHC. Epitopes with a percentile rank <1% were considered as binders with IC50 value< 500.

**Pathway, function and network analysis**

Ingenuity Pathway Analysis software (Qiagen) was used for network analysis of exosomal proteins with different abundance upon exosomes isolated from *S.* Typhimurium-infected RAW 264.7 macrophages at 24 and 48 hpi, as compared with exosomes isolated from uninfected cells. Canonical pathways and bio-functions of proteins were analyzed by the right-tailed Fisher’s exact test and Hochberg-Bonferroni multiple testing correction to show the most significant results. The calculated significance represents the probability of association of proteins with the canonical pathway by random chance alone, and the –log of this p-value is shown on the x-axis. For canonical pathways, the number of genes/proteins in a pathway from the dataset was displayed, both proteins which were upregulated (red) as well as downregulated (green) in exosomes released from cells after infection. The total number of known genes was displayed in the right part of the graph. For protein bio functions, the overall heat map of up- and down-regulated functions is shown. Moreover, activation of specific functions was identified and measured by Z-score higher than 2/-2, and relevant canonical pathways (CPs) were visualized as well. The top protein network was identified and overlaid with the most significant CPs. The regulator effect analysis was performed, and a network with a consistency score of 21.822 containing four diseases/functions was shown. Amongst the three upstream regulators predicted to be upregulated by the IPA software, STAT1 was identified in our dataset as upregulated (fold change of 3.6), therefore verifying this result. Upstream network analysis was performed, and the regulators predicted to be activated or inhibited were identified based on z-scores higher than +2/-2.

**Metabolomics**

Exosome from RAW 264.7 macrophages infected or not infected with S. Typhimurium (24 hpi and 48 hpi) were obtained as for the proteomics analysis. All samples were extracted following the cellular extraction procedure without pre-normalization of the sample protein content. Global metabolomics profiling was performed on a Thermo Q-Exactive Orbitrap mass spectrometer in-line with Dionex UHPLC, which was equipped with an autosampler. All samples were analyzed in positive and negative heated electrospray ionization by using a mass resolution of 35,000 at m/z 200. Separate injections were used for negative and positive electrospray ionization. Separation of ions was achieved on an ACE 18-pfp 100 x 2.1 mm, 2 µm column with mobile phase A as 0.1% formic acid in water and mobile phase B was acetonitrile. The flow rate for the UHPLC was set at 350 µL/min, while the column temperature was 25°C. 4 µL was injected for negative ions and 2 µL for positive ions. Data from positive and negative ion modes were separately subjected to statistical analyses. There were a total of 2391 features detected from the positive mode and 1227 features in the negative mode, as shown in Figure 1. All subsequent data analyses were normalized to the sum of metabolites for each sample. MZmine was used to identify features, deisotope, align features, and perform a gap-filling to fill in any features that may have been missed in the first alignment algorithm. All adducts and complexes were identified and removed from the dataset. The data were searched against the internal retention time metabolite library. Subsequent searches against HMDB were performed, and the data was then analyzed by Ingenuity Pathway Analysis software along with the proteomics data to integrate the omics results.

**Western blotting**

Exosomes or cells were lysed, as we described previously [1, 4]. For cell lysis, 0.1% NP-40 lysis buffer (0.1% NP-40, 150 mM NaCl, 20 mM CaCl2, 50 mM Tris pH 7.4) was used containing protease inhibitor cocktail (Roche Applied Science). Protein concentration was established by using a BCA assay, and equal amount of protein was taken from each sample to which a reducing sodium dodecyl sulfate buffer was added. The samples were boiled for 3 minutes at 95°C and resolved by using 4-12% gradient sodium dodecyl sulfate-polyacrylamide gel electrophoresis (SDS-PAGE). The proteins were then transferred onto polyvinylidene fluoride (PVDF; Bio-Rad) membrane by using Tran-Bot Turbo Transfer System (Bio-Rad). Membranes were blocked in PBS with 5% milk and 0.05% Tween-20 for 1 hour, washed in PBS containing 0.05% Tween-20, then probed with an appropriate primary antibody diluted in PBS containing 1% milk and 0.05% Tween-20, at 4°C for ~15 hours. The antibodies used were VPS37B, NOTCH2, JAK1 (Santa Cruz, USA) and CD9 and CD63 (Systems Biosciences, USA). The membrane was then washed four times with 0.05% Tween-20 solution in PBS, after which horseradish peroxidase (HRP)-conjugated secondary antibody in 1% milk, 0.05% Tween-20 in PBS was added to the membranes. The SuperSignal Chemiluminescent Substrate (Thermo Fisher Scientific) was used for visualization, and the blots were exposed to a film or visualized by using the iBright system (Thermo Fisher, USA). ImageJ was used for the measurements of band density, and graphs of the abundance of proteins were prepared in GraphPad Prism 7.

**Recombinant OmpA protein generation**

OmpA-containing pET SUMO plasmid was obtained from Dr. Ramnath Misra (Department of Clinical Immunology, Sanjay Gandhi Post Graduate Institute of Medical Sciences, Lucknow, India) and it was described elsewhere [5]. Briefly, the protein was expressed by growing *E. coli* BL21 (lambdaDE3) containing the plasmid at 37°C to mid-log phase (OD_600_= 0.6), after which protein expression was induced with IPTG and temperature reduced to 21°C. Cells were collected by centrifugation and lysed by a French press. Purification was done by column chromatography, first with Ni-NTA affinity chromatography (HiTrap column, GE Healthcare Life Sciences), followed by removal of His-tag with SUMO protease, and gel filtration/size exclusion chromatography (Sephacryl S-200 HR column, GE Healthcare Life Sciences).

**Immunohistochemistry**

*Specimen preparation.* Following euthanasia using cervical dislocation, the organs were explanted, embedded in optimal cutting temperature (OCT) compounds Tissue-Plus (Scigen, Scientific Gardena, Gardena, CA) and snap-frozen immediately. The frozen blocks were then sectioned to 12 m thickness and mounted on Fisherbrand™ Premium Frosted Microscope Slides (Gerhard Menzel, Braunschweig, Germany).

# *Sectioning, staining, and imaging.* The sections were fixed for 10 minutes in 4% formaldehyde, washed three times for 5 minutes each in PBS, permeabilized with 0.5% Triton X100 (Sigma- Aldrich, US) and then blocked using PBS with 5% goat serum (Life Technologies, CA) and for one hour. The allophycocyanin (APC)-conjugated rat monoclonal anti-mouse F4/80 antibody (BM8 clone) (eBiosciences, San Diego, CA) and FITC-conjugated hamster anti-mouse CD11c (HL3 clone) (eBiosciences, San Diego, CA) were diluted at 1:500 in an antibody dilution buffer consisting of PBS with 1% goat serum being added to each sample and incubated overnight at 4^o^C. The sections were again washed, mounted with coverslips using Prolong Gold Antifade with 4,6-diamidino-2- phenylindole (DAPI) (Life Technologies) and imaged using an inverted fluorescent microscope (Cytation 5 Cell Image Multimode Reader, Biotek, Winooski, VA, US).

***Antigen preparation***

For heat-inactivated *Salmonella*, the Ags were prepared as follows: *Salmonella* culture was grown overnight at 37ºC a 200 rpm with shaking. OD600 nm was read to determine CFU/ml. Cells were pelleted at 13,000 x g and washed twice with PBS, where the cells were pelleted again by centrifugation between each wash. Cells were resuspended in PBS to a concentration of 2e+10 CFU/ml. This cell suspension was aliquoted in volumes of 650-700 µl in 1.5 ml microfuge tubes and heated at 70ºC for 45 minutes for heat inactivation of the pathogen, which were the conditions for which no growth of bacteria was visible on LB agar plates.

For the sonication of bacteria and LPS-inactivation, the Ags were prepared as follows: stationary *Salmonella* culture was spun at 13,000 x g at 4ºC, and the obtained pellet was washed with PBS containing 5 mM EDTA, spun down again, and then washed with PBS alone. The cells were sonicated by using the Sonifier Cell Disruptor (Heat Systems-Ultrasonics Inc., Plainview, NY), followed by pelleting the debris 13,000 x g at 4ºC to obtain protein-containing supernatant. Filter sterilization of such obtained Ag preparation was carried out by filtering through a 0.22-µm PES (polyethersulfone) filter (Genesee Scientific, El Cajon, CA). For the NaOH inactivation of LPS, 10M NaOH was added to the Ag preparation to a final concentration of 0.25M NaOH, and the tube was incubated at 37ºC for 3 hours, where the control sonicated Ags (not NaOH-treated) were incubated alongside. Afterward, phenol red was added to monitor the alkaline pH. Hydrochloric acid was then titrated to adjust pH to neutral, which was then verified by the pH paper strip. The Ag preparations were incubated on LB agar plates to verify that no live cells remain.

***Bibliography***

1. Hui WW, Hercik K, Belsare S, Alugubelly N, Clapp B, Rinaldi C, et al. Salmonella enterica Serovar Typhimurium Alters the Extracellular Proteome of Macrophages and Leads to the Production of Proinflammatory Exosomes. Infect Immun. 2018;86(2). Epub 2017/11/22. doi: 10.1128/iai.00386-17. PubMed PMID: 29158431; PubMed Central PMCID: PMCPMC5778363.

2. Lee JH, Hou X, Kummari E, Borazjani A, Edelmann MJ, Ross MK. Endocannabinoid hydrolases in avian HD11 macrophages identified by chemoproteomics: inactivation by small-molecule inhibitors and pathogen-induced downregulation of their activity. Mol Cell Biochem. 2018;444(1-2):125-41. Epub 2017/12/03. doi: 10.1007/s11010-017-3237-0. PubMed PMID: 29196970.

3. Metsalu T, Vilo J. ClustVis: a web tool for visualizing clustering of multivariate data using Principal Component Analysis and heatmap. Nucleic Acids Res. 2015;43(W1):W566-70. Epub 2015/05/12. doi: 10.1093/nar/gkv468. PubMed PMID: 25969447; PubMed Central PMCID: PMCPMC4489295.

4. Edelmann MJ, Shack LA, Naske CD, Walters KB, Nanduri B. SILAC-based quantitative proteomic analysis of human lung cell response to copper oxide nanoparticles. PLoS One. 2014;9(12):e114390. Epub 2014/12/04. doi: 10.1371/journal.pone.0114390. PubMed PMID: 25470785; PubMed Central PMCID: PMCPMC4255034.

5. Chaurasia S, Shasany AK, Aggarwal A, Misra R. Recombinant Salmonella typhimurium outer membrane protein A is recognized by synovial fluid CD8 cells and stimulates synovial fluid mononuclear cells to produce interleukin (IL)-17/IL-23 in patients with reactive arthritis and undifferentiated spondyloarthropathy. Clin Exp Immunol. 2016;185(2):210-8. Epub 2016/05/23. doi: 10.1111/cei.12799. PubMed PMID: 27060348; PubMed Central PMCID: PMCPMC4955008.
