# Supplementary figures and images for: Antigen-encapsulating host extracellular vesicles derived from Salmonella-infected cells stimulate pathogen-specific Th1-type responses in vivo
Source: PLoS Pathog. 2021 May 6;17(5):e1009465. doi: 10.1371/journal.ppat.1009465 (PMC8101724; doi:10.1371/journal.ppat.1009465)

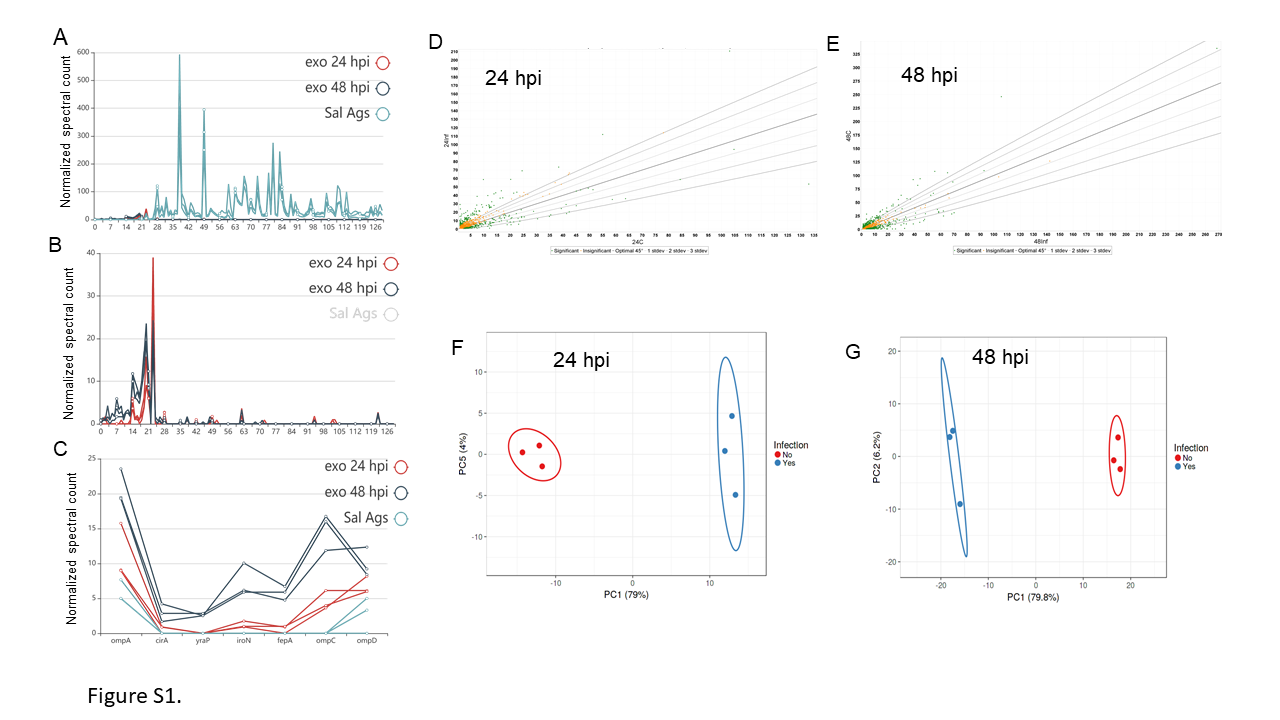

Supplement: S1 Fig — (A)-(C). Salmonella proteins enclosed in exosomes derived from macrophages at 24- and 48-hours post-infection in comparison to total Salmonella Ags. A. Graph showing normalized spectral count for Salmonella proteins in exosomes derived from infected macrophages (24 hrs and 48 hrs infection time points; S1 and S2 Tables, respectively) in comparison to proteins identified in Salmonella Ag preparation. B. Graph showing normalized spectral count for Salmonella proteins in exosomes derived from infected macrophages (24 hrs and 48 hrs infection time points; S1 and S2 Tables, respectively). C. Graph showing normalized spectral count for select Salmonella proteins in exosomes derived from infected macrophages (24 hrs and 48 hrs infection time points; S1 and S2 Tables) in comparison to proteins identified in Salmonella Ag preparation. (D)-(E). Quality of proteomic data analysis related to changes in exosomal proteins upon infection. Spectral quantification of proteins from exosomes derived from infected macrophages in comparison to exosomes from uninfected cells for each time point of infection (24 hrs and 48 hrs; S1 and S2 Tables) identified proteins with a significant abundance in fold change (Fisher’s test p<0.05, fold change more significant than -2/+2). A scatterplot representing proteins with various fold change is shown for each time point. (F)-(G). Principle Component Analysis (PCA) of Proteomic Analysis of Exosomes derived from macrophages infected with S. Typhimurium at 24 hours and 48 hours, each one in comparison to exosomes derived from uninfected control cells. (TIF) [file ppat.1009465.s002.TIF]

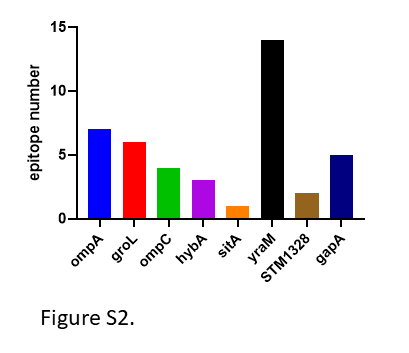

Supplement: S2 Fig — Number of predicted MHC II-binding epitopes for identified Ags in exosomes. The full FASTA sequences of bacterial proteins detected in exosomes by proteomics were analyzed by the MHC class II prediction tool (The Immune Epitope Database, IEDB) for 15-mer peptides. The consensus binding prediction algorithm analyzed the H2-IAd, H2-IEd, H2-IAb alleles to predict epitopes bound by class II MHC. Epitopes with a percentile rank <1% were considered as binders with IC50 values < 500. (TIF) [file ppat.1009465.s003.TIF]

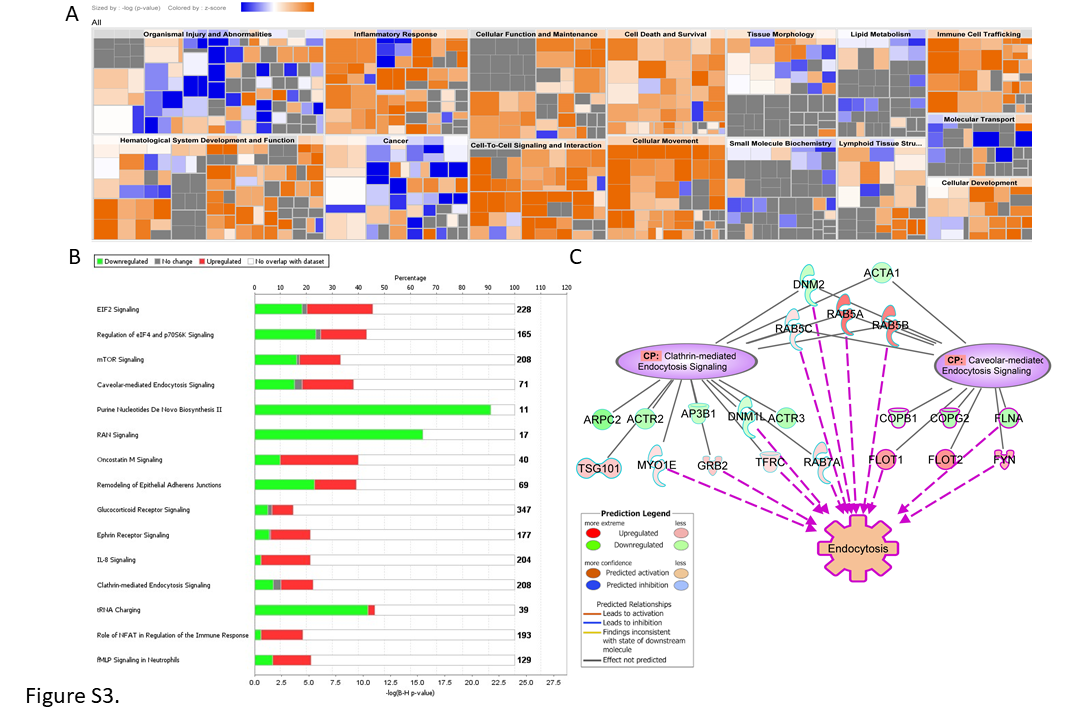

Supplement: S3 Fig — (A)-(C). Canonical pathways of identified exosomal proteins affected by S. Typhimurium infection at 48 hpi. Ingenuity Pathway Analysis (IPA) software was used to analyze the exosomal proteins with differential regulation from S. Typhimurium infection (48 hpi). (A). Heat map of affected downstream biological processes based on the identified exosomal proteins significantly increased or decreased in infection. (B). Canonical pathways represented by exosomal proteins differentially regulated upon infection were analyzed by IPA utilizing Hochberg-Bonferroni multiple testing corrections. (C). Clathrin- and caveolae-mediated endocytosis signaling pathways were identified as one of the top altered canonical pathways identified based on the abundance of exosomal proteins significantly altered upon S. Typhimurium infection. The included Prediction Legend explains the color scheme. (TIF) [file ppat.1009465.s004.TIF]

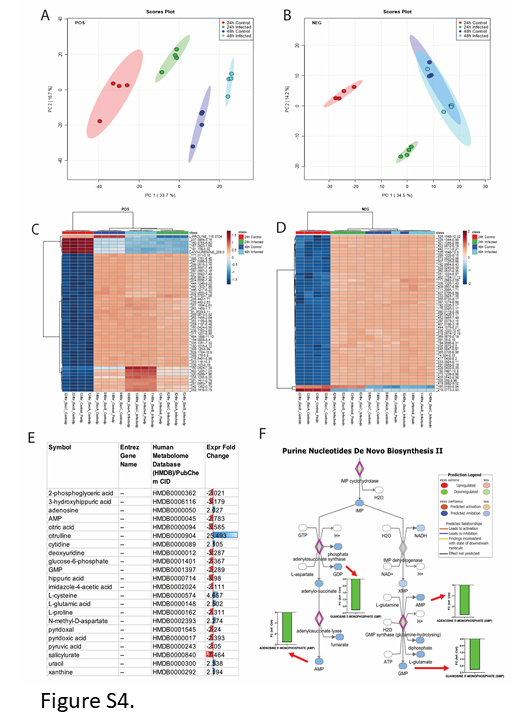

Supplement: S4 Fig — Metabolites carried in exosomes from S. Typhimurium-infected macrophages. (A)-(B). Principle Component Analysis (PCA) of metabolomic analysis of exosomes derived from RAW264.7 macrophages infected with S. Typhimurium at 24 hours and 48 hours, each one in comparison to exosomes derived from uninfected control cells. PCA graphs show the data from the positive and negative data set, including unknowns. (C)-(D). Heatmap of the top 50 metabolites identified by the ANOVA test. (E). Statistically significant changes in metabolites identified in exosomes derived from infected (48 hpi) RAW264.7 macrophages in comparison to control macrophages. Human Metabolome CIDs are included for each metabolite, as well as the experimental fold change (Expr Fold Change). All changes were statistically significant per the ANOVA test. (F). Ingenuity Pathway Analysis was used to identify the canonical pathway of proteins with altered abundance in exosomes generated upon infected cells (See Fig 2), based on which information the down- and up-regulation of specific metabolites were also identified (blue and orange molecules). The experimentally validated metabolites by the metabolomics approach were indicated as graphs (green colors) and indicated with red arrows. (TIF) [file ppat.1009465.s005.TIF]

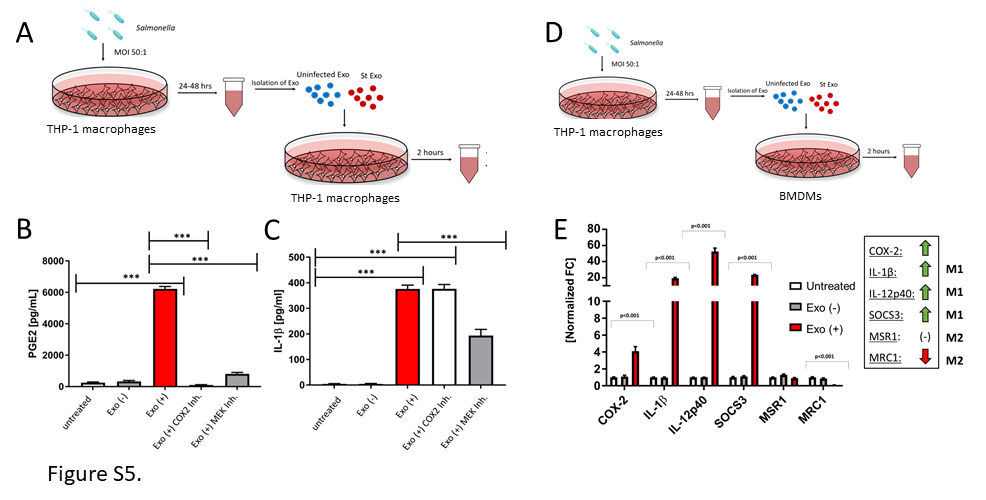

Supplement: S5 Fig — Effect of exosomes isolated from Salmonella-infected macrophages on the secretion of PGE2 and IL-1β or M1/M2 markers. (A-C). Human THP-1 macrophages were treated with exosomes obtained from Salmonella-infected or uninfected THP-1 macrophages in the presence or absence of COX2 or MEK inhibitors that regulate PGE2. Cell culture supernatant was analyzed for secreted PGE2 or IL-1β by using commercial ELISA assays. (D-F). BMDMs derived from BALB/c wild-type mice were treated with exosomes isolated from THP-1 macrophages. The polarization markers for M1 and M2 phenotype were analyzed by qPCR. A t-test was used for statistical analysis (n = 3). P-values were indicated as follows: * p≤ 0.05; ** p ≤ 0.01; *** p≤ 0.001; **** p ≤ 0.0001. (TIF) [file ppat.1009465.s006.TIF]

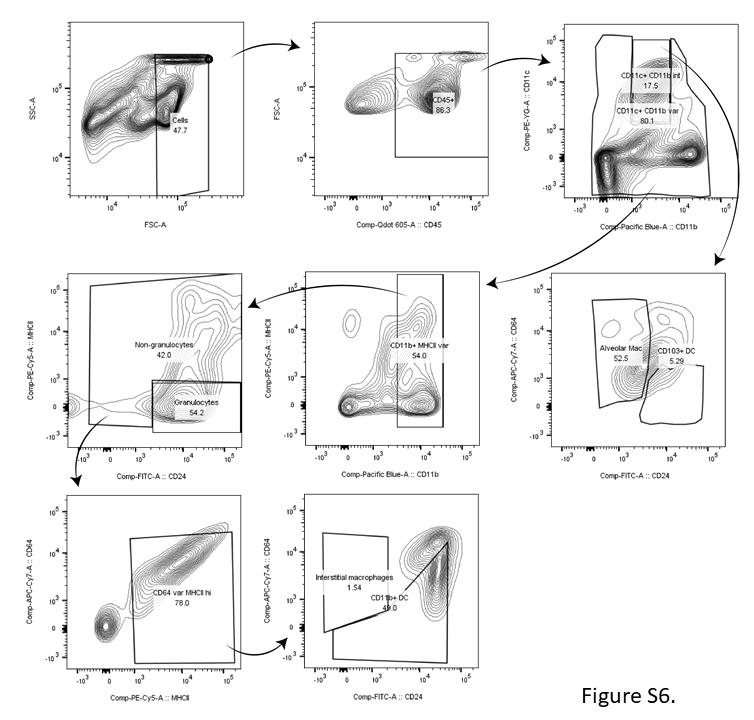

Supplement: S6 Fig — Gating strategy for myeloid cells in the lung. Cells were gated on high FSC-A to include myeloid cells. The leukocyte population was captured with the CD45+ gate. Further gating was done for alveolar macrophages, CD103+ dendritic cells, interstitial macrophages, and CD11b+ dendritic cells, as depicted. (TIF) [file ppat.1009465.s007.TIF]

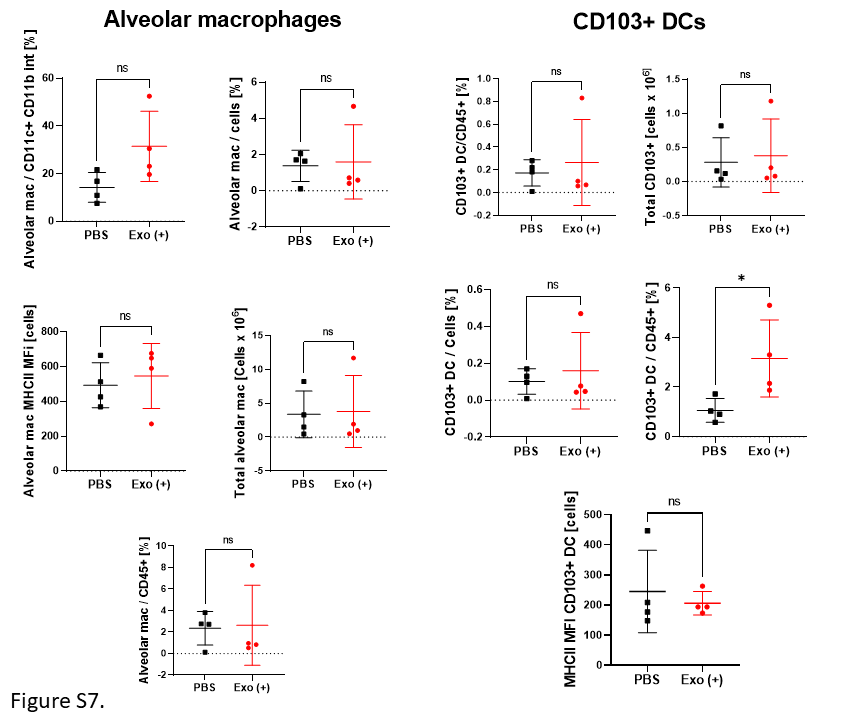

Supplement: S7 Fig — Myeloid cell populations in the lungs of BALB/c mice treated with exosomes derived from Salmonella- infected macrophages or PBS control. Exosomes were isolated from Salmonella-infected macrophages by ultracentrifugation and stored in -80°C after further use. A dose consisting of 40 μg of exosomes was administered I.N. to female BALB/c mice. At 24-hours post-administration mice were euthanized and lungs collected for cell subpopulation analysis. Percentage and numbers of alveolar macrophages and 1083+ DCs is shown as well as their MHCII expression. Data on graphs are represented as mean ± SD. The p-values were determined by t-test, and indicated as follows (n = 4): * p≤ 0.05; ** p ≤ 0.01; *** p≤ 0.001; **** p ≤ 0.0001. (TIF) [file ppat.1009465.s008.TIF]

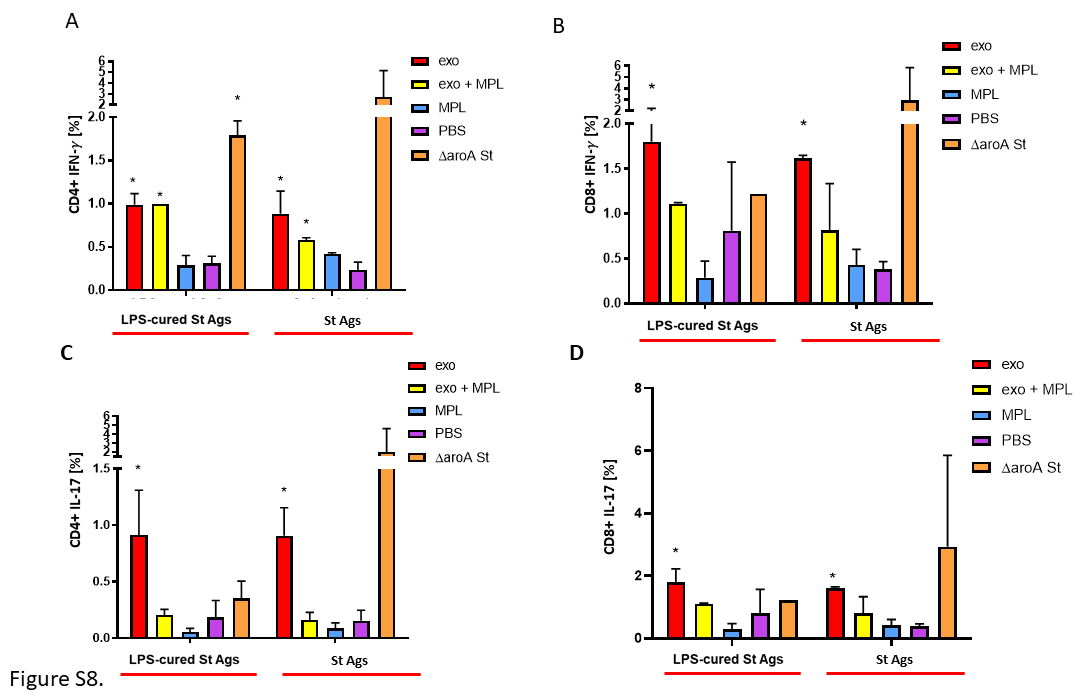

Supplement: S8 Fig — Analysis of stimulatory effects of exosomes on CD4+ and CD8+ T-lymphocytes in the spleen. Splenocytes obtained from mice immunized with 40 μg dose exosomes (with and without MPL), PBS, or ΔaroA S. Typhimurium were re-stimulated with media (control), whole Salmonella antigens, or LPS-cured Salmonella antigens. Splenocytes were stained intracellularly for IFN-γ (A, B) or IL-17 (C, D). Pools of indicated CD4+ or CD8+ T cells producing IFN-γ or IL-17 were analyzed by FACS. Memory T-lymphocytes were distinguished from naïve T-lymphocytes by gating for effector T-lymphocyte phenotype. Effector T-lymphocytes are characterized phenotypically by high expression levels of CD44+ hi and CD62 lo, in which we subsequently gated for CD4+ specific T-lymphocytes and then CD4+ T-lymphocytes secreting appropriate cytokines as indicated on figures. The results were analyzed by t-test (GraphPad prism), and the stars indicate the p-values of 0.05 or smaller in comparison with PBS-immunized mice (n = 3). (TIF) [file ppat.1009465.s009.TIF]

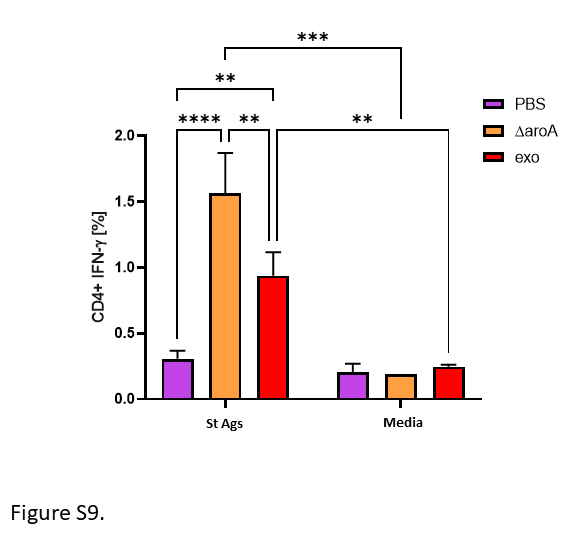

Supplement: S9 Fig — Analysis of stimulatory effects of exosomes on CD4+ T-lymphocytes in the spleen. Splenocytes obtained from mice immunized with 40 μg dose exosomes, PBS, or ΔaroA S. Typhimurium were re-stimulated with media (control), or Salmonella antigens. Splenocytes were stained intracellularly for IFN-γ. Pools of indicated CD4+ T cells producing IFN-γ were analyzed by FACS like in S8 Fig. The results were analyzed by two-way ANOVA (GraphPad Prism 9) for statistical significance, where the p-values are indicated as follows: * p≤ 0.05; ** p ≤ 0.01; *** p≤ 0.001; **** p ≤ 0.0001. (TIF) [file ppat.1009465.s010.TIF]

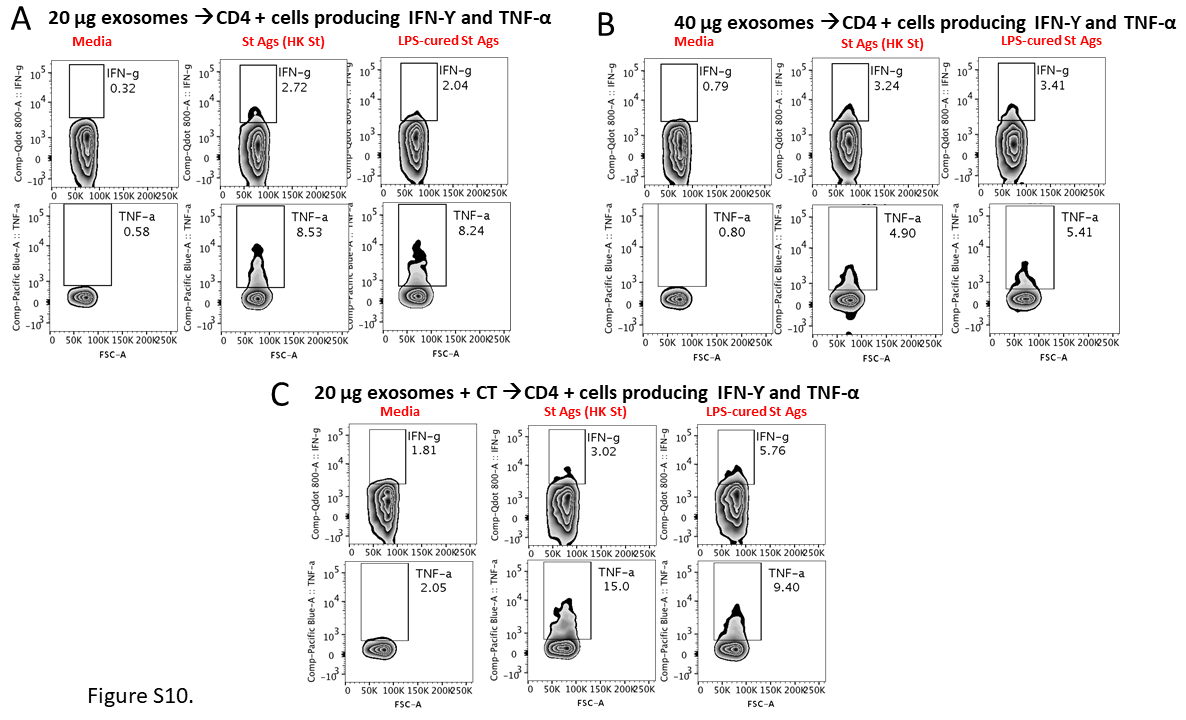

Supplement: S10 Fig — Examples of individual plots of FACS analysis of the TNF-α- and IFN-γ-producing CD4+ T lymphocytes from the spleen of mice immunized with exosomes derived from infected macrophages. (A)-(C). FACS analysis of T lymphocytes from the spleen of mice immunized with exosomes at the doses indicated on figures (20 μg, 40 μg, 20 μg with CT adjuvant, 40 μg with CT adjuvant, or PBS control) as well as mice immunized with live attenuated Salmonella vaccine (ΔaroA S. Typhimurium). Exosomes were derived from RAW264.7 macrophages infected with S. Typhimurium (pooled 24–48 hpi). Lymphocytes from the spleen were isolated and re-stimulated with Heat-killed S. Typhimurium (St Ags (HK St), LPS-detoxified S. Typhimurium antigens (LPS-cured St Ags), or a media control, and CD4+ T lymphocytes were analyzed for the secretion of TNF-α- and IFN-γ. The gating for analysis was done as in Fig 6. (TIF) [file ppat.1009465.s011.TIF]

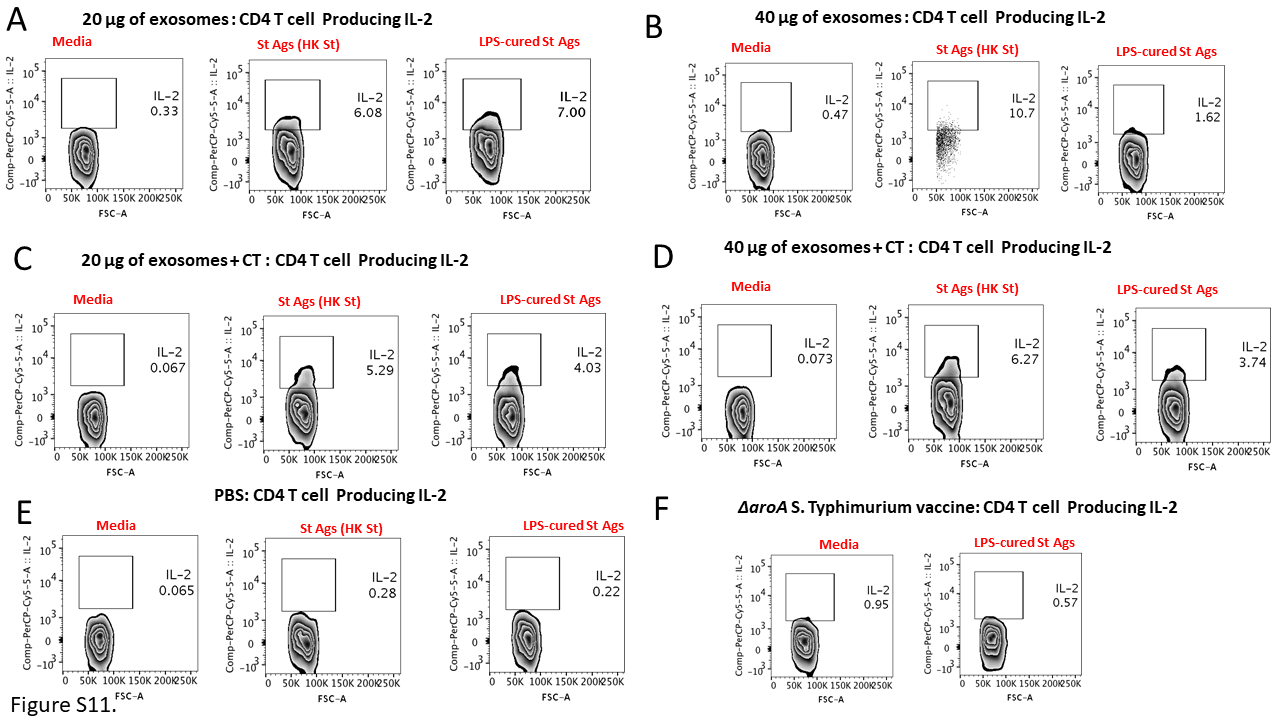

Supplement: S11 Fig — Examples of individual plots of FACS analysis of IL-2-producing CD4+ T lymphocytes from the spleen of mice immunized with exosomes derived from infected macrophages. (A)-(F). FACS analysis of T lymphocytes from the spleen of mice immunized with exosomes at the doses indicated on figures (20 μg, 40 μg, 20 μg with CT adjuvant, 40 μg with CT adjuvant, or PBS control) as well as mice immunized with live attenuated Salmonella vaccine (ΔaroA S. Typhimurium). Exosomes were derived from RAW264.7 macrophages infected with S. Typhimurium (pooled 24–48 hpi). Lymphocytes from the spleen were isolated and re-stimulated with Heat-killed S. Typhimurium (St Ags (HK St), LPS-detoxified S. Typhimurium antigens (LPS-cured St Ags), or a media control, and CD4+ T lymphocytes were analyzed for the secretion of IL-2. The gating for analysis was performed as shown in Fig 6. (TIF) [file ppat.1009465.s012.TIF]

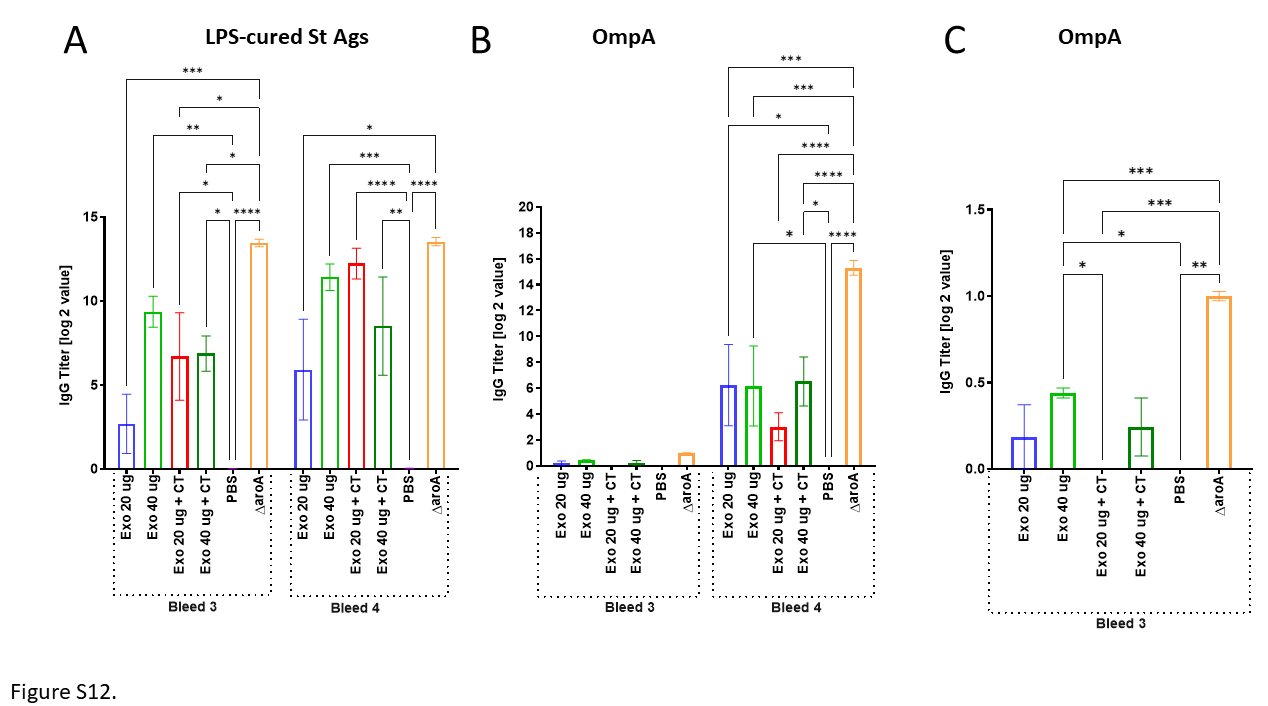

Supplement: S12 Fig — Exosomes derived from S. Typhimurium-infected macrophages induce the production of Salmonella-specific antibodies. Production of antibodies by mice immunized with exosomes with or without adjuvant (CT), PBS, or aroA-deficient S. Typhimurium. The analyzed antibodies were against (A) LPS-cured S. Typhimurium and (B-C) Outer Membrane Protein A (OmpA), all analyzed for bleed 3 and 4 (Fig 5). (TIF) [file ppat.1009465.s013.TIF]
