# Supplementary material for: Antigen-encapsulating host extracellular vesicles derived from Salmonella-infected cells stimulate pathogen-specific Th1-type responses in vivo
Source: PLoS Pathog. 2021 May 6;17(5):e1009465. doi: 10.1371/journal.ppat.1009465 (PMC8101724; doi:10.1371/journal.ppat.1009465)
Supplement: S1 Table — Calculated fold change was based on the normalized spectral values, and p-values were calculated by using Fisher’s test. ID, Symbol, Entrez Gene Name as well as predicted protein type and targeting drugs have been shown (Ingenuity Pathway Analysis, Qiagen). (PDF) [file ppat.1009465.s014.pdf]

Table S1

Expr p-value	Expr Fold Change	ID	Symbol	Entrez Gene Name	Type(s)
0.59	-2	Q9D2R0	AACS	acetoacetyl-CoA synthetase	enzyme
0.29	-1.25	Q8BGQ7	AARS	alanyl-tRNA synthetase	enzyme
0.82	1	P41233	ABCA1	ATP binding cassette subfamily A member 1	transporter
0.14	-3.333	Q8R420	ABCA3	ATP binding cassette subfamily A member 3	transporter
0.24	-2.5	P06795	Abcb1b	ATP-binding cassette, sub-family B (MDR/TAP), member 1B	transporter
0.59	-2	O35379	ABCC1	ATP binding cassette subfamily C member 1	transporter
0.47	1.7	P70170	ABCC9	ATP binding cassette subfamily C member 9	ion channel
0.23	1.5	P61222	ABCE1	ATP binding cassette subfamily E member 1	transporter
0.036	3.3	Q6P542	ABCF1	ATP binding cassette subfamily F member 1	transporter
0.24	4.4	Q99LE6	ABCF2	ATP binding cassette subfamily F member 2	transporter
0.32	-1.429	Q64343	ABCG1	ATP binding cassette subfamily G member 1	transporter
0.82	1	Q99JW1	ABHD17A	abhydrolase domain containing 17A	enzyme
0.82	1	Q7M759	ABHD17B	abhydrolase domain containing 17B	peptidase
0.82	1	Q5SSL4	ABR	ABR, RhoGEF and GTPase activating protein	other
0.53	1.1	Z4YJY0	ABRAXAS2	abraxas 2, BRISC complex subunit	other
0.051	-2	Q921H8	ACAA1	acetyl-CoA acyltransferase 1	enzyme
0.14	2.9	Q5SWU9	ACACA	acetyl-CoA carboxylase alpha	enzyme
0.82	1	Q8QZT1	ACAT1	acetyl-CoA acetyltransferase 1	enzyme
0.033	5.3	A0A0R4J079	ACBD3	acyl-CoA binding domain containing 3	other
0.34	1.1	Q3V117	ACLY	ATP citrate lyase	enzyme
0.0056	7.2	P28271	ACO1	aconitase 1	enzyme
0.051	7.1	Q99KI0	ACO2	aconitase 2	enzyme
0.47	3.3	P54987	ACOD1	aconitate decarboxylase 1	enzyme
0.51	1.1	Q91V12	ACOT7	acyl-CoA thioesterase 7	enzyme
0.11	-2	Q9QUJ7	ACSL4	acyl-CoA synthetase long chain family member 4	enzyme
0.51	1.6	P68134	ACTA1	actin, alpha 1, skeletal muscle	other
0.19	2.3	Q8BFZ3	ACTBL2	actin, beta like 2	other
0.0001	1.4	P63260	ACTG1	actin gamma 1	other
0.22	1.9	Q9Z2N8	ACTL6A	actin like 6A	other
0.00069	-3.333	A0A1L1SV25	ACTN4	actinin alpha 4	transcription regulator
0.47	1.6	P61164	ACTR1A	ARP1 actin related protein 1 homolog A	other

Table S1

0.59	1.4	Q8R5C5	ACTR1B	ARP1 actin related protein 1 homolog B	other
0.43	1.8	P61161	ACTR2	ARP2 actin related protein 2 homolog	other
0.54	1.1	Q99JY9	ACTR3	ARP3 actin related protein 3 homolog	other
0.64	1	Q641P0	ACTR3B	ARP3 actin related protein 3 homolog B	other
0.0053	-3.333	O35598	ADAM10	ADAM metallopeptidase domain 10	peptidase
0.0047	-5	Q3U7G2	ADAM8	ADAM metallopeptidase domain 8	peptidase
0.24	4.3	Q99MU3	ADAR	adenosine deaminase, RNA specific	enzyme
0.59	1.4	P28474	ADH5	alcohol dehydrogenase 5 (class III), chi polypeptide	enzyme
0.62	2.2	P55264	ADK	adenosine kinase	kinase
0.12	1.7	P54822	ADSL	adenylosuccinate lyase	enzyme
0.47	1.2	P46664	ADSS	adenylosuccinate synthase	enzyme
0.3	2.5	J3QN31	ADSSL1	adenylosuccinate synthase like 1	enzyme
0.0001	-25	M0QWP1	AGRN	agrin	other
0.017	2.7	P50247	AHCY	adenosylhomocysteinase	enzyme
0.35	-1.25	F8WGT1	AHCYL2	adenosylhomocysteinase like 2	enzyme
0.0001	-5	E9Q616	AHNAK	AHNAK nucleoprotein	other
0.53	-1.667	Q8BK64	AHSA1	activator of HSP90 ATPase activity 1	other
0.32	-1.25	Q3UZG4	AIMP1	aminoacyl tRNA synthetase complex interacting multifunctional protein 1	cytokine
0.56	1.1	Q8R010	AIMP2	aminoacyl tRNA synthetase complex interacting multifunctional protein 2	other
0.3	2.1	O88845	AKAP10	A-kinase anchoring protein 10	other
0.14	-5	Q9JII6	AKR1A1	aldo-keto reductase family 1 member A1	enzyme
0.34	1.9	P45376	AKR1B1	aldo-keto reductase family 1 member B	enzyme
0.34	3.6	P45377	AKR1B10	aldo-keto reductase family 1 member B10	enzyme
0.59	-1.429	Q8CG76	AKR7A2	aldo-keto reductase family 7 member A2	enzyme
0.0001	-3.333	ALBU_BOVIN	ALB	albumin	transporter
0.076	-2	ALBU_HUMAN	ALB	albumin	transporter
0.23	-2	E9Q3Q6	ALCAM	activated leukocyte cell adhesion molecule	other
0.36	-1.111	A0A1B0GSU0	ALDH16A1	aldehyde dehydrogenase 16 family member A1	enzyme
0.042	-10	Q80VQ0	ALDH3B1	aldehyde dehydrogenase 3 family member B1	enzyme

Table S1

0.29	1.5	Q3U367	ALDH9A1	aldehyde dehydrogenase 9 family member A1	enzyme
0.0001	-3.333	A6ZI44	ALDOA	aldolase, fructose-bisphosphate A	enzyme
0.042	-5	P05063	ALDOC	aldolase, fructose-bisphosphate C	enzyme
0.51	1.8	Q8JZV7	AMDHD2	amidohydrolase domain containing 2	enzyme
0.62	1.2	A0A1L1SRX2	AMPD3	adenosine monophosphate deaminase 3	enzyme
0.62	2.2	A0A087WNU5	ANK3	ankyrin 3	other
0.13	-1.429	Q810B6	ANKFY1	ankyrin repeat and FYVE domain containing 1	transcription regulator
0.14	1.8	Q9JHZ2	ANKH	ANKH inorganic pyrophosphate transport regulator	transporter
0.82	1	Q3U0L2	ANKRD33B	ankyrin repeat domain 33B	other
0.59	-2.5	Q6P9J9	ANO6	anoctamin 6	ion channel
0.47	2	Q9EST5	Anp32b	acidic (leucine-rich) nuclear phosphoprotein 32 family, member B	other
0.82	1	P97822	Anp32e	acidic (leucine-rich) nuclear phosphoprotein 32 family, member E	other
0.012	-1.667	P97449	ANPEP	alanyl aminopeptidase, membrane	peptidase
0.59	-1.111	Q6DFX2	ANTXR2	ANTXR cell adhesion molecule 2	transmembrane receptor
0.0001	-2	P10107	ANXA1	annexin A1	enzyme
0.0001	-3.333	P97384	ANXA11	annexin A11	other
0.00059	-1.429	P07356	ANXA2	annexin A2	other
0.54	1	O35639	ANXA3	annexin A3	enzyme
0.0001	-3.333	P97429	ANXA4	annexin A4	other
0.38	-3.333	ANXA5_HUMAN	ANXA5	annexin A5	transporter
0.0001	-2.5	P48036	ANXA5	annexin A5	transporter
0.0001	-5	F8WIT2	ANXA6	annexin A6	ion channel
0.0001	-2.5	Q07076	ANXA7	annexin A7	ion channel
0.61	1.3	Q5SVG4	AP1B1	adaptor related protein complex 1 subunit beta 1	transporter
0.36	-1.667	Q8CBB7	AP1G1	adaptor related protein complex 1 subunit gamma 1	transporter
0.39	1.3	P17426	AP2A1	adaptor related protein complex 2 subunit alpha 1	transporter
0.61	1.3	P17427	AP2A2	adaptor related protein complex 2 subunit alpha 2	transporter
0.052	2.7	H3BKM0	Ap2b1	adaptor-related protein complex 2, beta 1 subunit	other
0.53	-1.111	Q3TWV4	AP2M1	adaptor related protein complex 2 subunit mu 1	transporter
0.64	1.4	Q9Z1T1	AP3B1	adaptor related protein complex 3 subunit beta 1	transporter

Table S1

0.13	2.6	O54774	AP3D1	adaptor related protein complex 3 subunit delta 1	transporter
0.59	1.4	A0A0R4J107	APEH	acylaminoacyl-peptide hydrolase	peptidase
0.78	1.8	F6QA74	APEX1	apurinic/apyrimidinic endodeoxyribonuclease 1	enzyme
0.031	1.8	P08030	APRT	adenine phosphoribosyltransferase	enzyme
0.12	5.7	A2AQA7	AQR	aquarius intron-binding spliceosomal factor	other
0.62	2.4	Q4LDD4	ARAP1	ArfGAP with RhoGAP domain, ankyrin repeat and PH domain 1	other
0.24	4.6	Q5XJY5	ARCN1	archain 1	other
0.15	-2	P61205	ARF3	ADP ribosylation factor 3	enzyme
0.53	-1.111	A2AH25	ARHGAP1	Rho GTPase activating protein 1	other
0.82	1	E9QAJ9	ARHGAP17	Rho GTPase activating protein 17	other
0.53	-1.25	E9QMX7	ARHGAP30	Rho GTPase activating protein 30	other
0.46	-1.111	G3X9Q3	ARHGAP45	Rho GTPase activating protein 45	transporter
0.026	-3.333	Q99PT1	ARHGDIA	Rho GDP dissociation inhibitor alpha	other
0.11	-2	Q61599	ARHGDIB	Rho GDP dissociation inhibitor beta	enzyme
0.0083	3.1	E9PUF7	ARHGEF1	Rho guanine nucleotide exchange factor 1	other
0.34	3.6	A2AWP8	ARHGEF10L	Rho guanine nucleotide exchange factor 10 like	enzyme
0.34	2.1	H3BKH9	ARHGEF2	Rho/Rac guanine nucleotide exchange factor 2	other
0.46	1	F6WMJ3	ARHGEF6	Rac/Cdc42 guanine nucleotide exchange factor 6	other
0.53	-1.111	Q9ES28	ARHGEF7	Rho guanine nucleotide exchange factor 7	other
0.23	-1.429	P61211	ARL1	ADP ribosylation factor like GTPase 1	enzyme
0.14	-3.333	Q91Z25	ARPC1B	actin related protein 2/3 complex subunit 1B	other
0.59	-1.429	D3YXG6	ARPC2	actin related protein 2/3 complex subunit 2	other
0.076	-5	P59999	ARPC4	actin related protein 2/3 complex subunit 4	other
0.051	-3.333	Q99KN1	ARRDC1	arrestin domain containing 1	other
0.59	1.4	Q7TPQ9	ARRDC3	arrestin domain containing 3	other
0.59	1.4	O54984	ASNA1	arsA arsenite transporter, ATP-binding, homolog 1 (bacterial)	transporter
0.52	1	Q61024	ASNS	asparagine synthetase (glutamine-hydrolyzing)	enzyme

Table S1

0.82	1	D6REV1	ASXL2	ASXL transcriptional regulator 2	other
0.82	1	Q9CPX6	ATG3	autophagy related 3	enzyme
0.82	1	Q9D906	ATG7	autophagy related 7	enzyme
0.33	-1.25	Q9CWJ9	ATIC	5-aminoimidazole-4-carboxamide ribonucleotide formyltransferase/IMP cyclohydrolase	enzyme
0.0001	-1.667	Q8VDN2	ATP1A1	ATPase Na+/K+ transporting subunit alpha 1	transporter
0.004	-2	P97370	ATP1B3	ATPase Na+/K+ transporting subunit beta 3	transporter
0.0035	-5	G5E829	ATP2B1	ATPase plasma membrane Ca2+ transporting 1	transporter
0.23	-2	Q3UZR5	ATP2C1	ATPase secretory pathway Ca2+ transporting 1	transporter
0.59	1	Q03265	ATP5F1A	ATP synthase F1 subunit alpha	transporter
0.13	2.1	P56480	ATP5F1B	ATP synthase F1 subunit beta	transporter
0.076	-5	Q9CYN9	ATP6AP2	ATPase H+ transporting accessory protein 2	transporter
0.14	-3.333	Q9Z1G4	ATP6V0A1	ATPase H+ transporting V0 subunit a1	transporter
0.034	-3.333	P51863	ATP6V0D1	ATPase H+ transporting V0 subunit d1	transporter
0.54	1	P50516	ATP6V1A	ATPase H+ transporting V1 subunit A	transporter
0.0097	-2	P62814	ATP6V1B2	ATPase H+ transporting V1 subunit B2	transporter
0.39	-1.25	A0A0N4SW07	ATP6V1E1	ATPase H+ transporting V1 subunit E1	transporter
0.48	-1.111	A0A0A6YX18	ATP6V1H	ATPase H+ transporting V1 subunit H	transporter
0.59	-1.111	A2AG68	ATP7A	ATPase copper transporting alpha	transporter
0.38	-3.333	Q9WU60	ATRN	attractin	other
0.64	1.6	P01887	B2M	beta-2-microglobulin	transmembrane receptor
0.64	-1.111	Q8K3W0	BABAM2	BRISC and BRCA1 A complex member 2	other
0.62	1.5	Q9JLV1	BAG3	BCL2 associated athanogene 3	other
0.47	1.4	A0A1B0GX81	BAG6	BCL2 associated athanogene 6	enzyme
0.38	-3.333	O54962	BANF1	barrier to autointegration factor 1	other
0.59	-1.429	Q91XV3	BASP1	brain abundant membrane attached signal protein 1	transcription regulator
0.042	-5	A0A1B0GTA4	BAX	BCL2 associated X, apoptosis regulator	transporter
0.3	2.1	S4R2P8	BIRC6	baculoviral IAP repeat containing 6	enzyme

Table S1

0.42	-1.25	Q9CY64	BLVRA	biliverdin reductase A	enzyme
0.39	1.5	P36895	BMPR1A	bone morphogenetic protein receptor type 1A	kinase
0.82	1	O35607	BMPR2	bone morphogenetic protein receptor type 2	kinase
0.62	2.1	P97452	BOP1	block of proliferation 1	other
0.1	-2.5	Q8K2Q7	BROX	BRO1 domain and CAAX motif containing	other
0.0026	-2.5	P18572	BSG	basigin (Ok blood group)	transporter
0.039	-2.5	Q8R2Q8	Bst2	bone marrow stromal cell antigen 2	other
0.62	2.6	P35991	BTK	Bruton tyrosine kinase	kinase
0.39	2.3	Q9WVA3	BUB3	BUB3, mitotic checkpoint protein	other
0.17	5	O54825	BYSL	bystin like	other
0.78	1.8	Q91VK1	BZW2	basic leucine zipper and W2 domains 2	translation regulator
0.042	-5	Q8C708	C16orf54	chromosome 16 open reading frame 54	other
0.59	-1.429	Q8C3W1	C1orf198	chromosome 1 open reading frame 198	other
0.23	-3.333	P01027	C3	complement C3	peptidase
0.77	1.2	P30993	C5AR1	complement C5a receptor 1	G-protein coupled receptor
0.38	-2	A0A0A0MQD4	C5orf15	chromosome 5 open reading frame 15	other
0.82	1	G3X8U3	C9orf64	chromosome 9 open reading frame 64	other
0.0046	-2	P00920	CA2	carbonic anhydrase 2	enzyme
0.59	-1.111	Q99246	CACNA1D	calcium voltage-gated channel subunit alpha1 D	ion channel
0.0001	4.8	B2RQC6	CAD	carbamoyl-phosphate synthetase 2, aspartate transcarbamylase, and dihydroorotase	enzyme
0.38	-1.667	Q91YS8	CAMK1	calcium/calmodulin dependent protein kinase I	kinase
0.51	1.2	E9Q1W0	CAMK2D	calcium/calmodulin dependent protein kinase II delta	kinase
0.3	2.2	Q6ZQ38	CAND1	cullin associated and neddylation dissociated 1	transcription regulator
0.22	-1.25	P40124	CAP1	cyclase associated actin cytoskeleton regulatory protein 1	other
0.0066	-1.667	Q99LB4	CAPG	capping actin protein, gelsolin like	other
0.46	-1.429	O08529	CAPN2	calpain 2	peptidase
0.82	1	A0A0R4IZW8	CAPNS1	calpain small subunit 1	peptidase
0.46	1.2	Q5RKN9	CAPZA1	capping actin protein of muscle Z-line subunit alpha 1	other
0.77	1.1	P47754	CAPZA2	capping actin protein of muscle Z-line subunit alpha 2	other

Table S1

0.15	-1.667	A2AMW0	CAPZB	capping actin protein of muscle Z-line subunit beta	other
0.38	-2.5	Q8CIS0	CARD11	caspase recruitment domain family member 11	kinase
0.34	3.6	A2AIV8	CARD9	caspase recruitment domain family member 9	other
0.62	1	D3YUP1	CARM1	coactivator associated arginine methyltransferase 1	transcription regulator
0.82	1	P24270	CAT	catalase	enzyme
0.59	-1.429	Q8K1A6	CC2D1A	coiled-coil and C2 domain containing 1A	transcription regulator
0.00044	-5	F6XC25	CC2D1B	coiled-coil and C2 domain containing 1B	transcription regulator
0.077	6.4	Q8VDP4	CCAR2	cell cycle and apoptosis regulator 2	peptidase
0.64	1.2	Q9JIG7	CCDC22	coiled-coil domain containing 22	other
0.34	1.9	Q4QRL3	CCDC88B	coiled-coil domain containing 88B	enzyme
0.49	-1.25	P51670	Ccl9	chemokine (C-C motif) ligand 9	cytokine
0.34	2.1	G3UY65	CCNB1	cyclin B1	kinase
0.0001	2.5	P80314	CCT2	chaperonin containing TCP1 subunit 2	kinase
0.013	1.4	P80318	CCT3	chaperonin containing TCP1 subunit 3	other
0.032	1.5	P80315	CCT4	chaperonin containing TCP1 subunit 4	other
0.00093	2.2	P80316	CCT5	chaperonin containing TCP1 subunit 5	other
0.028	1.5	P80317	CCT6A	chaperonin containing TCP1 subunit 6A	other
0.036	1.5	P80313	CCT7	chaperonin containing TCP1 subunit 7	other
0.025	1.5	P42932	CCT8	chaperonin containing TCP1 subunit 8	enzyme
0.25	-1.111	P10810	CD14	CD14 molecule	transmembrane receptor
0.59	-1.111	Q62192	CD180	CD180 molecule	other
0.0025	-10	Q9JLQ0	CD2AP	CD2 associated protein	other
0.59	-1.429	Q6SJQ0	CD300A	CD300a molecule	transmembrane receptor
0.59	-2	A2A6Z2	CD300LF	CD300 molecule like family member f	other
0.15	-2.5	Q08857	CD36	CD36 molecule	transmembrane receptor
0.82	1	P27512	CD40	CD40 molecule	transmembrane receptor
0.014	-3.333	A2APM2	CD44	CD44 molecule (Indian blood group)	other
0.15	-2	Q61735	CD47	CD47 molecule	transmembrane receptor
0.042	-5	P21855	CD72	CD72 molecule	transmembrane receptor
0.00029	-5	P35762	CD81	CD81 molecule	other
0.72	-1.111	P40237	CD82	CD82 molecule	other

Table S1

0.084	-1.429	P40240	CD9	CD9 molecule	other
0.49	-1.25	Q61081	CDC37	cell division cycle 37	kinase
0.14	-1.25	P60766	Cdc42	cell division cycle 42	enzyme
0.82	1	A0A1D5RLQ9	CDC42BPA	CDC42 binding protein kinase alpha	kinase
0.0011	3.2	P11440	CDK1	cyclin dependent kinase 1	kinase
0.34	3.6	P30285	CDK4	cyclin dependent kinase 4	kinase
0.62	2	Q64261	CDK6	cyclin dependent kinase 6	kinase
0.24	2.3	Q99J95	CDK9	cyclin dependent kinase 9	kinase
0.62	1.2	Q5FWI3	CEMIP2	cell migration inducing hyaluronidase 2	enzyme
0.076	-5	Q8BT07	CEP55	centrosomal protein 55	other
0.14	-5	Q8K4Q7	CERK	ceramide kinase	kinase
0.22	-1.429	P18760	CFL1	cofilin 1	other
0.00075	7.9	Q6PDQ2	CHD4	chromodomain helicase DNA binding protein 4	enzyme
0.14	-3.333	Q9DB34	CHMP2A	charged multivesicular body protein 2A	other
0.0087	-5	Q9D8B3	CHMP4B	charged multivesicular body protein 4B	other
0.82	1	A2A7F6	CLCN6	chloride voltage-gated channel 6	ion channel
0.21	-1.25	Q9Z1Q5	CLIC1	chloride intracellular channel 1	ion channel
0.095	-3.333	Q9QYB1	CLIC4	chloride intracellular channel 4	ion channel
0.15	-3.333	B1AWE0	CLTA	clathrin light chain A	other
0.0001	-1.667	Q68FD5	CLTC	clathrin heavy chain	other
0.0088	10	A0A0R4J140	CLUH	clustered mitochondria homolog	translation regulator
0.47	3.2	Q99KK2	CMAS	cytidine monophosphate N-acetylneuraminic acid synthetase	enzyme
0.38	-3.333	Q9D486	CMIP	c-Maf inducing protein	other
0.051	7.2	Q3U5Q7	CMPK2	cytidine/uridine monophosphate kinase 2	kinase
0.59	1.4	Q9DBC3	CMTR1	cap methyltransferase 1	enzyme
0.55	1	Q9D1A2	CNDP2	carnosine dipeptidase 2	peptidase
0.82	1	Q32NY4	CNNM3	cyclin and CBS domain divalent metal cation transport mediator 3	other
0.051	7.4	Q6ZQ08	CNOT1	CCR4-NOT transcription complex subunit 1	other
0.38	-1.667	Q8K4Q8	COLEC12	collectin subfamily member 12	transmembrane receptor
0.036	3.3	Q8K297	COLGALT1	collagen beta(1-O)galactosyltransferase 1	enzyme
0.0019	1.9	F8WHL2	COPA	coatomer protein complex subunit alpha	transporter
0.036	3.1	Q9JIF7	COPB1	coatomer protein complex subunit beta 1	transporter
0.14	1.6	O55029	COPB2	coatomer protein complex subunit beta 2	transporter

Table S1

0.62	2.2	O89079	COPE	coatomer protein complex subunit epsilon	transporter
0.013	1.9	Q9QZE5	COPG1	coatomer protein complex subunit gamma 1	transporter
0.022	5.8	Q9QXK3	COPG2	coatomer protein complex subunit gamma 2	transporter
0.47	1.3	O88543	COPS3	COP9 signalosome subunit 3	other
0.028	1.9	O89053	CORO1A	coronin 1A	other
0.34	3.6	Q9WUM3	CORO1B	coronin 1B	other
0.34	1.6	Q9WUM4	CORO1C	coronin 1C	other
0.051	7.5	Q9D2V7	CORO7/CORO7-PAM16	coronin 7	other
0.82	1	O89001	CPD	carboxypeptidase D	peptidase
0.0016	-3.333	Q8C166	CPNE1	copine 1	transporter
0.087	-3.333	A0A0R4J1D0	CPNE2	copine 2	other
0.39	-1.429	Q8BT60	CPNE3	copine 3	kinase
0.095	-1.667	Q9DC53	CPNE8	copine 8	other
0.34	2.1	Q9EPU4	CPSF1	cleavage and polyadenylation specific factor 1	other
0.62	2.5	O35218	CPSF2	cleavage and polyadenylation specific factor 2	other
0.62	2.5	H3BJ30	CPSF6	cleavage and polyadenylation specific factor 6	other
0.042	-5	Q64735	CR1L	complement C3b/C4b receptor 1 like	other
0.82	1	P47199	CRYZ	crystallin zeta	enzyme
0.051	4.9	Q9CZU6	CS	citrate synthase	enzyme
0.01	4	Q91W50	CSDE1	cold shock domain containing E1	enzyme
0.0014	6.8	Q9ERK4	CSE1L	chromosome segregation 1 like	transporter
0.022	8.7	P09920	CSF3	colony stimulating factor 3	cytokine
0.51	1.3	P41241	CSK	C-terminal Src kinase	kinase
0.00078	-11.111	CASB_BOVIN	CSN2	casein beta	kinase
0.46	-1.25	E9Q4G7	CSNK1A1	casein kinase 1 alpha 1	kinase
0.62	1.4	A0A0U1RP94	CSNK1G1	casein kinase 1 gamma 1	kinase
0.38	-3.333	Q8C4X2	CSNK1G3	casein kinase 1 gamma 3	kinase
0.59	-1.429	Q60737	CSNK2A1	casein kinase 2 alpha 1	kinase
0.59	-2	O54833	CSNK2A2	casein kinase 2 alpha 2	kinase
0.59	-2	Q62426	CSTB	cystatin B	peptidase
0.59	1.4	Q99LC2	CSTF1	cleavage stimulation factor subunit 1	other
0.78	1.7	A0A0J9YU62	CTBP1	C-terminal binding protein 1	enzyme
0.17	5	Q91YZ2	CTBP2	C-terminal binding protein 2	transcription regulator
0.78	1.8	Q8VCN5	CTH	cystathionine gamma-lyase	enzyme
0.82	1	P26231	CTNNA1	catenin alpha 1	other

Table S1

0.64	1.2	Q9CWL8	CTNNBL1	catenin beta like 1	other
0.0056	5.7	P70698	CTPS1	CTP synthase 1	enzyme
0.23	-2.5	P16675	CTSA	cathepsin A	peptidase
0.53	1.1	P10605	CTSB	cathepsin B	peptidase
0.11	-1.667	P18242	CTSD	cathepsin D	peptidase
0.038	2.2	P55097	CTSK	cathepsin K	peptidase
0.53	-1.25	O70370	CTSS	cathepsin S	peptidase
0.64	1.5	P06797	CTSV	cathepsin V	peptidase
0.62	1.4	G3UXB4	CTU2	cytosolic thiouridylase subunit 2	other
0.24	4.3	Q9D4H8	CUL2	cullin 2	enzyme
0.62	1.4	Q3TCH7	CUL4A	cullin 4A	other
0.38	-1.667	G3X914	CUL5	cullin 5	ion channel
0.23	-3.333	F2Z456	Cyb5r3	cytochrome b5 reductase 3	enzyme
0.1	-2.5	Q61462	CYBA	cytochrome b-245 alpha chain	enzyme
0.15	-2.5	Q61093	CYBB	cytochrome b-245 beta chain	enzyme
0.12	1.3	Q7TMB8	CYFIP1	cytoplasmic FMR1 interacting protein 1	other
0.77	1.2	Q8BPM0	DAAM1	dishevelled associated activator of morphogenesis 1	other
0.24	-3.333	E9QL31	DAB2	DAB2, clathrin adaptor protein	other
0.075	1.6	Q922B2	DARS	aspartyl-tRNA synthetase	enzyme
0.59	-2	Q3UGB5	Dazap1	DAZ associated protein 1	other
0.82	1	P61963	DCAF7	DDB1 and CUL4 associated factor 7	other
0.82	1	Q91ZV3	DCBLD2	discoidin, CUB and LCCL domain containing 2	other
0.59	1.4	P43346	DCK	deoxycytidine kinase	kinase
0.17	5.1	E9Q3M3	DCTN1	dynactin subunit 1	other
0.77	1.2	Q3U1J4	DDB1	damage specific DNA binding protein 1	other
0.14	2.1	Q91VR5	DDX1	DEAD-box helicase 1	enzyme
0.033	5.4	Q501J6	DDX17	DEAD-box helicase 17	enzyme
0.47	2.9	Q8K363	DDX18	DEAD-box helicase 18	enzyme
0.19	1.8	Q61655	DDX19A	DEAD-box helicase 19A	enzyme
0.0003	4	Q9JIK5	DDX21	DExD-box helicase 21	enzyme
0.074	2.6	Q8VDW0	DDX39A	DExD-box helicase 39A	enzyme
0.77	1.1	Q9Z1N5	DDX39B	DExD-box helicase 39B	enzyme
0.0001	4	Q62167	DDX3X	DEAD-box helicase 3 X-linked	enzyme
0.62	1.2	Q569Z5	DDX46	DEAD-box helicase 46	enzyme
0.13	2.5	Q9CWX9	DDX47	DEAD-box helicase 47	enzyme
0.0001	38	Q61656	DDX5	DEAD-box helicase 5	enzyme
0.47	1.9	Q9D0R4	DDX56	DEAD-box helicase 56	enzyme
0.47	1.7	Q6Q899	DDX58	DExD/H-box helicase 58	enzyme
0.24	2.8	P54823	DDX6	DEAD-box helicase 6	enzyme
0.17	5.1	A0A0R4J172	DENND4B	DENN domain containing 4B	other

Table S1

0.35	-2	Q91YP3	DERA	deoxyribose-phosphate aldolase	enzyme
0.0022	3	O35286	DHX15	DEAH-box helicase 15	enzyme
0.0035	7.6	Q6PGC1	DHX29	DExH-box helicase 29	enzyme
0.82	1	Q99PU8	DHX30	DExH-box helicase 30	enzyme
0.62	2.4	Q8K1G9	DHX35	DEAH-box helicase 35	enzyme
0.59	1.4	Q8VHK9	DHX36	DEAH-box helicase 36	enzyme
0.34	3.6	Q80X98	DHX38	DEAH-box helicase 38	enzyme
0.12	5.7	A2A4P0	DHX8	DEAH-box helicase 8	enzyme
0.0001	4	A0A087WPL5	DHX9	DExH-box helicase 9	enzyme
0.0001	-10	Q3UH60	DIP2B	disco interacting protein 2 homolog B	other
0.23	-2.5	B7ZMZ7	DIP2C	disco interacting protein 2 homolog C	other
0.17	5.1	Q9CSH3	DIS3	DIS3 homolog, exosome endoribonuclease and 3'-5' exoribonuclease	enzyme
0.47	-1.25	Q9ESX5	DKC1	dyskerin pseudouridine synthase 1	enzyme
0.38	-2.5	Q8BMF4	DLAT	dihydrolipoamide S-acetyltransferase	enzyme
0.82	1	O08749	DLD	dihydrolipoamide dehydrogenase	enzyme
0.62	2.6	B9EJR8	DNAAF5	dynein axonemal assembly factor 5	other
0.36	1	P63037	DNAJA1	DnaJ heat shock protein family (Hsp40) member A1	other
0.33	-1.429	Q9QYJ0	DNAJA2	DnaJ heat shock protein family (Hsp40) member A2	enzyme
0.34	2.5	Q9QYJ3	DNAJB1	DnaJ heat shock protein family (Hsp40) member B1	transcription regulator
0.23	-1.667	G3X922	DNAJC13	DnaJ heat shock protein family (Hsp40) member C13	other
0.62	1	Q9QYI3	DNAJC7	DnaJ heat shock protein family (Hsp40) member C7	other
0.052	3.5	E9PUD2	DNM1L	dynamin 1 like	enzyme
0.0019	4.3	P39054	DNM2	dynamin 2	enzyme
0.00051	14	P13864	DNMT1	DNA methyltransferase 1	enzyme
0.23	-3.333	Q9Z2W0	DNPEP	aspartyl aminopeptidase	peptidase
0.54	1.5	Q8BZN6	DOCK10	dedicator of cytokinesis 10	other
0.0078	4.3	Q8C3J5	DOCK2	dedicator of cytokinesis 2	other
0.24	2.6	A0A0U1RNK7	DOCK7	dedicator of cytokinesis 7	other
0.077	6.6	O70469	DOK2	docking protein 2	other
0.62	1.4	H7BWZ9	DOP1A	DOP1 leucine zipper like protein A	other
0.82	1	Q5NCQ5	DPH1	diphthamide biosynthesis 1	other
0.82	1	Q99KK7	DPP3	dipeptidyl peptidase 3	peptidase
0.42	-1.111	O08553	DPYSL2	dihydropyrimidinase like 2	enzyme

Table S1

0.095	-2.5	P32233	DRG1	developmentally regulated GTP binding protein 1	other
0.022	5.8	Q9QXB9	DRG2	developmentally regulated GTP binding protein 2	other
0.82	1	Q6PDK8	DTX4	deltex E3 ubiquitin ligase 4	enzyme
0.24	4.4	A0A0R4IZY9	DUS3L	dihydrouridine synthase 3 like	other
0.34	2.4	B1AQF4	DUSP3	dual specificity phosphatase 3	phosphatase
0.0001	2.1	Q9JHU4	DYNC1H1	dynein cytoplasmic 1 heavy chain 1	peptidase
0.34	3.6	Q3TPJ8	Dync1i2	dynein cytoplasmic 1 intermediate chain 2	other
0.17	1.5	Q8R1Q8	DYNC1LI1	dynein cytoplasmic 1 light intermediate chain 1	other
0.49	-1.429	Q6PDL0	DYNC1LI2	dynein cytoplasmic 1 light intermediate chain 2	other
0.00075	5.1	Q6PDI5	ECPAS	Ecm29 proteasome adaptor and scaffold	other
0.077	3.5	Q3UJB9	EDC4	enhancer of mRNA decapping 4	other
0.0001	-2.5	Q8C4U8	EDIL3	EGF like repeats and discoidin domains 3	other
0.0041	1.5	P10126	EEF1A1	eukaryotic translation elongation factor 1 alpha 1	translation regulator
0.41	1.2	F6ZFU0	EEF1D	eukaryotic translation elongation factor 1 delta	translation regulator
0.032	1.7	Q9D8N0	EEF1G	eukaryotic translation elongation factor 1 gamma	translation regulator
0.066	1.3	P58252	EEF2	eukaryotic translation elongation factor 2	translation regulator
0.59	-1.429	Q8C845	EFHD2	EF-hand domain family member D2	other
0.82	1	Q8C0D5	EFL1	elongation factor like GTPase 1	translation regulator
0.0029	7	O08810	EFTUD2	elongation factor Tu GTP binding domain containing 2	enzyme
0.53	1	E9QP49	Ehbp1l1	EH domain binding protein 1-like 1	other
0.22	-1.111	Q9WVK4	EHD1	EH domain containing 1	other
0.26	1.4	Q8BH64	EHD2	EH domain containing 2	other
0.04	-1.429	Q9EQP2	EHD4	EH domain containing 4	enzyme
0.24	4.3	Q8BJW6	EIF2A	eukaryotic translation initiation factor 2A	translation regulator
0.78	1.7	Q03963	EIF2AK2	eukaryotic translation initiation factor 2 alpha kinase 2	kinase
0.077	4.4	Q99LC8	EIF2B1	eukaryotic translation initiation factor 2B subunit alpha	translation regulator
0.82	1	B1AUN2	EIF2B3	eukaryotic translation initiation factor 2B subunit gamma	other

Table S1

0.64	1.3	Q61749	EIF2B4	eukaryotic translation initiation factor 2B subunit delta	other
0.51	1.2	Q6ZWX6	EIF2S1	eukaryotic translation initiation factor 2 subunit alpha	translation regulator
0.051	4.8	Q9Z0N1	EIF2S3	eukaryotic translation initiation factor 2 subunit gamma	translation regulator
0.19	1.4	P23116	EIF3A	eukaryotic translation initiation factor 3 subunit A	other
0.37	-1.25	Q8JZQ9	EIF3B	eukaryotic translation initiation factor 3 subunit B	translation regulator
0.0022	2.6	Q8R1B4	EIF3C	eukaryotic translation initiation factor 3 subunit C	translation regulator
0.16	1.4	O70194	EIF3D	eukaryotic translation initiation factor 3 subunit D	other
0.29	1.2	P60229	EIF3E	eukaryotic translation initiation factor 3 subunit E	other
0.44	1.1	Q9DCH4	EIF3F	eukaryotic translation initiation factor 3 subunit F	translation regulator
0.19	-1.429	Q91WK2	EIF3H	eukaryotic translation initiation factor 3 subunit H	other
0.52	1.1	Q9QZD9	EIF3I	eukaryotic translation initiation factor 3 subunit I	translation regulator
0.12	1.5	Q8QZY1	EIF3L	eukaryotic translation initiation factor 3 subunit L	other
0.47	3	Q99JX4	EIF3M	eukaryotic translation initiation factor 3 subunit M	other
0.0001	2.3	P60843	EIF4A1	eukaryotic translation initiation factor 4A1	translation regulator
0.82	1	P10630	EIF4A2	eukaryotic translation initiation factor 4A2	translation regulator
0.00032	15	Q91VC3	EIF4A3	eukaryotic translation initiation factor 4A3	enzyme
0.15	-2.5	Q8BGD9	EIF4B	eukaryotic translation initiation factor 4B	translation regulator
0.59	1.4	P63073	EIF4E	eukaryotic translation initiation factor 4E	translation regulator
0.0023	2.9	Q6NZJ6	EIF4G1	eukaryotic translation initiation factor 4 gamma 1	translation regulator
0.62	2	G3XA17	EIF4G2	eukaryotic translation initiation factor 4 gamma 2	translation regulator
0.59	1.4	P59325	EIF5	eukaryotic translation initiation factor 5	translation regulator
0.36	-1.667	A0A0A0MQM0	EIF5A	eukaryotic translation initiation factor 5A	translation regulator

Table S1

0.34	3.6	Q05D44	EIF5B	eukaryotic translation initiation factor 5B	translation regulator
0.61	-1.111	O55135	EIF6	eukaryotic translation initiation factor 6	translation regulator
0.82	1	Q80Y81	ELAC2	elaC ribonuclease Z 2	enzyme
0.64	1.4	P70372	ELAVL1	ELAV like RNA binding protein 1	other
0.62	2	Q8BPU7	ELMO1	engulfment and cell motility 1	other
0.62	2.6	Q7TT37	ELP1	elongator complex protein 1	other
0.34	3.7	Q9CZX0	ELP3	elongator acetyltransferase complex subunit 3	enzyme
0.016	-3.333	Q8K482	EMILIN2	elastin microfibril interfacer 2	other
0.15	-1.111	P17182	ENO1	enolase 1	enzyme
0.59	-2	Q8BTJ4	ENPP4	ectonucleotide pyrophosphatase/phosphodiesterase 4	enzyme
0.24	-2.5	A2A841	EPB41	erythrocyte membrane protein band 4.1	other
0.62	2	Q3UV95	EPB42	erythrocyte membrane protein band 4.2	transporter
0.0049	1.5	Q8CGC7	EPRS	glutamyl-prolyl-tRNA synthetase	enzyme
0.59	-1.429	Q08509	EPS8	epidermal growth factor receptor pathway substrate 8	peptidase
0.62	1.2	B7ZNX6	ERBIN	erbb2 interacting protein	other
0.17	1.5	H3BKH6	ESD	esterase D	enzyme
0.0031	-10	Q3U7R1	ESYT1	extended synaptotagmin 1	other
0.38	-3.333	Q8BWY3	ETF1	eukaryotic translation termination factor 1	translation regulator
0.045	-2.5	P20934	EVI2A	ecotropic viral integration site 2A	transmembrane receptor
0.49	-1.25	O35382	EXOC4	exocyst complex component 4	transporter
0.82	1	Q9CRA8	EXOSC5	exosome component 5	enzyme
0.53	-1.25	F7AQX0	EZH2	enhancer of zeste 2 polycomb repressive complex 2 subunit	transcription regulator
0.23	-5	P26040	EZR	ezrin	other
0.43	1.4	O88792	F11R	F11 receptor	other
0.38	-2.5	Q05816	FABP5	fatty acid binding protein 5	transporter
0.0022	12	Q6A0A9	FAM120A	family with sequence similarity 120A	other
0.46	-1.25	E9PYV4	FAM129A	family with sequence similarity 129 member A	other
0.35	-2	Q8R1F1	FAM129B	family with sequence similarity 129 member B	transcription regulator
0.14	-3.333	Q921M7	FAM49B	family with sequence similarity 49 member B	other
0.62	2.1	A0A0N4SV29	FANCD2	FA complementation group D2	other

Table S1

0.018	3.3	Q8C0C7	FARSA	phenylalanyl-tRNA synthetase subunit alpha	enzyme
0.41	1.2	Q9WUA2	FARSB	phenylalanyl-tRNA synthetase subunit beta	enzyme
0.0001	1.9	P19096	FASN	fatty acid synthase	enzyme
0.51	1.8	P35550	FBL	fibrillarin	enzyme
0.62	2.1	Q61555	FBN2	fibrillin 2	other
0.21	-1.667	P20491	FCER1G	Fc fragment of IgE receptor Ig	transmembrane receptor
0.034	-3.333	P26151	FCGR1A	Fc fragment of IgG receptor Ia	transmembrane receptor
0.46	1.1	A0A0B4J1G1	FCGR2B	Fc fragment of IgG receptor IIb	transmembrane receptor
0.11	-1.667	Q920E5	FDPS	farnesyl diphosphate synthase	enzyme
0.077	6.9	Q91Z50	FEN1	flap structure-specific endonuclease 1	enzyme
0.051	-1.667	Q8K1B8	FERMT3	fermitin family member 3	enzyme
0.11	-2	P30416	FKBP4	FK506 binding protein 4	enzyme
0.51	1.6	Q64378	FKBP5	FK506 binding protein 5	enzyme
0.34	3.8	Q9JJ28	FLII	FLII, actin remodeling protein	other
0.46	1	Q8BTM8	FLNA	filamin A	other
0.022	-5	G3UYU4	FLOT1	flotillin 1	other
0.38	-3.333	Q60634	FLOT2	flotillin 2	other
0.59	1.4	A0A0R4J0A4	FLT1	fms related tyrosine kinase 1	kinase
0.4	-1.111	A2AB60	Fmnl1	formin-like 1	other
0.23	-5	D3Z7A7	FMNL3	formin like 3	other
0.17	5.1	E9QAT0	FMR1	fragile X mental retardation 1	translation regulator
0.59	-1.429	A0A0R4J0H8	FNDC3B	fibronectin type III domain containing 3B	other
0.82	1	Q920B0	FRMD4B	FERM domain containing 4B	other
0.82	1	Q8K385	FRRS1	ferric chelate reductase 1	transmembrane receptor
0.59	1.1	P09528	FTH1	ferritin heavy chain 1	enzyme
0.62	2.2	Q99LJ1	FUCA1	alpha-L-fucosidase 1	enzyme
0.62	2.5	Q7TMC8	FUK	fucokinase	kinase
0.48	-1.25	P39688	FYN	FYN proto-oncogene, Src family tyrosine kinase	kinase
0.12	3.9	P97855	G3BP1	G3BP stress granule assembly factor 1	enzyme
0.22	1.9	P97379	G3BP2	G3BP stress granule assembly factor 2	enzyme
0.42	1.1	Q00612	G6PD	glucose-6-phosphate dehydrogenase	enzyme
0.62	1.4	P70699	GAA	glucosidase alpha, acid	enzyme
0.53	-1.25	P60521	GABARAPL2	GABA type A receptor associated protein like 2	other
0.82	1	A0A0R4J0F6	GAK	cyclin G associated kinase	kinase
0.066	2.3	Q9R0N0	GALK1	galactokinase 1	kinase
0.38	-2.5	Q80VA0	GALNT7	polypeptide N-acetylgalactosaminyltransferase 7	enzyme

Table S1

0.62	2.5	Q8BHN3	GANAB	glucosidase II alpha subunit	enzyme
0.4	1	P16858	GAPDH	glyceraldehyde-3-phosphate dehydrogenase	enzyme
0.34	2.4	D3YZ09	Gar1	GAR1 ribonucleoprotein	other
0.14	1.3	Q9CZD3	GARS	glycyl-tRNA synthetase	enzyme
0.32	1.3	Q64737	GART	phosphoribosylglycinamide formyltransferase, phosphoribosylglycinamide synthetase, phosphoribosylaminoimidazole synthetase	enzyme
0.051	-3.333	Q3U432	GAS7	growth arrest specific 7	transcription regulator
0.042	-5	P17439	GBA	glucosylceramidase beta	enzyme
0.72	-1.111	O09172	GCLM	glutamate-cysteine ligase modifier subunit	enzyme
0.0001	8.2	E9PVA8	GCN1	GCN1, eIF2 alpha kinase activator homolog	translation regulator
0.00063	-2.5	Q61598	GDI2	GDP dissociation inhibitor 2	other
0.00037	3.9	P47856	GFPT1	glutamine--fructose-6-phosphate transaminase 1	enzyme
0.82	1	Q9JLQ2	GIT2	GIT ArfGAP 2	other
0.82	1	P23242	GJA1	gap junction protein alpha 1	transporter
0.82	1	Q8BGZ6	GLA	galactosidase alpha	enzyme
0.14	-5	Q9CYL5	GLIPR2	GLI pathogenesis related 2	other
0.53	-1.667	Q9JHJ3	GLMP	glycosylated lysosomal membrane protein	transcription regulator
0.065	1.6	A0A1Y7VKY1	Gm11361	ribosomal protein S18 pseudogene	other
0.29	1.3	V9GXQ2	Gm17087	predicted gene 17087	other
0.53	1.1	A0A0A6YVU8	Gm9774	adhesion regulating molecule 1 pseudogene	other
0.077	6.4	Q8K0C9	GMDS	GDP-mannose 4,6-dehydratase	enzyme
0.72	-1.111	Q922H4	GMPPA	GDP-mannose pyrophosphorylase A	enzyme
0.036	4.7	Q8BTZ7	GMPPB	GDP-mannose pyrophosphorylase B	enzyme
0.82	1	Q9DCZ1	GMPR	guanosine monophosphate reductase	enzyme
0.47	3	Q99L27	GMPR2	guanosine monophosphate reductase 2	enzyme
0.053	4.3	Q3THK7	GMPS	guanine monophosphate synthase	enzyme
0.11	-1.667	P27601	GNA13	G protein subunit alpha 13	enzyme
0.13	-1.25	P08752	GNAI2	G protein subunit alpha i2	enzyme
0.029	-2.5	Q9DC51	GNAI3	G protein subunit alpha i3	enzyme
0.051	-2	P21279	GNAQ	G protein subunit alpha q	enzyme
0.054	-2	Q6R0H7	GNAS	GNAS complex locus	enzyme

Table S1

0.27	-1.429	P62874	GNB1	G protein subunit beta 1	enzyme
0.075	-1.667	P62880	GNB2	G protein subunit beta 2	enzyme
0.022	8.7	P36916	GNL1	G protein nucleolar 1 (putative)	other
0.3	2.5	Q99LH1	GNL2	G protein nucleolar 2	enzyme
0.34	3.6	Q8CI11	GNL3	G protein nucleolar 3	other
0.49	-2	Q91W53	GOLGA7	golgin A7	other
0.38	-2.5	Q9CR60	GOLT1B	golgi transport 1B	other
0.82	1	O88630	GOSR1	golgi SNAP receptor complex member 1	transporter
0.0047	-5	P05201	GOT1	glutamic-oxaloacetic transaminase 1	enzyme
0.38	-2.5	Q3ULJ0	GPD1L	glycerol-3-phosphate dehydrogenase 1 like	enzyme
0.0001	-3.333	P06745	GPI	glucose-6-phosphate isomerase	enzyme
0.076	-5	Q99P91	GPNMB	glycoprotein nmb	enzyme
0.82	1	A0A0R4J100	GPR84	G protein-coupled receptor 84	G-protein coupled receptor
0.35	-2	G3UXW9	GPS1	G protein pathway suppressor 1	other
0.022	-10	B1AT92	GRB2	growth factor receptor bound protein 2	kinase
0.59	-1.429	Q91Z53	GRHPR	glyoxylate and hydroxypyruvate reductase	enzyme
0.62	2.2	Q7TS64	GRK2	G protein-coupled receptor kinase 2	kinase
0.38	-3.333	P28798	GRN	granulin precursor	growth factor
0.62	1.3	Q9D8T2	GSDMD	gasdermin D	other
0.042	-5	GELS_HUMAN	GSN	gelsolin	other
0.095	-2.5	P13020	GSN	gelsolin	other
0.34	1.8	Q8R050	GSPT1	G1 to S phase transition 1	translation regulator
0.34	3.6	Q99ME9	GTPBP4	GTP binding protein 4	enzyme
0.1	-1.667	P12265	GUSB	glucuronidase beta	enzyme
0.022	5.3	Q9Z1E4	GYS1	glycogen synthase 1	enzyme
0.57	-1.667	Q9QZQ8	H2AFY	H2A histone family member Y	other
0.82	1	P0C0S6	H2AFZ	H2A histone family member Z	other
0.78	1.7	Q8BY71	HAT1	histone acetyltransferase 1	enzyme
0.82	1	B1AUX2	HCFC1	host cell factor C1	transcription regulator
0.82	1	F6UND7	HCK	HCK proto-oncogene, Src family tyrosine kinase	kinase
0.099	2	O09106	HDAC1	histone deacetylase 1	transcription regulator
0.61	1.1	Q8VDJ3	HDLBP	high density lipoprotein binding protein	transporter
0.38	-1.667	G3X9B1	HEATR1	HEAT repeat containing 1	other
0.051	4.9	A2AS03	HELZ2	helicase with zinc finger 2	transcription regulator

Table S1

0.54	1.2	P29416	HEXA	hexosaminidase subunit alpha	enzyme
0.53	-1.667	B1ATZ0	HGS	hepatocyte growth factor-regulated tyrosine kinase substrate	other
0.33	-1.429	P70349	HINT1	histidine triad nucleotide binding protein 1	enzyme
0.022	8.9	P43275	Hist1h1a	histone cluster 1, H1a	other
0.01	5.8	P43276	Hist1h1b	histone cluster 1, H1b	other
0.023	2.3	P15864	HIST1H1C	histone cluster 1 H1 family member c	other
0.62	1.4	P43277	HIST1H1D	histone cluster 1 H1 family member d	other
0.17	2.7	P43274	Hist1h1e	histone cluster 1, H1e	other
0.049	2	Q8CGP5	HIST1H2AJ	histone cluster 1 H2A family member j	other
0.82	1	A0A1W2P768	HIST1H3C	histone cluster 1 H3 family member c	other
0.56	1.2	E9Q3Z4	HK3	hexokinase 3	kinase
0.35	-2	P01901	HLA-A	major histocompatibility complex, class I, A	other
0.15	-1.667	P01897	HLA-A	major histocompatibility complex, class I, A	other
0.11	-1.429	P01902	HLA-A	major histocompatibility complex, class I, A	other
0.33	-1.111	P01900	HLA-A	major histocompatibility complex, class I, A	other
0.47	1.7	P22907	HMBS	hydroxymethylbilane synthase	enzyme
0.26	1.4	P52927	Hmga2	high mobility group AT-hook 2	enzyme
0.47	1.9	Q8JZK9	HMGCS1	3-hydroxy-3-methylglutaryl-CoA synthase 1	enzyme
0.24	4.4	Q9CX86	HNRNPA0	heterogeneous nuclear ribonucleoprotein A0	other
0.36	-1.429	Q5EBP8	Hnrnpa1	heterogeneous nuclear ribonucleoprotein A1	other
0.076	-5	O88569	HNRNPA2B1	heterogeneous nuclear ribonucleoprotein A2/B1	other
0.16	2.2	Q8BG05	Hnrnpa3	heterogeneous nuclear ribonucleoprotein A3	transporter
0.44	-1.25	Q99020	HNRNPAB	heterogeneous nuclear ribonucleoprotein A/B	enzyme
0.099	1.8	Q9Z204	HNRNPC	heterogeneous nuclear ribonucleoprotein C (C1/C2)	other
0.24	4.3	Q60668	HNRNPD	heterogeneous nuclear ribonucleoprotein D	transcription regulator
0.091	1.9	Q9Z2X1	HNRNPF	heterogeneous nuclear ribonucleoprotein F	other
0.034	1.8	Q8C2Q7	HNRNPH1	heterogeneous nuclear ribonucleoprotein H1	other
0.26	1.4	P61979	HNRNPK	heterogeneous nuclear ribonucleoprotein K	transcription regulator
0.0023	4.9	G5E924	HNRNPL	heterogeneous nuclear ribonucleoprotein L	other
0.47	3.2	Q921F4	HNRNPLL	heterogeneous nuclear ribonucleoprotein L like	other

Table S1

0.16	1.5	Q9D0E1	HNRNPM	heterogeneous nuclear ribonucleoprotein M	other
0.12	3.1	Q8VHM5	HNRNPR	heterogeneous nuclear ribonucleoprotein R	other
0.13	1.5	Q8VEK3	HNRNPU	heterogeneous nuclear ribonucleoprotein U	transporter
0.34	2	Q8VDM6	HNRNPUL1	heterogeneous nuclear ribonucleoprotein U like 1	other
0.47	3	Q00PI9	HNRNPUL2	heterogeneous nuclear ribonucleoprotein U like 2	other
0.16	2.6	Z4YKB8	HP1BP3	heterochromatin protein 1 binding protein 3	other
0.38	-1.429	P00493	HPRT1	hypoxanthine phosphoribosyltransferase 1	enzyme
0.82	1	P51660	HSD17B4	hydroxysteroid 17-beta dehydrogenase 4	enzyme
0.48	1	P07901	HSP90AA1	heat shock protein 90 alpha family class A member 1	enzyme
0.16	-1.111	P11499	HSP90AB1	heat shock protein 90 alpha family class B member 1	enzyme
0.44	-1.25	P08113	HSP90B1	heat shock protein 90 beta family member 1	other
0.087	-3.333	P17879	Hspa1b	heat shock protein 1B	other
0.21	-1.429	Q3U2G2	HSPA4	heat shock protein family A (Hsp70) member 4	other
0.82	1	P48722	HSPA4L	heat shock protein family A (Hsp70) member 4 like	other
0.00054	-2.5	P20029	HSPA5	heat shock protein family A (Hsp70) member 5	enzyme
0.0001	-2	P63017	HSPA8	heat shock protein family A (Hsp70) member 8	enzyme
0.62	-1.111	P38647	HSPA9	heat shock protein family A (Hsp70) member 9	other
0.78	1.8	P63038	HSPD1	heat shock protein family D (Hsp60) member 1	enzyme
0.54	1	Q61699	HSPH1	heat shock protein family H (Hsp110) member 1	other
0.82	1	G3X9H5	HTT	huntingtin	transcription regulator
0.34	3.6	A2AFQ0	HUWE1	HECT, UBA and WWE domain containing 1, E3 ubiquitin protein ligase	transcription regulator
0.39	1.4	Q9JKR6	HYOU1	hypoxia up-regulated 1	other
0.0028	2.4	Q8BU30	IARS	isoleucyl-tRNA synthetase	enzyme
0.0026	-5	P13597	ICAM1	intercellular adhesion molecule 1	transmembrane receptor
0.59	-2	F6RPJ9	IDE	insulin degrading enzyme	peptidase
0.17	1.5	O88844	IDH1	isocitrate dehydrogenase (NADP(+)) 1, cytosolic	enzyme
0.47	2.9	P0DOV2	IFI16	interferon gamma inducible protein 16	transcription regulator
0.78	1.8	Q9R002	Ifi202b	interferon activated gene 202B	other
0.64	1.1	Q8BV66	IFI44	interferon induced protein 44	other

Table S1

0.033	1.9	Q9BDB7	IFI44L	interferon induced protein 44 like	other
0.64	1.7	Q61635	Ifi47	interferon gamma inducible protein 47	other
0.61	1.3	Q8R5F7	IFIH1	interferon induced with helicase C domain 1	enzyme
0.12	5.8	Q64282	IFIT1B	interferon induced protein with tetratricopeptide repeats 1B	other
0.24	-2	Q99J93	IFITM2	interferon induced transmembrane protein 2	other
0.25	-1.25	Q9CQW9	IFITM3	interferon induced transmembrane protein 3	other
0.23	-2	P19182	IFRD1	interferon related developmental regulator 1	other
0.39	1.5	Q07113	IGF2R	insulin like growth factor 2 receptor	transmembrane receptor
0.0001	-2.5	Q8R366	IGSF8	immunoglobulin superfamily member 8	other
0.59	1.4	A0A0R4J0T4	IKBKB	inhibitor of nuclear factor kappa B kinase subunit beta	kinase
0.59	1.4	Q60943	IL17RA	interleukin 17 receptor A	transmembrane receptor
0.82	1	P16382	IL4R	interleukin 4 receptor	transmembrane receptor
0.32	-1.667	Q00560	IL6ST	interleukin 6 signal transducer	transmembrane receptor
0.24	2.3	Q9CXY6	ILF2	interleukin enhancer binding factor 2	transcription regulator
0.07	2.4	O55222	ILK	integrin linked kinase	kinase
0.34	2	P24547	IMPDH2	inosine monophosphate dehydrogenase 2	enzyme
0.59	1.4	Q0GNC1	INF2	inverted formin, FH2 and WH2 domain containing	other
0.22	2.5	Q9ES52	INPP5D	inositol polyphosphate-5-phosphatase D	phosphatase
0.82	1	A0A087WPT7	INPPL1	inositol polyphosphate phosphatase like 1	phosphatase
0.62	2.5	K3W4P2	INTS1	integrator complex subunit 1	other
0.24	2.3	A0A0G2JFJ6	INTS3	integrator complex subunit 3	other
0.34	2.2	Q8CIM8	INTS4	integrator complex subunit 4	other
0.53	1.1	Q8CHT3	INTS5	integrator complex subunit 5	other
0.82	1	A0A0R4J0E4	INTS7	integrator complex subunit 7	other
0.62	2.1	Q8K2V6	IPO11	importin 11	transporter
0.82	1	Q8K0C1	IPO13	importin 13	transporter
0.052	3.5	Q8VI75	IPO4	importin 4	transporter
0.48	-1.111	Q8BKC5	IPO5	importin 5	transporter
0.0068	4.3	Q9EPL8	IPO7	importin 7	transporter
0.18	-1.25	Q9JKF1	IQGAP1	IQ motif containing GTPase activating protein 1	other

Table S1

0.0061	-10	A0A140LIF8	IRGM	immunity related GTPase M	enzyme
0.51	1.2	J7NUP1	Irgm1	immunity-related GTPase family M member 1	other
0.17	3.4	Q64339	ISG15	ISG15 ubiquitin-like modifier	other
0.24	-2.5	Q9CX00	IST1	IST1, ESCRT-III associated factor	other
0.36	-2.5	Q9JHU9	ISYNA1	inositol-3-phosphate synthase 1	enzyme
0.46	-1.25	Q8C863	ITCH	itchy E3 ubiquitin protein ligase	enzyme
0.0001	-5	Q792F9	ITGA4	integrin subunit alpha 4	transmembrane receptor
0.78	1.7	P11688	ITGA5	integrin subunit alpha 5	transmembrane receptor
0.00055	-2.5	E9Q604	ITGAM	integrin subunit alpha M	transmembrane receptor
0.38	-2.5	P43406	ITGAV	integrin subunit alpha V	transmembrane receptor
0.0001	-3.333	P09055	ITGB1	integrin subunit beta 1	transmembrane receptor
0.0001	-3.333	P11835	ITGB2	integrin subunit beta 2	transmembrane receptor
0.38	-2.5	P26011	ITGB7	integrin subunit beta 7	transmembrane receptor
0.14	-3.333	G3X977	ITIH2	inter-alpha-trypsin inhibitor heavy chain 2	other
0.53	-1.111	A0A087WRM2	ITM2C	integral membrane protein 2C	other
0.15	-2	B1ASP2	JAK1	Janus kinase 1	kinase
0.82	1	Q99MN1	KARS	lysyl-tRNA synthetase	enzyme
0.62	2.5	Q8BNW9	KBTBD11	kelch repeat and BTB domain containing 11	other
0.82	1	P35561	KCNJ2	potassium voltage-gated channel subfamily J member 2	ion channel
0.34	1.6	O89109	KCNN4	potassium calcium-activated channel subfamily N member 4	ion channel
0.59	1.4	Q6ZQ88	KDM1A	lysine demethylase 1A	enzyme
0.78	1.7	A0A1L1SS10	KEAP1	kelch like ECH associated protein 1	transcription regulator
0.063	-2.5	Q3U0V1	KHSRP	KH-type splicing regulatory protein	enzyme
0.019	-2	A0A1Y7VME9	KIDINS220	kinase D interacting substrate 220	transcription regulator
0.62	2.4	Q6P9L6	KIF15	kinesin family member 15	other
0.51	-1.111	Q61768	KIF5B	kinesin family member 5B	other
0.47	2.9	Q6PAR0	KLHDC10	kelch domain containing 10	other
0.36	1.2	P52293	KPNA2	karyopherin subunit alpha 2	transporter
0.59	1.4	O35345	KPNA6	karyopherin subunit alpha 6	transporter
0.17	1.3	P70168	KPNB1	karyopherin subunit beta 1	transporter

Table S1

0.62	2.5	G3UZA0	KRR1	KRR1, small subunit processome component homolog	other
0.0001	-2.5	K2C1_HUMAN	KRT1	keratin 1	other
0.0012	-2.5	K1C10_HUMAN	KRT10	keratin 10	other
0.38	-2.5	Q9Z2K1	KRT16	keratin 16	other
0.029	-3.333	Q9QWL7	KRT17	keratin 17	other
0.027	-1.667	K22E_HUMAN	KRT2	keratin 2	other
0.042	-5	P50446	KRT6B	keratin 6B	other
0.0001	-2.5	K1C9_HUMAN	KRT9	keratin 9	other
0.64	-1.25	P11438	LAMP1	lysosomal associated membrane protein 1	other
0.24	-2	P17047	LAMP2	lysosomal associated membrane protein 2	enzyme
0.38	-1.667	Q9CQ22	LAMTOR1	late endosomal/lysosomal adaptor, MAPK and MTOR activator 1	other
0.59	-1.429	F6RJV6	LANCL2	LanC like 2	other
0.38	-2.5	Q9CPY7	LAP3	leucine aminopeptidase 3	peptidase
0.14	-5	F8WH95	LAPTM5	lysosomal protein transmembrane 5	other
0.34	3.8	Z4YJT3	LARP1	La ribonucleoprotein domain family member 1	translation regulator
0.34	3.8	Q05CL8	LARP7	La ribonucleoprotein domain family member 7	other
0.00032	2.6	Q8BMJ2	LARS	leucyl-tRNA synthetase	enzyme
0.23	-5	Q61792	LASP1	LIM and SH3 protein 1	transporter
0.59	-1.111	Q8BYR1	LCMT2	leucine carboxyl methyltransferase 2	enzyme
0.00034	-2.5	Q61233	LCP1	lymphocyte cytosolic protein 1	other
0.47	3.3	Q60787	LCP2	lymphocyte cytosolic protein 2	other
0.029	-1.667	P06151	LDHA	lactate dehydrogenase A	enzyme
0.56	1	P35951	LDLR	low density lipoprotein receptor	transporter
0.62	2	A0A087WQH1	Lemd1	LEM domain containing 1	other
0.59	-2.5	P16045	LGALS1	galectin 1	other
0.063	-2.5	P16110	LGALS3	galectin 3	other
0.0057	1.5	Q07797	LGALS3BP	galectin 3 binding protein	transmembrane receptor
0.59	-2	Q9JL15	LGALS8	galectin 8	other
0.36	-2.5	B1AQR8	LGALS9B	galectin 9B	other
0.033	8	Q3U4X8	LIG1	DNA ligase 1	enzyme
0.19	-1.429	Q64281	LILRB4	leukocyte immunoglobulin like receptor B4	other
0.62	1.2	Q8K0B2	LMBRD1	LMBR1 domain containing 1	enzyme
0.045	-2.5	P48678	LMNA	lamin A/C	other
0.15	-2.5	Q8C129	LNPEP	leucyl and cystinyl aminopeptidase	peptidase

Table S1

0.82	1	Q8BYI6	LPCAT2	lysophosphatidylcholine acyltransferase 2	enzyme
0.0018	2	P11152	LPL	lipoprotein lipase	enzyme
0.59	-2	Q99N69	LPXN	leupaxin	transcription regulator
0.62	1.2	E9Q3Y4	LRBA	LPS responsive beige-like anchor protein	other
0.00033	-5	Q91ZX7	LRP1	LDL receptor related protein 1	transmembrane receptor
0.38	-1.667	Q7TQH7	LRP10	LDL receptor related protein 10	transmembrane receptor
0.15	-2.5	Q8BUJ9	LRP12	LDL receptor related protein 12	transmembrane receptor
0.64	1.3	A2API5	LRP1B	LDL receptor related protein 1B	transmembrane receptor
0.82	1	A0A0R4J0W6	LRRC40	leucine rich repeat containing 40	other
0.82	1	Q8R502	LRRC8C	leucine rich repeat containing 8 VRAC subunit C	ion channel
0.82	1	Q8BLN5	LSS	lanosterol synthase	enzyme
0.51	1.1	Q05CX5	LUC7L2	LUC7 like 2, pre-mRNA splicing factor	other
0.59	1.4	Q8R4U7	LUZP1	leucine zipper protein 1	other
0.014	6.3	Q08288	LYAR	Ly1 antibody reactive	other
0.0014	-2	P25911	LYN	LYN proto-oncogene, Src family tyrosine kinase	kinase
0.32	-1.25	P08905	LYZ	lysozyme	enzyme
0.072	-2	P24668	M6PR	mannose-6-phosphate receptor, cation dependent	transporter
0.82	1	E9PZ88	MAN2C1	mannosidase alpha class 2C member 1	enzyme
0.16	2.1	Q8C052	MAP1S	microtubule associated protein 1S	enzyme
0.3	2	P31938	MAP2K1	mitogen-activated protein kinase kinase 1	kinase
0.34	3.6	O09110	MAP2K3	mitogen-activated protein kinase kinase 3	kinase
0.042	-2.5	B7ZNR9	MAP4K4	mitogen-activated protein kinase kinase kinase kinase 4	kinase
0.53	-1.111	P63085	MAPK1	mitogen-activated protein kinase 1	kinase
0.26	1.8	Q63844	MAPK3	mitogen-activated protein kinase 3	kinase
0.78	1.8	P49138	MAPKAPK2	mitogen-activated protein kinase-activated protein kinase 2	kinase
0.49	-1.429	P28667	MARCKSL1	MARCKS like 1	other
0.03	1.6	E9QB02	MARS	methionyl-tRNA synthetase	enzyme
0.14	2.1	Q3THS6	MAT2A	methionine adenosyltransferase 2A	enzyme
0.39	2.2	Q99LB6	MAT2B	methionine adenosyltransferase 2B	enzyme
0.0001	12	Q8K310	MATR3	matrin 3	other

Table S1

0.0001	3.6	P97310	MCM2	minichromosome maintenance complex component 2	enzyme
0.0001	4.7	P25206	MCM3	minichromosome maintenance complex component 3	enzyme
0.0001	8.3	P49717	MCM4	minichromosome maintenance complex component 4	enzyme
0.018	2.6	Q52KC3	MCM5	minichromosome maintenance complex component 5	enzyme
0.023	2.1	P97311	MCM6	minichromosome maintenance complex component 6	enzyme
0.0015	2.3	Q61881	MCM7	minichromosome maintenance complex component 7	enzyme
0.47	1.9	Q8R3C0	MCMBP	minichromosome maintenance complex binding protein	other
0.23	-2.5	A0A087WRH9	MDFIC	MyoD family inhibitor domain containing	other
0.042	-5	P14152	MDH1	malate dehydrogenase 1	enzyme
0.62	2	P08249	MDH2	malate dehydrogenase 2	enzyme
0.43	1.8	A2ANY6	MDN1	midasin AAA ATPase 1	other
0.24	4.3	Q91VH6	MEMO1	mediator of cell motility 1	other
0.62	2.2	Q8K3A9	MEPCE	methylphosphate capping enzyme	enzyme
0.34	3.6	Q8BP48	METAP1	methionyl aminopeptidase 1	peptidase
0.47	2.7	Q91YR5	METTL13	methyltransferase like 13	enzyme
0.59	1.4	A0A0R4J1C7	MFAP3	microfibril associated protein 3	other
0.0001	-3.333	P21956	MFGE8	milk fat globule-EGF factor 8 protein	other
0.62	1.5	B5THE2	MGAM	maltase-glucoamylase	enzyme
0.36	-1.667	Q9D074	MGRN1	mahogunin ring finger 1	enzyme
0.62	-1.111	H3BKH2	Mia2	melanoma inhibitory activity 2	other
0.59	1.4	P34884	MIF	macrophage migration inhibitory factor	cytokine
0.0061	-10	Q9JM52	MINK1	misshapen like kinase 1	kinase
0.62	2.2	Q9D2Y4	MLKL	mixed lineage kinase domain like pseudokinase	kinase
0.22	2.9	Q9D071	MMS19	MMS19 homolog, cytosolic iron-sulfur assembly component	transcription regulator
0.0032	-3.333	D3YVL0	MOV10	Mov10 RISC complex RNA helicase	enzyme
0.049	-2	E9QN37	MPEG1	macrophage expressed 1	other
0.59	-1.429	B7ZCL8	MPP1	membrane palmitoylated protein 1	kinase
0.47	1.6	Q9CQT1	MRI1	methylthioribose-1-phosphate isomerase 1	translation regulator
0.64	1.4	E0CZ22	MROH1	maestro heat like repeat family member 1	other
0.47	2.9	P54276	MSH6	mutS homolog 6	enzyme

Table S1

0.0001	-5	P26041	MSN	moesin	other
0.38	-3.333	P30204	MSR1	macrophage scavenger receptor 1	transmembrane receptor
0.47	1.2	E9PUB7	MSTO1	misato 1, mitochondrial distribution and morphology regulator	other
0.0038	4.6	Q9R190	MTA2	metastasis associated 1 family member 2	transcription regulator
0.47	3	Q9CQ65	MTAP	methylthioadenosine phosphorylase	enzyme
0.0016	2.5	Q922D8	MTHFD1	methylenetetrahydrofolate dehydrogenase, cyclohydrolase and formyltetrahydrofolate synthetase 1	enzyme
0.17	3.5	Q9JLN9	MTOR	mechanistic target of rapamycin kinase	kinase
0.014	9.4	Q9CZU3	MTREX	Mtr4 exosome RNA helicase	other
0.07	-2	Q78HU3	MVB12A	multivesicular body subunit 12A	other
0.82	1	Q99JF5	MVD	mevalonate diphosphate decarboxylase	enzyme
0.00024	-3.333	E9Q3X0	MVP	major vault protein	other
0.4	-1.25	O35682	MYADM	myeloid associated differentiation marker	other
0.0001	2.7	Q7TPV4	MYBBP1A	MYB binding protein 1a	transcription regulator
0.62	2.6	F6SMY7	MYCBP2	MYC binding protein 2, E3 ubiquitin protein ligase	enzyme
0.59	1.4	P22366	MYD88	myeloid differentiation primary response 88	other
0.59	-2	Q6URW6	MYH14	myosin heavy chain 14	enzyme
0.024	1.4	Q8VDD5	MYH9	myosin heavy chain 9	enzyme
0.35	-1.25	Q60605	MYL6	myosin light chain 6	enzyme
0.82	1	B1B1A8	MYLK	myosin light chain kinase	kinase
0.17	3.1	Q9JMH9	MYO18A	myosin XVIIIA	other
0.00018	-3.333	Q9WTI7	MYO1C	myosin IC	enzyme
0.23	-3.333	Q5SYD0	MYO1D	myosin ID	enzyme
0.26	2.3	E9Q634	MYO1E	myosin IE	enzyme
0.074	-2	Q5SUA5	MYO1G	myosin IG	other
0.035	-2	Q69ZN7	MYOF	myoferlin	other
0.12	3.1	Q3V4D5	NAA10	N(alpha)-acetyltransferase 10, NatA catalytic subunit	enzyme
0.018	3.1	G3X8Y3	NAA15	N(alpha)-acetyltransferase 15, NatA auxiliary subunit	transcription regulator
0.62	2	Q8VE10	NAA40	N(alpha)-acetyltransferase 40, NatD catalytic subunit	other
0.3	2.1	Q6PGB6	NAA50	N(alpha)-acetyltransferase 50, NatE catalytic subunit	enzyme
0.61	-1.111	O88325	NAGLU	N-acetyl-alpha-glucosaminidase	enzyme

Table S1

0.49	1.1	Q99J77	NANS	N-acetylneuraminate synthase	enzyme
0.53	-1.667	E9PW66	NAP1L1	nucleosome assembly protein 1 like 1	other
0.23	-2	B7ZNL2	NAP1L4	nucleosome assembly protein 1 like 4	other
0.14	-5	Q9DB05	NAPA	NSF attachment protein alpha	transporter
0.23	-3.333	D3Z4B2	NAPG	NSF attachment protein gamma	transporter
0.15	-1.667	O09043	NAPSA	napsin A aspartic peptidase	peptidase
0.16	1.5	Q8BP47	NARS	asparaginyl-tRNA synthetase	enzyme
0.24	4.4	Q8K224	NAT10	N-acetyltransferase 10	enzyme
0.64	1	P97432	NBR1	NBR1, autophagy cargo receptor	other
0.47	2.9	Q8K2Z4	NCAPD2	non-SMC condensin I complex subunit D2	other
0.34	3.6	Q3UYV9	NCBP1	nuclear cap binding protein subunit 1	other
0.43	1.6	S4R293	NCF1	neutrophil cytosolic factor 1	enzyme
0.47	1.7	O70145	NCF2	neutrophil cytosolic factor 2	enzyme
0.62	1.2	P97369	NCF4	neutrophil cytosolic factor 4	enzyme
0.38	1.2	Q8K1X4	NCKAP1L	NCK associated protein 1 like	other
0.0086	1.8	P09405	NCL	nucleolin	other
0.15	-1.667	P57716	NCSTN	nicastrin	peptidase
0.49	-1.25	Q8R0W6	NDFIP1	Nedd4 family interacting protein 1	other
0.36	-1.25	M0QWK1	NDFIP2	Nedd4 family interacting protein 2	other
0.075	-1.667	Q62433	NDRG1	N-myc downstream regulated 1	kinase
0.62	2.5	D3YUM1	NDUFV1	NADH:ubiquinone oxidoreductase core subunit V1	enzyme
0.59	1.4	P29595	NEDD8	neural precursor cell expressed, developmentally down-regulated 8	enzyme
0.47	3.1	Q8K1R7	NEK9	NIMA related kinase 9	kinase
0.77	1	Q9WTK5	NFKB2	nuclear factor kappa B subunit 2	transcription regulator
0.47	3.2	Q9CRB2	NHP2	NHP2 ribonucleoprotein	other
0.82	1	A0A0R4J0G3	NIPAL2	NIPA like domain containing 2	other
0.59	1.4	Q8VEJ4	NLE1	notchless homolog 1	enzyme
0.82	1	Q8R4B8	NLRP3	NLR family pyrin domain containing 3	other
0.025	2.7	O70310	NMT1	N-myristoyltransferase 1	enzyme
0.34	4	Q8BW10	NOB1	NIN1 (RPN12) binding protein 1 homolog	enzyme
0.62	2.4	Q8BHY2	NOC4L	nucleolar complex associated 4 homolog	transcription regulator

Table S1

0.05	2.3	Q99K48	NONO	non-POU domain containing octamer binding	transcription regulator
0.82	1	Q9CQS2	NOP10	NOP10 ribonucleoprotein	other
0.78	1.7	Q8R3N1	NOP14	NOP14 nucleolar protein	other
0.052	3.6	E9QN31	NOP2	NOP2 nucleolar protein	other
0.56	1	Q9D6Z1	NOP56	NOP56 ribonucleoprotein	other
0.066	2.7	Q6DFW4	NOP58	NOP58 ribonucleoprotein	enzyme
0.47	2.9	Q8BMC4	NOP9	NOP9 nucleolar protein	other
0.82	1	P29477	NOS2	nitric oxide synthase 2	enzyme
0.095	-2.5	Q6WKZ7	NOSTRIN	nitric oxide synthase trafficking	transcription regulator
0.38	-2.5	G5E8J0	NOTCH2	notch 2	transcription regulator
0.53	-1.25	Q9QZQ0	NPAS3	neuronal PAS domain protein 3	transcription regulator
0.38	-3.333	O35604	NPC1	NPC intracellular cholesterol transporter 1	transporter
0.59	1.4	Q11011	NPEPPS	aminopeptidase puromycin sensitive	peptidase
0.47	1.1	Q61937	NPM1	nucleophosmin 1	transcription regulator
0.36	-1.667	P97300	Nptn	neuroplastin	other
0.14	-5	Q8CJ26	Nradd	neurotrophin receptor associated death domain	cytokine
0.0026	-3.333	A0A0G2JGP4	NRAS	NRAS proto-oncogene, GTPase	enzyme
0.033	-2.5	P97333	NRP1	neuropilin 1	transmembrane receptor
0.051	-2	O35375	NRP2	neuropilin 2	kinase
0.82	1	Q6P9K9	Nrxn3	neurexin III	other
0.49	1.1	P46460	NSF	N-ethylmaleimide sensitive factor, vesicle fusing ATPase	transporter
0.23	-3.333	Q9CZ44	NSFL1C	NSFL1 cofactor	other
0.0011	4.1	H3BKN0	NSUN2	NOP2/Sun RNA methyltransferase family member 2	enzyme
0.62	2.5	Q9JM14	NT5C	5', 3'-nucleotidase, cytosolic	phosphatase
0.59	1.4	Q8C5P5	NT5DC1	5'-nucleotidase domain containing 1	other
0.38	-2.5	A0A0A6YWF9	NTRK3	neurotrophic receptor tyrosine kinase 3	kinase
0.47	3.3	A0A0G2JGQ4	NUB1	negative regulator of ubiquitin like proteins 1	other
0.38	-3.333	O35685	NUDC	nuclear distribution C, dynein complex regulator	other
0.39	1.9	Q9CQ48	NUDCD2	NudC domain containing 2	other
0.82	1	Q9CQF3	NUDT21	nudix hydrolase 21	other
0.62	1.5	Q9JKX6	NUDT5	nudix hydrolase 5	phosphatase
0.47	3	E9Q7G0	NUMA1	nuclear mitotic apparatus protein 1	other
0.014	9.5	Q6ZQH8	NUP188	nucleoporin 188	other
0.47	3	A0A0J9YUD5	NUP205	nucleoporin 205	other
0.82	1	Q8R480	NUP85	nucleoporin 85	other

Table S1

0.62	2.6	Q8BJ71	NUP93	nucleoporin 93	other
0.59	-1.429	Q99LJ8	NUS1	NUS1, dehydrodolichyl diphosphate synthase subunit	enzyme
0.82	1	P61971	NUTF2	nuclear transport factor 2	transporter
0.82	1	Q99JX7	NXF1	nuclear RNA export factor 1	other
0.11	-2	P11928	OAS1	2'-5'-oligoadenylate synthetase 1	enzyme
0.53	-1.25	E9Q9A9	OAS2	2'-5'-oligoadenylate synthetase 2	enzyme
0.0001	5	Q8VI93	OAS3	2'-5'-oligoadenylate synthetase 3	enzyme
0.3	1.5	Q8VI94	OASL	2'-5'-oligoadenylate synthetase like	enzyme
0.24	4.2	P29758	OAT	ornithine aminotransferase	enzyme
0.24	4.3	P00860	ODC1	ornithine decarboxylase 1	enzyme
0.51	1.2	Q9EQQ9	OGA	O-GlcNAcase	enzyme
0.34	3.7	Q99PG2	OGFR	opioid growth factor receptor	other
0.62	2.5	Q8CGY8	OGT	O-linked N-acetylglucosamine (GlcNAc) transferase	enzyme
0.22	2.9	Q9CZ30	OLA1	Obg like ATPase 1	enzyme
0.82	1	H7BX01	OPA1	OPA1, mitochondrial dynamin like GTPase	enzyme
0.14	-3.333	B9EJ86	OSBPL8	oxysterol binding protein like 8	transporter
0.62	2.5	Q8BWU5	OSGEP	O-sialoglycoprotein endopeptidase	peptidase
0.53	-1.25	Q62422	OSTF1	osteoclast stimulating factor 1	transcription regulator
0.59	1	D3YWF6	Otub1	OTU domain, ubiquitin aldehyde binding 1	enzyme
0.24	4.3	Q3UJQ9	OXCT1	3-oxoacid CoA-transferase 1	enzyme
0.35	-2	Q6P9R2	OXSR1	oxidative stress responsive 1	kinase
0.24	-2.5	P09103	P4HB	prolyl 4-hydroxylase subunit beta	enzyme
0.49	1.2	Q3TGU7	PA2G4	proliferation-associated 2G4	transcription regulator
0.0081	-1.429	P29341	PABPC1	poly(A) binding protein cytoplasmic 1	translation regulator
0.33	-1.667	Q6PHQ9	PABPC4	poly(A) binding protein cytoplasmic 4	translation regulator
0.23	-3.333	Q9WVE8	PACSIN2	protein kinase C and casein kinase substrate in neurons 2	transporter
0.82	1	P63005	PAFAH1B1	platelet activating factor acetylhydrolase 1b regulatory subunit 1	enzyme

Table S1

0.21	1.8	Q9DCL9	PAICS	phosphoribosylaminoimidazole carboxylase and phosphoribosylaminoimidazolesuccinocarboxamide synthase	enzyme
0.59	1.4	Q9DCE5	PAK1IP1	PAK1 interacting protein 1	other
0.59	-1.111	Q8CIN4	PAK2	p21 (RAC1) activated kinase 2	kinase
0.23	-5	Q61036	PAK3	p21 (RAC1) activated kinase 3	kinase
0.82	1	Q3U4S0	PANK2	pantothenate kinase 2	kinase
0.24	4.7	Q921K2	PARP1	poly(ADP-ribose) polymerase 1	enzyme
0.15	-2	Q8BZ20	PARP12	poly(ADP-ribose) polymerase family member 12	other
0.62	2.4	Q2EMV9	PARP14	poly(ADP-ribose) polymerase family member 14	enzyme
0.62	2.5	Q8CAS9	PARP9	poly(ADP-ribose) polymerase family member 9	enzyme
0.24	4.4	Q3TC46	PATL1	PAT1 homolog 1, processing body mRNA decay factor	translation regulator
0.3	-1.111	P60335	PCBP1	poly(rC) binding protein 1	translation regulator
0.23	-1.667	Q61990	PCBP2	poly(rC) binding protein 2	other
0.36	-1.429	A2RS43	PCDH7	protocadherin 7	other
0.12	5.9	Q8BH04	PCK2	phosphoenolpyruvate carboxykinase 2, mitochondrial	kinase
0.38	-2.5	F7D432	Pcmt1	protein-L-isoaspartate (D-aspartate) O-methyltransferase 1	enzyme
0.39	-1.25	P17918	PCNA	proliferating cell nuclear antigen	enzyme
0.59	1.4	Q922E4	PCYT2	phosphate cytidylyltransferase 2, ethanolamine	enzyme
0.4	1	P12815	PDCD6	programmed cell death 6	other
0.0001	-2	Q9WU78	PDCD6IP	programmed cell death 6 interacting protein	other
0.82	1	Q3TIU4	PDE12	phosphodiesterase 12	enzyme
0.012	-5	P27773	PDIA3	protein disulfide isomerase family A member 3	peptidase
0.012	-5	Q922R8	PDIA6	protein disulfide isomerase family A member 6	enzyme
0.47	1.6	E9QPI5	PDS5A	PDS5 cohesin associated factor A	other
0.44	1	Q8K183	PDXK	pyridoxal kinase	kinase
0.023	-3.333	Q8BFY6	PEF1	penta-EF-hand domain containing 1	other
0.62	2.2	Q9DBD5	PELP1	proline, glutamate and leucine rich protein 1	other

Table S1

0.47	3.3	Q5SQ20	PES1	pescadillo ribosomal biogenesis factor 1	other
0.0031	2.6	Q5SUR0	PFAS	phosphoribosylformylglycinamidine synthase	enzyme
0.0018	6.4	P12382	PFKL	phosphofructokinase, liver type	kinase
0.02	1.8	Q8C605	PFKP	phosphofructokinase, platelet	kinase
0.00035	-2.5	P62962	PFN1	profilin 1	other
0.0011	-3.333	Q9DBJ1	PGAM1	phosphoglycerate mutase 1	phosphatase
0.028	-2	Q9DCD0	PGD	phosphogluconate dehydrogenase	enzyme
0.37	1.1	P09411	PGK1	phosphoglycerate kinase 1	kinase
0.23	-3.333	Q9CQ60	PGLS	6-phosphogluconolactonase	enzyme
0.82	1	Q9D0F9	PGM1	phosphoglucomutase 1	enzyme
0.62	1.5	Q8BHF7	PGS1	phosphatidylglycerophosphate synthase 1	enzyme
0.26	-1.111	Q61753	PHGDH	phosphoglycerate dehydrogenase	enzyme
0.15	-2	Q2TBE6	PI4K2A	phosphatidylinositol 4-kinase type 2 alpha	kinase
0.61	1	Q8CBQ5	PI4K2B	phosphatidylinositol 4-kinase type 2 beta	kinase
0.51	1.8	A0A140T8I9	PI4KA	phosphatidylinositol 4-kinase alpha	kinase
0.59	-2	E2JF22	PIEZO1	piezo type mechanosensitive ion channel component 1	ion channel
0.82	1	Q8VD65	PIK3R4	phosphoinositide-3-kinase regulatory subunit 4	kinase
0.35	-2	O70172	PIP4K2A	phosphatidylinositol-5-phosphate 4-kinase type 2 alpha	kinase
0.24	-2.5	F8WHW3	PIP4P1	phosphatidylinositol-4,5-bisphosphate 4-phosphatase 1	phosphatase
0.82	1	Q9CZX7	PIP4P2	phosphatidylinositol-4,5-bisphosphate 4-phosphatase 2	phosphatase
0.72	-1.25	F8WI18	PIP5K1A	phosphatidylinositol-4-phosphate 5-kinase type 1 alpha	kinase
0.34	1.1	P52480	PKM	pyruvate kinase M1/2	kinase
0.78	1.7	P70268	PKN1	protein kinase N1	kinase
0.51	-1.111	Q9DBX5	PLA2G4A	phospholipase A2 group IVA	enzyme
0.82	1	P27612	PLAA	phospholipase A2 activating protein	other
0.15	-1.429	P06869	PLAU	plasminogen activator, urokinase	peptidase
0.62	1.3	P35456	PLAUR	plasminogen activator, urokinase receptor	transmembrane receptor

Table S1

0.022	6	Q8CIH5	PLCG2	phospholipase C gamma 2	enzyme
0.38	-3.333	D6RH77	PLD1	phospholipase D1	enzyme
0.62	1.1	O35405	PLD3	phospholipase D family member 3	enzyme
0.00039	3.3	Q9QXS1	PLEC	plectin	other
0.46	-1.25	Q9JHK5	PLEK	pleckstrin	transcription regulator
0.62	2.5	F8WIK5	PLEKHA2	pleckstrin homology domain containing A2	other
0.82	1	Q8VCE9	PLEKHH3	pleckstrin homology, MyTH4 and FERM domain containing H3	other
0.59	-2	A0A1W2P7X5	PLEKHN1	pleckstrin homology domain containing N1	other
0.051	-5	Q9R0E2	PLOD1	procollagen-lysine,2-oxoglutarate 5-dioxygenase 1	enzyme
0.39	1.9	Q9R0E1	PLOD3	procollagen-lysine,2-oxoglutarate 5-dioxygenase 3	enzyme
0.11	-2	Q9R1Q7	PLP2	proteolipid protein 2	transporter
0.14	-5	Q99K51	PLS3	plastin 3	other
0.076	-5	Q9JJ00	PLSCR1	phospholipid scramblase 1	enzyme
0.59	-1.111	Q9JIZ9	PLSCR3	phospholipid scramblase 3	enzyme
0.15	-3.333	P70206	PLXNA1	plexin A1	transmembrane receptor
0.0018	-2	B2RXS4	PLXNB2	plexin B2	transmembrane receptor
0.62	2.2	Q9DC61	PMPCA	peptidase, mitochondrial processing alpha subunit	peptidase
0.47	3.3	Q9JLV6	PNKP	polynucleotide kinase 3'-phosphatase	kinase
0.077	6.5	P52431	POLD1	DNA polymerase delta 1, catalytic subunit	enzyme
0.62	2.5	Q9WVF7	POLE	DNA polymerase epsilon, catalytic subunit	enzyme
0.78	1.8	P70700	POLR1B	RNA polymerase I subunit B	enzyme
0.033	7.8	P52432	POLR1C	RNA polymerase I and III subunit C	enzyme
0.006	3.1	A0A0R4J0V5	POLR2A	RNA polymerase II subunit A	enzyme
0.017	3.9	Q8CFI7	POLR2B	RNA polymerase II subunit B	enzyme
0.62	2.1	P97760	POLR2C	RNA polymerase II subunit C	enzyme
0.24	2.9	B2RXC6	POLR3A	RNA polymerase III subunit A	enzyme
0.59	-2.5	P37040	POR	cytochrome p450 oxidoreductase	enzyme
0.47	1.7	Q8CIH9	PPAT	phosphoribosyl pyrophosphate amidotransferase	enzyme
0.015	-1.667	P17742	PPIA	peptidylprolyl isomerase A	enzyme

Table S1

0.33	-1.667	P30412	PPIC	peptidylprolyl isomerase C	enzyme
0.59	1.4	A0A0J9YVG0	PPM1G	protein phosphatase, Mg2+/Mn2+ dependent 1G	phosphatase
0.017	2.9	P62137	PPP1CA	protein phosphatase 1 catalytic subunit alpha	phosphatase
0.47	1.5	P62141	PPP1CB	protein phosphatase 1 catalytic subunit beta	phosphatase
0.3	2.5	P63087	Ppp1cc	protein phosphatase 1 catalytic subunit gamma	phosphatase
0.26	2.1	Q3UM45	PPP1R7	protein phosphatase 1 regulatory subunit 7	phosphatase
0.12	2.4	P63330	PPP2CA	protein phosphatase 2 catalytic subunit alpha	phosphatase
0.28	1.4	Q76MZ3	PPP2R1A	protein phosphatase 2 scaffold subunit Aalpha	phosphatase
0.3	2.5	Q6P1F6	PPP2R2A	protein phosphatase 2 regulatory subunit Balpha	phosphatase
0.82	1	Q6PD03	PPP2R5A	protein phosphatase 2 regulatory subunit B'alpha	phosphatase
0.78	1.8	Q91V89	PPP2R5D	protein phosphatase 2 regulatory subunit B'delta	phosphatase
0.62	1.2	Q61151	PPP2R5E	protein phosphatase 2 regulatory subunit B'epsilon	phosphatase
0.82	1	P97470	PPP4C	protein phosphatase 4 catalytic subunit	phosphatase
0.82	1	Q60676	PPP5C	protein phosphatase 5 catalytic subunit	phosphatase
0.2	-1.429	P35700	PRDX1	peroxiredoxin 1	enzyme
0.057	-3.333	Q61171	PRDX2	peroxiredoxin 2	enzyme
0.23	-3.333	Q6GT24	PRDX6	peroxiredoxin 6	enzyme
0.36	1.2	Q9QUR6	PREP	prolyl endopeptidase	peptidase
0.62	2	J3QN19	PRIM1	DNA primase subunit 1	enzyme
0.47	3.2	P33610	PRIM2	DNA primase subunit 2	enzyme
0.72	-1.111	Q5EG47	PRKAA1	protein kinase AMP-activated catalytic subunit alpha 1	kinase
0.53	-1.25	P68181	PRKACB	protein kinase cAMP-activated catalytic subunit beta	kinase
0.82	1	O54950	PRKAG1	protein kinase AMP-activated non-catalytic subunit gamma 1	kinase
0.32	1.4	Q9DBC7	PRKAR1A	protein kinase cAMP-dependent type I regulatory subunit alpha	kinase
0.17	3.2	A0A0A6YX73	PRKAR2A	protein kinase cAMP-dependent type II regulatory subunit alpha	kinase
0.59	-2	P23298	PRKCH	protein kinase C eta	kinase
0.51	1.9	A0A171KXD3	PRMT1	protein arginine methyltransferase 1	enzyme
0.62	2.2	Q8CIG8	PRMT5	protein arginine methyltransferase 5	enzyme

Table S1

0.47	2.7	Q99KP6	PRPF19	pre-mRNA processing factor 19	enzyme
0.34	4	Q8CCF0	PRPF31	pre-mRNA processing factor 31	other
0.22	2	Q9DAW6	PRPF4	pre-mRNA processing factor 4	other
0.82	1	Q9R1C7	PRPF40A	pre-mRNA processing factor 40 homolog A	other
0.0001	5.3	Q99PV0	PRPF8	pre-mRNA processing factor 8	other
0.64	-1.111	G3UXL2	Prps1l3	phosphoribosyl pyrophosphate synthetase 1-like 3	kinase
0.53	-1.111	Q9D0M1	PRPSAP1	phosphoribosyl pyrophosphate synthetase associated protein 1	other
0.34	2.2	Q8R574	PRPSAP2	phosphoribosyl pyrophosphate synthetase associated protein 2	other
0.042	-10	J3QPG5	PSAP	prosaposin	enzyme
0.1	-2	Q99K85	PSAT1	phosphoserine aminotransferase 1	enzyme
0.82	1	P49769	PSEN1	presenilin 1	peptidase
0.44	1.1	Q9R1P4	PSMA1	proteasome subunit alpha 1	peptidase
0.01	-2	P49722	PSMA2	proteasome subunit alpha 2	peptidase
0.045	-2.5	O70435	PSMA3	proteasome subunit alpha 3	peptidase
0.35	-1.667	Q9R1P0	PSMA4	proteasome subunit alpha 4	peptidase
0.57	-1.25	Q9Z2U1	PSMA5	proteasome subunit alpha 5	peptidase
0.23	-1.667	Q9QUM9	PSMA6	proteasome subunit alpha 6	peptidase
0.0035	-5	Q9Z2U0	PSMA7	proteasome subunit alpha 7	peptidase
0.029	-5	O09061	PSMB1	proteasome subunit beta 1	peptidase
0.64	1.4	Q9R1P3	PSMB2	proteasome subunit beta 2	peptidase
0.32	-1.429	Q9R1P1	PSMB3	proteasome subunit beta 3	peptidase
0.31	-1.667	P99026	PSMB4	proteasome subunit beta 4	peptidase
0.23	-2.5	Q60692	PSMB6	proteasome subunit beta 6	peptidase
0.23	-3.333	P28063	PSMB8	proteasome subunit beta 8	peptidase
0.38	1.2	P62192	PSMC1	proteasome 26S subunit, ATPase 1	peptidase
0.014	1.7	Q8BVQ9	PSMC2	proteasome 26S subunit, ATPase 2	peptidase
0.013	2	O88685	PSMC3	proteasome 26S subunit, ATPase 3	enzyme
0.0028	2.6	P54775	PSMC4	proteasome 26S subunit, ATPase 4	peptidase

Table S1

0.32	1.2	P62196	PSMC5	proteasome 26S subunit, ATPase 5	transcription regulator
0.22	1.4	P62334	PSMC6	proteasome 26S subunit, ATPase 6	peptidase
0.46	1.1	Q3TXS7	PSMD1	proteasome 26S subunit, non-ATPase 1	other
0.23	1.5	Q8BG32	PSMD11	proteasome 26S subunit, non-ATPase 11	other
0.35	-1.25	Q9D8W5	PSMD12	proteasome 26S subunit, non-ATPase 12	other
0.77	1.1	Q9WVJ2	PSMD13	proteasome 26S subunit, non-ATPase 13	peptidase
0.11	1.9	O35593	PSMD14	proteasome 26S subunit, non-ATPase 14	peptidase
0.0059	2	Q8VDM4	PSMD2	proteasome 26S subunit, non-ATPase 2	other
0.32	1.2	P14685	PSMD3	proteasome 26S subunit, non-ATPase 3	other
0.49	-1.429	Q8BJY1	PSMD5	proteasome 26S subunit, non-ATPase 5	other
0.57	1	Q99JI4	PSMD6	proteasome 26S subunit, non-ATPase 6	enzyme
0.12	1.7	P26516	PSMD7	proteasome 26S subunit, non-ATPase 7	other
0.59	-1.429	Q9CX56	PSMD8	proteasome 26S subunit, non-ATPase 8	other
0.82	1	G3X9V0	PSME2	proteasome activator subunit 2	peptidase
0.59	1.4	P61290	PSME3	proteasome activator subunit 3	peptidase
0.82	1	Q5SSW2	PSME4	proteasome activator subunit 4	other
0.59	1.4	Q9JK23	PSMG1	proteasome assembly chaperone 1	other
0.23	-3.333	A0A0R4J0P5	PSTPIP1	proline-serine-threonine phosphatase interacting protein 1	other
0.43	1.7	Q8BGJ5	PTBP1	polypyrimidine tract binding protein 1	enzyme
0.47	3	Q8BXC0	PTGIS	prostaglandin I2 synthase	enzyme
0.38	-2.5	P22437	PTGS1	prostaglandin-endoperoxide synthase 1	enzyme
0.077	6.4	Q05769	PTGS2	prostaglandin-endoperoxide synthase 2	enzyme
0.78	1.8	E9Q2A6	PTK2B	protein tyrosine kinase 2 beta	kinase
0.72	-1.111	Q66GT5	PTPMT1	protein tyrosine phosphatase, mitochondrial 1	phosphatase
0.17	4.9	P35831	PTPN12	protein tyrosine phosphatase, non-receptor type 12	phosphatase
0.051	-3.333	Q6PB44	PTPN23	protein tyrosine phosphatase, non-receptor type 23	phosphatase
0.37	1.1	P29351	PTPN6	protein tyrosine phosphatase, non-receptor type 6	phosphatase

Table S1

0.015	-2	Q91V35	PTPRA	protein tyrosine phosphatase, receptor type A	phosphatase
0.0001	-3.333	S4R1M0	PTPRC	protein tyrosine phosphatase, receptor type C	phosphatase
0.59	1	A2AWF9	PTPRJ	protein tyrosine phosphatase, receptor type J	phosphatase
0.17	3.3	A0A0N4SUH4	PUM3	pumilio RNA binding family member 3	other
0.41	1.6	P42669	PURA	purine rich element binding protein A	transcription regulator
0.35	-1.667	O35295	PURB	purine rich element binding protein B	transcription regulator
0.24	2.3	Q91VU7	PUS7	pseudouridylate synthase 7	enzyme
0.59	1.4	Q8BU03	Pwp2	PWP2 periodic tryptophan protein homolog (yeast)	other
0.59	-2	Q8CI94	PYGB	glycogen phosphorylase B	enzyme
0.0001	4.9	D3Z158	Qars	glutaminyl-tRNA synthetase	enzyme
0.011	-3.333	P61027	RAB10	RAB10, member RAS oncogene family	enzyme
0.15	-2.5	F8WGS1	RAB11A	RAB11A, member RAS oncogene family	enzyme
0.14	-1.667	Q91V41	RAB14	RAB14, member RAS oncogene family	enzyme
0.14	-2.5	P35293	RAB18	RAB18, member RAS oncogene family	enzyme
0.11	-2	Q5SW88	RAB1A	RAB1A, member RAS oncogene family	enzyme
0.087	-3.333	Q9D1G1	RAB1B	RAB1B, member RAS oncogene family	other
0.0061	-10	P35282	RAB21	RAB21, member RAS oncogene family	enzyme
0.23	-2.5	P35285	RAB22A	RAB22A, member RAS oncogene family	enzyme
0.23	-5	P53994	RAB2A	RAB2A, member RAS oncogene family	enzyme
0.057	-3.333	Q3TXV4	RAB31	RAB31, member RAS oncogene family	enzyme
0.59	-2.5	Q6PHN9	RAB35	RAB35, member RAS oncogene family	enzyme
0.78	1.8	A0A1D5RLG3	RAB3GAP1	RAB3 GTPase activating protein catalytic subunit 1	other
0.07	-2.5	Q9CQD1	RAB5A	RAB5A, member RAS oncogene family	enzyme
0.14	-3.333	P61021	RAB5B	RAB5B, member RAS oncogene family	enzyme
0.0085	-2	P35278	RAB5C	RAB5C, member RAS oncogene family	enzyme
0.14	-5	P35279	RAB6A	RAB6A, member RAS oncogene family	enzyme
0.038	-1.667	P51150	RAB7A	RAB7A, member RAS oncogene family	enzyme
0.051	-3.333	P55258	RAB8A	RAB8A, member RAS oncogene family	enzyme

Table S1

0.0032	-3.333	P61028	RAB8B	RAB8B, member RAS oncogene family	enzyme
0.59	-1.429	Q9R0M6	RAB9A	RAB9A, member RAS oncogene family	enzyme
0.36	-2	Q3TLP8	RAC1	Rac family small GTPase 1	enzyme
0.075	-2	Q05144	RAC2	Rac family small GTPase 2	enzyme
0.0001	2.8	P68040	RACK1	receptor for activated C kinase 1	enzyme
0.38	-2.5	Q61550	RAD21	RAD21 cohesin complex component	transcription regulator
0.62	2.4	Q5SV02	RAD50	RAD50 double strand break repair protein	enzyme
0.14	-3.333	O08604	Raet1c	retinoic acid early transcript gamma	other
0.0047	-5	P63321	RALA	RAS like proto-oncogene A	enzyme
0.15	-2	Q9JIW9	RALB	RAS like proto-oncogene B	enzyme
0.47	3.3	Q64012	RALY	RALY heterogeneous nuclear ribonucleoprotein	transcription regulator
0.22	1.4	P62827	RAN	RAN, member RAS oncogene family	enzyme
0.23	-2.5	P34022	RANBP1	RAN binding protein 1	other
0.23	-2	A0A0R4J0G4	RANBP10	RAN binding protein 10	other
0.49	1	Q9CT10	RANBP3	RAN binding protein 3	other
0.64	1	P46061	RANGAP1	Ran GTPase activating protein 1	other
0.02	-1.667	Q99JI6	RAP1B	RAP1B, member of RAS oncogene family	enzyme
0.022	-5	P61226	RAP2B	RAP2B, member of RAS oncogene family	enzyme
0.0025	-5	Q8BU31	RAP2C	RAP2C, member of RAS oncogene family	enzyme
0.82	1	A0A0A6YWG7	RAPGEF2	Rap guanine nucleotide exchange factor 2	other
0.2	1.3	Q9D0I9	RARS	arginyl-tRNA synthetase	enzyme
0.26	2.3	E9PYG6	RASA1	RAS p21 protein activator 1	transporter
0.82	1	Q6PFQ7	RASA4	RAS p21 protein activator 4	other
0.62	2.4	Q60972	RBBP4	RB binding protein 4, chromatin remodeling factor	enzyme
0.34	3.6	A2AFJ1	RBBP7	RB binding protein 7, chromatin remodeling factor	transcription regulator
0.24	2.3	Q8VH51	RBM39	RNA binding motif protein 39	transcription regulator
0.34	3.7	P31266	RBPJ	recombination signal binding protein for immunoglobulin kappa J region	transcription regulator
0.033	8.1	Q6PFB2	RCC1	regulator of chromosome condensation 1	other
0.0041	2.9	Q8BK67	RCC2	regulator of chromosome condensation 2	other

Table S1

0.00078	-12.5	P26043	RDX	radixin	other
0.34	3.7	Q3UI84	RFC4	replication factor C subunit 4	other
0.38	-2.5	Q6A0D4	RFTN1	raftlin, lipid raft linker 1	other
0.59	1.4	Q9ERU9	RGPD4 (includes others)	RANBP2-like and GRIP domain containing 5	enzyme
0.82	1	Q80WQ6	RHBDF2	rhomboid 5 homolog 2	other
0.23	-2.5	Q9QUI0	RHOA	ras homolog family member A	enzyme
0.37	-1.111	Q62159	RHOC	ras homolog family member C	enzyme
0.33	-2	P84096	RHOG	ras homolog family member G	enzyme
0.78	1.8	Q9JJF3	RIOX1	ribosomal oxygenase 1	enzyme
0.47	2.9	Q9QZL0	RIPK3	receptor interacting serine/threonine kinase 3	kinase
0.14	-3.333	O54965	RNF13	ring finger protein 13	enzyme
0.15	-2.5	Q5SVR5	RNF130	ring finger protein 130	peptidase
0.21	-1.667	Q3U2C5	RNF149	ring finger protein 149	enzyme
0.0001	4.5	E9Q555	RNF213	ring finger protein 213	enzyme
0.087	-5	Q91VI7	RNH1	ribonuclease/angiogenin inhibitor 1	other
0.59	1.4	Q9D0L8	RNMT	RNA guanine-7 methyltransferase	enzyme
0.51	1	Q8VCT3	RNPEP	arginyl aminopeptidase	peptidase
0.24	4.4	Q5SWN2	RPA1	replication protein A1	other
0.43	1.2	G3X926	RPF2	ribosome production factor 2 homolog	other
0.62	2	A0A067XG46	RPGR	retinitis pigmentosa GTPase regulator	other
0.39	1.3	I7HLV2	RPL10	ribosomal protein L10	translation regulator
0.23	1.6	Q5XJF6	RPL10A	ribosomal protein L10a	other
0.44	-1.25	Q9CXW4	RPL11	ribosomal protein L11	other
0.39	-1.25	P35979	RPL12	ribosomal protein L12	other
0.52	1	P47963	RPL13	ribosomal protein L13	other
0.33	1.2	Q9CR57	RPL14	ribosomal protein L14	other
0.41	-1.429	Q9CPR4	RPL17	ribosomal protein L17	other
0.25	1.2	P35980	RPL18	ribosomal protein L18	other
0.45	-1.111	P62717	RPL18A	ribosomal protein L18a	other
0.23	1.3	P84099	RPL19	ribosomal protein L19	other
0.53	1	Q9CQM8	RPL21	ribosomal protein L21	other
0.17	3.3	P67984	RPL22	ribosomal protein L22	translation regulator
0.036	2.9	P62830	RPL23	ribosomal protein L23	other
0.023	2.5	Q8BP67	RPL24	ribosomal protein L24	other
0.16	1.9	P61255	RPL26	ribosomal protein L26	other
0.22	2.9	P61358	RPL27	ribosomal protein L27	other
0.22	-1.667	P14115	RPL27A	ribosomal protein L27a	other
0.39	1	P41105	RPL28	ribosomal protein L28	other
0.48	-1.25	P47915	Rpl29 (includes others)	ribosomal protein L29	other
0.28	1.1	P27659	RPL3	ribosomal protein L3	other

Table S1

0.49	1	P62889	RPL30	ribosomal protein L30	other
0.57	1	P62900	RPL31	ribosomal protein L31	other
0.46	-1.429	P62911	Rpl32	ribosomal protein L32	other
0.17	3.5	Q9D1R9	Rpl34 (includes others)	ribosomal protein L34	other
0.62	2.4	Q6ZWV7	RPL35	ribosomal protein L35	other
0.62	1.2	O55142	RPL35A	ribosomal protein L35a	other
0.47	1.6	Q6ZWZ4	Rpl36	ribosomal protein L36	other
0.34	3.7	P83882	Rpl36a	ribosomal protein L36A	other
0.51	1.4	P61514	RPL37A	ribosomal protein L37a	other
0.5	1	Q9D8E6	RPL4	ribosomal protein L4	enzyme
0.1	1.5	P47962	RPL5	ribosomal protein L5	other
0.044	1.4	P47911	RPL6	ribosomal protein L6	other
0.24	-1.111	P14148	RPL7	ribosomal protein L7	transcription regulator
0.32	1.1	P12970	RPL7A	ribosomal protein L7a	other
0.04	2	P62918	RPL8	ribosomal protein L8	other
0.0004	6.7	P51410	RPL9	ribosomal protein L9	other
0.092	-1.429	P14869	RPLP0	ribosomal protein lateral stalk subunit P0	other
0.22	-1.667	P47955	Rplp1 (includes others)	ribosomal protein, large, P1	other
0.016	-5	Q91YQ5	RPN1	ribophorin I	enzyme
0.64	1.1	P63325	RPS10	ribosomal protein S10	other
0.46	1.2	P62281	RPS11	ribosomal protein S11	other
0.78	1.7	A0A1W2P7A1	RPS12	ribosomal protein S12	other
0.56	1.1	P62301	RPS13	ribosomal protein S13	other
0.035	2.4	P62264	RPS14	ribosomal protein S14	translation regulator
0.077	4.4	P62843	RPS15	ribosomal protein S15	other
0.036	3.8	P62245	RPS15A	ribosomal protein S15a	other
0.0026	3.5	P14131	RPS16	ribosomal protein S16	other
0.03	2.2	P63276	RPS17	ribosomal protein S17	other
0.53	-1.667	Q9CZX8	RPS19	ribosomal protein S19	other
0.49	1.1	P25444	RPS2	ribosomal protein S2	other
0.54	1.6	P60867	RPS20	ribosomal protein S20	other
0.11	2.5	P62267	RPS23	ribosomal protein S23	translation regulator
0.049	2.1	P62849	RPS24	ribosomal protein S24	other
0.22	2.4	P62852	RPS25	ribosomal protein S25	other
0.46	1.1	P62855	RPS26	ribosomal protein S26	other
0.61	1.3	Q6ZWU9	Rps27/Rps27rt	ribosomal protein S27	other
0.0001	-1.667	P62983	RPS27A	ribosomal protein S27a	other
0.00049	2	P62908	RPS3	ribosomal protein S3	enzyme
0.0026	1.9	P97351	Rps3a1	ribosomal protein S3A1	other
0.0087	1.9	P62702	RPS4Y1	ribosomal protein S4 Y-linked 1	other
0.027	1.7	Q91V55	RPS5	ribosomal protein S5	other
0.0001	3.5	P62754	RPS6	ribosomal protein S6	other
0.39	1.2	P62082	RPS7	ribosomal protein S7	other

Table S1

0.36	1.1	P62242	RPS8	ribosomal protein S8	other
0.17	1.5	Q6ZWN5	RPS9	ribosomal protein S9	translation regulator
0.009	1.6	P14206	RPSA	ribosomal protein SA	translation regulator
0.59	-2.5	P10833	RRAS	RAS related	enzyme
0.53	-1.25	P62071	RRAS2	RAS related 2	enzyme
0.47	1.2	A2AVJ7	Rrbp1	ribosome binding protein 1	transporter
0.47	2.9	P07742	RRM1	ribonucleotide reductase catalytic subunit M1	enzyme
0.59	1.4	P11157	RRM2	ribonucleotide reductase regulatory subunit M2	enzyme
0.012	2.9	Q6P5B0	RRP12	ribosomal RNA processing 12 homolog	other
0.62	1.4	Q91WM3	RRP9	ribosomal RNA processing 9, U3 small nucleolar RNA binding protein	other
0.53	-2	Q9CYH6	RRS1	ribosome biogenesis regulator homolog	other
0.1	3.1	Q8BVY0	RSL1D1	ribosomal L1 domain containing 1	other
0.0021	3	Q99LF4	RTCB	RNA 2',3'-cyclic phosphate and 5'-OH ligase	enzyme
0.0001	-16.667	Q99P72	RTN4	reticulon 4	other
0.42	1.1	P60122	RUVBL1	RuvB like AAA ATPase 1	transcription regulator
0.12	1.5	Q9WTM5	RUVBL2	RuvB like AAA ATPase 2	transcription regulator
0.23	-3.333	P50543	S100a11	S100 calcium binding protein A11	other
0.49	-1.429	A0A0G2JGD2	S100A4	S100 calcium binding protein A4	other
0.82	1	E9QNY8	Sacs	sacsin	other
0.59	1	Q9R1T2	SAE1	SUMO1 activating enzyme subunit 1	enzyme
0.59	1.4	Q60710	SAMHD1	SAM and HD domain containing deoxynucleoside triphosphate triphosphohydrolase 1	enzyme
0.00017	3	Q8C483	SARS	seryl-tRNA synthetase	enzyme
0.34	4	Q6ZPE2	SBF1	SET binding factor 1	phosphatase
0.042	-5	Q8K021	SCAMP1	secretory carrier membrane protein 1	transporter
0.15	-2.5	Q9ERN0	SCAMP2	secretory carrier membrane protein 2	transporter
0.075	-1.667	E9Q855	SCAMP3	secretory carrier membrane protein 3	transporter
0.59	-1.429	Q61009	SCARB1	scavenger receptor class B member 1	transporter
0.82	1	O35114	SCARB2	scavenger receptor class B member 2	transmembrane receptor
0.62	2.4	Q8BRF7	SCFD1	sec1 family domain containing 1	transporter

Table S1

0.82	1	Q3UU41	SCIMP	SLP adaptor and CSK interacting membrane protein	other
0.022	-5	O35988	SDC4	syndecan 4	other
0.17	-1.111	Q3TMX0	SDCBP	syndecan binding protein	enzyme
0.17	5	Q8K2B3	SDHA	succinate dehydrogenase complex flavoprotein subunit A	enzyme
0.82	1	O08547	SEC22B	SEC22 homolog B, vesicle trafficking protein (gene/pseudogene)	other
0.15	-2.5	Q9D662	SEC23B	Sec23 homolog B, coat complex II component	transporter
0.78	1.8	Q6NZC7	SEC23IP	SEC23 interacting protein	other
0.59	-1.429	A2AA71	SEC24A	SEC24 homolog A, COPII coat complex component	transporter
0.024	5.1	Q80ZX0	SEC24B	SEC24 homolog B, COPII coat complex component	transporter
0.38	-1.429	G3X972	SEC24C	SEC24 homolog C, COPII coat complex component	transporter
0.017	2.4	Q3UPL0	SEC31A	SEC31 homolog A, COPII coat complex component	other
0.22	-1.667	P42208	SEPT2	septin 2	enzyme
0.46	-1.429	E9Q1G8	SEPT7	septin 7	other
0.72	-1.429	Q80UG5	SEPT9	septin 9	enzyme
0.23	-2	Q3UMP4	SERBP1	SERPINE1 mRNA binding protein 1	other
0.15	-2.5	Q9QZI8	SERINC1	serine incorporator 1	transporter
0.59	-2.5	Q9QZI9	SERINC3	serine incorporator 3	transporter
0.57	-1.111	Q60854	SERPINB6	serpin family B member 6	other
0.24	4.3	Q91WC0	SETD3	SET domain containing 3	enzyme
0.62	1	Q9D554	SF3A3	splicing factor 3a subunit 3	other
0.34	1.3	G5E866	SF3B1	splicing factor 3b subunit 1	other
0.47	1	Q921M3	SF3B3	splicing factor 3b subunit 3	other
0.16	1.9	Q8VIJ6	SFPQ	splicing factor proline and glutamine rich	transcription regulator
0.02	-5	Q62419	SH3GL1	SH3 domain containing GRB2 like 1, endophilin A2	other
0.59	1.4	Q8R550	SH3KBP1	SH3 domain containing kinase binding protein 1	other
0.47	1.6	P98083	SHC1	SHC adaptor protein 1	other
0.62	2.5	P50431	SHMT1	serine hydroxymethyltransferase 1	enzyme
0.0014	13	Q9CZN7	SHMT2	serine hydroxymethyltransferase 2	enzyme
0.62	2.6	E9Q0Y4	SIPA1	signal-induced proliferation-associated 1	other
0.026	-2.5	Q6P6I8	SIRPA	signal regulatory protein alpha	phosphatase
0.62	1.3	Q6NZR5	SKIV2L	Ski2 like RNA helicase	enzyme

Table S1

0.82	1	P49282	SLC11A2	solute carrier family 11 member 2	transporter
0.23	-2.5	F8WIJ0	SLC12A4	solute carrier family 12 member 4	transporter
0.38	-2.5	Q8VI23	SLC12A8	solute carrier family 12 member 8	transporter
0.66	-1.429	Q8BPX9	SLC15A3	solute carrier family 15 member 3	transporter
0.23	-3.333	Q91W98	SLC15A4	solute carrier family 15 member 4	transporter
0.00083	-3.333	P53986	SLC16A1	solute carrier family 16 member 1	transporter
0.82	1	P57787	SLC16A3	solute carrier family 16 member 3	transporter
0.38	-1.667	B1AT66	SLC16A6	solute carrier family 16 member 6	transporter
0.00031	-2	Q9ESU7	SLC1A5	solute carrier family 1 member 5	transporter
0.82	1	Q61609	SLC20A1	solute carrier family 20 member 1	transporter
0.57	1	Q9EPR4	SLC23A2	solute carrier family 23 member 2	transporter
0.076	-5	Q9JIM1	SLC29A1	solute carrier family 29 member 1 (Augustine blood group)	transporter
0.055	-2	P17809	SLC2A1	solute carrier family 2 member 1	transporter
0.23	-2.5	P32037	SLC2A3	solute carrier family 2 member 3	transporter
0.36	-1.667	A2AR26	SLC2A6	solute carrier family 2 member 6	transporter
0.12	-1.667	Q8CFE6	SLC38A2	solute carrier family 38 member 2	transporter
0.0017	-2.5	P10852	SLC3A2	solute carrier family 3 member 2	transporter
0.076	-5	A2AMH5	SLC44A1	solute carrier family 44 member 1	transporter
0.02	-2.5	F8VQC9	SLC4A7	solute carrier family 4 member 7	transporter
0.087	-3.333	Q9JKZ2	SLC5A3	solute carrier family 5 member 3	transporter
0.15	-2	G5E8Z4	SLC6A12	solute carrier family 6 member 12	transporter
0.59	-1.429	O35316	SLC6A6	solute carrier family 6 member 6	transporter
0.013	-2.5	Q09143	SLC7A1	solute carrier family 7 member 1	transporter
0.0035	-5	Q8K078	SLCO4A1	solute carrier organic anion transporter family member 4A1	transporter
0.62	2	B1ARD6	SLFN13	schlafen family member 13	enzyme
0.47	2.9	Q8CBA2	SLFN5	schlafen family member 5	enzyme
0.82	1	Q80TR4	SLIT1	slit guidance ligand 1	other
0.62	2.4	Q91ZW3	SMARCA5	SWI/SNF related, matrix associated, actin dependent regulator of chromatin, subfamily a, member 5	transcription regulator

Table S1

0.62	1.2	Q04692	SMARCAD1	SWI/SNF-related, matrix-associated actin-dependent regulator of chromatin, subfamily a, containing DEAD/H box 1	enzyme
0.34	3.6	Q61466	SMARCD1	SWI/SNF related, matrix associated, actin dependent regulator of chromatin, subfamily d, member 1	transcription regulator
0.34	3.6	Q8CG48	SMC2	structural maintenance of chromosomes 2	transporter
0.38	-2	Q9CW03	SMC3	structural maintenance of chromosomes 3	other
0.62	2	Q8CG47	SMC4	structural maintenance of chromosomes 4	transporter
0.24	2.6	Q6P5D8	SMCHD1	structural maintenance of chromosomes flexible hinge domain containing 1	enzyme
0.34	3.6	Q8BKX6	SMG1	SMG1, nonsense mediated mRNA decay associated PI3K related kinase	kinase
0.15	-2	P58242	SMPDL3B	sphingomyelin phosphodiesterase acid like 3B	enzyme
0.59	1.4	Q3UKJ7	SMU1	SMU1, DNA replication regulator and spliceosomal factor	other
0.016	-5	Q9D3L3	SNAP23	synaptosome associated protein 23	transporter
0.0001	4.2	Q78PY7	SND1	staphylococcal nuclease and tudor domain containing 1	enzyme
0.0001	4.7	Q6P4T2	SNRNP200	small nuclear ribonucleoprotein U5 subunit 200	enzyme
0.077	4.4	Q6PE01	SNRNP40	small nuclear ribonucleoprotein U5 subunit 40	other
0.59	1.4	Q62376	SNRNP70	small nuclear ribonucleoprotein U1 subunit 70	other
0.38	-1.429	P27048	SNRPB	small nuclear ribonucleoprotein polypeptides B and B1	other
0.34	3.9	P62315	SNRPD1	small nuclear ribonucleoprotein D1 polypeptide	other
0.53	-1.667	P62305	Snrpe	small nuclear ribonucleoprotein E	other
0.66	-1.429	Q61235	SNTB2	syntrophin beta 2	other
0.61	-1.25	Q6NZD2	SNX1	sorting nexin 1	transporter
0.82	1	Q8BVL3	SNX17	sorting nexin 17	transporter
0.3	-1.429	Q9CWK8	SNX2	sorting nexin 2	transporter
0.54	-1.111	Q3UHD6	SNX27	sorting nexin family member 27	other
0.38	-1.429	Q78ZM0	SNX3	sorting nexin 3	transporter

Table S1

0.47	1.6	Q91YJ2	SNX4	sorting nexin 4	transporter
0.35	-1.667	Q9D8U8	SNX5	sorting nexin 5	transporter
0.82	1	Q6P8X1	SNX6	sorting nexin 6	transporter
0.052	-2	Q91VH2	SNX9	sorting nexin 9	transporter
0.36	-2	P08228	SOD1	superoxide dismutase 1	enzyme
0.62	2.5	A0A1L1SST5	SPG21	SPG21, maspardin	enzyme
0.46	-1.25	F8WIP8	SPP1	secreted phosphoprotein 1	cytokine
0.82	1	Q64105	SPR	sepiapterin reductase	enzyme
0.074	-2	Q924S8	SPRED1	sprouty related EVH1 domain containing 1	other
0.62	2.2	Q9WTP2	SPRY4	sprouty RTK signaling antagonist 4	other
0.72	-1.429	Q3TFQ1	SPRYD7	SPRY domain containing 7	other
0.25	-1.25	Q64337	SQSTM1	sequestosome 1	transcription regulator
0.0001	-10	Q91Z67	SRGAP2	SLIT-ROBO Rho GTPase activating protein 2	other
0.0001	-12.5	Q6P069	SRI	sorcin	transporter
0.16	1.9	Q64674	SRM	spermidine synthase	enzyme
0.64	1.4	Q9D104	SRP19	signal recognition particle 19	other
0.14	2.8	Q8BMA6	SRP68	signal recognition particle 68	other
0.014	9.6	F8VQC1	SRP72	signal recognition particle 72	kinase
0.62	2.2	O70551	SRPK1	SRSF protein kinase 1	kinase
0.2	1.6	Q99MR6	SRRT	serrate, RNA effector molecule	other
0.36	-2	P84104	SRSF3	serine and arginine rich splicing factor 3	other
0.53	-1.667	Q3TWW8	SRSF6	serine and arginine rich splicing factor 6	other
0.019	1.9	P32067	SSB	Sjogren syndrome antigen B	enzyme
0.077	6.6	Q8R2K3	SSBP1	single stranded DNA binding protein 1	other
0.33	1.7	Q08943	SSRP1	structure specific recognition protein 1	transcription regulator
0.23	-2	F8WJK8	ST13	ST13, Hsp70 interacting protein	other
0.82	1	Q64692	ST8SIA4	ST8 alpha-N-acetyl-neuraminide alpha-2,8-sialyltransferase 4	enzyme
0.24	-1.667	O88811	STAM2	signal transducing adaptor molecule 2	other
0.23	-3.333	Q9CQ26	STAMBP	STAM binding protein	enzyme
0.47	1.9	Q61542	STARD3	StAR related lipid transfer domain containing 3	transporter
0.14	2.1	A0A087WSP5	STAT1	signal transducer and activator of transcription 1	transcription regulator
0.78	1.7	Q9QXJ2	STAT2	signal transducer and activator of transcription 2	transcription regulator
0.36	-2	E9QN92	STEAP3	STEAP3 metalloreductase	transporter

Table S1

0.23	-2.5	Q60864	STIP1	stress induced phosphoprotein 1	other
0.15	-3.333	O55098	STK10	serine/threonine kinase 10	kinase
0.59	1.4	Q3TAA7	STK11IP	serine/threonine kinase 11 interacting protein	other
0.0021	-1.667	P54116	STOM	stomatin	other
0.21	1.6	Q9Z1Z2	STRAP	serine/threonine kinase receptor associated protein	other
0.82	1	B2RQS1	STRN3	striatin 3	transcription regulator
0.53	-1.667	Q9WUD1	STUB1	STIP1 homology and U-box containing protein 1	enzyme
0.38	-2	Q9ER00	STX12	syntaxin 12	other
0.59	-2	Q80W45	STX2	syntaxin 2	transporter
0.62	2.6	Q64704	STX3	syntaxin 3	transporter
0.48	-1.111	P70452	STX4	syntaxin 4	transporter
0.47	1.3	Q9JKK1	STX6	syntaxin 6	transporter
0.53	1	Q8BH40	STX7	syntaxin 7	transporter
0.095	-3.333	O88983	STX8	syntaxin 8	other
0.72	-1.25	O08599	STXBP1	syntaxin binding protein 1	transporter
0.18	-1.429	Q64324	STXBP2	syntaxin binding protein 2	transporter
0.22	-1.667	Q60770	STXBP3	syntaxin binding protein 3	transporter
0.34	3.6	G3X956	SUPT16H	SPT16 homolog, facilitates chromatin remodeling subunit	transcription regulator
0.0029	4.5	O55201	SUPT5H	SPT5 homolog, DSIF elongation factor subunit	transcription regulator
0.62	1.3	P70279	SURF6	surfeit 6	other
0.59	-1.429	P48025	SYK	spleen associated tyrosine kinase	kinase
0.47	2.9	F8WJD4	SYMPK	symplekin	other
0.47	1.1	G3UZ48	SYNCRIP	synaptotagmin binding cytoplasmic RNA interacting protein	other
0.35	-1.667	P30548	TACR1	tachykinin receptor 1	G-protein coupled receptor
0.076	-5	Q9WVA4	TAGLN2	transgelin 2	other
0.61	1.3	A0A1B0GR11	TALDO1	transaldolase 1	enzyme
0.59	-1.111	Q0VGY8	TANC1	tetratricopeptide repeat, ankyrin repeat and coiled-coil containing 1	other
0.14	1.6	Q921F2	TARDBP	TAR DNA binding protein	transcription regulator
0.48	-1.25	Q9D0R2	TARS	threonyl-tRNA synthetase	enzyme
0.29	-1.429	Q3UKC1	TAX1BP1	Tax1 binding protein 1	other
0.51	1.2	Q9CXF4	TBC1D15	TBC1 domain family member 15	other
0.14	2.1	Q8BYA0	TBCD	tubulin folding cofactor D	other
0.47	1.4	Q9WUN2	TBK1	TANK binding kinase 1	kinase
0.59	1.4	Q8C4J7	TBL3	transducin beta like 3	peptidase

Table S1

0.44	-1.25	Q9JHF5	TCIRG1	T cell immune regulator 1, ATPase H+ transporting V0 subunit a3	enzyme
0.0001	2.1	P11983	TCP1	t-complex 1	other
0.59	-2.5	Q9Z1A1	TFG	TRK-fused gene	other
0.0001	-2.5	Q62351	TFRC	transferrin receptor	transporter
0.048	-2.5	Q62312	TGFBR2	transforming growth factor beta receptor 2	kinase
0.17	3.3	A8C756	THADA	THADA, armadillo repeat containing	other
0.38	-1.429	B1AZI6	THOC2	THO complex 2	other
0.34	3.6	Q9R1X4	TIMELESS	timeless circadian regulator	other
0.0071	-2	P40142	TKT	transketolase	enzyme
0.62	2.1	Q8C0V0	TLK1	tousled like kinase 1	kinase
0.012	-1.429	P26039	TLN1	talin 1	other
0.59	-2	Q9QUN7	TLR2	toll like receptor 2	transmembrane receptor
0.35	-1.25	P58681	TLR7	toll like receptor 7	transmembrane receptor
0.59	-1.429	Q9ET30	TM9SF3	transmembrane 9 superfamily member 3	transporter
0.82	1	Q8BH24	TM9SF4	transmembrane 9 superfamily member 4	transporter
0.82	1	A0A1Y7VM54	TMED10	transmembrane p24 trafficking protein 10	transporter
0.38	-1.429	Q8VC04	TMEM106A	transmembrane protein 106A	other
0.59	-1.429	D3Z0M2	TMEM106B	transmembrane protein 106B	other
0.82	1	P52875	TMEM165	transmembrane protein 165	other
0.36	-1.667	Q9R1Q6	TMEM176B	transmembrane protein 176B	other
0.023	-3.333	D3YVM2	TMEM59	transmembrane protein 59	peptidase
0.82	1	Q91YT8	TMEM63A	transmembrane protein 63A	other
0.59	-2	Q9JJR8	TMEM9B	TMEM9 domain family member B	other
0.14	-5	Q80YX1	TNC	tenascin C	other
0.59	1.4	Q60769	TNFAIP3	TNF alpha induced protein 3	enzyme
0.51	1.2	P25119	TNFRSF1B	TNF receptor superfamily member 1B	transmembrane receptor
0.56	1	D3Z2W0	TNIP1	TNFAIP3 interacting protein 1	other
0.82	1	Q6PFX9	TNKS	tankyrase	enzyme
0.34	2.4	Q8BFY9	TNPO1	transportin 1	transporter
0.34	3.6	Q6P2B1	TNPO3	transportin 3	other
0.34	3.4	Q5SSZ5	TNS3	tensin 3	phosphatase
0.78	1.7	Q9D2E2	TOE1	target of EGR1, exonuclease	enzyme
0.033	-2.5	Q9QZ06	TOLLIP	toll interacting protein	other
0.48	-1.111	Q3UDC3	TOM1	target of myb1 membrane trafficking protein	transporter

Table S1

0.59	-1.429	Q923U0	TOM1L1	target of myb1 like 1 membrane trafficking protein	other
0.47	2.9	Q5SRX1	TOM1L2	target of myb1 like 2 membrane trafficking protein	transporter
0.61	1.3	Q04750	TOP1	DNA topoisomerase I	enzyme
0.41	1.2	Q01320	TOP2A	DNA topoisomerase II alpha	enzyme
0.62	2	A2A5R0	TP53RK	TP53 regulating kinase	kinase
0.054	-1.667	P17751	TPI1	triosephosphate isomerase 1	enzyme
0.14	-3.333	D3Z2H9	Tpm3-rs7	tropomyosin 3, related sequence 7	other
0.39	1.8	Q64514	TPP2	tripeptidyl peptidase 2	peptidase
0.53	-1.111	E9PWG2	TRAPPC8	trafficking protein particle complex 8	transporter
0.59	1.4	Q91XB0	TREX1	three prime repair exonuclease 1	enzyme
0.0045	-2.5	Q8BVW3	TRIM14	tripartite motif containing 14	other
0.077	4.3	Q61510	TRIM25	tripartite motif containing 25	transcription regulator
0.59	-1.111	Q99PN3	TRIM26	tripartite motif containing 26	other
0.0001	6.7	Q62318	TRIM28	tripartite motif containing 28	transcription regulator
0.38	-1.429	A0A0R4J0Q6	TRIM56	tripartite motif containing 56	enzyme
0.24	2.5	G5E870	TRIP12	thyroid hormone receptor interactor 12	enzyme
0.59	1.4	Q3TX08	TRMT1	tRNA methyltransferase 1	enzyme
0.3	2.6	Q8BNV1	TRMT2A	tRNA methyltransferase 2 homolog A	kinase
0.47	2.9	Q9D0C4	TRMT5	tRNA methyltransferase 5	other
0.82	1	Q8CE96	TRMT6	tRNA methyltransferase 6	other
0.59	1.4	Q80XC2	TRMT61A	tRNA methyltransferase 61A	enzyme
0.0031	-10	Q9WTR1	TRPV2	transient receptor potential cation channel subfamily V member 2	ion channel
0.017	-2.5	Q61187	TSG101	tumor susceptibility 101	transcription regulator
0.14	-5	Q8QZY6	TSPAN14	tetraspanin 14	other
0.05	2.3	Q5SWD9	TSR1	TSR1, ribosome maturation factor	other
0.59	-2.5	P23591	TSTA3	tissue specific transplantation antigen P35B	enzyme
0.077	4.4	F8VPK0	TTC37	tetratricopeptide repeat domain 37	other
0.12	4	Q3UDE2	TTLL12	tubulin tyrosine ligase like 12	other
0.64	1	A2ASS6	TTN	titin	kinase
0.38	-2.5	Q3TH73	TTYH2	tweety family member 2	ion channel

Table S1

0.15	-2	Q6P5F7	TTYH3	tweety family member 3	ion channel
0.54	1	P68369	TUBA1A	tubulin alpha 1a	other
0.00058	1.5	P05213	TUBA1B	tubulin alpha 1b	other
0.54	1.2	A0A0A0MQA5	TUBA4A	tubulin alpha 4a	other
0.0001	1.6	P99024	TUBB	tubulin beta class I	other
0.64	-1.25	Q7TMM9	TUBB2A	tubulin beta 2A class IIa	other
0.41	1.3	P68372	TUBB4B	tubulin beta 4B class IVb	other
0.0047	1.9	Q922F4	TUBB6	tubulin beta 6 class V	other
0.59	1.4	P83887	TUBG1	tubulin gamma 1	other
0.38	-2.5	Q9JMH6	TXNRD1	thioredoxin reductase 1	enzyme
0.051	-3.333	A0A140LHP7	TYROBP	TYRO protein tyrosine kinase binding protein	transmembrane receptor
0.36	-1.25	Q9D883	U2af1	U2 small nuclear ribonucleoprotein auxiliary factor (U2AF) 1	other
0.48	1	Q3TW96	UAP1L1	UDP-N-acetylglucosamine pyrophosphorylase 1 like 1	other
0.072	1.2	Q02053	UBA1	ubiquitin like modifier activating enzyme 1	enzyme
0.39	2.2	Q9Z1F9	UBA2	ubiquitin like modifier activating enzyme 2	enzyme
0.82	1	Q8C878	UBA3	ubiquitin like modifier activating enzyme 3	enzyme
0.59	1.4	Q8VE47	UBA5	ubiquitin like modifier activating enzyme 5	enzyme
0.82	1	Q8C7R4	UBA6	ubiquitin like modifier activating enzyme 6	enzyme
0.82	1	Q9DBK7	UBA7	ubiquitin like modifier activating enzyme 7	enzyme
0.62	1.4	A0A0G2JGL0	UBE2D3	ubiquitin conjugating enzyme E2 D3	enzyme
0.53	-1.111	P61082	UBE2M	ubiquitin conjugating enzyme E2 M	enzyme
0.38	-2.5	P61089	UBE2N	ubiquitin conjugating enzyme E2 N	enzyme
0.82	1	Q9ES00	UBE4B	ubiquitination factor E4B	enzyme
0.59	-2	Q9Z2M6	UBL3	ubiquitin like 3	other
0.036	3.3	A2AN08	UBR4	ubiquitin protein ligase E3 component n-recognin 4	enzyme
0.78	1.8	Q9WUP7	UCHL5	ubiquitin C-terminal hydrolase L5	peptidase
0.47	2.9	Q99PM9	UCK2	uridine-cytidine kinase 2	kinase
0.82	1	O88693	UGCG	UDP-glucose ceramide glucosyltransferase	enzyme
0.38	1.3	P13439	UMPS	uridine monophosphate synthetase	enzyme
0.72	-1.111	B1AQD9	UNC119	unc-119 lipid binding chaperone	other
0.087	-3.333	E9PYK0	UNC93B1	unc-93 homolog B1, TLR signaling regulator	transporter
0.00012	11	Q9EPU0	UPF1	UPF1, RNA helicase and ATPase	enzyme
0.35	-1.667	E9PYI8	USP14	ubiquitin specific peptidase 14	peptidase

Table S1

0.12	3	Q8R5H1	USP15	ubiquitin specific peptidase 15	peptidase
0.62	2.6	E9PV45	USP24	ubiquitin specific peptidase 24	peptidase
0.34	3.8	P35123	USP4	ubiquitin specific peptidase 4	peptidase
0.52	1.2	Q3U4W8	USP5	ubiquitin specific peptidase 5	peptidase
0.59	1.4	F8VPX1	USP7	ubiquitin specific peptidase 7	peptidase
0.24	-1.429	Q80U87	USP8	ubiquitin specific peptidase 8	peptidase
0.34	2.1	Q4FE56	USP9X	ubiquitin specific peptidase 9 X-linked	peptidase
0.47	3.3	Q5SSI6	UTP18	UTP18, small subunit processome component	other
0.62	2.2	A0A0R4J114	UTP25	UTP25, small subunit processor component	other
0.59	1.4	Q8R2N2	UTP4	UTP4, small subunit processome component	other
0.62	1.1	Q80WQ2	VAC14	Vac14, PIKFYVE complex component	other
0.62	1.1	Q8BSN6	VAMP4	vesicle associated membrane protein 4	other
0.23	-2.5	P70280	VAMP7	vesicle associated membrane protein 7	transporter
0.44	-1.25	O70404	VAMP8	vesicle associated membrane protein 8	transporter
0.00014	2	Q9Z1Q9	VARS	valyl-tRNA synthetase	enzyme
0.33	1.3	P70460	VASP	vasodilator stimulated phosphoprotein	other
0.053	-2	Q62465	VAT1	vesicle amine transport 1	transporter
0.62	2	P27870	VAV1	vav guanine nucleotide exchange factor 1	transcription regulator
0.38	-3.333	Q64727	VCL	vinculin	enzyme
0.0026	-1.667	Q01853	VCP	valosin containing protein	enzyme
0.82	1	G3UX26	VDAC2	voltage dependent anion channel 2	ion channel
0.06	1.8	P20152	VIM	vimentin	other
0.82	1	L7N2E9	Vmn2r2	vomeronasal 2, receptor 2	other
0.051	4.7	Q91W86	VPS11	VPS11, CORVET/HOPS core subunit	transporter
0.36	-1.667	Q8BX70	VPS13C	vacuolar protein sorting 13 homolog C	other
0.22	2.5	G3X8X7	VPS16	VPS16, CORVET/HOPS core subunit	transporter
0.78	1.7	Q8R307	VPS18	VPS18, CORVET/HOPS core subunit	transporter
0.38	-2.5	Q9CQ80	VPS25	vacuolar protein sorting 25 homolog	other
0.23	-2	Q9D1C8	VPS28	VPS28, ESCRT-I subunit	transporter
0.82	1	D3YYD5	VPS29	VPS29, retromer complex component	transporter
0.82	1	Q9D2N9	VPS33A	VPS33A, CORVET/HOPS core subunit	transporter
0.51	1	Q9EQH3	VPS35	VPS35, retromer complex component	transporter

Table S1

0.38	-1.667	Q91XD6	VPS36	vacuolar protein sorting 36 homolog	other
0.00078	-12.5	Q8R0J7	VPS37B	VPS37B, ESCRT-I subunit	other
0.095	-2.5	Q8R105	VPS37C	VPS37C, ESCRT-I subunit	other
0.47	2.9	Q5KU39	VPS41	VPS41, HOPS complex subunit	transporter
0.0028	-3.333	P46467	VPS4B	vacuolar protein sorting 4 homolog B	transporter
0.12	5.8	Q3UVL4	VPS51	VPS51, GARP complex subunit	other
0.59	1.4	Q8C754	VPS52	VPS52, GARP complex subunit	other
0.24	3	Q8CCB4	VPS53	VPS53, GARP complex subunit	other
0.82	1	Q80X41	VRK1	vaccinia related kinase 1	kinase
0.23	-3.333	Q9CR26	VTA1	vesicle trafficking 1	other
0.62	1.3	Q91XH6	VTI1B	vesicle transport through interaction with t-SNAREs 1B	transporter
0.54	-1.111	Q99KC8	VWA5A	von Willebrand factor A domain containing 5A	other
0.049	1.9	P32921	WARS	tryptophanyl-tRNA synthetase	enzyme
0.14	-5	Q8BH43	WASF2	WAS protein family member 2	other
0.59	-2	Q3UMB9	WASHC4	WASH complex subunit 4	other
0.59	1.4	Q8C2E7	WASHC5	WASH complex subunit 5	other
0.82	1	P97765	WBP2	WW domain binding protein 2	transcription regulator
0.47	3.1	E9Q2M9	WDFY4	WDFY family member 4	other
0.82	1	O88342	WDR1	WD repeat domain 1	other
0.59	1.4	G5E8J3	WDR11	WD repeat domain 11	other
0.34	3.7	Q4VBE8	WDR18	WD repeat domain 18	other
0.59	1.4	Q8BHB4	WDR3	WD repeat domain 3	other
0.59	1.4	Q8K4P0	WDR33	WD repeat domain 33	other
0.59	1.4	Q3TAQ9	WDR36	WD repeat domain 36	other
0.59	1.4	Q6ZQL4	WDR43	WD repeat domain 43	other
0.82	1	Q9CX97	WDR55	WD repeat domain 55	other
0.34	3.7	Q9ERF3	WDR61	WD repeat domain 61	other
0.64	1.1	K4DI77	WDR81	WD repeat domain 81	other
0.82	1	Q8BFQ4	WDR82	WD repeat domain 82	other
0.47	1.7	S4R1X1	WDR91	WD repeat domain 91	other
0.038	-2	A0A1D5RM92	WWP2	WW domain containing E3 ubiquitin protein ligase 2	enzyme
0.24	2.9	Q9DCD2	XAB2	XPA binding protein 2	other
0.057	-2.5	S4R1I3	XPNPEP1	X-prolyl aminopeptidase 1	peptidase
0.022	8.7	Q6P5F9	XPO1	exportin 1	transporter
0.07	3	Q924C1	XPO5	exportin 5	transporter
0.59	1.4	Q9EPK7	XPO7	exportin 7	transporter
0.38	-2.5	Q9Z0U0	XPR1	xenotropic and polytropic retrovirus receptor 1	G-protein coupled receptor
0.22	2.9	Q9DBR1	XRN2	5'-3' exoribonuclease 2	enzyme
0.82	1	A2A7S7	YARS	tyrosyl-tRNA synthetase	enzyme

Table S1

0.38	1.2	P62960	YBX1	Y-box binding protein 1	transcription regulator
0.78	1.8	Q9JKB3	YBX3	Y-box binding protein 3	transcription regulator
0.23	-2	Q9CQV8	YWHAB	tyrosine 3-monooxygenase/tryptophan 5-monooxygenase activation protein beta	other
0.11	-2	P62259	YWHAE	tyrosine 3-monooxygenase/tryptophan 5-monooxygenase activation protein epsilon	other
0.023	-3.333	P61982	YWHAG	tyrosine 3-monooxygenase/tryptophan 5-monooxygenase activation protein gamma	other
0.53	-1.111	P68510	YWHAH	tyrosine 3-monooxygenase/tryptophan 5-monooxygenase activation protein eta	transcription regulator
0.35	-1.667	P68254	YWHAQ	tyrosine 3-monooxygenase/tryptophan 5-monooxygenase activation protein theta	other
0.1	-1.667	P63101	YWHAZ	tyrosine 3-monooxygenase/tryptophan 5-monooxygenase activation protein zeta	enzyme
0.063	-2.5	Q3UPF5	ZC3HAV1	zinc finger CCCH-type containing, antiviral 1	other
0.82	1	Q9CWU2	ZDHHC13	zinc finger DHHC-type containing 13	transcription regulator
0.82	1	Q80TN5	ZDHHC17	zinc finger DHHC-type containing 17	enzyme
0.23	-2	Q5Y5T1	ZDHHC20	zinc finger DHHC-type containing 20	enzyme
0.59	-1.429	Q8VDZ4	ZDHHC5	zinc finger DHHC-type containing 5	enzyme
0.82	1	P59268	ZDHHC9	zinc finger DHHC-type containing 9	enzyme
0.82	1	B1AWL2	ZNF462	zinc finger protein 462	transcription regulator
0.053	4.3	O54692	ZW10	zw10 kinetochore protein	other
0.15	-2	E9Q5W5	ZZEF1	zinc finger ZZ-type and EF-hand domain containing 1	other
0.0001	-12.5	CAS1_BOVIN			
0.022	-10	CAS2_BOVIN			
0.23	-3.333	P10400			
0.063	-3.333	P10404			
0.23	-3.333	E9PZF0			
0.38	-2.5	KRA61_SHEEP			
0.15	-2.5	P0AA28			
0.0001	-2.5	TRYP_PIG			
0.59	-2	E9PY39			

Table S1

0.59	-1.429	Q8ZJV0			
0.59	-1.429	A2AGH5			
0.094	-1.429	Q9CPX4			
0.42	-1.25	P68433			
0.53	-1.111	P17095			
0.82	1	P84244			
0.82	1	Q8ZPD6			
0.82	1	Q8ZQT5			
0.82	1	Q8ZRP0			
0.82	1	Q7CPQ6			
0.82	1	Q7CPX8			
0.82	1	Q8ZLU4			
0.82	1	Q8ZN72			
0.82	1	Q8ZQ10			
0.82	1	Q8ZRC1			
0.82	1	Q8ZRQ2			
0.82	1	Q93GL9			
0.034	1.3	P10853			
0.59	1.4	P06185			
0.54	1.4	A0A140T8M7			
0.59	1.4	Q8ZLZ4			
0.003	1.6	P62806			
0.78	1.7	P37432			
0.3	1.7	E9QAZ2			
0.78	1.7	Q8ZNL0			
0.78	1.8	Q7CQW9			
0.78	1.8	Q8ZR40			
0.0003	2	Q6GSS7			
0.62	2	A2A4P3			
0.62	2.2	P0A1D3			
0.62	2.5	Q8ZPT3			
0.14	2.8	A0A1B0GS68			
0.47	2.9	P0A1H5			
0.47	2.9	Q8ZMN0			
0.34	3.7	Q7CQV8			
0.24	4.3	P14576			
0.17	5	Q8ZRJ9			
0.12	5.8	P0A1X0			
0.12	5.8	P52616			
0.12	5.8	Q7CQN4			
0.12	6	O30916			
0.0056	11	P0A263			
0.0035	12	P23988			
0.00019	16	P37592			
0.0001	27	P02936			
0.0001	89	P06179			
