# Supplementary material for: Antigen-encapsulating host extracellular vesicles derived from Salmonella-infected cells stimulate pathogen-specific Th1-type responses in vivo
Source: PLoS Pathog. 2021 May 6;17(5):e1009465. doi: 10.1371/journal.ppat.1009465 (PMC8101724; doi:10.1371/journal.ppat.1009465)
Supplement: S3 Table — Calculated fold change was based on the normalized spectral values (48 hpi in comparison to 24 hpi as control), and p-values were calculated by using Fisher’s test. ID, Symbol, and Entrez Gene Name have been shown (Ingenuity Pathway Analysis, IPA, Qiagen). Only significant values are shown for differentially regulated proteins. (PDF) [file ppat.1009465.s016.pdf]

Table S3

Expr p-value	Expr Fold Change	ID	Symbol	Entrez Gene Name	Location	Type(s)
0.0001	-10000	B2RQC6	CAD	carbamoyl-phosphate synthetase 2, aspartate transcarbamylase, and dihydroorotase	Cytoplasm	enzyme
0.0001	-10000	Q61656	DDX5	DEAD-box helicase 5	Nucleus	enzyme
0.0001	-10000	P13864	DNMT1	DNA methyltransferase 1	Nucleus	enzyme
0.0001	-10000	Q8BTM8	FLNA	filamin A	Cytoplasm	other
0.0001	-10000	P11152	LPL	lipoprotein lipase	Cytoplasm	enzyme
0.0001	-10000	P97310	MCM2	minichromosome maintenance complex component 2	Nucleus	enzyme
0.0001	-10000	Q5SUR0	PFAS	phosphoribosylformylglycinamidine synthase	Cytoplasm	enzyme
0.0001	-10000	Q6P4T2	SNRNP200	small nuclear ribonucleoprotein U5 subunit 200	Nucleus	enzyme
0.0001	-10000	P05213	TUBA1B	tubulin alpha 1b	Cytoplasm	other
0.0001	-10000	P99024	TUBB	tubulin beta class I	Cytoplasm	other
0.0001	-10000	P06179				
0.00013	-7692.308	P80314	CCT2	chaperonin containing TCP1 subunit 2	Cytoplasm	kinase
0.00013	-7692.308	P60843	EIF4A1	eukaryotic translation initiation factor 4A1	Cytoplasm	translation regulator
0.00013	-7692.308	Q07797	LGALS3BP	galectin 3 binding protein	Plasma Membrane	transmembrane receptor
0.00017	-5882.353	P11440	CDK1	cyclin dependent kinase 1	Nucleus	kinase
0.00022	-4545.455	P80316	CCT5	chaperonin containing TCP1 subunit 5	Cytoplasm	other
0.00022	-4545.455	Q62318	TRIM28	tripartite motif containing 28	Nucleus	transcription regulator
0.00023	-4347.826	Q6PDQ2	CHD4	chromodomain helicase DNA binding protein 4	Nucleus	enzyme
0.00027	-3703.704	Q62167	DDX3X	DEAD-box helicase 3 X-linked	Cytoplasm	enzyme
0.00031	-3225.806	Q8R1B4	EIF3C	eukaryotic translation initiation factor 3 subunit C	Cytoplasm	translation regulator
0.00035	-2857.143	Q8K310	MATR3	matrin 3	Nucleus	other
0.00043	-2325.581	Q9JHU4	DYNC1H1	dynein cytoplasmic 1 heavy chain 1	Cytoplasm	peptidase
0.00043	-2325.581	P49717	MCM4	minichromosome maintenance complex component 4	Nucleus	enzyme
0.00053	-1886.792	Q6NZJ6	EIF4G1	eukaryotic translation initiation factor 4 gamma 1	Cytoplasm	translation regulator
0.00054	-1851.852	Q7TPV4	MYBBP1A	MYB binding protein 1a	Nucleus	transcription regulator
0.00058	-1724.138	P25206	MCM3	minichromosome maintenance complex component 3	Nucleus	enzyme
0.00062	-1612.903	Q61881	MCM7	minichromosome maintenance complex component 7	Nucleus	enzyme
0.00064	-1562.5	D3Z158	Qars	glutaminyl-tRNA synthetase	Cytoplasm	enzyme
0.00075	-1333.333	P12382	PFKL	phosphofructokinase, liver type	Cytoplasm	kinase
0.0011	-909.091	P80318	CCT3	chaperonin containing TCP1 subunit 3	Cytoplasm	other
0.0012	-833.333	P52293	KPNA2	karyopherin subunit alpha 2	Nucleus	transporter
0.0012	-833.333	Q9R1P4	PSMA1	proteasome subunit alpha 1	Cytoplasm	peptidase
0.0013	-769.231	P62264	RPS14	ribosomal protein S14	Cytoplasm	translation regulator

Table S3

0.0014	-714.286	P19096	FASN	fatty acid synthase	Cytoplasm	enzyme
0.0014	-714.286	P06869	PLAU	plasminogen activator, urokinase	Extracellular Space	peptidase
0.0015	-666.667	Q9JIK5	DDX21	DExD-box helicase 21	Nucleus	enzyme
0.0016	-625	Q9CZU3	MTREX	Mtr4 exosome RNA helicase	Nucleus	other
0.0023	-434.783	Q9EPU0	UPF1	UPF1, RNA helicase and ATPase	Nucleus	enzyme
0.0024	-416.667	P70460	VASP	vasodilator stimulated phosphoprotein	Plasma Membrane	other
0.0025	-400	A0A0R4J0V5	POLR2A	RNA polymerase II subunit A	Nucleus	enzyme
0.0032	-312.5	P80315	CCT4	chaperonin containing TCP1 subunit 4	Cytoplasm	other
0.0033	-303.03	P52480	PKM	pyruvate kinase M1/2	Cytoplasm	kinase
0.0034	-294.118	Q78PY7	SND1	staphylococcal nuclease and tudor domain containing 1	Nucleus	enzyme
0.0035	-285.714	Q91W50	CSDE1	cold shock domain containing E1	Cytoplasm	enzyme
0.0037	-270.27	G5E924	HNRNPL	heterogeneous nuclear ribonucleoprotein L	Nucleus	other
0.0038	-263.158	Q9QXS1	PLEC	plectin	Cytoplasm	other
0.0041	-243.902	Q02053	UBA1	ubiquitin like modifier activating enzyme 1	Cytoplasm	enzyme
0.0042	-238.095	Q08288	LYAR	Ly1 antibody reactive	Plasma Membrane	other
0.0042	-238.095	Q6ZQH8	NUP188	nucleoporin 188	Nucleus	other
0.0043	-232.558	Q6ZQ08	CNOT1	CCR4-NOT transcription complex subunit 1	Cytoplasm	other
0.0045	-222.222	P11983	TCP1	t-complex 1	Cytoplasm	other
0.0062	-161.29	Q501J6	DDX17	DEAD-box helicase 17	Nucleus	enzyme
0.0071	-140.845	Q6PGC1	DHX29	DExH-box helicase 29	Cytoplasm	enzyme
0.0074	-135.135	P55097	CTSK	cathepsin K	Cytoplasm	peptidase
0.0078	-128.205	O70194	EIF3D	eukaryotic translation initiation factor 3 subunit D	Cytoplasm	other
0.0087	-114.943	O70469	DOK2	docking protein 2	Plasma Membrane	other
0.0087	-114.943	P52431	POLD1	DNA polymerase delta 1, catalytic subunit	Nucleus	enzyme
0.0087	-114.943	F8VPK0	TTC37	tetratricopeptide repeat domain 37	Nucleus	other
0.0094	-106.383	ALBU_HUMAN	ALB	albumin	Extracellular Space	transporter
0.0095	-105.263	Q91V55	RPS5	ribosomal protein S5	Cytoplasm	other
0.0096	-104.167	O88844	IDH1	isocitrate dehydrogenase (NADP(+)) 1, cytosolic	Cytoplasm	enzyme
0.01	-100	P70168	KPNB1	karyopherin subunit beta 1	Nucleus	transporter
0.01	-100	Q922D8	MTHFD1	methylenetetrahydrofolate dehydrogenase, cyclohydrolase and formyltetrahydrofolate synthetase 1	Cytoplasm	enzyme
0.011	-90.909	Q99PV0	PRPF8	pre-mRNA processing factor 8	Nucleus	other
0.011	-90.909	P63276	RPS17	ribosomal protein S17	Cytoplasm	other
0.012	-83.333	Q6P542	ABCF1	ATP binding cassette subfamily F member 1	Cytoplasm	transporter

Table S3

0.013	-76.923	Q9DCL9	PAICS	phosphoribosylaminoimidazole carboxylase and phosphoribosylaminoimidazolesuccinocarboxamide synthase	Cytoplasm	enzyme
0.014	-71.429	Q8CGC7	EPRS	glutamyl-prolyl-tRNA synthetase	Cytoplasm	enzyme
0.014	-71.429	Q3THS6	MAT2A	methionine adenosyltransferase 2A	Cytoplasm	enzyme
0.014	-71.429	P52432	POLR1C	RNA polymerase I and III subunit C	Nucleus	enzyme
0.015	-66.667	Q922F4	TUBB6	tubulin beta 6 class V	Cytoplasm	other
0.016	-62.5	A0A1B0GSU0	ALDH16A1	aldehyde dehydrogenase 16 family member A1	Cytoplasm	enzyme
0.016	-62.5	P97311	MCM6	minichromosome maintenance complex component 6	Nucleus	enzyme
0.017	-58.824	Q9QXK3	COPG2	coatomer protein complex subunit gamma 2	Cytoplasm	transporter
0.017	-58.824	P97855	G3BP1	G3BP stress granule assembly factor 1	Nucleus	enzyme
0.017	-58.824	Q3THK7	GMPS	guanine monophosphate synthase	Nucleus	enzyme
0.017	-58.824	Q3V4D5	NAA10	N(alpha)-acetyltransferase 10, NatA catalytic subunit	Nucleus	enzyme
0.017	-58.824	Q8BK67	RCC2	regulator of chromosome condensation 2	Nucleus	other
0.018	-55.556	V9GXQ2	Gm17087	predicted gene 17087	Other	other
0.018	-55.556	P14206	RPSA	ribosomal protein SA	Cytoplasm	translation regulator
0.019	-52.632	Q9D8N0	EEF1G	eukaryotic translation elongation factor 1 gamma	Cytoplasm	translation regulator
0.019	-52.632	P23116	EIF3A	eukaryotic translation initiation factor 3 subunit A	Cytoplasm	other
0.02	-50	Q80ZX0	SEC24B	SEC24 homolog B, COPII coat complex component	Cytoplasm	transporter
0.02	-50	Q8VIJ6	SFPQ	splicing factor proline and glutamine rich	Nucleus	transcription regulator
0.021	-47.619	Q924C1	XPO5	exportin 5	Nucleus	transporter
0.022	-45.455	P07901	HSP90AA1	heat shock protein 90 alpha family class A member 1	Cytoplasm	enzyme
0.023	-43.478	Q9ERK4	CSE1L	chromosome segregation 1 like	Nucleus	transporter
0.023	-43.478	Q6A0A9	FAM120A	family with sequence similarity 120A	Cytoplasm	other
0.023	-43.478	Q8CFI7	POLR2B	RNA polymerase II subunit B	Nucleus	enzyme
0.023	-43.478	Q99LF4	RTCB	RNA 2',3'-cyclic phosphate and 5'-OH ligase	Cytoplasm	enzyme
0.024	-41.667	P50247	AHCY	adenosylhomocysteinase	Cytoplasm	enzyme
0.024	-41.667	P42932	CCT8	chaperonin containing TCP1 subunit 8	Cytoplasm	enzyme
0.024	-41.667	Q8C3J5	DOCK2	dedicator of cytokinesis 2	Cytoplasm	other
0.024	-41.667	Q6PDI5	ECPAS	Ecm29 proteasome adaptor and scaffold	Cytoplasm	other
0.024	-41.667	P51410	RPL9	ribosomal protein L9	Nucleus	other
0.024	-41.667	O55201	SUPT5H	SPT5 homolog, DSIF elongation factor subunit	Nucleus	transcription regulator
0.026	-38.462	P97351	Rps3a1	ribosomal protein S3A1	Cytoplasm	other
0.026	-38.462	Q3U4W8	USP5	ubiquitin specific peptidase 5	Cytoplasm	peptidase
0.031	-32.258	P80317	CCT6A	chaperonin containing TCP1 subunit 6A	Cytoplasm	other

Table S3

0.031	-32.258	P36916	GNL1	G protein nucleolar 1 (putative)	Nucleus	other
0.031	-32.258	O54692	ZW10	zw10 kinetochore protein	Nucleus	other
0.033	-30.303	S4R2P8	BIRC6	baculoviral IAP repeat containing 6	Cytoplasm	enzyme
0.033	-30.303	E9QAT0	FMR1	fragile X mental retardation 1	Cytoplasm	translation regulator
0.035	-28.571	P62830	RPL23	ribosomal protein L23	Cytoplasm	other
0.035	-28.571	Q9Z1Z2	STRAP	serine/threonine kinase receptor associated protein	Plasma Membrane	other
0.035	-28.571	Q9Z1Q9	VARS	valyl-tRNA synthetase	Cytoplasm	enzyme
0.036	-27.778	O55029	COPB2	coatomer protein complex subunit beta 2	Cytoplasm	transporter
0.036	-27.778	Q9Z2X1	HNRNPF	heterogeneous nuclear ribonucleoprotein F	Nucleus	other
0.036	-27.778	G3UZ48	SYNCRIP	synaptotagmin binding cytoplasmic RNA interacting protein	Nucleus	other
0.038	-26.316	Q01853	VCP	valosin containing protein	Cytoplasm	enzyme
0.04	-25	Q921M3	SF3B3	splicing factor 3b subunit 3	Nucleus	other
0.04	-25	Q3UDE2	TTLL12	tubulin tyrosine ligase like 12	Other	other
0.044	-22.727	P58252	EEF2	eukaryotic translation elongation factor 2	Cytoplasm	translation regulator
0.045	-22.222	Q99LC8	EIF2B1	eukaryotic translation initiation factor 2B subunit alpha	Cytoplasm	translation regulator
0.045	-22.222	P84099	RPL19	ribosomal protein L19	Cytoplasm	other
0.045	-22.222	P13439	UMPS	uridine monophosphate synthetase	Cytoplasm	enzyme
0.047	-21.277	Q8C2Q7	HNRNPH1	heterogeneous nuclear ribonucleoprotein H1	Nucleus	other
0.048	-20.833	Q99KI0	ACO2	aconitase 2	Cytoplasm	enzyme
0.048	-20.833	Q8QZY1	EIF3L	eukaryotic translation initiation factor 3 subunit L	Cytoplasm	other
0.049	-20.408	Q3TW96	UAP1L1	UDP-N-acetylglucosamine pyrophosphorylase 1 like 1	Other	other
0.05	-20	A0A0R4J079	ACBD3	acyl-CoA binding domain containing 3	Cytoplasm	other
0.05	-20	Q3U4X8	LIG1	DNA ligase 1	Nucleus	enzyme
0.05	-20	Q61937	NPM1	nucleophosmin 1	Nucleus	transcription regulator
0.05	-20	Q921F2	TARDBP	TAR DNA binding protein	Nucleus	transcription regulator
0.051	-19.608	Q8BU30	IARS	isoleucyl-tRNA synthetase	Cytoplasm	enzyme
0.052	-19.231	P09920	CSF3	colony stimulating factor 3	Extracellular Space	cytokine
0.053	-18.868	Q8K297	COLGALT1	collagen beta(1-O)galactosyltransferase 1	Cytoplasm	enzyme
0.053	-18.868	Q9D0I9	RARS	arginyl-tRNA synthetase	Cytoplasm	enzyme
0.054	-18.519	A0A0R4J140	CLUH	clustered mitochondria homolog	Cytoplasm	translation regulator
0.059	-16.949	P39054	DNM2	dynamin 2	Plasma Membrane	enzyme
0.059	-16.949	Q8BMJ2	LARS	leucyl-tRNA synthetase	Cytoplasm	enzyme
0.06	-16.667	H3BKN0	NSUN2	NOP2/Sun RNA methyltransferase family member 2	Nucleus	enzyme
0.061	-16.393	E9QB02	MARS	methionyl-tRNA synthetase	Cytoplasm	enzyme

Table S3

0.062	-16.129	P01902	HLA-A	major histocompatibility complex, class I, A	Plasma Membrane	other
0.064	-15.625	Q8BJW6	EIF2A	eukaryotic translation initiation factor 2A	Cytoplasm	translation regulator
0.064	-15.625	Q9CX86	HNRNPA0	heterogeneous nuclear ribonucleoprotein A0	Nucleus	other
0.064	-15.625	Q8BW10	NOB1	NIN1 (RPN12) binding protein 1 homolog	Nucleus	enzyme
0.064	-15.625	P00860	ODC1	ornithine decarboxylase 1	Cytoplasm	enzyme
0.064	-15.625	Q5SWN2	RPA1	replication protein A1	Nucleus	other
0.064	-15.625	Q6P5D8	SMCHD1	structural maintenance of chromosomes flexible hinge domain containing 1	Nucleus	enzyme
0.066	-15.152	P11499	HSP90AB1	heat shock protein 90 alpha family class B member 1	Cytoplasm	enzyme
0.068	-14.706	Q9QZE5	COPG1	coatomer protein complex subunit gamma 1	Cytoplasm	transporter
0.068	-14.706	Q7TMB8	CYFIP1	cytoplasmic FMR1 interacting protein 1	Cytoplasm	other
0.071	-14.085	E9Q3M3	DCTN1	dynactin subunit 1	Cytoplasm	other
0.071	-14.085	Q9CSH3	DIS3	DIS3 homolog, exosome endoribonuclease and 3'-5' exoribonuclease	Nucleus	enzyme
0.071	-14.085	Q921K2	PARP1	poly(ADP-ribose) polymerase 1	Nucleus	enzyme
0.071	-14.085	A0A0A6YX73	PRKAR2A	protein kinase cAMP-dependent type II regulatory subunit alpha	Cytoplasm	kinase
0.071	-14.085	P99026	PSMB4	proteasome subunit beta 4	Cytoplasm	peptidase
0.073	-13.699	Q9Z204	HNRNPC	heterogeneous nuclear ribonucleoprotein C (C1/C2)	Nucleus	other
0.074	-13.514	P60335	PCBP1	poly(rC) binding protein 1	Nucleus	translation regulator
0.076	-13.158	A2A4P0	DHX8	DEAH-box helicase 8	Nucleus	enzyme
0.076	-13.158	P97379	G3BP2	G3BP stress granule assembly factor 2	Cytoplasm	enzyme
0.076	-13.158	Q9EPL8	IPO7	importin 7	Nucleus	transporter
0.076	-13.158	P61358	RPL27	ribosomal protein L27	Cytoplasm	other
0.076	-13.158	Q9DBR1	XRN2	5'-3' exoribonuclease 2	Nucleus	enzyme
0.078	-12.821	ALBU_BOVIN	ALB	albumin	Extracellular Space	transporter
0.078	-12.821	Q8JZQ9	EIF3B	eukaryotic translation initiation factor 3 subunit B	Cytoplasm	translation regulator
0.079	-12.658	G3X9Q3	ARHGAP45	Rho GTPase activating protein 45	Cytoplasm	transporter
0.079	-12.658	P70698	CTPS1	CTP synthase 1	Nucleus	enzyme
0.079	-12.658	Q91Z50	FEN1	flap structure-specific endonuclease 1	Nucleus	enzyme
0.079	-12.658	Q8BKC5	IPO5	importin 5	Nucleus	transporter
0.08	-12.5	P17918	PCNA	proliferating cell nuclear antigen	Nucleus	enzyme
0.08	-12.5	Q9DBC7	PRKAR1A	protein kinase cAMP-dependent type I regulatory subunit alpha	Cytoplasm	kinase
0.081	-12.346	Q5SWU9	ACACA	acetyl-CoA carboxylase alpha	Cytoplasm	enzyme
0.081	-12.346	Q8BFZ3	ACTBL2	actin, beta like 2	Nucleus	other

Table S3

0.081	-12.346	Q9D2V7	CORO7/CORO7-PAM16	coronin 7	Cytoplasm	other
0.081	-12.346	P60229	EIF3E	eukaryotic translation initiation factor 3 subunit E	Cytoplasm	other
0.081	-12.346	Q99MR6	SRRT	serrate, RNA effector molecule	Nucleus	other
0.081	-12.346	Q91W86	VPS11	VPS11, CORVET/HOPS core subunit	Cytoplasm	transporter
0.084	-11.905	O35286	DHX15	DEAH-box helicase 15	Nucleus	enzyme
0.086	-11.628	Q9R190	MTA2	metastasis associated 1 family member 2	Nucleus	transcription regulator
0.092	-10.87	Q91VC3	EIF4A3	eukaryotic translation initiation factor 4A3	Nucleus	enzyme
0.1	-10	A0A1Y7VKY1	Gm11361	ribosomal protein S18 pseudogene	Other	other
0.1	-10	P09405	NCL	nucleolin	Nucleus	other
0.11	-9.091	Q3V117	ACLY	ATP citrate lyase	Cytoplasm	enzyme
0.11	-9.091	E9Q555	RNF213	ring finger protein 213	Cytoplasm	enzyme
0.11	-9.091	Q8BP67	RPL24	ribosomal protein L24	Cytoplasm	other
0.11	-9.091	P32067	SSB	Sjogren syndrome antigen B	Nucleus	enzyme
0.11	-9.091	Q99KC8	VWA5A	von Willebrand factor A domain containing 5A	Nucleus	other
0.11	-9.091	P62806				
0.12	-8.333	P07356	ANXA2	annexin A2	Plasma Membrane	other
0.12	-8.333	A2AIV8	CARD9	caspase recruitment domain family member 9	Cytoplasm	other
0.12	-8.333	G3UY65	CCNB1	cyclin B1	Cytoplasm	kinase
0.12	-8.333	P30285	CDK4	cyclin dependent kinase 4	Nucleus	kinase
0.12	-8.333	F8WHL2	COPA	coatomer protein complex subunit alpha	Cytoplasm	transporter
0.12	-8.333	F6ZFU0	EEF1D	eukaryotic translation elongation factor 1 delta	Cytoplasm	translation regulator
0.12	-8.333	Q9Z0N1	EIF2S3	eukaryotic translation initiation factor 2 subunit gamma	Cytoplasm	translation regulator
0.12	-8.333	Q05D44	EIF5B	eukaryotic translation initiation factor 5B	Cytoplasm	translation regulator
0.12	-8.333	Q64378	FKBP5	FK506 binding protein 5	Nucleus	enzyme
0.12	-8.333	Q8BTZ7	GMPPB	GDP-mannose pyrophosphorylase B	Cytoplasm	enzyme
0.12	-8.333	Q8CI11	GNL3	G protein nucleolar 3	Nucleus	other
0.12	-8.333	Q921F4	HNRNPLL	heterogeneous nuclear ribonucleoprotein L like	Other	other
0.12	-8.333	A0A0G2JFJ6	INTS3	integrator complex subunit 3	Nucleus	other
0.12	-8.333	Z4YJT3	LARP1	La ribonucleoprotein domain family member 1	Cytoplasm	translation regulator
0.12	-8.333	Q05CL8	LARP7	La ribonucleoprotein domain family member 7	Nucleus	other
0.12	-8.333	Q91VH6	MEMO1	mediator of cell motility 1	Cytoplasm	other
0.12	-8.333	Q8BP48	METAP1	methionyl aminopeptidase 1	Cytoplasm	peptidase
0.12	-8.333	Q61753	PHGDH	phosphoglycerate dehydrogenase	Cytoplasm	enzyme
0.12	-8.333	P30412	PPIC	peptidylprolyl isomerase C	Cytoplasm	enzyme
0.12	-8.333	Q8CCF0	PRPF31	pre-mRNA processing factor 31	Nucleus	other
0.12	-8.333	Q91VU7	PUS7	pseudouridylate synthase 7	Nucleus	enzyme

Table S3

0.12	-8.333	A2AFJ1	RBBP7	RB binding protein 7, chromatin remodeling factor	Nucleus	transcription regulator
0.12	-8.333	Q91WC0	SETD3	SET domain containing 3	Nucleus	enzyme
0.12	-8.333	Q61466	SMARCD1	SWI/SNF related, matrix associated, actin dependent regulator of chromatin, subfamily d, member 1	Nucleus	transcription regulator
0.12	-8.333	Q8CG48	SMC2	structural maintenance of chromosomes 2	Nucleus	transporter
0.12	-8.333	F8WIP8	SPP1	secreted phosphoprotein 1	Extracellular Space	cytokine
0.12	-8.333	Q8BYA0	TBCD	tubulin folding cofactor D	Cytoplasm	other
0.12	-8.333	Q9R1X4	TIMELESS	timeless circadian regulator	Nucleus	other
0.12	-8.333	Q5SSZ5	TNS3	tensin 3	Plasma Membrane	phosphatase
0.12	-8.333	Q01320	TOP2A	DNA topoisomerase II alpha	Nucleus	enzyme
0.12	-8.333	Q4VBE8	WDR18	WD repeat domain 18	Nucleus	other
0.12	-8.333	Q9ERF3	WDR61	WD repeat domain 61	Nucleus	other
0.12	-8.333	P14576				
0.12	-8.333	A0A140T8M7				
0.13	-7.692	O54825	BYSL	bystin like	Nucleus	other
0.13	-7.692	Q6ZQ38	CAND1	cullin associated and neddylation dissociated 1	Cytoplasm	transcription regulator
0.13	-7.692	Q8VDP4	CCAR2	cell cycle and apoptosis regulator 2	Cytoplasm	peptidase
0.13	-7.692	Q8BH64	EHD2	EH domain containing 2	Nucleus	other
0.13	-7.692	Q99LH1	GNL2	G protein nucleolar 2	Nucleus	enzyme
0.13	-7.692	O09106	HDAC1	histone deacetylase 1	Nucleus	transcription regulator
0.13	-7.692	Q8VHM5	HNRNPR	heterogeneous nuclear ribonucleoprotein R	Nucleus	other
0.13	-7.692	Q8C052	MAP1S	microtubule associated protein 1S	Cytoplasm	enzyme
0.13	-7.692	Q9JLN9	MTOR	mechanistic target of rapamycin kinase	Nucleus	kinase
0.13	-7.692	Q3UM45	PPP1R7	protein phosphatase 1 regulatory subunit 7	Nucleus	phosphatase
0.13	-7.692	Q9DAW6	PRPF4	pre-mRNA processing factor 4	Nucleus	other
0.13	-7.692	P29351	PTPN6	protein tyrosine phosphatase, non-receptor type 6	Cytoplasm	phosphatase
0.13	-7.692	P47915	Rpl29 (includes others)	ribosomal protein L29	Cytoplasm	other
0.13	-7.692	P62849	RPS24	ribosomal protein S24	Cytoplasm	other
0.13	-7.692	A2AVJ7	Rrbp1	ribosome binding protein 1	Cytoplasm	transporter
0.13	-7.692	Q6PE01	SNRNP40	small nuclear ribonucleoprotein U5 subunit 40	Nucleus	other
0.13	-7.692	Q8BNV1	TRMT2A	tRNA methyltransferase 2 homolog A	Other	kinase
0.13	-7.692	Q8R5H1	USP15	ubiquitin specific peptidase 15	Cytoplasm	peptidase
0.13	-7.692	Q3UVL4	VPS51	VPS51, GARP complex subunit	Cytoplasm	other
0.13	-7.692	Q7CQN4				
0.13	-7.692	O30916				
0.14	-7.143	P09411	PGK1	phosphoglycerate kinase 1	Cytoplasm	kinase
0.14	-7.143	P26516	PSMD7	proteasome 26S subunit, non-ATPase 7	Cytoplasm	other

Table S3

0.14	-7.143	P60122	RUVBL1	RuvB like AAA ATPase 1	Nucleus	transcription regulator
0.15	-6.667	E9PUF7	ARHGEF1	Rho guanine nucleotide exchange factor 1	Cytoplasm	other
0.16	-6.25	O54774	AP3D1	adaptor related protein complex 3 subunit delta 1	Cytoplasm	transporter
0.16	-6.25	Q8R1Q8	DYNC1LI1	dynein cytoplasmic 1 light intermediate chain 1	Cytoplasm	other
0.16	-6.25	P52927	Hmga2	high mobility group AT-hook 2	Nucleus	enzyme
0.16	-6.25	Q9D0E1	HNRNPM	heterogeneous nuclear ribonucleoprotein M	Nucleus	other
0.16	-6.25	Q8CIH5	PLCG2	phospholipase C gamma 2	Cytoplasm	enzyme
0.16	-6.25	P49722	PSMA2	proteasome subunit alpha 2	Cytoplasm	peptidase
0.16	-6.25	Q8BVY0	RSL1D1	ribosomal L1 domain containing 1	Nucleus	other
0.16	-6.25	F8VQC1	SRP72	signal recognition particle 72	Nucleus	kinase
0.17	-5.882	P62827	RAN	RAN, member RAS oncogene family	Nucleus	enzyme
0.17	-5.882	P62918	RPL8	ribosomal protein L8	Cytoplasm	other
0.18	-5.556	Q8K0C9	GMDS	GDP-mannose 4,6-dehydratase	Cytoplasm	enzyme
0.18	-5.556	Q8R2K3	SSBP1	single stranded DNA binding protein 1	Cytoplasm	other
0.19	-5.263	P28271	ACO1	aconitase 1	Cytoplasm	enzyme
0.19	-5.263	P46664	ADSS	adenylosuccinate synthase	Cytoplasm	enzyme
0.19	-5.263	Q9WUM4	CORO1C	coronin 1C	Cytoplasm	other
0.19	-5.263	E9QPI5	PDS5A	PDS5 cohesin associated factor A	Nucleus	other
0.19	-5.263	P62245	RPS15A	ribosomal protein S15a	Cytoplasm	other
0.19	-5.263	P17751	TPI1	triosephosphate isomerase 1	Cytoplasm	enzyme
0.2	-5	Q99LE6	ABCF2	ATP binding cassette subfamily F member 2	Cytoplasm	transporter
0.2	-5	Q99MU3	ADAR	adenosine deaminase, RNA specific	Nucleus	enzyme
0.2	-5	Q60668	HNRNPD	heterogeneous nuclear ribonucleoprotein D	Nucleus	transcription regulator
0.2	-5	Q9CXY6	ILF2	interleukin enhancer binding factor 2	Nucleus	transcription regulator
0.2	-5	Q99LB6	MAT2B	methionine adenosyltransferase 2B	Cytoplasm	enzyme
0.2	-5	Q52KC3	MCM5	minichromosome maintenance complex component 5	Nucleus	enzyme
0.2	-5	Q6PGB6	NAA50	N(alpha)-acetyltransferase 50, NatE catalytic subunit	Cytoplasm	enzyme
0.2	-5	S4R293	NCF1	neutrophil cytosolic factor 1	Cytoplasm	enzyme
0.2	-5	Q9CQ48	NUDCD2	NudC domain containing 2	Cytoplasm	other
0.2	-5	Q8K183	PDXK	pyridoxal kinase	Cytoplasm	kinase
0.2	-5	Q9R0E1	PLOD3	procollagen-lysine,2-oxoglutarate 5-dioxygenase 3	Cytoplasm	enzyme
0.2	-5	B2RXC6	POLR3A	RNA polymerase III subunit A	Nucleus	enzyme
0.2	-5	Q9QUR6	PREP	prolyl endopeptidase	Cytoplasm	peptidase
0.2	-5	O70435	PSMA3	proteasome subunit alpha 3	Cytoplasm	peptidase
0.2	-5	Q9R1P1	PSMB3	proteasome subunit beta 3	Cytoplasm	peptidase
0.2	-5	Q8VH51	RBM39	RNA binding motif protein 39	Nucleus	transcription regulator
0.2	-5	Q9D1R9	Rpl34 (includes others)	ribosomal protein L34	Cytoplasm	other

Table S3

0.2	-5	P60867	RPS20	ribosomal protein S20	Cytoplasm	other
0.2	-5	A8C756	THADA	THADA, armadillo repeat containing	Cytoplasm	other
0.2	-5	G5E870	TRIP12	thyroid hormone receptor interactor 12	Cytoplasm	enzyme
0.21	-4.762	P70170	ABCC9	ATP binding cassette subfamily C member 9	Plasma Membrane	ion channel
0.21	-4.762	F6WMJ3	ARHGEF6	Rac/Cdc42 guanine nucleotide exchange factor 6	Cytoplasm	other
0.21	-4.762	Q4QRL3	CCDC88B	coiled-coil domain containing 88B	Nucleus	enzyme
0.21	-4.762	Q61081	CDC37	cell division cycle 37	Cytoplasm	kinase
0.21	-4.762	Q9Z1Q5	CLIC1	chloride intracellular channel 1	Nucleus	ion channel
0.21	-4.762	O88543	COPS3	COP9 signalosome subunit 3	Cytoplasm	other
0.21	-4.762	O35218	CPSF2	cleavage and polyadenylation specific factor 2	Nucleus	other
0.21	-4.762	Q9CWX9	DDX47	DEAD-box helicase 47	Nucleus	enzyme
0.21	-4.762	Q9D0R4	DDX56	DEAD-box helicase 56	Nucleus	enzyme
0.21	-4.762	Q9QYJ3	DNAJB1	DnaJ heat shock protein family (Hsp40) member B1	Nucleus	transcription regulator
0.21	-4.762	Q6ZWX6	EIF2S1	eukaryotic translation initiation factor 2 subunit alpha	Cytoplasm	translation regulator
0.21	-4.762	Q99JX4	EIF3M	eukaryotic translation initiation factor 3 subunit M	Cytoplasm	other
0.21	-4.762	Q8BHN3	GANAB	glucosidase II alpha subunit	Cytoplasm	enzyme
0.21	-4.762	D3YZ09	Gar1	GAR1 ribonucleoprotein	Nucleus	other
0.21	-4.762	G3UXW9	GPS1	G protein pathway suppressor 1	Nucleus	other
0.21	-4.762	Q3U0V1	KHSRP	KH-type splicing regulatory protein	Nucleus	enzyme
0.21	-4.762	Q6PAR0	KLHDC10	kelch domain containing 10	Nucleus	other
0.21	-4.762	Q60787	LCP2	lymphocyte cytosolic protein 2	Cytoplasm	other
0.21	-4.762	Q05CX5	LUC7L2	LUC7 like 2, pre-mRNA splicing factor	Other	other
0.21	-4.762	O09110	MAP2K3	mitogen-activated protein kinase kinase 3	Cytoplasm	kinase
0.21	-4.762	Q8R3C0	MCMBP	minichromosome maintenance complex binding protein	Nucleus	other
0.21	-4.762	P54276	MSH6	mutS homolog 6	Nucleus	enzyme
0.21	-4.762	Q8K1R7	NEK9	NIMA related kinase 9	Nucleus	kinase
0.21	-4.762	Q9JM14	NT5C	5', 3'-nucleotidase, cytosolic	Cytoplasm	phosphatase
0.21	-4.762	E9Q7G0	NUMA1	nuclear mitotic apparatus protein 1	Nucleus	other
0.21	-4.762	F8WIK5	PLEKHA2	pleckstrin homology domain containing A2	Cytoplasm	other
0.21	-4.762	P33610	PRIM2	DNA primase subunit 2	Nucleus	enzyme
0.21	-4.762	Q9R1P0	PSMA4	proteasome subunit alpha 4	Cytoplasm	peptidase
0.21	-4.762	Q9R1P3	PSMB2	proteasome subunit beta 2	Cytoplasm	peptidase
0.21	-4.762	Q60692	PSMB6	proteasome subunit beta 6	Nucleus	peptidase
0.21	-4.762	Q8BXC0	PTGIS	prostaglandin I2 synthase	Cytoplasm	enzyme
0.21	-4.762	Q64012	RALY	RALY heterogeneous nuclear ribonucleoprotein	Nucleus	transcription regulator
0.21	-4.762	Q9CT10	RANBP3	RAN binding protein 3	Nucleus	other
0.21	-4.762	Q3UI84	RFC4	replication factor C subunit 4	Nucleus	other
0.21	-4.762	P61514	RPL37A	ribosomal protein L37a	Cytoplasm	other

Table S3

0.21	-4.762	P47911	RPL6	ribosomal protein L6	Nucleus	other
0.21	-4.762	P07742	RRM1	ribonucleotide reductase catalytic subunit M1	Nucleus	enzyme
0.21	-4.762	Q99PM9	UCK2	uridine-cytidine kinase 2	Cytoplasm	kinase
0.21	-4.762	S4R1X1	WDR91	WD repeat domain 91	Cytoplasm	other
0.22	-4.545	Q8R010	AIMP2	aminoacyl tRNA synthetase complex interacting multifunctional protein 2	Plasma Membrane	other
0.22	-4.545	P43275	Hist1h1a	histone cluster 1, H1a	Nucleus	other
0.22	-4.545	P68040	RACK1	receptor for activated C kinase 1	Cytoplasm	enzyme
0.22	-4.545	Q6P5B0	RRP12	ribosomal RNA processing 12 homolog	Nucleus	other
0.22	-4.545	P20152	VIM	vimentin	Cytoplasm	other
0.23	-4.348	O35639	ANXA3	annexin A3	Cytoplasm	enzyme
0.23	-4.348	Q91VR5	DDX1	DEAD-box helicase 1	Nucleus	enzyme
0.23	-4.348	P43276	Hist1h1b	histone cluster 1, H1b	Nucleus	other
0.23	-4.348	Q3TXS7	PSMD1	proteasome 26S subunit, non-ATPase 1	Cytoplasm	other
0.23	-4.348	Q6PFB2	RCC1	regulator of chromosome condensation 1	Cytoplasm	other
0.23	-4.348	P32921	WARS	tryptophanyl-tRNA synthetase	Cytoplasm	enzyme
0.24	-4.167	P54822	ADSL	adenylosuccinate lyase	Cytoplasm	enzyme
0.24	-4.167	P10605	CTSB	cathepsin B	Cytoplasm	peptidase
0.24	-4.167	Q61655	DDX19A	DEAD-box helicase 19A	Nucleus	enzyme
0.24	-4.167	P63037	DNAJA1	DnaJ heat shock protein family (Hsp40) member A1	Nucleus	other
0.24	-4.167	Q9WUA2	FARSB	phenylalanyl-tRNA synthetase subunit beta	Cytoplasm	enzyme
0.24	-4.167	P54775	PSMC4	proteasome 26S subunit, ATPase 4	Nucleus	peptidase
0.24	-4.167	Q9CQM8	RPL21	ribosomal protein L21	Cytoplasm	other
0.24	-4.167	P62960	YBX1	Y-box binding protein 1	Nucleus	transcription regulator
0.25	-4	H3BKM0	Ap2b1	adaptor-related protein complex 2, beta 1 subunit	Plasma Membrane	other
0.25	-4	Q922B2	DARS	aspartyl-tRNA synthetase	Cytoplasm	enzyme
0.25	-4	P12265	GUSB	glucuronidase beta	Cytoplasm	enzyme
0.25	-4	Z4YKB8	HP1BP3	heterochromatin protein 1 binding protein 3	Nucleus	other
0.25	-4	Q8VI75	IPO4	importin 4	Nucleus	transporter
0.25	-4	O09043	NAPSA	napsin A aspartic peptidase	Extracellular Space	peptidase
0.25	-4	P46460	NSF	N-ethylmaleimide sensitive factor, vesicle fusing ATPase	Cytoplasm	transporter
0.25	-4	Q64674	SRM	spermidine synthase	Cytoplasm	enzyme
0.25	-4	Q9CPX4				
0.26	-3.846	O08553	DPYSL2	dihydropyrimidinase like 2	Cytoplasm	enzyme
0.26	-3.846	Q91WK2	EIF3H	eukaryotic translation initiation factor 3 subunit H	Cytoplasm	other
0.26	-3.846	E9PVA8	GCN1	GCN1, eIF2 alpha kinase activator homolog	Cytoplasm	translation regulator
0.26	-3.846	P24547	IMPDH2	inosine monophosphate dehydrogenase 2	Cytoplasm	enzyme
0.26	-3.846	Q8VI93	OAS3	2'-5'-oligoadenylate synthetase 3	Cytoplasm	enzyme

Table S3

0.26	-3.846	Q8BH04	PCK2	phosphoenolpyruvate carboxykinase 2, mitochondrial	Cytoplasm	kinase
0.26	-3.846	O88685	PSMC3	proteasome 26S subunit, ATPase 3	Nucleus	enzyme
0.26	-3.846	Q9CZN7	SHMT2	serine hydroxymethyltransferase 2	Cytoplasm	enzyme
0.26	-3.846	G3X8X7	VPS16	VPS16, CORVET/HOPS core subunit	Cytoplasm	transporter
0.27	-3.704	Q8R366	IGSF8	immunoglobulin superfamily member 8	Plasma Membrane	other
0.27	-3.704	P62192	PSMC1	proteasome 26S subunit, ATPase 1	Nucleus	peptidase
0.27	-3.704	O35593	PSMD14	proteasome 26S subunit, non-ATPase 14	Cytoplasm	peptidase
0.27	-3.704	P62082	RPS7	ribosomal protein S7	Cytoplasm	other
0.27	-3.704	Q3UPL0	SEC31A	SEC31 homolog A, COPII coat complex component	Cytoplasm	other
0.28	-3.571	J3QN31	ADSSL1	adenylosuccinate synthase like 1	Cytoplasm	enzyme
0.28	-3.571	P10810	CD14	CD14 molecule	Plasma Membrane	transmembrane receptor
0.28	-3.571	A0A0R4J172	DENND4B	DENN domain containing 4B	Extracellular Space	other
0.28	-3.571	Q9QXB9	DRG2	developmentally regulated GTP binding protein 2	Cytoplasm	other
0.28	-3.571	O88792	F11R	F11 receptor	Plasma Membrane	other
0.28	-3.571	P43274	Hist1h1e	histone cluster 1, H1e	Nucleus	other
0.28	-3.571	P09055	ITGB1	integrin subunit beta 1	Plasma Membrane	transmembrane receptor
0.28	-3.571	Q99K48	NONO	non-POU domain containing octamer binding	Nucleus	transcription regulator
0.28	-3.571	P67984	RPL22	ribosomal protein L22	Cytoplasm	translation regulator
0.28	-3.571	Q5SWD9	TSR1	TSR1, ribosome maturation factor	Nucleus	other
0.29	-3.448	Q9CWJ9	ATIC	5-aminoimidazole-4-carboxamide ribonucleotide formyltransferase/IMP cyclohydrolase	Cytoplasm	enzyme
0.29	-3.448	P36895	BMPR1A	bone morphogenetic protein receptor type 1A	Plasma Membrane	kinase
0.29	-3.448	Q99J95	CDK9	cyclin dependent kinase 9	Nucleus	kinase
0.29	-3.448	Q3UJB9	EDC4	enhancer of mRNA decapping 4	Cytoplasm	other
0.29	-3.448	Q9DCH4	EIF3F	eukaryotic translation initiation factor 3 subunit F	Cytoplasm	translation regulator
0.29	-3.448	P29416	HEXA	hexosaminidase subunit alpha	Cytoplasm	enzyme
0.29	-3.448	Q9BDB7	IFI44L	interferon induced protein 44 like	Nucleus	other
0.29	-3.448	Q91ZX7	LRP1	LDL receptor related protein 1	Plasma Membrane	transmembrane receptor
0.29	-3.448	G3X8Y3	NAA15	N(alpha)-acetyltransferase 15, NatA auxiliary subunit	Nucleus	transcription regulator
0.29	-3.448	Q8K224	NAT10	N-acetyltransferase 10	Nucleus	enzyme

Table S3

0.29	-3.448	Q3TC46	PATL1	PAT1 homolog 1, processing body mRNA decay factor	Cytoplasm	translation regulator
0.29	-3.448	Q9Z2U1	PSMA5	proteasome subunit alpha 5	Cytoplasm	peptidase
0.29	-3.448	P62754	RPS6	ribosomal protein S6	Cytoplasm	other
0.29	-3.448	Q91VH2	SNX9	sorting nexin 9	Cytoplasm	transporter
0.29	-3.448	Q64514	TPP2	tripeptidyl peptidase 2	Cytoplasm	peptidase
0.29	-3.448	P68369	TUBA1A	tubulin alpha 1a	Cytoplasm	other
0.29	-3.448	A0A0A0MQA5	TUBA4A	tubulin alpha 4a	Cytoplasm	other
0.29	-3.448	Q9DCD2	XAB2	XPA binding protein 2	Nucleus	other
0.3	-3.333	P08030	APRT	adenine phosphoribosyltransferase	Cytoplasm	enzyme
0.31	-3.226	B1AQF4	DUSP3	dual specificity phosphatase 3	Cytoplasm	phosphatase
0.31	-3.226	Q3TPJ8	Dync1i2	dynein cytoplasmic 1 intermediate chain 2	Cytoplasm	other
0.31	-3.226	Q99ME9	GTPBP4	GTP binding protein 4	Nucleus	enzyme
0.31	-3.226	Q8VDJ3	HDLBP	high density lipoprotein binding protein	Nucleus	transporter
0.31	-3.226	P70349	HINT1	histidine triad nucleotide binding protein 1	Nucleus	enzyme
0.31	-3.226	A2AFQ0	HUWE1	HECT, UBA and WWE domain containing 1, E3 ubiquitin protein ligase	Nucleus	transcription regulator
0.31	-3.226	Q8R5F7	IFIH1	interferon induced with helicase C domain 1	Nucleus	enzyme
0.31	-3.226	P31938	MAP2K1	mitogen-activated protein kinase kinase 1	Cytoplasm	kinase
0.31	-3.226	O88325	NAGLU	N-acetyl-alpha-glucosaminidase	Cytoplasm	enzyme
0.31	-3.226	Q3UYV9	NCBP1	nuclear cap binding protein subunit 1	Nucleus	other
0.31	-3.226	O35375	NRP2	neuropilin 2	Plasma Membrane	kinase
0.31	-3.226	Q9EQQ9	OGA	O-GlcNAcase	Cytoplasm	enzyme
0.31	-3.226	Q99PG2	OGFR	opioid growth factor receptor	Plasma Membrane	other
0.31	-3.226	Q8VCT3	RNPEP	arginyl aminopeptidase	Cytoplasm	peptidase
0.31	-3.226	P47963	RPL13	ribosomal protein L13	Nucleus	other
0.31	-3.226	P61255	RPL26	ribosomal protein L26	Cytoplasm	other
0.31	-3.226	Q3UMP4	SERBP1	SERPINE1 mRNA binding protein 1	Cytoplasm	other
0.31	-3.226	Q8BKX6	SMG1	SMG1, nonsense mediated mRNA decay associated PI3K related kinase	Cytoplasm	kinase
0.32	-3.125	Q3UZG4	AIMP1	aminoacyl tRNA synthetase complex interacting multifunctional protein 1	Extracellular Space	cytokine
0.32	-3.125	P17426	AP2A1	adaptor related protein complex 2 subunit alpha 1	Cytoplasm	transporter
0.32	-3.125	Q8VDW0	DDX39A	DExD-box helicase 39A	Nucleus	enzyme
0.32	-3.125	A0A087WPL5	DHX9	DExH-box helicase 9	Nucleus	enzyme
0.32	-3.125	Q8BG05	Hnrnpa3	heterogeneous nuclear ribonucleoprotein A3	Nucleus	transporter

Table S3

0.32	-3.125	P97333	NRP1	neuropilin 1	Plasma Membrane	transmembrane receptor
0.33	-3.03	Q641P0	ACTR3B	ARP3 actin related protein 3 homolog B	Cytoplasm	other
0.33	-3.03	Q9EST5	Anp32b	acidic (leucine-rich) nuclear phosphoprotein 32 family, member B	Nucleus	other
0.33	-3.03	A0A1B0GX81	BAG6	BCL2 associated athanogene 6	Nucleus	enzyme
0.33	-3.03	P80313	CCT7	chaperonin containing TCP1 subunit 7	Cytoplasm	other
0.33	-3.03	Q99KK2	CMAS	cytidine monophosphate N-acetylneuraminic acid synthetase	Nucleus	enzyme
0.33	-3.03	H3BJ30	CPSF6	cleavage and polyadenylation specific factor 6	Nucleus	other
0.33	-3.03	Q9CWL8	CTNNBL1	catenin beta like 1	Nucleus	other
0.33	-3.03	Q8K363	DDX18	DEAD-box helicase 18	Nucleus	enzyme
0.33	-3.03	Q6PDL0	DYNC1LI2	dynein cytoplasmic 1 light intermediate chain 2	Cytoplasm	other
0.33	-3.03	P70372	ELAVL1	ELAV like RNA binding protein 1	Cytoplasm	other
0.33	-3.03	O35382	EXOC4	exocyst complex component 4	Cytoplasm	transporter
0.33	-3.03	Q99L27	GMPR2	guanosine monophosphate reductase 2	Cytoplasm	enzyme
0.33	-3.03	Q8CIM8	INTS4	integrator complex subunit 4	Nucleus	other
0.33	-3.03	K22E_HUMAN	KRT2	keratin 2	Cytoplasm	other
0.33	-3.03	Q91YR5	METTL13	methyltransferase like 13	Other	enzyme
0.33	-3.03	Q9CQ65	MTAP	methylthioadenosine phosphorylase	Nucleus	enzyme
0.33	-3.03	F6SMY7	MYCBP2	MYC binding protein 2, E3 ubiquitin protein ligase	Nucleus	enzyme
0.33	-3.03	Q8BJ71	NUP93	nucleoporin 93	Nucleus	other
0.33	-3.03	Q9JLV6	PNKP	polynucleotide kinase 3'-phosphatase	Nucleus	kinase
0.33	-3.03	Q60972	RBBP4	RB binding protein 4, chromatin remodeling factor	Nucleus	enzyme
0.33	-3.03	A0A0G2JGD2	S100A4	S100 calcium binding protein A4	Cytoplasm	other
0.33	-3.03	Q8BRF7	SCFD1	sec1 family domain containing 1	Cytoplasm	transporter
0.33	-3.03	E9Q0Y4	SIPA1	signal-induced proliferation-associated 1	Cytoplasm	other
0.33	-3.03	Q61235	SNTB2	syntrophin beta 2	Plasma Membrane	other
0.33	-3.03	Q9D104	SRP19	signal recognition particle 19	Cytoplasm	other
0.33	-3.03	P0AA28				
0.33	-3.03	P0A1H5				
0.34	-2.941	Q9Z2N8	ACTL6A	actin like 6A	Nucleus	other
0.34	-2.941	O08810	EFTUD2	elongation factor Tu GTP binding domain containing 2	Nucleus	enzyme
0.34	-2.941	Q4FE56	USP9X	ubiquitin specific peptidase 9 X-linked	Plasma Membrane	peptidase
0.35	-2.857	A0A087WNU5	ANK3	ankyrin 3	Plasma Membrane	other

Table S3

0.35	-2.857	Q3TWV4	AP2M1	adaptor related protein complex 2 subunit mu 1	Cytoplasm	transporter
0.35	-2.857	F6QA74	APEX1	apurinic/apyrimidinic endodeoxyribonuclease 1	Nucleus	enzyme
0.35	-2.857	Q9ES28	ARHGEF7	Rho guanine nucleotide exchange factor 7	Cytoplasm	other
0.35	-2.857	P97452	BOP1	block of proliferation 1	Nucleus	other
0.35	-2.857	G3UXB4	CTU2	cytosolic thiouridylase subunit 2	Cytoplasm	other
0.35	-2.857	B9EJR8	DNAAF5	dynein axonemal assembly factor 5	Cytoplasm	other
0.35	-2.857	G3XA17	EIF4G2	eukaryotic translation initiation factor 4 gamma 2	Cytoplasm	translation regulator
0.35	-2.857	Q3UV95	EPB42	erythrocyte membrane protein band 4.2	Plasma Membrane	transporter
0.35	-2.857	A0A0N4SV29	FANCD2	FA complementation group D2	Nucleus	other
0.35	-2.857	Q61555	FBN2	fibrillin 2	Extracellular Space	other
0.35	-2.857	Q99LJ1	FUCA1	alpha-L-fucosidase 1	Cytoplasm	enzyme
0.35	-2.857	P43277	HIST1H1D	histone cluster 1 H1 family member d	Nucleus	other
0.35	-2.857	Q5EBP8	Hnrnpa1	heterogeneous nuclear ribonucleoprotein A1	Nucleus	other
0.35	-2.857	K3W4P2	INTS1	integrator complex subunit 1	Nucleus	other
0.35	-2.857	Q8CHT3	INTS5	integrator complex subunit 5	Nucleus	other
0.35	-2.857	Q8BNW9	KBTBD11	kelch repeat and BTB domain containing 11	Other	other
0.35	-2.857	G3UZA0	KRR1	KRR1, small subunit processome component homolog	Nucleus	other
0.35	-2.857	A0A087WQH1	Lemd1	LEM domain containing 1	Other	other
0.35	-2.857	P63085	MAPK1	mitogen-activated protein kinase 1	Cytoplasm	kinase
0.35	-2.857	Q8K3A9	MEPCE	methylphosphate capping enzyme	Other	enzyme
0.35	-2.857	Q9CQT1	MRI1	methylthioribose-1-phosphate isomerase 1	Cytoplasm	translation regulator
0.35	-2.857	Q8VE10	NAA40	N(alpha)-acetyltransferase 40, NatD catalytic subunit	Other	other
0.35	-2.857	Q9QZQ0	NPAS3	neuronal PAS domain protein 3	Nucleus	transcription regulator
0.35	-2.857	Q9R0E2	PLOD1	procollagen-lysine,2-oxoglutarate 5-dioxygenase 1	Cytoplasm	enzyme
0.35	-2.857	Q9WVF7	POLE	DNA polymerase epsilon, catalytic subunit	Nucleus	enzyme
0.35	-2.857	J3QN19	PRIM1	DNA primase subunit 1	Nucleus	enzyme
0.35	-2.857	O09061	PSMB1	proteasome subunit beta 1	Cytoplasm	peptidase
0.35	-2.857	A0A1D5RLG3	RAB3GAP1	RAB3 GTPase activating protein catalytic subunit 1	Cytoplasm	other
0.35	-2.857	P46061	RANGAP1	Ran GTPase activating protein 1	Nucleus	other
0.35	-2.857	A0A067XG46	RPGR	retinitis pigmentosa GTPase regulator	Cytoplasm	other
0.35	-2.857	Q9CR57	RPL14	ribosomal protein L14	Cytoplasm	other
0.35	-2.857	Q9CYH6	RRS1	ribosome biogenesis regulator homolog	Nucleus	other

Table S3

0.35	-2.857	Q6NZC7	SEC23IP	SEC23 interacting protein	Cytoplasm	other
0.35	-2.857	Q9D554	SF3A3	splicing factor 3a subunit 3	Nucleus	other
0.35	-2.857	Q9JKZ2	SLC5A3	solute carrier family 5 member 3	Plasma Membrane	transporter
0.35	-2.857	B1ARD6	SLFN13	schlafen family member 13	Nucleus	enzyme
0.35	-2.857	Q8CG47	SMC4	structural maintenance of chromosomes 4	Nucleus	transporter
0.35	-2.857	A0A1L1SST5	SPG21	SPG21, maspardin	Plasma Membrane	enzyme
0.35	-2.857	P70279	SURF6	surfeit 6	Nucleus	other
0.35	-2.857	Q8C0V0	TLK1	tousled like kinase 1	Nucleus	kinase
0.35	-2.857	A2A5R0	TP53RK	TP53 regulating kinase	Nucleus	kinase
0.35	-2.857	E9PV45	USP24	ubiquitin specific peptidase 24	Nucleus	peptidase
0.35	-2.857	A0A0R4J114	UTP25	UTP25, small subunit processor component	Nucleus	other
0.35	-2.857	E9Q5W5	ZZEF1	zinc finger ZZ-type and EF-hand domain containing 1	Other	other
0.35	-2.857	A2A4P3				
0.36	-2.778	Q64737	GART	phosphoribosylglycinamide formyltransferase, phosphoribosylglycinamide synthetase, phosphoribosylaminoimidazole synthetase	Cytoplasm	enzyme
0.37	-2.703	O88845	AKAP10	A-kinase anchoring protein 10	Cytoplasm	other
0.37	-2.703	A2AH25	ARHGAP1	Rho GTPase activating protein 1	Cytoplasm	other
0.37	-2.703	Q9WVA3	BUB3	BUB3, mitotic checkpoint protein	Nucleus	other
0.37	-2.703	F6XC25	CC2D1B	coiled-coil and C2 domain containing 1B	Nucleus	transcription regulator
0.37	-2.703	P48678	LMNA	lamin A/C	Nucleus	other
0.37	-2.703	A2ANY6	MDN1	midasin AAA ATPase 1	Nucleus	other
0.37	-2.703	Q9JMH9	MYO18A	myosin XVIIIA	Cytoplasm	other
0.37	-2.703	Q8BGJ5	PTBP1	polypyrimidine tract binding protein 1	Nucleus	enzyme
0.37	-2.703	P35831	PTPN12	protein tyrosine phosphatase, non-receptor type 12	Cytoplasm	phosphatase
0.37	-2.703	Q60770	STXBP3	syntaxin binding protein 3	Plasma Membrane	transporter
0.37	-2.703	A2AN08	UBR4	ubiquitin protein ligase E3 component n-recognin 4	Nucleus	enzyme
0.38	-2.632	Q8VDM4	PSMD2	proteasome 26S subunit, non-ATPase 2	Cytoplasm	other
0.38	-2.632	P62281	RPS11	ribosomal protein S11	Cytoplasm	other
0.39	-2.564	Q5XJY5	ARCN1	archain 1	Cytoplasm	other
0.39	-2.564	Q8BZN6	DOCK10	dedicator of cytokinesis 10	Cytoplasm	other
0.39	-2.564	Q99020	HNRNPAB	heterogeneous nuclear ribonucleoprotein A/B	Nucleus	enzyme
0.39	-2.564	P08905	LYZ	lysozyme	Extracellular Space	enzyme
0.39	-2.564	Q8BZ20	PARP12	poly(ADP-ribose) polymerase family member 12	Nucleus	other
0.39	-2.564	Q8C605	PFKP	phosphofructokinase, platelet	Cytoplasm	kinase
0.39	-2.564	P17742	PPIA	peptidylprolyl isomerase A	Cytoplasm	enzyme
0.39	-2.564	Q9QUM9	PSMA6	proteasome subunit alpha 6	Cytoplasm	peptidase

Table S3

0.39	-2.564	P35980	RPL18	ribosomal protein L18	Cytoplasm	other
0.39	-2.564	P62900	RPL31	ribosomal protein L31	Cytoplasm	other
0.39	-2.564	Q6ZPE2	SBF1	SET binding factor 1	Plasma Membrane	phosphatase
0.39	-2.564	Q9Z1F9	UBA2	ubiquitin like modifier activating enzyme 2	Cytoplasm	enzyme
0.39	-2.564	Q8CCB4	VPS53	VPS53, GARP complex subunit	Cytoplasm	other
0.4	-2.5	O35598	ADAM10	ADAM metallopeptidase domain 10	Plasma Membrane	peptidase
0.4	-2.5	Q3TGU7	PA2G4	proliferation-associated 2G4	Nucleus	transcription regulator
0.4	-2.5	P62843	RPS15	ribosomal protein S15	Cytoplasm	other
0.41	-2.439	A0A0B4J1G1	FCGR2B	Fc fragment of IgG receptor IIb	Plasma Membrane	transmembrane receptor
0.41	-2.439	Q8K1B8	FERMT3	fermitin family member 3	Cytoplasm	enzyme
0.41	-2.439	P61979	HNRNPK	heterogeneous nuclear ribonucleoprotein K	Nucleus	transcription regulator
0.41	-2.439	O55222	ILK	integrin linked kinase	Plasma Membrane	kinase
0.41	-2.439	Q6P5F9	XPO1	exportin 1	Nucleus	transporter
0.42	-2.381	Q9WUM3	CORO1B	coronin 1B	Cytoplasm	other
0.42	-2.381	Q80X98	DHX38	DEAH-box helicase 38	Nucleus	enzyme
0.42	-2.381	P31266	RBPJ	recombination signal binding protein for immunoglobulin kappa J region	Nucleus	transcription regulator
0.42	-2.381	Q6ZWU9	Rps27/Rps27rt	ribosomal protein S27	Other	other
0.42	-2.381	Q6NZD2	SNX1	sorting nexin 1	Cytoplasm	transporter
0.42	-2.381	G3X956	SUPT16H	SPT16 homolog, facilitates chromatin remodeling subunit	Nucleus	transcription regulator
0.43	-2.326	Q99JY9	ACTR3	ARP3 actin related protein 3 homolog	Plasma Membrane	other
0.43	-2.326	Q9R0N0	GALK1	galactokinase 1	Cytoplasm	kinase
0.43	-2.326	Q8VDM6	HNRNPUL1	heterogeneous nuclear ribonucleoprotein U like 1	Nucleus	other
0.43	-2.326	Q9ES52	INPP5D	inositol polyphosphate-5-phosphatase D	Cytoplasm	phosphatase
0.43	-2.326	Q9D071	MMS19	MMS19 homolog, cytosolic iron-sulfur assembly component	Nucleus	transcription regulator
0.43	-2.326	E9QN31	NOP2	NOP2 nucleolar protein	Nucleus	other
0.43	-2.326	P62137	PPP1CA	protein phosphatase 1 catalytic subunit alpha	Cytoplasm	phosphatase
0.43	-2.326	P35979	RPL12	ribosomal protein L12	Nucleus	other
0.43	-2.326	P62852	RPS25	ribosomal protein S25	Cytoplasm	other
0.43	-2.326	Q9WTM5	RUVBL2	RuvB like AAA ATPase 2	Nucleus	transcription regulator
0.43	-2.326	Q8BMA6	SRP68	signal recognition particle 68	Nucleus	other
0.43	-2.326	P52616				
0.44	-2.273	Q8C0C7	FARSA	phenylalanyl-tRNA synthetase subunit alpha	Cytoplasm	enzyme
0.44	-2.273	P14685	PSMD3	proteasome 26S subunit, non-ATPase 3	Cytoplasm	other
0.44	-2.273	P62267	RPS23	ribosomal protein S23	Cytoplasm	translation regulator
0.45	-2.222	F8WGT1	AHCYL2	adenosylhomocysteinase like 2	Other	enzyme

Table S3

0.45	-2.222	H3BKH9	ARHGEF2	Rho/Rac guanine nucleotide exchange factor 2	Cytoplasm	other
0.45	-2.222	Q9JIG7	CCDC22	coiled-coil domain containing 22	Cytoplasm	other
0.45	-2.222	Q3U5Q7	CMPK2	cytidine/uridine monophosphate kinase 2	Cytoplasm	kinase
0.45	-2.222	Q9EPU4	CPSF1	cleavage and polyadenylation specific factor 1	Nucleus	other
0.45	-2.222	Q91YZ2	CTBP2	C-terminal binding protein 2	Nucleus	transcription regulator
0.45	-2.222	P06797	CTSV	cathepsin V	Cytoplasm	peptidase
0.45	-2.222	Q00PI9	HNRNPUL2	heterogeneous nuclear ribonucleoprotein U like 2	Nucleus	other
0.45	-2.222	Q6P9L6	KIF15	kinesin family member 15	Nucleus	other
0.45	-2.222	P35951	LDLR	low density lipoprotein receptor	Plasma Membrane	transporter
0.45	-2.222	A2API5	LRP1B	LDL receptor related protein 1B	Plasma Membrane	transmembrane receptor
0.45	-2.222	P97432	NBR1	NBR1, autophagy cargo receptor	Cytoplasm	other
0.45	-2.222	D3YUM1	NDUFV1	NADH:ubiquinone oxidoreductase core subunit V1	Cytoplasm	enzyme
0.45	-2.222	Q9WTK5	NFKB2	nuclear factor kappa B subunit 2	Nucleus	transcription regulator
0.45	-2.222	Q8BMC4	NOP9	NOP9 nucleolar protein	Nucleus	other
0.45	-2.222	A0A0J9YUD5	NUP205	nucleoporin 205	Nucleus	other
0.45	-2.222	Q8CGY8	OGT	O-linked N-acetylglucosamine (GlcNAc) transferase	Cytoplasm	enzyme
0.45	-2.222	P63087	Ppp1cc	protein phosphatase 1 catalytic subunit gamma	Cytoplasm	phosphatase
0.45	-2.222	Q61171	PRDX2	peroxiredoxin 2	Cytoplasm	enzyme
0.45	-2.222	Q8R574	PRPSAP2	phosphoribosyl pyrophosphate synthetase associated protein 2	Other	other
0.45	-2.222	Q9Z2U0	PSMA7	proteasome subunit alpha 7	Cytoplasm	peptidase
0.45	-2.222	O35295	PURB	purine rich element binding protein B	Nucleus	transcription regulator
0.45	-2.222	Q8K2B3	SDHA	succinate dehydrogenase complex flavoprotein subunit A	Cytoplasm	enzyme
0.45	-2.222	Q91ZW3	SMARCA5	SWI/SNF related, matrix associated, actin dependent regulator of chromatin, subfamily a, member 5	Nucleus	transcription regulator
0.45	-2.222	Q91YJ2	SNX4	sorting nexin 4	Cytoplasm	transporter
0.45	-2.222	Q9D8U8	SNX5	sorting nexin 5	Cytoplasm	transporter
0.45	-2.222	Q61542	STARD3	StAR related lipid transfer domain containing 3	Cytoplasm	transporter
0.45	-2.222	Q5KU39	VPS41	VPS41, HOPS complex subunit	Cytoplasm	transporter
0.45	-2.222	Q8ZPT3				
0.46	-2.174	P15864	HIST1H1C	histone cluster 1 H1 family member c	Nucleus	other
0.47	-2.128	Q61699	HSPH1	heat shock protein family H (Hsp110) member 1	Cytoplasm	other
0.47	-2.128	Q6ZWN5	RPS9	ribosomal protein S9	Cytoplasm	translation regulator

Table S3

0.48	-2.083	Q8K1X4	NCKAP1L	NCK associated protein 1 like	Plasma Membrane	other
0.48	-2.083	Q8BG32	PSMD11	proteasome 26S subunit, non-ATPase 11	Cytoplasm	other
0.48	-2.083	E9PYG6	RASA1	RAS p21 protein activator 1	Cytoplasm	transporter
0.49	-2.041	Q99PT1	ARHGDIA	Rho GDP dissociation inhibitor alpha	Cytoplasm	other
0.49	-2.041	Q9D4H8	CUL2	cullin 2	Nucleus	enzyme
0.49	-2.041	P29758	OAT	ornithine aminotransferase	Cytoplasm	enzyme
0.5	-2	P55264	ADK	adenosine kinase	Nucleus	kinase
0.5	-2	Q9JHZ2	ANKH	ANKH inorganic pyrophosphate transport regulator	Plasma Membrane	transporter
0.5	-2	Q91VK1	BZW2	basic leucine zipper and W2 domains 2	Cytoplasm	translation regulator
0.5	-2	P40237	CD82	CD82 molecule	Plasma Membrane	other
0.5	-2	O89079	COPE	coatomer protein complex subunit epsilon	Cytoplasm	transporter
0.5	-2	Q3TCH7	CUL4A	cullin 4A	Nucleus	other
0.5	-2	Q8K1G9	DHX35	DEAH-box helicase 35	Other	enzyme
0.5	-2	Q8BPU7	ELMO1	engulfment and cell motility 1	Cytoplasm	other
0.5	-2	B7ZNX6	ERBIN	erbb2 interacting protein	Cytoplasm	other
0.5	-2	F7AQX0	EZH2	enhancer of zeste 2 polycomb repressive complex 2 subunit	Nucleus	transcription regulator
0.5	-2	Q7TMC8	FUK	fucokinase	Cytoplasm	kinase
0.5	-2	P70699	GAA	glucosidase alpha, acid	Cytoplasm	enzyme
0.5	-2	Q3U432	GAS7	growth arrest specific 7	Cytoplasm	transcription regulator
0.5	-2	Q9QWL7	KRT17	keratin 17	Cytoplasm	other
0.5	-2	P08249	MDH2	malate dehydrogenase 2	Cytoplasm	enzyme
0.5	-2	B5THE2	MGAM	maltase-glucoamylase	Plasma Membrane	enzyme
0.5	-2	Q8R3N1	NOP14	NOP14 nucleolar protein	Nucleus	other
0.5	-2	Q6P9R2	OXSR1	oxidative stress responsive 1	Nucleus	kinase
0.5	-2	P70268	PKN1	protein kinase N1	Cytoplasm	kinase
0.5	-2	P35456	PLAUR	plasminogen activator, urokinase receptor	Plasma Membrane	transmembrane receptor
0.5	-2	Q9DC61	PMPCA	peptidase, mitochondrial processing alpha subunit	Cytoplasm	peptidase
0.5	-2	P97760	POLR2C	RNA polymerase II subunit C	Nucleus	enzyme
0.5	-2	Q6ZWV7	RPL35	ribosomal protein L35	Cytoplasm	other
0.5	-2	Q91WM3	RRP9	ribosomal RNA processing 9, U3 small nucleolar RNA binding protein	Nucleus	other
0.5	-2	Q6NZR5	SKIV2L	Ski2 like RNA helicase	Nucleus	enzyme
0.5	-2	O70551	SRPK1	SRSF protein kinase 1	Nucleus	kinase
0.5	-2	CAS1_BOVIN				
0.51	-1.961	P45376	AKR1B1	aldo-keto reductase family 1 member B	Cytoplasm	enzyme
0.51	-1.961	Q9CZ30	OLA1	Obg like ATPase 1	Cytoplasm	enzyme
0.51	-1.961	P0A1X0				
0.52	-1.923	Q8JZV7	AMDHD2	amidohydrolase domain containing 2	Nucleus	enzyme

Table S3

0.52	-1.923	Q5SVG4	AP1B1	adaptor related protein complex 1 subunit beta 1	Cytoplasm	transporter
0.52	-1.923	P01887	B2M	beta-2-microglobulin	Plasma Membrane	transmembrane receptor
0.52	-1.923	A0A0U1RNK7	DOCK7	dedicator of cytokinesis 7	Plasma Membrane	other
0.52	-1.923	Q9CZX0	ELP3	elongator acetyltransferase complex subunit 3	Nucleus	enzyme
0.52	-1.923	P16110	LGALS3	galectin 3	Extracellular Space	other
0.52	-1.923	P62315	SNRPD1	small nuclear ribonucleoprotein D1 polypeptide	Nucleus	other
0.52	-1.923	A0A1B0GR11	TALDO1	transaldolase 1	Cytoplasm	enzyme
0.52	-1.923	P35123	USP4	ubiquitin specific peptidase 4	Nucleus	peptidase
0.54	-1.852	A0A0N4SUH4	PUM3	pumilio RNA binding family member 3	Nucleus	other
0.54	-1.852	P62301	RPS13	ribosomal protein S13	Cytoplasm	other
0.55	-1.818	A0A1L1SV25	ACTN4	actinin alpha 4	Cytoplasm	transcription regulator
0.56	-1.786	Q8R5C5	ACTR1B	ARP1 actin related protein 1 homolog B	Cytoplasm	other
0.56	-1.786	P28474	ADH5	alcohol dehydrogenase 5 (class III), chi polypeptide	Cytoplasm	enzyme
0.56	-1.786	M0QWP1	AGRN	agrin	Plasma Membrane	other
0.56	-1.786	A0A0R4J107	APEH	acylaminoacyl-peptide hydrolase	Cytoplasm	peptidase
0.56	-1.786	E9QMX7	ARHGAP30	Rho GTPase activating protein 30	Cytoplasm	other
0.56	-1.786	O54984	ASNA1	arsA arsenite transporter, ATP-binding, homolog 1 (bacterial)	Nucleus	transporter
0.56	-1.786	Q99LC2	CSTF1	cleavage stimulation factor subunit 1	Nucleus	other
0.56	-1.786	P43346	DCK	deoxycytidine kinase	Nucleus	kinase
0.56	-1.786	Q8VHK9	DHX36	DEAH-box helicase 36	Cytoplasm	enzyme
0.56	-1.786	Q9Z2W0	DNPEP	aspartyl aminopeptidase	Cytoplasm	peptidase
0.56	-1.786	P59325	EIF5	eukaryotic translation initiation factor 5	Cytoplasm	translation regulator
0.56	-1.786	Q00612	G6PD	glucose-6-phosphate dehydrogenase	Cytoplasm	enzyme
0.56	-1.786	Q6ZQ88	KDM1A	lysine demethylase 1A	Nucleus	enzyme
0.56	-1.786	F6RJV6	LANCL2	LanC like 2	Plasma Membrane	other
0.56	-1.786	P49138	MAPKAPK2	mitogen-activated protein kinase-activated protein kinase 2	Nucleus	kinase
0.56	-1.786	P34884	MIF	macrophage migration inhibitory factor	Extracellular Space	cytokine
0.56	-1.786	Q8VEJ4	NLE1	notchless homolog 1	Nucleus	enzyme
0.56	-1.786	Q11011	NPEPPS	aminopeptidase puromycin sensitive	Cytoplasm	peptidase
0.56	-1.786	Q91V89	PPP2R5D	protein phosphatase 2 regulatory subunit B'delta	Nucleus	phosphatase
0.56	-1.786	Q9JK23	PSMG1	proteasome assembly chaperone 1	Plasma Membrane	other

Table S3

0.56	-1.786	O08604	Raet1c	retinoic acid early transcript gamma	Plasma Membrane	other
0.56	-1.786	Q9JJF3	RIOX1	ribosomal oxygenase 1	Nucleus	enzyme
0.56	-1.786	P11157	RRM2	ribonucleotide reductase regulatory subunit M2	Nucleus	enzyme
0.56	-1.786	Q9R1T2	SAE1	SUMO1 activating enzyme subunit 1	Cytoplasm	enzyme
0.56	-1.786	Q60710	SAMHD1	SAM and HD domain containing deoxynucleoside triphosphate triphosphohydrolase 1	Nucleus	enzyme
0.56	-1.786	O35988	SDC4	syndecan 4	Plasma Membrane	other
0.56	-1.786	Q3UKJ7	SMU1	SMU1, DNA replication regulator and spliceosomal factor	Nucleus	other
0.56	-1.786	P27048	SNRPB	small nuclear ribonucleoprotein polypeptides B and B1	Nucleus	other
0.56	-1.786	Q9WVA4	TAGLN2	transgelin 2	Cytoplasm	other
0.56	-1.786	Q80XC2	TRMT61A	tRNA methyltransferase 61A	Nucleus	enzyme
0.56	-1.786	Q9WUP7	UCHL5	ubiquitin C-terminal hydrolase L5	Cytoplasm	peptidase
0.56	-1.786	Q8K4P0	WDR33	WD repeat domain 33	Nucleus	other
0.57	-1.754	Q8K3W0	BABAM2	BRISC and BRCA1 A complex member 2	Cytoplasm	other
0.57	-1.754	Q8JZK9	HMGCS1	3-hydroxy-3-methylglutaryl-CoA synthase 1	Cytoplasm	enzyme
0.57	-1.754	Q8K2Z4	NCAPD2	non-SMC condensin I complex subunit D2	Nucleus	other
0.57	-1.754	Q8R0W6	NDFIP1	Nedd4 family interacting protein 1	Cytoplasm	other
0.57	-1.754	Q9CRB2	NHP2	NHP2 ribonucleoprotein	Nucleus	other
0.57	-1.754	P62141	PPP1CB	protein phosphatase 1 catalytic subunit beta	Cytoplasm	phosphatase
0.57	-1.754	E9Q2M9	WDFY4	WDFY family member 4	Other	other
0.58	-1.724	Q8K482	EMILIN2	elastin microfibril interfacer 2	Extracellular Space	other
0.58	-1.724	Q60854	SERPINB6	serpin family B member 6	Cytoplasm	other
0.61	-1.639	P17427	AP2A2	adaptor related protein complex 2 subunit alpha 2	Cytoplasm	transporter
0.61	-1.639	A0A140T8I9	PI4KA	phosphatidylinositol 4-kinase alpha	Cytoplasm	kinase
0.61	-1.639	P83882	Rpl36a	ribosomal protein L36A	Cytoplasm	other
0.62	-1.613	Q99KN1	ARRDC1	arrestin domain containing 1	Cytoplasm	other
0.62	-1.613	Q64261	CDK6	cyclin dependent kinase 6	Nucleus	kinase
0.62	-1.613	A0A0U1RP94	CSNK1G1	casein kinase 1 gamma 1	Cytoplasm	kinase
0.62	-1.613	Q3U1J4	DDB1	damage specific DNA binding protein 1	Nucleus	other
0.62	-1.613	A0A0A0MQM0	EIF5A	eukaryotic translation initiation factor 5A	Cytoplasm	translation regulator
0.62	-1.613	Q7TS64	GRK2	G protein-coupled receptor kinase 2	Cytoplasm	kinase
0.62	-1.613	Q8K2V6	IPO11	importin 11	Nucleus	transporter
0.62	-1.613	Q9D2Y4	MLKL	mixed lineage kinase domain like pseudokinase	Cytoplasm	kinase
0.62	-1.613	Q9JKX6	NUDT5	nudix hydrolase 5	Cytoplasm	phosphatase

Table S3

0.62	-1.613	Q62422	OSTF1	osteoclast stimulating factor 1	Nucleus	transcription regulator
0.62	-1.613	Q9DBD5	PELP1	proline, glutamate and leucine rich protein 1	Nucleus	other
0.62	-1.613	P68181	PRKACB	protein kinase cAMP-activated catalytic subunit beta	Cytoplasm	kinase
0.62	-1.613	Q5SV02	RAD50	RAD50 double strand break repair protein	Nucleus	enzyme
0.62	-1.613	P63325	RPS10	ribosomal protein S10	Cytoplasm	other
0.62	-1.613	P98083	SHC1	SHC adaptor protein 1	Cytoplasm	other
0.62	-1.613	A2AR26	SLC2A6	solute carrier family 2 member 6	Plasma Membrane	transporter
0.62	-1.613	Q64704	STX3	syntaxin 3	Plasma Membrane	transporter
0.65	-1.538	G3X977	ITIH2	inter-alpha-trypsin inhibitor heavy chain 2	Extracellular Space	other
0.67	-1.493	Q5SQ20	PES1	pescadillo ribosomal biogenesis factor 1	Nucleus	other
0.7	-1.429	Q8BK64	AHSA1	activator of HSP90 ATPase activity 1	Cytoplasm	other
0.7	-1.429	A0A0J9YU62	CTBP1	C-terminal binding protein 1	Nucleus	enzyme
0.7	-1.429	Q569Z5	DDX46	DEAD-box helicase 46	Nucleus	enzyme
0.7	-1.429	Q9JHU9	ISYNA1	inositol-3-phosphate synthase 1	Cytoplasm	enzyme
0.71	-1.408	Q8R050	GSPT1	G1 to S phase transition 1	Cytoplasm	translation regulator
0.73	-1.37	P47754	CAPZA2	capping actin protein of muscle Z-line subunit alpha 2	Cytoplasm	other
0.73	-1.37	Q9Z1N5	DDX39B	DExD-box helicase 39B	Nucleus	enzyme
0.73	-1.37	Q8BWU5	OSGEP	O-sialoglycoprotein endopeptidase	Nucleus	peptidase
0.73	-1.37	Q8CAS9	PARP9	poly(ADP-ribose) polymerase family member 9	Nucleus	enzyme
0.73	-1.37	Q8CIH9	PPAT	phosphoribosyl pyrophosphate amidotransferase	Cytoplasm	enzyme
0.52	1	Q921H8	ACAA1	acetyl-CoA acyltransferase 1	Cytoplasm	enzyme
0.81	1	Q8QZT1	ACAT1	acetyl-CoA acetyltransferase 1	Cytoplasm	enzyme
0.56	1	P97822	Anp32e	acidic (leucine-rich) nuclear phosphoprotein 32 family, member E	Cytoplasm	other
0.65	1	Q6DFX2	ANTXR2	ANTXR cell adhesion molecule 2	Plasma Membrane	transmembrane receptor
0.62	1	Q8CBB7	AP1G1	adaptor related protein complex 1 subunit gamma 1	Cytoplasm	transporter
0.52	1	Q9Z1T1	AP3B1	adaptor related protein complex 3 subunit beta 1	Plasma Membrane	transporter
0.54	1	Q61599	ARHGDIB	Rho GDP dissociation inhibitor beta	Cytoplasm	enzyme
0.56	1	D3YXG6	ARPC2	actin related protein 2/3 complex subunit 2	Cytoplasm	other
0.65	1	P59999	ARPC4	actin related protein 2/3 complex subunit 4	Cytoplasm	other
0.81	1	D6REV1	ASXL2	ASXL transcriptional regulator 2	Extracellular Space	other
0.56	1	Q9CPX6	ATG3	autophagy related 3	Cytoplasm	enzyme
0.81	1	Q9D906	ATG7	autophagy related 7	Cytoplasm	enzyme

Table S3

0.81	1	Q9CYN9	ATP6AP2	ATPase H+ transporting accessory protein 2	Cytoplasm	transporter
0.56	1	P01027	C3	complement C3	Extracellular Space	peptidase
0.81	1	G3X8U3	C9orf64	chromosome 9 open reading frame 64	Other	other
0.65	1	Q91YS8	CAMK1	calcium/calmodulin dependent protein kinase I	Cytoplasm	kinase
0.42	1	P40124	CAP1	cyclase associated actin cytoskeleton regulatory protein 1	Plasma Membrane	other
0.81	1	Q8CIS0	CARD11	caspase recruitment domain family member 11	Cytoplasm	kinase
0.81	1	P21855	CD72	CD72 molecule	Plasma Membrane	transmembrane receptor
0.46	1	P60766	Cdc42	cell division cycle 42	Plasma Membrane	enzyme
0.81	1	Q8BT07	CEP55	centrosomal protein 55	Cytoplasm	other
0.65	1	Q9DBC3	CMTR1	cap methyltransferase 1	Nucleus	enzyme
0.54	1	Q9JIF7	COPB1	coatomer protein complex subunit beta 1	Cytoplasm	transporter
0.81	1	P47199	CRYZ	crystallin zeta	Cytoplasm	enzyme
0.57	1	Q9CZU6	CS	citrate synthase	Cytoplasm	enzyme
0.81	1	P26231	CTNNA1	catenin alpha 1	Plasma Membrane	other
0.65	1	P16675	CTSA	cathepsin A	Cytoplasm	peptidase
0.81	1	Q3UGB5	Dazap1	DAZ associated protein 1	Cytoplasm	other
0.81	1	Q8BMF4	DLAT	dihydrolipoamide S-acetyltransferase	Cytoplasm	enzyme
0.81	1	O08749	DLD	dihydrolipoamide dehydrogenase	Cytoplasm	enzyme
0.49	1	E9PUD2	DNM1L	dynamin 1 like	Cytoplasm	enzyme
0.81	1	Q5NCQ5	DPH1	diphthamide biosynthesis 1	Cytoplasm	other
0.56	1	Q99KK7	DPP3	dipeptidyl peptidase 3	Cytoplasm	peptidase
0.81	1	Q8C0D5	EFL1	elongation factor like GTPase 1	Cytoplasm	translation regulator
0.81	1	B1AUN2	EIF2B3	eukaryotic translation initiation factor 2B subunit gamma	Cytoplasm	other
0.65	1	P63073	EIF4E	eukaryotic translation initiation factor 4E	Cytoplasm	translation regulator
0.81	1	Q80Y81	ELAC2	elaC ribonuclease Z 2	Nucleus	enzyme
0.33	1	P17182	ENO1	enolase 1	Cytoplasm	enzyme
0.81	1	Q9CRA8	EXOSC5	exosome component 5	Nucleus	enzyme
0.56	1	P26040	EZR	ezrin	Plasma Membrane	other
0.57	1	Q8R1F1	FAM129B	family with sequence similarity 129 member B	Cytoplasm	transcription regulator
0.56	1	Q80VA0	GALNT7	polypeptide N-acetylgalactosaminyltransferase 7	Cytoplasm	enzyme
0.49	1	P16858	GAPDH	glyceraldehyde-3-phosphate dehydrogenase	Cytoplasm	enzyme
0.48	1	Q9CZD3	GARS	glycyl-tRNA synthetase	Cytoplasm	enzyme
0.56	1	Q8BGZ6	GLA	galactosidase alpha	Cytoplasm	enzyme
0.81	1	Q9DCZ1	GMPR	guanosine monophosphate reductase	Cytoplasm	enzyme

Table S3

0.48	1	P08752	GNAI2	G protein subunit alpha i2	Plasma Membrane	enzyme
0.56	1	P62874	GNB1	G protein subunit beta 1	Plasma Membrane	enzyme
0.56	1	Q9CR60	GOLT1B	golgi transport 1B	Cytoplasm	other
0.81	1	Q3ULJ0	GPD1L	glycerol-3-phosphate dehydrogenase 1 like	Cytoplasm	enzyme
0.81	1	Q91Z53	GRHPR	glyoxylate and hydroxypyruvate reductase	Cytoplasm	enzyme
0.81	1	P28798	GRN	granulin precursor	Extracellular Space	growth factor
0.55	1	Q9D8T2	GSDMD	gasdermin D	Extracellular Space	other
0.81	1	GELS_HUMAN	GSN	gelsolin	Extracellular Space	other
0.81	1	B1AUX2	HCFC1	host cell factor C1	Nucleus	transcription regulator
0.65	1	G3X9B1	HEATR1	HEAT repeat containing 1	Nucleus	other
0.57	1	A2AS03	HELZ2	helicase with zinc finger 2	Nucleus	transcription regulator
0.51	1	P01900	HLA-A	major histocompatibility complex, class I, A	Plasma Membrane	other
0.57	1	P22907	HMBS	hydroxymethylbilane synthase	Cytoplasm	enzyme
0.81	1	O88569	HNRNPA2B1	heterogeneous nuclear ribonucleoprotein A2/B1	Nucleus	other
0.49	1	Q8VEK3	HNRNPU	heterogeneous nuclear ribonucleoprotein U	Nucleus	transporter
0.65	1	P00493	HPRT1	hypoxanthine phosphoribosyltransferase 1	Cytoplasm	enzyme
0.81	1	P51660	HSD17B4	hydroxysteroid 17-beta dehydrogenase 4	Cytoplasm	enzyme
0.81	1	G3X9H5	HTT	huntingtin	Cytoplasm	transcription regulator
0.55	1	Q9JKR6	HYOU1	hypoxia up-regulated 1	Cytoplasm	other
0.58	1	Q07113	IGF2R	insulin like growth factor 2 receptor	Plasma Membrane	transmembrane receptor
0.65	1	Q0GNC1	INF2	inverted formin, FH2 and WH2 domain containing	Cytoplasm	other
0.81	1	A0A0R4J0E4	INTS7	integrator complex subunit 7	Nucleus	other
0.81	1	Q8K0C1	IPO13	importin 13	Nucleus	transporter
0.48	1	Q9JKF1	IQGAP1	IQ motif containing GTPase activating protein 1	Cytoplasm	other
0.48	1	Q792F9	ITGA4	integrin subunit alpha 4	Plasma Membrane	transmembrane receptor
0.81	1	P26011	ITGB7	integrin subunit beta 7	Plasma Membrane	transmembrane receptor
0.65	1	O35345	KPNA6	karyopherin subunit alpha 6	Nucleus	transporter
0.36	1	K2C1_HUMAN	KRT1	keratin 1	Cytoplasm	other
0.81	1	Q9Z2K1	KRT16	keratin 16	Cytoplasm	other
0.81	1	P50446	KRT6B	keratin 6B	Cytoplasm	other
0.57	1	P11438	LAMP1	lysosomal associated membrane protein 1	Plasma Membrane	other

Table S3

0.56	1	Q8BYR1	LCMT2	leucine carboxyl methyltransferase 2	Cytoplasm	enzyme
0.56	1	P06151	LDHA	lactate dehydrogenase A	Cytoplasm	enzyme
0.56	1	A0A0R4J0W6	LRRC40	leucine rich repeat containing 40	Nucleus	other
0.56	1	Q8R4U7	LUZP1	leucine zipper protein 1	Nucleus	other
0.65	1	P14152	MDH1	malate dehydrogenase 1	Cytoplasm	enzyme
0.65	1	B7ZCL8	MPP1	membrane palmitoylated protein 1	Plasma Membrane	kinase
0.81	1	Q99JF5	MVD	mevalonate diphosphate decarboxylase	Cytoplasm	enzyme
0.81	1	A0A0R4J0G3	NIPAL2	NIPA like domain containing 2	Other	other
0.54	1	O70310	NMT1	N-myristoyltransferase 1	Cytoplasm	enzyme
0.58	1	Q8BHY2	NOC4L	nucleolar complex associated 4 homolog	Nucleus	transcription regulator
0.54	1	Q6DFW4	NOP58	NOP58 ribonucleoprotein	Nucleus	enzyme
0.81	1	Q9CZ44	NSFL1C	NSFL1 cofactor	Cytoplasm	other
0.81	1	A0A0A6YWF9	NTRK3	neurotrophic receptor tyrosine kinase 3	Plasma Membrane	kinase
0.81	1	O35685	NUDC	nuclear distribution C, dynein complex regulator	Cytoplasm	other
0.56	1	Q8R480	NUP85	nucleoporin 85	Cytoplasm	other
0.81	1	P61971	NUTF2	nuclear transport factor 2	Nucleus	transporter
0.81	1	H7BX01	OPA1	OPA1, mitochondrial dynamin like GTPase	Cytoplasm	enzyme
0.49	1	Q3UJQ9	OXCT1	3-oxoacid CoA-transferase 1	Cytoplasm	enzyme
0.4	1	P29341	PABPC1	poly(A) binding protein cytoplasmic 1	Cytoplasm	translation regulator
0.81	1	P63005	PAFAH1B1	platelet activating factor acetylhydrolase 1b regulatory subunit 1	Cytoplasm	enzyme
0.65	1	Q9DCE5	PAK1IP1	PAK1 interacting protein 1	Nucleus	other
0.81	1	Q3U4S0	PANK2	pantothenate kinase 2	Cytoplasm	kinase
0.57	1	Q2EMV9	PARP14	poly(ADP-ribose) polymerase family member 14	Cytoplasm	enzyme
0.51	1	P12815	PDCD6	programmed cell death 6	Cytoplasm	other
0.52	1	Q9WU78	PDCD6IP	programmed cell death 6 interacting protein	Cytoplasm	other
0.81	1	Q3TIU4	PDE12	phosphodiesterase 12	Cytoplasm	enzyme
0.65	1	Q9CQ60	PGLS	6-phosphogluconolactonase	Cytoplasm	enzyme
0.56	1	Q9D0F9	PGM1	phosphoglucomutase 1	Cytoplasm	enzyme
0.81	1	Q8VD65	PIK3R4	phosphoinositide-3-kinase regulatory subunit 4	Cytoplasm	kinase
0.79	1	F8WI18	PIP5K1A	phosphatidylinositol-4-phosphate 5-kinase type 1 alpha	Cytoplasm	kinase
0.55	1	Q9DBX5	PLA2G4A	phospholipase A2 group IVA	Cytoplasm	enzyme
0.81	1	P27612	PLAA	phospholipase A2 activating protein	Cytoplasm	other
0.79	1	P70700	POLR1B	RNA polymerase I subunit B	Nucleus	enzyme
0.56	1	A0A0J9YVG0	PPM1G	protein phosphatase, Mg2+/Mn2+ dependent 1G	Nucleus	phosphatase
0.5	1	P63330	PPP2CA	protein phosphatase 2 catalytic subunit alpha	Cytoplasm	phosphatase
0.55	1	Q76MZ3	PPP2R1A	protein phosphatase 2 scaffold subunit Aalpha	Cytoplasm	phosphatase

Table S3

0.56	1	P97470	PPP4C	protein phosphatase 4 catalytic subunit	Cytoplasm	phosphatase
0.81	1	Q60676	PPP5C	protein phosphatase 5 catalytic subunit	Nucleus	phosphatase
0.56	1	O54950	PRKAG1	protein kinase AMP-activated non-catalytic subunit gamma 1	Nucleus	kinase
0.58	1	Q99KP6	PRPF19	pre-mRNA processing factor 19	Nucleus	enzyme
0.81	1	P28063	PSMB8	proteasome subunit beta 8	Cytoplasm	peptidase
0.4	1	Q8BVQ9	PSMC2	proteasome 26S subunit, ATPase 2	Nucleus	peptidase
0.47	1	P62334	PSMC6	proteasome 26S subunit, ATPase 6	Nucleus	peptidase
0.65	1	Q9CX56	PSMD8	proteasome 26S subunit, non-ATPase 8	Cytoplasm	other
0.81	1	P22437	PTGS1	prostaglandin-endoperoxide synthase 1	Cytoplasm	enzyme
0.81	1	Q61550	RAD21	RAD21 cohesin complex component	Nucleus	transcription regulator
0.52	1	Q9JIW9	RALB	RAS like proto-oncogene B	Cytoplasm	enzyme
0.56	1	Q6A0D4	RFTN1	raftlin, lipid raft linker 1	Plasma Membrane	other
0.65	1	Q9D0L8	RNMT	RNA guanine-7 methyltransferase	Nucleus	enzyme
0.33	1	P27659	RPL3	ribosomal protein L3	Nucleus	other
0.3	1	Q9D8E6	RPL4	ribosomal protein L4	Cytoplasm	enzyme
0.36	1	P14148	RPL7	ribosomal protein L7	Nucleus	transcription regulator
0.46	1	P62855	RPS26	ribosomal protein S26	Cytoplasm	other
0.46	1	P62908	RPS3	ribosomal protein S3	Cytoplasm	enzyme
0.5	1	P62242	RPS8	ribosomal protein S8	Cytoplasm	other
0.65	1	Q8R550	SH3KBP1	SH3 domain containing kinase binding protein 1	Cytoplasm	other
0.81	1	Q8VI23	SLC12A8	solute carrier family 12 member 8	Other	transporter
0.56	1	Q80TR4	SLIT1	slit guidance ligand 1	Extracellular Space	other
0.58	1	Q9CWK8	SNX2	sorting nexin 2	Cytoplasm	transporter
0.56	1	Q64105	SPR	sepiapterin reductase	Cytoplasm	enzyme
0.43	1	Q08943	SSRP1	structure specific recognition protein 1	Nucleus	transcription regulator
0.81	1	Q64692	ST8SIA4	ST8 alpha-N-acetyl-neuraminide alpha-2,8-sialyltransferase 4	Cytoplasm	enzyme
0.65	1	Q60864	STIP1	stress induced phosphoprotein 1	Cytoplasm	other
0.65	1	Q3TAA7	STK11IP	serine/threonine kinase 11 interacting protein	Cytoplasm	other
0.81	1	B2RQS1	STRN3	striatin 3	Nucleus	transcription regulator
0.55	1	P70452	STX4	syntaxin 4	Plasma Membrane	transporter
0.57	1	P30548	TACR1	tachykinin receptor 1	Plasma Membrane	G-protein coupled receptor
0.55	1	Q9D0R2	TARS	threonyl-tRNA synthetase	Nucleus	enzyme
0.56	1	Q80YX1	TNC	tenascin C	Extracellular Space	other

Table S3

0.65	1	Q60769	TNFAIP3	TNF alpha induced protein 3	Nucleus	enzyme
0.61	1	Q6P2B1	TNPO3	transportin 3	Cytoplasm	other
0.65	1	Q91XB0	TREX1	three prime repair exonuclease 1	Nucleus	enzyme
0.65	1	Q99PN3	TRIM26	tripartite motif containing 26	Cytoplasm	other
0.65	1	Q3TX08	TRMT1	tRNA methyltransferase 1	Extracellular Space	enzyme
0.57	1	Q9D0C4	TRMT5	tRNA methyltransferase 5	Cytoplasm	other
0.81	1	P23591	TSTA3	tissue specific transplantation antigen P35B	Plasma Membrane	enzyme
0.81	1	Q8C878	UBA3	ubiquitin like modifier activating enzyme 3	Cytoplasm	enzyme
0.56	1	Q9DBK7	UBA7	ubiquitin like modifier activating enzyme 7	Cytoplasm	enzyme
0.56	1	P61089	UBE2N	ubiquitin conjugating enzyme E2 N	Cytoplasm	enzyme
0.65	1	F8VPX1	USP7	ubiquitin specific peptidase 7	Nucleus	peptidase
0.61	1	Q5SSI6	UTP18	UTP18, small subunit processome component	Nucleus	other
0.65	1	Q8R2N2	UTP4	UTP4, small subunit processome component	Nucleus	other
0.62	1	Q8R307	VPS18	VPS18, CORVET/HOPS core subunit	Cytoplasm	transporter
0.81	1	D3YYD5	VPS29	VPS29, retromer complex component	Cytoplasm	transporter
0.81	1	Q80X41	VRK1	vaccinia related kinase 1	Nucleus	kinase
0.56	1	O88342	WDR1	WD repeat domain 1	Extracellular Space	other
0.65	1	Q3TAQ9	WDR36	WD repeat domain 36	Extracellular Space	other
0.65	1	Q6ZQL4	WDR43	WD repeat domain 43	Nucleus	other
0.65	1	Q9EPK7	XPO7	exportin 7	Nucleus	transporter
0.81	1	A2A7S7	YARS	tyrosyl-tRNA synthetase	Cytoplasm	enzyme
0.81	1	CAS2_BOVIN				
0.81	1	KRA61_SHEEP				
0.38	1	P10853				
0.81	1	E9PY39				
0.34	1.1	P48036	ANXA5	annexin A5	Plasma Membrane	transporter
0.47	1.1	Q07076	ANXA7	annexin A7	Plasma Membrane	ion channel
0.52	1.1	A2AQA7	AQR	aquarius intron-binding spliceosomal factor	Nucleus	other
0.58	1.1	A0A0N4SW07	ATP6V1E1	ATPase H+ transporting V1 subunit E1	Cytoplasm	transporter
0.37	1.1	Q9CY64	BLVRA	biliverdin reductase A	Cytoplasm	enzyme
0.37	1.1	P00920	CA2	carbonic anhydrase 2	Cytoplasm	enzyme
0.54	1.1	Q99LB4	CAPG	capping actin protein, gelsolin like	Nucleus	other
0.49	1.1	Q5RKN9	CAPZA1	capping actin protein of muscle Z-line subunit alpha 1	Cytoplasm	other
0.48	1.1	A2AMW0	CAPZB	capping actin protein of muscle Z-line subunit beta	Cytoplasm	other
0.48	1.1	P40240	CD9	CD9 molecule	Plasma Membrane	other

Table S3

0.45	1.1	Q9D1A2	CNDP2	carnosine dipeptidase 2	Cytoplasm	peptidase
0.4	1.1	O89053	CORO1A	coronin 1A	Cytoplasm	other
0.61	1.1	P41241	CSK	C-terminal Src kinase	Cytoplasm	kinase
0.43	1.1	P18242	CTSD	cathepsin D	Cytoplasm	peptidase
0.62	1.1	O70370	CTSS	cathepsin S	Cytoplasm	peptidase
0.55	1.1	P54823	DDX6	DEAD-box helicase 6	Nucleus	enzyme
0.57	1.1	Q91YP3	DERA	deoxyribose-phosphate aldolase	Cytoplasm	enzyme
0.58	1.1	A0A0R4IZY9	DUS3L	dihydrouridine synthase 3 like	Other	other
0.26	1.1	Q8C4U8	EDIL3	EGF like repeats and discoidin domains 3	Extracellular Space	other
0.57	1.1	Q8BGD9	EIF4B	eukaryotic translation initiation factor 4B	Cytoplasm	translation regulator
0.58	1.1	Q7TT37	ELP1	elongator complex protein 1	Cytoplasm	other
0.62	1.1	A2A841	EPB41	erythrocyte membrane protein band 4.1	Plasma Membrane	other
0.58	1.1	P30416	FKBP4	FK506 binding protein 4	Nucleus	enzyme
0.47	1.1	A2AB60	Fmnl1	formin-like 1	Cytoplasm	other
0.48	1.1	P09528	FTH1	ferritin heavy chain 1	Cytoplasm	enzyme
0.48	1.1	P0DOV2	IFI16	interferon gamma inducible protein 16	Nucleus	transcription regulator
0.62	1.1	E9Q3Y4	LRBA	LPS responsive beige-like anchor protein	Cytoplasm	other
0.48	1.1	Q63844	MAPK3	mitogen-activated protein kinase 3	Cytoplasm	kinase
0.5	1.1	P21956	MFGE8	milk fat globule-EGF factor 8 protein	Extracellular Space	other
0.55	1.1	Q99J77	NANS	N-acetylneuraminate synthase	Cytoplasm	enzyme
0.4	1.1	Q8BP47	NARS	asparaginyl-tRNA synthetase	Cytoplasm	enzyme
0.55	1.1	M0QWK1	NDFIP2	Nedd4 family interacting protein 2	Cytoplasm	other
0.62	1.1	P97300	Nptn	neuroplastin	Plasma Membrane	other
0.61	1.1	A0A0G2JGQ4	NUB1	negative regulator of ubiquitin like proteins 1	Nucleus	other
0.45	1.1	Q9DCD0	PGD	phosphogluconate dehydrogenase	Cytoplasm	enzyme
0.62	1.1	Q61151	PPP2R5E	protein phosphatase 2 regulatory subunit B'epsilon	Cytoplasm	phosphatase
0.48	1.1	G3UXL2	Prps1l3	phosphoribosyl pyrophosphate synthetase 1-like 3	Other	kinase
0.34	1.1	P62196	PSMC5	proteasome 26S subunit, ATPase 5	Nucleus	transcription regulator
0.57	1.1	Q8BJY1	PSMD5	proteasome 26S subunit, non-ATPase 5	Other	other
0.57	1.1	P42669	PURA	purine rich element binding protein A	Nucleus	transcription regulator
0.5	1.1	Q62159	RHOC	ras homolog family member C	Plasma Membrane	enzyme
0.53	1.1	Q5XJF6	RPL10A	ribosomal protein L10a	Nucleus	other
0.38	1.1	P41105	RPL28	ribosomal protein L28	Cytoplasm	other
0.35	1.1	P62702	RPS4Y1	ribosomal protein S4 Y-linked 1	Cytoplasm	other
0.54	1.1	E9Q855	SCAMP3	secretory carrier membrane protein 3	Cytoplasm	transporter
0.23	1.1	Q3TMX0	SDCBP	syndecan binding protein	Plasma Membrane	enzyme

Table S3

0.27	1.1	G5E866	SF3B1	splicing factor 3b subunit 1	Nucleus	other
0.57	1.1	P50431	SHMT1	serine hydroxymethyltransferase 1	Cytoplasm	enzyme
0.46	1.1	G5E8Z4	SLC6A12	solute carrier family 6 member 12	Plasma Membrane	transporter
0.5	1.1	Q04692	SMARCAD1	SWI/SNF-related, matrix-associated actin-dependent regulator of chromatin, subfamily a, containing DEAD/H box 1	Nucleus	enzyme
0.62	1.1	P84104	SRSF3	serine and arginine rich splicing factor 3	Nucleus	other
0.58	1.1	F8WJD4	SYMPK	symplekin	Cytoplasm	other
0.18	1.1	P26039	TLN1	talin 1	Plasma Membrane	other
0.61	1.1	Q8BFY9	TNPO1	transportin 1	Nucleus	transporter
0.62	1.1	P61082	UBE2M	ubiquitin conjugating enzyme E2 M	Cytoplasm	enzyme
0.48	1.1	E9PYI8	USP14	ubiquitin specific peptidase 14	Cytoplasm	peptidase
0.57	1.1	K4DI77	WDR81	WD repeat domain 81	Plasma Membrane	other
0.62	1.1	P17095				
0.43	1.1	P68433				
0.27	1.2	P61222	ABCE1	ATP binding cassette subfamily E member 1	Cytoplasm	transporter
0.55	1.2	Z4YJY0	ABRAXAS2	abraxas 2, BRISC complex subunit	Nucleus	other
0.52	1.2	P61164	ACTR1A	ARP1 actin related protein 1 homolog A	Cytoplasm	other
0.44	1.2	E9Q616	AHNAK	AHNAK nucleoprotein	Nucleus	other
0.42	1.2	P45377	AKR1B10	aldo-keto reductase family 1 member B10	Cytoplasm	enzyme
0.35	1.2	A6ZI44	ALDOA	aldolase, fructose-bisphosphate A	Cytoplasm	enzyme
0.48	1.2	A0A0A6YX18	ATP6V1H	ATPase H+ transporting V1 subunit H	Cytoplasm	transporter
0.67	1.2	D3YUP1	CARM1	coactivator associated arginine methyltransferase 1	Nucleus	transcription regulator
0.45	1.2	Q8BT60	CPNE3	copine 3	Cytoplasm	kinase
0.34	1.2	E9Q4G7	CSNK1A1	casein kinase 1 alpha 1	Cytoplasm	kinase
0.55	1.2	H7BWZ9	DOP1A	DOP1 leucine zipper like protein A	Cytoplasm	other
0.001	1.2	P10126	EEF1A1	eukaryotic translation elongation factor 1 alpha 1	Cytoplasm	translation regulator
0.14	1.2	Q9EQP2	EHD4	EH domain containing 4	Plasma Membrane	enzyme
0.58	1.2	Q61749	EIF2B4	eukaryotic translation initiation factor 2B subunit delta	Cytoplasm	other
0.37	1.2	Q9QZD9	EIF3I	eukaryotic translation initiation factor 3 subunit I	Cytoplasm	translation regulator
0.33	1.2	H3BKH6	ESD	esterase D	Cytoplasm	enzyme
0.42	1.2	E9PYV4	FAM129A	family with sequence similarity 129 member A	Cytoplasm	other
0.57	1.2	P26151	FCGR1A	Fc fragment of IgG receptor Ia	Plasma Membrane	transmembrane receptor
0.51	1.2	Q920E5	FDPS	farnesyl diphosphate synthase	Cytoplasm	enzyme

Table S3

0.62	1.2	O09172	GCLM	glutamate-cysteine ligase modifier subunit	Cytoplasm	enzyme
0.17	1.2	P47856	GFPT1	glutamine--fructose-6-phosphate transaminase 1	Cytoplasm	enzyme
0.23	1.2	P62880	GNB2	G protein subunit beta 2	Plasma Membrane	enzyme
0.33	1.2	Q8CGP5	HIST1H2AJ	histone cluster 1 H2A family member j	Nucleus	other
0.57	1.2	P01901	HLA-A	major histocompatibility complex, class I, A	Plasma Membrane	other
0.35	1.2	Q3U2G2	HSPA4	heat shock protein family A (Hsp70) member 4	Cytoplasm	other
0.62	1.2	P38647	HSPA9	heat shock protein family A (Hsp70) member 9	Cytoplasm	other
0.073	1.2	Q9CQW9	IFITM3	interferon induced transmembrane protein 3	Plasma Membrane	other
0.57	1.2	Q64339	ISG15	ISG15 ubiquitin-like modifier	Extracellular Space	other
0.38	1.2	O89109	KCNN4	potassium calcium-activated channel subfamily N member 4	Plasma Membrane	ion channel
0.52	1.2	E9PUB7	MSTO1	misato 1, mitochondrial distribution and morphology regulator	Cytoplasm	other
0.42	1.2	Q78HU3	MVB12A	multivesicular body subunit 12A	Cytoplasm	other
0.41	1.2	Q5SUA5	MYO1G	myosin IG	Cytoplasm	other
0.65	1.2	Q8C5P5	NT5DC1	5'-nucleotidase domain containing 1	Other	other
0.65	1.2	D3YWF6	Otub1	OTU domain, ubiquitin aldehyde binding 1	Cytoplasm	enzyme
0.35	1.2	Q6P1F6	PPP2R2A	protein phosphatase 2 regulatory subunit Balpha	Cytoplasm	phosphatase
0.62	1.2	Q66GT5	PTPMT1	protein tyrosine phosphatase, mitochondrial 1	Cytoplasm	phosphatase
0.65	1.2	P34022	RANBP1	RAN binding protein 1	Nucleus	other
0.51	1.2	P62889	RPL30	ribosomal protein L30	Cytoplasm	other
0.28	1.2	P47962	RPL5	ribosomal protein L5	Cytoplasm	other
0.67	1.2	A0A1W2P7A1	RPS12	ribosomal protein S12	Cytoplasm	other
0.27	1.2	P14131	RPS16	ribosomal protein S16	Cytoplasm	other
0.17	1.2	Q8C483	SARS	seryl-tRNA synthetase	Cytoplasm	enzyme
0.65	1.2	P32037	SLC2A3	solute carrier family 2 member 3	Plasma Membrane	transporter
0.5	1.2	Q62376	SNRNP70	small nuclear ribonucleoprotein U1 subunit 70	Nucleus	other
0.4	1.2	P40142	TKT	transketolase	Cytoplasm	enzyme
0.62	1.2	Q9D2E2	TOE1	target of EGR1, exonuclease	Nucleus	enzyme
0.42	1.2	Q04750	TOP1	DNA topoisomerase I	Nucleus	enzyme
0.43	1.2	Q61187	TSG101	tumor susceptibility 101	Cytoplasm	transcription regulator
0.37	1.2	P68372	TUBB4B	tubulin beta 4B class IVb	Cytoplasm	other
0.5	1.2	Q8BHB4	WDR3	WD repeat domain 3	Cytoplasm	other
0.087	1.2	Q6GSS7				
0.13	1.2	TRYP_PIG				
0.34	1.2	A0A1B0GS68				
0.17	1.3	Q8BGQ7	AARS	alanyl-tRNA synthetase	Cytoplasm	enzyme

Table S3

0.0001	1.3	P63260	ACTG1	actin gamma 1	Cytoplasm	other
0.35	1.3	P61161	ACTR2	ARP2 actin related protein 2 homolog	Plasma Membrane	other
0.5	1.3	Q8CG76	AKR7A2	aldo-keto reductase family 7 member A2	Cytoplasm	enzyme
0.15	1.3	P10107	ANXA1	annexin A1	Plasma Membrane	enzyme
0.11	1.3	P97429	ANXA4	annexin A4	Plasma Membrane	other
0.56	1.3	ANXA5_HUMAN	ANXA5	annexin A5	Plasma Membrane	transporter
0.56	1.3	E9QAJ9	ARHGAP17	Rho GTPase activating protein 17	Cytoplasm	other
0.42	1.3	A2AWP8	ARHGEF10L	Rho guanine nucleotide exchange factor 10 like	Cytoplasm	enzyme
0.42	1.3	P61211	ARL1	ADP ribosylation factor like GTPase 1	Cytoplasm	enzyme
0.13	1.3	P50516	ATP6V1A	ATPase H+ transporting V1 subunit A	Plasma Membrane	transporter
0.51	1.3	Q8R2Q8	Bst2	bone marrow stromal cell antigen 2	Plasma Membrane	other
0.48	1.3	P35991	BTK	Bruton tyrosine kinase	Cytoplasm	kinase
0.56	1.3	P24270	CAT	catalase	Cytoplasm	enzyme
0.58	1.3	P51670	Ccl9	chemokine (C-C motif) ligand 9	Extracellular Space	cytokine
0.62	1.3	Q9JLQ0	CD2AP	CD2 associated protein	Cytoplasm	other
0.55	1.3	B1AWE0	CLTA	clathrin light chain A	Plasma Membrane	other
0.00015	1.3	Q68FD5	CLTC	clathrin heavy chain	Plasma Membrane	other
0.29	1.3	Q8C166	CPNE1	copine 1	Nucleus	transporter
0.65	1.3	G3X914	CUL5	cullin 5	Nucleus	ion channel
0.48	1.3	P32233	DRG1	developmentally regulated GTP binding protein 1	Cytoplasm	other
0.55	1.3	P60521	GABARAPL2	GABA type A receptor associated protein like 2	Cytoplasm	other
0.25	1.3	Q61598	GDI2	GDP dissociation inhibitor 2	Cytoplasm	other
0.56	1.3	Q9JLQ2	GIT2	GIT ArfGAP 2	Nucleus	other
0.55	1.3	Q9JHJ3	GLMP	glycosylated lysosomal membrane protein	Cytoplasm	transcription regulator
0.27	1.3	P21279	GNAQ	G protein subunit alpha q	Plasma Membrane	enzyme
0.37	1.3	Q9Z1E4	GYS1	glycogen synthase 1	Cytoplasm	enzyme
0.00014	1.3	P63017	HSPA8	heat shock protein family A (Hsp70) member 8	Cytoplasm	enzyme
0.5	1.3	Q60943	IL17RA	interleukin 17 receptor A	Plasma Membrane	transmembrane receptor
0.56	1.3	Q99MN1	KARS	lysyl-tRNA synthetase	Cytoplasm	enzyme
0.083	1.3	K1C9_HUMAN	KRT9	keratin 9	Cytoplasm	other
0.5	1.3	A0A087WRH9	MDFIC	MyoD family inhibitor domain containing	Nucleus	other
0.5	1.3	P22366	MYD88	myeloid differentiation primary response 88	Plasma Membrane	other
0.56	1.3	Q9CQF3	NUDT21	nudix hydrolase 21	Nucleus	other
0.56	1.3	Q9WVE8	PACSIN2	protein kinase C and casein kinase substrate in neurons 2	Cytoplasm	transporter

Table S3

0.56	1.3	F7D432	Pcmt1	protein-L-isoaspartate (D-aspartate) O-methyltransferase 1	Cytoplasm	enzyme
0.29	1.3	P35700	PRDX1	peroxiredoxin 1	Cytoplasm	enzyme
0.79	1.3	Q5EG47	PRKAA1	protein kinase AMP-activated catalytic subunit alpha 1	Cytoplasm	kinase
0.46	1.3	A0A171KXD3	PRMT1	protein arginine methyltransferase 1	Nucleus	enzyme
0.38	1.3	Q99K85	PSAT1	phosphoserine aminotransferase 1	Cytoplasm	enzyme
0.5	1.3	P61290	PSME3	proteasome activator subunit 3	Cytoplasm	peptidase
0.62	1.3	E9Q2A6	PTK2B	protein tyrosine kinase 2 beta	Cytoplasm	kinase
0.5	1.3	Q8BU03	Pwp2	PWP2 periodic tryptophan protein homolog (yeast)	Nucleus	other
0.38	1.3	Q9CXW4	RPL11	ribosomal protein L11	Cytoplasm	other
0.21	1.3	P62717	RPL18A	ribosomal protein L18a	Cytoplasm	other
0.51	1.3	P62911	Rpl32	ribosomal protein L32	Cytoplasm	other
0.69	1.3	Q6ZWZ4	Rpl36	ribosomal protein L36	Nucleus	other
0.18	1.3	P12970	RPL7A	ribosomal protein L7a	Cytoplasm	other
0.45	1.3	P47955	Rplp1 (includes others)	ribosomal protein, large, P1	Nucleus	other
0.037	1.3	P62983	RPS27A	ribosomal protein S27a	Cytoplasm	other
0.5	1.3	G3X972	SEC24C	SEC24 homolog C, COPII coat complex component	Cytoplasm	transporter
0.51	1.3	E9Q1G8	SEPT7	septin 7	Cytoplasm	other
0.58	1.3	Q62419	SH3GL1	SH3 domain containing GRB2 like 1, endophilin A2	Cytoplasm	other
0.65	1.3	Q9CW03	SMC3	structural maintenance of chromosomes 3	Nucleus	other
0.55	1.3	P08228	SOD1	superoxide dismutase 1	Cytoplasm	enzyme
0.11	1.3	Q64337	SQSTM1	sequestosome 1	Cytoplasm	transcription regulator
0.67	1.3	Q3TWW8	SRSF6	serine and arginine rich splicing factor 6	Nucleus	other
0.62	1.3	O55098	STK10	serine/threonine kinase 10	Cytoplasm	kinase
0.42	1.3	Q9CXF4	TBC1D15	TBC1 domain family member 15	Cytoplasm	other
0.2	1.3	Q62351	TFRC	transferrin receptor	Plasma Membrane	transporter
0.55	1.3	Q9R1Q6	TMEM176B	transmembrane protein 176B	Other	other
0.29	1.3	Q3UDC3	TOM1	target of myb1 membrane trafficking protein	Cytoplasm	transporter
0.38	1.3	Q5SRX1	TOM1L2	target of myb1 like 2 membrane trafficking protein	Cytoplasm	transporter
0.58	1.3	A2ASS6	TTN	titin	Cytoplasm	kinase
0.51	1.3	Q6P5F7	TTYH3	tweety family member 3	Plasma Membrane	ion channel
0.55	1.3	Q9D883	U2af1	U2 small nuclear ribonucleoprotein auxiliary factor (U2AF) 1	Nucleus	other
0.79	1.3	B1AQD9	UNC119	unc-119 lipid binding chaperone	Cytoplasm	other
0.43	1.3	Q8BX70	VPS13C	vacuolar protein sorting 13 homolog C	Cytoplasm	other
0.27	1.3	Q9EQH3	VPS35	VPS35, retromer complex component	Cytoplasm	transporter

Table S3

0.5	1.3	Q91XD6	VPS36	vacuolar protein sorting 36 homolog	Cytoplasm	other
0.5	1.3	Q8C754	VPS52	VPS52, GARP complex subunit	Cytoplasm	other
0.5	1.3	G5E8J3	WDR11	WD repeat domain 11	Cytoplasm	other
0.79	1.3	Q9JKB3	YBX3	Y-box binding protein 3	Nucleus	transcription regulator
0.38	1.3	P68254	YWHAQ	tyrosine 3-monooxygenase/tryptophan 5-monooxygenase activation protein theta	Cytoplasm	other
0.23	1.3	P63101	YWHAZ	tyrosine 3-monooxygenase/tryptophan 5-monooxygenase activation protein zeta	Cytoplasm	enzyme
0.5	1.3	Q8ZLZ4				
0.65	1.4	Q5SSL4	ABR	ABR, RhoGEF and GTPase activating protein	Cytoplasm	other
0.21	1.4	Q3U367	ALDH9A1	aldehyde dehydrogenase 9 family member A1	Cytoplasm	enzyme
0.13	1.4	Q61024	ASNS	asparagine synthetase (glutamine-hydrolyzing)	Cytoplasm	enzyme
0.8	1.4	O54962	BANF1	barrier to autointegration factor 1	Nucleus	other
0.65	1.4	Q99246	CACNA1D	calcium voltage-gated channel subunit alpha1 D	Plasma Membrane	ion channel
0.65	1.4	Q8K1A6	CC2D1A	coiled-coil and C2 domain containing 1A	Cytoplasm	transcription regulator
0.34	1.4	A2APM2	CD44	CD44 molecule (Indian blood group)	Plasma Membrane	other
0.8	1.4	F2Z456	Cyb5r3	cytochrome b5 reductase 3	Cytoplasm	enzyme
0.34	1.4	Q61462	CYBA	cytochrome b-245 alpha chain	Cytoplasm	enzyme
0.65	1.4	P61963	DCAF7	DDB1 and CUL4 associated factor 7	Cytoplasm	other
0.8	1.4	Q05816	FABP5	fatty acid binding protein 5	Cytoplasm	transporter
0.65	1.4	P0C0S6	H2AFZ	H2A histone family member Z	Nucleus	other
0.55	1.4	Q8BY71	HAT1	histone acetyltransferase 1	Nucleus	enzyme
0.29	1.4	P01897	HLA-A	major histocompatibility complex, class I, A	Plasma Membrane	other
0.43	1.4	P17879	Hspa1b	heat shock protein 1B	Cytoplasm	other
0.48	1.4	Q8BV66	IFI44	interferon induced protein 44	Cytoplasm	other
0.26	1.4	Q64282	IFIT1B	interferon induced protein with tetratricopeptide repeats 1B	Cytoplasm	other
0.8	1.4	P43406	ITGAV	integrin subunit alpha V	Plasma Membrane	transmembrane receptor
0.65	1.4	F8WH95	LAPTM5	lysosomal protein transmembrane 5	Plasma Membrane	other
0.061	1.4	Q61233	LCP1	lymphocyte cytosolic protein 1	Cytoplasm	other
0.65	1.4	P16045	LGALS1	galectin 1	Extracellular Space	other
0.65	1.4	Q8R502	LRRC8C	leucine rich repeat containing 8 VRAC subunit C	Plasma Membrane	ion channel
0.65	1.4	Q8BLN5	LSS	lanosterol synthase	Cytoplasm	enzyme
0.0039	1.4	Q8VDD5	MYH9	myosin heavy chain 9	Cytoplasm	enzyme
0.65	1.4	B1B1A8	MYLK	myosin light chain kinase	Cytoplasm	kinase
0.48	1.4	B7ZNL2	NAP1L4	nucleosome assembly protein 1 like 4	Cytoplasm	other

Table S3

0.8	1.4	Q99JX7	NXF1	nuclear RNA export factor 1	Nucleus	other
0.31	1.4	Q61990	PCBP2	poly(rC) binding protein 2	Nucleus	other
0.65	1.4	Q9CZX7	PIP4P2	phosphatidylinositol-4,5-bisphosphate 4-phosphatase 2	Cytoplasm	phosphatase
0.65	1.4	Q8VCE9	PLEKHH3	pleckstrin homology, MyTH4 and FERM domain containing H3	Extracellular Space	other
0.43	1.4	Q8CIG8	PRMT5	protein arginine methyltransferase 5	Cytoplasm	enzyme
0.33	1.4	Q9D0M1	PRPSAP1	phosphoribosyl pyrophosphate synthetase associated protein 1	Extracellular Space	other
0.8	1.4	A0A0R4J0P5	PSTPIP1	proline-serine-threonine phosphatase interacting protein 1	Cytoplasm	other
0.13	1.4	Q91V41	RAB14	RAB14, member RAS oncogene family	Cytoplasm	enzyme
0.29	1.4	Q05144	RAC2	Rac family small GTPase 2	Cytoplasm	enzyme
0.1	1.4	Q99JI6	RAP1B	RAP1B, member of RAS oncogene family	Cytoplasm	enzyme
0.65	1.4	A0A0A6YWG7	RAPGEF2	Rap guanine nucleotide exchange factor 2	Cytoplasm	other
0.38	1.4	Q5SVR5	RNF130	ring finger protein 130	Cytoplasm	peptidase
0.65	1.4	E9QNY8	Sacs	sacsin	Cytoplasm	other
0.65	1.4	A2AA71	SEC24A	SEC24 homolog A, COPII coat complex component	Cytoplasm	transporter
0.38	1.4	Q8CBA2	SLFN5	schlafen family member 5	Nucleus	enzyme
0.25	1.4	P58242	SMPDL3B	sphingomyelin phosphodiesterase acid like 3B	Extracellular Space	enzyme
0.43	1.4	Q9WTP2	SPRY4	sprouty RTK signaling antagonist 4	Plasma Membrane	other
0.67	1.4	Q3TFQ1	SPRYD7	SPRY domain containing 7	Other	other
0.65	1.4	Q9CQ26	STAMBP	STAM binding protein	Nucleus	enzyme
0.55	1.4	Q9QXJ2	STAT2	signal transducer and activator of transcription 2	Nucleus	transcription regulator
0.036	1.4	P54116	STOM	stomatin	Plasma Membrane	other
0.65	1.4	Q6PFX9	TNKS	tankyrase	Nucleus	enzyme
0.35	1.4	Q9QZ06	TOLLIP	toll interacting protein	Cytoplasm	other
0.65	1.4	Q923U0	TOM1L1	target of myb1 like 1 membrane trafficking protein	Cytoplasm	other
0.65	1.4	A0A0R4J0Q6	TRIM56	tripartite motif containing 56	Cytoplasm	enzyme
0.65	1.4	Q8CE96	TRMT6	tRNA methyltransferase 6	Nucleus	other
0.8	1.4	Q9JMH6	TXNRD1	thioredoxin reductase 1	Cytoplasm	enzyme
0.65	1.4	Q9ES00	UBE4B	ubiquitination factor E4B	Cytoplasm	enzyme
0.55	1.4	Q80WQ2	VAC14	Vac14, PIKFYVE complex component	Cytoplasm	other
0.33	1.4	P27870	VAV1	vav guanine nucleotide exchange factor 1	Nucleus	transcription regulator
0.65	1.4	L7N2E9	Vmn2r2	vomeronasal 2, receptor 2	Other	other
0.8	1.4	Q9D2N9	VPS33A	VPS33A, CORVET/HOPS core subunit	Cytoplasm	transporter
0.8	1.4	Q9CR26	VTA1	vesicle trafficking 1	Cytoplasm	other
0.8	1.4	Q8BH43	WASF2	WAS protein family member 2	Plasma Membrane	other
0.65	1.4	Q8BFQ4	WDR82	WD repeat domain 82	Nucleus	other

Table S3

0.42	1.4	Q5Y5T1	ZDHHC20	zinc finger DHHC-type containing 20	Plasma Membrane	enzyme
0.65	1.4	P59268	ZDHHC9	zinc finger DHHC-type containing 9	Cytoplasm	enzyme
0.55	1.4	Q7CQW9				
0.066	1.5	F8WIT2	ANXA6	annexin A6	Plasma Membrane	ion channel
0.34	1.5	O08529	CAPN2	calpain 2	Cytoplasm	peptidase
0.38	1.5	P35762	CD81	CD81 molecule	Plasma Membrane	other
0.058	1.5	Q9DC53	CPNE8	copine 8	Cytoplasm	other
0.16	1.5	Q9ESX5	DKC1	dyskerin pseudouridine synthase 1	Nucleus	enzyme
0.23	1.5	Q6R0H7	GNAS	GNAS complex locus	Plasma Membrane	enzyme
0.24	1.5	P06745	GPI	glucose-6-phosphate isomerase	Extracellular Space	enzyme
0.38	1.5	Q9QZQ8	H2AFY	H2A histone family member Y	Nucleus	other
0.19	1.5	Q61768	KIF5B	kinesin family member 5B	Cytoplasm	other
0.38	1.5	P28667	MARCKSL1	MARCKS like 1	Cytoplasm	other
0.21	1.5	Q9WTI7	MYO1C	myosin IC	Cytoplasm	enzyme
0.38	1.5	P29595	NEDD8	neural precursor cell expressed, developmentally down-regulated 8	Nucleus	enzyme
0.34	1.5	Q6PHQ9	PABPC4	poly(A) binding protein cytoplasmic 4	Cytoplasm	translation regulator
0.38	1.5	Q922E4	PCYT2	phosphate cytidylyltransferase 2, ethanolamine	Cytoplasm	enzyme
0.085	1.5	P62962	PFN1	profilin 1	Cytoplasm	other
0.58	1.5	O70172	PIP4K2A	phosphatidylinositol-5-phosphate 4-kinase type 2 alpha	Cytoplasm	kinase
0.35	1.5	Q9R1Q7	PLP2	proteolipid protein 2	Cytoplasm	transporter
0.25	1.5	Q99JI4	PSMD6	proteasome 26S subunit, non-ATPase 6	Cytoplasm	enzyme
0.21	1.5	P14115	RPL27A	ribosomal protein L27a	Cytoplasm	other
0.089	1.5	P14869	RPLP0	ribosomal protein lateral stalk subunit P0	Cytoplasm	other
0.046	1.5	P25444	RPS2	ribosomal protein S2	Cytoplasm	other
0.5	1.5	P50543	S100a11	S100 calcium binding protein A11	Cytoplasm	other
0.13	1.5	Q64324	STXBP2	syntaxin binding protein 2	Plasma Membrane	transporter
0.48	1.5	Q9WUN2	TBK1	TANK binding kinase 1	Cytoplasm	kinase
0.43	1.5	Q91XH6	VTI1B	vesicle transport through interaction with t-SNAREs 1B	Plasma Membrane	transporter
0.38	1.5	S4R1I3	XPNPEP1	X-prolyl aminopeptidase 1	Cytoplasm	peptidase
0.42	1.5	P10404				
0.055	1.5	P37592				
0.08	1.6	Q810B6	ANKFY1	ankyrin repeat and FYVE domain containing 1	Cytoplasm	transcription regulator
0.03	1.6	P97384	ANXA11	annexin A11	Nucleus	other
0.21	1.6	Q8K2Q7	BROX	BRO1 domain and CAAX motif containing	Cytoplasm	other
0.25	1.6	A0A0R4J1D0	CPNE2	copine 2	Cytoplasm	other

Table S3

0.48	1.6	B7ZMZ7	DIP2C	disco interacting protein 2 homolog C	Other	other
0.43	1.6	A0A0A6YVU8	Gm9774	adhesion regulating molecule 1 pseudogene	Other	other
0.55	1.6	B1ATZ0	HGS	hepatocyte growth factor-regulated tyrosine kinase substrate	Cytoplasm	other
0.5	1.6	A0A0R4J0T4	IKBKB	inhibitor of nuclear factor kappa B kinase subunit beta	Cytoplasm	kinase
0.12	1.6	O35682	MYADM	myeloid associated differentiation marker	Nucleus	other
0.14	1.6	P57716	NCSTN	nicastrin	Plasma Membrane	peptidase
0.38	1.6	Q6WKZ7	NOSTRIN	nitric oxide synthase trafficking	Cytoplasm	transcription regulator
0.18	1.6	Q5SW88	RAB1A	RAB1A, member RAS oncogene family	Cytoplasm	enzyme
0.022	1.6	P51150	RAB7A	RAB7A, member RAS oncogene family	Cytoplasm	enzyme
0.38	1.6	Q9ERU9	RGPD4 (includes others)	RANBP2-like and GRIP domain containing 5	Nucleus	enzyme
0.43	1.6	P62071	RRAS2	RAS related 2	Plasma Membrane	enzyme
0.21	1.6	P42208	SEPT2	septin 2	Cytoplasm	enzyme
0.013	1.6	Q9ESU7	SLC1A5	solute carrier family 1 member 5	Plasma Membrane	transporter
0.24	1.6	Q924S8	SPRED1	sprouty related EVH1 domain containing 1	Plasma Membrane	other
0.5	1.6	B1AZI6	THOC2	THO complex 2	Nucleus	other
0.38	1.6	P83887	TUBG1	tubulin gamma 1	Cytoplasm	other
0.15	1.6	Q62465	VAT1	vesicle amine transport 1	Plasma Membrane	transporter
0.43	1.6	P0A1D3				
0.65	1.7	P41233	ABCA1	ATP binding cassette subfamily A member 1	Plasma Membrane	transporter
0.65	1.7	Q7M759	ABHD17B	abhydrolase domain containing 17B	Plasma Membrane	peptidase
0.65	1.7	Q9JII6	AKR1A1	aldo-keto reductase family 1 member A1	Cytoplasm	enzyme
0.65	1.7	P05063	ALDOC	aldolase, fructose-bisphosphate C	Cytoplasm	enzyme
0.5	1.7	A0A1B0GTA4	BAX	BCL2 associated X, apoptosis regulator	Cytoplasm	transporter
0.65	1.7	A0A1D5RLQ9	CDC42BPA	CDC42 binding protein kinase alpha	Cytoplasm	kinase
0.65	1.7	Q62426	CSTB	cystatin B	Cytoplasm	peptidase
0.0026	1.7	Q9WVK4	EHD1	EH domain containing 1	Cytoplasm	other
0.65	1.7	P10630	EIF4A2	eukaryotic translation initiation factor 4A2	Cytoplasm	translation regulator
0.34	1.7	O55135	EIF6	eukaryotic translation initiation factor 6	Cytoplasm	translation regulator
0.23	1.7	P20934	EVI2A	ecotropic viral integration site 2A	Plasma Membrane	transmembrane receptor
0.16	1.7	P20491	FCER1G	Fc fragment of IgE receptor Ig	Plasma Membrane	transmembrane receptor

Table S3

0.65	1.7	D3Z7A7	FMNL3	formin like 3	Cytoplasm	other
0.084	1.7	E9Q3Z4	HK3	hexokinase 3	Cytoplasm	kinase
0.43	1.7	P63038	HSPD1	heat shock protein family D (Hsp60) member 1	Cytoplasm	enzyme
0.34	1.7	P13597	ICAM1	intercellular adhesion molecule 1	Plasma Membrane	transmembrane receptor
0.65	1.7	F6RPJ9	IDE	insulin degrading enzyme	Extracellular Space	peptidase
0.34	1.7	Q61635	Ifi47	interferon gamma inducible protein 47	Cytoplasm	other
0.65	1.7	A0A087WPT7	INPPL1	inositol polyphosphate phosphatase like 1	Cytoplasm	phosphatase
0.33	1.7	A0A087WRM2	ITM2C	integral membrane protein 2C	Cytoplasm	other
0.5	1.7	Q9CQ22	LAMTOR1	late endosomal/lysosomal adaptor, MAPK and MTOR activator 1	Plasma Membrane	other
0.65	1.7	Q8BYI6	LPCAT2	lysophosphatidylcholine acyltransferase 2	Cytoplasm	enzyme
0.2	1.7	B7ZNR9	MAP4K4	mitogen-activated protein kinase kinase kinase kinase 4	Cytoplasm	kinase
0.55	1.7	H3BKH2	Mia2	melanoma inhibitory activity 2	Cytoplasm	other
0.65	1.7	Q9CQS2	NOP10	NOP10 ribonucleoprotein	Nucleus	other
0.057	1.7	Q9D6Z1	NOP56	NOP56 ribonucleoprotein	Nucleus	other
0.33	1.7	A2RS43	PCDH7	protocadherin 7	Plasma Membrane	other
0.34	1.7	Q8BFY6	PEF1	penta-EF-hand domain containing 1	Cytoplasm	other
0.15	1.7	Q9DBJ1	PGAM1	phosphoglycerate mutase 1	Cytoplasm	phosphatase
0.5	1.7	Q9JIZ9	PLSCR3	phospholipid scramblase 3	Plasma Membrane	enzyme
0.65	1.7	J3QPG5	PSAP	prosaposin	Extracellular Space	enzyme
0.057	1.7	S4R1M0	PTPRC	protein tyrosine phosphatase, receptor type C	Plasma Membrane	phosphatase
0.48	1.7	P35285	RAB22A	RAB22A, member RAS oncogene family	Cytoplasm	enzyme
0.014	1.7	P35278	RAB5C	RAB5C, member RAS oncogene family	Cytoplasm	enzyme
0.65	1.7	P35279	RAB6A	RAB6A, member RAS oncogene family	Cytoplasm	enzyme
0.33	1.7	Q3TLP8	RAC1	Rac family small GTPase 1	Plasma Membrane	enzyme
0.43	1.7	O55142	RPL35A	ribosomal protein L35a	Cytoplasm	other
0.3	1.7	Q9D662	SEC23B	Sec23 homolog B, coat complex II component	Extracellular Space	transporter
0.057	1.7	P53986	SLC16A1	solute carrier family 16 member 1	Plasma Membrane	transporter
0.65	1.7	A2AMH5	SLC44A1	solute carrier family 44 member 1	Plasma Membrane	transporter
0.55	1.7	P62305	Snrpe	small nuclear ribonucleoprotein E	Nucleus	other
0.5	1.7	F8WJK8	ST13	ST13, Hsp70 interacting protein	Cytoplasm	other
0.087	1.7	A0A087WSP5	STAT1	signal transducer and activator of transcription 1	Nucleus	transcription regulator
0.65	1.7	Q80W45	STX2	syntaxin 2	Cytoplasm	transporter

Table S3

0.38	1.7	Q7TMM9	TUBB2A	tubulin beta 2A class IIa	Cytoplasm	other
0.25	1.7	A0A0G2JGL0	UBE2D3	ubiquitin conjugating enzyme E2 D3	Cytoplasm	enzyme
0.65	1.7	Q9CQ80	VPS25	vacuolar protein sorting 25 homolog	Cytoplasm	other
0.65	1.7	Q8ZQ10				
0.65	1.7	Q8ZRC1				
0.15	1.8	Q64343	ABCG1	ATP binding cassette subfamily G member 1	Plasma Membrane	transporter
0.5	1.8	Q99JW1	ABHD17A	abhydrolase domain containing 17A	Plasma Membrane	enzyme
0.38	1.8	Q91Z25	ARPC1B	actin related protein 2/3 complex subunit 1B	Cytoplasm	other
0.03	1.8	P62814	ATP6V1B2	ATPase H+ transporting V1 subunit B2	Cytoplasm	transporter
0.069	1.8	P18572	BSG	basigin (Ok blood group)	Plasma Membrane	transporter
0.5	1.8	A0A0R4IZW8	CAPNS1	calpain small subunit 1	Cytoplasm	peptidase
0.38	1.8	Q62192	CD180	CD180 molecule	Plasma Membrane	other
0.38	1.8	Q9DB34	CHMP2A	charged multivesicular body protein 2A	Cytoplasm	other
0.5	1.8	Q60737	CSNK2A1	casein kinase 2 alpha 1	Nucleus	kinase
0.5	1.8	Q99PU8	DHX30	DExH-box helicase 30	Nucleus	enzyme
0.33	1.8	E9QP49	Ehbp1l1	EH domain binding protein 1-like 1	Other	other
0.55	1.8	Q03963	EIF2AK2	eukaryotic translation initiation factor 2 alpha kinase 2	Cytoplasm	kinase
0.38	1.8	Q921M7	FAM49B	family with sequence similarity 49 member B	Extracellular Space	other
0.11	1.8	P27601	GNA13	G protein subunit alpha 13	Plasma Membrane	enzyme
0.021	1.8	P11835	ITGB2	integrin subunit beta 2	Plasma Membrane	transmembrane receptor
0.17	1.8	P24668	M6PR	mannose-6-phosphate receptor, cation dependent	Cytoplasm	transporter
0.25	1.8	Q9D074	MGRN1	mahogunin ring finger 1	Cytoplasm	enzyme
0.12	1.8	D3YVL0	MOV10	Mov10 RISC complex RNA helicase	Nucleus	enzyme
0.016	1.8	Q62433	NDRG1	N-myc downstream regulated 1	Nucleus	kinase
0.5	1.8	G3X9V0	PSME2	proteasome activator subunit 2	Cytoplasm	peptidase
0.34	1.8	P84096	RHOG	ras homolog family member G	Cytoplasm	enzyme
0.38	1.8	O88983	STX8	syntaxin 8	Plasma Membrane	other
0.5	1.8	A0A1Y7VM54	TMED10	transmembrane p24 trafficking protein 10	Cytoplasm	transporter
0.43	1.8	E9PWG2	TRAPPC8	trafficking protein particle complex 8	Cytoplasm	transporter
0.059	1.8	Q61510	TRIM25	tripartite motif containing 25	Cytoplasm	transcription regulator
0.5	1.8	Q8C7R4	UBA6	ubiquitin like modifier activating enzyme 6	Cytoplasm	enzyme
0.33	1.8	Q8BSN6	VAMP4	vesicle associated membrane protein 4	Cytoplasm	other
0.5	1.8	Q3UMB9	WASHC4	WASH complex subunit 4	Cytoplasm	other

Table S3

0.5	1.8	P97765	WBP2	WW domain binding protein 2	Cytoplasm	transcription regulator
0.14	1.8	P62259	YWHAE	tyrosine 3-monooxygenase/tryptophan 5-monooxygenase activation protein epsilon	Cytoplasm	other
0.0012	1.8	P02936				
0.27	1.8	E9PZF0				
0.14	1.8	E9QAZ2				
0.21	1.9	Q91V12	ACOT7	acyl-CoA thioesterase 7	Cytoplasm	enzyme
0.0001	1.9	Q8VDN2	ATP1A1	ATPase Na+/K+ transporting subunit alpha 1	Plasma Membrane	transporter
0.17	1.9	P51863	ATP6V0D1	ATPase H+ transporting V0 subunit d1	Cytoplasm	transporter
0.15	1.9	Q61735	CD47	CD47 molecule	Plasma Membrane	transmembrane receptor
0.33	1.9	E9QL31	DAB2	DAB2, clathrin adaptor protein	Plasma Membrane	other
0.12	1.9	P35550	FBL	fibrillarin	Nucleus	enzyme
0.014	1.9	J7NUP1	Irgm1	immunity-related GTPase family M member 1	Cytoplasm	other
0.12	1.9	Q8C863	ITCH	itchy E3 ubiquitin protein ligase	Nucleus	enzyme
0.029	1.9	E9Q604	ITGAM	integrin subunit alpha M	Plasma Membrane	transmembrane receptor
0.002	1.9	K1C10_HUMAN	KRT10	keratin 10	Cytoplasm	other
0.03	1.9	Q64281	LILRB4	leukocyte immunoglobulin like receptor B4	Plasma Membrane	other
0.00028	1.9	P25911	LYN	LYN proto-oncogene, Src family tyrosine kinase	Cytoplasm	kinase
0.43	1.9	E9PW66	NAP1L1	nucleosome assembly protein 1 like 1	Nucleus	other
0.33	1.9	P09103	P4HB	prolyl 4-hydroxylase subunit beta	Cytoplasm	enzyme
0.27	1.9	A0A0R4J0G4	RANBP10	RAN binding protein 10	Cytoplasm	other
0.084	1.9	Q9CPR4	RPL17	ribosomal protein L17	Cytoplasm	other
0.023	1.9	D3Z2W0	TNIP1	TNFAIP3 interacting protein 1	Nucleus	other
0.018	1.9	Q80U87	USP8	ubiquitin specific peptidase 8	Cytoplasm	peptidase
0.1	1.9	P46467	VPS4B	vacuolar protein sorting 4 homolog B	Cytoplasm	transporter
0.077	2	Q9QUJ7	ACSL4	acyl-CoA synthetase long chain family member 4	Cytoplasm	enzyme
0.0013	2	P97449	ANPEP	alanyl aminopeptidase, membrane	Plasma Membrane	peptidase
0.65	2	O35607	BMPR2	bone morphogenetic protein receptor type 2	Plasma Membrane	kinase
0.12	2	Q9QYJ0	DNAJA2	DnaJ heat shock protein family (Hsp40) member A2	Nucleus	enzyme
0.12	2	G3X922	DNAJC13	DnaJ heat shock protein family (Hsp40) member C13	Cytoplasm	other
0.65	2	Q8C845	EFHD2	EF-hand domain family member D2	Other	other
0.15	2	Q9JJ28	FLII	FLII, actin remodeling protein	Nucleus	other

Table S3

0.65	2	Q8K385	FRRS1	ferric chelate reductase 1	Plasma Membrane	transmembrane receptor
0.65	2	P23242	GJA1	gap junction protein alpha 1	Plasma Membrane	transporter
0.25	2	P05201	GOT1	glutamic-oxaloacetic transaminase 1	Cytoplasm	enzyme
0.12	2	Q00560	IL6ST	interleukin 6 signal transducer	Plasma Membrane	transmembrane receptor
0.43	2	A0A1L1SS10	KEAP1	kelch like ECH associated protein 1	Cytoplasm	transcription regulator
0.65	2	E9PZ88	MAN2C1	mannosidase alpha class 2C member 1	Cytoplasm	enzyme
0.38	2	A0A0R4J1C7	MFAP3	microfibril associated protein 3	Extracellular Space	other
0.38	2	Q9JM52	MINK1	misshapen like kinase 1	Cytoplasm	kinase
0.65	2	Q6URW6	MYH14	myosin heavy chain 14	Extracellular Space	enzyme
0.33	2	P97369	NCF4	neutrophil cytosolic factor 4	Cytoplasm	enzyme
0.65	2	Q6P9K9	Nrxn3	neurexin III	Plasma Membrane	other
0.044	2	Q8VI94	OASL	2'-5'-oligoadenylate synthetase like	Cytoplasm	enzyme
0.65	2	E2JF22	PIEZO1	piezo type mechanosensitive ion channel component 1	Cytoplasm	ion channel
0.0023	2	B2RXS4	PLXNB2	plexin B2	Plasma Membrane	transmembrane receptor
0.13	2	Q9WVJ2	PSMD13	proteasome 26S subunit, non-ATPase 13	Cytoplasm	peptidase
0.0069	2	Q91V35	PTPRA	protein tyrosine phosphatase, receptor type A	Plasma Membrane	phosphatase
0.43	2	Q9D1G1	RAB1B	RAB1B, member RAS oncogene family	Cytoplasm	other
0.25	2	P55258	RAB8A	RAB8A, member RAS oncogene family	Plasma Membrane	enzyme
0.018	2	I7HLV2	RPL10	ribosomal protein L10	Cytoplasm	translation regulator
0.65	2	Q6P8X1	SNX6	sorting nexin 6	Cytoplasm	transporter
0.38	2	Q0VGY8	TANC1	tetratricopeptide repeat, ankyrin repeat and coiled-coil containing 1	Plasma Membrane	other
0.069	2	P58681	TLR7	toll like receptor 7	Plasma Membrane	transmembrane receptor
0.38	2	D3Z2H9	Tpm3-rs7	tropomyosin 3, related sequence 7	Other	other
0.27	2	Q8VE47	UBA5	ubiquitin like modifier activating enzyme 5	Cytoplasm	enzyme
0.65	2	Q9CX97	WDR55	WD repeat domain 55	Nucleus	other
0.25	2	P68510	YWHAH	tyrosine 3-monooxygenase/tryptophan 5-monooxygenase activation protein eta	Cytoplasm	transcription regulator
0.65	2	B1AWL2	ZNF462	zinc finger protein 462	Nucleus	transcription regulator
0.65	2	Q8ZJV0				

Table S3

0.27	2.1	Q03265	ATP5F1A	ATP synthase F1 subunit alpha	Cytoplasm	transporter
0.5	2.1	Q91XV3	BASP1	brain abundant membrane attached signal protein 1	Nucleus	transcription regulator
0.5	2.1	Q8C3W1	C1orf198	chromosome 1 open reading frame 198	Cytoplasm	other
0.12	2.1	E9Q1W0	CAMK2D	calcium/calmodulin dependent protein kinase II delta	Cytoplasm	kinase
0.5	2.1	Q6SJQ0	CD300A	CD300a molecule	Plasma Membrane	transmembrane receptor
0.068	2.1	Q08857	CD36	CD36 molecule	Plasma Membrane	transmembrane receptor
0.17	2.1	Q6Q899	DDX58	DExD/H-box helicase 58	Cytoplasm	enzyme
0.5	2.1	Q8BWY3	ETF1	eukaryotic translation termination factor 1	Cytoplasm	translation regulator
0.5	2.1	A0A0R4J0H8	FNDC3B	fibronectin type III domain containing 3B	Cytoplasm	other
0.5	2.1	Q920B0	FRMD4B	FERM domain containing 4B	Cytoplasm	other
0.5	2.1	A0A0R4J0F6	GAK	cyclin G associated kinase	Nucleus	kinase
0.064	2.1	Q9DC51	GNAI3	G protein subunit alpha i3	Cytoplasm	enzyme
0.5	2.1	F6UND7	HCK	HCK proto-oncogene, Src family tyrosine kinase	Cytoplasm	kinase
0.0057	2.1	P20029	HSPA5	heat shock protein family A (Hsp70) member 5	Cytoplasm	enzyme
0.18	2.1	Q99J93	IFITM2	interferon induced transmembrane protein 2	Cytoplasm	other
0.27	2.1	Q8CIN4	PAK2	p21 (RAC1) activated kinase 2	Cytoplasm	kinase
0.25	2.1	Q8BHF7	PGS1	phosphatidylglycerophosphate synthase 1	Cytoplasm	enzyme
0.5	2.1	Q6GT24	PRDX6	peroxiredoxin 6	Cytoplasm	enzyme
0.5	2.1	Q8CI94	PYGB	glycogen phosphorylase B	Cytoplasm	enzyme
0.23	2.1	Q9QZL0	RIPK3	receptor interacting serine/threonine kinase 3	Plasma Membrane	kinase
0.43	2.1	Q80UG5	SEPT9	septin 9	Cytoplasm	enzyme
0.18	2.1	Q91Z67	SRGAP2	SLIT-ROBO Rho GTPase activating protein 2	Cytoplasm	other
0.27	2.1	P48025	SYK	spleen associated tyrosine kinase	Cytoplasm	kinase
0.5	2.1	Q9Z2M6	UBL3	ubiquitin like 3	Cytoplasm	other
0.088	2.1	O70404	VAMP8	vesicle associated membrane protein 8	Plasma Membrane	transporter
0.5	2.1	G3UX26	VDAC2	voltage dependent anion channel 2	Cytoplasm	ion channel
0.5	2.1	P10400				
0.5	2.1	A2AGH5				
0.25	2.2	Q9JLV1	BAG3	BCL2 associated athanogene 3	Cytoplasm	other
0.02	2.2	P18760	CFL1	cofilin 1	Nucleus	other
0.38	2.2	Q32NY4	CNNM3	cyclin and CBS domain divalent metal cation transport mediator 3	Other	other
0.18	2.2	Q61093	CYBB	cytochrome b-245 beta chain	Cytoplasm	enzyme
0.026	2.2	P39688	FYN	FYN proto-oncogene, Src family tyrosine kinase	Plasma Membrane	kinase
0.33	2.2	Q922H4	GMPPA	GDP-mannose pyrophosphorylase A	Cytoplasm	enzyme

Table S3

0.27	2.2	P19182	IFRD1	interferon related developmental regulator 1	Nucleus	other
0.006	2.2	A0A1Y7VME9	KIDINS220	kinase D interacting substrate 220	Nucleus	transcription regulator
0.068	2.2	Q9D8W5	PSMD12	proteasome 26S subunit, non-ATPase 12	Cytoplasm	other
0.38	2.2	P35293	RAB18	RAB18, member RAS oncogene family	Cytoplasm	enzyme
0.38	2.2	P53994	RAB2A	RAB2A, member RAS oncogene family	Cytoplasm	enzyme
0.38	2.2	Q6PHN9	RAB35	RAB35, member RAS oncogene family	Cytoplasm	enzyme
0.066	2.2	P61028	RAB8B	RAB8B, member RAS oncogene family	Cytoplasm	enzyme
0.088	2.2	Q9EPR4	SLC23A2	solute carrier family 23 member 2	Plasma Membrane	transporter
0.0026	2.2	P17809	SLC2A1	solute carrier family 2 member 1	Plasma Membrane	transporter
0.0019	2.2	Q8CFE6	SLC38A2	solute carrier family 38 member 2	Plasma Membrane	transporter
0.047	2.2	Q62312	TGFBR2	transforming growth factor beta receptor 2	Plasma Membrane	kinase
0.17	2.2	Q8R105	VPS37C	VPS37C, ESCRT-I subunit	Cytoplasm	other
0.38	2.2	P84244				
0.27	2.3	Q7TPQ9	ARRDC3	arrestin domain containing 3	Plasma Membrane	other
0.23	2.3	P30993	C5AR1	complement C5a receptor 1	Plasma Membrane	G-protein coupled receptor
0.25	2.3	Q5FWI3	CEMIP2	cell migration inducing hyaluronidase 2	Cytoplasm	enzyme
0.25	2.3	Q8VCN5	CTH	cystathionine gamma-lyase	Cytoplasm	enzyme
0.33	2.3	P11688	ITGA5	integrin subunit alpha 5	Plasma Membrane	transmembrane receptor
0.17	2.3	O70145	NCF2	neutrophil cytosolic factor 2	Cytoplasm	enzyme
0.18	2.3	E9Q9A9	OAS2	2'-5'-oligoadenylate synthetase 2	Cytoplasm	enzyme
0.65	2.3	A0A1W2P7X5	PLEKHN1	pleckstrin homology domain containing N1	Other	other
0.65	2.3	P23298	PRKCH	protein kinase C eta	Cytoplasm	kinase
0.65	2.3	Q9R1C7	PRPF40A	pre-mRNA processing factor 40 homolog A	Nucleus	other
0.2	2.3	P35282	RAB21	RAB21, member RAS oncogene family	Cytoplasm	enzyme
0.27	2.3	Q9QUI0	RHOA	ras homolog family member A	Cytoplasm	enzyme
0.00083	2.3	P10852	SLC3A2	solute carrier family 3 member 2	Plasma Membrane	transporter
0.27	2.3	Q8C4J7	TBL3	transducin beta like 3	Cytoplasm	peptidase
0.27	2.3	Q9D1C8	VPS28	VPS28, ESCRT-I subunit	Cytoplasm	transporter
0.2	2.3	Q8C2E7	WASHC5	WASH complex subunit 5	Cytoplasm	other
0.65	2.3	Q9CWU2	ZDHHC13	zinc finger DHHC-type containing 13	Nucleus	transcription regulator
0.0026	2.3	P23988				
0.5	2.4	Q3U0L2	ANKRD33B	ankyrin repeat domain 33B	Other	other
0.38	2.4	O54833	CSNK2A2	casein kinase 2 alpha 2	Cytoplasm	kinase
0.17	2.4	Q91W53	GOLGA7	golgin A7	Cytoplasm	other

Table S3

0.38	2.4	A0A0R4J100	GPR84	G protein-coupled receptor 84	Plasma Membrane	G-protein coupled receptor
0.38	2.4	P48722	HSPA4L	heat shock protein family A (Hsp70) member 4 like	Cytoplasm	other
0.068	2.4	Q60605	MYL6	myosin light chain 6	Cytoplasm	enzyme
0.035	2.4	P11928	OAS1	2'-5'-oligoadenylate synthetase 1	Cytoplasm	enzyme
0.38	2.4	Q9JJ00	PLSCR1	phospholipid scramblase 1	Plasma Membrane	enzyme
0.5	2.4	Q6PD03	PPP2R5A	protein phosphatase 2 regulatory subunit B'alpha	Cytoplasm	phosphatase
0.38	2.4	Q5SSW2	PSME4	proteasome activator subunit 4	Cytoplasm	other
0.094	2.4	P61027	RAB10	RAB10, member RAS oncogene family	Cytoplasm	enzyme
0.064	2.4	G3X926	RPF2	ribosome production factor 2 homolog	Nucleus	other
0.5	2.4	P49282	SLC11A2	solute carrier family 11 member 2	Plasma Membrane	transporter
0.5	2.4	P52875	TMEM165	transmembrane protein 165	Plasma Membrane	other
0.5	2.4	Q8QZY6	TSPAN14	tetraspanin 14	Plasma Membrane	other
0.14	2.4	P70280	VAMP7	vesicle associated membrane protein 7	Cytoplasm	transporter
0.5	2.4	Q8R0J7	VPS37B	VPS37B, ESCRT-I subunit	Cytoplasm	other
0.5	2.4	Q8ZN72				
0.38	2.5	Q9D2R0	AACS	acetoacetyl-CoA synthetase	Cytoplasm	enzyme
0.036	2.5	P61205	ARF3	ADP ribosylation factor 3	Cytoplasm	enzyme
0.13	2.5	Q9D8B3	CHMP4B	charged multivesicular body protein 4B	Cytoplasm	other
0.27	2.5	CASB_BOVIN	CSN2	casein beta	Extracellular Space	kinase
0.068	2.5	P13020	GSN	gelsolin	Extracellular Space	other
0.27	2.5	A0A1W2P768	HIST1H3C	histone cluster 1 H3 family member c	Nucleus	other
0.096	2.5	P17047	LAMP2	lysosomal associated membrane protein 2	Plasma Membrane	enzyme
0.38	2.5	Q9JL15	LGALS8	galectin 8	Extracellular Space	other
0.27	2.5	Q7TQH7	LRP10	LDL receptor related protein 10	Plasma Membrane	transmembrane receptor
0.068	2.5	E0CZ22	MROH1	maestro heat like repeat family member 1	Other	other
0.0031	2.5	E9Q634	MYO1E	myosin IE	Cytoplasm	enzyme
0.38	2.5	Q99K51	PLS3	plastin 3	Cytoplasm	other
0.38	2.5	Q61009	SCARB1	scavenger receptor class B member 1	Plasma Membrane	transporter
0.27	2.5	O35316	SLC6A6	solute carrier family 6 member 6	Plasma Membrane	transporter
0.066	2.5	Q3UHD6	SNX27	sorting nexin family member 27	Cytoplasm	other
0.05	2.5	P25119	TNFRSF1B	TNF receptor superfamily member 1B	Plasma Membrane	transmembrane receptor
0.0025	2.5	Q8BVW3	TRIM14	tripartite motif containing 14	Cytoplasm	other

Table S3

0.25	2.5	A0A140LHP7	TYROBP	TYRO protein tyrosine kinase binding protein	Plasma Membrane	transmembrane receptor
0.38	2.5	O88693	UGCG	UDP-glucose ceramide glucosyltransferase	Cytoplasm	enzyme
0.00079	2.5	A0A1D5RM92	WWP2	WW domain containing E3 ubiquitin protein ligase 2	Cytoplasm	enzyme
0.17	2.6	Q4LDD4	ARAP1	ArfGAP with RhoGAP domain, ankyrin repeat and PH domain 1	Cytoplasm	other
0.0019	2.6	P56480	ATP5F1B	ATP synthase F1 subunit beta	Cytoplasm	transporter
0.25	2.6	Q9QYI3	DNAJC7	DnaJ heat shock protein family (Hsp40) member C7	Cytoplasm	other
0.18	2.6	B1AQR8	LGALS9B	galectin 9B	Cytoplasm	other
0.0001	2.6	P26041	MSN	moesin	Plasma Membrane	other
0.0044	2.6	Q9JHK5	PLEK	pleckstrin	Cytoplasm	transcription regulator
0.008	2.6	Q09143	SLC7A1	solute carrier family 7 member 1	Plasma Membrane	transporter
0.2	2.6	Q78ZM0	SNX3	sorting nexin 3	Cytoplasm	transporter
0.2	2.6	Q9CQV8	YWHAB	tyrosine 3-monooxygenase/tryptophan 5-monooxygenase activation protein beta	Cytoplasm	other
0.092	2.6	P61982	YWHAG	tyrosine 3-monooxygenase/tryptophan 5-monooxygenase activation protein gamma	Cytoplasm	other
0.38	2.7	O35379	ABCC1	ATP binding cassette subfamily C member 1	Plasma Membrane	transporter
0.2	2.7	A0A0A0MQD4	C5orf15	chromosome 5 open reading frame 15	Other	other
0.38	2.7	P27512	CD40	CD40 molecule	Plasma Membrane	transmembrane receptor
0.38	2.7	A2A7F6	CLCN6	chloride voltage-gated channel 6	Plasma Membrane	ion channel
0.38	2.7	Q8BTJ4	ENPP4	ectonucleotide pyrophosphatase/phosphodiesterase 4	Cytoplasm	enzyme
0.38	2.7	Q08509	EPS8	epidermal growth factor receptor pathway substrate 8	Plasma Membrane	peptidase
0.13	2.7	Q8K0B2	LMBRD1	LMBR1 domain containing 1	Cytoplasm	enzyme
0.38	2.7	P49769	PSEN1	presenilin 1	Plasma Membrane	peptidase
0.38	2.7	Q9R0M6	RAB9A	RAB9A, member RAS oncogene family	Cytoplasm	enzyme
0.38	2.7	Q6PFQ7	RASA4	RAS p21 protein activator 4	Cytoplasm	other
0.0042	2.7	Q3U2C5	RNF149	ring finger protein 149	Cytoplasm	enzyme
0.38	2.7	O08547	SEC22B	SEC22 homolog B, vesicle trafficking protein (gene/pseudogene)	Cytoplasm	other
0.096	2.7	O88811	STAM2	signal transducing adaptor molecule 2	Cytoplasm	other
0.13	2.7	Q8BH40	STX7	syntaxin 7	Plasma Membrane	transporter
0.003	2.8	P68134	ACTA1	actin, alpha 1, skeletal muscle	Cytoplasm	other

Table S3

0.049	2.8	Q3UH60	DIP2B	disco interacting protein 2 homolog B	Cytoplasm	other
0.14	2.8	A0A0R4J0A4	FLT1	fms related tyrosine kinase 1	Plasma Membrane	kinase
0.019	2.8	P08113	HSP90B1	heat shock protein 90 beta family member 1	Cytoplasm	other
0.0058	2.8	E9QN37	MPEG1	macrophage expressed 1	Cytoplasm	other
0.27	2.8	P30204	MSR1	macrophage scavenger receptor 1	Plasma Membrane	transmembrane receptor
0.068	2.8	F8WGS1	RAB11A	RAB11A, member RAS oncogene family	Cytoplasm	enzyme
0.14	2.8	Q8K021	SCAMP1	secretory carrier membrane protein 1	Cytoplasm	transporter
0.18	2.8	P37432				
0.18	2.9	A0A1L1SRX2	AMPD3	adenosine monophosphate deaminase 3	Cytoplasm	enzyme
0.096	2.9	Q8C129	LNPEP	leucyl and cystinyl aminopeptidase	Cytoplasm	peptidase
0.018	2.9	A0A0G2JGP4	NRAS	NRAS proto-oncogene, GTPase	Plasma Membrane	enzyme
0.2	2.9	B9EJ86	OSBPL8	oxysterol binding protein like 8	Plasma Membrane	transporter
0.094	2.9	O54965	RNF13	ring finger protein 13	Cytoplasm	enzyme
0.096	2.9	Q9ERN0	SCAMP2	secretory carrier membrane protein 2	Cytoplasm	transporter
0.27	2.9	Q3UU41	SCIMP	SLP adaptor and CSK interacting membrane protein	Plasma Membrane	other
0.096	2.9	Q9QZI8	SERINC1	serine incorporator 1	Plasma Membrane	transporter
0.0069	2.9	Q6P6I8	SIRPA	signal regulatory protein alpha	Plasma Membrane	phosphatase
0.036	2.9	Q3UPF5	ZC3HAV1	zinc finger CCCH-type containing, antiviral 1	Plasma Membrane	other
0.0053	3	Q9CQD1	RAB5A	RAB5A, member RAS oncogene family	Cytoplasm	enzyme
0.096	3	Q9CZX8	RPS19	ribosomal protein S19	Cytoplasm	other
0.38	3	Q91W98	SLC15A4	solute carrier family 15 member 4	Cytoplasm	transporter
0.026	3	D3YVM2	TMEM59	transmembrane protein 59	Plasma Membrane	peptidase
0.38	3	Q91YT8	TMEM63A	transmembrane protein 63A	Cytoplasm	other
0.5	3	Q80TN5	ZDHHC17	zinc finger DHHC-type containing 17	Cytoplasm	enzyme
0.38	3	Q8VDZ4	ZDHHC5	zinc finger DHHC-type containing 5	Nucleus	enzyme
0.0001	3.1	P97370	ATP1B3	ATPase Na+/K+ transporting subunit beta 3	Plasma Membrane	transporter
0.27	3.1	Q9WU60	ATRN	attractin	Extracellular Space	other
0.27	3.1	P35561	KCNJ2	potassium voltage-gated channel subfamily J member 2	Plasma Membrane	ion channel
0.27	3.1	Q9CPY7	LAP3	leucine aminopeptidase 3	Cytoplasm	peptidase
0.27	3.1	Q61792	LASP1	LIM and SH3 protein 1	Cytoplasm	transporter
0.27	3.1	Q5SYD0	MYO1D	myosin ID	Cytoplasm	enzyme
0.094	3.1	P27773	PDIA3	protein disulfide isomerase family A member 3	Cytoplasm	peptidase
0.38	3.1	P26043	RDX	radixin	Cytoplasm	other

Table S3

0.094	3.1	Q9ER00	STX12	syntaxin 12	Cytoplasm	other
0.049	3.1	Q9JKK1	STX6	syntaxin 6	Cytoplasm	transporter
0.067	3.1	O08599	STXBP1	syntaxin binding protein 1	Cytoplasm	transporter
0.0069	3.1	Q9JHF5	TCIRG1	T cell immune regulator 1, ATPase H+ transporting V0 subunit a3	Plasma Membrane	enzyme
0.38	3.1	D3Z0M2	TMEM106B	transmembrane protein 106B	Cytoplasm	other
0.27	3.1	Q8ZLU4				
0.2	3.2	Q64735	CR1L	complement C3b/C4b receptor 1 like	Plasma Membrane	other
0.2	3.2	O88630	GOSR1	golgi SNAP receptor complex member 1	Cytoplasm	transporter
0.067	3.2	Q9CX00	IST1	IST1, ESCRT-III associated factor	Cytoplasm	other
0.2	3.2	Q9QUN7	TLR2	toll like receptor 2	Plasma Membrane	transmembrane receptor
0.0001	3.3	E9Q3X0	MVP	major vault protein	Nucleus	other
0.18	3.3	F8WHW3	PIP4P1	phosphatidylinositol-4,5-bisphosphate 4-phosphatase 1	Cytoplasm	phosphatase
0.14	3.3	P61226	RAP2B	RAP2B, member of RAS oncogene family	Plasma Membrane	enzyme
0.14	3.3	Q9QZI9	SERINC3	serine incorporator 3	Cytoplasm	transporter
0.094	3.3	Q6P069	SRI	sorcin	Cytoplasm	transporter
0.13	3.3	E9PYK0	UNC93B1	unc-93 homolog B1, TLR signaling regulator	Cytoplasm	transporter
0.0001	3.3	P0A263				
0.012	3.4	E9Q3Q6	ALCAM	activated leukocyte cell adhesion molecule	Plasma Membrane	other
0.27	3.4	Q6P9J9	ANO6	anoctamin 6	Plasma Membrane	ion channel
0.27	3.4	Q8C708	C16orf54	chromosome 16 open reading frame 54	Extracellular Space	other
0.27	3.4	A2A6Z2	CD300LF	CD300 molecule like family member f	Plasma Membrane	other
0.27	3.4	Q6PDK8	DTX4	deltex E3 ubiquitin ligase 4	Cytoplasm	enzyme
0.27	3.4	P16382	IL4R	interleukin 4 receptor	Plasma Membrane	transmembrane receptor
0.27	3.4	Q9DB05	NAPA	NSF attachment protein alpha	Cytoplasm	transporter
0.27	3.4	D6RH77	PLD1	phospholipase D1	Cytoplasm	enzyme
0.00028	3.4	Q3UKC1	TAX1BP1	Tax1 binding protein 1	Cytoplasm	other
0.024	3.5	Q9QYB1	CLIC4	chloride intracellular channel 4	Plasma Membrane	ion channel
0.094	3.5	Q922R8	PDIA6	protein disulfide isomerase family A member 6	Cytoplasm	enzyme
0.032	3.5	Q91YQ5	RPN1	ribophorin I	Cytoplasm	enzyme
0.2	3.5	Q9JJR8	TMEM9B	TMEM9 domain family member B	Other	other
0.2	3.5	Q64727	VCL	vinculin	Plasma Membrane	enzyme
0.2	3.5	Q9Z0U0	XPR1	xenotropic and polytropic retrovirus receptor 1	Plasma Membrane	G-protein coupled receptor
0.2	3.5	Q8ZRP0				
0.2	3.5	Q7CPX8				

Table S3

0.064	3.6	Q99P91	GPNMB	glycoprotein nmb	Plasma Membrane	enzyme
0.0063	3.6	Q8CBQ5	PI4K2B	phosphatidylinositol 4-kinase type 2 beta	Cytoplasm	kinase
0.022	3.6	Q6PB44	PTPN23	protein tyrosine phosphatase, non-receptor type 23	Cytoplasm	phosphatase
0.064	3.6	Q99P72	RTN4	reticulon 4	Cytoplasm	other
0.094	3.6	O35114	SCARB2	scavenger receptor class B member 2	Plasma Membrane	transmembrane receptor
0.00024	3.6	F8VQC9	SLC4A7	solute carrier family 4 member 7	Plasma Membrane	transporter
0.067	3.6	Q9WUD1	STUB1	STIP1 homology and U-box containing protein 1	Cytoplasm	enzyme
0.0014	3.7	B1ASP2	JAK1	Janus kinase 1	Cytoplasm	kinase
0.27	3.7	D3Z4B2	NAPG	NSF attachment protein gamma	Cytoplasm	transporter
0.096	3.7	O35405	PLD3	phospholipase D family member 3	Cytoplasm	enzyme
0.008	3.7	Q3TXV4	RAB31	RAB31, member RAS oncogene family	Cytoplasm	enzyme
0.27	3.7	Q3TH73	TTYH2	tweety family member 2	Other	ion channel
0.2	3.8	Q9CYL5	GLIPR2	GLI pathogenesis related 2	Cytoplasm	other
0.14	3.8	P37040	POR	cytochrome p450 oxidoreductase	Cytoplasm	enzyme
0.0001	3.8	Q05769	PTGS2	prostaglandin-endoperoxide synthase 2	Cytoplasm	enzyme
0.2	3.8	Q9Z1A1	TFG	TRK-fused gene	Cytoplasm	other
0.094	3.8	Q8VC04	TMEM106A	transmembrane protein 106A	Cytoplasm	other
0.015	3.9	Q3U7G2	ADAM8	ADAM metallopeptidase domain 8	Plasma Membrane	peptidase
0.029	3.9	P06185				
0.2	4	Q8K4Q7	CERK	ceramide kinase	Plasma Membrane	kinase
0.017	4	Q8BUJ9	LRP12	LDL receptor related protein 12	Plasma Membrane	transmembrane receptor
0.2	4	Q61036	PAK3	p21 (RAC1) activated kinase 3	Cytoplasm	kinase
0.2	4	Q8BH24	TM9SF4	transmembrane 9 superfamily member 4	Cytoplasm	transporter
0.047	4	Q8ZNL0				
0.14	4.1	Q8R4B8	NLRP3	NLR family pyrin domain containing 3	Cytoplasm	other
0.0067	4.1	P63321	RALA	RAS like proto-oncogene A	Cytoplasm	enzyme
0.0055	4.1	Q8K078	SLCO4A1	solute carrier organic anion transporter family member 4A1	Plasma Membrane	transporter
0.14	4.4	Q91ZV3	DCBLD2	discoidin, CUB and LCCL domain containing 2	Plasma Membrane	other
0.14	4.4	Q99N69	LPXN	leupaxin	Cytoplasm	transcription regulator
0.022	4.4	P70206	PLXNA1	plexin A1	Plasma Membrane	transmembrane receptor
0.0001	4.4	Q8ZRJ9				
0.029	4.5	Q8K4Q8	COLEC12	collectin subfamily member 12	Plasma Membrane	transmembrane receptor

Table S3

0.0067	4.5	Q91VI7	RNH1	ribonuclease/angiogenin inhibitor 1	Cytoplasm	other
0.094	4.5	Q8ZRQ2				
0.064	4.6	P57787	SLC16A3	solute carrier family 16 member 3	Plasma Membrane	transporter
0.00028	4.6	Q7CQV8				
0.029	4.7	Q9WTR1	TRPV2	transient receptor potential cation channel subfamily V member 2	Plasma Membrane	ion channel
0.094	4.8	Q8CJ26	Nradd	neurotrophin receptor associated death domain	Extracellular Space	cytokine
0.094	4.8	P10833	RRAS	RAS related	Cytoplasm	enzyme
0.094	4.8	Q8BVL3	SNX17	sorting nexin 17	Cytoplasm	transporter
0.094	4.8	Q9ET30	TM9SF3	transmembrane 9 superfamily member 3	Cytoplasm	transporter
0.01	4.9	Q8BU31	RAP2C	RAP2C, member of RAS oncogene family	Cytoplasm	enzyme
0.043	5.2	O35604	NPC1	NPC intracellular cholesterol transporter 1	Cytoplasm	transporter
0.064	5.2	Q80WQ6	RHBDF2	rhomboid 5 homolog 2	Cytoplasm	other
0.0001	5.3	Q2TBE6	PI4K2A	phosphatidylinositol 4-kinase type 2 alpha	Cytoplasm	kinase
0.019	5.4	Q8R420	ABCA3	ATP binding cassette subfamily A member 3	Plasma Membrane	transporter
0.0045	5.4	Q9R002	Ifi202b	interferon activated gene 202B	Nucleus	other
0.012	5.4	F8WIJ0	SLC12A4	solute carrier family 12 member 4	Plasma Membrane	transporter
0.0067	5.4	Q9D3L3	SNAP23	synaptosome associated protein 23	Plasma Membrane	transporter
0.043	5.5	B1AT92	GRB2	growth factor receptor bound protein 2	Cytoplasm	kinase
0.0011	5.5	Q8BPX9	SLC15A3	solute carrier family 15 member 3	Cytoplasm	transporter
0.0067	5.5	E9QN92	STEAP3	STEAP3 metalloreductase	Cytoplasm	transporter
0.043	5.8	Q8ZQT5				
0.029	5.9	Q8C4X2	CSNK1G3	casein kinase 1 gamma 3	Cytoplasm	kinase
0.0001	6	Q69ZN7	MYOF	myoferlin	Nucleus	other
0.012	6	Q9JIM1	SLC29A1	solute carrier family 29 member 1 (Augustine blood group)	Plasma Membrane	transporter
0.043	6.1	O89001	CPD	carboxypeptidase D	Extracellular Space	peptidase
0.0001	6.1	Q8ZMN0				
0.00014	6.2	Q8BPM0	DAAM1	dishevelled associated activator of morphogenesis 1	Cytoplasm	other
0.029	6.2	Q7CPQ6				
0.0021	6.3	A2AG68	ATP7A	ATPase copper transporting alpha	Plasma Membrane	transporter
0.0052	6.3	P17439	GBA	glucosylceramidase beta	Cytoplasm	enzyme
0.0052	6.3	B1AT66	SLC16A6	solute carrier family 16 member 6	Plasma Membrane	transporter
0.043	6.4	Q99LJ8	NUS1	NUS1, dehydrodolichyl diphosphate synthase subunit	Cytoplasm	enzyme
0.0001	6.5	G5E829	ATP2B1	ATPase plasma membrane Ca2+ transporting 1	Plasma Membrane	transporter
0.019	6.6	Q93GL9				
0.008	6.7	P61021	RAB5B	RAB5B, member RAS oncogene family	Cytoplasm	enzyme

Table S3

0.0001	6.8	P06795	Abcb1b	ATP-binding cassette, sub-family B (MDR/TAP), member 1B	Plasma Membrane	transporter
0.0001	6.9	P54987	ACOD1	aconitate decarboxylase 1	Cytoplasm	enzyme
0.0021	6.9	Q9Z1G4	ATP6V0A1	ATPase H+ transporting V0 subunit a1	Cytoplasm	transporter
0.019	6.9	Q9D486	CMIP	c-Maf inducing protein	Cytoplasm	other
0.008	7.3	Q3U7R1	ESYT1	extended synaptotagmin 1	Cytoplasm	other
0.00085	7.6	G3UYU4	FLOT1	flotillin 1	Plasma Membrane	other
0.00034	7.6	Q8ZR40				
0.0052	7.7	G5E8J0	NOTCH2	notch 2	Plasma Membrane	transcription regulator
0.012	7.8	Q60634	FLOT2	flotillin 2	Plasma Membrane	other
0.008	7.9	Q80VQ0	ALDH3B1	aldehyde dehydrogenase 3 family member B1	Cytoplasm	enzyme
0.008	7.9	P29477	NOS2	nitric oxide synthase 2	Cytoplasm	enzyme
0.00021	8.1	A2AWF9	PTPRJ	protein tyrosine phosphatase, receptor type J	Plasma Membrane	phosphatase
0.0033	8.9	Q8ZPD6				
0.00013	9	Q3UZR5	ATP2C1	ATPase secretory pathway Ca2+ transporting 1	Cytoplasm	transporter
0.00021	9.1	A0A140LIF8	IRGM	immunity related GTPase M	Cytoplasm	enzyme
0.0001	16	Q61609	SLC20A1	solute carrier family 20 member 1	Plasma Membrane	transporter
