# Supplementary material for: Antigen-encapsulating host extracellular vesicles derived from Salmonella-infected cells stimulate pathogen-specific Th1-type responses in vivo
Source: PLoS Pathog. 2021 May 6;17(5):e1009465. doi: 10.1371/journal.ppat.1009465 (PMC8101724; doi:10.1371/journal.ppat.1009465)
Supplement: S4 Table — The exosomal proteins identified by proteomics with a protein level differentially regulated by infection (48 hpi) were analyzed by Ingenuity Pathway Analysis software to identify the downstream processes that these proteins regulate. P-value and prediction of the activated state, as well as activation z-score of each function, are shown. Molecules involved in each pathway related to a particular process, as well as the number of proteins identified in our differentially expressed datasets, are also shown. (PDF) [file ppat.1009465.s017.pdf]

Table S4

Categories	Diseases or Functions Annotation	p-Value	Predicted Activation State	Activation z-score	Molecules	# Molecules
**Protein Synthesis**	Metabolism of protein	5.2E-35		1.074	ABCA3,ATP1B3,CCT2,CCT3,CCT4,CCT5,CCT6A,CCT7,CCT8,CFL1,CNDP2,CNOT1,CTSD,CTSK,DDX39B,DDX3X,DKC1,DNMT1,ECPAS,EEF1D,EEF1G,EIF2A,EIF2S1,EIF2S3,EIF3A,EIF3B,EIF3C,EIF3D,EIF3E,EIF3F,EIF3I,EIF3L,EIF3M,FARSB,FLNA,FLOT1,FLOT2,FYN,GBA,GOLGA7,HNRNPL,HSP90AA1,HSP90AB1,ICAM1,IDE,IRGM,ITCH,JAK1,LYN,MAPK3,MFGE8,MYH9,NAA15,NCBP1,NCL,NCSTN,NOS2,NPC1,NRAS,PFN1,PLAU,PPAT,PPP1CA,PPP2CA,PREP,PTPRC,RAB7A,RACK1,RNF149,RNF213,RNPEP,RPL10,RPL13,RPL14,RPL17,RPL18,RPL18A,RPL21,RPL24,RPL26,RPL27A,RPL28,RPL3,RPL4,RPL6,RPL7,RPL7A,RPL8,RPS14,RPS26,RPS27A,RPS6,RPS8,RPS9,RPSA,SARS,SHMT1,SQSTM1,STUB1,TCIRG1,TCP1,TNIP1,TPP2,TRIM25,TSG101,VIM,WARS,XPNPEP1,XPO1,YBX1	110
**Protein Synthesis**	Initiation of translation of protein	2.1E-33			DDX3X,EIF2A,EIF2S1,EIF2S3,EIF3A,EIF3B,EIF3C,EIF3D,EIF3E,EIF3F,EIF3I,EIF3L,EIF3M,NCBP1,RPL10,RPL13,RPL14,RPL17,RPL18,RPL18A,RPL21,RPL24,RPL26,RPL27A,RPL28,RPL3,RPL4,RPL6,RPL7,RPL7A,RPL8,RPS14,RPS26,RPS27A,RPS6,RPS8,RPS9,RPSA	38
**Protein Synthesis**	Translation	4E-31	Increased	2.159	CNOT1,DDX3X,DKC1,EEF1D,EEF1G,EIF2A,EIF2S1,EIF2S3,EIF3A,EIF3B,EIF3C,EIF3D,EIF3E,EIF3F,EIF3I,EIF3L,EIF3M,FARSB,NCBP1,NCL,NPC1,PPP1CA,RACK1,RPL10,RPL13,RPL14,RPL17,RPL18,RPL18A,RPL21,RPL24,RPL26,RPL27A,RPL28,RPL3,RPL4,RPL6,RPL7,RPL7A,RPL8,RPS14,RPS26,RPS27A,RPS6,RPS8,RPS9,RPSA,SARS,SHMT1,TNIP1,WARS,YBX1	52
**Protein Synthesis**	Translation of protein	8.9E-29	Increased	2.595	CNOT1,DDX3X,DKC1,EIF2A,EIF2S1,EIF2S3,EIF3A,EIF3B,EIF3C,EIF3D,EIF3E,EIF3F,EIF3I,EIF3L,EIF3M,FARSB,NCBP1,NCL,PPP1CA,RACK1,RPL10,RPL13,RPL14,RPL17,RPL18,RPL18A,RPL21,RPL24,RPL26,RPL27A,RPL28,RPL3,RPL4,RPL6,RPL7,RPL7A,RPL8,RPS14,RPS26,RPS27A,RPS6,RPS8,RPS9,RPSA,SARS,SHMT1,TNIP1,WARS,YBX1	49
**Protein Synthesis**	Synthesis of protein	5.3E-28		1.583	CNOT1,DDX39B,DDX3X,DKC1,DNMT1,EEF1D,EEF1G,EIF2A,EIF2S1,EIF2S3,EIF3A,EIF3B,EIF3C,EIF3D,EIF3E,EIF3F,EIF3I,EIF3L,EIF3M,FARSB,HNRNPL,ICAM1,LYN,MAPK3,NCBP1,NCL,NPC1,NRAS,PPAT,PPP1CA,PTPRC,RACK1,RPL10,RPL13,RPL14,RPL17,RPL18,RPL18A,RPL21,RPL24,RPL26,RPL27A,RPL28,RPL3,RPL4,RPL6,RPL7,RPL7A,RPL8,RPS14,RPS26,RPS27A,RPS6,RPS8,RPS9,RPSA,SARS,SHMT1,TNIP1,VIM,WARS,YBX1	62
**RNA Damage and Repair**	Nonsense-mediated mRNA decay	1.2E-25			EIF3E,NCBP1,PPP2CA,PPP2R1A,PPP2R2A,RPL10,RPL13,RPL14,RPL17,RPL18,RPL18A,RPL21,RPL24,RPL26,RPL27A,RPL28,RPL3,RPL4,RPL6,RPL7,RPL7A,RPL8,RPS14,RPS26,RPS27A,RPS6,RPS8,RPS9,RPSA	29
**Protein Synthesis**	Expression of protein	1.4E-25	Increased	2.763	CNOT1,DDX3X,DKC1,EIF2A,EIF2S1,EIF2S3,EIF3A,EIF3B,EIF3C,EIF3D,EIF3E,EIF3F,EIF3I,EIF3L,EIF3M,FARSB,LYN,NCBP1,NCL,PPP1CA,PTPRC,RACK1,RPL10,RPL13,RPL14,RPL17,RPL18,RPL18A,RPL21,RPL24,RPL26,RPL27A,RPL28,RPL3,RPL4,RPL6,RPL7,RPL7A,RPL8,RPS14,RPS26,RPS27A,RPS6,RPS8,RPS9,RPSA,SARS,SHMT1,TNIP1,WARS,YBX1	51
**Inflammatory Response**	Degranulation	1.5E-23		1.814	ACAA1,ACLY,ACTR2,ADAM8,ALDH3B1,ATP6V0A1,C5AR1,CAND1,CCT2,CCT8,CD36,CD47,COPB1,CORO1B,CTSD,DDX3X,DNAJC13,DOCK2,FCER1G,FERMT3,FLNA,FYN,GOLGA7,GUSB,HK3,HSP90AA1,HSP90AB1,IDH1,ITGAM,ITGB2,KPNB1,KRT1,LGALS3BP,LILRB4,LYN,MAPK3,MVP,NCSTN,NPC1,NRAS,OLA1,PDXK,PI4K2A,PKM,PLA2G4A,PLAU,PLEK,PTPRC,PTPRJ,RAB31,RAB5B,RAB5C,RAB7A,RALA,RAP1B,RAP2C,SDCBP,SIRPA,SNAP23,STAM2,STOM,TCIRG1,TNFRSF1B,TOM1,VAMP8	65
**Cellular Compromise,Inflammatory Response**	Degranulation of granulocytes	5.7E-23			ACAA1,ACLY,ACTR2,ADAM8,ALDH3B1,ATP6V0A1,C5AR1,CAND1,CCT2,CCT8,CD36,CD47,COPB1,CTSD,DDX3X,DNAJC13,DOCK2,FCER1G,GOLGA7,GUSB,HK3,HSP90AA1,HSP90AB1,IDH1,ITGAM,ITGB2,KPNB1,KRT1,MVP,NCSTN,NRAS,PDXK,PI4K2A,PKM,PLAU,PTPRC,PTPRJ,RAB31,RAB5B,RAB5C,RAB7A,RAP1B,RAP2C,SDCBP,SIRPA,SNAP23,STOM,TCIRG1,TNFRSF1B,TOM1,VAMP8	51
**Cellular Compromise,Inflammatory Response**	Degranulation of neutrophils	1.2E-22			ACAA1,ACLY,ACTR2,ADAM8,ALDH3B1,ATP6V0A1,C5AR1,CAND1,CCT2,CCT8,CD36,CD47,COPB1,CTSD,DDX3X,DNAJC13,DOCK2,FCER1G,GOLGA7,GUSB,HK3,HSP90AA1,HSP90AB1,IDH1,ITGAM,ITGB2,KPNB1,KRT1,MVP,NCSTN,NRAS,PDXK,PKM,PLAU,PTPRC,PTPRJ,RAB31,RAB5B,RAB5C,RAB7A,RAP1B,RAP2C,SDCBP,SIRPA,SNAP23,STOM,TCIRG1,TNFRSF1B,TOM1,VAMP8	50
**Cellular Compromise,Inflammatory Response**	Degranulation of phagocytes	2E-22		0.518	ACAA1,ACLY,ACTR2,ADAM8,ALDH3B1,ATP6V0A1,C5AR1,CAND1,CCT2,CCT8,CD36,CD47,COPB1,CORO1B,CTSD,DDX3X,DNAJC13,DOCK2,FCER1G,FYN,GOLGA7,GUSB,HK3,HSP90AA1,HSP90AB1,IDH1,ITGAM,ITGB2,KPNB1,KRT1,LILRB4,LYN,MAPK3,MVP,NCSTN,NRAS,PDXK,PKM,PLA2G4A,PLAU,PTPRC,PTPRJ,RAB31,RAB5B,RAB5C,RAB7A,RAP1B,RAP2C,SDCBP,SIRPA,SNAP23,STOM,TCIRG1,TNFRSF1B,TOM1,VAMP8	56
**Cellular Compromise,Inflammatory Response**	Degranulation of cells	2.7E-22		1.393	ACAA1,ACLY,ACTR2,ADAM8,ALDH3B1,ATP6V0A1,C5AR1,CAND1,CCT2,CCT8,CD36,CD47,COPB1,CORO1B,CTSD,DDX3X,DNAJC13,DOCK2,FCER1G,FERMT3,FLNA,FYN,GOLGA7,GUSB,HK3,HSP90AA1,HSP90AB1,IDH1,ITGAM,ITGB2,KPNB1,KRT1,LGALS3BP,LILRB4,LYN,MAPK3,MVP,NCSTN,NRAS,OLA1,PDXK,PI4K2A,PKM,PLA2G4A,PLAU,PLEK,PTPRC,PTPRJ,RAB31,RAB5B,RAB5C,RAB7A,RALA,RAP1B,RAP2C,SDCBP,SIRPA,SNAP23,STOM,TCIRG1,TNFRSF1B,TOM1,VAMP8	63

Table S4

**Cancer,Endocrine System Disorders,Organismal Injury and Abnormalities**	Thyroid carcinoma	7.5E-22			ABCA3,ABCE1,ABCG1,ACAA1,ACLY,ACO2,ACSL4,ACTA1,ACTR1A,ACTR2,ACTR3,ADSL,ADSSL1,AHCY,AHNAK,AKR1B1,ALCAM,ALDH9A1,AMDHD2,AP3B1,ARAP1,ARPC2,ATIC,ATP1A1,ATP1B3,ATP2B1,ATP2C1,ATP6V0A1,ATP7A,CA2,CAND1,CAPZA1,CCT2,CCT4,CCT5,CCT6A,CCT7,CCT8,CD47,CDK1,CFL1,CLUH,CMAS,CMIP,CNDP2,CNOT1,COLEC12,COPB1,COPG2,CORO1B,CORO7/CORO7-PAM16,CPD,CPNE8,CRYZ,CSE1L,CSNK1G3,CTPS1,CTSD,CTSK,DAAM1,DCTN1,DDX17,DDX21,DDX39B,DDX3X,DENND4B,DHX15,DHX29,DIS3,DKC1,DNAJA1,DNAJC13,DNM1L,DNM2,DNMT1,DOCK2,DPYSL2,ECPAS,EDIL3,EEF1G,EHD1,EIF2A,EIF2S1,EIF2S3,EIF3A,EIF3B,EIF3C,EIF3E,EIF3F,EIF3I,EIF3L,EIF3M,ENO1,ESYT1,FARSA,FARSB,FASN,FERMT3,FKBP4,FLNA,FLOT1,FLOT2,G3BP1,G6PD,GALK1,GARS,GART,GBA,GFPT1,GMDS,GMPS,GNA13,GNAI2,GNAS,GNB2,GOLGA7,GRB2,GUSB,HLA-A,HNRNPL,HNRNPM,HNRNPU,HSD17B4,HSP90AA1,HSP90AB1,HSPA4,ICAM1,IDE,IDH1,IL6ST,IPO7,ITCH,ITGAM,ITGB2,JAK1,KCNN4,KIDINS220,KPNA2,KRT1,KRT10,KRT2,KRT9,LGALS3BP,LIG1,LILRB4,LRP12,LYN,MAPK3,MARS,MAT2A,MCM2,MCM7,MEMO1,MFGE8,MOV10,MRI1,MSN,MSTO1,MTA2,MTHFD1,MVP,MYH9,MYO1C,MYO1E,MYO1G,MYOF,NAA15,NCBP1,NCL,NCSTN,NDRG1,NOP56,NOS2,NOTCH2,NPC1,NRAS,NUP93,NUS1,OASL,OLA1,PDS5A,PDXK,PFAS,PFN1,PGD,PGK1,PI4K2A,PI4K2B,PKM,PLA2G4A,PLAU,PLD3,PLEC,PLXNA1,PLXNB2,PNKP,POLD1,POLR1C,POLR2B,PPAT,PPP1CA,PPP1R7,PPP2R1A,PPP2R2A,PRPF19,PRPF4,PSMG1,PTGS2,PTPN23,PTPRA,PTPRC,PTPRJ,RAB31,RAB5A,RAB5C,RALA,RANGAP1,RARS,RBBP7,RBPJ,RCC2,RHBDF2,RIPK3,RNF149,RNF213,RNH1,RNPEP,RPF2,RPL13,RPL17,RPL18A,RPL21,RPL24,RPL26,RPL28,RPL3,RPL4,RPL6,RPL7,RPL7A,RPL8,RPN1,RPS14,RPS26,RPS6,RPS8,RPSA,RRP9,RTCB,SCAMP2,SCFD1,SDCBP,SEC24B,SF3B1,SFPQ,SHMT1,SHMT2,SIPA1,SIRPA,SLC12A4,SLC15A3,SLC16A3,SLC1A5,SLC23A2,SLC29A1,SLC2A1,SLC3A2,SLC4A7,SLC7A1,SLCO4A1,SMPDL3B,SMU1,SND1,SNRNP200,SNRNP40,SNX2,SNX27,SNX5,SQSTM1,SRM,STAM2,STAT1,STEAP3,STOM,STRAP,STUB1,STX6,SUPT5H,TALDO1,TAX1BP1,TCIRG1,TCP1,TFRC,TGFBR2,TKT,TLR7,TMEM59,TNFRSF1B,TNIP1,TOM1,TPP2,TRIM14,TRIM25,TRIM28,TRPV2,TSG101,TSR1,TSTA3,TTC37,TTLL12,UAP1L1,UBA1,UBA2,UCHL5,USP5,USP8,VASP,VIM,VPS13C,VWA5A,WARS,WDR61,WDR82,WWP2,XPNPEP1,XPO1,XPO7,YBX1,ZC3HAV1	329
**Cancer,Endocrine System Disorders,Organismal Injury and Abnormalities**	Endocrine gland tumor	1.6E-21			ABCA3,ABCE1,ABCG1,ACAA1,ACLY,ACO2,ACSL4,ACTA1,ACTR1A,ACTR2,ACTR3,ADSL,ADSSL1,AHCY,AHNAK,AKR1B1,ALCAM,ALDH9A1,AMDHD2,AP3B1,ARAP1,ARPC2,ATIC,ATP1A1,ATP1B3,ATP2B1,ATP2C1,ATP6V0A1,ATP7A,CA2,CAND1,CAPZA1,CCT2,CCT4,CCT5,CCT6A,CCT7,CCT8,CD47,CDK1,CFL1,CLUH,CMAS,CMIP,CNDP2,CNOT1,COLEC12,COPB1,COPG2,CORO1B,CORO7/CORO7-PAM16,CPD,CPNE8,CRYZ,CSE1L,CSNK1G3,CTPS1,CTSD,CTSK,DAAM1,DCTN1,DDX17,DDX21,DDX39B,DDX3X,DENND4B,DHX15,DHX29,DIS3,DKC1,DNAJA1,DNAJC13,DNM1L,DNM2,DNMT1,DOCK2,DPYSL2,ECPAS,EDIL3,EEF1G,EHD1,EIF2A,EIF2S1,EIF2S3,EIF3A,EIF3B,EIF3C,EIF3E,EIF3F,EIF3I,EIF3L,EIF3M,ENO1,ESYT1,FARSA,FARSB,FASN,FERMT3,FKBP4,FLNA,FLOT1,FLOT2,G3BP1,G6PD,GALK1,GARS,GART,GBA,GFPT1,GMDS,GMPS,GNA13,GNAI2,GNAS,GNB2,GOLGA7,GRB2,GUSB,HLA-A,HNRNPL,HNRNPM,HNRNPU,HSD17B4,HSP90AA1,HSP90AB1,HSPA4,ICAM1,IDE,IDH1,IFIT1B,IFITM3,IL6ST,IPO7,ITCH,ITGAM,ITGB2,JAK1,KCNN4,KIDINS220,KPNA2,KRT1,KRT10,KRT2,KRT9,LGALS3BP,LIG1,LILRB4,LRP12,LYN,MAPK3,MARS,MAT2A,MCM2,MCM7,MEMO1,MFGE8,MOV10,MRI1,MSN,MSTO1,MTA2,MTHFD1,MVP,MYH9,MYO1C,MYO1E,MYO1G,MYOF,NAA15,NCBP1,NCL,NCSTN,NDRG1,NOP56,NOS2,NOTCH2,NPC1,NRAS,NUP93,NUS1,OASL,OLA1,PDS5A,PDXK,PFAS,PFN1,PGD,PGK1,PI4K2A,PI4K2B,PKM,PLA2G4A,PLAU,PLD3,PLEC,PLXNA1,PLXNB2,PNKP,POLD1,POLR1C,POLR2B,PPAT,PPP1CA,PPP1R7,PPP2R1A,PPP2R2A,PRPF19,PRPF4,PSMG1,PTGS2,PTPN23,PTPRA,PTPRC,PTPRJ,RAB31,RAB5A,RAB5C,RALA,RANGAP1,RARS,RBBP7,RBPJ,RCC2,RHBDF2,RIPK3,RNF149,RNF213,RNH1,RNPEP,RPF2,RPL13,RPL17,RPL18A,RPL21,RPL24,RPL26,RPL28,RPL3,RPL4,RPL6,RPL7,RPL7A,RPL8,RPN1,RPS14,RPS26,RPS6,RPS8,RPSA,RRP9,RTCB,SCAMP2,SCFD1,SDCBP,SEC24B,SF3B1,SFPQ,SHMT1,SHMT2,SIPA1,SIRPA,SLC12A4,SLC15A3,SLC16A3,SLC1A5,SLC23A2,SLC29A1,SLC2A1,SLC3A2,SLC4A7,SLC7A1,SLCO4A1,SLFN13,SMPDL3B,SMU1,SND1,SNRNP200,SNRNP40,SNX2,SNX27,SNX5,SQSTM1,SRM,STAM2,STAT1,STEAP3,STOM,STRAP,STUB1,STX6,SUPT5H,TALDO1,TAX1BP1,TCIRG1,TCP1,TFRC,TGFBR2,TKT,TLR7,TMEM59,TNFRSF1B,TNIP1,TOM1,TPP2,TRIM14,TRIM25,TRIM28,TRPV2,TSG101,TSR1,TSTA3,TTC37,TTLL12,UAP1L1,UBA1,UBA2,UCHL5,USP5,USP8,VASP,VIM,VPS13C,VWA5A,WARS,WDR61,WDR82,WWP2,XPNPEP1,XPO1,XPO7,YBX1,ZC3HAV1	332

Table S4

**Cancer,Organismal Injury and Abnormalities**	Head and neck tumor	2.2E-20		-0.391	ABCA3,ABCE1,ABCG1,ACAA1,ACLY,ACO2,ACSL4,ACTA1,ACTR1A,ACTR2,ACTR3,ADAM8,ADSL,ADSSL1,AHCY,AHNAK,AKR1B1,ALCAM,ALDH9A1,AMDHD2,AP3B1,APEX1,ARAP1,ARPC2,ATIC,ATP1A1,ATP1B3,ATP2B1,ATP2C1,ATP6V0A1,ATP7A,CA2,CAND1,CAPZA1,CCT2,CCT3,CCT4,CCT5,CCT6A,CCT7,CCT8,CD47,CDK1,CFL1,CLUH,CMAS,CMIP,CNDP2,CNOT1,COLEC12,COPB1,COPG2,CORO1B,CORO7/CORO7-PAM16,CPD,CPNE8,CRYZ,CSE1L,CSNK1G3,CTPS1,CTSD,CTSK,DAAM1,DCTN1,DDX17,DDX21,DDX39B,DDX3X,DENND4B,DHX15,DHX29,DIS3,DKC1,DNAJA1,DNAJC13,DNM1L,DNM2,DNMT1,DOCK2,DPYSL2,ECPAS,EDIL3,EEF1G,EHD1,EIF2A,EIF2S1,EIF2S3,EIF3A,EIF3B,EIF3C,EIF3E,EIF3F,EIF3I,EIF3L,EIF3M,ENO1,ESYT1,FARSA,FARSB,FASN,FERMT3,FKBP4,FLNA,FLOT1,FLOT2,G3BP1,G6PD,GALK1,GARS,GART,GBA,GFPT1,GMDS,GMPS,GNA13,GNAI2,GNAS,GNB2,GOLGA7,GRB2,GUSB,HIST1H1C,HLA-A,HNRNPL,HNRNPM,HNRNPU,HSD17B4,HSP90AA1,HSP90AB1,HSPA4,ICAM1,IDE,IDH1,IFIT1B,IFITM3,IL6ST,IPO7,ITCH,ITGAM,ITGB2,JAK1,KCNN4,KIDINS220,KPNA2,KRT1,KRT10,KRT2,KRT9,LGALS3BP,LIG1,LILRB4,LRP12,LYN,MAPK3,MARS,MAT2A,MCM2,MCM7,MEMO1,MFGE8,MOV10,MRI1,MSN,MSTO1,MTA2,MTHFD1,MVP,MYH9,MYO1C,MYO1E,MYO1G,MYOF,NAA15,NCBP1,NCL,NCSTN,NDRG1,NOP56,NOS2,NOTCH2,NPC1,NRAS,NUP93,NUS1,OASL,OLA1,PCNA,PDS5A,PDXK,PFAS,PFN1,PGD,PGK1,PI4K2A,PI4K2B,PKM,PLA2G4A,PLAU,PLD3,PLEC,PLXNA1,PLXNB2,PNKP,POLD1,POLR1C,POLR2B,PPAT,PPP1CA,PPP1R7,PPP2R1A,PPP2R2A,PRIM2,PRPF19,PRPF4,PSMG1,PTGS2,PTPN23,PTPRA,PTPRC,PTPRJ,RAB31,RAB5A,RAB5C,RALA,RANGAP1,RARS,RBBP7,RBPJ,RCC2,RHBDF2,RIPK3,RNF149,RNF213,RNH1,RNPEP,RPF2,RPL10,RPL13,RPL14,RPL17,RPL18A,RPL21,RPL24,RPL26,RPL28,RPL3,RPL4,RPL6,RPL7,RPL7A,RPL8,RPN1,RPS14,RPS26,RPS6,RPS8,RPSA,RRP9,RTCB,SCAMP2,SCFD1,SDCBP,SEC24B,SF3B1,SFPQ,SHMT1,SHMT2,SIPA1,SIRPA,SLC12A4,SLC15A3,SLC16A3,SLC1A5,SLC23A2,SLC29A1,SLC2A1,SLC3A2,SLC4A7,SLC7A1,SLCO4A1,SMPDL3B,SMU1,SND1,SNRNP200,SNRNP40,SNX2,SNX27,SNX5,SQSTM1,SRM,STAM2,STAT1,STEAP3,STOM,STRAP,STUB1,STX6,SUPT5H,TALDO1,TAX1BP1,TCIRG1,TCP1,TFRC,TGFBR2,TKT,TLR7,TMEM59,TNFRSF1B,TNIP1,TOM1,TPP2,TRIM14,TRIM25,TRIM28,TRPV2,TSG101,TSR1,TSTA3,TTC37,TTLL12,UAP1L1,UBA1,UBA2,UCHL5,USP5,USP8,VASP,VIM,VPS13C,VWA5A,WARS,WDR61,WDR82,WWP2,XPNPEP1,XPO1,XPO7,YBX1,ZC3HAV1	339
**Cancer,Organismal Injury and Abnormalities**	Head and neck cancer	2.1E-19		-0.391	ABCA3,ABCE1,ABCG1,ACAA1,ACLY,ACO2,ACSL4,ACTA1,ACTR1A,ACTR2,ACTR3,ADAM8,ADSL,ADSSL1,AHCY,AHNAK,AKR1B1,ALCAM,ALDH9A1,AMDHD2,AP3B1,APEX1,ARAP1,ARPC2,ATIC,ATP1A1,ATP1B3,ATP2B1,ATP2C1,ATP6V0A1,ATP7A,CA2,CAND1,CAPZA1,CCT2,CCT3,CCT4,CCT5,CCT6A,CCT7,CCT8,CD47,CDK1,CFL1,CLUH,CMAS,CMIP,CNDP2,CNOT1,COLEC12,COPB1,COPG2,CORO1B,CORO7/CORO7-PAM16,CPD,CPNE8,CRYZ,CSE1L,CSNK1G3,CTPS1,CTSD,CTSK,DAAM1,DCTN1,DDX17,DDX21,DDX39B,DDX3X,DENND4B,DHX15,DHX29,DIS3,DKC1,DNAJA1,DNAJC13,DNM1L,DNM2,DNMT1,DOCK2,DPYSL2,ECPAS,EDIL3,EEF1G,EHD1,EIF2A,EIF2S1,EIF2S3,EIF3A,EIF3B,EIF3C,EIF3E,EIF3F,EIF3I,EIF3L,EIF3M,ENO1,ESYT1,FARSA,FARSB,FASN,FERMT3,FKBP4,FLNA,FLOT1,FLOT2,G3BP1,G6PD,GALK1,GARS,GART,GBA,GFPT1,GMDS,GMPS,GNA13,GNAI2,GNAS,GNB2,GOLGA7,GRB2,GUSB,HIST1H1C,HLA-A,HNRNPL,HNRNPM,HNRNPU,HSD17B4,HSP90AA1,HSP90AB1,HSPA4,ICAM1,IDE,IDH1,IL6ST,IPO7,ITCH,ITGAM,ITGB2,JAK1,KCNN4,KIDINS220,KPNA2,KRT1,KRT10,KRT2,KRT9,LGALS3BP,LIG1,LILRB4,LRP12,LYN,MAPK3,MARS,MAT2A,MCM2,MCM7,MEMO1,MFGE8,MOV10,MRI1,MSN,MSTO1,MTA2,MTHFD1,MVP,MYH9,MYO1C,MYO1E,MYO1G,MYOF,NAA15,NCBP1,NCL,NCSTN,NDRG1,NOP56,NOS2,NOTCH2,NPC1,NRAS,NUP93,NUS1,OASL,OLA1,PDS5A,PDXK,PFAS,PFN1,PGD,PGK1,PI4K2A,PI4K2B,PKM,PLA2G4A,PLAU,PLD3,PLEC,PLXNA1,PLXNB2,PNKP,POLD1,POLR1C,POLR2B,PPAT,PPP1CA,PPP1R7,PPP2R1A,PPP2R2A,PRIM2,PRPF19,PRPF4,PSMG1,PTGS2,PTPN23,PTPRA,PTPRC,PTPRJ,RAB31,RAB5A,RAB5C,RALA,RANGAP1,RARS,RBBP7,RBPJ,RCC2,RHBDF2,RIPK3,RNF149,RNF213,RNH1,RNPEP,RPF2,RPL10,RPL13,RPL14,RPL17,RPL18A,RPL21,RPL24,RPL26,RPL28,RPL3,RPL4,RPL6,RPL7,RPL7A,RPL8,RPN1,RPS14,RPS26,RPS6,RPS8,RPSA,RRP9,RTCB,SCAMP2,SCFD1,SDCBP,SEC24B,SF3B1,SFPQ,SHMT1,SHMT2,SIPA1,SIRPA,SLC12A4,SLC15A3,SLC16A3,SLC1A5,SLC23A2,SLC29A1,SLC2A1,SLC3A2,SLC4A7,SLC7A1,SLCO4A1,SMPDL3B,SMU1,SND1,SNRNP200,SNRNP40,SNX2,SNX27,SNX5,SQSTM1,SRM,STAM2,STAT1,STEAP3,STOM,STRAP,STUB1,STX6,SUPT5H,TALDO1,TAX1BP1,TCIRG1,TCP1,TFRC,TGFBR2,TKT,TLR7,TMEM59,TNFRSF1B,TNIP1,TOM1,TPP2,TRIM14,TRIM25,TRIM28,TRPV2,TSG101,TSR1,TSTA3,TTC37,TTLL12,UAP1L1,UBA1,UBA2,UCHL5,USP5,USP8,VASP,VIM,VPS13C,VWA5A,WARS,WDR61,WDR82,WWP2,XPNPEP1,XPO1,XPO7,YBX1,ZC3HAV1	336

Table S4

**Cancer,Organismal Injury and Abnormalities**	Head and neck carcinoma	5.5E-19			ABCA3,ABCE1,ABCG1,ACAA1,ACLY,ACO2,ACSL4,ACTA1,ACTR1A,ACTR2,ACTR3,ADSL,ADSSL1,AHCY,AHNAK,AKR1B1,ALCAM,ALDH9A1,AMDHD2,AP3B1,ARAP1,ARPC2,ATIC,ATP1A1,ATP1B3,ATP2B1,ATP2C1,ATP6V0A1,ATP7A,CA2,CAND1,CAPZA1,CCT2,CCT3,CCT4,CCT5,CCT6A,CCT7,CCT8,CD47,CDK1,CFL1,CLUH,CMAS,CMIP,CNDP2,CNOT1,COLEC12,COPB1,COPG2,CORO1B,CORO7/CORO7-PAM16,CPD,CPNE8,CRYZ,CSE1L,CSNK1G3,CTPS1,CTSD,CTSK,DAAM1,DCTN1,DDX17,DDX21,DDX39B,DDX3X,DENND4B,DHX15,DHX29,DIS3,DKC1,DNAJA1,DNAJC13,DNM1L,DNM2,DNMT1,DOCK2,DPYSL2,ECPAS,EDIL3,EEF1G,EHD1,EIF2A,EIF2S1,EIF2S3,EIF3A,EIF3B,EIF3C,EIF3E,EIF3F,EIF3I,EIF3L,EIF3M,ENO1,ESYT1,FARSA,FARSB,FASN,FERMT3,FKBP4,FLNA,FLOT1,FLOT2,G3BP1,G6PD,GALK1,GARS,GART,GBA,GFPT1,GMDS,GMPS,GNA13,GNAI2,GNAS,GNB2,GOLGA7,GRB2,GUSB,HIST1H1C,HLA-A,HNRNPL,HNRNPM,HNRNPU,HSD17B4,HSP90AA1,HSP90AB1,HSPA4,ICAM1,IDE,IDH1,IL6ST,IPO7,ITCH,ITGAM,ITGB2,JAK1,KCNN4,KIDINS220,KPNA2,KRT1,KRT10,KRT2,KRT9,LGALS3BP,LIG1,LILRB4,LRP12,LYN,MAPK3,MARS,MAT2A,MCM2,MCM7,MEMO1,MFGE8,MOV10,MRI1,MSN,MSTO1,MTA2,MTHFD1,MVP,MYH9,MYO1C,MYO1E,MYO1G,MYOF,NAA15,NCBP1,NCL,NCSTN,NDRG1,NOP56,NOS2,NOTCH2,NPC1,NRAS,NUP93,NUS1,OASL,OLA1,PDS5A,PDXK,PFAS,PFN1,PGD,PGK1,PI4K2A,PI4K2B,PKM,PLA2G4A,PLAU,PLD3,PLEC,PLXNA1,PLXNB2,PNKP,POLD1,POLR1C,POLR2B,PPAT,PPP1CA,PPP1R7,PPP2R1A,PPP2R2A,PRIM2,PRPF19,PRPF4,PSMG1,PTGS2,PTPN23,PTPRA,PTPRC,PTPRJ,RAB31,RAB5A,RAB5C,RALA,RANGAP1,RARS,RBBP7,RBPJ,RCC2,RHBDF2,RIPK3,RNF149,RNF213,RNH1,RNPEP,RPF2,RPL10,RPL13,RPL14,RPL17,RPL18A,RPL21,RPL24,RPL26,RPL28,RPL3,RPL4,RPL6,RPL7,RPL7A,RPL8,RPN1,RPS14,RPS26,RPS6,RPS8,RPSA,RRP9,RTCB,SCAMP2,SCFD1,SDCBP,SEC24B,SF3B1,SFPQ,SHMT1,SHMT2,SIPA1,SIRPA,SLC12A4,SLC15A3,SLC16A3,SLC1A5,SLC23A2,SLC29A1,SLC2A1,SLC3A2,SLC4A7,SLC7A1,SLCO4A1,SMPDL3B,SMU1,SND1,SNRNP200,SNRNP40,SNX2,SNX27,SNX5,SQSTM1,SRM,STAM2,STAT1,STEAP3,STOM,STRAP,STUB1,STX6,SUPT5H,TALDO1,TAX1BP1,TCIRG1,TCP1,TFRC,TGFBR2,TKT,TLR7,TMEM59,TNFRSF1B,TNIP1,TOM1,TPP2,TRIM14,TRIM25,TRIM28,TRPV2,TSG101,TSR1,TSTA3,TTC37,TTLL12,UAP1L1,UBA1,UBA2,UCHL5,USP5,USP8,VASP,VIM,VPS13C,VWA5A,WARS,WDR61,WDR82,WWP2,XPNPEP1,XPO1,XPO7,YBX1,ZC3HAV1	334
**Cancer,Organismal Injury and Abnormalities**	Cancer of secretory structure	6.4E-18		0.218	ABCA3,ABCE1,ABCG1,ACAA1,ACLY,ACO2,ACSL4,ACTA1,ACTR1A,ACTR2,ACTR3,ADSL,ADSSL1,AHCY,AHNAK,AKR1B1,ALCAM,ALDH9A1,AMDHD2,AP3B1,APEX1,ARAP1,ARPC2,ATIC,ATP1A1,ATP1B3,ATP2B1,ATP2C1,ATP6V0A1,ATP7A,C5AR1,CA2,CAND1,CAPZA1,CCT2,CCT4,CCT5,CCT6A,CCT7,CCT8,CD47,CDK1,CFL1,CLUH,CMAS,CMIP,CNDP2,CNOT1,COLEC12,COPB1,COPG2,CORO1B,CORO7/CORO7-PAM16,CPD,CPNE8,CRYZ,CSE1L,CSNK1G3,CTPS1,CTSD,CTSK,DAAM1,DCTN1,DDX17,DDX21,DDX39B,DDX3X,DENND4B,DHX15,DHX29,DIS3,DKC1,DNAJA1,DNAJC13,DNM1L,DNM2,DNMT1,DOCK2,DPYSL2,ECPAS,EDIL3,EEF1G,EHD1,EIF2A,EIF2S1,EIF2S3,EIF3A,EIF3B,EIF3C,EIF3E,EIF3F,EIF3I,EIF3L,EIF3M,ENO1,ESYT1,FARSA,FARSB,FASN,FERMT3,FKBP4,FLNA,FLOT1,FLOT2,FYN,G3BP1,G6PD,GALK1,GARS,GART,GBA,GFPT1,GMDS,GMPS,GNA13,GNAI2,GNAS,GNB2,GOLGA7,GPNMB,GRB2,GUSB,HK3,HLA-A,HNRNPL,HNRNPM,HNRNPU,HSD17B4,HSP90AA1,HSP90AB1,HSPA4,ICAM1,IDE,IDH1,IL6ST,IPO5,IPO7,ITCH,ITGAM,ITGB2,JAK1,KCNN4,KIDINS220,KPNA2,KRT1,KRT10,KRT2,KRT9,LGALS3BP,LIG1,LILRB4,LRP12,LYN,MAPK3,MARS,MAT2A,MCM2,MCM7,MDH1,MEMO1,MFGE8,MOV10,MRI1,MSN,MSTO1,MTA2,MTHFD1,MVP,MYADM,MYH9,MYO1C,MYO1E,MYO1G,MYOF,NAA15,NANS,NCBP1,NCL,NCSTN,NDRG1,NOP56,NOS2,NOTCH2,NPC1,NRAS,NUP93,NUS1,OASL,OLA1,PCNA,PDS5A,PDXK,PFAS,PFN1,PGD,PGK1,PHGDH,PI4K2A,PI4K2B,PKM,PLA2G4A,PLAU,PLD3,PLEC,PLEK,PLXNA1,PLXNB2,PNKP,POLD1,POLR1C,POLR2B,PPAT,PPP1CA,PPP1R7,PPP2CA,PPP2R1A,PPP2R2A,PRIM2,PRPF19,PRPF4,PSMG1,PTGS2,PTPN23,PTPRA,PTPRC,PTPRJ,RAB31,RAB5A,RAB5C,RALA,RANGAP1,RARS,RBBP7,RBPJ,RCC2,RHBDF2,RIPK3,RNF149,RNF213,RNH1,RNPEP,RPF2,RPL13,RPL17,RPL18A,RPL21,RPL24,RPL26,RPL28,RPL3,RPL4,RPL6,RPL7,RPL7A,RPL8,RPN1,RPS14,RPS26,RPS27A,RPS6,RPS8,RPSA,RRP9,RTCB,SCAMP2,SCFD1,SDCBP,SEC24B,SF3B1,SFPQ,SHMT1,SHMT2,SIPA1,SIRPA,SLC12A4,SLC15A3,SLC16A3,SLC1A5,SLC23A2,SLC29A1,SLC2A1,SLC3A2,SLC4A7,SLC7A1,SLCO4A1,SMPDL3B,SMU1,SND1,SNRNP200,SNRNP40,SNX2,SNX27,SNX5,SQSTM1,SRM,STAM2,STAT1,STEAP3,STOM,STRAP,STUB1,STX6,SUPT5H,TALDO1,TAX1BP1,TCIRG1,TCP1,TFRC,TGFBR2,TKT,TLR7,TMEM59,TNFRSF1B,TNIP1,TOM1,TPP2,TRIM14,TRIM25,TRIM28,TRPV2,TSG101,TSR1,TSTA3,TTC37,TTLL12,UAP1L1,UBA1,UBA2,UCHL5,USP5,USP8,VASP,VIM,VPS13C,VWA5A,WARS,WDR61,WDR82,WWP2,XPNPEP1,XPO1,XPO7,YBX1,ZC3HAV1	344

Table S4

**Cancer,Organismal Injury and Abnormalities**	Carcinoma	9.2E-18		0.261	ABCA3,ABCE1,ABCG1,ACAA1,ACLY,ACO2,ACSL4,ACTA1,ACTR1A,ACTR2,ACTR3,ADAM8,ADSL,ADSSL1,AHCY,AHNAK,AKR1B1,AKR1B10,ALCAM,ALDH9A1,AMDHD2,AP3B1,APEX1,ARAP1,ARPC2,ATIC,ATP1A1,ATP1B3,ATP2B1,ATP2C1,ATP5F1B,ATP6V0A1,ATP7A,C5AR1,CA2,CAND1,CAPZA1,CCT2,CCT3,CCT4,CCT5,CCT6A,CCT7,CCT8,CD36,CD47,CDK1,CFL1,CLUH,CMAS,CMIP,CNDP2,CNOT1,COLEC12,COPB1,COPG1,COPG2,CORO1B,CORO1C,CORO7/CORO7-PAM16,CPD,CPNE8,CRYZ,CSE1L,CSNK1G3,CTPS1,CTSD,CTSK,DAAM1,DCTN1,DDX17,DDX21,DDX39B,DDX3X,DENND4B,DHX15,DHX29,DIS3,DKC1,DNAJA1,DNAJC13,DNM1L,DNM2,DNMT1,DOCK2,DPYSL2,ECPAS,EDIL3,EEF1D,EEF1G,EHD1,EIF2A,EIF2S1,EIF2S3,EIF3A,EIF3B,EIF3C,EIF3D,EIF3E,EIF3F,EIF3I,EIF3L,EIF3M,ENO1,ESYT1,FARSA,FARSB,FASN,FERMT3,FKBP4,FLNA,FLOT1,FLOT2,FYN,G3BP1,G6PD,GALK1,GARS,GART,GBA,GFPT1,GMDS,GMPS,GNA13,GNAI2,GNAI3,GNAS,GNB2,GOLGA7,GPNMB,GRB2,GUSB,HIST1H1C,HIST1H2AJ,HK3,HLA-A,HNRNPL,HNRNPM,HNRNPU,HSD17B4,HSP90AA1,HSP90AB1,HSPA4,ICAM1,IDE,IDH1,IFIT1B,IFITM3,IL6ST,IPO5,IPO7,ITCH,ITGAM,ITGB2,JAK1,KCNN4,KIDINS220,KPNA2,KPNB1,KRT1,KRT10,KRT2,KRT9,LGALS3BP,LIG1,LILRB4,LRP12,LYN,MAPK3,MARS,MAT2A,MAT2B,MCM2,MCM7,MDH1,MEMO1,MFGE8,MOV10,MPEG1,MRI1,MSN,MSTO1,MTA2,MTHFD1,MVP,MYADM,MYH9,MYO1C,MYO1E,MYO1G,MYOF,NAA15,NANS,NCBP1,NCF2,NCL,NCSTN,NDRG1,NOP56,NOS2,NOTCH2,NPC1,NRAS,NUP93,NUS1,OASL,OLA1,PCNA,PDS5A,PDXK,PFAS,PFN1,PGD,PGK1,PHGDH,PI4K2A,PI4K2B,PKM,PLA2G4A,PLAU,PLD3,PLEC,PLEK,PLXNA1,PLXNB2,PNKP,POLD1,POLR1C,POLR2A,POLR2B,PPAT,PPP1CA,PPP1R7,PPP2CA,PPP2R1A,PPP2R2A,PREP,PRIM2,PRPF19,PRPF4,PSMG1,PTGS2,PTPN23,PTPRA,PTPRC,PTPRJ,RAB31,RAB5A,RAB5B,RAB5C,RAB8B,RACK1,RALA,RAN,RANGAP1,RAP1B,RAP2C,RARS,RBBP7,RBPJ,RCC2,RHBDF2,RIPK3,RNF149,RNF213,RNH1,RNMT,RNPEP,RPF2,RPL10,RPL13,RPL14,RPL17,RPL18,RPL18A,RPL21,RPL24,RPL26,RPL28,RPL3,RPL4,RPL6,RPL7,RPL7A,RPL8,RPN1,RPS14,RPS26,RPS27A,RPS6,RPS8,RPSA,RRP9,RTCB,SARS,SCAMP2,SCFD1,SDCBP,SEC24B,SF3B1,SFPQ,SHMT1,SHMT2,SIPA1,SIRPA,SLC12A4,SLC15A3,SLC16A3,SLC16A6,SLC1A5,SLC20A1,SLC23A2,SLC29A1,SLC2A1,SLC38A2,SLC3A2,SLC4A7,SLC7A1,SLCO4A1,SLFN13,SMPDL3B,SMU1,SNAP23,SND1,SNRNP200,SNRNP40,SNX2,SNX27,SNX5,SPRED1,SQSTM1,SRM,STAM2,STAT1,STEAP3,STOM,STRAP,STUB1,STX6,SUPT5H,TALDO1,TAX1BP1,TCIRG1,TCP1,TFRC,TGFBR2,TKT,TLR7,TMEM59,TNFRSF1B,TNIP1,TOM1,TPI1,TPP2,TRIM14,TRIM25,TRIM28,TRPV2,TSG101,TSR1,TSTA3,TTC37,TTLL12,UAP1L1,UBA1,UBA2,UCHL5,USP5,USP8,VAMP8,VASP,VIM,VPS13C,VWA5A,WARS,WDR61,WDR82,WWP2,XPNPEP1,XPO1,XPO7,YBX1,ZC3HAV1	383
**Cancer,Organismal Injury and Abnormalities**	Tumorigenesis of tissue	1.4E-17		-0.365	ABCA3,ABCE1,ABCG1,ACAA1,ACLY,ACO2,ACSL4,ACTA1,ACTR1A,ACTR2,ACTR3,ADAM8,ADSL,ADSSL1,AHCY,AHNAK,AKR1B1,AKR1B10,ALCAM,ALDH9A1,AMDHD2,AP3B1,APEX1,ARAP1,ARPC2,ATIC,ATP1A1,ATP1B3,ATP2B1,ATP2C1,ATP5F1B,ATP6V0A1,ATP7A,C5AR1,CA2,CAND1,CAPZA1,CCT2,CCT3,CCT4,CCT5,CCT6A,CCT7,CCT8,CD36,CD47,CDK1,CFL1,CLUH,CMAS,CMIP,CNDP2,CNOT1,COLEC12,COPB1,COPG1,COPG2,CORO1B,CORO1C,CORO7/CORO7-PAM16,CPD,CPNE8,CRYZ,CSE1L,CSNK1G3,CTPS1,CTSD,CTSK,DAAM1,DCTN1,DDX17,DDX21,DDX39B,DDX3X,DENND4B,DHX15,DHX29,DIS3,DKC1,DNAJA1,DNAJC13,DNM1L,DNM2,DNMT1,DOCK2,DPYSL2,ECPAS,EDIL3,EEF1D,EEF1G,EHD1,EIF2A,EIF2S1,EIF2S3,EIF3A,EIF3B,EIF3C,EIF3D,EIF3E,EIF3F,EIF3I,EIF3L,EIF3M,ENO1,ESYT1,FARSA,FARSB,FASN,FCER1G,FERMT3,FKBP4,FLNA,FLOT1,FLOT2,FYN,G3BP1,G6PD,GALK1,GARS,GART,GBA,GFPT1,GMDS,GMPS,GNA13,GNAI2,GNAI3,GNAS,GNB2,GOLGA7,GPNMB,GRB2,GUSB,HIST1H1C,HIST1H2AJ,HK3,HLA-A,HNRNPL,HNRNPM,HNRNPU,HSD17B4,HSP90AA1,HSP90AB1,HSPA4,ICAM1,IDE,IDH1,IFIT1B,IFITM3,IL6ST,IPO5,IPO7,ITCH,ITGAM,ITGB2,JAK1,KCNN4,KIDINS220,KPNA2,KPNB1,KRT1,KRT10,KRT2,KRT9,LGALS3BP,LIG1,LILRB4,LRP12,LYN,MAPK3,MARS,MAT2A,MAT2B,MCM2,MCM7,MDH1,MEMO1,MFGE8,MOV10,MPEG1,MRI1,MSN,MSTO1,MTA2,MTHFD1,MVP,MYADM,MYH9,MYO1C,MYO1E,MYO1G,MYOF,NAA15,NANS,NCBP1,NCF2,NCL,NCSTN,NDRG1,NOP56,NOS2,NOTCH2,NPC1,NRAS,NUP93,NUS1,OASL,OLA1,PCNA,PDS5A,PDXK,PFAS,PFN1,PGD,PGK1,PHGDH,PI4K2A,PI4K2B,PKM,PLA2G4A,PLAU,PLD3,PLEC,PLEK,PLXNA1,PLXNB2,PNKP,POLD1,POLR1C,POLR2A,POLR2B,PPAT,PPP1CA,PPP1R7,PPP2CA,PPP2R1A,PPP2R2A,PREP,PRIM2,PRPF19,PRPF4,PSMG1,PTGS2,PTPN23,PTPRA,PTPRC,PTPRJ,RAB31,RAB5A,RAB5B,RAB5C,RAB8B,RACK1,RALA,RAN,RANGAP1,RAP1B,RAP2C,RARS,RBBP7,RBPJ,RCC2,RHBDF2,RIPK3,RNF149,RNF213,RNH1,RNMT,RNPEP,RPF2,RPL10,RPL13,RPL14,RPL17,RPL18,RPL18A,RPL21,RPL24,RPL26,RPL28,RPL3,RPL4,RPL6,RPL7,RPL7A,RPL8,RPN1,RPS14,RPS26,RPS27A,RPS6,RPS8,RPSA,RRP9,RTCB,SARS,SCAMP2,SCFD1,SDCBP,SEC24B,SF3B1,SFPQ,SHMT1,SHMT2,SIPA1,SIRPA,SLC12A4,SLC15A3,SLC16A3,SLC16A6,SLC1A5,SLC20A1,SLC23A2,SLC29A1,SLC2A1,SLC38A2,SLC3A2,SLC4A7,SLC7A1,SLCO4A1,SLFN13,SMPDL3B,SMU1,SNAP23,SND1,SNRNP200,SNRNP40,SNX2,SNX27,SNX5,SPRED1,SQSTM1,SRM,STAM2,STAT1,STEAP3,STOM,STRAP,STUB1,STX6,SUPT5H,TALDO1,TAX1BP1,TCIRG1,TCP1,TFRC,TGFBR2,TKT,TLR7,TMEM59,TNFRSF1B,TNIP1,TOM1,TPI1,TPP2,TRIM14,TRIM25,TRIM28,TRPV2,TSG101,TSR1,TSTA3,TTC37,TTLL12,UAP1L1,UBA1,UBA2,UCHL5,USP5,USP8,VAMP8,VASP,VIM,VPS13C,VWA5A,WARS,WDR61,WDR82,WWP2,XPNPEP1,XPO1,XPO7,YBX1,ZC3HAV1	384

Table S4

**Organismal Survival**	Morbidity or mortality	2.8E-17		-1.233	ABCA3,Abcb1b,ACACA,ACLY,ACTA1,ACTL6A,APEX1,ATP1A1,ATP2B1,ATP2C1,C5AR1,CD36,CD47,CDK1,CFL1,CLUH,CSE1L,CTSD,DCTN1,DDX17,DDX3X,DKC1,DNM1L,DNM2,DNMT1,DOCK2,EHD1,EIF2S1,EIF3D,EIF3M,FASN,FCER1G,FERMT3,FKBP4,FLNA,FYN,G6PD,GBA,GNA13,GNAI2,GNAI3,GNAS,GPNMB,GRB2,HIST1H1C,HLA-A,HNRNPL,HSD17B4,HSP90AA1,HSP90AB1,ICAM1,IDE,IDH1,IL6ST,Irgm1,ITGAM,ITGB2,JAK1,KIDINS220,KRT1,LIG1,LYN,MAPK3,MCM2,MFGE8,MSN,MTA2,MYH9,NCSTN,NDRG1,NOS2,NOTCH2,NPC1,NRAS,NUS1,OASL,OLA1,PCNA,PDS5A,PFN1,PHGDH,PKM,PLAU,PLEC,PLXNB2,POLD1,POLR2A,PPP2CA,PPP2R1A,PRPF19,PSMG1,PTGS2,PTPRC,PTPRJ,RAB31,RAB5A,RAB7A,RAB8B,RALA,RBPJ,RHBDF2,RIPK3,RNMT,RPL24,RPL4,RPL6,RPSA,SF3B1,SHMT2,SLC20A1,SLC23A2,SLC2A1,SLC3A2,SLC7A1,SNAP23,SNX2,SNX27,SPRED1,SQSTM1,STAM2,STAT1,STUB1,TAX1BP1,TCIRG1,TFRC,TGFBR2,TKT,TLR7,TNFRSF1B,TNIP1,TPP2,TRIM28,TRPV2,TSG101,TSTA3,USP8,VASP,VIM,WWP2,YBX1	140
**Organismal Survival**	Organismal death	6E-17		-1.525	ABCA3,Abcb1b,ACACA,ACLY,ACTA1,ACTL6A,APEX1,ATP1A1,ATP2B1,ATP2C1,C5AR1,CD36,CD47,CDK1,CFL1,CLUH,CSE1L,CTSD,DCTN1,DDX17,DDX3X,DKC1,DNM1L,DNM2,DNMT1,DOCK2,EHD1,EIF2S1,EIF3D,EIF3M,FASN,FCER1G,FERMT3,FKBP4,FLNA,FYN,G6PD,GBA,GNA13,GNAI2,GNAI3,GNAS,GPNMB,GRB2,HIST1H1C,HNRNPL,HSD17B4,HSP90AA1,HSP90AB1,ICAM1,IDE,IDH1,IL6ST,Irgm1,ITGAM,ITGB2,JAK1,KIDINS220,KRT1,LIG1,LYN,MAPK3,MCM2,MFGE8,MSN,MTA2,MYH9,NCSTN,NDRG1,NOS2,NOTCH2,NPC1,NRAS,NUS1,OLA1,PCNA,PDS5A,PFN1,PHGDH,PKM,PLAU,PLEC,PLXNB2,POLD1,POLR2A,PPP2CA,PPP2R1A,PRPF19,PSMG1,PTGS2,PTPRC,PTPRJ,RAB31,RAB5A,RAB7A,RAB8B,RALA,RBPJ,RHBDF2,RIPK3,RNMT,RPL24,RPL4,RPL6,RPSA,SF3B1,SHMT2,SLC20A1,SLC23A2,SLC2A1,SLC3A2,SLC7A1,SNAP23,SNX2,SNX27,SPRED1,SQSTM1,STAM2,STAT1,STUB1,TAX1BP1,TCIRG1,TFRC,TGFBR2,TKT,TLR7,TNFRSF1B,TNIP1,TPP2,TRIM28,TRPV2,TSG101,TSTA3,USP8,VASP,VIM,WWP2,YBX1	138
**Immunological Disease**	Systemic autoimmune syndrome	6.7E-17		1.197	ABCG1,ACLY,ACTA1,ACTL6A,ADAM8,AKR1B1,ATIC,ATP1A1,ATP2B1,ATP2C1,C5AR1,CA2,CD36,CD47,DDX39B,DNM1L,DNMT1,EEF1G,EIF3C,EIF3D,EIF3E,EIF3L,ENO1,FCER1G,FLOT1,FYN,GNAS,GNB2,GPNMB,GUSB,HELZ2,HLA-A,ICAM1,IDE,Ifi202b,IFIT1B,IL6ST,IRGM,Irgm1,ITGAM,ITGB2,JAK1,LGALS3BP,LILRB4,LYN,MAPK3,MPEG1,MTA2,MYH9,MYO1C,NCF2,NRAS,OASL,PFAS,PGK1,PLEK,POLD1,PPAT,PPP1CA,PPP1R7,PPP2CA,PREP,PTGS2,PTPRC,RAB31,RAB5A,RAB5B,RIPK3,RNF149,RPL18,RPL18A,RPSA,SEC24B,SLC3A2,SLC7A1,SND1,SNRNP200,SPRED1,STAT1,TALDO1,TCIRG1,TFRC,TGFBR2,TLR7,TNFRSF1B,TPP2,TRIM25,TRIM28,VIM,WARS	90
**Cancer,Organismal Injury and Abnormalities**	Nonhematologic malignant neoplasm	7.1E-17		0.192	ABCA3,ABCE1,ABCG1,ACAA1,ACLY,ACO2,ACSL4,ACTA1,ACTL6A,ACTR1A,ACTR2,ACTR3,ADAM8,ADSL,ADSSL1,AHCY,AHNAK,AKR1B1,AKR1B10,ALCAM,ALDH9A1,AMDHD2,AP3B1,APEX1,ARAP1,ARPC2,ATIC,ATP1A1,ATP1B3,ATP2B1,ATP2C1,ATP5F1B,ATP6V0A1,ATP7A,C5AR1,CA2,CAND1,CAPZA1,CCT2,CCT3,CCT4,CCT5,CCT6A,CCT7,CCT8,CD36,CD47,CDK1,CFL1,CLUH,CMAS,CMIP,CNDP2,CNOT1,COLEC12,COPB1,COPG1,COPG2,CORO1B,CORO1C,CORO7/CORO7-PAM16,CPD,CPNE8,CRYZ,CSE1L,CSNK1G3,CTPS1,CTSD,CTSK,DAAM1,DCTN1,DDX17,DDX21,DDX39B,DDX3X,DENND4B,DHX15,DHX29,DIS3,DKC1,DNAJA1,DNAJC13,DNM1L,DNM2,DNMT1,DOCK2,DPYSL2,ECPAS,EDIL3,EEF1D,EEF1G,EHD1,EIF2A,EIF2S1,EIF2S3,EIF3A,EIF3B,EIF3C,EIF3D,EIF3E,EIF3F,EIF3I,EIF3L,EIF3M,ENO1,ESYT1,FARSA,FARSB,FASN,FERMT3,FKBP4,FLNA,FLOT1,FLOT2,FYN,G3BP1,G6PD,GALK1,GARS,GART,GBA,GFPT1,GMDS,GMPS,GNA13,GNAI2,GNAI3,GNAS,GNB2,GOLGA7,GPNMB,GRB2,GUSB,HIST1H1C,HIST1H2AJ,HK3,HLA-A,HNRNPL,HNRNPM,HNRNPU,HSD17B4,HSP90AA1,HSP90AB1,HSPA4,ICAM1,IDE,IDH1,IFIT1B,IFITM3,IL6ST,IPO5,IPO7,ITCH,ITGAM,ITGB2,JAK1,KCNN4,KIDINS220,KPNA2,KPNB1,KRT1,KRT10,KRT2,KRT9,LGALS3BP,LIG1,LILRB4,LRP12,LYN,MAPK3,MARS,MAT2A,MAT2B,MCM2,MCM7,MDH1,MEMO1,MFGE8,MOV10,MPEG1,MRI1,MSN,MSTO1,MTA2,MTHFD1,MVP,MYADM,MYH9,MYO1C,MYO1E,MYO1G,MYOF,NAA15,NANS,NCBP1,NCF2,NCL,NCSTN,NDRG1,NOP56,NOS2,NOTCH2,NPC1,NRAS,NUP93,NUS1,OASL,OLA1,OSGEP,PCNA,PDS5A,PDXK,PFAS,PFN1,PGD,PGK1,PHGDH,PI4K2A,PI4K2B,PKM,PLA2G4A,PLAU,PLD3,PLEC,PLEK,PLXNA1,PLXNB2,PNKP,POLD1,POLR1C,POLR2A,POLR2B,PPAT,PPP1CA,PPP1R7,PPP2CA,PPP2R1A,PPP2R2A,PREP,PRIM2,PRPF19,PRPF4,PSMG1,PTGS2,PTPN23,PTPRA,PTPRC,PTPRJ,RAB31,RAB5A,RAB5B,RAB5C,RAB8B,RACK1,RALA,RAN,RANGAP1,RAP1B,RAP2C,RARS,RBBP7,RBPJ,RCC2,RHBDF2,RIPK3,RNF149,RNF213,RNH1,RNMT,RNPEP,RPF2,RPL10,RPL13,RPL14,RPL17,RPL18,RPL18A,RPL21,RPL24,RPL26,RPL28,RPL3,RPL4,RPL6,RPL7,RPL7A,RPL8,RPN1,RPS14,RPS26,RPS27A,RPS6,RPS8,RPSA,RRP9,RTCB,SARS,SCAMP2,SCFD1,SDCBP,SEC24B,SF3B1,SFPQ,SHMT1,SHMT2,SIPA1,SIRPA,SLC12A4,SLC15A3,SLC16A3,SLC16A6,SLC1A5,SLC20A1,SLC23A2,SLC29A1,SLC2A1,SLC38A2,SLC3A2,SLC4A7,SLC7A1,SLCO4A1,SLFN13,SMPDL3B,SMU1,SNAP23,SND1,SNRNP200,SNRNP40,SNX2,SNX27,SNX5,SPRED1,SQSTM1,SRM,STAM2,STAT1,STEAP3,STOM,STRAP,STUB1,STX6,SUPT5H,TALDO1,TAX1BP1,TCIRG1,TCP1,TFRC,TGFBR2,TKT,TLR7,TMEM59,TNFRSF1B,TNIP1,TOM1,TPI1,TPP2,TRIM14,TRIM25,TRIM28,TRPV2,TSG101,TSR1,TSTA3,TTC37,TTLL12,UAP1L1,UBA1,UBA2,UCHL5,USP5,USP8,VAMP8,VASP,VIM,VPS13C,VWA5A,WARS,WDR61,WDR82,WWP2,XPNPEP1,XPO1,XPO7,YBX1,ZC3HAV1	385
**Cellular Function and Maintenance**	Endocytosis	1E-16	Increased	2.226	ATP5F1B,CD36,CD47,COLEC12,CORO1C,DNM2,DOCK2,DPYSL2,EHD1,FCER1G,FLNA,FLOT1,FYN,GRB2,Hist1h1a,HSP90AA1,ICAM1,IFITM3,ITGAM,ITGB2,LYN,MAPK3,MFGE8,MYH9,MYO1E,MYO1G,NCL,NPC1,PFN1,PLA2G4A,PLAU,PTPRC,PTPRJ,RAB31,RAB5A,RAB5B,RAB5C,RAB7A,RACK1,RALA,SCAMP2,SFPQ,SIRPA,SNAP23,SNX5,STAM2,TFRC,TGFBR2,TRPV2,VAMP8,VIM	51

Table S4

**Cellular Movement**	Cell movement	2.9E-16	Increased	2.245	Abcb1b,ABCG1,ACO2,ACTR3,ADAM8,AHCY,AHNAK,AKR1B1,ALCAM,ARAP1,ARPC2,ATP1B3,ATP5F1B,C5AR1,CA2,CD36,CD47,CDK1,CFL1,CORO1B,CORO1C,CSE1L,DNAJA1,DNM2,DNMT1,DOCK2,DPYSL2,EDIL3,EHD1,EIF3E,ENO1,FASN,FCER1G,FERMT3,FKBP4,FLNA,FLOT1,FYN,G6PD,GBA,GNA13,GNAI2,GNAI3,GNAS,GNB2,GPNMB,GRB2,HLA-A,HNRNPL,HSP90AA1,HSP90AB1,ICAM1,IDH1,IL6ST,Irgm1,ITGAM,ITGB2,JAK1,KCNN4,KPNA2,KRT10,KRT2,LRP12,LYN,MAPK3,MCM2,MCM7,MSN,MYADM,MYH9,MYO1C,MYO1E,MYO1G,NCF2,NCL,NDRG1,NOS2,NPC1,NRAS,NUS1,PFN1,PKM,PLA2G4A,PLAU,PLEC,PLXNA1,PLXNB2,PTGS2,PTPN23,PTPRA,PTPRC,PTPRJ,RAB5A,RACK1,RALA,RAP1B,RAP2C,RCC2,RNH1,RPSA,SDCBP,SEC24B,SIRPA,SLC16A3,SLC3A2,STAT1,STX6,TAX1BP1,TCIRG1,TGFBR2,TKT,TLR7,TNFRSF1B,TNIP1,TPI1,TRPV2,VASP,VIM,WARS,YBX1	120
**Cell Death and Survival**	Necrosis	6.2E-16		1.188	ABCG1,ACLY,ADAM8,AKR1B1,APEX1,ATP1A1,ATP1B3,ATP2C1,ATP7A,C5AR1,CCT2,CD36,CD47,CDK1,COPG1,CSE1L,CTSD,DNM1L,EDIL3,EEF1D,EIF2A,EIF2S1,EIF3B,EIF3C,EIF3E,EIF3F,EIF3L,ENO1,FASN,FCER1G,FKBP4,FLNA,FYN,G6PD,GBA,GFPT1,GNAI2,GNAS,HIST1H1C,HLA-A,HSP90AA1,HSP90AB1,HSPA4,ICAM1,IDE,Ifi202b,IL6ST,Irgm1,ITGAM,ITGB2,JAK1,KIDINS220,KPNB1,LIG1,LYN,MAPK3,MCM2,MDH1,MFGE8,MTA2,MVP,NCF2,NCL,NDRG1,NOS2,NOTCH2,NPC1,NRAS,PCNA,PFN1,PKM,PLA2G4A,PLAU,PLEC,PPP2CA,PTGS2,PTPRA,PTPRC,RACK1,RAN,RBPJ,RIPK3,RPL10,RPL13,RPL27A,RPL3,RPL6,RPL7,RPL7A,RPS14,RPS27A,RPS6,RPSA,SF3B1,SIRPA,SLC20A1,SLC29A1,SLC2A1,STAM2,STAT1,STEAP3,STUB1,TCP1,TFRC,TGFBR2,TLR7,TNFRSF1B,TNIP1,TPP2,TRIM28,TSG101,UCHL5,VIM,WWP2,XPO1,YBX1	116
**Cancer,Organismal Injury and Abnormalities**	Extracranial solid tumor	3.2E-15		0.733	ABCA3,ABCE1,ABCG1,ACAA1,ACLY,ACO2,ACSL4,ACTA1,ACTR1A,ACTR2,ACTR3,ADAM8,ADSL,ADSSL1,AHCY,AHNAK,AKR1B1,AKR1B10,ALCAM,ALDH9A1,AMDHD2,AP3B1,APEX1,ARAP1,ARPC2,ATIC,ATP1A1,ATP1B3,ATP2B1,ATP2C1,ATP5F1B,ATP6V0A1,ATP7A,C5AR1,CA2,CAND1,CAPZA1,CCT2,CCT3,CCT4,CCT5,CCT6A,CCT7,CCT8,CD36,CD47,CDK1,CFL1,CLUH,CMAS,CMIP,CNDP2,CNOT1,COLEC12,COPB1,COPG1,COPG2,CORO1B,CORO1C,CORO7/CORO7-PAM16,CPD,CPNE8,CRYZ,CSE1L,CSNK1G3,CTPS1,CTSD,CTSK,DAAM1,DCTN1,DDX17,DDX21,DDX39B,DDX3X,DENND4B,DHX15,DHX29,DIS3,DKC1,DNAJA1,DNAJC13,DNM1L,DNM2,DNMT1,DOCK2,DPYSL2,ECPAS,EDIL3,EEF1D,EEF1G,EHD1,EIF2A,EIF2S1,EIF2S3,EIF3A,EIF3B,EIF3C,EIF3D,EIF3E,EIF3F,EIF3I,EIF3L,EIF3M,ENO1,ESYT1,FARSA,FARSB,FASN,FERMT3,FKBP4,FLNA,FLOT1,FLOT2,FYN,G3BP1,G6PD,GALK1,GARS,GART,GBA,GFPT1,GMDS,GMPS,GNA13,GNAI2,GNAI3,GNAS,GNB2,GOLGA7,GPNMB,GRB2,GUSB,HIST1H1C,HIST1H2AJ,HK3,HLA-A,HNRNPL,HNRNPM,HNRNPU,HSD17B4,HSP90AA1,HSP90AB1,HSPA4,ICAM1,IDE,IDH1,Ifi202b,IFIT1B,IFITM3,IL6ST,IPO5,IPO7,Irgm1,ITCH,ITGAM,ITGB2,JAK1,KCNN4,KIDINS220,KPNA2,KPNB1,KRT1,KRT10,KRT2,KRT9,LGALS3BP,LIG1,LILRB4,LRP12,LYN,MAPK3,MARS,MAT2A,MAT2B,MCM2,MCM7,MDH1,MEMO1,MFGE8,MOV10,MPEG1,MRI1,MSN,MSTO1,MTA2,MTHFD1,MVP,MYADM,MYH9,MYO1C,MYO1E,MYO1G,MYOF,NAA15,NANS,NCBP1,NCF2,NCL,NCSTN,NDRG1,NOP56,NOS2,NOTCH2,NPC1,NRAS,NUP93,NUS1,OASL,OLA1,OSGEP,PCNA,PDS5A,PDXK,PFAS,PFN1,PGD,PGK1,PHGDH,PI4K2A,PI4K2B,PKM,PLA2G4A,PLAU,PLD3,PLEC,PLEK,PLXNA1,PLXNB2,PNKP,POLD1,POLR1C,POLR2A,POLR2B,PPAT,PPP1CA,PPP1R7,PPP2CA,PPP2R1A,PPP2R2A,PREP,PRIM2,PRPF19,PRPF4,PSMG1,PTGS2,PTPN23,PTPRA,PTPRC,PTPRJ,RAB31,RAB5A,RAB5B,RAB5C,RAB8B,RACK1,RALA,RAN,RANGAP1,RAP1B,RAP2C,RARS,RBBP7,RBPJ,RCC2,RHBDF2,RIPK3,RNF149,RNF213,RNH1,RNMT,RNPEP,RPF2,RPL10,RPL13,RPL14,RPL17,RPL18,RPL18A,RPL21,RPL24,RPL26,RPL27A,RPL28,RPL3,RPL4,RPL6,RPL7,RPL7A,RPL8,RPN1,RPS14,RPS26,RPS27A,RPS6,RPS8,RPSA,RRP9,RTCB,SARS,SCAMP2,SCFD1,SDCBP,SEC24B,SF3B1,SFPQ,SHMT1,SHMT2,SIPA1,SIRPA,SLC12A4,SLC15A3,SLC16A3,SLC16A6,SLC1A5,SLC20A1,SLC23A2,SLC29A1,SLC2A1,SLC38A2,SLC3A2,SLC4A7,SLC7A1,SLCO4A1,SLFN13,SMPDL3B,SMU1,SNAP23,SND1,SNRNP200,SNRNP40,SNX2,SNX27,SNX5,SPRED1,SQSTM1,SRM,STAM2,STAT1,STEAP3,STOM,STRAP,STUB1,STX6,SUPT5H,TALDO1,TAX1BP1,TCIRG1,TCP1,TFRC,TGFBR2,TKT,TLR7,TMEM59,TNFRSF1B,TNIP1,TOM1,TPI1,TPP2,TRIM14,TRIM25,TRIM28,TRPV2,TSG101,TSR1,TSTA3,TTC37,TTLL12,UAP1L1,UBA1,UBA2,UCHL5,USP5,USP8,VAMP8,VASP,VIM,VPS13C,VWA5A,WARS,WDR61,WDR82,WWP2,XPNPEP1,XPO1,XPO7,YBX1,ZC3HAV1	387
**Inflammatory Response**	Immune response of cells	3.6E-15	Increased	2.541	Abcb1b,C5AR1,CD36,CD47,COLEC12,CORO1C,DNM2,DOCK2,EHD1,FCER1G,FLNA,FYN,GNAS,GRB2,HLA-A,HSP90AA1,ICAM1,IL6ST,ITGAM,ITGB2,JAK1,LILRB4,LYN,MFGE8,MYH9,MYO1G,NOS2,PFN1,PLA2G4A,PLAU,PLXNA1,PTPRC,PTPRJ,RAB31,RAB5B,RAB8B,RACK1,RALA,SIRPA,SLC1A5,SNAP23,STAT1,TCIRG1,TGFBR2,TLR7,TNFRSF1B,TRPV2,VIM	48

Table S4

**Cancer,Organismal Injury and Abnormalities**	Cancer	5.8E-15		0.701	ABCA3,ABCE1,ABCG1,ACAA1,ACLY,ACO2,ACSL4,ACTA1,ACTL6A,ACTR1A,ACTR2,ACTR3,ADAM8,ADSL,ADSSL1,AHCY,AHNAK,AKR1B1,AKR1B10,ALCAM,ALDH9A1,AMDHD2,AP3B1,APEX1,ARAP1,ARPC2,ATIC,ATP1A1,ATP1B3,ATP2B1,ATP2C1,ATP5F1B,ATP6V0A1,ATP7A,C5AR1,CA2,CAND1,CAPZA1,CCT2,CCT3,CCT4,CCT5,CCT6A,CCT7,CCT8,CD36,CD47,CDK1,CFL1,CLUH,CMAS,CMIP,CNDP2,CNOT1,COLEC12,COPB1,COPG1,COPG2,CORO1B,CORO1C,CORO7/CORO7-PAM16,CPD,CPNE8,CRYZ,CSE1L,CSNK1G3,CTPS1,CTSD,CTSK,DAAM1,DCTN1,DDX17,DDX21,DDX39B,DDX3X,DENND4B,DHX15,DHX29,DIS3,DKC1,DNAJA1,DNAJC13,DNM1L,DNM2,DNMT1,DOCK2,DPYSL2,ECPAS,EDIL3,EEF1D,EEF1G,EHD1,EIF2A,EIF2S1,EIF2S3,EIF3A,EIF3B,EIF3C,EIF3D,EIF3E,EIF3F,EIF3I,EIF3L,EIF3M,ENO1,ESYT1,FARSA,FARSB,FASN,FCER1G,FERMT3,FKBP4,FLNA,FLOT1,FLOT2,FYN,G3BP1,G6PD,GALK1,GARS,GART,GBA,GFPT1,GMDS,GMPS,GNA13,GNAI2,GNAI3,GNAS,GNB2,GOLGA7,GPNMB,GRB2,GUSB,HIST1H1C,HIST1H2AJ,HK3,HLA-A,HNRNPL,HNRNPM,HNRNPU,HSD17B4,HSP90AA1,HSP90AB1,HSPA4,ICAM1,IDE,IDH1,Ifi202b,IFIT1B,IFITM3,IL6ST,IPO5,IPO7,ITCH,ITGAM,ITGB2,JAK1,KCNN4,KIDINS220,KPNA2,KPNB1,KRT1,KRT10,KRT2,KRT9,LGALS3BP,LIG1,LILRB4,LRP12,LYN,MAPK3,MARS,MAT2A,MAT2B,MCM2,MCM7,MDH1,MEMO1,MFGE8,MOV10,MPEG1,MRI1,MSN,MSTO1,MTA2,MTHFD1,MVP,MYADM,MYH9,MYO1C,MYO1E,MYO1G,MYOF,NAA15,NANS,NCBP1,NCF2,NCL,NCSTN,NDRG1,NOP56,NOS2,NOTCH2,NPC1,NRAS,NUP93,NUS1,OASL,OLA1,OSGEP,PCNA,PDS5A,PDXK,PFAS,PFN1,PGD,PGK1,PHGDH,PI4K2A,PI4K2B,PKM,PLA2G4A,PLAU,PLD3,PLEC,PLEK,PLXNA1,PLXNB2,PNKP,POLD1,POLR1C,POLR2A,POLR2B,PPAT,PPP1CA,PPP1R7,PPP2CA,PPP2R1A,PPP2R2A,PREP,PRIM2,PRPF19,PRPF4,PSMG1,PTGS2,PTPN23,PTPRA,PTPRC,PTPRJ,RAB31,RAB5A,RAB5B,RAB5C,RAB8B,RACK1,RALA,RAN,RANGAP1,RAP1B,RAP2C,RARS,RBBP7,RBPJ,RCC2,RHBDF2,RIPK3,RNF149,RNF213,RNH1,RNMT,RNPEP,RPF2,RPL10,RPL13,RPL14,RPL17,RPL18,RPL18A,RPL21,RPL24,RPL26,RPL27A,RPL28,RPL3,RPL4,RPL6,RPL7,RPL7A,RPL8,RPN1,RPS14,RPS26,RPS27A,RPS6,RPS8,RPSA,RRP9,RTCB,SARS,SCAMP2,SCFD1,SDCBP,SEC24B,SF3B1,SFPQ,SHMT1,SHMT2,SIPA1,SIRPA,SLC12A4,SLC15A3,SLC16A3,SLC16A6,SLC1A5,SLC20A1,SLC23A2,SLC29A1,SLC2A1,SLC38A2,SLC3A2,SLC4A7,SLC7A1,SLCO4A1,SLFN13,SMPDL3B,SMU1,SNAP23,SND1,SNRNP200,SNRNP40,SNX2,SNX27,SNX5,SPRED1,SQSTM1,SRM,STAM2,STAT1,STEAP3,STOM,STRAP,STUB1,STX6,SUPT5H,TALDO1,TAX1BP1,TCIRG1,TCP1,TFRC,TGFBR2,TKT,TLR7,TMEM59,TNFRSF1B,TNIP1,TOM1,TPI1,TPP2,TRIM14,TRIM25,TRIM28,TRPV2,TSG101,TSR1,TSTA3,TTC37,TTLL12,UAP1L1,UBA1,UBA2,UCHL5,USP5,USP8,VAMP8,VASP,VIM,VPS13C,VWA5A,WARS,WDR61,WDR82,WWP2,XPNPEP1,XPO1,XPO7,YBX1,ZC3HAV1	388
**Cancer,Organismal Injury and Abnormalities**	Solid tumor	9.3E-15		0.285	ABCA3,ABCE1,ABCG1,ACAA1,ACLY,ACO2,ACSL4,ACTA1,ACTL6A,ACTR1A,ACTR2,ACTR3,ADAM8,ADSL,ADSSL1,AHCY,AHNAK,AKR1B1,AKR1B10,ALCAM,ALDH9A1,AMDHD2,AP3B1,APEX1,ARAP1,ARPC2,ATIC,ATP1A1,ATP1B3,ATP2B1,ATP2C1,ATP5F1B,ATP6V0A1,ATP7A,C5AR1,CA2,CAND1,CAPZA1,CCT2,CCT3,CCT4,CCT5,CCT6A,CCT7,CCT8,CD36,CD47,CDK1,CFL1,CLUH,CMAS,CMIP,CNDP2,CNOT1,COLEC12,COPB1,COPG1,COPG2,CORO1B,CORO1C,CORO7/CORO7-PAM16,CPD,CPNE8,CRYZ,CSE1L,CSNK1G3,CTPS1,CTSD,CTSK,DAAM1,DCTN1,DDX17,DDX21,DDX39B,DDX3X,DENND4B,DHX15,DHX29,DIS3,DKC1,DNAJA1,DNAJC13,DNM1L,DNM2,DNMT1,DOCK2,DPYSL2,ECPAS,EDIL3,EEF1D,EEF1G,EHD1,EIF2A,EIF2S1,EIF2S3,EIF3A,EIF3B,EIF3C,EIF3D,EIF3E,EIF3F,EIF3I,EIF3L,EIF3M,ENO1,ESYT1,FARSA,FARSB,FASN,FERMT3,FKBP4,FLNA,FLOT1,FLOT2,FYN,G3BP1,G6PD,GALK1,GARS,GART,GBA,GFPT1,GMDS,GMPS,GNA13,GNAI2,GNAI3,GNAS,GNB2,GOLGA7,GPNMB,GRB2,GUSB,HIST1H1C,HIST1H2AJ,HK3,HLA-A,HNRNPL,HNRNPM,HNRNPU,HSD17B4,HSP90AA1,HSP90AB1,HSPA4,ICAM1,IDE,IDH1,Ifi202b,IFIT1B,IFITM3,IL6ST,IPO5,IPO7,Irgm1,ITCH,ITGAM,ITGB2,JAK1,KCNN4,KIDINS220,KPNA2,KPNB1,KRT1,KRT10,KRT2,KRT9,LGALS3BP,LIG1,LILRB4,LRP12,LYN,MAPK3,MARS,MAT2A,MAT2B,MCM2,MCM7,MDH1,MEMO1,MFGE8,MOV10,MPEG1,MRI1,MSN,MSTO1,MTA2,MTHFD1,MVP,MYADM,MYH9,MYO1C,MYO1E,MYO1G,MYOF,NAA15,NANS,NCBP1,NCF2,NCL,NCSTN,NDRG1,NOP56,NOS2,NOTCH2,NPC1,NRAS,NUP93,NUS1,OASL,OLA1,OSGEP,PCNA,PDS5A,PDXK,PFAS,PFN1,PGD,PGK1,PHGDH,PI4K2A,PI4K2B,PKM,PLA2G4A,PLAU,PLD3,PLEC,PLEK,PLXNA1,PLXNB2,PNKP,POLD1,POLR1C,POLR2A,POLR2B,PPAT,PPP1CA,PPP1R7,PPP2CA,PPP2R1A,PPP2R2A,PREP,PRIM2,PRPF19,PRPF4,PSMG1,PTGS2,PTPN23,PTPRA,PTPRC,PTPRJ,RAB31,RAB5A,RAB5B,RAB5C,RAB8B,RACK1,RALA,RAN,RANGAP1,RAP1B,RAP2C,RARS,RBBP7,RBPJ,RCC2,RHBDF2,RIPK3,RNF149,RNF213,RNH1,RNMT,RNPEP,RPF2,RPL10,RPL13,RPL14,RPL17,RPL18,RPL18A,RPL21,RPL24,RPL26,RPL27A,RPL28,RPL3,RPL4,RPL6,RPL7,RPL7A,RPL8,RPN1,RPS14,RPS26,RPS27A,RPS6,RPS8,RPSA,RRP9,RTCB,SARS,SCAMP2,SCFD1,SDCBP,SEC24B,SF3B1,SFPQ,SHMT1,SHMT2,SIPA1,SIRPA,SLC12A4,SLC15A3,SLC16A3,SLC16A6,SLC1A5,SLC20A1,SLC23A2,SLC29A1,SLC2A1,SLC38A2,SLC3A2,SLC4A7,SLC7A1,SLCO4A1,SLFN13,SMPDL3B,SMU1,SNAP23,SND1,SNRNP200,SNRNP40,SNX2,SNX27,SNX5,SPRED1,SQSTM1,SRM,STAM2,STAT1,STEAP3,STOM,STRAP,STUB1,STX6,SUPT5H,TALDO1,TAX1BP1,TCIRG1,TCP1,TFRC,TGFBR2,TKT,TLR7,TMEM59,TNFRSF1B,TNIP1,TOM1,TPI1,TPP2,TRIM14,TRIM25,TRIM28,TRPV2,TSG101,TSR1,TSTA3,TTC37,TTLL12,UAP1L1,UBA1,UBA2,UCHL5,USP5,USP8,VAMP8,VASP,VIM,VPS13C,VWA5A,WARS,WDR61,WDR82,WWP2,XPNPEP1,XPO1,XPO7,YBX1,ZC3HAV1	388

Table S4

**Cancer,Organismal Injury and Abnormalities**	Malignant solid tumor	1.5E-14		-0.002	ABCA3,ABCE1,ABCG1,ACAA1,ACLY,ACO2,ACSL4,ACTA1,ACTL6A,ACTR1A,ACTR2,ACTR3,ADAM8,ADSL,ADSSL1,AHCY,AHNAK,AKR1B1,AKR1B10,ALCAM,ALDH9A1,AMDHD2,AP3B1,APEX1,ARAP1,ARPC2,ATIC,ATP1A1,ATP1B3,ATP2B1,ATP2C1,ATP5F1B,ATP6V0A1,ATP7A,C5AR1,CA2,CAND1,CAPZA1,CCT2,CCT3,CCT4,CCT5,CCT6A,CCT7,CCT8,CD36,CD47,CDK1,CFL1,CLUH,CMAS,CMIP,CNDP2,CNOT1,COLEC12,COPB1,COPG1,COPG2,CORO1B,CORO1C,CORO7/CORO7-PAM16,CPD,CPNE8,CRYZ,CSE1L,CSNK1G3,CTPS1,CTSD,CTSK,DAAM1,DCTN1,DDX17,DDX21,DDX39B,DDX3X,DENND4B,DHX15,DHX29,DIS3,DKC1,DNAJA1,DNAJC13,DNM1L,DNM2,DNMT1,DOCK2,DPYSL2,ECPAS,EDIL3,EEF1D,EEF1G,EHD1,EIF2A,EIF2S1,EIF2S3,EIF3A,EIF3B,EIF3C,EIF3D,EIF3E,EIF3F,EIF3I,EIF3L,EIF3M,ENO1,ESYT1,FARSA,FARSB,FASN,FERMT3,FKBP4,FLNA,FLOT1,FLOT2,FYN,G3BP1,G6PD,GALK1,GARS,GART,GBA,GFPT1,GMDS,GMPS,GNA13,GNAI2,GNAI3,GNAS,GNB2,GOLGA7,GPNMB,GRB2,GUSB,HIST1H1C,HIST1H2AJ,HK3,HLA-A,HNRNPL,HNRNPM,HNRNPU,HSD17B4,HSP90AA1,HSP90AB1,HSPA4,ICAM1,IDE,IDH1,IFIT1B,IFITM3,IL6ST,IPO5,IPO7,ITCH,ITGAM,ITGB2,JAK1,KCNN4,KIDINS220,KPNA2,KPNB1,KRT1,KRT10,KRT2,KRT9,LGALS3BP,LIG1,LILRB4,LRP12,LYN,MAPK3,MARS,MAT2A,MAT2B,MCM2,MCM7,MDH1,MEMO1,MFGE8,MOV10,MPEG1,MRI1,MSN,MSTO1,MTA2,MTHFD1,MVP,MYADM,MYH9,MYO1C,MYO1E,MYO1G,MYOF,NAA15,NANS,NCBP1,NCF2,NCL,NCSTN,NDRG1,NOP56,NOS2,NOTCH2,NPC1,NRAS,NUP93,NUS1,OASL,OLA1,OSGEP,PCNA,PDS5A,PDXK,PFAS,PFN1,PGD,PGK1,PHGDH,PI4K2A,PI4K2B,PKM,PLA2G4A,PLAU,PLD3,PLEC,PLEK,PLXNA1,PLXNB2,PNKP,POLD1,POLR1C,POLR2A,POLR2B,PPAT,PPP1CA,PPP1R7,PPP2CA,PPP2R1A,PPP2R2A,PREP,PRIM2,PRPF19,PRPF4,PSMG1,PTGS2,PTPN23,PTPRA,PTPRC,PTPRJ,RAB31,RAB5A,RAB5B,RAB5C,RAB8B,RACK1,RALA,RAN,RANGAP1,RAP1B,RAP2C,RARS,RBBP7,RBPJ,RCC2,RHBDF2,RIPK3,RNF149,RNF213,RNH1,RNMT,RNPEP,RPF2,RPL10,RPL13,RPL14,RPL17,RPL18,RPL18A,RPL21,RPL24,RPL26,RPL27A,RPL28,RPL3,RPL4,RPL6,RPL7,RPL7A,RPL8,RPN1,RPS14,RPS26,RPS27A,RPS6,RPS8,RPSA,RRP9,RTCB,SARS,SCAMP2,SCFD1,SDCBP,SEC24B,SF3B1,SFPQ,SHMT1,SHMT2,SIPA1,SIRPA,SLC12A4,SLC15A3,SLC16A3,SLC16A6,SLC1A5,SLC20A1,SLC23A2,SLC29A1,SLC2A1,SLC38A2,SLC3A2,SLC4A7,SLC7A1,SLCO4A1,SLFN13,SMPDL3B,SMU1,SNAP23,SND1,SNRNP200,SNRNP40,SNX2,SNX27,SNX5,SPRED1,SQSTM1,SRM,STAM2,STAT1,STEAP3,STOM,STRAP,STUB1,STX6,SUPT5H,TALDO1,TAX1BP1,TCIRG1,TCP1,TFRC,TGFBR2,TKT,TLR7,TMEM59,TNFRSF1B,TNIP1,TOM1,TPI1,TPP2,TRIM14,TRIM25,TRIM28,TRPV2,TSG101,TSR1,TSTA3,TTC37,TTLL12,UAP1L1,UBA1,UBA2,UCHL5,USP5,USP8,VAMP8,VASP,VIM,VPS13C,VWA5A,WARS,WDR61,WDR82,WWP2,XPNPEP1,XPO1,XPO7,YBX1,ZC3HAV1	386
**Gene Expression**	Expression of mRNA	2.8E-14		0.928	CD47,CNOT1,DDX3X,EIF2A,EIF2S1,EIF2S3,EIF3B,EIF3C,EIF3D,EIF3E,EIF3F,EIF3I,EIF3L,EIF3M,FARSB,JAK1,NCBP1,PFN1,PPP1CA,RACK1,RPL10,RPL24,RPL27A,RPS14,RPS9,SARS,SHMT1,STAT1,TNIP1,WARS	30
**Gene Expression, Protein Synthesis**	Translation of mRNA	3.2E-14		1	CNOT1,DDX3X,EIF2A,EIF2S1,EIF2S3,EIF3B,EIF3C,EIF3D,EIF3E,EIF3F,EIF3I,EIF3L,EIF3M,FARSB,NCBP1,PPP1CA,RACK1,RPL10,RPL24,RPL27A,RPS14,RPS9,SARS,SHMT1,TNIP1,WARS	26
**Cellular Movement**	Migration of cells	3.9E-14	Increased	2.498	Abcb1b,ABCG1,ACTR3,ADAM8,AHCY,AHNAK,ALCAM,ARPC2,ATP1B3,ATP5F1B,C5AR1,CD36,CD47,CDK1,CFL1,CORO1C,CSE1L,DNM2,DNMT1,DOCK2,DPYSL2,EDIL3,EHD1,EIF3E,FASN,FCER1G,FERMT3,FKBP4,FLNA,FLOT1,FYN,G6PD,GBA,GNA13,GNAI2,GNAI3,GNAS,GPNMB,GRB2,HLA-A,HNRNPL,HSP90AA1,HSP90AB1,ICAM1,IDH1,IL6ST,Irgm1,ITGAM,ITGB2,JAK1,KCNN4,KPNA2,KRT10,KRT2,LRP12,LYN,MAPK3,MCM2,MCM7,MSN,MYADM,MYH9,MYO1C,MYO1G,NCF2,NCL,NDRG1,NOS2,NPC1,NRAS,NUS1,PFN1,PKM,PLA2G4A,PLAU,PLEC,PLXNA1,PLXNB2,PTGS2,PTPRA,PTPRC,PTPRJ,RAB5A,RACK1,RALA,RAP1B,RAP2C,RCC2,RNH1,RPSA,SDCBP,SIRPA,SLC16A3,SLC3A2,STAT1,STX6,TAX1BP1,TCIRG1,TGFBR2,TLR7,TNFRSF1B,TNIP1,TRPV2,VASP,VIM,WARS,YBX1	107

Table S4

**Cancer,Gastrointestinal Disease,Organismal Injury and Abnormalities**	Digestive organ tumor	2.2E-13		-0.955	ABCA3,ABCE1,ABCG1,ACAA1,ACLY,ACO2,ACSL4,ACTA1,ACTR1A,ACTR2,ACTR3,ADSL,ADSSL1,AHCY,AHNAK,AKR1B1,AKR1B10,ALCAM,ALDH9A1,AMDHD2,AP3B1,APEX1,ARAP1,ATIC,ATP1A1,ATP1B3,ATP2B1,ATP2C1,ATP6V0A1,ATP7A,C5AR1,CA2,CAND1,CCT2,CCT4,CCT5,CCT6A,CCT7,CCT8,CD36,CD47,CDK1,CFL1,CLUH,CMAS,CMIP,CNDP2,CNOT1,COLEC12,COPB1,COPG1,CORO1B,CORO1C,CORO7/CORO7-PAM16,CPD,CPNE8,CRYZ,CSE1L,CSNK1G3,CTPS1,CTSD,CTSK,DAAM1,DCTN1,DDX17,DDX21,DDX39B,DDX3X,DENND4B,DHX15,DHX29,DIS3,DKC1,DNAJA1,DNAJC13,DNM1L,DNM2,DNMT1,DOCK2,DPYSL2,ECPAS,EDIL3,EEF1D,EEF1G,EHD1,EIF2A,EIF2S3,EIF3A,EIF3B,EIF3C,EIF3D,EIF3E,EIF3F,EIF3I,EIF3L,EIF3M,ENO1,ESYT1,FARSA,FARSB,FASN,FERMT3,FKBP4,FLNA,FLOT1,FLOT2,FYN,G3BP1,G6PD,GALK1,GARS,GART,GFPT1,GMDS,GMPS,GNA13,GNAI2,GNAS,GNB2,GPNMB,GRB2,GUSB,HIST1H1C,HK3,HLA-A,HNRNPL,HNRNPM,HNRNPU,HSD17B4,HSP90AA1,HSP90AB1,HSPA4,ICAM1,IDE,IDH1,IFIT1B,IFITM3,IL6ST,IPO5,IPO7,ITCH,ITGAM,ITGB2,JAK1,KCNN4,KIDINS220,KPNA2,KPNB1,KRT1,KRT10,KRT2,KRT9,LGALS3BP,LIG1,LILRB4,LRP12,LYN,MAPK3,MARS,MAT2A,MAT2B,MCM2,MCM7,MDH1,MFGE8,MOV10,MRI1,MSN,MSTO1,MTA2,MTHFD1,MVP,MYADM,MYH9,MYO1C,MYO1E,MYO1G,MYOF,NAA15,NANS,NCBP1,NCF2,NCL,NCSTN,NDRG1,NOP56,NOS2,NOTCH2,NPC1,NRAS,NUP93,OASL,OLA1,OSGEP,PCNA,PDS5A,PDXK,PFAS,PFN1,PGK1,PHGDH,PI4K2A,PKM,PLA2G4A,PLAU,PLD3,PLEC,PLXNA1,PLXNB2,PNKP,POLD1,POLR1C,POLR2B,PPP1CA,PPP2CA,PPP2R1A,PPP2R2A,PREP,PRIM2,PRPF4,PTGS2,PTPN23,PTPRA,PTPRC,PTPRJ,RAB31,RAB5A,RAB5B,RAB8B,RACK1,RALA,RAN,RANGAP1,RAP1B,RAP2C,RARS,RBBP7,RBPJ,RCC2,RHBDF2,RIPK3,RNF213,RNH1,RNMT,RNPEP,RPF2,RPL10,RPL17,RPL18,RPL18A,RPL21,RPL26,RPL27A,RPL28,RPL3,RPL4,RPL6,RPL7,RPL7A,RPL8,RPN1,RPS14,RPS27A,RPS6,RPSA,RRP9,RTCB,SARS,SCAMP2,SCFD1,SEC24B,SF3B1,SFPQ,SHMT1,SHMT2,SIPA1,SIRPA,SLC12A4,SLC15A3,SLC16A3,SLC16A6,SLC1A5,SLC20A1,SLC23A2,SLC29A1,SLC2A1,SLC38A2,SLC3A2,SLC4A7,SLC7A1,SLCO4A1,SLFN13,SMPDL3B,SMU1,SNAP23,SND1,SNRNP200,SNX2,SNX27,SPRED1,SQSTM1,SRM,STAM2,STAT1,STEAP3,STOM,STRAP,STUB1,STX6,SUPT5H,TALDO1,TAX1BP1,TCIRG1,TCP1,TFRC,TGFBR2,TKT,TLR7,TMEM59,TNFRSF1B,TNIP1,TOM1,TPI1,TPP2,TRIM14,TRIM25,TRIM28,TRPV2,TSG101,TTC37,TTLL12,UAP1L1,UBA1,UBA2,UCHL5,USP5,USP8,VASP,VIM,VPS13C,VWA5A,WARS,WDR82,WWP2,XPNPEP1,XPO1,XPO7,YBX1,ZC3HAV1	349
**Cellular Function and Maintenance,Inflammatory Response**	Phagocytosis	7.5E-13	Increased	2.502	CD36,CD47,COLEC12,CORO1C,DNM2,DOCK2,EHD1,FCER1G,FLNA,FYN,GRB2,ICAM1,ITGAM,ITGB2,LYN,MFGE8,MSN,MYH9,MYO1G,PFN1,PLA2G4A,PLAU,PTPRC,PTPRJ,RAB31,RAB5A,RAB7A,RACK1,RALA,SIRPA,SNAP23,TRPV2,VIM	33
**Cancer,Organismal Injury and Abnormalities**	Abdominal carcinoma	9.2E-13		-0.383	ABCA3,ABCE1,ABCG1,ACAA1,ACLY,ACO2,ACSL4,ACTA1,ACTR1A,ACTR2,ACTR3,ADAM8,ADSL,ADSSL1,AHCY,AHNAK,AKR1B1,AKR1B10,ALCAM,ALDH9A1,AP3B1,APEX1,ARAP1,ARPC2,ATIC,ATP1A1,ATP1B3,ATP2B1,ATP2C1,ATP6V0A1,ATP7A,C5AR1,CA2,CAND1,CAPZA1,CCT2,CCT3,CCT4,CCT5,CCT6A,CCT7,CCT8,CD36,CD47,CDK1,CFL1,CLUH,CMAS,CMIP,CNDP2,CNOT1,COLEC12,COPB1,COPG1,CORO1B,CORO1C,CORO7/CORO7-PAM16,CPD,CPNE8,CSE1L,CSNK1G3,CTPS1,CTSD,CTSK,DAAM1,DCTN1,DDX17,DDX21,DDX39B,DDX3X,DENND4B,DHX15,DHX29,DIS3,DKC1,DNAJA1,DNAJC13,DNM1L,DNM2,DNMT1,DOCK2,DPYSL2,ECPAS,EDIL3,EEF1D,EEF1G,EHD1,EIF2S1,EIF2S3,EIF3A,EIF3B,EIF3C,EIF3D,EIF3E,EIF3F,EIF3I,EIF3L,EIF3M,ENO1,ESYT1,FARSA,FARSB,FASN,FERMT3,FKBP4,FLNA,FLOT1,FLOT2,FYN,G3BP1,G6PD,GALK1,GARS,GART,GFPT1,GMDS,GMPS,GNA13,GNAI2,GNAS,GNB2,GPNMB,GRB2,GUSB,HIST1H1C,HIST1H2AJ,HK3,HLA-A,HNRNPL,HNRNPM,HNRNPU,HSD17B4,HSP90AA1,HSP90AB1,HSPA4,ICAM1,IDE,IDH1,IFIT1B,IFITM3,IL6ST,IPO5,IPO7,ITCH,ITGAM,ITGB2,JAK1,KCNN4,KIDINS220,KPNA2,KPNB1,KRT1,KRT10,KRT2,KRT9,LGALS3BP,LIG1,LILRB4,LRP12,LYN,MAPK3,MARS,MAT2B,MCM2,MCM7,MDH1,MEMO1,MFGE8,MOV10,MRI1,MSN,MSTO1,MTA2,MTHFD1,MVP,MYADM,MYH9,MYO1C,MYO1E,MYO1G,MYOF,NAA15,NANS,NCBP1,NCF2,NCL,NCSTN,NOP56,NOS2,NOTCH2,NPC1,NRAS,NUP93,OASL,OLA1,PCNA,PDS5A,PDXK,PFAS,PFN1,PGK1,PHGDH,PI4K2A,PKM,PLA2G4A,PLAU,PLD3,PLEC,PLEK,PLXNA1,PLXNB2,PNKP,POLD1,POLR1C,POLR2B,PPP1CA,PPP1R7,PPP2CA,PPP2R1A,PPP2R2A,PREP,PRIM2,PRPF4,PTGS2,PTPN23,PTPRA,PTPRC,PTPRJ,RAB5A,RAB5B,RAB8B,RACK1,RALA,RAN,RANGAP1,RAP1B,RAP2C,RARS,RBBP7,RBPJ,RCC2,RHBDF2,RIPK3,RNF149,RNF213,RNH1,RNMT,RNPEP,RPF2,RPL10,RPL14,RPL17,RPL18,RPL28,RPL3,RPL4,RPL6,RPL7A,RPL8,RPN1,RPS14,RPS27A,RPS6,RPSA,RRP9,RTCB,SARS,SCAMP2,SCFD1,SEC24B,SF3B1,SFPQ,SHMT1,SHMT2,SIPA1,SIRPA,SLC12A4,SLC15A3,SLC16A3,SLC16A6,SLC1A5,SLC20A1,SLC23A2,SLC29A1,SLC2A1,SLC38A2,SLC3A2,SLC4A7,SLC7A1,SLCO4A1,SLFN13,SMPDL3B,SMU1,SNAP23,SND1,SNRNP200,SNX2,SNX27,SPRED1,SQSTM1,SRM,STAM2,STAT1,STEAP3,STOM,STRAP,STUB1,STX6,SUPT5H,TALDO1,TAX1BP1,TCIRG1,TCP1,TFRC,TGFBR2,TKT,TLR7,TMEM59,TNFRSF1B,TNIP1,TOM1,TPI1,TPP2,TRIM14,TRIM25,TRIM28,TRPV2,TSG101,TSR1,TTC37,TTLL12,UAP1L1,UBA1,UBA2,UCHL5,USP5,USP8,VAMP8,VASP,VIM,VPS13C,VWA5A,WARS,WDR82,WWP2,XPNPEP1,XPO1,XPO7,YBX1,ZC3HAV1	350

Table S4

**Cancer,Organismal Injury and Abnormalities**	Abdominal neoplasm	1.3E-12		0.234	ABCA3,ABCE1,ABCG1,ACAA1,ACLY,ACO2,ACSL4,ACTA1,ACTR1A,ACTR2,ACTR3,ADAM8,ADSL,ADSSL1,AHCY,AHNAK,AKR1B1,AKR1B10,ALCAM,ALDH9A1,AP3B1,APEX1,ARAP1,ARPC2,ATIC,ATP1A1,ATP1B3,ATP2B1,ATP2C1,ATP6V0A1,ATP7A,C5AR1,CA2,CAND1,CAPZA1,CCT2,CCT3,CCT4,CCT5,CCT6A,CCT7,CCT8,CD36,CD47,CDK1,CFL1,CLUH,CMAS,CMIP,CNDP2,CNOT1,COLEC12,COPB1,COPG1,CORO1B,CORO1C,CORO7/CORO7-PAM16,CPD,CPNE8,CRYZ,CSE1L,CSNK1G3,CTPS1,CTSD,CTSK,DAAM1,DCTN1,DDX17,DDX21,DDX39B,DDX3X,DENND4B,DHX15,DHX29,DIS3,DKC1,DNAJA1,DNAJC13,DNM1L,DNM2,DNMT1,DOCK2,DPYSL2,ECPAS,EDIL3,EEF1D,EEF1G,EHD1,EIF2A,EIF2S1,EIF2S3,EIF3A,EIF3B,EIF3C,EIF3D,EIF3E,EIF3F,EIF3I,EIF3L,EIF3M,ENO1,ESYT1,FARSA,FARSB,FASN,FERMT3,FKBP4,FLNA,FLOT1,FLOT2,FYN,G3BP1,G6PD,GALK1,GARS,GART,GFPT1,GMDS,GMPS,GNA13,GNAI2,GNAS,GNB2,GPNMB,GRB2,GUSB,HIST1H1C,HIST1H2AJ,HK3,HLA-A,HNRNPL,HNRNPM,HNRNPU,HSD17B4,HSP90AA1,HSP90AB1,HSPA4,ICAM1,IDE,IDH1,IFIT1B,IFITM3,IL6ST,IPO5,IPO7,ITCH,ITGAM,ITGB2,JAK1,KCNN4,KIDINS220,KPNA2,KPNB1,KRT1,KRT10,KRT2,KRT9,LGALS3BP,LIG1,LILRB4,LRP12,LYN,MAPK3,MARS,MAT2A,MAT2B,MCM2,MCM7,MDH1,MEMO1,MFGE8,MOV10,MRI1,MSN,MSTO1,MTA2,MTHFD1,MVP,MYADM,MYH9,MYO1C,MYO1E,MYO1G,MYOF,NAA15,NANS,NCBP1,NCF2,NCL,NCSTN,NDRG1,NOP56,NOS2,NOTCH2,NPC1,NRAS,NUP93,OASL,OLA1,OSGEP,PCNA,PDS5A,PDXK,PFAS,PFN1,PGK1,PHGDH,PI4K2A,PKM,PLA2G4A,PLAU,PLD3,PLEC,PLEK,PLXNA1,PLXNB2,PNKP,POLD1,POLR1C,POLR2B,PPAT,PPP1CA,PPP1R7,PPP2CA,PPP2R1A,PPP2R2A,PREP,PRIM2,PRPF4,PTGS2,PTPN23,PTPRA,PTPRC,PTPRJ,RAB31,RAB5A,RAB5B,RAB8B,RACK1,RALA,RAN,RANGAP1,RAP1B,RAP2C,RARS,RBBP7,RBPJ,RCC2,RHBDF2,RIPK3,RNF149,RNF213,RNH1,RNMT,RNPEP,RPF2,RPL10,RPL14,RPL17,RPL18,RPL21,RPL26,RPL27A,RPL28,RPL3,RPL4,RPL6,RPL7,RPL7A,RPL8,RPN1,RPS14,RPS27A,RPS6,RPSA,RRP9,RTCB,SARS,SCAMP2,SCFD1,SEC24B,SF3B1,SFPQ,SHMT1,SHMT2,SIPA1,SIRPA,SLC12A4,SLC15A3,SLC16A3,SLC16A6,SLC1A5,SLC20A1,SLC23A2,SLC29A1,SLC2A1,SLC38A2,SLC3A2,SLC4A7,SLC7A1,SLCO4A1,SLFN13,SMPDL3B,SMU1,SNAP23,SND1,SNRNP200,SNX2,SNX27,SPRED1,SQSTM1,SRM,STAM2,STAT1,STEAP3,STOM,STRAP,STUB1,STX6,SUPT5H,TALDO1,TAX1BP1,TCIRG1,TCP1,TFRC,TGFBR2,TKT,TLR7,TMEM59,TNFRSF1B,TNIP1,TOM1,TPI1,TPP2,TRIM14,TRIM25,TRIM28,TRPV2,TSG101,TSR1,TTC37,TTLL12,UAP1L1,UBA1,UBA2,UCHL5,USP5,USP8,VAMP8,VASP,VIM,VPS13C,VWA5A,WARS,WDR82,WWP2,XPNPEP1,XPO1,XPO7,YBX1,ZC3HAV1	361
**Cancer,Organismal Injury and Abnormalities**	Adenocarcinoma	2.2E-12		-0.457	ABCA3,ABCE1,ABCG1,ACAA1,ACLY,ACSL4,ACTA1,ACTR1A,ACTR2,ACTR3,ADAM8,ADSL,ADSSL1,AHCY,AHNAK,AKR1B1,AKR1B10,ALCAM,ALDH9A1,AP3B1,APEX1,ARAP1,ARPC2,ATIC,ATP1A1,ATP1B3,ATP2B1,ATP2C1,ATP6V0A1,ATP7A,C5AR1,CA2,CAND1,CAPZA1,CCT2,CCT3,CCT4,CCT5,CCT6A,CCT7,CCT8,CD36,CD47,CDK1,CFL1,CLUH,CMAS,CMIP,CNDP2,CNOT1,COLEC12,COPB1,CORO1B,CORO1C,CORO7/CORO7-PAM16,CPD,CPNE8,CRYZ,CSE1L,CSNK1G3,CTPS1,CTSD,CTSK,DAAM1,DCTN1,DDX17,DDX21,DDX39B,DDX3X,DENND4B,DHX15,DHX29,DIS3,DKC1,DNAJA1,DNAJC13,DNM1L,DNM2,DNMT1,DOCK2,DPYSL2,ECPAS,EDIL3,EEF1D,EEF1G,EHD1,EIF2S1,EIF2S3,EIF3A,EIF3B,EIF3C,EIF3D,EIF3E,EIF3F,EIF3I,EIF3L,EIF3M,ENO1,ESYT1,FARSA,FARSB,FASN,FERMT3,FKBP4,FLNA,FLOT1,FLOT2,FYN,G3BP1,G6PD,GALK1,GARS,GART,GFPT1,GMDS,GMPS,GNA13,GNAI2,GNAS,GNB2,GPNMB,GRB2,GUSB,HIST1H1C,HK3,HLA-A,HNRNPL,HNRNPM,HNRNPU,HSD17B4,HSP90AA1,HSP90AB1,HSPA4,ICAM1,IDE,IDH1,IFIT1B,IL6ST,IPO5,IPO7,ITCH,ITGAM,ITGB2,JAK1,KCNN4,KIDINS220,KPNA2,KPNB1,KRT1,KRT10,KRT2,KRT9,LGALS3BP,LIG1,LILRB4,LRP12,LYN,MAPK3,MARS,MAT2B,MCM2,MCM7,MDH1,MEMO1,MFGE8,MOV10,MRI1,MSN,MTA2,MVP,MYADM,MYH9,MYO1C,MYO1E,MYO1G,MYOF,NAA15,NANS,NCBP1,NCF2,NCL,NCSTN,NOP56,NOS2,NOTCH2,NPC1,NRAS,NUP93,OLA1,PCNA,PDS5A,PDXK,PFAS,PGK1,PHGDH,PI4K2A,PKM,PLA2G4A,PLAU,PLD3,PLEC,PLEK,PLXNA1,PLXNB2,PNKP,POLD1,POLR1C,POLR2A,POLR2B,PPP1CA,PPP1R7,PPP2R1A,PPP2R2A,PREP,PRIM2,PRPF19,PTGS2,PTPN23,PTPRA,PTPRC,PTPRJ,RAB5A,RAB5B,RAB8B,RACK1,RALA,RAN,RANGAP1,RAP1B,RAP2C,RARS,RBBP7,RBPJ,RCC2,RHBDF2,RIPK3,RNF149,RNF213,RNH1,RNMT,RNPEP,RPF2,RPL10,RPL14,RPL18,RPL18A,RPL28,RPL3,RPL4,RPL8,RPN1,RPS14,RPS27A,RPS6,RRP9,RTCB,SARS,SCAMP2,SCFD1,SEC24B,SF3B1,SFPQ,SHMT1,SHMT2,SIPA1,SIRPA,SLC12A4,SLC15A3,SLC16A3,SLC16A6,SLC1A5,SLC20A1,SLC23A2,SLC29A1,SLC2A1,SLC38A2,SLC3A2,SLC4A7,SLC7A1,SLCO4A1,SLFN13,SMPDL3B,SMU1,SNAP23,SND1,SNRNP200,SNX2,SNX27,SPRED1,SQSTM1,SRM,STAT1,STEAP3,STOM,STRAP,STUB1,STX6,SUPT5H,TALDO1,TAX1BP1,TCIRG1,TCP1,TFRC,TGFBR2,TKT,TLR7,TMEM59,TNIP1,TOM1,TPI1,TPP2,TRIM14,TRIM25,TRIM28,TRPV2,TSG101,TSR1,TTC37,TTLL12,UAP1L1,UBA1,UBA2,UCHL5,USP5,USP8,VAMP8,VASP,VIM,VPS13C,VWA5A,WARS,WDR82,WWP2,XPNPEP1,XPO1,XPO7,YBX1,ZC3HAV1	338

Table S4

**Cancer,Gastrointestinal Disease,Organismal Injury and Abnormalities**	Digestive system cancer	3.4E-12		-0.848	ABCA3,ABCE1,ABCG1,ACAA1,ACLY,ACO2,ACSL4,ACTA1,ACTR1A,ACTR2,ACTR3,ADSL,ADSSL1,AHCY,AHNAK,AKR1B1,AKR1B10,ALCAM,ALDH9A1,AMDHD2,AP3B1,APEX1,ARAP1,ATIC,ATP1A1,ATP1B3,ATP2B1,ATP2C1,ATP6V0A1,ATP7A,C5AR1,CA2,CAND1,CCT2,CCT4,CCT5,CCT6A,CCT7,CCT8,CD36,CD47,CDK1,CFL1,CLUH,CMAS,CMIP,CNDP2,CNOT1,COLEC12,COPB1,COPG1,CORO1B,CORO1C,CORO7/CORO7-PAM16,CPD,CPNE8,CRYZ,CSE1L,CSNK1G3,CTPS1,CTSD,CTSK,DAAM1,DCTN1,DDX17,DDX21,DDX39B,DDX3X,DENND4B,DHX15,DHX29,DIS3,DKC1,DNAJA1,DNAJC13,DNM1L,DNM2,DNMT1,DOCK2,DPYSL2,ECPAS,EDIL3,EEF1D,EEF1G,EHD1,EIF2S3,EIF3A,EIF3B,EIF3C,EIF3D,EIF3E,EIF3F,EIF3I,EIF3L,EIF3M,ENO1,ESYT1,FARSA,FARSB,FASN,FERMT3,FKBP4,FLNA,FLOT1,FLOT2,FYN,G3BP1,G6PD,GALK1,GARS,GART,GFPT1,GMDS,GMPS,GNA13,GNAI2,GNAS,GNB2,GPNMB,GRB2,GUSB,HIST1H1C,HK3,HLA-A,HNRNPL,HNRNPM,HNRNPU,HSD17B4,HSP90AA1,HSP90AB1,HSPA4,ICAM1,IDE,IDH1,IFIT1B,IFITM3,IL6ST,IPO5,IPO7,ITCH,ITGAM,ITGB2,JAK1,KCNN4,KIDINS220,KPNA2,KPNB1,KRT1,KRT10,KRT2,KRT9,LGALS3BP,LIG1,LILRB4,LRP12,LYN,MAPK3,MARS,MAT2A,MAT2B,MCM2,MCM7,MDH1,MFGE8,MOV10,MRI1,MSN,MSTO1,MTA2,MTHFD1,MVP,MYADM,MYH9,MYO1C,MYO1E,MYO1G,MYOF,NAA15,NANS,NCBP1,NCF2,NCL,NCSTN,NDRG1,NOP56,NOS2,NOTCH2,NPC1,NRAS,NUP93,OASL,OLA1,PCNA,PDS5A,PFAS,PFN1,PGK1,PHGDH,PI4K2A,PKM,PLA2G4A,PLAU,PLD3,PLEC,PLXNA1,PLXNB2,PNKP,POLD1,POLR1C,POLR2B,PPP1CA,PPP2CA,PPP2R1A,PPP2R2A,PREP,PRIM2,PRPF4,PTGS2,PTPN23,PTPRA,PTPRC,PTPRJ,RAB31,RAB5A,RAB5B,RAB8B,RACK1,RALA,RAN,RANGAP1,RAP1B,RAP2C,RARS,RBBP7,RBPJ,RCC2,RHBDF2,RIPK3,RNF213,RNH1,RNMT,RNPEP,RPL10,RPL18,RPL18A,RPL27A,RPL28,RPL3,RPL4,RPL6,RPL7A,RPL8,RPN1,RPS14,RPS27A,RPS6,RPSA,RRP9,RTCB,SARS,SCAMP2,SCFD1,SEC24B,SF3B1,SFPQ,SHMT1,SHMT2,SIPA1,SIRPA,SLC12A4,SLC15A3,SLC16A3,SLC16A6,SLC1A5,SLC20A1,SLC23A2,SLC29A1,SLC2A1,SLC38A2,SLC3A2,SLC4A7,SLC7A1,SLCO4A1,SLFN13,SMPDL3B,SMU1,SNAP23,SND1,SNRNP200,SNX2,SNX27,SPRED1,SQSTM1,SRM,STAM2,STAT1,STEAP3,STOM,STRAP,STUB1,STX6,SUPT5H,TALDO1,TAX1BP1,TCIRG1,TCP1,TFRC,TGFBR2,TKT,TLR7,TMEM59,TNFRSF1B,TNIP1,TOM1,TPI1,TPP2,TRIM14,TRIM25,TRIM28,TRPV2,TSG101,TTC37,TTLL12,UAP1L1,UBA1,UBA2,UCHL5,USP5,USP8,VASP,VIM,VPS13C,VWA5A,WARS,WDR82,WWP2,XPNPEP1,XPO1,XPO7,YBX1,ZC3HAV1	341
**Cancer,Organismal Injury and Abnormalities**	Abdominal adenocarcinoma	3.4E-12		0	ABCA3,ABCE1,ABCG1,ACAA1,ACLY,ACSL4,ACTA1,ACTR1A,ACTR2,ACTR3,ADAM8,ADSL,ADSSL1,AHCY,AHNAK,AKR1B1,AKR1B10,ALCAM,ALDH9A1,AP3B1,APEX1,ARAP1,ARPC2,ATIC,ATP1A1,ATP1B3,ATP2B1,ATP2C1,ATP6V0A1,ATP7A,C5AR1,CA2,CAND1,CAPZA1,CCT2,CCT3,CCT4,CCT5,CCT6A,CCT7,CCT8,CD36,CD47,CDK1,CFL1,CLUH,CMAS,CMIP,CNDP2,CNOT1,COLEC12,COPB1,CORO1B,CORO1C,CORO7/CORO7-PAM16,CPD,CPNE8,CSE1L,CSNK1G3,CTPS1,CTSK,DAAM1,DCTN1,DDX17,DDX21,DDX39B,DDX3X,DENND4B,DHX15,DHX29,DIS3,DKC1,DNAJA1,DNAJC13,DNM1L,DNM2,DNMT1,DOCK2,DPYSL2,ECPAS,EDIL3,EEF1D,EEF1G,EHD1,EIF2S1,EIF2S3,EIF3A,EIF3B,EIF3C,EIF3D,EIF3E,EIF3F,EIF3I,EIF3L,EIF3M,ESYT1,FARSA,FARSB,FASN,FERMT3,FKBP4,FLNA,FLOT1,FLOT2,FYN,G3BP1,G6PD,GALK1,GARS,GART,GFPT1,GMDS,GMPS,GNA13,GNAI2,GNAS,GNB2,GPNMB,GRB2,GUSB,HIST1H1C,HK3,HLA-A,HNRNPL,HNRNPM,HNRNPU,HSD17B4,HSP90AA1,HSP90AB1,HSPA4,ICAM1,IDE,IDH1,IFIT1B,IL6ST,IPO5,IPO7,ITCH,ITGAM,ITGB2,JAK1,KCNN4,KIDINS220,KPNA2,KPNB1,KRT1,KRT10,KRT2,KRT9,LGALS3BP,LIG1,LILRB4,LRP12,LYN,MAPK3,MARS,MAT2B,MCM2,MCM7,MDH1,MEMO1,MFGE8,MOV10,MRI1,MSN,MTA2,MVP,MYADM,MYH9,MYO1C,MYO1E,MYO1G,MYOF,NAA15,NANS,NCBP1,NCF2,NCL,NCSTN,NOP56,NOS2,NOTCH2,NPC1,NRAS,NUP93,OLA1,PCNA,PDS5A,PDXK,PFAS,PGK1,PHGDH,PI4K2A,PKM,PLA2G4A,PLAU,PLD3,PLEC,PLEK,PLXNA1,PLXNB2,PNKP,POLD1,POLR1C,POLR2B,PPP1CA,PPP1R7,PPP2R1A,PPP2R2A,PREP,PRIM2,PTGS2,PTPN23,PTPRA,PTPRC,PTPRJ,RAB5A,RAB5B,RAB8B,RACK1,RALA,RAN,RANGAP1,RAP1B,RAP2C,RARS,RBBP7,RBPJ,RCC2,RHBDF2,RIPK3,RNF149,RNF213,RNH1,RNMT,RNPEP,RPF2,RPL10,RPL14,RPL18,RPL28,RPL3,RPL4,RPL8,RPN1,RPS14,RPS27A,RPS6,RRP9,RTCB,SARS,SCAMP2,SCFD1,SEC24B,SF3B1,SFPQ,SHMT1,SHMT2,SIPA1,SIRPA,SLC12A4,SLC15A3,SLC16A3,SLC16A6,SLC1A5,SLC20A1,SLC23A2,SLC29A1,SLC2A1,SLC38A2,SLC3A2,SLC4A7,SLC7A1,SLCO4A1,SLFN13,SMPDL3B,SMU1,SNAP23,SND1,SNRNP200,SNX2,SNX27,SPRED1,SQSTM1,SRM,STAT1,STEAP3,STOM,STRAP,STUB1,STX6,SUPT5H,TALDO1,TAX1BP1,TCIRG1,TCP1,TFRC,TGFBR2,TKT,TLR7,TMEM59,TNIP1,TOM1,TPI1,TPP2,TRIM14,TRIM25,TRIM28,TRPV2,TSG101,TSR1,TTC37,TTLL12,UAP1L1,UBA1,UBA2,UCHL5,USP5,USP8,VAMP8,VASP,VIM,VPS13C,VWA5A,WARS,WDR82,WWP2,XPNPEP1,XPO1,XPO7,YBX1,ZC3HAV1	333
**Infectious Diseases**	Viral Infection	3.6E-12		-1.814	ACTR3,ATP5F1B,CD36,CD47,DCTN1,DDX17,DDX3X,DNAJA1,DNM1L,DNM2,FASN,FLNA,FLOT2,GRB2,HIST1H1C,HLA-A,HNRNPM,HSP90AB1,HSPA4,ICAM1,IFITM3,ITCH,ITGAM,ITGB2,JAK1,KCNN4,KPNB1,MOV10,MVP,NCL,NOS2,NPC1,OASL,PDXK,PLAU,PNKP,POLD1,PTGS2,PTPRC,RAB31,RAN,RBPJ,RPS27A,SDCBP,SLC2A1,STAT1,TALDO1,TCIRG1,TFRC,TGFBR2,TKT,TLR7,TNFRSF1B,TSG101,VAMP8,WWP2,XPO1,ZC3HAV1	58

Table S4

**Cancer,Cell Death and Survival,Organismal Injury and Abnormalities,Tumor Morphology**	Necrosis of tumor	4.3E-12		0.387	ACLY,CD47,CDK1,COPG1,CSE1L,EIF3B,EIF3C,EIF3E,EIF3F,EIF3L,ENO1,FASN,HSP90AB1,Ifi202b,IL6ST,KPNB1,MCM2,MFGE8,NDRG1,NRAS,PLAU,PPP2CA,PTGS2,PTPRA,RACK1,RAN,RBPJ,RPL10,RPL13,RPL27A,RPL3,RPL6,RPL7,RPL7A,RPS14,RPS27A,SF3B1,TGFBR2,TLR7,TNFRSF1B,TSG101	41
**Cancer,Organismal Injury and Abnormalities**	Abdominal cancer	5.1E-12		-0.26	ABCA3,ABCE1,ABCG1,ACAA1,ACLY,ACO2,ACSL4,ACTA1,ACTR1A,ACTR2,ACTR3,ADAM8,ADSL,ADSSL1,AHCY,AHNAK,AKR1B1,AKR1B10,ALCAM,ALDH9A1,AP3B1,APEX1,ARAP1,ARPC2,ATIC,ATP1A1,ATP1B3,ATP2B1,ATP2C1,ATP6V0A1,ATP7A,C5AR1,CA2,CAND1,CAPZA1,CCT2,CCT3,CCT4,CCT5,CCT6A,CCT7,CCT8,CD36,CD47,CDK1,CFL1,CLUH,CMAS,CMIP,CNDP2,CNOT1,COLEC12,COPB1,COPG1,CORO1B,CORO1C,CORO7/CORO7-PAM16,CPD,CPNE8,CRYZ,CSE1L,CSNK1G3,CTPS1,CTSD,CTSK,DAAM1,DCTN1,DDX17,DDX21,DDX39B,DDX3X,DENND4B,DHX15,DHX29,DIS3,DKC1,DNAJA1,DNAJC13,DNM1L,DNM2,DNMT1,DOCK2,DPYSL2,ECPAS,EDIL3,EEF1D,EEF1G,EHD1,EIF2S1,EIF2S3,EIF3A,EIF3B,EIF3C,EIF3D,EIF3E,EIF3F,EIF3I,EIF3L,EIF3M,ENO1,ESYT1,FARSA,FARSB,FASN,FERMT3,FKBP4,FLNA,FLOT1,FLOT2,FYN,G3BP1,G6PD,GALK1,GARS,GART,GFPT1,GMDS,GMPS,GNA13,GNAI2,GNAS,GNB2,GPNMB,GRB2,GUSB,HIST1H1C,HIST1H2AJ,HK3,HLA-A,HNRNPL,HNRNPM,HNRNPU,HSD17B4,HSP90AA1,HSP90AB1,HSPA4,ICAM1,IDE,IDH1,IFIT1B,IFITM3,IL6ST,IPO5,IPO7,ITCH,ITGAM,ITGB2,JAK1,KCNN4,KIDINS220,KPNA2,KPNB1,KRT1,KRT10,KRT2,KRT9,LGALS3BP,LIG1,LILRB4,LRP12,LYN,MAPK3,MARS,MAT2A,MAT2B,MCM2,MCM7,MDH1,MEMO1,MFGE8,MOV10,MRI1,MSN,MSTO1,MTA2,MTHFD1,MVP,MYADM,MYH9,MYO1C,MYO1E,MYO1G,MYOF,NAA15,NANS,NCBP1,NCF2,NCL,NCSTN,NDRG1,NOP56,NOS2,NOTCH2,NPC1,NRAS,NUP93,OASL,OLA1,PCNA,PDS5A,PDXK,PFAS,PFN1,PGK1,PHGDH,PI4K2A,PKM,PLA2G4A,PLAU,PLD3,PLEC,PLEK,PLXNA1,PLXNB2,PNKP,POLD1,POLR1C,POLR2B,PPAT,PPP1CA,PPP1R7,PPP2CA,PPP2R1A,PPP2R2A,PREP,PRIM2,PRPF4,PTGS2,PTPN23,PTPRA,PTPRC,PTPRJ,RAB31,RAB5A,RAB5B,RAB8B,RACK1,RALA,RAN,RANGAP1,RAP1B,RAP2C,RARS,RBBP7,RBPJ,RCC2,RHBDF2,RIPK3,RNF149,RNF213,RNH1,RNMT,RNPEP,RPF2,RPL10,RPL14,RPL17,RPL18,RPL27A,RPL28,RPL3,RPL4,RPL6,RPL7A,RPL8,RPN1,RPS14,RPS27A,RPS6,RPSA,RRP9,RTCB,SARS,SCAMP2,SCFD1,SEC24B,SF3B1,SFPQ,SHMT1,SHMT2,SIPA1,SIRPA,SLC12A4,SLC15A3,SLC16A3,SLC16A6,SLC1A5,SLC20A1,SLC23A2,SLC29A1,SLC2A1,SLC38A2,SLC3A2,SLC4A7,SLC7A1,SLCO4A1,SLFN13,SMPDL3B,SMU1,SNAP23,SND1,SNRNP200,SNX2,SNX27,SPRED1,SQSTM1,SRM,STAM2,STAT1,STEAP3,STOM,STRAP,STUB1,STX6,SUPT5H,TALDO1,TAX1BP1,TCIRG1,TCP1,TFRC,TGFBR2,TKT,TLR7,TMEM59,TNFRSF1B,TNIP1,TOM1,TPI1,TPP2,TRIM14,TRIM25,TRIM28,TRPV2,TSG101,TSR1,TTC37,TTLL12,UAP1L1,UBA1,UBA2,UCHL5,USP5,USP8,VAMP8,VASP,VIM,VPS13C,VWA5A,WARS,WDR82,WWP2,XPNPEP1,XPO1,XPO7,YBX1,ZC3HAV1	356
**Cell Death and Survival**	Apoptosis	6E-12		0.718	ABCG1,ACLY,ACSL4,ADAM8,AKR1B1,ALCAM,APEX1,ATP1A1,ATP1B3,ATP2C1,ATP7A,C5AR1,CCT2,CD36,CD47,CDK1,CFL1,CMIP,CSE1L,CTSD,DCTN1,DDX3X,DNAJA1,DNM1L,DNM2,DNMT1,EDIL3,EEF1D,EHD1,EIF2A,EIF2S1,EIF3F,ENO1,FASN,FCER1G,FKBP4,FLNA,FLOT2,FYN,G6PD,GFPT1,GNA13,GNAI2,GNAS,HIST1H1C,HSP90AA1,HSP90AB1,HSPA4,ICAM1,Ifi202b,IL6ST,Irgm1,ITCH,ITGAM,ITGB2,KIDINS220,KPNB1,LIG1,LYN,MAPK3,MCM2,MDH1,MFGE8,MTA2,MVP,NAA15,NCF2,NCL,NCSTN,NDRG1,NOS2,NOTCH2,NPC1,NRAS,PCNA,PDXK,PFN1,PKM,PLA2G4A,PLAU,POLR2A,PPP1CA,PPP2CA,PPP2R1A,PRPF19,PTGS2,PTPRC,RACK1,RAP1B,RBPJ,RIPK3,RPL10,RPS27A,RPS6,SF3B1,SLC20A1,SLC2A1,SLC4A7,SQSTM1,STAT1,STEAP3,STUB1,TAX1BP1,TFRC,TGFBR2,TLR7,TNFRSF1B,TNIP1,TPP2,TRIM28,UBA1,UCHL5,VASP,VIM,WWP2,XPO1,XPO7,YBX1	118
**Cellular Function and Maintenance**	Engulfment of cells	8.1E-12		1.993	CD36,CD47,COLEC12,CORO1C,DNM2,DOCK2,EHD1,FCER1G,FLNA,FLOT1,FYN,GRB2,HSP90AA1,ICAM1,ITGAM,ITGB2,LYN,MFGE8,MYH9,MYO1G,PFN1,PLA2G4A,PLAU,PTPRC,PTPRJ,RAB31,RAB5B,RAB5C,RAB7A,RACK1,RALA,SFPQ,SIRPA,SNAP23,SNX5,STAM2,TRPV2,VAMP8,VIM	39
**Cellular Movement**	Cell movement of blood cells	8.5E-12	Increased	2.635	Abcb1b,ABCG1,ADAM8,AKR1B1,ALCAM,ATP1B3,C5AR1,CD36,CD47,DOCK2,DPYSL2,EDIL3,FCER1G,FERMT3,FLNA,FLOT1,FYN,GBA,GNA13,GNAI2,GNAI3,GNAS,GRB2,HLA-A,HNRNPL,ICAM1,IL6ST,Irgm1,ITGAM,ITGB2,JAK1,KCNN4,KRT10,LYN,MAPK3,MSN,MYADM,MYH9,MYO1G,NDRG1,NOS2,NPC1,NRAS,PFN1,PLA2G4A,PLAU,PLEC,PTGS2,PTPRA,PTPRC,PTPRJ,RACK1,RAP1B,SIRPA,SLC16A3,SLC3A2,STAT1,TAX1BP1,TCIRG1,TGFBR2,TLR7,TNFRSF1B,TNIP1,TRPV2,VASP,YBX1	66

Table S4

**Cancer,Cell Death and Survival,Organismal Injury and Abnormalities,Tumor Morphology**	Cell death of tumor cells	8.6E-12		0.387	ACLY,CD47,CDK1,COPG1,CSE1L,EIF3B,EIF3C,EIF3E,EIF3F,EIF3L,ENO1,FASN,HSP90AB1,Ifi202b,IL6ST,KPNB1,MCM2,MFGE8,NDRG1,NRAS,PLAU,PPP2CA,PTGS2,PTPRA,RACK1,RAN,RBPJ,RPL10,RPL13,RPL27A,RPL3,RPL6,RPL7,RPL7A,RPS14,RPS27A,SF3B1,TGFBR2,TLR7,TSG101	40
**Connective Tissue Disorders,Inflammatory Disease,Organismal Injury and Abnormalities,Skeletal and Muscular Disorders**	Rheumatic Disease	9E-12		0.256	ACLY,ACTA1,ACTL6A,ACTR3,ADAM8,AP3B1,ATIC,ATP2B1,ATP2C1,C5AR1,CA2,CD36,DCTN1,DDX39B,DNM1L,DNMT1,EEF1G,EIF3E,ENO1,FASN,FCER1G,GNAI2,GNAS,GNB2,GUSB,HELZ2,HLA-A,ICAM1,IDE,IL6ST,ITGAM,ITGB2,JAK1,KRT10,LYN,MAPK3,MTA2,MYH9,MYO1C,NCF2,NOS2,OASL,PFAS,PGK1,PLA2G4A,PLAU,POLD1,PPAT,PPP1CA,PPP1R7,PPP2CA,PREP,PRIM2,PTGS2,PTPRC,RAB31,RAB5A,RALA,RBPJ,RIPK3,RNF149,RPL18A,RPSA,SEC24B,SIPA1,SLC7A1,SND1,SNRNP200,STAT1,TALDO1,TCIRG1,TFRC,TGFBR2,TLR7,TNFRSF1B,TRIM25,TRIM28,VIM	78
**Cell-To-Cell Signaling and Interaction, Cellular Function and Maintenance,Inflammatory Response**	Phagocytosis of cells	1E-11	Increased	2.118	CD36,CD47,COLEC12,CORO1C,DNM2,DOCK2,EHD1,FCER1G,FLNA,FYN,GRB2,ICAM1,ITGAM,ITGB2,LYN,MFGE8,MYH9,MYO1G,PFN1,PLA2G4A,PLAU,PTPRC,PTPRJ,RAB31,RACK1,RALA,SIRPA,SNAP23,TRPV2,VIM	30
**Cancer,Organismal Injury and Abnormalities**	Genitourinary tumor	1.3E-11		1.46	ABCA3,ABCE1,ACAA1,ACLY,ACSL4,ACTR1A,ACTR2,ACTR3,ADAM8,ADSL,ADSSL1,AHNAK,AKR1B1,ALCAM,ALDH9A1,AP3B1,APEX1,ARPC2,ATIC,ATP1A1,ATP2B1,ATP2C1,ATP6V0A1,ATP7A,CA2,CAND1,CAPZA1,CCT2,CCT3,CCT5,CCT6A,CD36,CD47,CDK1,CLUH,CMAS,CMIP,CNDP2,CNOT1,COLEC12,COPB1,CORO1C,CPD,CPNE8,CSE1L,CTPS1,CTSD,CTSK,DCTN1,DDX17,DDX21,DDX39B,DDX3X,DENND4B,DHX29,DIS3,DKC1,DNAJA1,DNAJC13,DNM1L,DNMT1,DOCK2,ECPAS,EDIL3,EEF1D,EEF1G,EIF2A,EIF2S1,EIF2S3,EIF3A,EIF3B,EIF3C,EIF3D,EIF3E,EIF3F,EIF3I,ENO1,ESYT1,FASN,FERMT3,FKBP4,FLNA,FLOT1,FYN,G3BP1,G6PD,GART,GBA,GFPT1,GMDS,GNA13,GNAI2,GNAI3,GNAS,GNB2,GPNMB,GRB2,GUSB,HIST1H1C,HIST1H2AJ,HK3,HLA-A,HNRNPL,HNRNPM,HNRNPU,HSD17B4,HSP90AA1,HSP90AB1,HSPA4,ICAM1,IDH1,Ifi202b,IPO5,ITCH,ITGAM,ITGB2,JAK1,KIDINS220,KPNA2,KRT1,KRT10,KRT2,KRT9,LGALS3BP,LIG1,LILRB4,LRP12,LYN,MAPK3,MAT2B,MCM2,MCM7,MDH1,MEMO1,MFGE8,MOV10,MSN,MSTO1,MVP,MYADM,MYH9,MYO1C,MYO1E,MYO1G,MYOF,NAA15,NANS,NCBP1,NCF2,NCL,NCSTN,NDRG1,NOP56,NOS2,NOTCH2,NPC1,NRAS,NUP93,NUS1,OASL,OLA1,PCNA,PDS5A,PDXK,PFAS,PFN1,PGK1,PI4K2A,PKM,PLAU,PLD3,PLEC,PLEK,PLXNA1,PLXNB2,PNKP,POLD1,POLR1C,POLR2A,POLR2B,PPAT,PPP1R7,PPP2CA,PPP2R1A,PREP,PRIM2,PTGS2,PTPN23,PTPRA,PTPRC,PTPRJ,RAB31,RAB5B,RAB5C,RALA,RANGAP1,RAP1B,RAP2C,RARS,RBBP7,RCC2,RIPK3,RNF149,RNF213,RNPEP,RPF2,RPL14,RPL17,RPL4,RPL6,RPS27A,RPS6,RRP9,RTCB,SCFD1,SDCBP,SEC24B,SF3B1,SFPQ,SHMT2,SIRPA,SLC12A4,SLC15A3,SLC16A3,SLC20A1,SLC23A2,SLC29A1,SLC2A1,SLC38A2,SLC3A2,SLC4A7,SLC7A1,SLFN13,SMPDL3B,SND1,SNRNP200,SNX27,STAT1,STEAP3,STOM,STUB1,STX6,SUPT5H,TALDO1,TCP1,TFRC,TGFBR2,TKT,TLR7,TMEM59,TNFRSF1B,TNIP1,TOM1,TPI1,TPP2,TRIM25,TRIM28,TRPV2,TSG101,TSR1,TTC37,TTLL12,UBA1,UBA2,UCHL5,USP5,USP8,VAMP8,VIM,VPS13C,VWA5A,WWP2,XPO1,XPO7,YBX1	275
**Cell Death and Survival**	Cell survival	1.5E-11		0.472	Abcb1b,ACLY,ACTL6A,ALCAM,APEX1,ATP7A,CD47,CDK1,CSE1L,CTSD,DDX3X,DNM1L,DNMT1,EIF2A,EIF3A,EIF3C,ENO1,FASN,FCER1G,FYN,GPNMB,GRB2,HLA-A,HSP90AB1,HSPA4,ICAM1,IDE,IDH1,IL6ST,ITGB2,JAK1,LIG1,LYN,MAPK3,MCM2,MCM7,MVP,NCF2,NDRG1,NOS2,NOTCH2,NPC1,NRAS,PCNA,PKM,PLA2G4A,PLAU,PNKP,PPP1CA,PPP2R2A,PTGS2,PTPRA,PTPRC,RAB5A,RBPJ,RIPK3,SF3B1,SIPA1,SND1,STAM2,STAT1,TNFRSF1B,TRIM28,TSG101,VIM,XPO1	66

Table S4

**Cellular Movement,Immune Cell Trafficking**	Leukocyte migration	1.6E-11	Increased	2.783	Abcb1b,ABCG1,ADAM8,ALCAM,ATP1B3,C5AR1,CD36,CD47,DOCK2,DPYSL2,EDIL3,FCER1G,FERMT3,FLNA,FLOT1,FYN,GBA,GNA13,GNAI2,GNAI3,GNAS,GRB2,HLA-A,HNRNPL,ICAM1,IL6ST,Irgm1,ITGAM,ITGB2,JAK1,KCNN4,KRT10,LYN,MAPK3,MSN,MYADM,MYH9,MYO1G,NDRG1,NOS2,NPC1,NRAS,PFN1,PLA2G4A,PLAU,PLEC,PTGS2,PTPRA,PTPRC,PTPRJ,RACK1,RAP1B,SIRPA,SLC16A3,SLC3A2,STAT1,TAX1BP1,TCIRG1,TGFBR2,TLR7,TNFRSF1B,TNIP1,TRPV2,VASP,YBX1	65
**Cancer,Organismal Injury and Abnormalities**	Lymphatic system tumor	1.9E-11	Decreased	-2.183	ABCG1,ADSL,AHNAK,ALDH9A1,ATIC,CA2,CCT3,CCT7,CD36,CD47,CDK1,CFL1,CMIP,CNOT1,CPD,CSE1L,CTSK,DDX3X,DHX15,DIS3,DNM1L,DNM2,DNMT1,DOCK2,EHD1,EIF2A,FASN,FLNA,FYN,G3BP1,GART,GNA13,GNAI2,GRB2,HIST1H1C,HLA-A,HNRNPM,HNRNPU,HSP90AA1,HSP90AB1,ICAM1,IDH1,IL6ST,IPO5,IPO7,ITGAM,ITGB2,JAK1,KPNA2,KPNB1,KRT1,KRT10,KRT2,LYN,MAPK3,MARS,MPEG1,MYO1G,MYOF,NOS2,NOTCH2,NRAS,OASL,PCNA,PLAU,PLXNB2,POLD1,POLR2B,PPAT,PPP2CA,PREP,PRIM2,PRPF19,PTGS2,PTPRC,RALA,RAN,RNF213,RPL10,RPL13,RPS6,RPSA,SARS,SF3B1,SHMT1,SHMT2,SLC2A1,SPRED1,STAT1,STEAP3,TAX1BP1,TFRC,TGFBR2,TLR7,TNIP1,TRIM25,TSR1,UBA2,USP8,VIM,XPO1	101
**Cellular Movement,Hematological System Development and Function,Immune Cell Trafficking**	Cell movement of leukocytes	2E-11	Increased	2.697	Abcb1b,ABCG1,ADAM8,ALCAM,C5AR1,CD36,CD47,DOCK2,DPYSL2,EDIL3,FCER1G,FERMT3,FLNA,FLOT1,FYN,GBA,GNA13,GNAI2,GNAI3,GNAS,HLA-A,HNRNPL,ICAM1,IL6ST,Irgm1,ITGAM,ITGB2,JAK1,KCNN4,KRT10,LYN,MAPK3,MSN,MYADM,MYH9,MYO1G,NDRG1,NOS2,NPC1,NRAS,PFN1,PLA2G4A,PLAU,PLEC,PTGS2,PTPRA,PTPRJ,RAP1B,SIRPA,STAT1,TCIRG1,TGFBR2,TNFRSF1B,TNIP1,TRPV2,VASP,YBX1	57
**Cellular Function and Maintenance**	Endocytosis by eukaryotic cells	2E-11		1.416	CD36,CD47,DNM2,DOCK2,EHD1,FCER1G,FLNA,FYN,GRB2,HSP90AA1,ICAM1,ITGAM,ITGB2,LYN,MFGE8,MYH9,MYO1G,PFN1,PLA2G4A,PLAU,PTPRC,PTPRJ,RAB31,RACK1,RALA,SFPQ,SIRPA,SNAP23,STAM2,TRPV2,VIM	31
**Protein Degradation,Protein Synthesis**	Catabolism of protein	2.7E-11		-0.146	ATP1B3,CCT2,CCT3,CCT4,CCT5,CCT6A,CCT7,CCT8,CFL1,CNDP2,CTSD,CTSK,ECPAS,FLNA,FLOT1,FLOT2,FYN,GBA,GOLGA7,HSP90AA1,HSP90AB1,IDE,IRGM,ITCH,JAK1,MYH9,NAA15,NCSTN,NOS2,PFN1,PLAU,PPP2CA,PREP,PTPRC,RAB7A,RNF149,RNF213,RNPEP,SQSTM1,STUB1,TCIRG1,TCP1,TPP2,TRIM25,TSG101,XPNPEP1,XPO1	47
**Molecular Transport**	Transport of molecule	2.9E-11		1.733	Abcb1b,ABCG1,ACLY,ACSL4,ACTR1A,AP3B1,ATP1A1,ATP1B3,ATP2B1,ATP2C1,ATP5F1B,ATP6V0A1,ATP7A,CA2,CAND1,CD36,CFL1,CORO7/CORO7-PAM16,CPSF1,DCTN1,DDX39B,DDX3X,DNAJA1,DNM1L,DNM2,DPYSL2,EHD1,FCER1G,FKBP4,FLNA,FLOT2,FYN,GNA13,GNAI2,GNAI3,GNAS,HLA-A,IL6ST,IPO5,IPO7,KCNN4,KPNA2,KPNB1,LYN,MSN,MVP,MYH9,MYO1C,NCBP1,NOS2,NPC1,NUP93,PLA2G4A,PTGS2,PTPRC,RAB7A,RAN,RANGAP1,RHBDF2,SCAMP2,SCFD1,SIRPA,SLC12A4,SLC15A3,SLC16A3,SLC1A5,SLC20A1,SLC23A2,SLC29A1,SLC2A1,SLC38A2,SLC3A2,SLC4A7,SLC7A1,SNAP23,SNX27,SQSTM1,STAT1,STEAP3,STX6,TCIRG1,TFRC,TNFRSF1B,TRIM28,TSG101,VAMP8,VPS13C,WWP2,XPO1,XPO7	90
**Post-Translational Modification,Protein Folding**	Folding of protein	4.7E-11			CCT2,CCT3,CCT4,CCT5,CCT6A,CCT7,CCT8,DNAJA1,FKBP4,GNAI2,GNAI3,GNB2,HSP90AA1,HSP90AB1,RAB7A,STUB1,TCP1	17
**Cancer,Cell Death and Survival,Organismal Injury and Abnormalities,Tumor Morphology**	Necrosis of malignant tumor	4.9E-11		0.448	ACLY,CD47,CDK1,COPG1,CSE1L,EIF3B,EIF3C,EIF3E,EIF3F,EIF3L,ENO1,FASN,HSP90AB1,Ifi202b,IL6ST,KPNB1,MCM2,NDRG1,NRAS,PTGS2,PTPRA,RAN,RBPJ,RPL10,RPL13,RPL27A,RPL3,RPL6,RPL7,RPL7A,RPS14,RPS27A,SF3B1,TLR7,TNFRSF1B	35

Table S4

**Cancer,Organismal Injury and Abnormalities**	Malignant genitourinary solid tumor	5.6E-11		0.579	ABCA3,ABCE1,ACAA1,ACLY,ACSL4,ACTR1A,ACTR2,ACTR3,ADAM8,ADSL,ADSSL1,AHNAK,AKR1B1,ALCAM,ALDH9A1,AP3B1,APEX1,ARPC2,ATIC,ATP1A1,ATP2B1,ATP2C1,ATP6V0A1,ATP7A,CA2,CAND1,CAPZA1,CCT2,CCT3,CCT5,CCT6A,CD36,CD47,CDK1,CLUH,CMAS,CMIP,CNDP2,CNOT1,COLEC12,COPB1,CORO1C,CPD,CPNE8,CSE1L,CTPS1,CTSD,CTSK,DCTN1,DDX17,DDX21,DDX39B,DDX3X,DENND4B,DHX29,DIS3,DKC1,DNAJA1,DNAJC13,DNM1L,DNMT1,DOCK2,ECPAS,EDIL3,EEF1D,EEF1G,EIF2S1,EIF2S3,EIF3A,EIF3B,EIF3C,EIF3D,EIF3E,EIF3F,EIF3I,ENO1,ESYT1,FASN,FERMT3,FKBP4,FLNA,FLOT1,FYN,G3BP1,G6PD,GART,GBA,GFPT1,GMDS,GNA13,GNAI2,GNAI3,GNAS,GNB2,GPNMB,GRB2,GUSB,HIST1H1C,HIST1H2AJ,HK3,HLA-A,HNRNPL,HNRNPM,HNRNPU,HSD17B4,HSP90AA1,HSP90AB1,HSPA4,ICAM1,IDH1,IPO5,ITCH,ITGAM,ITGB2,JAK1,KIDINS220,KPNA2,KRT1,KRT10,KRT2,KRT9,LGALS3BP,LIG1,LILRB4,LRP12,MAPK3,MAT2B,MCM2,MCM7,MDH1,MEMO1,MFGE8,MOV10,MSN,MSTO1,MVP,MYADM,MYH9,MYO1C,MYO1E,MYO1G,MYOF,NAA15,NANS,NCBP1,NCF2,NCL,NCSTN,NOP56,NOS2,NOTCH2,NPC1,NRAS,NUP93,NUS1,OASL,OLA1,PCNA,PDS5A,PDXK,PFAS,PFN1,PGK1,PI4K2A,PKM,PLAU,PLD3,PLEC,PLEK,PLXNA1,PLXNB2,PNKP,POLD1,POLR1C,POLR2A,POLR2B,PPAT,PPP1R7,PPP2CA,PPP2R1A,PREP,PRIM2,PTGS2,PTPN23,PTPRA,PTPRC,PTPRJ,RAB31,RAB5B,RAB5C,RALA,RANGAP1,RAP1B,RAP2C,RARS,RBBP7,RCC2,RIPK3,RNF149,RNF213,RNPEP,RPF2,RPL14,RPL17,RPL4,RPL6,RPS27A,RPS6,RRP9,RTCB,SCFD1,SDCBP,SEC24B,SF3B1,SFPQ,SHMT2,SIRPA,SLC12A4,SLC15A3,SLC16A3,SLC20A1,SLC23A2,SLC29A1,SLC2A1,SLC38A2,SLC3A2,SLC7A1,SLFN13,SMPDL3B,SND1,SNRNP200,SNX27,STAT1,STEAP3,STOM,STUB1,STX6,SUPT5H,TALDO1,TCP1,TFRC,TGFBR2,TKT,TLR7,TNFRSF1B,TNIP1,TOM1,TPI1,TPP2,TRIM25,TRIM28,TRPV2,TSG101,TSR1,TTC37,TTLL12,UBA1,UBA2,UCHL5,USP5,USP8,VAMP8,VIM,VPS13C,VWA5A,WWP2,XPO1,XPO7,YBX1	269
**Hematological Disease,Immunological Disease**	Lymphoproliferative disorder	5.6E-11	Decreased	-2.4	ABCG1,ADSL,AHNAK,ALDH9A1,ATIC,CA2,CCT3,CCT7,CD36,CD47,CDK1,CFL1,CMIP,CNOT1,CPD,CSE1L,CTSK,DDX3X,DIS3,DNM1L,DNM2,DNMT1,DOCK2,EIF2A,FASN,FLNA,FYN,G3BP1,GART,GNA13,GNAI2,GRB2,HIST1H1C,HLA-A,HNRNPM,HNRNPU,HSP90AA1,HSP90AB1,ICAM1,IDH1,IL6ST,IPO5,IPO7,ITGAM,ITGB2,JAK1,KPNA2,KPNB1,KRT1,KRT10,KRT2,LYN,MAPK3,MARS,MPEG1,MYO1G,MYOF,NOS2,NOTCH2,NRAS,OASL,PCNA,PLXNB2,POLD1,PPAT,PPP2CA,PREP,PRIM2,PRPF19,PTGS2,PTPRC,RALA,RAN,RIPK3,RNF213,RPL10,RPL13,RPS6,RPSA,SARS,SF3B1,SHMT1,SHMT2,SLC2A1,SPRED1,STAT1,STEAP3,TAX1BP1,TFRC,TGFBR2,TLR7,TNIP1,TRIM25,TSR1,UBA2,USP8,VIM,XPO1	98
**Cancer,Cell Death and Survival,Organismal Injury and Abnormalities,Tumor Morphology**	Cell death of osteosarcoma cells	5.6E-11		0.471	COPG1,CSE1L,EIF3B,EIF3E,EIF3F,EIF3L,KPNB1,RAN,RPL10,RPL13,RPL27A,RPL3,RPL6,RPL7,RPL7A,RPS14,RPS27A,SF3B1	18
**Cancer,Gastrointestinal Disease,Organismal Injury and Abnormalities**	Gastrointestinal adenocarcinoma	6.6E-11			ABCA3,ABCE1,ABCG1,ACAA1,ACLY,ACSL4,ACTA1,ACTR1A,ADSL,ADSSL1,AHCY,AHNAK,AKR1B1,AKR1B10,ALCAM,ALDH9A1,AP3B1,APEX1,ARAP1,ATIC,ATP1A1,ATP1B3,ATP2B1,ATP2C1,ATP6V0A1,ATP7A,C5AR1,CA2,CAND1,CCT2,CCT4,CCT7,CCT8,CDK1,CFL1,CLUH,CMAS,CMIP,CNDP2,CNOT1,COLEC12,COPB1,CORO1B,CORO1C,CORO7/CORO7-PAM16,CPD,CPNE8,CSE1L,CSNK1G3,CTSD,CTSK,DAAM1,DCTN1,DDX17,DDX21,DDX39B,DDX3X,DENND4B,DHX15,DHX29,DIS3,DKC1,DNAJA1,DNAJC13,DNM2,DNMT1,DOCK2,DPYSL2,ECPAS,EDIL3,EEF1D,EEF1G,EHD1,EIF2S3,EIF3A,EIF3B,EIF3C,EIF3D,EIF3E,EIF3F,EIF3I,EIF3L,EIF3M,ESYT1,FARSA,FARSB,FASN,FERMT3,FKBP4,FLNA,FLOT1,FLOT2,FYN,G3BP1,G6PD,GALK1,GARS,GART,GFPT1,GMDS,GMPS,GNA13,GNAI2,GNAS,GNB2,GPNMB,GRB2,GUSB,HIST1H1C,HK3,HLA-A,HNRNPL,HNRNPM,HSD17B4,HSP90AA1,HSP90AB1,HSPA4,ICAM1,IDE,IDH1,IFIT1B,IL6ST,IPO5,IPO7,ITCH,ITGAM,ITGB2,JAK1,KCNN4,KIDINS220,KPNA2,KPNB1,KRT1,KRT2,LGALS3BP,LIG1,LILRB4,LRP12,LYN,MAPK3,MARS,MCM2,MCM7,MFGE8,MOV10,MRI1,MSN,MTA2,MVP,MYH9,MYO1C,MYO1E,MYO1G,MYOF,NAA15,NANS,NCBP1,NCF2,NCL,NCSTN,NOP56,NOS2,NOTCH2,NPC1,NRAS,NUP93,PDS5A,PFAS,PGK1,PHGDH,PI4K2A,PKM,PLA2G4A,PLAU,PLEC,PLXNA1,PLXNB2,PNKP,POLD1,POLR1C,POLR2B,PPP1CA,PPP2R1A,PPP2R2A,PREP,PRIM2,PTGS2,PTPN23,PTPRA,PTPRC,PTPRJ,RAB5A,RAB5B,RAB8B,RALA,RAN,RANGAP1,RAP1B,RAP2C,RARS,RBBP7,RBPJ,RCC2,RHBDF2,RIPK3,RNF213,RNH1,RNMT,RPL10,RPL18,RPL18A,RPL28,RPL3,RPL4,RPL8,RPN1,RPS14,RPS27A,RPS6,RRP9,RTCB,SARS,SCAMP2,SCFD1,SEC24B,SF3B1,SFPQ,SHMT1,SHMT2,SIPA1,SIRPA,SLC12A4,SLC15A3,SLC16A3,SLC16A6,SLC1A5,SLC20A1,SLC23A2,SLC2A1,SLC38A2,SLC3A2,SLC4A7,SLC7A1,SLCO4A1,SLFN13,SMPDL3B,SMU1,SNAP23,SND1,SNRNP200,SNX2,SNX27,SPRED1,SQSTM1,SRM,STAT1,STEAP3,STOM,STRAP,STUB1,STX6,SUPT5H,TALDO1,TAX1BP1,TCIRG1,TCP1,TFRC,TGFBR2,TKT,TLR7,TMEM59,TNIP1,TOM1,TPI1,TPP2,TRIM14,TRIM28,TRPV2,TSG101,TTC37,TTLL12,UAP1L1,UBA1,UBA2,UCHL5,USP5,USP8,VASP,VIM,VPS13C,VWA5A,WARS,WDR82,WWP2,XPNPEP1,XPO1,XPO7,YBX1,ZC3HAV1	299

Table S4

**Cancer,Gastrointestinal Disease,Organismal Injury and Abnormalities**	Large intestine adenocarcinoma	7E-11			ABCA3,ABCE1,ABCG1,ACAA1,ACLY,ACSL4,ACTA1,ACTR1A,ADSL,ADSSL1,AHCY,AHNAK,AKR1B1,AKR1B10,ALCAM,ALDH9A1,AP3B1,APEX1,ARAP1,ATIC,ATP1A1,ATP1B3,ATP2B1,ATP2C1,ATP6V0A1,ATP7A,C5AR1,CA2,CAND1,CCT2,CCT4,CCT7,CCT8,CDK1,CFL1,CLUH,CMAS,CMIP,CNDP2,CNOT1,COLEC12,COPB1,CORO1B,CORO1C,CORO7/CORO7-PAM16,CPD,CPNE8,CSE1L,CSNK1G3,CTSD,CTSK,DAAM1,DCTN1,DDX17,DDX21,DDX39B,DENND4B,DHX15,DHX29,DIS3,DKC1,DNAJA1,DNAJC13,DNM2,DNMT1,DOCK2,DPYSL2,ECPAS,EDIL3,EEF1D,EEF1G,EHD1,EIF2S3,EIF3A,EIF3B,EIF3C,EIF3D,EIF3E,EIF3F,EIF3I,EIF3L,EIF3M,ESYT1,FARSA,FARSB,FASN,FERMT3,FKBP4,FLNA,FLOT1,FLOT2,FYN,G3BP1,G6PD,GALK1,GARS,GART,GFPT1,GMDS,GMPS,GNA13,GNAI2,GNAS,GNB2,GPNMB,GRB2,GUSB,HIST1H1C,HK3,HLA-A,HNRNPL,HNRNPM,HSD17B4,HSP90AA1,HSPA4,ICAM1,IDE,IDH1,IFIT1B,IL6ST,IPO5,IPO7,ITCH,ITGAM,ITGB2,JAK1,KCNN4,KIDINS220,KPNA2,KPNB1,KRT1,KRT2,LGALS3BP,LIG1,LILRB4,LRP12,LYN,MAPK3,MARS,MCM2,MCM7,MFGE8,MOV10,MRI1,MSN,MTA2,MVP,MYH9,MYO1C,MYO1E,MYO1G,MYOF,NAA15,NANS,NCBP1,NCF2,NCL,NCSTN,NOP56,NOS2,NOTCH2,NPC1,NRAS,NUP93,PDS5A,PFAS,PGK1,PI4K2A,PKM,PLA2G4A,PLAU,PLEC,PLXNA1,PLXNB2,PNKP,POLD1,POLR1C,POLR2B,PPP1CA,PPP2R1A,PPP2R2A,PREP,PRIM2,PTGS2,PTPN23,PTPRA,PTPRC,PTPRJ,RAB5A,RAB5B,RAB8B,RALA,RAN,RANGAP1,RAP1B,RAP2C,RARS,RBBP7,RBPJ,RCC2,RHBDF2,RIPK3,RNF213,RNH1,RNMT,RPL10,RPL18,RPL28,RPL3,RPL4,RPL8,RPN1,RPS14,RPS27A,RPS6,RRP9,RTCB,SARS,SCAMP2,SCFD1,SEC24B,SF3B1,SFPQ,SHMT1,SHMT2,SIPA1,SIRPA,SLC12A4,SLC15A3,SLC16A3,SLC16A6,SLC1A5,SLC20A1,SLC23A2,SLC2A1,SLC38A2,SLC3A2,SLC4A7,SLC7A1,SLCO4A1,SLFN13,SMPDL3B,SMU1,SNAP23,SND1,SNRNP200,SNX2,SNX27,SPRED1,SQSTM1,SRM,STAT1,STEAP3,STOM,STRAP,STUB1,STX6,SUPT5H,TALDO1,TAX1BP1,TCIRG1,TCP1,TFRC,TGFBR2,TKT,TLR7,TMEM59,TNIP1,TOM1,TPI1,TPP2,TRIM14,TRIM28,TRPV2,TSG101,TTC37,TTLL12,UAP1L1,UBA1,UBA2,UCHL5,USP5,USP8,VASP,VIM,VPS13C,VWA5A,WDR82,WWP2,XPNPEP1,XPO1,XPO7,YBX1,ZC3HAV1	294
**Nucleic Acid Metabolism**	Metabolism of nucleic acid component or derivative	7.1E-11		-1.485	ACACA,ACLY,ADSL,ADSSL1,AHCY,ATIC,ATP5F1B,ATP7A,C5AR1,CDK1,CMAS,CTPS1,DNMT1,DPYSL2,FASN,G6PD,GART,GFPT1,GMDS,GMPPB,GMPS,GNAS,GNB2,MTHFD1,NOS2,OLA1,PFAS,PGD,PGK1,PKM,PPAT,PTGS2,SHMT1,SLC23A2,SLC29A1,SNX5,TALDO1,TSTA3	38
**Cancer,Organismal Injury and Abnormalities**	Growth of tumor	7.6E-11		-0.012	ACLY,AHCY,AKR1B1,AKR1B10,ALCAM,CD36,CD47,CTSK,DNMT1,EIF3F,FASN,FCER1G,FERMT3,FLNA,GNA13,GPNMB,GRB2,HSPA4,ICAM1,Ifi202b,IFITM3,IL6ST,ITCH,ITGAM,KIDINS220,MEMO1,MFGE8,NDRG1,NOS2,NOTCH2,NRAS,PGD,PI4K2A,PKM,PLA2G4A,PLAU,PLEC,PPP2CA,PPP2R1A,PPP2R2A,PREP,PTGS2,PTPRA,RACK1,RALA,SLC16A3,SLC1A5,SLC20A1,SLC2A1,SLC3A2,SLC4A7,SQSTM1,STAT1,TGFBR2,TNFRSF1B,TRIM25,UBA2,XPO1,YBX1	59
**Cancer,Organismal Injury and Abnormalities**	Secondary tumor	8.3E-11		0.793	ALCAM,ATIC,C5AR1,CD36,CD47,COLEC12,CRYZ,CSE1L,DNMT1,DPYSL2,FASN,FERMT3,FLNA,FYN,GART,GNAS,GPNMB,HIST1H1C,HNRNPM,HSP90AA1,HSP90AB1,ICAM1,IDH1,IFITM3,JAK1,KIDINS220,LYN,MYO1C,NDRG1,NOS2,NRAS,NUP93,PCNA,PLAU,PLEC,POLD1,PPAT,PPP2R1A,PRIM2,PTGS2,PTPRA,PTPRC,PTPRJ,RAB31,RACK1,RNH1,RPL7,RPS27A,RPS6,SDCBP,SF3B1,SLC16A3,SQSTM1,STAT1,STEAP3,TGFBR2,TLR7,VIM,XPO1	59
**Cellular Movement,Hematological System Development and Function,Immune Cell Trafficking**	Cell movement of lymphocytes	8.4E-11		1.874	Abcb1b,ABCG1,ADAM8,CD47,DOCK2,DPYSL2,FERMT3,FLOT1,FYN,GNA13,GNAI2,GNAI3,GNAS,HLA-A,HNRNPL,ICAM1,IL6ST,ITGB2,JAK1,KCNN4,KRT10,MSN,MYADM,MYH9,MYO1G,NOS2,NRAS,PLAU,PLEC,PTGS2,PTPRA,RAP1B,STAT1,TGFBR2,TNFRSF1B,TNIP1	36
**Dermatological Diseases and Conditions,Inflammatory Disease,Inflammatory Response,Organismal Injury and Abnormalities**	Dermatitis	8.8E-11		0.572	AHCY,AHNAK,ATP1A1,C5AR1,CD47,CFL1,DPYSL2,EIF3E,ENO1,FCER1G,FKBP4,FLNA,FLOT1,FYN,GBA,HLA-A,ICAM1,IDE,ITGB2,JAK1,KCNN4,KRT1,KRT10,LYN,MSN,MTA2,NCSTN,NOS2,PHGDH,PTGS2,PTPRC,RBPJ,STAT1,TAX1BP1,TLR7,TNIP1,TPI1	37

Table S4

**Cellular Movement,Immune Cell Trafficking**	Cell movement of lymphatic system cells	1E-10	Increased	2.137	Abcb1b,ABCG1,ADAM8,ALCAM,CD47,DOCK2,DPYSL2,FERMT3,FLOT1,FYN,GNA13,GNAI2,GNAI3,GNAS,HLA-A,HNRNPL,ICAM1,IL6ST,ITGB2,JAK1,KCNN4,KRT10,MSN,MYADM,MYH9,MYO1G,NOS2,NRAS,PLAU,PLEC,PTGS2,PTPRA,RAP1B,STAT1,TGFBR2,TNFRSF1B,TNIP1	37
**Cancer,Gastrointestinal Disease,Organismal Injury and Abnormalities**	Intestinal carcinoma	1.1E-10			ABCA3,ABCE1,ABCG1,ACAA1,ACLY,ACSL4,ACTA1,ACTR1A,ADSL,ADSSL1,AHCY,AHNAK,AKR1B1,AKR1B10,ALCAM,ALDH9A1,AP3B1,APEX1,ARAP1,ATIC,ATP1A1,ATP1B3,ATP2B1,ATP2C1,ATP6V0A1,ATP7A,C5AR1,CA2,CAND1,CCT2,CCT4,CCT7,CCT8,CDK1,CFL1,CLUH,CMAS,CMIP,CNDP2,CNOT1,COLEC12,COPB1,CORO1B,CORO1C,CORO7/CORO7-PAM16,CPD,CPNE8,CSE1L,CSNK1G3,CTSD,CTSK,DAAM1,DCTN1,DDX17,DDX21,DDX39B,DENND4B,DHX15,DHX29,DIS3,DKC1,DNAJA1,DNAJC13,DNM2,DNMT1,DOCK2,DPYSL2,ECPAS,EDIL3,EEF1D,EEF1G,EHD1,EIF2S3,EIF3A,EIF3B,EIF3C,EIF3D,EIF3E,EIF3F,EIF3I,EIF3L,EIF3M,ESYT1,FARSA,FARSB,FASN,FERMT3,FKBP4,FLNA,FLOT1,FLOT2,FYN,G3BP1,G6PD,GALK1,GARS,GART,GFPT1,GMDS,GMPS,GNA13,GNAI2,GNAS,GNB2,GPNMB,GRB2,GUSB,HIST1H1C,HK3,HLA-A,HNRNPL,HNRNPM,HSD17B4,HSP90AA1,HSP90AB1,HSPA4,ICAM1,IDE,IDH1,IFIT1B,IL6ST,IPO5,IPO7,ITCH,ITGAM,ITGB2,JAK1,KCNN4,KIDINS220,KPNA2,KPNB1,KRT1,KRT2,LGALS3BP,LIG1,LILRB4,LRP12,LYN,MAPK3,MARS,MCM2,MCM7,MFGE8,MOV10,MRI1,MSN,MTA2,MVP,MYH9,MYO1C,MYO1E,MYO1G,MYOF,NAA15,NANS,NCBP1,NCF2,NCL,NCSTN,NOP56,NOS2,NOTCH2,NPC1,NRAS,NUP93,PDS5A,PFAS,PGK1,PI4K2A,PKM,PLA2G4A,PLAU,PLEC,PLXNA1,PLXNB2,PNKP,POLD1,POLR1C,POLR2B,PPP1CA,PPP2R1A,PPP2R2A,PREP,PRIM2,PTGS2,PTPN23,PTPRA,PTPRC,PTPRJ,RAB5A,RAB5B,RAB8B,RALA,RAN,RANGAP1,RAP1B,RAP2C,RARS,RBBP7,RBPJ,RCC2,RHBDF2,RIPK3,RNF213,RNH1,RNMT,RPL10,RPL18,RPL28,RPL3,RPL4,RPL8,RPN1,RPS14,RPS27A,RPS6,RRP9,RTCB,SARS,SCAMP2,SCFD1,SEC24B,SF3B1,SFPQ,SHMT1,SHMT2,SIPA1,SIRPA,SLC12A4,SLC15A3,SLC16A3,SLC16A6,SLC1A5,SLC20A1,SLC23A2,SLC2A1,SLC38A2,SLC3A2,SLC4A7,SLC7A1,SLCO4A1,SLFN13,SMPDL3B,SMU1,SNAP23,SND1,SNRNP200,SNX2,SNX27,SPRED1,SQSTM1,SRM,STAT1,STEAP3,STOM,STRAP,STUB1,STX6,SUPT5H,TALDO1,TAX1BP1,TCIRG1,TCP1,TFRC,TGFBR2,TKT,TLR7,TMEM59,TNIP1,TOM1,TPI1,TPP2,TRIM14,TRIM28,TRPV2,TSG101,TTC37,TTLL12,UAP1L1,UBA1,UBA2,UCHL5,USP5,USP8,VASP,VIM,VPS13C,VWA5A,WDR82,WWP2,XPNPEP1,XPO1,XPO7,YBX1,ZC3HAV1	295
**Cancer,Hematological Disease,Organismal Injury and Abnormalities**	Lymphocytic neoplasm	1.1E-10	Decreased	-2.183	ABCG1,ADSL,AHNAK,ALDH9A1,ATIC,CA2,CCT3,CCT7,CD36,CD47,CDK1,CFL1,CMIP,CNOT1,CPD,CSE1L,CTSK,DDX3X,DIS3,DNM1L,DNM2,DNMT1,DOCK2,EIF2A,FASN,FLNA,FYN,G3BP1,GART,GNA13,GNAI2,GRB2,HIST1H1C,HLA-A,HNRNPM,HNRNPU,HSP90AA1,HSP90AB1,ICAM1,IDH1,IL6ST,IPO5,IPO7,ITGAM,ITGB2,JAK1,KPNA2,KPNB1,KRT1,KRT10,KRT2,LYN,MAPK3,MARS,MPEG1,MYO1G,MYOF,NOS2,NOTCH2,NRAS,OASL,PCNA,PLXNB2,POLD1,PPAT,PPP2CA,PREP,PRIM2,PRPF19,PTGS2,PTPRC,RALA,RAN,RNF213,RPL10,RPL13,RPS6,RPSA,SARS,SF3B1,SHMT1,SHMT2,SLC2A1,SPRED1,STAT1,STEAP3,TAX1BP1,TFRC,TGFBR2,TLR7,TNIP1,TRIM25,TSR1,UBA2,USP8,VIM,XPO1	97
**Cancer,Hematological Disease,Organismal Injury and Abnormalities**	Lymphohematopoietic neoplasia	1.1E-10		-1.698	ABCG1,ACSL4,ADSL,AHNAK,ALDH9A1,APEX1,ARAP1,ATIC,ATP1A1,CA2,CCT3,CCT7,CD36,CD47,CDK1,CFL1,CLUH,CMIP,CNOT1,CPD,CSE1L,CTSK,DAAM1,DDX3X,DHX15,DIS3,DNAJA1,DNM1L,DNM2,DNMT1,DOCK2,ECPAS,EEF1D,EIF2A,EIF3C,EIF3D,EIF3I,EIF3L,FASN,FCER1G,FLNA,FYN,G3BP1,GART,GMDS,GNA13,GNAI2,GNAS,GRB2,HIST1H1C,HK3,HLA-A,HNRNPM,HNRNPU,HSP90AA1,HSP90AB1,HSPA4,ICAM1,IDH1,IL6ST,IPO5,IPO7,ITGAM,ITGB2,JAK1,KPNA2,KPNB1,KRT1,KRT10,KRT2,LILRB4,LYN,MAPK3,MARS,MCM7,MPEG1,MYH9,MYO1E,MYO1G,MYOF,NANS,NCSTN,NOS2,NOTCH2,NRAS,OASL,PCNA,PDS5A,PKM,PLAU,PLXNB2,POLD1,PPAT,PPP1R7,PPP2CA,PREP,PRIM2,PRPF19,PTGS2,PTPRA,PTPRC,RACK1,RALA,RAN,RIPK3,RNF213,RNPEP,RPL10,RPL13,RPL28,RPL3,RPL4,RPL6,RPL7,RPS14,RPS27A,RPS6,RPSA,SARS,SDCBP,SF3B1,SHMT1,SHMT2,SIPA1,SLC2A1,SMU1,SPRED1,STAT1,STEAP3,SUPT5H,TAX1BP1,TFRC,TGFBR2,TLR7,TNIP1,TPI1,TRIM25,TSR1,UBA2,USP8,VIM,XPO1,XPO7	143
**Cancer,Hematological Disease,Organismal Injury and Abnormalities**	Hematopoietic neoplasm	1.5E-10		-1.698	ABCG1,ACSL4,ADSL,AHNAK,ALDH9A1,APEX1,ARAP1,ATIC,ATP1A1,CA2,CCT3,CCT7,CD36,CD47,CDK1,CFL1,CLUH,CMIP,CNOT1,CPD,CSE1L,CTSK,DAAM1,DDX3X,DHX15,DIS3,DNAJA1,DNM1L,DNM2,DNMT1,DOCK2,ECPAS,EEF1D,EIF2A,EIF3C,EIF3D,EIF3I,EIF3L,FASN,FCER1G,FLNA,FYN,G3BP1,GART,GMDS,GNA13,GNAI2,GNAS,GRB2,HIST1H1C,HK3,HLA-A,HNRNPM,HNRNPU,HSP90AA1,HSP90AB1,HSPA4,ICAM1,IDH1,IL6ST,IPO5,IPO7,ITGAM,ITGB2,JAK1,KPNA2,KPNB1,KRT1,KRT10,KRT2,LILRB4,LYN,MAPK3,MARS,MCM7,MPEG1,MYH9,MYO1E,MYO1G,MYOF,NANS,NCSTN,NOS2,NOTCH2,NRAS,OASL,PCNA,PDS5A,PKM,PLXNB2,POLD1,PPAT,PPP1R7,PPP2CA,PREP,PRIM2,PRPF19,PTGS2,PTPRA,PTPRC,RACK1,RALA,RAN,RIPK3,RNF213,RNPEP,RPL10,RPL13,RPL28,RPL3,RPL4,RPL6,RPL7,RPS14,RPS27A,RPS6,RPSA,SARS,SDCBP,SF3B1,SHMT1,SHMT2,SIPA1,SLC2A1,SMU1,SPRED1,STAT1,STEAP3,SUPT5H,TAX1BP1,TFRC,TGFBR2,TLR7,TNIP1,TPI1,TRIM25,TSR1,UBA2,USP8,VIM,XPO1,XPO7	142
**RNA Post-Transcriptional Modification**	Processing of RNA	1.6E-10		-1.192	AHNAK,CMTR1,CPSF1,DDX17,DDX39B,DHX15,DIS3,DKC1,HNRNPL,HNRNPM,HNRNPU,MTREX,NCBP1,NOP56,POLR2A,POLR2B,PRPF19,PRPF4,RNMT,RPL14,RPL26,RPL7,RPS14,RPS6,RPS9,RRP9,SARS,SF3B1,SFPQ,SMU1,SNRNP200,SNRNP40,SUPT5H,YBX1	34

Table S4

**Cancer,Cell Death and Survival,Organismal Injury and Abnormalities,Tumor Morphology**	Cell death of cancer cells	1.7E-10		0.448	ACLY,CD47,CDK1,COPG1,CSE1L,EIF3B,EIF3C,EIF3E,EIF3F,EIF3L,ENO1,FASN,HSP90AB1,Ifi202b,IL6ST,KPNB1,MCM2,NDRG1,NRAS,PTGS2,PTPRA,RAN,RBPJ,RPL10,RPL13,RPL27A,RPL3,RPL6,RPL7,RPL7A,RPS14,RPS27A,SF3B1,TLR7	34
**Cancer,Hematological Disease,Organismal Injury and Abnormalities**	Lymphohematopoietic cancer	1.9E-10	Decreased	-2.052	ABCG1,ACSL4,ADSL,AHNAK,ALDH9A1,APEX1,ARAP1,ATIC,ATP1A1,CA2,CCT3,CCT7,CD36,CD47,CDK1,CFL1,CLUH,CMIP,CNOT1,CPD,CSE1L,CTSK,DAAM1,DDX3X,DHX15,DIS3,DNAJA1,DNM1L,DNM2,DNMT1,DOCK2,ECPAS,EEF1D,EIF2A,EIF3C,EIF3D,EIF3I,EIF3L,FASN,FCER1G,FLNA,FYN,G3BP1,GART,GMDS,GNA13,GNAI2,GNAS,GRB2,HIST1H1C,HK3,HLA-A,HNRNPM,HNRNPU,HSP90AA1,HSP90AB1,HSPA4,ICAM1,IDH1,IL6ST,IPO5,IPO7,ITGAM,ITGB2,JAK1,KPNA2,KPNB1,KRT1,KRT10,KRT2,LILRB4,LYN,MAPK3,MARS,MCM7,MPEG1,MYH9,MYO1E,MYO1G,MYOF,NANS,NCSTN,NOS2,NOTCH2,NRAS,OASL,PCNA,PDS5A,PLAU,PLXNB2,POLD1,PPAT,PPP1R7,PPP2CA,PRIM2,PRPF19,PTGS2,PTPRA,PTPRC,RACK1,RALA,RAN,RIPK3,RNF213,RNPEP,RPL10,RPL13,RPL28,RPL3,RPL4,RPL6,RPL7,RPS14,RPS27A,RPS6,RPSA,SARS,SDCBP,SF3B1,SHMT1,SHMT2,SIPA1,SLC2A1,SMU1,SPRED1,STAT1,STEAP3,SUPT5H,TAX1BP1,TFRC,TGFBR2,TLR7,TNIP1,TPI1,TRIM25,TSR1,UBA2,USP8,VIM,XPO1,XPO7	141
**Cancer,Gastrointestinal Disease,Organismal Injury and Abnormalities**	Large intestine neoplasm	1.9E-10		0.221	ABCA3,ABCE1,ABCG1,ACAA1,ACLY,ACSL4,ACTA1,ACTR1A,ADSL,ADSSL1,AHCY,AHNAK,AKR1B1,AKR1B10,ALCAM,ALDH9A1,AP3B1,APEX1,ARAP1,ATIC,ATP1A1,ATP1B3,ATP2B1,ATP2C1,ATP6V0A1,ATP7A,C5AR1,CA2,CAND1,CCT2,CCT4,CCT5,CCT7,CCT8,CD36,CDK1,CFL1,CLUH,CMAS,CMIP,CNDP2,CNOT1,COLEC12,COPB1,CORO1B,CORO1C,CORO7/CORO7-PAM16,CPD,CPNE8,CRYZ,CSE1L,CSNK1G3,CTSD,CTSK,DAAM1,DCTN1,DDX17,DDX21,DDX39B,DENND4B,DHX15,DHX29,DIS3,DKC1,DNAJA1,DNAJC13,DNM2,DNMT1,DOCK2,DPYSL2,ECPAS,EDIL3,EEF1D,EEF1G,EHD1,EIF2S3,EIF3A,EIF3B,EIF3C,EIF3D,EIF3E,EIF3F,EIF3I,EIF3L,EIF3M,ENO1,ESYT1,FARSA,FARSB,FASN,FERMT3,FKBP4,FLNA,FLOT1,FLOT2,FYN,G3BP1,G6PD,GALK1,GARS,GART,GFPT1,GMDS,GMPS,GNA13,GNAI2,GNAS,GNB2,GPNMB,GRB2,GUSB,HIST1H1C,HK3,HLA-A,HNRNPL,HNRNPM,HSD17B4,HSP90AA1,HSP90AB1,HSPA4,ICAM1,IDE,IDH1,IFIT1B,IFITM3,IL6ST,IPO5,IPO7,ITCH,ITGAM,ITGB2,JAK1,KCNN4,KIDINS220,KPNA2,KPNB1,KRT1,KRT2,LGALS3BP,LIG1,LILRB4,LRP12,LYN,MAPK3,MARS,MAT2A,MCM2,MCM7,MFGE8,MOV10,MRI1,MSN,MTA2,MVP,MYH9,MYO1C,MYO1E,MYO1G,MYOF,NAA15,NANS,NCBP1,NCF2,NCL,NCSTN,NDRG1,NOP56,NOS2,NOTCH2,NPC1,NRAS,NUP93,PCNA,PDS5A,PFAS,PGK1,PI4K2A,PKM,PLA2G4A,PLAU,PLEC,PLXNA1,PLXNB2,PNKP,POLD1,POLR1C,POLR2B,PPP1CA,PPP2R1A,PPP2R2A,PREP,PRIM2,PTGS2,PTPN23,PTPRA,PTPRC,PTPRJ,RAB31,RAB5A,RAB5B,RAB8B,RALA,RAN,RANGAP1,RAP1B,RAP2C,RARS,RBBP7,RBPJ,RCC2,RHBDF2,RIPK3,RNF213,RNH1,RNMT,RPL10,RPL18,RPL28,RPL3,RPL4,RPL8,RPN1,RPS14,RPS27A,RPS6,RRP9,RTCB,SARS,SCAMP2,SCFD1,SEC24B,SF3B1,SFPQ,SHMT1,SHMT2,SIPA1,SIRPA,SLC12A4,SLC15A3,SLC16A3,SLC16A6,SLC1A5,SLC20A1,SLC23A2,SLC29A1,SLC2A1,SLC38A2,SLC3A2,SLC4A7,SLC7A1,SLCO4A1,SLFN13,SMPDL3B,SMU1,SNAP23,SND1,SNRNP200,SNX2,SNX27,SPRED1,SQSTM1,SRM,STAT1,STEAP3,STOM,STRAP,STUB1,STX6,SUPT5H,TALDO1,TAX1BP1,TCIRG1,TCP1,TFRC,TGFBR2,TKT,TLR7,TMEM59,TNIP1,TOM1,TPI1,TPP2,TRIM14,TRIM28,TRPV2,TSG101,TTC37,TTLL12,UAP1L1,UBA1,UBA2,UCHL5,USP5,USP8,VASP,VIM,VPS13C,VWA5A,WDR82,WWP2,XPNPEP1,XPO1,XPO7,YBX1,ZC3HAV1	305
**Cellular Movement,Hematological System Development and Function,Immune Cell Trafficking**	Migration of mononuclear leukocytes	2E-10		1.096	Abcb1b,CD47,DOCK2,DPYSL2,FERMT3,FLOT1,FYN,GNA13,GNAI2,GNAI3,HLA-A,HNRNPL,ICAM1,IL6ST,ITGAM,ITGB2,JAK1,KCNN4,MSN,MYADM,MYH9,MYO1G,NOS2,NRAS,PLAU,PLEC,PTGS2,PTPRA,RAP1B,SIRPA,STAT1,TGFBR2,TNFRSF1B,TNIP1	34
**Cancer,Hematological Disease,Organismal Injury and Abnormalities**	Lymphoid cancer	2E-10	Decreased	-2.183	ABCG1,ADSL,AHNAK,ALDH9A1,ATIC,CA2,CCT3,CCT7,CD36,CD47,CDK1,CFL1,CMIP,CNOT1,CPD,CSE1L,CTSK,DDX3X,DIS3,DNM1L,DNM2,DNMT1,DOCK2,EIF2A,FASN,FLNA,FYN,G3BP1,GART,GNA13,GNAI2,GRB2,HIST1H1C,HLA-A,HNRNPM,HNRNPU,HSP90AA1,HSP90AB1,ICAM1,IDH1,IL6ST,IPO5,IPO7,ITGAM,ITGB2,JAK1,KPNA2,KPNB1,KRT1,KRT10,KRT2,LYN,MAPK3,MARS,MPEG1,MYO1G,MYOF,NOS2,NOTCH2,NRAS,OASL,PCNA,PLAU,PLXNB2,POLD1,PPAT,PPP2CA,PRIM2,PRPF19,PTGS2,PTPRC,RALA,RAN,RNF213,RPL10,RPL13,RPS6,RPSA,SARS,SF3B1,SHMT1,SHMT2,SLC2A1,SPRED1,STAT1,STEAP3,TAX1BP1,TFRC,TGFBR2,TLR7,TNIP1,TRIM25,TSR1,UBA2,USP8,VIM,XPO1	97

Table S4

**Cancer,Gastrointestinal Disease,Organismal Injury and Abnormalities**	Malignant neoplasm of large intestine	2.3E-10		-1	ABCA3,ABCE1,ABCG1,ACAA1,ACLY,ACSL4,ACTA1,ACTR1A,ADSL,ADSSL1,AHCY,AHNAK,AKR1B1,AKR1B10,ALCAM,ALDH9A1,AP3B1,APEX1,ARAP1,ATIC,ATP1A1,ATP1B3,ATP2B1,ATP2C1,ATP6V0A1,ATP7A,C5AR1,CA2,CAND1,CCT2,CCT4,CCT5,CCT7,CCT8,CD36,CDK1,CFL1,CLUH,CMAS,CMIP,CNDP2,CNOT1,COLEC12,COPB1,CORO1B,CORO1C,CORO7/CORO7-PAM16,CPD,CPNE8,CRYZ,CSE1L,CSNK1G3,CTSD,CTSK,DAAM1,DCTN1,DDX17,DDX21,DDX39B,DENND4B,DHX15,DHX29,DIS3,DKC1,DNAJA1,DNAJC13,DNM2,DNMT1,DOCK2,DPYSL2,ECPAS,EDIL3,EEF1D,EEF1G,EHD1,EIF2S3,EIF3A,EIF3B,EIF3C,EIF3D,EIF3E,EIF3F,EIF3I,EIF3L,EIF3M,ENO1,ESYT1,FARSA,FARSB,FASN,FERMT3,FKBP4,FLNA,FLOT1,FLOT2,FYN,G3BP1,G6PD,GALK1,GARS,GART,GFPT1,GMDS,GMPS,GNA13,GNAI2,GNAS,GNB2,GPNMB,GRB2,GUSB,HIST1H1C,HK3,HLA-A,HNRNPL,HNRNPM,HSD17B4,HSP90AA1,HSP90AB1,HSPA4,ICAM1,IDE,IDH1,IFIT1B,IFITM3,IL6ST,IPO5,IPO7,ITCH,ITGAM,ITGB2,JAK1,KCNN4,KIDINS220,KPNA2,KPNB1,KRT1,KRT2,LGALS3BP,LIG1,LILRB4,LRP12,LYN,MAPK3,MARS,MAT2A,MCM2,MCM7,MFGE8,MOV10,MRI1,MSN,MTA2,MVP,MYH9,MYO1C,MYO1E,MYO1G,MYOF,NAA15,NANS,NCBP1,NCF2,NCL,NCSTN,NDRG1,NOP56,NOS2,NOTCH2,NPC1,NRAS,NUP93,PCNA,PDS5A,PFAS,PGK1,PI4K2A,PKM,PLA2G4A,PLAU,PLEC,PLXNA1,PLXNB2,PNKP,POLD1,POLR1C,POLR2B,PPP1CA,PPP2R1A,PPP2R2A,PREP,PRIM2,PTGS2,PTPN23,PTPRA,PTPRC,PTPRJ,RAB31,RAB5A,RAB5B,RAB8B,RALA,RAN,RANGAP1,RAP1B,RAP2C,RARS,RBBP7,RBPJ,RCC2,RHBDF2,RIPK3,RNF213,RNH1,RNMT,RPL10,RPL18,RPL28,RPL3,RPL4,RPL8,RPN1,RPS14,RPS27A,RPS6,RRP9,RTCB,SARS,SCAMP2,SCFD1,SEC24B,SF3B1,SFPQ,SHMT1,SHMT2,SIPA1,SIRPA,SLC12A4,SLC15A3,SLC16A3,SLC16A6,SLC1A5,SLC20A1,SLC23A2,SLC2A1,SLC38A2,SLC3A2,SLC4A7,SLC7A1,SLCO4A1,SLFN13,SMPDL3B,SMU1,SNAP23,SND1,SNRNP200,SNX2,SNX27,SPRED1,SQSTM1,SRM,STAT1,STEAP3,STOM,STRAP,STUB1,STX6,SUPT5H,TALDO1,TAX1BP1,TCIRG1,TCP1,TFRC,TGFBR2,TKT,TLR7,TMEM59,TNIP1,TOM1,TPI1,TPP2,TRIM14,TRIM28,TRPV2,TSG101,TTC37,TTLL12,UAP1L1,UBA1,UBA2,UCHL5,USP5,USP8,VASP,VIM,VPS13C,VWA5A,WDR82,WWP2,XPNPEP1,XPO1,XPO7,YBX1,ZC3HAV1	304
**Cancer,Hematological Disease,Organismal Injury and Abnormalities**	Hematologic cancer	2.4E-10	Decreased	-2.052	ABCG1,ACSL4,ADSL,AHNAK,ALDH9A1,APEX1,ARAP1,ATIC,ATP1A1,CA2,CCT3,CCT7,CD36,CD47,CDK1,CFL1,CLUH,CMIP,CNOT1,CPD,CSE1L,CTSK,DAAM1,DDX3X,DHX15,DIS3,DNAJA1,DNM1L,DNM2,DNMT1,DOCK2,ECPAS,EEF1D,EIF2A,EIF3C,EIF3D,EIF3I,EIF3L,FASN,FCER1G,FLNA,FYN,G3BP1,GART,GMDS,GNA13,GNAI2,GNAS,GRB2,HIST1H1C,HK3,HLA-A,HNRNPM,HNRNPU,HSP90AA1,HSP90AB1,HSPA4,ICAM1,IDH1,IL6ST,IPO5,IPO7,ITGAM,ITGB2,JAK1,KPNA2,KPNB1,KRT1,KRT10,KRT2,LILRB4,LYN,MAPK3,MARS,MCM7,MPEG1,MYH9,MYO1E,MYO1G,MYOF,NANS,NCSTN,NOS2,NOTCH2,NRAS,OASL,PCNA,PDS5A,PLXNB2,POLD1,PPAT,PPP1R7,PPP2CA,PRIM2,PRPF19,PTGS2,PTPRA,PTPRC,RACK1,RALA,RAN,RIPK3,RNF213,RNPEP,RPL10,RPL13,RPL28,RPL3,RPL4,RPL6,RPL7,RPS14,RPS27A,RPS6,RPSA,SARS,SDCBP,SF3B1,SHMT1,SHMT2,SIPA1,SLC2A1,SMU1,SPRED1,STAT1,STEAP3,SUPT5H,TAX1BP1,TFRC,TGFBR2,TLR7,TNIP1,TPI1,TRIM25,TSR1,UBA2,USP8,VIM,XPO1,XPO7	140
**Cellular Movement,Hematological System Development and Function,Immune Cell Trafficking**	Cell movement of mononuclear leukocytes	2.6E-10		1.741	Abcb1b,ABCG1,ADAM8,CD47,DOCK2,DPYSL2,FERMT3,FLOT1,FYN,GNA13,GNAI2,GNAI3,GNAS,HLA-A,HNRNPL,ICAM1,IL6ST,ITGAM,ITGB2,JAK1,KCNN4,KRT10,MSN,MYADM,MYH9,MYO1G,NOS2,NRAS,PLAU,PLEC,PTGS2,PTPRA,RAP1B,SIRPA,STAT1,TCIRG1,TGFBR2,TNFRSF1B,TNIP1	39
**Immunological Disease**	Hypersensitive reaction	3.7E-10	Increased	2.221	AHCY,AHNAK,ALCAM,CD47,CFL1,CORO1B,DPYSL2,EIF3E,ENO1,FCER1G,FKBP4,FLNA,FLOT1,FYN,GARS,HLA-A,ICAM1,IDE,ITGB2,JAK1,KRT1,KRT10,LGALS3BP,LILRB4,LYN,MSN,NOS2,PHGDH,PLA2G4A,PTGS2,PTPRC,SRM,STAT1,TAX1BP1,TCIRG1,TPI1	36
**Cellular Development,Cellular Growth and Proliferation**	Proliferation of blood cells	3.9E-10		0.571	ABCG1,ACLY,ACTL6A,AHNAK,C5AR1,CD36,CD47,CTPS1,CTSD,DNM2,DOCK2,FCER1G,FYN,GNAI2,GPNMB,HNRNPL,ICAM1,IL6ST,Irgm1,ITCH,ITGAM,ITGB2,JAK1,KCNN4,LILRB4,LYN,MAPK3,MSN,MTA2,MVP,MYH9,NCSTN,NOS2,NOTCH2,NPC1,NRAS,PLAU,PPAT,PTGS2,PTPRC,PTPRJ,RHBDF2,RIPK3,SF3B1,SLC29A1,SLC3A2,SLC7A1,STAM2,STAT1,TALDO1,TCIRG1,TFRC,TGFBR2,TLR7,TNFRSF1B,TPP2,WWP2	57

Table S4

**Cardiovascular Disease,Hematological Disease,Organismal Injury and Abnormalities**	Anemia	4.1E-10		-1.963	ACTL6A,CD47,DNMT1,FCER1G,G6PD,GNAS,ICAM1,IDH1,IL6ST,KCNN4,LIG1,LYN,MTHFD1,NRAS,POLD1,PPAT,PRIM2,PTGS2,PTPRC,PTPRJ,RPL26,RPS14,RPS26,RPS6,SF3B1,SIPA1,SLC12A4,SLC20A1,SLC29A1,SLC2A1,SLC7A1,STEAP3,TFRC,TNIP1,TPP2,TRIM28	36
**Cellular Movement,Immune Cell Trafficking**	Migration of lymphatic system cells	4.7E-10		1.15	Abcb1b,ALCAM,CD47,DOCK2,DPYSL2,FERMT3,FLOT1,FYN,GNA13,GNAI2,GNAI3,HLA-A,HNRNPL,ICAM1,IL6ST,ITGB2,JAK1,KCNN4,MSN,MYADM,MYH9,MYO1G,NOS2,NRAS,PLAU,PLEC,PTGS2,PTPRA,RAP1B,STAT1,TGFBR2,TNFRSF1B,TNIP1	33
**Cellular Movement,Hematological System Development and Function,Immune Cell Trafficking**	Lymphocyte migration	5E-10		1.015	Abcb1b,CD47,DOCK2,DPYSL2,FERMT3,FLOT1,FYN,GNA13,GNAI2,GNAI3,HLA-A,HNRNPL,ICAM1,IL6ST,ITGB2,JAK1,KCNN4,MSN,MYADM,MYH9,MYO1G,NOS2,NRAS,PLAU,PLEC,PTGS2,PTPRA,RAP1B,STAT1,TGFBR2,TNFRSF1B,TNIP1	32
**Cell Death and Survival**	Cell viability	7.5E-10		0.587	Abcb1b,ACLY,ACTL6A,APEX1,ATP7A,CD47,CDK1,CSE1L,CTSD,DNM1L,DNMT1,EIF2A,EIF3A,EIF3C,ENO1,FASN,FCER1G,GPNMB,GRB2,HLA-A,ICAM1,IDE,IDH1,IL6ST,ITGB2,JAK1,LIG1,LYN,MAPK3,MCM2,MCM7,MVP,NCF2,NDRG1,NOS2,NPC1,NRAS,PCNA,PLA2G4A,PLAU,PNKP,PPP1CA,PPP2R2A,PTGS2,PTPRA,PTPRC,RAB5A,RBPJ,RIPK3,SF3B1,SIPA1,SND1,STAM2,STAT1,TNFRSF1B,TRIM28,XPO1	57
**Cancer,Gastrointestinal Disease,Organismal Injury and Abnormalities**	Gastrointestinal tumor	8.3E-10		-1.503	ABCA3,ABCE1,ABCG1,ACAA1,ACLY,ACSL4,ACTA1,ACTR1A,ADSL,ADSSL1,AHCY,AHNAK,AKR1B1,AKR1B10,ALCAM,ALDH9A1,AMDHD2,AP3B1,APEX1,ARAP1,ATIC,ATP1A1,ATP1B3,ATP2B1,ATP2C1,ATP6V0A1,ATP7A,C5AR1,CA2,CAND1,CCT2,CCT4,CCT5,CCT7,CCT8,CD36,CDK1,CFL1,CLUH,CMAS,CMIP,CNDP2,CNOT1,COLEC12,COPB1,CORO1B,CORO1C,CORO7/CORO7-PAM16,CPD,CPNE8,CRYZ,CSE1L,CSNK1G3,CTPS1,CTSD,CTSK,DAAM1,DCTN1,DDX17,DDX21,DDX39B,DDX3X,DENND4B,DHX15,DHX29,DIS3,DKC1,DNAJA1,DNAJC13,DNM2,DNMT1,DOCK2,DPYSL2,ECPAS,EDIL3,EEF1D,EEF1G,EHD1,EIF2S3,EIF3A,EIF3B,EIF3C,EIF3D,EIF3E,EIF3F,EIF3I,EIF3L,EIF3M,ENO1,ESYT1,FARSA,FARSB,FASN,FERMT3,FKBP4,FLNA,FLOT1,FLOT2,FYN,G3BP1,G6PD,GALK1,GARS,GART,GFPT1,GMDS,GMPS,GNA13,GNAI2,GNAS,GNB2,GPNMB,GRB2,GUSB,HIST1H1C,HK3,HLA-A,HNRNPL,HNRNPM,HSD17B4,HSP90AA1,HSP90AB1,HSPA4,ICAM1,IDE,IDH1,IFIT1B,IFITM3,IL6ST,IPO5,IPO7,ITCH,ITGAM,ITGB2,JAK1,KCNN4,KIDINS220,KPNA2,KPNB1,KRT1,KRT2,LGALS3BP,LIG1,LILRB4,LRP12,LYN,MAPK3,MARS,MAT2A,MCM2,MCM7,MFGE8,MOV10,MRI1,MSN,MTA2,MVP,MYH9,MYO1C,MYO1E,MYO1G,MYOF,NAA15,NANS,NCBP1,NCF2,NCL,NCSTN,NDRG1,NOP56,NOS2,NOTCH2,NPC1,NRAS,NUP93,OLA1,PCNA,PDS5A,PFAS,PGK1,PHGDH,PI4K2A,PKM,PLA2G4A,PLAU,PLEC,PLXNA1,PLXNB2,PNKP,POLD1,POLR1C,POLR2B,PPP1CA,PPP2CA,PPP2R1A,PPP2R2A,PREP,PRIM2,PTGS2,PTPN23,PTPRA,PTPRC,PTPRJ,RAB31,RAB5A,RAB5B,RAB8B,RALA,RAN,RANGAP1,RAP1B,RAP2C,RARS,RBBP7,RBPJ,RCC2,RHBDF2,RIPK3,RNF213,RNH1,RNMT,RPL10,RPL18,RPL18A,RPL28,RPL3,RPL4,RPL8,RPN1,RPS14,RPS27A,RPS6,RRP9,RTCB,SARS,SCAMP2,SCFD1,SEC24B,SF3B1,SFPQ,SHMT1,SHMT2,SIPA1,SIRPA,SLC12A4,SLC15A3,SLC16A3,SLC16A6,SLC1A5,SLC20A1,SLC23A2,SLC29A1,SLC2A1,SLC38A2,SLC3A2,SLC4A7,SLC7A1,SLCO4A1,SLFN13,SMPDL3B,SMU1,SNAP23,SND1,SNRNP200,SNX2,SNX27,SPRED1,SQSTM1,SRM,STAT1,STEAP3,STOM,STRAP,STUB1,STX6,SUPT5H,TALDO1,TAX1BP1,TCIRG1,TCP1,TFRC,TGFBR2,TKT,TLR7,TMEM59,TNIP1,TOM1,TPI1,TPP2,TRIM14,TRIM28,TRPV2,TSG101,TTC37,TTLL12,UAP1L1,UBA1,UBA2,UCHL5,USP5,USP8,VASP,VIM,VPS13C,VWA5A,WARS,WDR82,WWP2,XPNPEP1,XPO1,XPO7,YBX1,ZC3HAV1	313

Table S4

**Cellular Development,Cellular Growth and Proliferation,Hematological System Development and Function,Lymphoid Tissue Structure and Development**	Cell proliferation of T lymphocytes	8.6E-10		1.505	ABCG1,AHNAK,C5AR1,CD36,CD47,CTPS1,CTSD,DNM2,DOCK2,FCER1G,FYN,GNAI2,GPNMB,HNRNPL,ICAM1,IL6ST,Irgm1,ITCH,ITGAM,ITGB2,KCNN4,LILRB4,MAPK3,MSN,MTA2,MVP,NCSTN,NOS2,PLAU,PPAT,PTGS2,PTPRC,PTPRJ,RIPK3,SLC3A2,SLC7A1,STAM2,STAT1,TCIRG1,TFRC,TGFBR2,TNFRSF1B,TPP2,WWP2	44
**Hematological System Development and Function,Tissue Morphology**	Quantity of blood cells	1E-09		0.226	Abcb1b,ABCG1,ADAM8,AP3B1,C5AR1,CD36,CD47,CTSD,DKC1,DNMT1,DOCK2,EEF1D,FCER1G,FERMT3,FLNA,FYN,GBA,GNA13,GNAI2,GNAS,HLA-A,ICAM1,IL6ST,Irgm1,ITGAM,ITGB2,JAK1,KIDINS220,LIG1,LILRB4,LYN,MAPK3,MYO1G,NOS2,NOTCH2,NPC1,PLAU,PPP2CA,PREP,PTGS2,PTPRC,PTPRJ,RAP1B,RBPJ,RIPK3,RPS6,SIPA1,SIRPA,SLC20A1,SLC2A1,SLC7A1,SPRED1,STAM2,STAT1,STEAP3,TCIRG1,TFRC,TGFBR2,TNFRSF1B,TNIP1,TPP2,TSTA3,VASP,YBX1	64
**Cellular Development,Cellular Growth and Proliferation,Hematological System Development and Function,Lymphoid Tissue Structure and Development**	Proliferation of immune cells	1.3E-09		1.171	ABCG1,ACLY,AHNAK,C5AR1,CD36,CD47,CTPS1,CTSD,DNM2,DOCK2,FCER1G,FYN,GNAI2,GPNMB,HNRNPL,ICAM1,IL6ST,Irgm1,ITCH,ITGAM,ITGB2,JAK1,KCNN4,LILRB4,LYN,MAPK3,MSN,MTA2,MVP,NCSTN,NOS2,NOTCH2,NPC1,PLAU,PPAT,PTGS2,PTPRC,PTPRJ,RIPK3,SLC29A1,SLC3A2,SLC7A1,STAM2,STAT1,TALDO1,TCIRG1,TFRC,TGFBR2,TLR7,TNFRSF1B,TPP2,WWP2	52
**Cancer,Hematological Disease,Immunological Disease,Organismal Injury and Abnormalities**	Neoplasia of leukocytes	1.3E-09	Decreased	-2.183	ABCG1,ADSL,AHNAK,ALDH9A1,ATIC,CA2,CCT3,CCT7,CD36,CD47,CDK1,CFL1,CMIP,CNOT1,CPD,CSE1L,CTSK,DDX3X,DIS3,DNM1L,DNMT1,DOCK2,EIF2A,FASN,FLNA,FYN,G3BP1,GART,GNA13,GNAI2,GRB2,HIST1H1C,HLA-A,HNRNPM,HNRNPU,HSP90AA1,HSP90AB1,ICAM1,IDH1,IL6ST,IPO5,IPO7,ITGAM,ITGB2,JAK1,KPNA2,KPNB1,KRT1,KRT10,KRT2,LYN,MAPK3,MARS,MPEG1,MYO1G,MYOF,NOS2,NOTCH2,NRAS,OASL,PCNA,PLXNB2,POLD1,PPAT,PPP2CA,PREP,PRIM2,PRPF19,PTGS2,PTPRC,RALA,RAN,RNF213,RPL13,RPS6,RPSA,SARS,SF3B1,SHMT1,SHMT2,SLC2A1,SPRED1,STAT1,STEAP3,TAX1BP1,TFRC,TGFBR2,TLR7,TNIP1,TRIM25,UBA2,USP8,VIM,XPO1	94
**Cellular Growth and Proliferation,Lymphoid Tissue Structure and Development**	Proliferation of lymphatic system cells	1.3E-09		0.885	ABCG1,ACLY,ACTL6A,AHNAK,C5AR1,CD36,CD47,CTPS1,CTSD,DNM2,DOCK2,FCER1G,FYN,GNAI2,GPNMB,HNRNPL,HSP90AB1,ICAM1,IL6ST,Irgm1,ITCH,ITGAM,ITGB2,KCNN4,LILRB4,LYN,MAPK3,MSN,MTA2,MVP,NCSTN,NOS2,NOTCH2,NPC1,NRAS,PLAU,PPAT,PTGS2,PTPRC,PTPRJ,RIPK3,SLC3A2,SLC7A1,SPRED1,STAM2,STAT1,TCIRG1,TFRC,TGFBR2,TLR7,TNFRSF1B,TPP2,WWP2	53

Table S4

**Cellular Development,Cellular Growth and Proliferation,Lymphoid Tissue Structure and Development**	Proliferation of lymphoid cells	1.4E-09		1.062	ABCG1,ACLY,AHNAK,C5AR1,CD36,CD47,CTPS1,CTSD,DNM2,DOCK2,FCER1G,FYN,GNAI2,GPNMB,HNRNPL,ICAM1,IL6ST,Irgm1,ITCH,ITGAM,ITGB2,KCNN4,LILRB4,LYN,MAPK3,MSN,MTA2,MVP,NCSTN,NOS2,NOTCH2,NPC1,PLAU,PPAT,PTGS2,PTPRC,PTPRJ,RIPK3,SLC3A2,SLC7A1,SPRED1,STAM2,STAT1,TCIRG1,TFRC,TGFBR2,TLR7,TNFRSF1B,TPP2,WWP2	50
**Cancer,Organismal Injury and Abnormalities**	Incidence of tumor	1.5E-09		-1.123	ABCA3,ABCE1,ACLY,ACSL4,ACTA1,ACTR2,ACTR3,ADAM8,ADSL,ADSSL1,AHNAK,AKR1B1,AKR1B10,ALCAM,AP3B1,APEX1,ARAP1,ARPC2,ATIC,ATP1A1,ATP2B1,ATP2C1,ATP7A,CA2,CAND1,CCT2,CCT3,CCT5,CCT6A,CCT7,CCT8,CD36,CD47,CDK1,CLUH,CMAS,CMIP,CNDP2,CNOT1,COLEC12,COPB1,CORO1C,CORO7/CORO7-PAM16,CPD,CPNE8,CRYZ,CSE1L,CTPS1,DCTN1,DDX17,DDX21,DDX39B,DDX3X,DENND4B,DHX15,DHX29,DIS3,DKC1,DNAJA1,DNAJC13,DNM1L,DNMT1,DOCK2,ECPAS,EDIL3,EEF1D,EHD1,EIF2S1,EIF2S3,EIF3A,EIF3C,EIF3D,EIF3E,EIF3I,EIF3L,ENO1,ESYT1,FASN,FLNA,FLOT1,FYN,G3BP1,G6PD,GALK1,GART,GFPT1,GMDS,GMPS,GNA13,GNAI2,GNAS,GNB2,GPNMB,GRB2,GUSB,HIST1H1C,HIST1H2AJ,HK3,HLA-A,HNRNPL,HNRNPM,HNRNPU,HSD17B4,HSP90AA1,HSP90AB1,HSPA4,IDE,IDH1,Ifi202b,IFIT1B,IFITM3,IL6ST,IPO5,ITCH,ITGAM,ITGB2,JAK1,KCNN4,KIDINS220,KPNA2,KPNB1,KRT1,KRT10,KRT2,KRT9,LGALS3BP,LIG1,LILRB4,LRP12,LYN,MAPK3,MARS,MAT2B,MCM2,MCM7,MDH1,MEMO1,MFGE8,MRI1,MSN,MTA2,MYH9,MYO1C,MYO1E,MYOF,NAA15,NANS,NCBP1,NCF2,NCL,NCSTN,NDRG1,NOP56,NOS2,NOTCH2,NPC1,NRAS,NUP93,OASL,OLA1,PCNA,PDXK,PFAS,PGK1,PHGDH,PKM,PLA2G4A,PLAU,PLD3,PLEC,PLXNA1,PLXNB2,PNKP,POLD1,POLR1C,POLR2A,PPP1CA,PPP1R7,PPP2CA,PPP2R1A,PREP,PRIM2,PRPF19,PTGS2,PTPN23,PTPRA,PTPRC,PTPRJ,RAB5B,RALA,RBBP7,RBPJ,RCC2,RHBDF2,RIPK3,RNF149,RNF213,RNH1,RNMT,RNPEP,RPF2,RPL14,RPL28,RPS6,RRP9,RTCB,SARS,SCFD1,SEC24B,SF3B1,SFPQ,SIPA1,SIRPA,SLC15A3,SLC16A3,SLC1A5,SLC20A1,SLC23A2,SLC29A1,SLC2A1,SLC38A2,SLC3A2,SLC7A1,SLCO4A1,SLFN13,SMPDL3B,SND1,SNRNP200,SNX2,SNX27,SPRED1,SQSTM1,SRM,STAT1,STOM,STRAP,STUB1,SUPT5H,TALDO1,TAX1BP1,TCIRG1,TCP1,TFRC,TGFBR2,TKT,TLR7,TMEM59,TNFRSF1B,TNIP1,TOM1,TPI1,TPP2,TRIM25,TRIM28,TRPV2,TSR1,TTC37,TTLL12,UBA1,UBA2,USP5,USP8,VAMP8,VIM,VPS13C,WARS,WDR82,WWP2,XPO1,XPO7	270
**Cancer,Gastrointestinal Disease,Organismal Injury and Abnormalities**	Gastrointestinal carcinoma	1.6E-09			ABCA3,ABCE1,ABCG1,ACAA1,ACLY,ACSL4,ACTA1,ACTR1A,ADSL,ADSSL1,AHCY,AHNAK,AKR1B1,AKR1B10,ALCAM,ALDH9A1,AMDHD2,AP3B1,APEX1,ARAP1,ATIC,ATP1A1,ATP1B3,ATP2B1,ATP2C1,ATP6V0A1,ATP7A,C5AR1,CA2,CAND1,CCT2,CCT4,CCT7,CCT8,CDK1,CFL1,CLUH,CMAS,CMIP,CNDP2,CNOT1,COLEC12,COPB1,CORO1B,CORO1C,CORO7/CORO7-PAM16,CPD,CPNE8,CSE1L,CSNK1G3,CTPS1,CTSD,CTSK,DAAM1,DCTN1,DDX17,DDX21,DDX39B,DDX3X,DENND4B,DHX15,DHX29,DIS3,DKC1,DNAJA1,DNAJC13,DNM2,DNMT1,DOCK2,DPYSL2,ECPAS,EDIL3,EEF1D,EEF1G,EHD1,EIF2S3,EIF3A,EIF3B,EIF3C,EIF3D,EIF3E,EIF3F,EIF3I,EIF3L,EIF3M,ESYT1,FARSA,FARSB,FASN,FERMT3,FKBP4,FLNA,FLOT1,FLOT2,FYN,G3BP1,G6PD,GALK1,GARS,GART,GFPT1,GMDS,GMPS,GNA13,GNAI2,GNAS,GNB2,GPNMB,GRB2,GUSB,HIST1H1C,HK3,HLA-A,HNRNPL,HNRNPM,HSD17B4,HSP90AA1,HSP90AB1,HSPA4,ICAM1,IDE,IDH1,IFIT1B,IL6ST,IPO5,IPO7,ITCH,ITGAM,ITGB2,JAK1,KCNN4,KIDINS220,KPNA2,KPNB1,KRT1,KRT2,LGALS3BP,LIG1,LILRB4,LRP12,LYN,MAPK3,MARS,MCM2,MCM7,MFGE8,MOV10,MRI1,MSN,MTA2,MVP,MYH9,MYO1C,MYO1E,MYO1G,MYOF,NAA15,NANS,NCBP1,NCF2,NCL,NCSTN,NOP56,NOS2,NOTCH2,NPC1,NRAS,NUP93,OLA1,PDS5A,PFAS,PGK1,PHGDH,PI4K2A,PKM,PLA2G4A,PLAU,PLEC,PLXNA1,PLXNB2,PNKP,POLD1,POLR1C,POLR2B,PPP1CA,PPP2R1A,PPP2R2A,PREP,PRIM2,PTGS2,PTPN23,PTPRA,PTPRC,PTPRJ,RAB5A,RAB5B,RAB8B,RALA,RAN,RANGAP1,RAP1B,RAP2C,RARS,RBBP7,RBPJ,RCC2,RHBDF2,RIPK3,RNF213,RNH1,RNMT,RPL10,RPL18,RPL18A,RPL28,RPL3,RPL4,RPL8,RPN1,RPS14,RPS27A,RPS6,RRP9,RTCB,SARS,SCAMP2,SCFD1,SEC24B,SF3B1,SFPQ,SHMT1,SHMT2,SIPA1,SIRPA,SLC12A4,SLC15A3,SLC16A3,SLC16A6,SLC1A5,SLC20A1,SLC23A2,SLC2A1,SLC38A2,SLC3A2,SLC4A7,SLC7A1,SLCO4A1,SLFN13,SMPDL3B,SMU1,SNAP23,SND1,SNRNP200,SNX2,SNX27,SPRED1,SQSTM1,SRM,STAT1,STEAP3,STOM,STRAP,STUB1,STX6,SUPT5H,TALDO1,TAX1BP1,TCIRG1,TCP1,TFRC,TGFBR2,TKT,TLR7,TMEM59,TNIP1,TOM1,TPI1,TPP2,TRIM14,TRIM28,TRPV2,TSG101,TTC37,TTLL12,UAP1L1,UBA1,UBA2,UCHL5,USP5,USP8,VASP,VIM,VPS13C,VWA5A,WARS,WDR82,WWP2,XPNPEP1,XPO1,XPO7,YBX1,ZC3HAV1	302
**Protein Degradation,Protein Synthesis**	Stabilization of protein	1.7E-09			ATP1B3,CCT2,CCT3,CCT4,CCT5,CCT6A,CCT7,CCT8,FLNA,FLOT1,FLOT2,GOLGA7,HSP90AA1,HSP90AB1,IRGM,JAK1,NAA15,PFN1,RNF149,STUB1,TCP1	21

Table S4

**Cancer,Gastrointestinal Disease,Organismal Injury and Abnormalities**	Gastrointestinal tract cancer	1.7E-09		-1.195	ABCA3,ABCE1,ABCG1,ACAA1,ACLY,ACSL4,ACTA1,ACTR1A,ADSL,ADSSL1,AHCY,AHNAK,AKR1B1,AKR1B10,ALCAM,ALDH9A1,AMDHD2,AP3B1,APEX1,ARAP1,ATIC,ATP1A1,ATP1B3,ATP2B1,ATP2C1,ATP6V0A1,ATP7A,C5AR1,CA2,CAND1,CCT2,CCT4,CCT5,CCT7,CCT8,CD36,CDK1,CFL1,CLUH,CMAS,CMIP,CNDP2,CNOT1,COLEC12,COPB1,CORO1B,CORO1C,CORO7/CORO7-PAM16,CPD,CPNE8,CRYZ,CSE1L,CSNK1G3,CTPS1,CTSD,CTSK,DAAM1,DCTN1,DDX17,DDX21,DDX39B,DDX3X,DENND4B,DHX15,DHX29,DIS3,DKC1,DNAJA1,DNAJC13,DNM2,DNMT1,DOCK2,DPYSL2,ECPAS,EDIL3,EEF1D,EEF1G,EHD1,EIF2S3,EIF3A,EIF3B,EIF3C,EIF3D,EIF3E,EIF3F,EIF3I,EIF3L,EIF3M,ENO1,ESYT1,FARSA,FARSB,FASN,FERMT3,FKBP4,FLNA,FLOT1,FLOT2,FYN,G3BP1,G6PD,GALK1,GARS,GART,GFPT1,GMDS,GMPS,GNA13,GNAI2,GNAS,GNB2,GPNMB,GRB2,GUSB,HIST1H1C,HK3,HLA-A,HNRNPL,HNRNPM,HSD17B4,HSP90AA1,HSP90AB1,HSPA4,ICAM1,IDE,IDH1,IFIT1B,IFITM3,IL6ST,IPO5,IPO7,ITCH,ITGAM,ITGB2,JAK1,KCNN4,KIDINS220,KPNA2,KPNB1,KRT1,KRT2,LGALS3BP,LIG1,LILRB4,LRP12,LYN,MAPK3,MARS,MAT2A,MCM2,MCM7,MFGE8,MOV10,MRI1,MSN,MTA2,MVP,MYH9,MYO1C,MYO1E,MYO1G,MYOF,NAA15,NANS,NCBP1,NCF2,NCL,NCSTN,NDRG1,NOP56,NOS2,NOTCH2,NPC1,NRAS,NUP93,OLA1,PCNA,PDS5A,PFAS,PGK1,PHGDH,PI4K2A,PKM,PLA2G4A,PLAU,PLEC,PLXNA1,PLXNB2,PNKP,POLD1,POLR1C,POLR2B,PPP1CA,PPP2R1A,PPP2R2A,PREP,PRIM2,PTGS2,PTPN23,PTPRA,PTPRC,PTPRJ,RAB31,RAB5A,RAB5B,RAB8B,RALA,RAN,RANGAP1,RAP1B,RAP2C,RARS,RBBP7,RBPJ,RCC2,RHBDF2,RIPK3,RNF213,RNH1,RNMT,RPL10,RPL18,RPL18A,RPL28,RPL3,RPL4,RPL8,RPN1,RPS14,RPS27A,RPS6,RRP9,RTCB,SARS,SCAMP2,SCFD1,SEC24B,SF3B1,SFPQ,SHMT1,SHMT2,SIPA1,SIRPA,SLC12A4,SLC15A3,SLC16A3,SLC16A6,SLC1A5,SLC20A1,SLC23A2,SLC2A1,SLC38A2,SLC3A2,SLC4A7,SLC7A1,SLCO4A1,SLFN13,SMPDL3B,SMU1,SNAP23,SND1,SNRNP200,SNX2,SNX27,SPRED1,SQSTM1,SRM,STAT1,STEAP3,STOM,STRAP,STUB1,STX6,SUPT5H,TALDO1,TAX1BP1,TCIRG1,TCP1,TFRC,TGFBR2,TKT,TLR7,TMEM59,TNIP1,TOM1,TPI1,TPP2,TRIM14,TRIM28,TRPV2,TSG101,TTC37,TTLL12,UAP1L1,UBA1,UBA2,UCHL5,USP5,USP8,VASP,VIM,VPS13C,VWA5A,WARS,WDR82,WWP2,XPNPEP1,XPO1,XPO7,YBX1,ZC3HAV1	311
**Lymphoid Tissue Structure and Development,Tissue Morphology**	Quantity of lymphatic system cells	1.7E-09		0.933	Abcb1b,ABCG1,ALCAM,AP3B1,C5AR1,CD36,CD47,CTSD,DKC1,DNMT1,DOCK2,EEF1D,FCER1G,FLNA,FYN,GBA,GNA13,GNAI2,HLA-A,ICAM1,IL6ST,ITGAM,ITGB2,JAK1,KIDINS220,LIG1,LILRB4,LYN,MAPK3,MYO1G,NOS2,NOTCH2,NPC1,PLAU,PTPRC,PTPRJ,RAP1B,RBPJ,RIPK3,SIPA1,SIRPA,SLC20A1,SLC2A1,SPRED1,STAM2,STAT1,TCIRG1,TGFBR2,TNFRSF1B,TNIP1,TPP2,VASP	52
**Cellular Development,Cellular Growth and Proliferation,Hematological System Development and Function,Lymphoid Tissue Structure and Development**	Proliferation of mononuclear leukocytes	2.2E-09		1.05	ABCG1,ACLY,AHNAK,C5AR1,CD36,CD47,CTPS1,CTSD,DNM2,DOCK2,FCER1G,FYN,GNAI2,GPNMB,HNRNPL,ICAM1,IL6ST,Irgm1,ITCH,ITGAM,ITGB2,KCNN4,LILRB4,LYN,MAPK3,MSN,MTA2,MVP,NCSTN,NOS2,NOTCH2,NPC1,PLAU,PPAT,PTGS2,PTPRC,PTPRJ,RIPK3,SLC3A2,SLC7A1,STAM2,STAT1,TALDO1,TCIRG1,TFRC,TGFBR2,TLR7,TNFRSF1B,TPP2,WWP2	50
**Cancer,Organismal Injury and Abnormalities**	Advanced malignant tumor	2.2E-09		0.793	ALCAM,ATIC,C5AR1,CD36,CD47,COLEC12,CRYZ,CSE1L,DNMT1,DPYSL2,FASN,FERMT3,FLNA,FYN,GART,GNAS,GPNMB,HIST1H1C,HNRNPM,HSP90AA1,HSP90AB1,ICAM1,IDH1,IFITM3,JAK1,KIDINS220,LYN,MYO1C,NDRG1,NOS2,NRAS,NUP93,PCNA,PLAU,PLEC,POLD1,PPAT,PPP2R1A,PRIM2,PTGS2,PTPRA,PTPRC,PTPRJ,RAB31,RACK1,RNH1,RPL3,RPL7,RPS27A,RPS6,SDCBP,SF3B1,SLC16A3,SQSTM1,STAT1,STEAP3,TGFBR2,TLR7,VIM,XPO1	60
**Cell-To-Cell Signaling and Interaction, Hematological System Development and Function**	Binding of leukocytes	2.3E-09	Increased	2.55	ALCAM,CD36,CD47,FERMT3,FLOT1,FYN,GNAI2,ICAM1,IL6ST,Irgm1,ITGAM,ITGB2,JAK1,LYN,MSN,MYADM,MYO1G,NOS2,NOTCH2,PLAU,PTGS2,PTPRC,RAP1B,SIPA1,SIRPA,STAT1,TFRC,TGFBR2,TLR7,TNIP1,TRPV2,VASP	32

Table S4

**Molecular Transport,Protein Trafficking**	Transport of protein	2.6E-09	Decreased	-2.137	ABCG1,AP3B1,CFL1,DCTN1,DNAJA1,DNM2,EHD1,FLNA,IPO5,IPO7,KPNA2,KPNB1,MYH9,PTGS2,RAB7A,RAN,RANGAP1,SCAMP2,SCFD1,SNAP23,SNX27,SQSTM1,TRIM28,TSG101,WWP2,XPO1,XPO7	27
**Gastrointestinal Disease,Hepatic System Disease,Organismal Injury and Abnormalities**	Liver lesion	2.8E-09		-0.692	ABCA3,ABCG1,ACAA1,ACLY,ACO2,ACTA1,ACTR1A,ACTR2,ACTR3,AHNAK,AKR1B10,ALCAM,ALDH9A1,APEX1,ARAP1,ATIC,ATP1A1,ATP1B3,ATP6V0A1,ATP7A,CA2,CCT6A,CCT8,CD36,CD47,CNDP2,CNOT1,COPB1,COPG1,CPNE8,CSE1L,CTPS1,CTSD,CTSK,DAAM1,DCTN1,DDX17,DDX39B,DDX3X,DIS3,DKC1,DNAJA1,DNAJC13,DNM1L,DOCK2,EDIL3,EIF3A,EIF3E,EIF3I,ENO1,FARSA,FASN,FERMT3,FLNA,G6PD,GALK1,GARS,GMPS,GNA13,GNAS,GRB2,GUSB,HLA-A,HNRNPM,HNRNPU,HSP90AA1,HSP90AB1,HSPA4,ICAM1,IDE,IDH1,IFITM3,IL6ST,IPO5,IPO7,ITGAM,ITGB2,JAK1,KIDINS220,KPNB1,KRT10,KRT2,KRT9,LYN,MAPK3,MARS,MAT2A,MAT2B,MCM2,MCM7,MDH1,MSN,MSTO1,MTA2,MTHFD1,MVP,MYADM,MYO1C,MYO1E,MYOF,NAA15,NCBP1,NCL,NDRG1,NOP56,NOS2,NPC1,NRAS,OASL,OSGEP,PDS5A,PDXK,PFAS,PFN1,PHGDH,PKM,PLA2G4A,PLAU,PLD3,PLEC,PLXNA1,PLXNB2,POLD1,POLR2B,PPAT,PPP2CA,PPP2R1A,PRIM2,PRPF4,PTGS2,PTPN23,PTPRA,PTPRC,PTPRJ,RAB8B,RACK1,RALA,RAN,RAP1B,RBBP7,RBPJ,RCC2,RIPK3,RNF213,RNMT,RNPEP,RPL10,RPL17,RPL21,RPL26,RPL27A,RPL4,RPL6,RPL7,RPL7A,RPN1,RPS6,RPSA,RTCB,SARS,SCFD1,SEC24B,SF3B1,SFPQ,SLC12A4,SLC16A6,SLC1A5,SLC20A1,SLC29A1,SLC2A1,SLC38A2,SLC4A7,SLC7A1,SLCO4A1,SND1,SNRNP200,SNX2,SNX27,SQSTM1,SRM,STAM2,STAT1,STUB1,SUPT5H,TAX1BP1,TCIRG1,TGFBR2,TLR7,TNFRSF1B,TNIP1,TPI1,TPP2,TRIM25,TRIM28,TTC37,UBA2,UCHL5,VIM,VPS13C,VWA5A,WARS,XPNPEP1,YBX1,ZC3HAV1	204
**Cellular Function and Maintenance**	Internalization of cells	2.8E-09	Increased	2.603	CD36,CD47,COLEC12,DOCK2,FCER1G,FYN,GRB2,ICAM1,ITGAM,ITGB2,LYN,MFGE8,MYO1G,PFN1,PLAU,PTPRC,PTPRJ,RAB31,RALA,SIRPA,SNAP23,TRPV2,VIM	23
**Cellular Function and Maintenance**	Function of blood cells	2.9E-09			Abcb1b,AHNAK,CD36,CD47,FCER1G,FLNA,FLOT1,FYN,GNAI2,GNAI3,GPNMB,HLA-A,HSP90AA1,ICAM1,ITGAM,ITGB2,KCNN4,LILRB4,LYN,MYO1G,NOS2,PLA2G4A,PLAU,PLEC,PLXNA1,PTGS2,PTPRC,RHBDF2,RIPK3,SIRPA,SLC29A1,SLC2A1,SPRED1,STAT1,TAX1BP1,TCIRG1,TLR7,TPP2,VASP	39
**Cell-To-Cell Signaling and Interaction**	Binding of blood cells	3.1E-09	Increased	3.32	ALCAM,CD36,CD47,FCER1G,FERMT3,FLOT1,FYN,GNAI2,ICAM1,IL6ST,Irgm1,ITGAM,ITGB2,JAK1,LYN,MSN,MYADM,MYO1G,NOS2,NOTCH2,PLAU,PPP2CA,PTGS2,PTPRC,RAP1B,SIPA1,SIRPA,STAT1,TFRC,TGFBR2,TLR7,TNIP1,TRPV2,VASP	34
**Cancer,Hematological Disease,Immunological Disease,Organismal Injury and Abnormalities**	B-cell neoplasm	3.4E-09			ABCG1,ADSL,AHNAK,CA2,CCT3,CD36,CD47,CDK1,CFL1,CMIP,CNOT1,CPD,CSE1L,CTSK,DIS3,DNM1L,DNMT1,DOCK2,EIF2A,FASN,FLNA,FYN,G3BP1,GNA13,GNAI2,GRB2,HIST1H1C,HLA-A,HNRNPM,HNRNPU,HSP90AA1,HSP90AB1,ICAM1,IDH1,IL6ST,ITGB2,JAK1,KPNA2,KRT1,KRT10,KRT2,LYN,MARS,MPEG1,MYO1G,MYOF,NOS2,NOTCH2,NRAS,PCNA,POLD1,PPAT,PPP2CA,PRIM2,PRPF19,PTGS2,PTPRC,RALA,RNF213,RPS6,RPSA,SARS,SF3B1,SHMT1,SHMT2,SLC2A1,STEAP3,TFRC,TGFBR2,TLR7,TNIP1,USP8,VIM,XPO1	74
**Connective Tissue Disorders,Inflammatory Disease,Inflammatory Response,Organismal Injury and Abnormalities,Skeletal and Muscular Disorders**	Inflammation of joint	3.8E-09		0.175	ACLY,ACTA1,ACTL6A,ADAM8,AP3B1,ATIC,ATP2B1,ATP2C1,C5AR1,CA2,CD36,DCTN1,DDX39B,DNM1L,EEF1G,EIF3E,ENO1,FASN,FCER1G,GNAI2,GNB2,GUSB,HELZ2,HLA-A,ICAM1,IDE,IL6ST,ITGAM,ITGB2,JAK1,LYN,MAPK3,MYO1C,PFAS,PGK1,PLA2G4A,PLAU,POLD1,PPAT,PPP1R7,PREP,PTGS2,PTPRC,RALA,RBPJ,RNF149,RPL18A,RPSA,SEC24B,SIPA1,SLC7A1,SND1,SNRNP200,STAT1,TALDO1,TCIRG1,TFRC,TGFBR2,TLR7,TNFRSF1B,TRIM28,VIM	62

Table S4

**Cellular Development,Cellular Growth and Proliferation,Hematological System Development and Function,Lymphoid Tissue Structure and Development**	Proliferation of lymphocytes	3.9E-09		1.2	ABCG1,ACLY,AHNAK,C5AR1,CD36,CD47,CTPS1,CTSD,DNM2,DOCK2,FCER1G,FYN,GNAI2,GPNMB,HNRNPL,ICAM1,IL6ST,Irgm1,ITCH,ITGAM,ITGB2,KCNN4,LILRB4,LYN,MAPK3,MSN,MTA2,MVP,NCSTN,NOS2,NOTCH2,NPC1,PLAU,PPAT,PTGS2,PTPRC,PTPRJ,RIPK3,SLC3A2,SLC7A1,STAM2,STAT1,TCIRG1,TFRC,TGFBR2,TLR7,TNFRSF1B,TPP2,WWP2	49
**Cell-To-Cell Signaling and Interaction, Hematological System Development and Function,Immune Cell Trafficking**	Adhesion of immune cells	4.5E-09	Increased	2.392	ALCAM,CD36,CD47,FERMT3,FLOT1,FYN,GNAI2,ICAM1,IL6ST,Irgm1,ITGAM,ITGB2,JAK1,LYN,MSN,MYADM,MYO1G,NOS2,PLAU,PTGS2,PTPRC,RAP1B,SIPA1,SIRPA,STAT1,TGFBR2,TLR7,TNIP1,TRPV2,VASP	30
**Cellular Movement**	Invasion of cells	5.2E-09		1.354	AHCY,AHNAK,ALCAM,ATP6V0A1,CDK1,CSE1L,CTSK,DNM1L,DNM2,DPYSL2,EIF3E,FERMT3,FLNA,FYN,GNA13,GNAI3,GRB2,HSP90AB1,IFITM3,IPO7,ITGAM,LYN,MAPK3,MFGE8,NAA15,NDRG1,NOS2,NOTCH2,NRAS,PKM,PLAU,PTGS2,PTPN23,PTPRA,PTPRJ,RAB5A,RACK1,RALA,RNH1,SDCBP,SIPA1,SLC12A4,SLC2A1,SNAP23,SQSTM1,STAT1,TCIRG1,TGFBR2,TRPV2,VASP,VIM	51
**Protein Synthesis**	Polymerization of protein	5.6E-09		-0.412	ACACA,ADSL,AHNAK,ARPC2,CD47,CRYZ,DNM1L,EHD1,ENO1,FARSA,FARSB,FCER1G,FLOT1,Fmnl1,GFPT1,GRB2,HSP90AA1,IDE,KRT1,KRT10,MAT2A,PFN1,PKM,PPAT,RIPK3,RNF213,SHMT1,SHMT2,SNX2,SQSTM1,STOM,TRIM28,VASP	33
**Cancer,Organismal Injury and Abnormalities**	Frequency of tumor	6.3E-09		-1.183	ABCA3,ABCE1,ACLY,ACSL4,ACTA1,ACTR2,ADAM8,ADSL,ADSSL1,AHNAK,AKR1B1,AKR1B10,ALCAM,AP3B1,APEX1,ARAP1,ARPC2,ATIC,ATP1A1,ATP2B1,ATP2C1,ATP7A,CA2,CAND1,CCT2,CCT3,CCT5,CCT6A,CCT7,CCT8,CD36,CD47,CDK1,CLUH,CMAS,CMIP,CNDP2,CNOT1,COLEC12,COPB1,CORO1C,CORO7/CORO7-PAM16,CPD,CPNE8,CRYZ,CSE1L,CTPS1,DCTN1,DDX17,DDX21,DDX39B,DDX3X,DENND4B,DHX15,DHX29,DIS3,DKC1,DNAJA1,DNAJC13,DNM1L,DNMT1,DOCK2,ECPAS,EDIL3,EEF1D,EHD1,EIF2S1,EIF2S3,EIF3C,EIF3D,EIF3E,EIF3I,EIF3L,ENO1,ESYT1,FASN,FLNA,FLOT1,FYN,G3BP1,G6PD,GALK1,GART,GFPT1,GMDS,GMPS,GNA13,GNAI2,GNAS,GNB2,GPNMB,GRB2,GUSB,HIST1H1C,HK3,HLA-A,HNRNPL,HNRNPM,HNRNPU,HSD17B4,HSP90AA1,HSP90AB1,HSPA4,IDE,IDH1,Ifi202b,IFIT1B,IFITM3,IL6ST,IPO5,ITCH,ITGAM,ITGB2,JAK1,KCNN4,KIDINS220,KPNA2,KPNB1,KRT1,KRT10,KRT2,KRT9,LGALS3BP,LIG1,LILRB4,LRP12,LYN,MAPK3,MARS,MAT2B,MCM2,MCM7,MDH1,MEMO1,MFGE8,MRI1,MSN,MTA2,MYH9,MYO1C,MYO1E,MYOF,NAA15,NANS,NCBP1,NCF2,NCL,NCSTN,NOP56,NOS2,NOTCH2,NPC1,NRAS,NUP93,OASL,OLA1,PCNA,PDXK,PFAS,PGK1,PHGDH,PKM,PLA2G4A,PLAU,PLEC,PLXNA1,PLXNB2,PNKP,POLD1,POLR1C,POLR2A,PPP1CA,PPP1R7,PPP2CA,PPP2R1A,PREP,PRIM2,PRPF19,PTGS2,PTPN23,PTPRA,PTPRC,PTPRJ,RAB5B,RALA,RBBP7,RBPJ,RCC2,RHBDF2,RIPK3,RNF149,RNF213,RNH1,RNMT,RNPEP,RPF2,RPL14,RPL28,RPS6,RRP9,RTCB,SARS,SCFD1,SEC24B,SF3B1,SFPQ,SIPA1,SIRPA,SLC15A3,SLC1A5,SLC20A1,SLC23A2,SLC29A1,SLC2A1,SLC38A2,SLC3A2,SLCO4A1,SLFN13,SMPDL3B,SND1,SNRNP200,SNX2,SNX27,SPRED1,SQSTM1,SRM,STAT1,STOM,STRAP,STUB1,SUPT5H,TALDO1,TAX1BP1,TCIRG1,TCP1,TFRC,TGFBR2,TKT,TLR7,TMEM59,TNFRSF1B,TOM1,TPI1,TPP2,TRIM25,TRIM28,TRPV2,TSR1,TTC37,TTLL12,UBA1,UBA2,USP5,USP8,VIM,VPS13C,WARS,WDR82,WWP2,XPO1,XPO7	261
**Cell-To-Cell Signaling and Interaction**	Adhesion of blood cells	6.7E-09	Increased	2.884	ALCAM,CD36,CD47,FCER1G,FERMT3,FLOT1,FYN,GNAI2,ICAM1,IL6ST,Irgm1,ITGAM,ITGB2,JAK1,LYN,MSN,MYADM,MYO1G,NOS2,PLAU,PTGS2,PTPRC,RAP1B,SIPA1,SIRPA,STAT1,TGFBR2,TLR7,TNIP1,TRPV2,VASP	31

Table S4

**Hematological System Development and Function,Tissue Morphology**	Quantity of leukocytes	7.6E-09		0.059	Abcb1b,ABCG1,ADAM8,AP3B1,C5AR1,CD36,CD47,CTSD,DKC1,DNMT1,DOCK2,EEF1D,FCER1G,FERMT3,FLNA,FYN,GBA,GNA13,GNAI2,GNAS,HLA-A,ICAM1,IL6ST,ITGAM,ITGB2,JAK1,KIDINS220,LIG1,LILRB4,LYN,MAPK3,MYO1G,NOS2,NOTCH2,NPC1,PLAU,PPP2CA,PREP,PTGS2,PTPRC,PTPRJ,RAP1B,RBPJ,RIPK3,SIPA1,SIRPA,SLC2A1,SPRED1,STAM2,STAT1,TCIRG1,TGFBR2,TNFRSF1B,TNIP1,TPP2,TSTA3,YBX1	57
**Cell-mediated Immune Response,Cellular Movement,Hematological System Development and Function,Immune Cell Trafficking**	T cell migration	8.3E-09		1.7	Abcb1b,CD47,DOCK2,DPYSL2,FLOT1,FYN,GNAI2,GNAI3,HLA-A,HNRNPL,ICAM1,IL6ST,ITGB2,JAK1,KCNN4,MSN,MYH9,NOS2,PLAU,PLEC,PTGS2,PTPRA,STAT1,TGFBR2,TNIP1	25
**Cancer,Hematological Disease,Immunological Disease,Organismal Injury and Abnormalities**	Lymphocytic cancer	1E-08	Decreased	-2.183	ABCG1,ADSL,AHNAK,ALDH9A1,ATIC,CCT3,CCT7,CD36,CD47,CDK1,CFL1,CMIP,CNOT1,CPD,CSE1L,DDX3X,DNM1L,DNM2,DNMT1,DOCK2,EIF2A,FASN,FYN,G3BP1,GART,GNA13,GNAI2,GRB2,HIST1H1C,HLA-A,HNRNPM,HNRNPU,HSP90AA1,HSP90AB1,ICAM1,IDH1,IPO5,IPO7,ITGAM,ITGB2,JAK1,KPNA2,KPNB1,KRT1,KRT10,KRT2,LYN,MAPK3,MARS,MPEG1,MYO1G,MYOF,NOS2,NOTCH2,NRAS,OASL,PCNA,PLXNB2,POLD1,PPAT,PPP2CA,PRIM2,PRPF19,PTGS2,PTPRC,RALA,RAN,RNF213,RPL10,RPL13,RPSA,SARS,SF3B1,SHMT1,SHMT2,SLC2A1,SPRED1,STAT1,STEAP3,TAX1BP1,TFRC,TGFBR2,TLR7,TNIP1,TRIM25,TSR1,UBA2,USP8,XPO1	89
**Cell Morphology**	Shape change of blood cells	1.1E-08		0.552	CD36,CD47,DOCK2,FERMT3,FLNA,GNA13,ICAM1,ITGAM,ITGB2,KCNN4,LYN,MYH9,PTPRA,RAP1B,SIRPA	15
**Connective Tissue Disorders,Immunological Disease,Inflammatory Disease,Inflammatory Response,Organismal Injury and Abnormalities,Skeletal and Muscular Disorders**	Rheumatoid arthritis	1.3E-08			ACLY,ACTA1,ACTL6A,ADAM8,ATIC,ATP2B1,ATP2C1,C5AR1,CA2,CD36,DDX39B,DNM1L,EEF1G,EIF3E,ENO1,GNB2,GUSB,HELZ2,HLA-A,ICAM1,IDE,IL6ST,ITGB2,JAK1,MAPK3,MYO1C,PFAS,PGK1,POLD1,PPAT,PPP1R7,PREP,PTGS2,PTPRC,RNF149,RPL18A,RPSA,SEC24B,SLC7A1,SND1,SNRNP200,STAT1,TALDO1,TCIRG1,TFRC,TLR7,TNFRSF1B,TRIM28,VIM	49
**Cellular Movement**	Cell movement of myeloid cells	1.6E-08	Increased	2.425	ADAM8,C5AR1,CD36,CD47,DOCK2,EDIL3,FCER1G,FERMT3,FLNA,FLOT1,FYN,GBA,GNAI2,GNAI3,ICAM1,IL6ST,Irgm1,ITGAM,ITGB2,JAK1,LYN,MAPK3,NDRG1,NOS2,PFN1,PLA2G4A,PLAU,PLEC,PTGS2,PTPRC,PTPRJ,RAP1B,SIRPA,STAT1,TGFBR2,TNFRSF1B,TNIP1,TRPV2,VASP,YBX1	40
**Cellular Function and Maintenance**	Function of leukocytes	1.7E-08			Abcb1b,AHNAK,CD36,FCER1G,FLOT1,FYN,GNAI2,GNAI3,GPNMB,HLA-A,HSP90AA1,ICAM1,ITGAM,ITGB2,KCNN4,LILRB4,LYN,MYO1G,NOS2,PLA2G4A,PLAU,PLEC,PLXNA1,PTGS2,PTPRC,RHBDF2,RIPK3,SIRPA,SLC2A1,SPRED1,STAT1,TAX1BP1,TCIRG1,TLR7,TPP2	35

Table S4

**Cancer,Organismal Injury and Abnormalities**	Tumorigenesis of epithelial neoplasm	1.9E-08		-0.221	ABCA3,ABCE1,ACLY,ACSL4,ACTA1,ACTR2,ADAM8,ADSL,ADSSL1,AHNAK,AKR1B1,AKR1B10,ALCAM,AP3B1,APEX1,ARAP1,ARPC2,ATIC,ATP1A1,ATP2B1,ATP2C1,ATP7A,CA2,CAND1,CCT2,CCT3,CCT5,CCT6A,CCT7,CCT8,CD36,CD47,CDK1,CLUH,CMAS,CMIP,CNDP2,CNOT1,COLEC12,COPB1,CORO1C,CORO7/CORO7-PAM16,CPD,CPNE8,CRYZ,CSE1L,CTPS1,DCTN1,DDX17,DDX21,DDX39B,DDX3X,DENND4B,DHX15,DHX29,DIS3,DKC1,DNAJA1,DNAJC13,DNM1L,DNMT1,DOCK2,ECPAS,EDIL3,EEF1D,EHD1,EIF2S1,EIF2S3,EIF3C,EIF3D,EIF3E,EIF3I,EIF3L,ENO1,ESYT1,FASN,FLNA,FLOT1,FYN,G3BP1,G6PD,GALK1,GART,GFPT1,GMDS,GMPS,GNA13,GNAI2,GNAS,GNB2,GPNMB,GRB2,GUSB,HIST1H1C,HK3,HLA-A,HNRNPL,HNRNPM,HNRNPU,HSD17B4,HSP90AA1,HSP90AB1,HSPA4,IDE,IDH1,IFIT1B,IFITM3,IL6ST,IPO5,ITCH,ITGAM,ITGB2,JAK1,KCNN4,KIDINS220,KPNA2,KPNB1,KRT1,KRT10,KRT2,KRT9,LIG1,LILRB4,LRP12,LYN,MAPK3,MARS,MAT2B,MCM2,MCM7,MDH1,MEMO1,MFGE8,MRI1,MSN,MTA2,MYH9,MYO1C,MYO1E,MYOF,NAA15,NANS,NCBP1,NCF2,NCL,NCSTN,NOP56,NOS2,NOTCH2,NPC1,NRAS,NUP93,OASL,OLA1,PCNA,PDXK,PFAS,PGK1,PHGDH,PKM,PLA2G4A,PLAU,PLEC,PLXNA1,PLXNB2,PNKP,POLD1,POLR1C,POLR2A,PPP1CA,PPP1R7,PPP2R1A,PREP,PRIM2,PRPF19,PTGS2,PTPN23,PTPRA,PTPRC,PTPRJ,RAB5B,RALA,RBBP7,RBPJ,RCC2,RHBDF2,RIPK3,RNF149,RNF213,RNH1,RNMT,RNPEP,RPF2,RPL14,RPL28,RPS6,RRP9,RTCB,SARS,SCFD1,SEC24B,SF3B1,SFPQ,SIPA1,SIRPA,SLC15A3,SLC1A5,SLC20A1,SLC23A2,SLC29A1,SLC2A1,SLC38A2,SLC3A2,SLCO4A1,SLFN13,SMPDL3B,SND1,SNRNP200,SNX2,SNX27,SPRED1,SQSTM1,SRM,STAT1,STOM,STRAP,STUB1,SUPT5H,TALDO1,TAX1BP1,TCIRG1,TCP1,TFRC,TGFBR2,TLR7,TMEM59,TOM1,TPI1,TPP2,TRIM25,TRIM28,TRPV2,TSR1,TTC37,TTLL12,UBA1,UBA2,USP5,USP8,VIM,VPS13C,WARS,WDR82,WWP2,XPO1,XPO7	256
**Cellular Assembly and Organization,Cellular Function and Maintenance**	Organization of cytoplasm	2E-08		0.644	ACACA,ACTR2,ACTR3,AHNAK,ARAP1,ARPC2,ATP2C1,ATP5F1B,ATP7A,CD47,CDK1,CFL1,CLUH,CORO1B,CORO1C,CORO7/CORO7-PAM16,DAAM1,DCTN1,DNAJC13,DNM1L,DNM2,DOCK2,DPYSL2,FASN,FERMT3,FKBP4,FLNA,FYN,GNA13,GNAS,HSP90AA1,HSP90AB1,ICAM1,IDE,ITGAM,KIDINS220,KPNB1,KRT9,LYN,MAPK3,MSN,MSTO1,MYH9,NCF2,NDRG1,PFN1,PLAU,PLEC,PLEK,PTGS2,PTPN23,RAB31,RAB5A,RAB5B,RAB5C,RALA,RAN,RPS6,SIRPA,SNX2,SQSTM1,STX6,TLR7,USP8,VASP,VIM,VPS13C	67
**DNA Replication, Recombination, and Repair,Nucleic Acid Metabolism,Small Molecule Biochemistry**	Hydrolysis of nucleotide	2.1E-08		-0.687	ATP1A1,CCT4,CCT5,CDK1,DNM1L,GNAI2,GNAI3,GNAS,IPO5,RAB7A,RAN,RANGAP1,SNRNP200,UBA1,XPO1	15
**Cell-To-Cell Signaling and Interaction,Inflammatory Response**	Immune response of leukocytes	2.2E-08		1.806	C5AR1,CD36,CD47,DOCK2,FCER1G,FYN,GNAS,HSP90AA1,ICAM1,IL6ST,ITGAM,ITGB2,LILRB4,LYN,MFGE8,MYO1G,NOS2,PLAU,PLXNA1,PTPRC,PTPRJ,SIRPA,SLC1A5,TGFBR2,TLR7,TNFRSF1B,TRPV2	27
**Cancer,Gastrointestinal Disease,Hepatic System Disease,Organismal Injury and Abnormalities**	Liver tumor	2.3E-08		-0.245	ABCA3,ABCG1,ACAA1,ACLY,ACO2,ACTA1,ACTR1A,ACTR2,ACTR3,AHNAK,AKR1B10,ALCAM,ALDH9A1,APEX1,ARAP1,ATIC,ATP1A1,ATP1B3,ATP6V0A1,ATP7A,CA2,CCT6A,CCT8,CD36,CD47,CNDP2,CNOT1,COPB1,COPG1,CPNE8,CSE1L,CTPS1,CTSD,CTSK,DAAM1,DCTN1,DDX17,DDX39B,DDX3X,DIS3,DKC1,DNAJA1,DNAJC13,DNM1L,DOCK2,EDIL3,EIF3A,EIF3E,EIF3I,ENO1,FARSA,FASN,FERMT3,FLNA,G6PD,GALK1,GARS,GMPS,GNA13,GNAS,GRB2,GUSB,HNRNPM,HNRNPU,HSP90AA1,HSP90AB1,HSPA4,ICAM1,IDE,IDH1,IFITM3,IL6ST,IPO5,IPO7,ITGAM,JAK1,KIDINS220,KPNB1,KRT10,KRT2,KRT9,MARS,MAT2A,MAT2B,MCM2,MCM7,MDH1,MSN,MSTO1,MTA2,MTHFD1,MVP,MYADM,MYO1C,MYO1E,MYOF,NAA15,NCBP1,NCL,NDRG1,NOP56,NOS2,NPC1,NRAS,OASL,OSGEP,PDS5A,PDXK,PFAS,PFN1,PHGDH,PKM,PLA2G4A,PLAU,PLD3,PLEC,PLXNA1,PLXNB2,POLD1,POLR2B,PPP2CA,PPP2R1A,PRIM2,PRPF4,PTGS2,PTPN23,PTPRA,PTPRC,RAB8B,RACK1,RALA,RAN,RAP1B,RBBP7,RBPJ,RCC2,RNF213,RNMT,RNPEP,RPL10,RPL17,RPL21,RPL26,RPL27A,RPL4,RPL6,RPL7,RPL7A,RPN1,RPS6,RPSA,RTCB,SARS,SCFD1,SEC24B,SF3B1,SFPQ,SLC12A4,SLC16A6,SLC1A5,SLC29A1,SLC2A1,SLC38A2,SLC4A7,SLC7A1,SLCO4A1,SND1,SNRNP200,SNX2,SNX27,SQSTM1,SRM,STAM2,STAT1,SUPT5H,TAX1BP1,TCIRG1,TGFBR2,TLR7,TNFRSF1B,TNIP1,TPI1,TPP2,TRIM25,TRIM28,TTC37,UBA2,UCHL5,VIM,VPS13C,VWA5A,WARS,XPNPEP1,YBX1,ZC3HAV1	195

Table S4

**Cancer,Organismal Injury and Abnormalities**	Thoracic neoplasm	2.4E-08		0.915	ACLY,ADAM8,AHNAK,AKR1B1,AKR1B10,ALCAM,AP3B1,APEX1,ARAP1,ATIC,ATP1A1,ATP5F1B,C5AR1,CCT7,CDK1,CNDP2,CORO1C,CPD,CRYZ,CTSD,DDX17,DDX3X,DIS3,DNAJA1,DNMT1,EIF2S1,EIF3A,EIF3E,ENO1,FASN,FLNA,G3BP1,G6PD,GART,GNAS,GPNMB,HIST1H1C,HLA-A,HSD17B4,HSP90AA1,HSP90AB1,ICAM1,IDH1,IL6ST,Irgm1,JAK1,KIDINS220,KRT10,LIG1,LYN,MCM2,MFGE8,MSN,MTA2,MVP,MYH9,MYO1G,NCL,NDRG1,NOS2,NOTCH2,NRAS,NUP93,PCNA,PFAS,PKM,PLA2G4A,PLEC,POLD1,POLR2A,PPP1R7,PPP2R1A,PPP2R2A,PRIM2,PRPF19,PTGS2,PTPRA,PTPRC,PTPRJ,RACK1,RALA,RBBP7,RNF213,RNPEP,RPL7,RPS27A,RPS6,SCFD1,SF3B1,SHMT2,SLC29A1,SLCO4A1,SQSTM1,SRM,STAT1,TGFBR2,TPI1,TRIM25,TSG101,TTC37,UBA1,VIM,XPNPEP1	103
**Tissue Morphology**	Quantity of cells	2.5E-08		0.585	ABCA3,Abcb1b,ABCG1,ACACA,ADAM8,ALCAM,AP3B1,ATP7A,C5AR1,CD36,CD47,CTSD,CTSK,DDX3X,DKC1,DNMT1,DOCK2,EEF1D,EIF2S1,ENO1,FCER1G,FERMT3,FLNA,FYN,GBA,GNA13,GNAI2,GNAS,GPNMB,HLA-A,ICAM1,IL6ST,Irgm1,ITGAM,ITGB2,JAK1,KIDINS220,LIG1,LILRB4,LYN,MAPK3,MFGE8,MSN,MYH9,MYO1G,MYOF,NOS2,NOTCH2,NPC1,NRAS,PFN1,PLA2G4A,PLAU,PPP2CA,PREP,PTGS2,PTPRC,PTPRJ,RAB7A,RALA,RAP1B,RBPJ,RIPK3,RPS6,SIPA1,SIRPA,SLC20A1,SLC2A1,SLC4A7,SLC7A1,SNX27,SPRED1,SQSTM1,STAM2,STAT1,STEAP3,TCIRG1,TFRC,TGFBR2,TNFRSF1B,TNIP1,TPP2,TSTA3,VASP,VIM,YBX1	86
**Cancer,Organismal Injury and Abnormalities,Respiratory Disease**	Lung tumor	2.5E-08		1.273	ACLY,ADAM8,AHNAK,AKR1B1,AKR1B10,ALCAM,AP3B1,APEX1,ARAP1,ATIC,ATP1A1,ATP5F1B,C5AR1,CCT7,CDK1,CNDP2,CORO1C,CPD,CRYZ,CTSD,DDX17,DDX3X,DIS3,DNAJA1,DNMT1,EIF2S1,EIF3A,EIF3E,ENO1,FASN,FLNA,G3BP1,G6PD,GART,GNAS,GPNMB,HIST1H1C,HLA-A,HSD17B4,HSP90AA1,HSP90AB1,ICAM1,IDH1,IL6ST,Irgm1,JAK1,KIDINS220,KRT10,LIG1,LYN,MCM2,MFGE8,MSN,MTA2,MVP,MYH9,MYO1G,NCL,NDRG1,NOS2,NOTCH2,NRAS,NUP93,PCNA,PFAS,PKM,PLA2G4A,PLEC,POLR2A,PPP1R7,PPP2R1A,PPP2R2A,PRIM2,PRPF19,PTGS2,PTPRA,PTPRC,PTPRJ,RACK1,RALA,RBBP7,RNF213,RNPEP,RPL7,RPS27A,RPS6,SCFD1,SF3B1,SHMT2,SLC29A1,SLCO4A1,SQSTM1,SRM,STAT1,TGFBR2,TPI1,TRIM25,TSG101,TTC37,UBA1,VIM,XPNPEP1	102
**Cancer,Gastrointestinal Disease,Hepatic System Disease,Organismal Injury and Abnormalities**	Hepatobiliary system cancer	2.6E-08		-0.133	ABCA3,ABCG1,ACAA1,ACLY,ACO2,ACTR1A,ACTR2,ACTR3,AHNAK,AKR1B10,ALCAM,ALDH9A1,APEX1,ARAP1,ATIC,ATP1A1,ATP1B3,ATP6V0A1,ATP7A,CA2,CAND1,CCT6A,CCT8,CD36,CD47,CNDP2,CNOT1,COPB1,COPG1,CPNE8,CSE1L,CTPS1,CTSD,DAAM1,DDX17,DDX39B,DDX3X,DIS3,DKC1,DNAJA1,DNAJC13,DNM1L,DOCK2,EDIL3,EHD1,EIF3A,EIF3E,EIF3I,ENO1,FARSA,FASN,FERMT3,FLNA,G6PD,GALK1,GARS,GMPS,GNA13,GNAS,GRB2,GUSB,HNRNPM,HSP90AA1,HSP90AB1,HSPA4,ICAM1,IDE,IDH1,IFITM3,IL6ST,IPO5,IPO7,ITGAM,JAK1,KIDINS220,KPNA2,KPNB1,KRT10,KRT9,MARS,MAT2A,MAT2B,MCM2,MCM7,MDH1,MSTO1,MTA2,MTHFD1,MVP,MYADM,MYH9,MYO1C,MYO1E,MYOF,NAA15,NCBP1,NCL,NDRG1,NOP56,NOS2,NPC1,NRAS,OASL,PDS5A,PFAS,PFN1,PGK1,PHGDH,PKM,PLA2G4A,PLAU,PLD3,PLEC,PLXNA1,PLXNB2,POLD1,POLR2B,PPP2CA,PPP2R1A,PRIM2,PRPF4,PTGS2,PTPRA,PTPRC,RAB8B,RACK1,RALA,RAN,RAP1B,RBPJ,RNF213,RNMT,RNPEP,RPL27A,RPL4,RPL6,RPL7A,RPN1,RPSA,RTCB,SARS,SCFD1,SEC24B,SF3B1,SFPQ,SLC16A6,SLC1A5,SLC20A1,SLC29A1,SLC2A1,SLC38A2,SLC4A7,SLC7A1,SND1,SNRNP200,SNX2,SNX27,SQSTM1,SRM,STAM2,STAT1,SUPT5H,TAX1BP1,TCIRG1,TGFBR2,TLR7,TNFRSF1B,TNIP1,TPI1,TPP2,TRIM25,TRIM28,TTC37,UBA2,UCHL5,VIM,VPS13C,WARS,XPNPEP1,XPO1	180
**Cellular Function and Maintenance**	Cellular homeostasis	2.9E-08	Increased	2.163	ABCG1,ACLY,AKR1B1,AP3B1,ATP1A1,ATP1B3,ATP2C1,ATP7A,C5AR1,CA2,CAND1,CD36,CD47,CTSD,DCTN1,DNM1L,DNMT1,DOCK2,EEF1D,EIF2S1,FCER1G,FYN,GNA13,GNAI3,GNAS,GRB2,HELZ2,HLA-A,HNRNPL,HSP90AA1,ICAM1,IL6ST,IRGM,Irgm1,ITCH,ITGB2,JAK1,KCNN4,KIDINS220,LILRB4,LYN,MAPK3,MTHFD1,MYADM,NCSTN,NOS2,NOTCH2,NPC1,PLA2G4A,PLAU,PTGS2,PTPRC,PTPRJ,RAB7A,RAP1B,RBPJ,RIPK3,RPS6,SLC12A4,SLC2A1,SLC3A2,SLC4A7,SQSTM1,STAM2,STAT1,STUB1,SUPT5H,TCIRG1,TFRC,TGFBR2,TLR7,TMEM59,TNFRSF1B,TPP2,TSG101,VASP,VIM,WWP2,XPO1	79
**Cell Morphology**	Orientation of cells	3E-08		1.838	CD36,CD47,CFL1,DAAM1,DOCK2,DPYSL2,FCER1G,FYN,GNAI3,ITGB2,MSN,MYH9,NRAS,RBPJ,SEC24B,SIPA1,SIRPA,VASP	18
**Free Radical Scavenging**	Synthesis of reactive oxygen species	3.1E-08	Increased	2.6	ACOD1,AKR1B1,C5AR1,CD36,CD47,DNM1L,DNM2,DOCK2,FYN,G6PD,GNAS,HK3,HSP90AB1,ICAM1,IDH1,ITGAM,ITGB2,JAK1,LYN,NCF2,NOS2,PLA2G4A,PLAU,PTGS2,RACK1,RIPK3,RPL26,SLC2A1,SNAP23,SQSTM1,TFRC,TLR7	32
**Lymphoid Tissue Structure and Development,Tissue Morphology**	Quantity of lymphoid tissue	3.2E-08		-0.932	Abcb1b,ALCAM,C5AR1,CTSD,DOCK2,EEF1D,FCER1G,FYN,GBA,GNA13,GNAI2,IL6ST,ITGB2,JAK1,KIDINS220,LYN,MAPK3,MFGE8,NOS2,NOTCH2,PTPRC,PTPRJ,RAP1B,RIPK3,SPRED1,STAM2,STAT1,STEAP3,TCIRG1,TPP2	30
**Gene Expression, Protein Synthesis**	Initiation of translation of mRNA	3.3E-08			DDX3X,EIF2S3,EIF3B,EIF3C,EIF3D,EIF3E,EIF3F,EIF3I,EIF3L,NCBP1	10

Table S4

**Free Radical Scavenging**	Metabolism of reactive oxygen species	3.5E-08	Increased	2.638	ACOD1,AKR1B1,ATP7A,C5AR1,CD36,CD47,DNM1L,DNM2,DOCK2,FYN,G6PD,GNAS,HK3,HSP90AB1,ICAM1,IDH1,ITGAM,ITGB2,JAK1,LYN,NCF2,NOS2,PLA2G4A,PLAU,PTGS2,RACK1,RIPK3,RPL26,SLC2A1,SNAP23,SQSTM1,TFRC,TLR7	33
**Cellular Movement**	Cellular infiltration	3.7E-08		1.492	ABCG1,ACO2,ADAM8,AKR1B1,C5AR1,CD36,CD47,EDIL3,ENO1,FCER1G,GBA,HLA-A,ICAM1,IL6ST,ITGAM,ITGB2,KRT10,MAPK3,MYH9,NDRG1,NOS2,NPC1,PLA2G4A,PLAU,PLEC,PTGS2,STAT1,TCIRG1,TGFBR2,TKT,TNFRSF1B,TNIP1,TPI1,YBX1	34
**Cancer,Hematological Disease,Immunological Disease,Organismal Injury and Abnormalities**	B cell cancer	5.4E-08			ABCG1,ADSL,AHNAK,CCT3,CD36,CDK1,CMIP,CNOT1,CSE1L,DNM1L,DNMT1,DOCK2,EIF2A,FASN,FYN,G3BP1,GNA13,GNAI2,GRB2,HIST1H1C,HNRNPM,HNRNPU,HSP90AA1,HSP90AB1,ICAM1,IDH1,ITGB2,JAK1,KPNA2,KRT2,LYN,MPEG1,MYO1G,MYOF,NOS2,NOTCH2,NRAS,PCNA,POLD1,PPAT,PPP2CA,PRIM2,PRPF19,PTGS2,PTPRC,RALA,RNF213,RPSA,SARS,SF3B1,SHMT1,SHMT2,SLC2A1,STEAP3,TFRC,TGFBR2,TLR7,TNIP1,USP8,XPO1	60
**Hematological System Development and Function,Lymphoid Tissue Structure and Development,Tissue Morphology**	Quantity of lymphocytes	5.7E-08		0.506	Abcb1b,ABCG1,AP3B1,C5AR1,CD36,CD47,CTSD,DKC1,DNMT1,DOCK2,EEF1D,FCER1G,FYN,GNA13,GNAI2,HLA-A,ICAM1,IL6ST,ITGAM,ITGB2,JAK1,KIDINS220,LIG1,LILRB4,LYN,MAPK3,MYO1G,NOS2,NOTCH2,NPC1,PLAU,PTPRC,PTPRJ,RAP1B,RBPJ,RIPK3,SIPA1,SIRPA,SLC2A1,STAM2,STAT1,TCIRG1,TGFBR2,TNFRSF1B,TNIP1,TPP2	46
**Cellular Movement,Hematological System Development and Function,Immune Cell Trafficking,Inflammatory Response**	Cell movement of phagocytes	6E-08	Increased	2.379	ADAM8,ALCAM,C5AR1,CD36,CD47,DOCK2,EDIL3,FCER1G,FERMT3,FLNA,FLOT1,FYN,GBA,GNAI2,GNAI3,ICAM1,IL6ST,Irgm1,ITGAM,ITGB2,JAK1,LYN,MAPK3,NDRG1,NOS2,PFN1,PLA2G4A,PLAU,PLEC,PTGS2,PTPRJ,RAP1B,SIRPA,STAT1,TGFBR2,TNFRSF1B,TNIP1,TRPV2,YBX1	39
**Cancer,Hematological Disease,Immunological Disease,Organismal Injury and Abnormalities**	B-cell lymphoma	7.3E-08			ABCG1,ADSL,AHNAK,CCT3,CD36,CDK1,CMIP,CNOT1,CSE1L,DNMT1,DOCK2,EIF2A,FASN,FYN,G3BP1,GNA13,GNAI2,GRB2,HIST1H1C,HNRNPM,HNRNPU,HSP90AA1,HSP90AB1,ICAM1,IDH1,JAK1,KPNA2,KRT2,MYO1G,MYOF,NOS2,NOTCH2,NRAS,PCNA,POLD1,PPAT,PPP2CA,PRIM2,PTGS2,PTPRC,RALA,RNF213,RPSA,SARS,SF3B1,SHMT1,SHMT2,STEAP3,TLR7,TNIP1,USP8,XPO1	52
**Cancer,Organismal Injury and Abnormalities**	Thoracic cancer	7.3E-08		1.29	ACLY,ADAM8,AHNAK,AKR1B10,ALCAM,AP3B1,APEX1,ARAP1,ATIC,ATP1A1,ATP5F1B,C5AR1,CCT7,CDK1,CNDP2,CORO1C,CPD,CRYZ,CTSD,DDX17,DDX3X,DIS3,DNAJA1,DNMT1,EIF3A,EIF3E,ENO1,FASN,FLNA,G3BP1,G6PD,GART,GNAS,GPNMB,HIST1H1C,HLA-A,HSD17B4,HSP90AA1,HSP90AB1,ICAM1,IDH1,IL6ST,JAK1,KIDINS220,KRT10,LIG1,LYN,MCM2,MFGE8,MSN,MTA2,MVP,MYH9,MYO1G,NCL,NOS2,NOTCH2,NRAS,NUP93,PCNA,PFAS,PKM,PLA2G4A,PLEC,POLD1,POLR2A,PPP1R7,PPP2R1A,PPP2R2A,PRIM2,PRPF19,PTGS2,PTPRA,PTPRC,PTPRJ,RACK1,RALA,RBBP7,RNF213,RNPEP,RPL7,RPS27A,RPS6,SCFD1,SF3B1,SHMT2,SLC29A1,SLCO4A1,SQSTM1,SRM,STAT1,TGFBR2,TPI1,TRIM25,TSG101,TTC37,UBA1,VIM,XPNPEP1	99

Table S4

**Cancer,Organismal Injury and Abnormalities**	Pelvic cancer	7.4E-08			ABCA3,ABCE1,ACLY,ACSL4,ACTR1A,ACTR2,ACTR3,ADSL,ADSSL1,AHNAK,AKR1B1,ALCAM,ALDH9A1,AP3B1,APEX1,ATIC,ATP1A1,ATP2B1,ATP2C1,ATP6V0A1,ATP7A,CA2,CAND1,CAPZA1,CCT2,CCT5,CCT6A,CD36,CD47,CDK1,CLUH,CMIP,CNOT1,COLEC12,COPB1,CORO1C,CPD,CPNE8,CSE1L,CTPS1,CTSD,CTSK,DCTN1,DDX17,DDX21,DDX3X,DENND4B,DHX29,DIS3,DKC1,DNAJA1,DNAJC13,DNM1L,DNMT1,DOCK2,ECPAS,EDIL3,EEF1D,EEF1G,EIF2S1,EIF2S3,EIF3A,EIF3D,EIF3E,EIF3F,EIF3I,ENO1,FASN,FERMT3,FKBP4,FLNA,FLOT1,FYN,G3BP1,GART,GFPT1,GMDS,GNA13,GNAI2,GNAS,GNB2,GPNMB,GRB2,GUSB,HIST1H1C,HIST1H2AJ,HK3,HLA-A,HNRNPL,HNRNPM,HNRNPU,HSD17B4,HSP90AA1,HSP90AB1,HSPA4,ICAM1,IDH1,IPO5,ITCH,ITGAM,ITGB2,JAK1,KIDINS220,KPNA2,KRT1,KRT10,KRT9,LIG1,LILRB4,LRP12,MAT2B,MCM2,MCM7,MDH1,MEMO1,MFGE8,MSN,MSTO1,MVP,MYADM,MYH9,MYO1C,MYO1E,MYO1G,MYOF,NAA15,NCBP1,NCSTN,NOP56,NOS2,NOTCH2,NPC1,NRAS,NUP93,OASL,OLA1,PCNA,PDS5A,PDXK,PFAS,PGK1,PI4K2A,PKM,PLAU,PLD3,PLEC,PLEK,PLXNA1,PLXNB2,PNKP,POLD1,POLR1C,POLR2B,PPAT,PPP1R7,PPP2CA,PPP2R1A,PRIM2,PTGS2,PTPN23,PTPRA,PTPRC,PTPRJ,RALA,RANGAP1,RARS,RBBP7,RCC2,RIPK3,RNF149,RNF213,RNMT,RNPEP,RPF2,RPL14,RPL17,RPS27A,RPS6,RRP9,SCFD1,SEC24B,SF3B1,SFPQ,SHMT2,SIRPA,SLC12A4,SLC15A3,SLC16A3,SLC20A1,SLC23A2,SLC29A1,SLC2A1,SLC38A2,SLC3A2,SLC7A1,SLFN13,SMPDL3B,SND1,SNRNP200,SNX27,STAT1,STEAP3,STOM,STX6,SUPT5H,TALDO1,TAX1BP1,TFRC,TGFBR2,TKT,TLR7,TMEM59,TNIP1,TPI1,TPP2,TRIM25,TRIM28,TRPV2,TSG101,TSR1,TTC37,TTLL12,UBA1,UBA2,USP5,USP8,VAMP8,VIM,VPS13C,VWA5A,WWP2,XPO1,XPO7,YBX1	234
**Hematological System Development and Function,Tissue Morphology**	Quantity of mononuclear leukocytes	7.5E-08		0.189	Abcb1b,ABCG1,AP3B1,C5AR1,CD36,CD47,CTSD,DKC1,DNMT1,DOCK2,EEF1D,FCER1G,FYN,GNA13,GNAI2,GNAS,HLA-A,ICAM1,IL6ST,ITGAM,ITGB2,JAK1,KIDINS220,LIG1,LILRB4,LYN,MAPK3,MYO1G,NOS2,NOTCH2,NPC1,PLAU,PTPRC,PTPRJ,RAP1B,RBPJ,RIPK3,SIPA1,SIRPA,SLC2A1,STAM2,STAT1,TCIRG1,TGFBR2,TNFRSF1B,TNIP1,TPP2	47
**Cancer,Organismal Injury and Abnormalities**	Breast or colorectal cancer	7.5E-08		-1.091	ABCA3,ABCE1,ACAA1,ACLY,ACTA1,ADSL,ADSSL1,AHCY,AKR1B1,AKR1B10,ALCAM,AP3B1,APEX1,ARAP1,ATIC,ATP1A1,ATP2B1,ATP2C1,ATP7A,CA2,CCT2,CCT3,CCT4,CCT5,CCT7,CD36,CDK1,CFL1,CLUH,CMAS,CMIP,CNDP2,CNOT1,COLEC12,COPB1,CORO1C,CORO7/CORO7-PAM16,CPD,CPNE8,CRYZ,CSE1L,CTSD,CTSK,DCTN1,DDX17,DDX21,DDX39B,DENND4B,DHX15,DHX29,DIS3,DKC1,DNAJA1,DNAJC13,DNM1L,DNMT1,DOCK2,DPYSL2,EDIL3,EHD1,EIF2S3,EIF3A,EIF3B,EIF3C,EIF3E,EIF3F,EIF3I,EIF3L,EIF3M,ENO1,FASN,FLNA,FYN,G6PD,GALK1,GART,GBA,GMPS,GNA13,GNAI2,GNAI3,GNAS,GRB2,HIST1H1C,HK3,HLA-A,HNRNPL,HNRNPM,HSP90AA1,HSP90AB1,HSPA4,ICAM1,IDE,IDH1,IFITM3,IL6ST,IPO5,ITCH,ITGAM,ITGB2,JAK1,KCNN4,KIDINS220,KPNA2,KPNB1,KRT1,KRT2,LGALS3BP,LIG1,LILRB4,LRP12,LYN,MAPK3,MARS,MAT2A,MCM2,MCM7,MOV10,MRI1,MTA2,MYH9,MYO1C,MYO1E,MYOF,NANS,NCF2,NCL,NDRG1,NOTCH2,NPC1,NRAS,NUP93,NUS1,PCNA,PFAS,PFN1,PGK1,PI4K2A,PKM,PLAU,PLEC,PLXNA1,PLXNB2,POLD1,POLR2A,PPP1CA,PPP2R1A,PREP,PRIM2,PTGS2,PTPN23,PTPRA,PTPRC,PTPRJ,RAB31,RAB5B,RAB5C,RANGAP1,RAP1B,RCC2,RHBDF2,RNF213,RNH1,RNMT,RPL28,RPL4,RPL6,RPS6,RTCB,SF3B1,SFPQ,SHMT2,SIPA1,SIRPA,SLC15A3,SLC16A3,SLC20A1,SLC23A2,SLC2A1,SLC38A2,SLC3A2,SLFN13,SMPDL3B,SND1,SNRNP200,SNX2,SPRED1,SQSTM1,SRM,STAT1,STEAP3,STRAP,STUB1,SUPT5H,TAX1BP1,TCIRG1,TCP1,TFRC,TGFBR2,TLR7,TMEM59,TNFRSF1B,TPI1,TPP2,TRIM25,TRIM28,TRPV2,TSG101,UBA1,UBA2,UCHL5,VIM,VPS13C,VWA5A,WDR82,XPO1,XPO7	217
**Free Radical Scavenging**	Production of reactive oxygen species	7.8E-08	Increased	2.648	ACOD1,AKR1B1,C5AR1,CD36,DNM2,DOCK2,FYN,G6PD,GNAS,HK3,HSP90AB1,ICAM1,IDH1,ITGAM,ITGB2,JAK1,LYN,NCF2,NOS2,PLA2G4A,PTGS2,RACK1,RIPK3,SLC2A1,SNAP23,SQSTM1	26
**Cancer,Hematological Disease,Immunological Disease,Organismal Injury and Abnormalities**	Mature B-cell neoplasm	7.9E-08			ABCG1,ADSL,AHNAK,CA2,CCT3,CD36,CD47,CDK1,CFL1,CNOT1,CPD,CTSK,DIS3,DNM1L,DNMT1,DOCK2,EIF2A,FASN,FLNA,G3BP1,GNA13,GNAI2,GRB2,HIST1H1C,HLA-A,HNRNPM,HNRNPU,HSP90AA1,HSP90AB1,IDH1,IL6ST,ITGB2,JAK1,KPNA2,KRT1,KRT10,KRT2,LYN,MARS,MPEG1,MYO1G,MYOF,NOS2,NOTCH2,NRAS,PCNA,POLD1,PPP2CA,PRIM2,PRPF19,PTGS2,PTPRC,RALA,RPS6,RPSA,SARS,SF3B1,SHMT1,SHMT2,STEAP3,TFRC,TGFBR2,TLR7,TNIP1,USP8,VIM,XPO1	67

Table S4

**Cancer,Organismal Injury and Abnormalities**	Pelvic tumor	8E-08		1.698	ABCA3,ABCE1,ACLY,ACSL4,ACTR1A,ACTR2,ACTR3,ADSL,ADSSL1,AHNAK,AKR1B1,ALCAM,ALDH9A1,AP3B1,APEX1,ATIC,ATP1A1,ATP2B1,ATP2C1,ATP6V0A1,ATP7A,CA2,CAND1,CAPZA1,CCT2,CCT5,CCT6A,CD36,CD47,CDK1,CLUH,CMIP,CNOT1,COLEC12,COPB1,CORO1C,CPD,CPNE8,CSE1L,CTPS1,CTSD,CTSK,DCTN1,DDX17,DDX21,DDX3X,DENND4B,DHX29,DIS3,DKC1,DNAJA1,DNAJC13,DNM1L,DNMT1,DOCK2,ECPAS,EDIL3,EEF1D,EEF1G,EIF2S1,EIF2S3,EIF3A,EIF3D,EIF3E,EIF3F,EIF3I,ENO1,FASN,FERMT3,FKBP4,FLNA,FLOT1,FYN,G3BP1,GART,GFPT1,GMDS,GNA13,GNAI2,GNAS,GNB2,GPNMB,GRB2,GUSB,HIST1H1C,HIST1H2AJ,HK3,HLA-A,HNRNPL,HNRNPM,HNRNPU,HSD17B4,HSP90AA1,HSP90AB1,HSPA4,ICAM1,IDH1,IPO5,ITCH,ITGAM,ITGB2,JAK1,KIDINS220,KPNA2,KRT1,KRT10,KRT9,LIG1,LILRB4,LRP12,LYN,MAPK3,MAT2B,MCM2,MCM7,MDH1,MEMO1,MFGE8,MSN,MSTO1,MVP,MYADM,MYH9,MYO1C,MYO1E,MYO1G,MYOF,NAA15,NCBP1,NCSTN,NDRG1,NOP56,NOS2,NOTCH2,NPC1,NRAS,NUP93,OASL,OLA1,PCNA,PDS5A,PDXK,PFAS,PGK1,PI4K2A,PKM,PLAU,PLD3,PLEC,PLEK,PLXNA1,PLXNB2,PNKP,POLD1,POLR1C,POLR2B,PPAT,PPP1R7,PPP2CA,PPP2R1A,PRIM2,PTGS2,PTPN23,PTPRA,PTPRC,PTPRJ,RALA,RANGAP1,RARS,RBBP7,RCC2,RIPK3,RNF149,RNF213,RNMT,RNPEP,RPF2,RPL14,RPL17,RPS27A,RPS6,RRP9,SCFD1,SEC24B,SF3B1,SFPQ,SHMT2,SIRPA,SLC12A4,SLC15A3,SLC16A3,SLC20A1,SLC23A2,SLC29A1,SLC2A1,SLC38A2,SLC3A2,SLC7A1,SLFN13,SMPDL3B,SND1,SNRNP200,SNX27,STAT1,STEAP3,STOM,STX6,SUPT5H,TALDO1,TAX1BP1,TFRC,TGFBR2,TKT,TLR7,TMEM59,TNIP1,TPI1,TPP2,TRIM25,TRIM28,TRPV2,TSG101,TSR1,TTC37,TTLL12,UBA1,UBA2,USP5,USP8,VAMP8,VIM,VPS13C,VWA5A,WWP2,XPO1,XPO7,YBX1	237
**Cancer,Organismal Injury and Abnormalities,Reproductive System Disease**	Genital tumor	8.2E-08		1.067	ABCA3,ABCE1,ACLY,ACSL4,ACTR1A,ACTR2,ACTR3,ADSL,ADSSL1,AHNAK,AKR1B1,ALCAM,ALDH9A1,AP3B1,APEX1,ATIC,ATP1A1,ATP2B1,ATP2C1,ATP6V0A1,ATP7A,CA2,CAND1,CAPZA1,CCT2,CCT5,CCT6A,CD36,CD47,CDK1,CLUH,CMIP,CNOT1,COLEC12,COPB1,CORO1C,CPD,CPNE8,CSE1L,CTPS1,CTSD,CTSK,DCTN1,DDX17,DDX3X,DENND4B,DHX29,DIS3,DNAJA1,DNAJC13,DNM1L,DNMT1,DOCK2,ECPAS,EDIL3,EEF1D,EEF1G,EIF2S1,EIF2S3,EIF3A,EIF3C,EIF3D,EIF3E,EIF3F,EIF3I,ENO1,FASN,FKBP4,FLNA,FLOT1,FYN,G3BP1,GART,GFPT1,GMDS,GNA13,GNAI2,GNAS,GNB2,GPNMB,GRB2,GUSB,HIST1H1C,HIST1H2AJ,HK3,HLA-A,HNRNPL,HNRNPM,HNRNPU,HSD17B4,HSP90AA1,HSP90AB1,HSPA4,IDH1,IPO5,ITCH,ITGAM,JAK1,KIDINS220,KPNA2,KRT1,KRT10,KRT9,LIG1,LILRB4,LRP12,LYN,MAPK3,MAT2B,MCM2,MCM7,MDH1,MEMO1,MFGE8,MSN,MYADM,MYH9,MYO1C,MYO1E,MYO1G,MYOF,NAA15,NCBP1,NDRG1,NOP56,NOS2,NOTCH2,NPC1,NRAS,NUP93,OASL,OLA1,PCNA,PDS5A,PDXK,PFAS,PGK1,PKM,PLAU,PLD3,PLEC,PLEK,PLXNA1,PLXNB2,PNKP,POLD1,POLR1C,POLR2B,PPAT,PPP1R7,PPP2CA,PPP2R1A,PRIM2,PTGS2,PTPN23,PTPRA,PTPRC,PTPRJ,RALA,RANGAP1,RAP2C,RARS,RBBP7,RCC2,RIPK3,RNF149,RNF213,RNPEP,RPF2,RPL14,RPS27A,RPS6,RRP9,SCFD1,SEC24B,SF3B1,SFPQ,SHMT2,SIRPA,SLC12A4,SLC15A3,SLC16A3,SLC20A1,SLC29A1,SLC2A1,SLC38A2,SLC3A2,SLC7A1,SMPDL3B,SND1,SNRNP200,SNX27,STAT1,STEAP3,STOM,STX6,SUPT5H,TALDO1,TFRC,TGFBR2,TKT,TLR7,TMEM59,TNIP1,TPI1,TPP2,TRIM25,TRIM28,TRPV2,TSG101,TSR1,TTC37,TTLL12,UBA1,UBA2,USP5,USP8,VAMP8,VIM,VPS13C,VWA5A,WWP2,XPO1,XPO7,YBX1	225
**Inflammatory Response,Organismal Injury and Abnormalities**	Inflammation of organ	8.6E-08		-0.759	ABCA3,ABCG1,ACO2,AHCY,AHNAK,ALCAM,ATP1A1,C5AR1,CA2,CD36,CD47,CFL1,CTSD,DPYSL2,EIF3E,ENO1,FASN,FCER1G,FKBP4,FLNA,FLOT1,FYN,GBA,GNAI2,HLA-A,ICAM1,IDE,IL6ST,Irgm1,ITCH,ITGAM,ITGB2,JAK1,KCNN4,KRT1,KRT10,LYN,MAPK3,MFGE8,MSN,MTA2,MYH9,NCSTN,NOS2,NOTCH2,NPC1,PHGDH,PKM,PLA2G4A,PLAU,POLD1,PPAT,PPP2CA,PTGS2,PTPRC,PTPRJ,RBPJ,RIPK3,SF3B1,SQSTM1,STAT1,STUB1,TAX1BP1,TGFBR2,TKT,TLR7,TNFRSF1B,TNIP1,TPI1,TSTA3,YBX1	71
**Cancer,Organismal Injury and Abnormalities,Respiratory Disease**	Lung cancer	9E-08	Increased	2.088	ACLY,ADAM8,AHNAK,AKR1B10,ALCAM,AP3B1,APEX1,ARAP1,ATIC,ATP1A1,ATP5F1B,C5AR1,CCT7,CDK1,CNDP2,CORO1C,CPD,CRYZ,CTSD,DDX17,DDX3X,DIS3,DNAJA1,DNMT1,EIF3A,EIF3E,ENO1,FASN,FLNA,G3BP1,G6PD,GART,GNAS,GPNMB,HIST1H1C,HLA-A,HSD17B4,HSP90AA1,HSP90AB1,ICAM1,IDH1,IL6ST,JAK1,KIDINS220,KRT10,LIG1,LYN,MCM2,MFGE8,MSN,MTA2,MVP,MYH9,MYO1G,NCL,NOS2,NOTCH2,NRAS,NUP93,PCNA,PFAS,PKM,PLA2G4A,PLEC,POLR2A,PPP1R7,PPP2R1A,PPP2R2A,PRIM2,PRPF19,PTGS2,PTPRA,PTPRC,PTPRJ,RACK1,RALA,RBBP7,RNF213,RNPEP,RPL7,RPS27A,RPS6,SCFD1,SF3B1,SHMT2,SLC29A1,SLCO4A1,SQSTM1,SRM,STAT1,TGFBR2,TPI1,TRIM25,TSG101,TTC37,UBA1,VIM,XPNPEP1	98
**Cardiovascular System Development and Function,Cell-To-Cell Signaling and Interaction**	Adhesion of endothelial cells	9.1E-08		1.74	ALCAM,CD36,EDIL3,FERMT3,ICAM1,ITGAM,ITGB2,MYADM,RACK1,STAT1,STX6,TGFBR2,TSTA3,VASP,VIM	15

Table S4

**Cancer,Organismal Injury and Abnormalities**	Lymphoreticular neoplasm	9.3E-08		-1.572	ABCG1,ACSL4,ADSL,AHNAK,ALDH9A1,APEX1,ARAP1,ATIC,ATP1A1,CCT3,CCT7,CD36,CD47,CDK1,CLUH,CMIP,CNOT1,CSE1L,DAAM1,DDX3X,DHX15,DIS3,DNAJA1,DNM2,DNMT1,DOCK2,ECPAS,EEF1D,EIF2A,EIF3C,EIF3D,EIF3I,EIF3L,FASN,FCER1G,FLNA,FYN,G3BP1,GART,GMDS,GNA13,GNAI2,GNAS,GRB2,HIST1H1C,HK3,HNRNPM,HNRNPU,HSP90AA1,HSP90AB1,HSPA4,ICAM1,IDH1,IPO5,IPO7,ITGAM,JAK1,KPNA2,KPNB1,KRT2,LILRB4,LYN,MAPK3,MCM7,MYH9,MYO1E,MYO1G,MYOF,NANS,NCSTN,NOS2,NOTCH2,NRAS,OASL,PCNA,PDS5A,PLXNB2,POLD1,PPAT,PPP1R7,PPP2CA,PRIM2,PTGS2,PTPRA,PTPRC,RACK1,RALA,RAN,RIPK3,RNF213,RNPEP,RPL13,RPL28,RPL3,RPL4,RPL6,RPL7,RPS14,RPS27A,RPS6,RPSA,SARS,SDCBP,SF3B1,SHMT1,SHMT2,SIPA1,SMU1,STAT1,STEAP3,SUPT5H,TLR7,TNIP1,TPI1,TRIM25,UBA2,USP8,XPO1,XPO7	119
**Immunological Disease**	Allergy	1.1E-07		1.633	ACO2,AHCY,AHNAK,CFL1,CORO1B,DPYSL2,EIF3E,ENO1,FCER1G,FKBP4,FLNA,FLOT1,FYN,ICAM1,IDE,JAK1,KRT1,KRT10,LILRB4,LYN,MSN,MYH9,NOS2,PHGDH,PLA2G4A,PTGS2,PTPRC,STAT1,TFRC,TKT,TPI1	31
**Carbohydrate Metabolism,Molecular Transport**	Efflux of anthracycline	1.2E-07			Abcb1b,ATP7A,MVP,PTGS2	4
**Cancer,Organismal Injury and Abnormalities,Reproductive System Disease**	Genital tract cancer	1.2E-07			ABCA3,ABCE1,ACLY,ACSL4,ACTR1A,ACTR2,ACTR3,ADSL,ADSSL1,AHNAK,AKR1B1,ALCAM,ALDH9A1,AP3B1,APEX1,ATIC,ATP1A1,ATP2B1,ATP2C1,ATP6V0A1,ATP7A,CA2,CAND1,CAPZA1,CCT2,CCT5,CCT6A,CD36,CD47,CDK1,CLUH,CMIP,CNOT1,COLEC12,COPB1,CORO1C,CPD,CPNE8,CSE1L,CTPS1,CTSD,CTSK,DCTN1,DDX17,DDX3X,DENND4B,DHX29,DIS3,DNAJA1,DNAJC13,DNM1L,DNMT1,DOCK2,ECPAS,EDIL3,EEF1D,EEF1G,EIF2S1,EIF2S3,EIF3A,EIF3C,EIF3D,EIF3E,EIF3F,EIF3I,ENO1,FASN,FKBP4,FLNA,FLOT1,FYN,G3BP1,GART,GFPT1,GMDS,GNA13,GNAI2,GNAS,GNB2,GPNMB,GRB2,GUSB,HIST1H1C,HIST1H2AJ,HK3,HLA-A,HNRNPL,HNRNPM,HNRNPU,HSD17B4,HSP90AA1,HSP90AB1,HSPA4,IDH1,IPO5,ITCH,ITGAM,JAK1,KIDINS220,KPNA2,KRT1,KRT10,KRT9,LIG1,LILRB4,LRP12,MAT2B,MCM2,MCM7,MDH1,MEMO1,MFGE8,MSN,MYADM,MYH9,MYO1C,MYO1E,MYO1G,MYOF,NAA15,NCBP1,NOP56,NOS2,NOTCH2,NPC1,NRAS,NUP93,OASL,OLA1,PCNA,PDS5A,PDXK,PFAS,PGK1,PKM,PLAU,PLD3,PLEC,PLEK,PLXNA1,PLXNB2,PNKP,POLD1,POLR1C,POLR2B,PPAT,PPP1R7,PPP2CA,PPP2R1A,PRIM2,PTGS2,PTPN23,PTPRA,PTPRC,PTPRJ,RALA,RANGAP1,RAP2C,RARS,RBBP7,RCC2,RIPK3,RNF149,RNF213,RNPEP,RPF2,RPL14,RPS27A,RPS6,RRP9,SCFD1,SEC24B,SF3B1,SFPQ,SHMT2,SIRPA,SLC12A4,SLC15A3,SLC16A3,SLC20A1,SLC29A1,SLC2A1,SLC38A2,SLC3A2,SLC7A1,SMPDL3B,SND1,SNRNP200,SNX27,STAT1,STEAP3,STOM,STX6,SUPT5H,TALDO1,TFRC,TGFBR2,TKT,TLR7,TNIP1,TPI1,TPP2,TRIM25,TRIM28,TRPV2,TSG101,TSR1,TTC37,TTLL12,UBA1,UBA2,USP5,USP8,VAMP8,VIM,VPS13C,VWA5A,WWP2,XPO1,XPO7,YBX1	221
**Connective Tissue Disorders,Organismal Injury and Abnormalities,Skeletal and Muscular Disorders**	Non-traumatic arthropathy	1.4E-07		0.218	ACLY,ACTA1,ACTL6A,ADAM8,ATIC,ATP2B1,ATP2C1,C5AR1,CA2,CD36,DDX39B,DNM1L,EEF1G,EIF3E,ENO1,GNB2,GUSB,HELZ2,HLA-A,ICAM1,IDE,IL6ST,ITGB2,JAK1,MAPK3,MYO1C,PFAS,PGK1,POLD1,PPAT,PPP1R7,PREP,PTGS2,PTPRC,RBPJ,RNF149,RPL18A,RPSA,SEC24B,SLC7A1,SND1,SNRNP200,STAT1,TALDO1,TCIRG1,TFRC,TGFBR2,TLR7,TNFRSF1B,TRIM28,VIM	51
**Hematological System Development and Function,Tissue Development**	Accumulation of blood cells	1.5E-07		0.235	ABCG1,C5AR1,DOCK2,EDIL3,GNAI2,ICAM1,IL6ST,ITGAM,ITGB2,LYN,MAPK3,NCSTN,NOS2,NOTCH2,NPC1,PFN1,PLAU,PTGS2,RBPJ,RIPK3,STAT1,TGFBR2,TLR7,TNFRSF1B	24
**Cancer,Hematological Disease,Immunological Disease,Organismal Injury and Abnormalities**	Lymphoma	1.6E-07		-1.941	ABCG1,ADSL,AHNAK,ALDH9A1,ATIC,CCT3,CCT7,CD36,CD47,CDK1,CMIP,CNOT1,CSE1L,DDX3X,DNMT1,DOCK2,EIF2A,FASN,FYN,G3BP1,GART,GNA13,GNAI2,GRB2,HIST1H1C,HNRNPM,HNRNPU,HSP90AA1,HSP90AB1,ICAM1,IDH1,IPO5,IPO7,JAK1,KPNA2,KPNB1,KRT2,MAPK3,MYO1G,MYOF,NOS2,NOTCH2,NRAS,OASL,PCNA,PLXNB2,POLD1,PPAT,PPP2CA,PRIM2,PTGS2,PTPRC,RALA,RAN,RNF213,RPL13,RPSA,SARS,SF3B1,SHMT1,SHMT2,STAT1,STEAP3,TLR7,TNIP1,TRIM25,UBA2,USP8,XPO1	69

Table S4

**Cancer,Hematological Disease,Immunological Disease,Organismal Injury and Abnormalities**	Plasma cell dyscrasia	1.6E-07			CA2,CD36,CD47,CDK1,CTSK,DIS3,FASN,FLNA,G3BP1,GRB2,HLA-A,HSP90AA1,HSP90AB1,IDH1,IL6ST,JAK1,KPNA2,MPEG1,NOS2,NOTCH2,NRAS,PCNA,PPP2CA,PRIM2,PRPF19,PTGS2,PTPRC,RALA,RPS6,SF3B1,TFRC,TGFBR2,VIM	33
**Immunological Disease**	Immediate hypersensitivity	1.6E-07		1.524	AHCY,AHNAK,CFL1,CORO1B,DPYSL2,EIF3E,ENO1,FCER1G,FKBP4,FLNA,FLOT1,FYN,IDE,JAK1,KRT1,KRT10,LILRB4,LYN,MSN,NOS2,PHGDH,PLA2G4A,PTGS2,PTPRC,STAT1,TPI1	26
**Hematological System Development and Function,Immune Cell Trafficking,Inflammatory Response,Tissue Development**	Accumulation of leukocytes	1.7E-07		0.43	ABCG1,C5AR1,DOCK2,EDIL3,GNAI2,ICAM1,IL6ST,ITGAM,ITGB2,LYN,MAPK3,NOS2,NOTCH2,NPC1,PFN1,PLAU,PTGS2,RBPJ,RIPK3,STAT1,TGFBR2,TLR7,TNFRSF1B	23
**Cell-To-Cell Signaling and Interaction**	Binding of gonadal cells	1.7E-07			CCT2,CCT3,CCT4,CCT5,CCT6A,CCT7,CCT8,ICAM1,MFGE8,SLC3A2,TCP1	11
**Cell Death and Survival**	Cell death of blood cells	1.8E-07	Increased	2.028	ABCG1,ADAM8,CD47,CDK1,CTSD,FCER1G,FYN,GNAS,HIST1H1C,HSP90AB1,ICAM1,IL6ST,Irgm1,ITGAM,ITGB2,JAK1,LYN,MAPK3,MVP,NCF2,NOS2,NPC1,NRAS,PCNA,PFN1,PLA2G4A,PTPRC,RAN,RBPJ,RIPK3,RPS6,SF3B1,SIRPA,SLC2A1,STAM2,STAT1,STUB1,TGFBR2,TLR7,TNFRSF1B,TPP2,TRIM28,WWP2	43
**Cell Morphology**	Shape change of leukocytes	1.9E-07		0.896	CD47,DOCK2,FERMT3,ICAM1,ITGAM,ITGB2,KCNN4,LYN,MYH9,PTPRA,RAP1B,SIRPA	12
**Nucleic Acid Metabolism, Small Molecule Biochemistry**	Metabolism of nucleotide	1.9E-07		-0.988	ACLY,ADSL,ADSSL1,ATP5F1B,ATP7A,C5AR1,CDK1,CTPS1,FASN,G6PD,GART,GMDS,GMPPB,GMPS,GNAS,GNB2,MTHFD1,NOS2,OLA1,PFAS,PGD,PKM,PPAT,PTGS2,SHMT1,SNX5,TALDO1,TSTA3	28
**Cancer,Organismal Injury and Abnormalities**	Development of carcinoma	2E-07		0.451	ABCA3,ABCE1,ACLY,ACSL4,ACTA1,ACTR2,ADAM8,ADSL,ADSSL1,AHNAK,AKR1B1,AKR1B10,ALCAM,AP3B1,APEX1,ARAP1,ARPC2,ATIC,ATP2B1,ATP2C1,ATP7A,CA2,CAND1,CCT2,CCT3,CCT5,CCT6A,CCT7,CCT8,CD36,CD47,CDK1,CLUH,CMAS,CMIP,CNDP2,CNOT1,COLEC12,COPB1,CORO1C,CORO7/CORO7-PAM16,CPD,CPNE8,CRYZ,CSE1L,CTPS1,DCTN1,DDX17,DDX21,DDX39B,DDX3X,DENND4B,DHX15,DHX29,DIS3,DKC1,DNAJA1,DNAJC13,DNMT1,DOCK2,ECPAS,EDIL3,EEF1D,EHD1,EIF2S1,EIF2S3,EIF3C,EIF3D,EIF3E,EIF3I,EIF3L,ENO1,ESYT1,FASN,FLNA,FLOT1,FYN,G3BP1,G6PD,GALK1,GART,GFPT1,GMDS,GMPS,GNA13,GNAI2,GNAS,GNB2,GPNMB,GRB2,GUSB,HIST1H1C,HK3,HLA-A,HNRNPL,HNRNPM,HNRNPU,HSD17B4,HSP90AA1,HSP90AB1,HSPA4,IDE,IDH1,IL6ST,IPO5,ITCH,ITGAM,ITGB2,JAK1,KCNN4,KIDINS220,KPNA2,KPNB1,KRT1,KRT10,KRT2,KRT9,LIG1,LILRB4,LRP12,LYN,MAPK3,MARS,MAT2B,MCM2,MCM7,MDH1,MEMO1,MFGE8,MRI1,MSN,MTA2,MYH9,MYO1C,MYO1E,MYOF,NAA15,NANS,NCBP1,NCF2,NCL,NCSTN,NOP56,NOS2,NOTCH2,NPC1,NRAS,NUP93,OASL,OLA1,PCNA,PDXK,PFAS,PGK1,PHGDH,PKM,PLA2G4A,PLAU,PLEC,PLXNA1,PLXNB2,PNKP,POLD1,POLR1C,POLR2A,PPP1CA,PPP1R7,PPP2R1A,PREP,PRIM2,PRPF19,PTGS2,PTPN23,PTPRA,PTPRC,PTPRJ,RAB5B,RALA,RBBP7,RBPJ,RCC2,RHBDF2,RIPK3,RNF149,RNF213,RNH1,RNMT,RNPEP,RPF2,RPL14,RPL28,RPS6,RRP9,RTCB,SARS,SCFD1,SF3B1,SFPQ,SIPA1,SIRPA,SLC15A3,SLC1A5,SLC20A1,SLC23A2,SLC38A2,SLC3A2,SLCO4A1,SLFN13,SMPDL3B,SND1,SNRNP200,SNX2,SNX27,SPRED1,SQSTM1,SRM,STAT1,STOM,STRAP,STUB1,SUPT5H,TALDO1,TAX1BP1,TCIRG1,TCP1,TFRC,TGFBR2,TLR7,TMEM59,TOM1,TPI1,TPP2,TRIM25,TRIM28,TRPV2,TSR1,TTC37,TTLL12,UBA1,UBA2,USP5,USP8,VIM,VPS13C,WARS,WDR82,WWP2,XPO1,XPO7	249

Table S4

**Cancer,Hematological Disease,Organismal Injury and Abnormalities**	Hematologic cancer of cells	2E-07	Decreased	-2.183	ABCG1,ADSL,AHNAK,ALDH9A1,ATIC,CCT3,CCT7,CD36,CD47,CDK1,CFL1,CMIP,CNOT1,CPD,CSE1L,DDX3X,DNM1L,DNMT1,DOCK2,EIF2A,FASN,FYN,G3BP1,GART,GNA13,GNAI2,GRB2,HIST1H1C,HLA-A,HNRNPM,HNRNPU,HSP90AA1,HSP90AB1,ICAM1,IDH1,IPO5,IPO7,ITGAM,ITGB2,JAK1,KPNA2,KPNB1,KRT1,KRT10,KRT2,LYN,MAPK3,MARS,MYO1G,MYOF,NOS2,NOTCH2,NRAS,OASL,PCNA,PLXNB2,POLD1,PPAT,PPP2CA,PRIM2,PTGS2,PTPRC,RALA,RAN,RNF213,RPL13,RPSA,SARS,SF3B1,SHMT1,SHMT2,SLC2A1,SPRED1,STAT1,STEAP3,TAX1BP1,TGFBR2,TLR7,TNIP1,TRIM25,UBA2,USP8,XPO1	83
**Cancer,Hematological Disease,Immunological Disease,Organismal Injury and Abnormalities**	B-cell non-Hodgkin lymphoma	2E-07			ABCG1,ADSL,AHNAK,CCT3,CD36,CDK1,CNOT1,DNMT1,DOCK2,EIF2A,FASN,FYN,G3BP1,GNA13,GNAI2,GRB2,HIST1H1C,HNRNPM,HNRNPU,HSP90AA1,HSP90AB1,IDH1,JAK1,KPNA2,KRT2,MYO1G,MYOF,NOS2,NOTCH2,NRAS,PCNA,POLD1,PPAT,PPP2CA,PRIM2,PTGS2,PTPRC,RALA,RNF213,RPSA,SARS,SF3B1,SHMT1,SHMT2,STEAP3,TLR7,TNIP1,USP8,XPO1	49
**Cellular Assembly and Organization,Cellular Function and Maintenance**	Microtubule dynamics	2.1E-07		0.436	ACACA,ACTR2,ACTR3,AHNAK,ARAP1,ARPC2,ATP7A,CD47,CDK1,CFL1,DAAM1,DCTN1,DNM1L,DNM2,DPYSL2,FASN,FERMT3,FKBP4,FLNA,FYN,GNA13,GNAS,HSP90AA1,HSP90AB1,ICAM1,IDE,ITGAM,KIDINS220,KPNB1,LYN,MSN,MYH9,NCF2,NDRG1,PFN1,PLAU,PLEC,PTGS2,PTPN23,RAB31,RAB5A,RALA,RAN,SIRPA,SNX2,TLR7,VASP,VIM	48
**Cell Death and Survival**	Cell death of tumor cell lines	2.2E-07		1.205	AKR1B1,APEX1,ATP1B3,CCT2,CDK1,CSE1L,FASN,GNAS,HSP90AB1,HSPA4,IL6ST,ITGAM,LIG1,MAPK3,MTA2,MVP,NOS2,NOTCH2,NRAS,PCNA,PKM,PLAU,PPP2CA,PTPRC,RIPK3,SLC29A1,STAT1,TGFBR2,TNFRSF1B,TRIM28,UCHL5,XPO1,YBX1	33
**Cancer,Gastrointestinal Disease,Hepatic System Disease,Organismal Injury and Abnormalities**	Liver cancer	2.2E-07		-0.133	ABCA3,ABCG1,ACAA1,ACLY,ACO2,ACTR1A,ACTR2,ACTR3,AHNAK,AKR1B10,ALCAM,ALDH9A1,APEX1,ARAP1,ATIC,ATP1A1,ATP1B3,ATP6V0A1,ATP7A,CA2,CCT6A,CCT8,CD36,CD47,CNDP2,CNOT1,COPG1,CPNE8,CSE1L,CTPS1,CTSD,DAAM1,DDX17,DDX39B,DDX3X,DIS3,DKC1,DNAJA1,DNAJC13,DNM1L,DOCK2,EDIL3,EIF3A,EIF3E,EIF3I,ENO1,FARSA,FASN,FERMT3,FLNA,G6PD,GALK1,GARS,GMPS,GNA13,GNAS,GRB2,HNRNPM,HSP90AA1,HSP90AB1,HSPA4,ICAM1,IDE,IDH1,IFITM3,IL6ST,IPO5,IPO7,ITGAM,JAK1,KIDINS220,KPNB1,KRT10,KRT9,MARS,MAT2A,MAT2B,MCM2,MCM7,MDH1,MSTO1,MTA2,MTHFD1,MVP,MYADM,MYO1C,MYO1E,MYOF,NAA15,NCBP1,NCL,NDRG1,NOP56,NOS2,NPC1,NRAS,OASL,PDS5A,PFAS,PFN1,PHGDH,PKM,PLA2G4A,PLAU,PLD3,PLEC,PLXNA1,POLD1,POLR2B,PPP2CA,PPP2R1A,PRIM2,PRPF4,PTGS2,PTPRA,PTPRC,RAB8B,RACK1,RALA,RAN,RAP1B,RBPJ,RNF213,RNMT,RNPEP,RPL27A,RPL6,RPL7A,RPN1,RPSA,RTCB,SARS,SCFD1,SEC24B,SF3B1,SFPQ,SLC16A6,SLC1A5,SLC29A1,SLC2A1,SLC38A2,SLC4A7,SLC7A1,SND1,SNRNP200,SNX2,SNX27,SQSTM1,SRM,STAM2,STAT1,SUPT5H,TAX1BP1,TCIRG1,TGFBR2,TLR7,TNFRSF1B,TNIP1,TPI1,TPP2,TRIM25,TRIM28,TTC37,UBA2,UCHL5,VIM,VPS13C,WARS,XPNPEP1	169
**Cancer,Hematological Disease,Organismal Injury and Abnormalities**	Mature lymphocytic neoplasm	2.3E-07			ABCG1,ADSL,AHNAK,CA2,CCT3,CD36,CD47,CDK1,CFL1,CNOT1,CPD,CTSK,DIS3,DNM1L,DNMT1,DOCK2,EIF2A,FASN,FLNA,FYN,G3BP1,GNA13,GNAI2,GRB2,HIST1H1C,HLA-A,HNRNPM,HNRNPU,HSP90AA1,HSP90AB1,IDH1,IL6ST,ITGB2,JAK1,KPNA2,KRT1,KRT10,KRT2,LYN,MAPK3,MARS,MPEG1,MYO1G,MYOF,NOS2,NOTCH2,NRAS,OASL,PCNA,PLXNB2,POLD1,PPP2CA,PREP,PRIM2,PRPF19,PTGS2,PTPRC,RALA,RPS6,RPSA,SARS,SF3B1,SHMT1,SHMT2,STAT1,STEAP3,TFRC,TGFBR2,TLR7,TNIP1,TRIM25,USP8,VIM,XPO1	74

Table S4

**Cancer,Organismal Injury and Abnormalities**	Development of malignant tumor	2.5E-07		-0.971	ABCA3,ABCE1,ACLY,ACSL4,ACTA1,ACTR2,ADAM8,ADSL,ADSSL1,AHNAK,AKR1B1,AKR1B10,ALCAM,AP3B1,APEX1,ARAP1,ARPC2,ATIC,ATP2B1,ATP2C1,ATP7A,CA2,CAND1,CCT2,CCT3,CCT5,CCT6A,CCT7,CCT8,CD36,CD47,CDK1,CLUH,CMAS,CMIP,CNDP2,CNOT1,COLEC12,COPB1,CORO1C,CORO7/CORO7-PAM16,CPD,CPNE8,CRYZ,CSE1L,CTPS1,DCTN1,DDX17,DDX21,DDX39B,DDX3X,DENND4B,DHX15,DHX29,DIS3,DKC1,DNAJA1,DNAJC13,DNMT1,DOCK2,ECPAS,EDIL3,EEF1D,EHD1,EIF2S1,EIF2S3,EIF3C,EIF3D,EIF3E,EIF3I,EIF3L,ENO1,ESYT1,FASN,FLNA,FLOT1,FYN,G3BP1,G6PD,GALK1,GART,GFPT1,GMDS,GMPS,GNA13,GNAI2,GNAS,GNB2,GPNMB,GRB2,GUSB,HIST1H1C,HK3,HLA-A,HNRNPL,HNRNPM,HNRNPU,HSD17B4,HSP90AA1,HSP90AB1,HSPA4,IDE,IDH1,IL6ST,IPO5,ITCH,ITGAM,ITGB2,JAK1,KCNN4,KIDINS220,KPNA2,KPNB1,KRT1,KRT10,KRT2,KRT9,LGALS3BP,LIG1,LILRB4,LRP12,LYN,MAPK3,MARS,MAT2B,MCM2,MCM7,MDH1,MEMO1,MFGE8,MRI1,MSN,MTA2,MYH9,MYO1C,MYO1E,MYOF,NAA15,NANS,NCBP1,NCF2,NCL,NCSTN,NOP56,NOS2,NOTCH2,NPC1,NRAS,NUP93,OASL,OLA1,PCNA,PDXK,PFAS,PGK1,PHGDH,PKM,PLA2G4A,PLAU,PLEC,PLXNA1,PLXNB2,PNKP,POLD1,POLR1C,POLR2A,PPP1CA,PPP1R7,PPP2R1A,PREP,PRIM2,PRPF19,PTGS2,PTPN23,PTPRA,PTPRC,PTPRJ,RAB5B,RALA,RBBP7,RBPJ,RCC2,RHBDF2,RIPK3,RNF149,RNF213,RNH1,RNMT,RNPEP,RPF2,RPL14,RPL28,RPS6,RRP9,RTCB,SARS,SCFD1,SF3B1,SFPQ,SIPA1,SIRPA,SLC15A3,SLC1A5,SLC20A1,SLC23A2,SLC38A2,SLC3A2,SLCO4A1,SLFN13,SMPDL3B,SND1,SNRNP200,SNX2,SNX27,SPRED1,SQSTM1,SRM,STAT1,STOM,STRAP,STUB1,SUPT5H,TALDO1,TAX1BP1,TCIRG1,TCP1,TFRC,TGFBR2,TKT,TLR7,TMEM59,TOM1,TPI1,TPP2,TRIM25,TRIM28,TRPV2,TSR1,TTC37,TTLL12,UBA1,UBA2,USP5,USP8,VIM,VPS13C,WARS,WDR82,WWP2,XPO1,XPO7	251
**Cell-To-Cell Signaling and Interaction, Cellular Assembly and Organization,Hematological System Development and Function,Immune Cell Trafficking**	Cell-cell contact of leukocytes	3.1E-07			ALCAM,CD47,FERMT3,ICAM1,ITGAM,ITGB2,PTPRC,TNIP1	8
**Post-Translational Modification**	Phosphorylation of protein	3.4E-07		1.188	CD36,CD47,CDK1,CFL1,CORO1C,CSNK1G3,EDIL3,EIF2A,EIF2S1,FCER1G,FLNA,FLOT1,FYN,GNAS,GPNMB,HSP90AA1,HSPA4,IRGM,ITGAM,ITGB2,JAK1,LYN,MAPK3,MCM7,MYADM,NRAS,PLXNB2,PPP2CA,PTPRA,PTPRC,PTPRJ,RACK1,RIPK3,SDCBP,SIRPA,SLC3A2,SQSTM1,TGFBR2,TLR7,TRIM28,WARS	41
**RNA Post-Transcriptional Modification**	Processing of mRNA	3.5E-07			CMTR1,CPSF1,DDX17,DDX39B,DHX15,HNRNPL,HNRNPM,HNRNPU,MTREX,NCBP1,POLR2A,POLR2B,PRPF19,PRPF4,RNMT,SF3B1,SFPQ,SMU1,SNRNP200,SNRNP40,SUPT5H,YBX1	22
**Cardiovascular System Development and Function,Cell-To-Cell Signaling and Interaction**	Binding of endothelial cells	3.6E-07		1.197	ALCAM,CD36,CD47,EDIL3,FERMT3,ICAM1,ITGAM,ITGB2,KRT1,MYADM,RACK1,STAT1,STX6,TGFBR2,TSTA3,VASP,VIM	17
**Organismal Development**	Growth of organism	3.7E-07		0.414	Abcb1b,ACACA,CD36,CFL1,CTSD,DCTN1,DDX3X,DHX15,DNM1L,DNMT1,EIF3E,G6PD,GNA13,GNAS,HLA-A,HSD17B4,HSP90AA1,HSP90AB1,IL6ST,ITGAM,LIG1,LYN,MAPK3,MFGE8,NCL,NOS2,NOTCH2,NRAS,OLA1,PLXNB2,PNKP,PPP1CA,PRPF19,PTGS2,RACK1,RAN,RIPK3,SNX2,SNX27,STAT1,TCIRG1,TFRC,TGFBR2,TKT,TSG101,ZC3HAV1	46
**Cell-To-Cell Signaling and Interaction**	Response of antigen presenting cells	3.7E-07		1.877	ATP7A,CD36,CD47,DOCK2,FCER1G,FYN,HSP90AA1,IL6ST,ITGAM,ITGB2,LILRB4,LYN,MFGE8,NOS2,PTPRC,PTPRJ,SIRPA,TLR7,TRPV2	19

Table S4

**Hematological System Development and Function,Immunological Disease,Lymphoid Tissue Structure and Development,Organ Morphology,Organismal Development,Organismal Injury and Abnormalities,Tissue Morphology**	Abnormal morphology of spleen	3.8E-07			CD47,CTSD,DOCK2,FCER1G,FYN,GBA,IL6ST,ITGB2,JAK1,KCNN4,LIG1,LYN,MAPK3,MFGE8,NRAS,PTGS2,PTPRC,PTPRJ,RIPK3,SIPA1,SNX27,STEAP3,TCIRG1,TLR7,TPP2	25
**Carbohydrate Metabolism, Molecular Transport**	Efflux of carbohydrate	4E-07	Increased	2.219	Abcb1b,ABCG1,ATP7A,MVP,NPC1,PTGS2	6
**Cell-To-Cell Signaling and Interaction**	Binding of germ cells	4E-07			CCT2,CCT3,CCT4,CCT5,CCT6A,CCT7,CCT8,MFGE8,SLC3A2,TCP1	10
**Cellular Assembly and Organization,Cellular Function and Maintenance**	Organization of cytoskeleton	4E-07		0.644	ACACA,ACTR2,ACTR3,AHNAK,ARAP1,ARPC2,ATP2C1,ATP7A,CD47,CDK1,CFL1,CORO1B,CORO1C,CORO7/CORO7-PAM16,DAAM1,DCTN1,DNM1L,DNM2,DOCK2,DPYSL2,FASN,FERMT3,FKBP4,FLNA,FYN,GNA13,GNAS,HSP90AA1,HSP90AB1,ICAM1,IDE,ITGAM,KIDINS220,KPNB1,KRT9,LYN,MAPK3,MSN,MYH9,NCF2,NDRG1,PFN1,PLAU,PLEC,PLEK,PTGS2,PTPN23,RAB31,RAB5A,RALA,RAN,RPS6,SIRPA,SNX2,TLR7,VASP,VIM	57
**Cancer,Organismal Injury and Abnormalities**	Metastatic solid tumor	4.2E-07		1.706	ATIC,C5AR1,CD36,COLEC12,CRYZ,CSE1L,DPYSL2,FASN,FLNA,FYN,GART,GNAS,GPNMB,HIST1H1C,HSP90AA1,HSP90AB1,ICAM1,JAK1,KIDINS220,LYN,MYO1C,NRAS,NUP93,PLAU,PLEC,POLD1,PPP2R1A,PRIM2,PTGS2,PTPRC,PTPRJ,RAB31,RNH1,RPL7,RPS27A,RPS6,SLC16A3,SQSTM1,STAT1,STEAP3,XPO1	41
**Cell Morphology**	Polarization of cells	4.3E-07	Increased	2.204	CD36,CD47,CFL1,DAAM1,DOCK2,DPYSL2,FCER1G,FYN,GNAI3,ITGB2,MSN,MYH9,NRAS,RBPJ,SIPA1,SIRPA	16
**Cell Signaling,DNA Replication, Recombination, and Repair,Nucleic Acid Metabolism, Small Molecule Biochemistry**	Hydrolysis of GTP	4.7E-07		0.028	CDK1,DNM1L,GNAI2,GNAI3,GNAS,IPO5,RAB7A,RAN,RANGAP1,XPO1	10

Table S4

**Cell-To-Cell Signaling and Interaction,Inflammatory Response**	Immune response of antigen presenting cells	4.8E-07		1.712	CD36,CD47,DOCK2,FCER1G,FYN,HSP90AA1,IL6ST,ITGAM,ITGB2,LILRB4,LYN,MFGE8,NOS2,PTPRC,PTPRJ,SIRPA,TLR7,TRPV2	18
**Cell-To-Cell Signaling and Interaction**	Response of myeloid cells	5E-07	Increased	2.615	ATP7A,C5AR1,CD36,CD47,DOCK2,FCER1G,FYN,ICAM1,IL6ST,ITGAM,ITGB2,LYN,MFGE8,NOS2,PLAU,PTPRC,PTPRJ,SIRPA,TLR7,TRPV2	20
**Hematological System Development and Function,Humoral Immune Response,Lymphoid Tissue Structure and Development,Tissue Morphology**	Quantity of B lymphocytes	5.2E-07		1.21	ABCG1,CD36,DKC1,DOCK2,FCER1G,FYN,GNA13,IL6ST,ITGB2,JAK1,KIDINS220,LILRB4,LYN,MYO1G,NOS2,NOTCH2,PTPRC,PTPRJ,RAP1B,RBPJ,RIPK3,SLC2A1,STAT1,TCIRG1,TGFBR2,TNFRSF1B,TPP2	27
**Cell-To-Cell Signaling and Interaction,Inflammatory Response**	Response of phagocytes	5.9E-07	Increased	2.599	ATP7A,C5AR1,CD36,CD47,DOCK2,FCER1G,FYN,ICAM1,IL6ST,ITGAM,ITGB2,LYN,MFGE8,NOS2,PLAU,PTPRC,PTPRJ,SIRPA,TLR7,TRPV2	20
**Cell-To-Cell Signaling and Interaction**	Binding of lymphatic system cells	6.1E-07		1.653	ALCAM,CD47,FERMT3,FLOT1,GNAI2,ICAM1,IL6ST,ITGB2,JAK1,LYN,MSN,MYADM,MYO1G,PLAU,PPP2CA,RAP1B,TFRC	17
**Post-Translational Modification,Protein Synthesis,Protein Trafficking**	Tetramerization of protein	6.2E-07			ACACA,ADSL,CRYZ,DNM1L,ENO1,FARSA,FARSB,GFPT1,IDE,KRT1,KRT10,PKM,PPAT,SHMT1,SHMT2	15
**Dermatological Diseases and Conditions,Immunological Disease,Inflammatory Disease,Inflammatory Response,Organismal Injury and Abnormalities**	Atopic dermatitis	6.2E-07			AHCY,AHNAK,CFL1,DPYSL2,EIF3E,ENO1,FCER1G,FKBP4,FLNA,FLOT1,FYN,IDE,JAK1,KRT1,KRT10,LYN,MSN,PHGDH,PTGS2,STAT1,TPI1	21

Table S4

**Cell Death and Survival**	Cell death of immune cells	6.4E-07		1.625	ABCG1,ADAM8,CD47,CDK1,CTSD,FCER1G,FYN,GNAS,HIST1H1C,HSP90AB1,ICAM1,IL6ST,Irgm1,ITGAM,ITGB2,JAK1,LYN,MAPK3,MVP,NCF2,NOS2,NPC1,NRAS,PCNA,PLA2G4A,PTPRC,RAN,RBPJ,RIPK3,RPS6,SIRPA,SLC2A1,STAM2,STAT1,STUB1,TGFBR2,TLR7,TNFRSF1B,TPP2,WWP2	40
**Cancer,Organismal Injury and Abnormalities,Respiratory Disease**	Non-small cell lung carcinoma	6.4E-07		1.982	AHNAK,AKR1B10,ALCAM,AP3B1,APEX1,ATIC,ATP1A1,ATP5F1B,CCT7,CDK1,CNDP2,CORO1C,CPD,CRYZ,CTSD,DDX17,DDX3X,DIS3,EIF3E,ENO1,FASN,G3BP1,GART,GNAS,GPNMB,HIST1H1C,HLA-A,HSD17B4,HSP90AA1,HSP90AB1,ICAM1,IDH1,IL6ST,JAK1,KIDINS220,KRT10,LIG1,LYN,MCM2,MSN,MTA2,MYH9,NOTCH2,NRAS,NUP93,PCNA,PFAS,PKM,PLA2G4A,PLEC,POLR2A,PPP1R7,PPP2R1A,PPP2R2A,PRIM2,PRPF19,PTGS2,PTPRA,PTPRC,RACK1,RALA,RNF213,RPL7,RPS27A,RPS6,SCFD1,SF3B1,SLC29A1,SLCO4A1,SRM,STAT1,TGFBR2,TPI1,TRIM25,TTC37,VIM,XPNPEP1	77
**Hematological System Development and Function,Immunological Disease,Lymphoid Tissue Structure and Development,Organ Morphology,Organismal Injury and Abnormalities,Tissue Morphology**	Abnormal morphology of lymphoid organ	6.7E-07			CD47,CTSD,DOCK2,FCER1G,FYN,GBA,HIST1H1C,IL6ST,ITGB2,JAK1,KCNN4,LIG1,LYN,MAPK3,MFGE8,NOS2,NRAS,POLD1,PTGS2,PTPRC,PTPRJ,RIPK3,SIPA1,SNX27,STEAP3,TCIRG1,TLR7,TNFRSF1B,TPP2	29
**Cell-To-Cell Signaling and Interaction**	Activation of cells	7.1E-07		1.962	ADAM8,AHNAK,AP3B1,C5AR1,CD36,CD47,CFL1,DOCK2,EIF3A,FCER1G,FLNA,FYN,GNA13,GNAI2,GNAS,GPNMB,HLA-A,HSPA4,ICAM1,IL6ST,ITCH,ITGAM,ITGB2,KCNN4,KIDINS220,KRT2,LGALS3BP,LILRB4,LYN,MAPK3,NDRG1,NOS2,NOTCH2,NPC1,PLA2G4A,PLAU,PLEK,PLXNB2,PTGS2,PTPRC,PTPRJ,RAB5B,RAB8B,RBPJ,SIRPA,SQSTM1,STAT1,TGFBR2,TLR7,TNFRSF1B,VAMP8,VASP,VIM	53
**Cellular Movement,Hematological System Development and Function,Immune Cell Trafficking,Inflammatory Response**	Cell movement of neutrophils	7.1E-07		1.849	ADAM8,C5AR1,CD36,CD47,DOCK2,EDIL3,FCER1G,FERMT3,FLOT1,GNAI2,GNAI3,ICAM1,IL6ST,ITGAM,ITGB2,LYN,NOS2,PLAU,PTGS2,RAP1B,TGFBR2,TNFRSF1B,TNIP1,YBX1	24
**Cancer,Gastrointestinal Disease,Hepatic System Disease,Organismal Injury and Abnormalities**	Bile duct adenocarcinoma	7.3E-07			GNAS,HSP90AA1,HSP90AB1,IDH1,NOS2,NRAS,PGK1,PKM,PLAU,PTGS2,RACK1,RPL4,SF3B1,VIM	14

Table S4

**Cellular Development,Connective Tissue Development and Function,Skeletal and Muscular System Development and Function,Tissue Development**	Differentiation of osteoclasts	7.4E-07		1.811	ADAM8,C5AR1,CA2,CD47,FCER1G,GNAS,GPNMB,GRB2,IL6ST,LILRB4,NOS2,NOTCH2,PTGS2,SIRPA,SLC1A5,SQSTM1,STAT1,TFRC	18
**RNA Post-Transcriptional Modification**	Splicing of mRNA	7.5E-07			CPSF1,DDX17,DDX39B,DHX15,HNRNPL,HNRNPM,HNRNPU,MTREX,NCBP1,POLR2A,POLR2B,PRPF19,PRPF4,SF3B1,SFPQ,SMU1,SNRNP200,SNRNP40,YBX1	19
**Cancer,Gastrointestinal Disease,Hepatic System Disease,Organismal Injury and Abnormalities**	Biliary tract adenocarcinoma	7.9E-07			DOCK2,GNAS,HSP90AA1,HSP90AB1,IDH1,KPNA2,NOS2,NRAS,PGK1,PKM,PLAU,PTGS2,RACK1,RPL4,SF3B1,TGFBR2,VIM	17
**Cell-To-Cell Signaling and Interaction, Reproductive System Development and Function**	Binding of sperm	8.1E-07			CCT2,CCT3,CCT4,CCT5,CCT6A,CCT7,CCT8,MFGE8,TCP1	9
**Carbohydrate Metabolism**	Metabolism of carbohydrate	8.2E-07		-0.605	Abcb1b,AKR1B1,AMDHD2,AP3B1,CD36,CMAS,G6PD,GALK1,GFPT1,GMDS,GMPPB,GUSB,ICAM1,IDH1,IL6ST,ITGB2,LYN,MYOF,NANS,NOS2,PGD,PGK1,PI4K2A,PI4K2B,PKM,PLA2G4A,PLAU,PLEK,PPP1CA,PTPRC,RAB5A,RALA,SLC16A3,SLC23A2,SLC2A1,TALDO1,TKT,TPI1,TRPV2,TSTA3	40
**Immunological Disease,Inflammatory Disease**	Atopic disease	8.8E-07			AHCY,AHNAK,CFL1,DPYSL2,EIF3E,ENO1,FCER1G,FKBP4,FLNA,FLOT1,FYN,IDE,JAK1,KRT1,KRT10,LYN,MSN,NOS2,PHGDH,PTGS2,STAT1,TPI1	22
**Cell Morphology, Cellular Movement**	Cell spreading of blood cells	9.4E-07		0.822	CD36,CD47,FERMT3,FLNA,ICAM1,ITGAM,ITGB2,LYN,PTPRA,RAP1B,SIRPA	11
**Cellular Movement**	Cellular infiltration by blood cells	9.6E-07		1.482	ABCG1,ADAM8,AKR1B1,C5AR1,CD36,CD47,EDIL3,FCER1G,GBA,HLA-A,ICAM1,IL6ST,ITGAM,ITGB2,KRT10,MAPK3,NDRG1,NOS2,NPC1,PLA2G4A,PLAU,PLEC,PTGS2,STAT1,TCIRG1,TGFBR2,TNFRSF1B,TNIP1,YBX1	29
**Cancer,Gastrointestinal Disease,Hepatic System Disease,Organismal Injury and Abnormalities**	Cholangiocarcinoma	9.8E-07			GNAS,HSP90AA1,HSP90AB1,IDH1,NOS2,NRAS,PGK1,PKM,PLAU,PTGS2,RACK1,RPL4,VIM	13

Table S4

**Inflammatory Disease**	Chronic inflammatory disorder	9.9E-07			ABCG1,ACLY,ACTA1,ACTL6A,ADAM8,ATIC,ATP2B1,ATP2C1,C5AR1,CA2,CD36,CTSD,DDX39B,DNM1L,EEF1G,EIF3E,ENO1,G6PD,GNB2,GUSB,HELZ2,HLA-A,ICAM1,IDE,IL6ST,IRGM,ITGB2,JAK1,MAPK3,MYO1C,NCF2,NOS2,NOTCH2,PFAS,PGK1,PKM,POLD1,PPAT,PPP1R7,PREP,PTGS2,PTPRC,RNF149,RPL18A,RPSA,SEC24B,SF3B1,SLC7A1,SND1,SNRNP200,STAT1,TALDO1,TCIRG1,TFRC,TGFBR2,TLR7,TNFRSF1B,TRIM28,VIM	59
**Hereditary Disorder,Neurological Disease,Organismal Injury and Abnormalities**	Hereditary neuropathy	1E-06			ACO2,ACTA1,ATP1A1,ATP7A,CCT5,DCTN1,DNAJC13,DNM2,DNMT1,GARS,GBA,KIDINS220,MARS,NDRG1,PFN1,RAB7A,SQSTM1,UBA1,USP8,VPS13C,WARS	21
**Hereditary Disorder,Neurological Disease,Organismal Injury and Abnormalities**	Familial motor neuron disease	1.1E-06			ATP7A,DCTN1,DNAJC13,GARS,GBA,MARS,NOP56,PFN1,SQSTM1,UBA1,VPS13C,WARS	12
**Cancer,Organismal Injury and Abnormalities,Reproductive System Disease**	Mammary tumor	1.2E-06		0.624	ACAA1,AKR1B1,AP3B1,ATP1A1,ATP2C1,CCT3,CDK1,CMAS,CNDP2,CSE1L,CTSD,CTSK,DDX17,DDX39B,DENND4B,DHX29,DKC1,DNM1L,EIF2A,EIF3A,EIF3B,EIF3C,EIF3E,EIF3F,ENO1,FASN,FERMT3,FLNA,FYN,GBA,GNA13,GNAI3,GNAS,GPNMB,HIST1H1C,HLA-A,HNRNPM,HSP90AA1,HSP90AB1,IDH1,Ifi202b,ITCH,JAK1,KPNA2,KRT1,LGALS3BP,LYN,MAPK3,MCM2,MOV10,MYH9,NANS,NCF2,NCL,NOTCH2,NPC1,NRAS,NUP93,NUS1,PCNA,PFN1,PGK1,PI4K2A,PKM,PLAU,PLEC,PLXNA1,POLD1,POLR2A,PPP2R1A,PRIM2,PTGS2,PTPRA,PTPRC,PTPRJ,RAB31,RAB5C,RANGAP1,RAP1B,RPL4,RPL6,RTCB,SF3B1,SFPQ,SHMT2,SLC20A1,SLC23A2,SLC4A7,STAT1,STEAP3,SUPT5H,TCP1,TGFBR2,TNFRSF1B,TPI1,TRIM25,TRPV2,TSG101,UBA1,UBA2,UCHL5,VWA5A,WWP2,XPO1	104
**Hematological System Development and Function,Lymphoid Tissue Structure and Development,Tissue Morphology**	Quantity of T lymphocytes	1.2E-06		0.375	Abcb1b,AP3B1,C5AR1,CD47,CTSD,DKC1,DNMT1,DOCK2,EEF1D,FCER1G,FYN,GNAI2,HLA-A,ICAM1,IL6ST,ITGAM,ITGB2,JAK1,LIG1,LYN,MAPK3,NOS2,NOTCH2,PTPRC,RBPJ,RIPK3,SIPA1,SIRPA,STAM2,STAT1,TCIRG1,TGFBR2,TNFRSF1B,TNIP1,TPP2	35
**Molecular Transport,Protein Trafficking**	Import of protein	1.2E-06	Decreased	-2.121	CFL1,FLNA,IPO5,IPO7,KPNA2,KPNB1,PTGS2,RAN,RANGAP1,TRIM28,XPO1	11
**Cell Death and Survival**	Apoptosis of tumor cell lines	1.3E-06		0.505	AKR1B1,ATP1B3,CCT2,CDK1,CSE1L,FASN,GNAS,HSP90AB1,HSPA4,IL6ST,LIG1,MAPK3,MTA2,MVP,NOS2,NRAS,PCNA,PKM,PLAU,PPP2CA,STAT1,TGFBR2,TNFRSF1B,TRIM28,UCHL5,XPO1,YBX1	27
**Dermatological Diseases and Conditions, Organismal Injury and Abnormalities**	Chronic psoriasis	1.3E-06			CCT5,CD47,EIF2S1,GARS,KPNA2,KPNB1,KRT2,LGALS3BP,PGD,PKM,PPP2CA,SLC23A2,SRM,STAT1	14

Table S4

**Dermatological Diseases and Conditions, Organismal Injury and Abnormalities**	Psoriasis	1.3E-06			CCT5,CD36,CD47,CFL1,CTSK,EIF2S1,FASN,GARS,GBA,ICAM1,IL6ST,ITGB2,JAK1,KPNA2,KPNB1,KRT1,KRT10,KRT2,LGALS3BP,LYN,NOS2,OASL,PCNA,PGD,PKM,PLAU,PPP2CA,PTGS2,PTPRC,RAB5A,RAN,SLC23A2,SRM,STAT1,TNIP1	35
**Cell Morphology, Cellular Assembly and Organization,Cellular Function and Maintenance**	Formation of cellular protrusions	1.4E-06		1.014	ACACA,ACTR2,ACTR3,AHNAK,ARAP1,ARPC2,ATP7A,CD47,CFL1,DCTN1,DNM1L,DNM2,DPYSL2,FASN,FERMT3,FLNA,FYN,GNA13,HSP90AA1,HSP90AB1,KIDINS220,LYN,MSN,NCF2,PFN1,PLAU,PLEC,PTGS2,PTPN23,RAB31,RAB5A,RALA,SIRPA,SNX2,TLR7,VIM	36
**Organismal Survival**	Survival of organism	1.4E-06		0.229	ABCA3,Abcb1b,APEX1,ATP2C1,C5AR1,CD47,DDX3X,DNMT1,EIF3M,FASN,GNA13,GNAI2,GNAS,HLA-A,ICAM1,IDE,IDH1,IL6ST,Irgm1,ITGAM,MCM2,MTA2,MVP,NCL,NOS2,NOTCH2,NPC1,NRAS,PKM,PNKP,PTGS2,PTPRC,RAB5A,RIPK3,SF3B1,SLC12A4,SLC29A1,SLC2A1,SNX27,STAT1,TGFBR2,TLR7,TNFRSF1B,TSG101	44
**Cell-To-Cell Signaling and Interaction, Cellular Function and Maintenance,Inflammatory Response**	Phagocytosis of blood cells	1.5E-06		1.717	CD36,CD47,DOCK2,FCER1G,FYN,ICAM1,ITGAM,ITGB2,LYN,MFGE8,MYO1G,PLAU,PTPRC,PTPRJ,SIRPA,TRPV2	16
**Cancer,Organismal Injury and Abnormalities,Respiratory Disease**	Lung carcinoma	1.5E-06		1.518	AHNAK,AKR1B10,ALCAM,AP3B1,APEX1,ARAP1,ATIC,ATP1A1,ATP5F1B,CCT7,CDK1,CNDP2,CORO1C,CPD,CRYZ,CTSD,DDX17,DDX3X,DIS3,DNAJA1,EIF3E,ENO1,FASN,FLNA,G3BP1,G6PD,GART,GNAS,GPNMB,HIST1H1C,HLA-A,HSD17B4,HSP90AA1,HSP90AB1,ICAM1,IDH1,IL6ST,JAK1,KIDINS220,KRT10,LIG1,LYN,MCM2,MFGE8,MSN,MTA2,MVP,MYH9,MYO1G,NOS2,NOTCH2,NRAS,NUP93,PCNA,PFAS,PKM,PLA2G4A,PLEC,POLR2A,PPP1R7,PPP2R1A,PPP2R2A,PRIM2,PRPF19,PTGS2,PTPRA,PTPRC,RACK1,RALA,RBBP7,RNF213,RPL7,RPS27A,RPS6,SCFD1,SF3B1,SLC29A1,SLCO4A1,SRM,STAT1,TGFBR2,TPI1,TRIM25,TSG101,TTC37,UBA1,VIM,XPNPEP1	88
**Cell-To-Cell Signaling and Interaction, Hematological System Development and Function,Immune Cell Trafficking,Inflammatory Response**	Binding of macrophages	1.5E-06		1.807	ICAM1,Irgm1,ITGAM,ITGB2,LYN,MSN,PLAU,PTGS2,PTPRC,TRPV2	10
**Organismal Injury and Abnormalities**	Visceromegaly	1.6E-06			CA2,CD36,CD47,CTSD,DNM1L,EEF1D,FASN,FCER1G,FYN,GBA,GNAI2,GNAS,GRB2,ICAM1,IL6ST,ITGB2,JAK1,KCNN4,LIG1,LYN,MAPK3,MFGE8,MYH9,MYO1E,NOS2,NPC1,NRAS,PFN1,PGK1,PLA2G4A,PLAU,PPP1CA,PPP2CA,PTGS2,PTPRC,RIPK3,SIPA1,STAT1,TGFBR2,TLR7,TNFRSF1B,TPI1,TPP2	43

Table S4

**Molecular Transport,Protein Trafficking**	Internalization of protein	1.6E-06		-1.406	CFL1,FLNA,IPO5,IPO7,KPNA2,KPNB1,PTGS2,RAB5A,RAN,RANGAP1,TRIM28,XPO1	12
**Cell Morphology, Cellular Assembly and Organization,Cellular Function and Maintenance**	Formation of lamellipodia	1.7E-06		-0.463	ACTR3,ARPC2,ATP7A,CFL1,DNM1L,DNM2,FERMT3,FYN,HSP90AA1,NCF2,PFN1,PLAU,RAB5A,SNX2	14
**RNA Post-Transcriptional Modification**	Splicing of RNA	1.7E-06			AHNAK,CPSF1,DDX17,DDX39B,DHX15,HNRNPL,HNRNPM,HNRNPU,MTREX,NCBP1,POLR2A,POLR2B,PRPF19,PRPF4,SF3B1,SFPQ,SMU1,SNRNP200,SNRNP40,YBX1	20
**Cellular Assembly and Organization**	Quantity of actin filaments	1.8E-06		1	ARAP1,CFL1,DNM2,FYN,ITGB2,NRAS,PLAU,PLEC	8
**Developmental Disorder**	Growth Failure	1.8E-06		-1.338	ACACA,APEX1,ATP2C1,CTSK,DDX3X,DNAJA1,FKBP4,GARS,GNA13,GNAI2,GNAS,HIST1H1C,HSD17B4,LIG1,NCSTN,NOTCH2,NRAS,PDS5A,PLAU,POLR2A,PTGS2,PTPRJ,RALA,RBPJ,RPSA,SLC20A1,SLC2A1,SNX2,SNX27,STAT1,TCIRG1,TFRC,TGFBR2,TKT,TTC37,USP8,YBX1	37
**Nucleic Acid Metabolism, Small Molecule Biochemistry**	Synthesis of nucleotide	1.9E-06		-0.988	ADSL,ADSSL1,ATP5F1B,C5AR1,CDK1,CTPS1,FASN,G6PD,GART,GMDS,GMPPB,GMPS,GNAS,GNB2,MTHFD1,NOS2,PFAS,PKM,PPAT,PTGS2,SHMT1,SNX5,TSTA3	23
**Cell-To-Cell Signaling and Interaction, Hematological System Development and Function,Inflammatory Response**	Aggregation of blood platelets	2E-06	Increased	2.33	AKR1B1,CD36,CD47,FCER1G,FERMT3,FYN,GNA13,GNAI2,GNAS,LYN,NOS2,PLA2G4A,PLEK,PTGS2,RAP1B,TLR7,VASP	17
**Molecular Transport**	Quantity of metal	2E-06		-0.158	AKR1B1,AP3B1,ATP2B1,ATP7A,C5AR1,EIF2S1,FCER1G,FYN,GNA13,GNAI2,GNAI3,GNAS,HLA-A,ICAM1,ITGAM,KCNN4,LILRB4,LYN,MEMO1,MYH9,NOS2,NPC1,PLAU,PTGS2,PTPRC,PTPRJ,STEAP3,STUB1,TFRC	29
**Cancer,Organismal Injury and Abnormalities,Reproductive System Disease**	Breast or ovarian cancer	2.1E-06			ABCA3,ACAA1,ACTR2,AKR1B1,ALCAM,AP3B1,ATIC,ATP1A1,ATP2C1,CAND1,CCT3,CCT5,CCT6A,CD36,CD47,CDK1,CLUH,CMAS,CNDP2,CNOT1,CSE1L,CTSD,CTSK,DDX17,DDX39B,DDX3X,DENND4B,DHX29,DKC1,DNAJA1,DNM1L,DNMT1,EEF1D,EIF3A,EIF3B,EIF3C,EIF3E,EIF3F,ENO1,FASN,FLNA,FLOT1,FYN,GART,GBA,GMDS,GNA13,GNAI3,GNAS,HIST1H1C,HLA-A,HNRNPM,HSP90AA1,HSP90AB1,IDH1,ITCH,JAK1,KPNA2,KRT1,KRT9,LGALS3BP,LIG1,MAPK3,MAT2B,MCM2,MOV10,MYH9,MYO1E,MYOF,NANS,NCF2,NCL,NOS2,NOTCH2,NPC1,NRAS,NUP93,NUS1,OLA1,PCNA,PFN1,PGK1,PI4K2A,PKM,PLAU,PLEC,PLXNA1,PNKP,POLD1,POLR1C,POLR2A,PPP2R1A,PRIM2,PTGS2,PTPRC,PTPRJ,RAB31,RAB5C,RANGAP1,RAP1B,RNF149,RNPEP,RPF2,RPL4,RPL6,RTCB,SF3B1,SFPQ,SHMT2,SLC16A3,SLC20A1,SLC23A2,SNRNP200,SNX27,STAT1,STEAP3,STOM,SUPT5H,TCP1,TGFBR2,TNFRSF1B,TPI1,TRIM25,TRPV2,TSG101,TTC37,UBA1,UBA2,UCHL5,VIM,VPS13C,VWA5A,WWP2,XPO1,XPO7	135

Table S4

**Hematological System Development and Function,Immunological Disease,Inflammatory Disease,Lymphoid Tissue Structure and Development,Organ Morphology,Organismal Development,Organismal Injury and Abnormalities,Tissue Morphology**	Enlargement of spleen	2.1E-06			CD47,FCER1G,FYN,GBA,IL6ST,ITGB2,JAK1,KCNN4,LIG1,LYN,MAPK3,MFGE8,NRAS,PTGS2,PTPRC,RIPK3,SIPA1,TLR7,TPP2	19
**Cancer,Hematological Disease,Immunological Disease,Organismal Injury and Abnormalities**	Mature B-cell lymphoma	2.1E-06			ADSL,CCT3,CD36,CDK1,DNMT1,DOCK2,FASN,G3BP1,GNA13,GNAI2,GRB2,HIST1H1C,HNRNPM,HSP90AA1,HSP90AB1,IDH1,JAK1,KPNA2,MYO1G,NOS2,NOTCH2,PCNA,POLD1,PPP2CA,PRIM2,PTGS2,PTPRC,RALA,SHMT2,STEAP3,TLR7,TNIP1,XPO1	33
**Hematological System Development and Function,Lymphoid Tissue Structure and Development,Organ Morphology,Tissue Morphology**	Morphology of lymphoid organ	2.1E-06			ABCG1,CD47,CTSD,DOCK2,FCER1G,FYN,GBA,GNAI2,HIST1H1C,HNRNPL,IL6ST,ITGB2,JAK1,KCNN4,LIG1,LYN,MAPK3,MFGE8,NOS2,NRAS,POLD1,PTGS2,PTPRC,PTPRJ,RIPK3,SIPA1,SNX27,STEAP3,TCIRG1,TLR7,TNFRSF1B,TPP2	32
**Cell-To-Cell Signaling and Interaction, Hematological System Development and Function**	Aggregation of blood cells	2.2E-06	Increased	2.701	AKR1B1,CD36,CD47,FCER1G,FERMT3,FYN,GNA13,GNAI2,GNAS,ICAM1,ITGB2,LYN,NOS2,PLA2G4A,PLEK,PTGS2,RAP1B,TLR7,VASP	19

Table S4

**Hematological System Development and Function,Hematopoiesis,Tissue Morphology**	Quantity of hematopoietic progenitor cells	2.2E-06		0.674	Abcb1b,CD36,CD47,CTSD,EEF1D,FCER1G,FLNA,FYN,GNAI2,IL6ST,Irgm1,ITGB2,JAK1,KIDINS220,LIG1,LYN,MAPK3,NOS2,PTPRC,RAP1B,RPS6,SIPA1,SIRPA,SLC20A1,STAM2,STEAP3,TCIRG1,TFRC,TPP2,VASP	30
**Cellular Movement,Hematological System Development and Function,Immune Cell Trafficking**	Cellular infiltration by leukocytes	2.2E-06		1.603	ABCG1,ADAM8,C5AR1,CD36,CD47,EDIL3,FCER1G,GBA,HLA-A,ICAM1,IL6ST,ITGAM,ITGB2,KRT10,MAPK3,NDRG1,NOS2,NPC1,PLA2G4A,PLAU,PLEC,PTGS2,STAT1,TCIRG1,TGFBR2,TNFRSF1B,TNIP1,YBX1	28
**Cell-To-Cell Signaling and Interaction,Inflammatory Response**	Response of macrophages	2.3E-06	Increased	2.196	ATP7A,CD36,CD47,DOCK2,FCER1G,FYN,IL6ST,ITGAM,ITGB2,LYN,MFGE8,NOS2,PTPRC,PTPRJ,SIRPA,TRPV2	16
**Cancer,Gastrointestinal Disease,Organismal Injury and Abnormalities**	Colorectal tumor	2.3E-06		0.221	ABCA3,ABCE1,ACLY,ACTA1,ADSL,ADSSL1,AHCY,AKR1B1,AKR1B10,ALCAM,AP3B1,APEX1,ARAP1,ATIC,ATP2B1,ATP2C1,ATP7A,CA2,CCT2,CCT4,CCT5,CCT7,CD36,CDK1,CFL1,CLUH,CMAS,CMIP,CNOT1,COLEC12,COPB1,CORO1C,CORO7/CORO7-PAM16,CPD,CPNE8,CRYZ,CSE1L,DCTN1,DDX17,DDX21,DDX39B,DENND4B,DHX15,DHX29,DIS3,DKC1,DNAJA1,DNAJC13,DNMT1,DOCK2,DPYSL2,EDIL3,EHD1,EIF2S3,EIF3C,EIF3F,EIF3I,EIF3L,EIF3M,ENO1,FASN,FLNA,FYN,G6PD,GALK1,GART,GMPS,GNA13,GNAI2,GNAS,GRB2,HIST1H1C,HK3,HLA-A,HNRNPL,HNRNPM,HSD17B4,HSP90AA1,HSP90AB1,HSPA4,ICAM1,IDE,IDH1,IFITM3,IL6ST,IPO5,ITGAM,ITGB2,JAK1,KCNN4,KIDINS220,KPNA2,KPNB1,KRT1,KRT2,LIG1,LILRB4,LRP12,LYN,MAPK3,MARS,MAT2A,MCM2,MCM7,MRI1,MTA2,MYH9,MYO1C,MYO1E,MYOF,NANS,NCL,NDRG1,NOTCH2,NRAS,NUP93,PCNA,PFAS,PGK1,PKM,PLA2G4A,PLAU,PLEC,PLXNB2,POLD1,POLR1C,PPP1CA,PPP2R1A,PREP,PTGS2,PTPN23,PTPRA,PTPRC,PTPRJ,RAB31,RAB5B,RCC2,RHBDF2,RNF213,RNH1,RNMT,RPL28,RPS6,RTCB,SF3B1,SIPA1,SIRPA,SLC15A3,SLC16A3,SLC20A1,SLC23A2,SLC29A1,SLC2A1,SLC38A2,SLC3A2,SLFN13,SMPDL3B,SND1,SNRNP200,SNX2,SPRED1,SQSTM1,SRM,STAT1,STEAP3,STRAP,STUB1,SUPT5H,TAX1BP1,TCIRG1,TCP1,TFRC,TGFBR2,TLR7,TMEM59,TPI1,TPP2,TRIM28,TRPV2,UBA1,VIM,VPS13C,WDR82,XPO1,XPO7	185
**Cellular Assembly and Organization**	Development of cytoplasm	2.3E-06		0.363	ACTR3,ARAP1,ARPC2,CD47,CFL1,CORO1C,CORO7/CORO7-PAM16,CTSD,DCTN1,DNM1L,DPYSL2,EIF2A,FKBP4,FLNA,FYN,GNA13,GRB2,ICAM1,IRGM,Irgm1,MYADM,MYO1C,PFN1,PTPRA,RAB7A,SIRPA,TGFBR2,VASP,XPO1	29
**Amino Acid Metabolism,Small Molecule Biochemistry,Vitamin and Mineral Metabolism**	Metabolism of L-tetrahydrofolic acid	2.4E-06			ATIC,GART,MTHFD1,SHMT1,SHMT2	5
**Cell-To-Cell Signaling and Interaction,Inflammatory Response**	Immune response of phagocytes	2.4E-06	Increased	2.283	CD36,CD47,DOCK2,FCER1G,FYN,ICAM1,IL6ST,ITGAM,ITGB2,LYN,MFGE8,NOS2,PLAU,PTPRC,PTPRJ,SIRPA,TLR7,TRPV2	18

Table S4

**Cellular Assembly and Organization**	Binding of zona pellucida	2.5E-06			CCT2,CCT3,CCT4,CCT5,CCT6A,CCT7,CCT8,TCP1	8
**Protein Synthesis**	Quantity of cytokine	2.5E-06	Decreased	-2.214	APEX1,C5AR1,CD36,GNAI2,GNAS,HELZ2,IL6ST,Irgm1,ITGB2,KRT1,LGALS3BP,LYN,MTA2,NOS2,PTGS2,RHBDF2,SQSTM1,STAT1,TNFRSF1B,WWP2	20
**Connective Tissue Disorders,Immunological Disease,Inflammatory Disease,Organismal Injury and Abnormalities,Skeletal and Muscular Disorders**	Lupus erythematosus	2.6E-06			DNMT1,GNAS,HLA-A,ICAM1,IL6ST,ITGAM,JAK1,LYN,MTA2,MYH9,NCF2,NOS2,OASL,PPAT,PPP1CA,PPP2CA,PTGS2,PTPRC,RAB31,RAB5A,RIPK3,STAT1,TLR7,TRIM25	24
**Cellular Movement**	Homing of cells	2.6E-06	Increased	2.214	ADAM8,C5AR1,CD36,CD47,CORO1B,DOCK2,FCER1G,FLOT1,FYN,GNA13,GNAI2,GNAI3,GNAS,GNB2,GRB2,ICAM1,ITGAM,ITGB2,JAK1,LYN,MAPK3,NUS1,PFN1,PLAU,PLEC,PTGS2,PTPRA,PTPRC,PTPRJ,RALA,RAP1B,SIRPA,TCIRG1,TRPV2	34
**Organismal Development**	Abnormal morphology of body cavity	2.6E-06			ABCA3,ACSL4,APEX1,CA2,CD36,CD47,CTSD,CTSK,DNM1L,DNMT1,DOCK2,EEF1D,FASN,FCER1G,FKBP4,FLNA,FYN,G6PD,GBA,GNA13,GNAI2,GNAS,GRB2,HIST1H1C,IL6ST,ITGB2,JAK1,KCNN4,KIDINS220,LIG1,LYN,MAPK3,MFGE8,MYH9,NCSTN,NOS2,NOTCH2,NPC1,NRAS,PFN1,PGK1,PLA2G4A,PLAU,PLEC,POLD1,PPP1CA,PPP2CA,PTGS2,PTPRC,PTPRJ,RBPJ,RIPK3,RPSA,SIPA1,SLC20A1,SNX27,STAT1,STEAP3,TAX1BP1,TCIRG1,TGFBR2,TLR7,TNFRSF1B,TPI1,TPP2,TRIM25,TSTA3,YBX1	68
**Cell-To-Cell Signaling and Interaction, Hematological System Development and Function,Immune Cell Trafficking**	Binding of antigen presenting cells	2.7E-06	Increased	2.242	ALCAM,ICAM1,Irgm1,ITGAM,ITGB2,LYN,MSN,PLAU,PTGS2,PTPRC,TRPV2	11
**Cell Signaling,Small Molecule Biochemistry**	Synthesis of nitric oxide	2.7E-06	Increased	2.001	CD36,CPD,DNM2,FASN,G6PD,GNAI3,HSP90AA1,ICAM1,ITGAM,ITGB2,JAK1,NOS2,PFN1,PLAU,PTGS2,RPSA,SIRPA,SLC7A1,STAT1,TGFBR2,TNFRSF1B	21
**Cancer,Organismal Injury and Abnormalities**	Breast or pancreatic cancer	2.8E-06		0	ABCA3,ACAA1,ACLY,ACTR2,ACTR3,ADSL,AHNAK,AKR1B1,ALCAM,AP3B1,ATIC,ATP1A1,ATP2B1,ATP2C1,ATP6V0A1,C5AR1,CAND1,CCT3,CCT5,CCT6A,CD36,CD47,CDK1,CLUH,CMAS,CNDP2,CNOT1,COLEC12,CSE1L,CTSD,CTSK,DAAM1,DCTN1,DDX17,DDX39B,DDX3X,DENND4B,DHX29,DKC1,DNAJA1,DNAJC13,DNM1L,DNMT1,DOCK2,EDIL3,EEF1D,EHD1,EIF2S3,EIF3A,EIF3B,EIF3C,EIF3E,EIF3F,ENO1,FASN,FLNA,FLOT1,FYN,GART,GBA,GMDS,GMPS,GNA13,GNAI3,GNAS,HIST1H1C,HK3,HLA-A,HNRNPL,HNRNPM,HNRNPU,HSP90AA1,HSP90AB1,IDH1,IPO5,ITCH,JAK1,KPNA2,KRT1,KRT10,KRT9,LGALS3BP,LIG1,LILRB4,LYN,MAPK3,MAT2B,MCM2,MOV10,MYADM,MYH9,MYO1E,MYO1G,MYOF,NANS,NCF2,NCL,NOP56,NOS2,NOTCH2,NPC1,NRAS,NUP93,NUS1,OLA1,PCNA,PFAS,PFN1,PGK1,PHGDH,PI4K2A,PKM,PLAU,PLEC,PLXNA1,PLXNB2,PNKP,POLD1,POLR1C,POLR2A,PPP2R1A,PPP2R2A,PRIM2,PTGS2,PTPRC,PTPRJ,RAB31,RAB5C,RANGAP1,RAP1B,RHBDF2,RIPK3,RNF149,RNF213,RNPEP,RPF2,RPL4,RPL6,RTCB,SCAMP2,SEC24B,SF3B1,SFPQ,SHMT2,SIPA1,SLC16A3,SLC20A1,SLC23A2,SLC29A1,SNRNP200,SNX27,STAT1,STEAP3,STOM,SUPT5H,TALDO1,TCP1,TGFBR2,TNFRSF1B,TPI1,TRIM25,TRPV2,TSG101,TTC37,TTLL12,UBA1,UBA2,UCHL5,VIM,VPS13C,VWA5A,WWP2,XPO1,XPO7	174

Table S4

**Organismal Development**	Morphology of body cavity	2.9E-06			ABCA3,ABCG1,ACSL4,APEX1,CA2,CD36,CD47,CTSD,CTSK,DNM1L,DNMT1,DOCK2,EEF1D,EIF3M,FASN,FCER1G,FKBP4,FLNA,FYN,G6PD,GBA,GNA13,GNAI2,GNAS,GRB2,HELZ2,HIST1H1C,HNRNPL,IL6ST,ITGB2,JAK1,KCNN4,KIDINS220,KRT10,LIG1,LYN,MAPK3,MFGE8,MYH9,NCSTN,NOS2,NOTCH2,NPC1,NRAS,PFN1,PGK1,PLA2G4A,PLAU,PLEC,POLD1,PPP1CA,PPP2CA,PTGS2,PTPRC,PTPRJ,RBPJ,RIPK3,RPSA,SIPA1,SLC20A1,SNX27,STAT1,STEAP3,TAX1BP1,TCIRG1,TGFBR2,TKT,TLR7,TNFRSF1B,TPI1,TPP2,TRIM25,TSTA3,YBX1	74
**Cellular Function and Maintenance,Hematological System Development and Function**	Function of myeloid cells	2.9E-06			CD36,FCER1G,FYN,GNAI2,ICAM1,ITGAM,LYN,NOS2,PLA2G4A,PLAU,PTGS2,RHBDF2,RIPK3,SIRPA,SPRED1,STAT1,TCIRG1	17
**Cellular Function and Maintenance**	Function of antigen presenting cells	2.9E-06			CD36,FCER1G,FYN,GPNMB,HSP90AA1,ICAM1,ITGAM,LYN,NOS2,PLA2G4A,PLAU,PLXNA1,PTGS2,RHBDF2,RIPK3,SIRPA,STAT1,TCIRG1,TLR7	19
**Lipid Metabolism, Small Molecule Biochemistry**	Incorporation of fatty acid	3E-06		0.835	ABCA3,ACLY,ACSL4,CD36,NPC1,PLA2G4A	6
**Cellular Movement**	Invasion of tumor cell lines	3.1E-06		1.494	AHNAK,ALCAM,ATP6V0A1,CSE1L,CTSK,DNM1L,DPYSL2,EIF3E,FERMT3,GNA13,GNAI3,IFITM3,IPO7,LYN,MAPK3,NAA15,NDRG1,NOS2,PKM,PLAU,PTGS2,PTPRA,RAB5A,RALA,SDCBP,SIPA1,SLC12A4,SLC2A1,SNAP23,SQSTM1,STAT1,TCIRG1,TGFBR2,VIM	34
**Cell-To-Cell Signaling and Interaction**	Adhesion of lymphatic system cells	3.1E-06		1.286	ALCAM,CD47,FERMT3,FLOT1,GNAI2,ICAM1,IL6ST,ITGB2,JAK1,MYADM,MYO1G,PLAU,RAP1B	13
**Gene Expression**	Initiation of expression of RNA	3.1E-06			DDX3X,EIF2S3,EIF3B,EIF3C,EIF3D,EIF3E,EIF3F,EIF3I,EIF3L,MAPK3,NCBP1,NOTCH2,POLR1C,POLR2A,POLR2B,RBPJ,TRIM28	17
**Cellular Function and Maintenance**	Homeostasis of blood cells	3.3E-06	Increased	2.751	ABCG1,AP3B1,ATP7A,C5AR1,DNMT1,DOCK2,EEF1D,FCER1G,FYN,GRB2,HLA-A,HNRNPL,HSP90AA1,ICAM1,IL6ST,ITCH,ITGB2,JAK1,LILRB4,LYN,MAPK3,MTHFD1,NCSTN,NOS2,NOTCH2,PTGS2,PTPRC,RBPJ,RIPK3,RPS6,SLC3A2,STAT1,TGFBR2,TLR7,TNFRSF1B,TPP2	36
**Cell Morphology, Cellular Movement,Hematological System Development and Function,Immune Cell Trafficking**	Cell spreading of leukocytes	3.4E-06		0.896	CD47,FERMT3,ICAM1,ITGAM,ITGB2,LYN,PTPRA,RAP1B,SIRPA	9
**Cancer,Hematological Disease,Immunological Disease,Organismal Injury and Abnormalities**	Non-Hodgkin lymphoma	3.4E-06			ABCG1,ADSL,AHNAK,CCT3,CD36,CD47,CDK1,CNOT1,CSE1L,DDX3X,DNMT1,DOCK2,EIF2A,FASN,FYN,G3BP1,GNA13,GNAI2,GRB2,HIST1H1C,HNRNPM,HNRNPU,HSP90AA1,HSP90AB1,ICAM1,IDH1,JAK1,KPNA2,KRT2,MAPK3,MYO1G,MYOF,NOS2,NOTCH2,NRAS,OASL,PCNA,POLD1,PPAT,PPP2CA,PRIM2,PTGS2,PTPRC,RALA,RNF213,RPSA,SARS,SF3B1,SHMT1,SHMT2,STAT1,STEAP3,TLR7,TNIP1,TRIM25,USP8,XPO1	57

Table S4

**Cancer,Gastrointestinal Disease,Hepatic System Disease,Organismal Injury and Abnormalities**	Liver carcinoma	3.4E-06			ABCA3,ABCG1,ACAA1,ACLY,ACO2,ACTR1A,ACTR2,ACTR3,AHNAK,AKR1B10,ALCAM,ALDH9A1,APEX1,ARAP1,ATIC,ATP1A1,ATP1B3,ATP6V0A1,ATP7A,CA2,CCT6A,CCT8,CD36,CD47,CNDP2,CNOT1,COPG1,CPNE8,CSE1L,CTPS1,CTSD,DAAM1,DDX17,DDX39B,DIS3,DKC1,DNAJA1,DNAJC13,DNM1L,DOCK2,EDIL3,EIF3A,EIF3E,EIF3I,ENO1,FARSA,FASN,FERMT3,FLNA,G6PD,GALK1,GARS,GMPS,GNA13,GNAS,GRB2,HNRNPM,HSP90AA1,HSP90AB1,HSPA4,ICAM1,IDE,IDH1,IFITM3,IL6ST,IPO5,IPO7,ITGAM,JAK1,KIDINS220,KPNB1,KRT10,KRT9,MARS,MAT2B,MCM2,MCM7,MDH1,MSTO1,MTA2,MTHFD1,MVP,MYADM,MYO1C,MYO1E,MYOF,NAA15,NCBP1,NCL,NOP56,NOS2,NPC1,NRAS,OASL,PDS5A,PFAS,PFN1,PKM,PLA2G4A,PLAU,PLD3,PLEC,PLXNA1,POLD1,POLR2B,PPP2CA,PPP2R1A,PRIM2,PRPF4,PTGS2,PTPRA,PTPRC,RAB8B,RACK1,RALA,RBPJ,RNF213,RNMT,RNPEP,RPL6,RPL7A,RPN1,RPSA,RTCB,SARS,SCFD1,SEC24B,SF3B1,SFPQ,SLC16A6,SLC1A5,SLC29A1,SLC38A2,SLC4A7,SLC7A1,SND1,SNRNP200,SNX2,SNX27,SRM,STAM2,STAT1,SUPT5H,TAX1BP1,TCIRG1,TGFBR2,TLR7,TNFRSF1B,TNIP1,TPI1,TPP2,TRIM25,TTC37,UBA2,UCHL5,VIM,VPS13C,WARS,XPNPEP1	159
**Cell-To-Cell Signaling and Interaction, Hematological System Development and Function,Immune Cell Trafficking**	Adhesion of antigen presenting cells	3.5E-06		1.298	ALCAM,ICAM1,Irgm1,ITGAM,ITGB2,LYN,PLAU,PTGS2	8
**Cell-To-Cell Signaling and Interaction, Cellular Function and Maintenance,Inflammatory Response**	Phagocytosis of leukocytes	3.5E-06		1.955	CD36,CD47,DOCK2,FCER1G,FYN,ICAM1,ITGAM,ITGB2,MFGE8,MYO1G,PLAU,PTPRC,PTPRJ,SIRPA,TRPV2	15
**RNA Post-Transcriptional Modification**	Processing of rRNA	3.6E-06			DIS3,DKC1,MTREX,NOP56,RPL14,RPL26,RPL7,RPS14,RPS6,RPS9,RRP9	11
**Cellular Movement,Hematological System Development and Function,Immune Cell Trafficking**	Cell movement of granulocytes	3.7E-06	Increased	2.172	ADAM8,C5AR1,CD36,CD47,DOCK2,EDIL3,FCER1G,FERMT3,FLOT1,GNAI2,GNAI3,ICAM1,IL6ST,ITGAM,ITGB2,LYN,NOS2,PLAU,PTGS2,RAP1B,SIRPA,TGFBR2,TNFRSF1B,TNIP1,VASP,YBX1	26
**Cellular Assembly and Organization**	Organization of cellular membrane	3.7E-06			ACTR2,ACTR3,ARPC2,CFL1,CORO1B,DNM2,GRB2,MYH9,NUP93,PLEK,RAB5A,RAB8B,RALA,RPS27A,STAM2,TFRC,VAMP8	17
**Cellular Assembly and Organization,Cellular Function and Maintenance**	Organization of endosomes	3.7E-06			DNAJC13,RAB5A,RAB5B,RAB5C,SQSTM1,STX6,USP8	7

Table S4

**Cell-To-Cell Signaling and Interaction,Inflammatory Response**	Immune response of macrophages	3.7E-06	Increased	2.028	CD36,CD47,DOCK2,FCER1G,FYN,IL6ST,ITGAM,ITGB2,LYN,MFGE8,NOS2,PTPRC,PTPRJ,SIRPA,TRPV2	15
**Cellular Function and Maintenance,Molecular Transport**	Exocytosis by mast cells	3.9E-06			GNAI2,GNAI3,SCAMP2,VAMP8	4
**Cancer,Organismal Injury and Abnormalities,Respiratory Disease**	Sarcomatoid malignant pleural mesothelioma	3.9E-06			ATIC,GART,HSP90AA1,HSP90AB1	4
**Cancer,Organismal Injury and Abnormalities,Respiratory Disease**	Stage III ALK fusion negative EGFR mutation negative non-small cell lung carcinoma	3.9E-06			ATIC,GART,HSP90AA1,HSP90AB1	4
**Cancer,Organismal Injury and Abnormalities,Respiratory Disease**	Stage IIIA lung adenocarcinoma	3.9E-06			ATIC,GART,HSP90AA1,HSP90AB1	4
**Cancer,Organismal Injury and Abnormalities,Reproductive System Disease**	Tumorigenesis of reproductive tract	4E-06			ABCA3,ABCE1,ACSL4,ACTR2,ACTR3,ADSL,AHNAK,AKR1B1,ALCAM,AP3B1,ATIC,ATP2B1,ATP2C1,ATP7A,CA2,CAND1,CCT5,CCT6A,CD36,CD47,CDK1,CLUH,CNOT1,COLEC12,COPB1,CORO1C,CSE1L,CTPS1,DCTN1,DDX3X,DIS3,DNAJA1,DNAJC13,DNMT1,ECPAS,EEF1D,EIF2S1,EIF3A,EIF3D,EIF3E,EIF3F,ENO1,FLNA,FLOT1,GART,GFPT1,GMDS,GNA13,GNAI2,GNAS,GNB2,GRB2,HIST1H1C,HIST1H2AJ,HLA-A,HNRNPL,HNRNPM,HNRNPU,HSD17B4,HSP90AA1,HSP90AB1,IDH1,IPO5,ITCH,ITGAM,JAK1,KIDINS220,KPNA2,KRT1,KRT9,LIG1,LILRB4,LYN,MAPK3,MAT2B,MCM2,MCM7,MDH1,MEMO1,MFGE8,MSN,MYH9,MYO1C,MYO1E,MYOF,NAA15,NCBP1,NDRG1,NOP56,NOS2,NOTCH2,NPC1,NRAS,NUP93,OASL,OLA1,PCNA,PDXK,PFAS,PKM,PLAU,PLD3,PLEC,PLXNA1,PLXNB2,PNKP,POLR1C,PPP1R7,PPP2CA,PPP2R1A,PRIM2,PTGS2,PTPRA,PTPRJ,RALA,RBBP7,RIPK3,RNF149,RNPEP,RPF2,RPL14,RPS6,SCFD1,SF3B1,SFPQ,SHMT2,SIRPA,SLC15A3,SLC16A3,SLC20A1,SLC38A2,SLC7A1,SMPDL3B,SND1,SNRNP200,SNX27,STAT1,STOM,SUPT5H,TALDO1,TFRC,TGFBR2,TLR7,TMEM59,TNIP1,TPI1,TPP2,TRIM25,TSG101,TSR1,TTC37,TTLL12,UBA1,UBA2,USP5,USP8,VAMP8,VIM,VPS13C,WWP2,XPO1,XPO7	162
**Cancer,Hematological Disease,Immunological Disease,Organismal Injury and Abnormalities**	High grade lymphocytic cancer	4.1E-06			ADSL,CCT3,CD36,DNMT1,DOCK2,FYN,GNA13,GNAI2,GRB2,HIST1H1C,HNRNPM,HSP90AA1,HSP90AB1,ICAM1,IDH1,JAK1,MYO1G,NOTCH2,POLD1,PPAT,PRIM2,PTPRC,SHMT2,STEAP3,TNIP1,XPO1	26
**Cellular Function and Maintenance**	Function of phagocytes	4.2E-06			CD36,FCER1G,FYN,GPNMB,HSP90AA1,ICAM1,ITGAM,LILRB4,LYN,NOS2,PLA2G4A,PLAU,PLXNA1,PTGS2,RHBDF2,RIPK3,SIRPA,STAT1,TCIRG1,TLR7	20

Table S4

**Cancer,Organismal Injury and Abnormalities**	Genitourinary carcinoma	4.3E-06			ABCA3,ABCE1,ACLY,ACSL4,ACTR1A,ACTR2,ADAM8,ADSL,ADSSL1,AHNAK,AKR1B1,AP3B1,APEX1,ARPC2,ATIC,ATP1A1,ATP2B1,ATP2C1,ATP7A,CA2,CAND1,CAPZA1,CCT2,CCT3,CCT5,CCT6A,CD36,CD47,CLUH,CMIP,CNOT1,COLEC12,COPB1,CORO1C,CPD,CPNE8,CSE1L,CTPS1,CTSD,CTSK,DCTN1,DDX17,DDX21,DDX3X,DENND4B,DIS3,DNAJA1,DNAJC13,DNM1L,DNMT1,DOCK2,ECPAS,EDIL3,EEF1D,EEF1G,EIF2S1,EIF3A,EIF3D,EIF3E,EIF3F,EIF3I,ESYT1,FASN,FERMT3,FLNA,FLOT1,G3BP1,G6PD,GART,GFPT1,GMDS,GNA13,GNAI2,GNAS,GNB2,GPNMB,GRB2,GUSB,HIST1H1C,HIST1H2AJ,HK3,HLA-A,HNRNPL,HNRNPM,HNRNPU,HSD17B4,HSP90AA1,HSP90AB1,HSPA4,IDH1,IPO5,ITCH,ITGAM,ITGB2,JAK1,KIDINS220,KPNA2,KRT1,KRT10,KRT2,KRT9,LIG1,LILRB4,LRP12,MAT2B,MCM2,MCM7,MDH1,MEMO1,MFGE8,MSN,MSTO1,MVP,MYADM,MYH9,MYO1C,MYO1E,MYO1G,MYOF,NAA15,NCBP1,NCSTN,NOP56,NOS2,NOTCH2,NPC1,NRAS,NUP93,OASL,OLA1,PCNA,PDS5A,PDXK,PFAS,PI4K2A,PKM,PLD3,PLEC,PLEK,PLXNA1,PLXNB2,PNKP,POLD1,POLR1C,POLR2B,PPP1R7,PPP2R1A,PREP,PRIM2,PTGS2,PTPN23,PTPRA,PTPRC,PTPRJ,RAB5B,RALA,RANGAP1,RAP2C,RARS,RBBP7,RCC2,RIPK3,RNF149,RNF213,RNPEP,RPF2,RPL14,RPL17,RPS27A,RRP9,SCFD1,SEC24B,SF3B1,SFPQ,SIRPA,SLC12A4,SLC15A3,SLC16A3,SLC20A1,SLC29A1,SLC2A1,SLC38A2,SLC3A2,SLC7A1,SLFN13,SMPDL3B,SND1,SNRNP200,SNX27,STAT1,STEAP3,STOM,STX6,SUPT5H,TALDO1,TGFBR2,TKT,TLR7,TNIP1,TOM1,TPI1,TPP2,TRIM25,TRIM28,TRPV2,TSG101,TSR1,TTC37,TTLL12,UBA1,UBA2,USP5,USP8,VAMP8,VIM,VPS13C,VWA5A,WWP2,XPO1,XPO7,YBX1	221
**Cell-To-Cell Signaling and Interaction, Hematological System Development and Function,Immune Cell Trafficking**	Adhesion of mononuclear leukocytes	4.4E-06		1.32	CD47,FERMT3,FLOT1,GNAI2,ICAM1,IL6ST,ITGAM,ITGB2,JAK1,MYADM,MYO1G,PLAU,RAP1B,TGFBR2	14
**Cell-To-Cell Signaling and Interaction, Cellular Assembly and Organization**	Quantity of actin stress fibers	4.5E-06		1	ARAP1,CFL1,DNM2,FYN,NRAS,PLAU,PLEC	7
**Cancer,Organismal Injury and Abnormalities,Reproductive System Disease**	Female genital neoplasm	4.5E-06			ABCA3,ABCE1,ACSL4,ACTR2,ACTR3,ADSL,AHNAK,AKR1B1,ALCAM,AP3B1,ATIC,ATP2B1,ATP2C1,ATP7A,CA2,CAND1,CCT5,CCT6A,CD36,CD47,CDK1,CLUH,CNOT1,COLEC12,COPB1,CORO1C,CSE1L,CTPS1,DCTN1,DDX3X,DIS3,DNAJA1,DNAJC13,DNMT1,ECPAS,EEF1D,EIF2S1,EIF3A,EIF3D,EIF3E,EIF3F,ENO1,FLNA,FLOT1,GART,GFPT1,GMDS,GNA13,GNAI2,GNAS,GNB2,GRB2,HIST1H1C,HIST1H2AJ,HLA-A,HNRNPL,HNRNPM,HNRNPU,HSD17B4,HSP90AA1,HSP90AB1,IDH1,IPO5,ITCH,ITGAM,JAK1,KIDINS220,KPNA2,KRT1,KRT9,LIG1,LILRB4,LYN,MAPK3,MAT2B,MCM2,MCM7,MDH1,MEMO1,MFGE8,MSN,MYH9,MYO1C,MYO1E,MYOF,NAA15,NCBP1,NDRG1,NOP56,NOS2,NOTCH2,NPC1,NRAS,NUP93,OASL,OLA1,PCNA,PDXK,PFAS,PKM,PLAU,PLD3,PLEC,PLXNA1,PLXNB2,PNKP,POLR1C,PPP1R7,PPP2CA,PPP2R1A,PRIM2,PTGS2,PTPRA,PTPRJ,RALA,RBBP7,RIPK3,RNF149,RNPEP,RPF2,RPL14,RPS6,SCFD1,SF3B1,SFPQ,SHMT2,SIRPA,SLC15A3,SLC16A3,SLC20A1,SLC38A2,SLC7A1,SMPDL3B,SND1,SNRNP200,SNX27,STAT1,STOM,SUPT5H,TALDO1,TFRC,TGFBR2,TLR7,TMEM59,TNIP1,TPI1,TPP2,TRIM25,TSG101,TSR1,TTC37,TTLL12,UBA1,UBA2,USP5,USP8,VAMP8,VIM,VPS13C,WWP2,XPO1,XPO7	162
**Cancer,Hematological Disease,Organismal Injury and Abnormalities**	Refractory hematologic cancer	4.8E-06			DNMT1,FYN,HSP90AA1,HSP90AB1,IDH1,JAK1,LYN,NRAS,POLD1,PPAT,PRIM2,PTGS2,RPL3,RPL6,SF3B1	15

Table S4

**Hematological System Development and Function,Lymphoid Tissue Structure and Development,Organ Morphology,Organismal Development,Tissue Morphology**	Morphology of spleen	4.8E-06			CD47,CTSD,DOCK2,FCER1G,FYN,GBA,GNAI2,IL6ST,ITGB2,JAK1,KCNN4,LIG1,LYN,MAPK3,MFGE8,NRAS,PTGS2,PTPRC,PTPRJ,RIPK3,SIPA1,SNX27,STEAP3,TCIRG1,TLR7,TPP2	26
**Hematological System Development and Function,Tissue Development**	Accumulation of myeloid cells	4.9E-06		0	ABCG1,C5AR1,EDIL3,GNAI2,IL6ST,ITGAM,ITGB2,MAPK3,NCSTN,NOS2,NPC1,PFN1,PLAU,PTGS2,TLR7,TNFRSF1B	16
**Hereditary Disorder,Neurological Disease,Organismal Injury and Abnormalities,Skeletal and Muscular Disorders**	Distal spinal muscular atrophy	5.1E-06			ATP7A,DCTN1,GARS,MARS,UBA1,WARS	6
**Cancer,Hematological Disease,Organismal Injury and Abnormalities,Tumor Morphology**	Progressive malignant lymphoid neoplasm	5.1E-06			ATIC,GART,HSP90AA1,HSP90AB1,POLD1,SF3B1	6
**Cancer,Hematological Disease,Organismal Injury and Abnormalities**	Myelodysplastic syndrome	5.3E-06			CD36,CDK1,DNMT1,GNAS,IDH1,MYH9,NCSTN,NOTCH2,NRAS,POLD1,PPAT,PRIM2,RPL13,RPL28,RPL3,RPL4,RPL6,RPL7,RPS14,RPS27A,RPS6,SF3B1,XPO1	23

Table S4

**Cell-mediated Immune Response,Cellular Development,Cellular Function and Maintenance,Cellular Growth and Proliferation,Embryonic Development,Hematological System Development and Function,Hematopoiesis,Lymphoid Tissue Structure and Development,Organ Development,Organismal Development,Tissue Development**	T cell development	5.3E-06	Increased	2.744	ABCG1,AP3B1,ATP7A,C5AR1,DNMT1,DOCK2,EEF1D,FCER1G,FYN,GRB2,HLA-A,HNRNPL,HSP90AA1,ICAM1,IL6ST,ITCH,ITGB2,JAK1,LILRB4,MAPK3,NOS2,NOTCH2,PTGS2,PTPRC,RBPJ,RIPK3,RPS6,SLC3A2,STAT1,TGFBR2,TLR7,TNFRSF1B,TPP2	33
**Nucleic Acid Metabolism,Small Molecule Biochemistry**	Synthesis of ribonucleoside monophosphate	5.4E-06			ADSL,ADSSL1,GART,GMPS,PFAS	5
**Cancer,Hematological Disease,Immunological Disease,Neurological Disease,Organismal Injury and Abnormalities,Tumor Morphology**	Progressive primary central nervous system lymphoma	5.4E-06			ATIC,GART,HSP90AA1,HSP90AB1,POLD1	5

Table S4

**Cancer,Hematological Disease,Immunological Disease,Neurological Disease,Organismal Injury and Abnormalities**	Recurrent primary central nervous system lymphoma	5.4E-06			ATIC,GART,HSP90AA1,HSP90AB1,POLD1	5
**Cell Morphology,Cellular Assembly and Organization,Cellular Function and Maintenance**	Reorganization of actin cytoskeleton	5.5E-06		0	ARAP1,ATP2C1,CD47,CFL1,DPYSL2,FLNA,ICAM1,MYH9,PLAU,PLEK,RALA,RPS6	12
**Cancer,Organismal Injury and Abnormalities**	Advanced malignant solid tumor	5.5E-06		1.706	ATIC,C5AR1,CD36,COLEC12,CRYZ,CSE1L,DPYSL2,FASN,FLNA,FYN,GART,GNAS,GPNMB,HIST1H1C,HSP90AA1,HSP90AB1,ICAM1,JAK1,KIDINS220,LYN,MYO1C,NRAS,NUP93,PLAU,PLEC,POLD1,PPP2R1A,PRIM2,PTGS2,PTPRC,PTPRJ,RAB31,RNH1,RPL7,RPS27A,RPS6,SLC16A3,SQSTM1,STAT1,STEAP3,TGFBR2,XPO1	42
**Cellular Function and Maintenance**	Homeostasis of leukocytes	5.7E-06	Increased	2.751	ABCG1,AP3B1,ATP7A,C5AR1,DNMT1,DOCK2,EEF1D,FCER1G,FYN,GRB2,HLA-A,HNRNPL,HSP90AA1,ICAM1,IL6ST,ITCH,ITGB2,JAK1,LILRB4,LYN,MAPK3,MTHFD1,NOS2,NOTCH2,PTGS2,PTPRC,RBPJ,RIPK3,RPS6,SLC3A2,STAT1,TGFBR2,TLR7,TNFRSF1B,TPP2	35
**Hematological System Development and Function,Lymphoid Tissue Structure and Development,Tissue Morphology**	Morphology of lymphoid tissue	5.8E-06			ABCG1,CD47,CTSD,DOCK2,FCER1G,FYN,GBA,GNA13,GNAI2,HIST1H1C,HNRNPL,IL6ST,ITGB2,JAK1,KCNN4,LIG1,LYN,MAPK3,MFGE8,NOS2,NRAS,POLD1,PTGS2,PTPRC,PTPRJ,RIPK3,SIPA1,SNX27,STEAP3,TCIRG1,TLR7,TNFRSF1B,TPP2	33
**Cancer,Organismal Injury and Abnormalities**	Neoplasia of cells	5.8E-06		-0.309	ABCG1,ACO2,ACSL4,ADSL,AHNAK,ALCAM,ALDH9A1,AMDHD2,ARAP1,ATIC,ATP1A1,ATP2C1,ATP7A,CA2,CCT3,CCT7,CD36,CD47,CDK1,CFL1,CMIP,CNOT1,CORO1B,CPD,CSE1L,CTPS1,CTSK,DAAM1,DDX3X,DENND4B,DHX15,DIS3,DNAJC13,DNM1L,DNMT1,DOCK2,EDIL3,EEF1G,EIF2A,EIF2S3,EIF3A,EIF3B,FASN,FLNA,FYN,G3BP1,GART,GNA13,GNAI2,GNAS,GPNMB,GRB2,HIST1H1C,HIST1H2AJ,HLA-A,HNRNPM,HNRNPU,HSP90AA1,HSP90AB1,ICAM1,IDE,IDH1,Ifi202b,IL6ST,IPO5,IPO7,ITCH,ITGAM,ITGB2,JAK1,KIDINS220,KPNA2,KPNB1,KRT1,KRT10,KRT2,KRT9,LILRB4,LYN,MAPK3,MARS,MCM2,MPEG1,MSN,MTA2,MYH9,MYO1C,MYO1E,MYO1G,MYOF,NCL,NCSTN,NOS2,NOTCH2,NPC1,NRAS,NUS1,OASL,OLA1,PCNA,PKM,PLAU,PLEC,PLXNB2,POLD1,PPAT,PPP1CA,PPP2CA,PPP2R1A,PREP,PRIM2,PRPF19,PTGS2,PTPRA,PTPRC,RALA,RAN,RHBDF2,RIPK3,RNF213,RNH1,RPL10,RPL13,RPL14,RPL4,RPS6,RPSA,SARS,SDCBP,SF3B1,SFPQ,SHMT1,SHMT2,SIRPA,SLC23A2,SLC2A1,SMPDL3B,SND1,SNRNP200,SNX2,SNX27,SPRED1,STAT1,STEAP3,STRAP,TAX1BP1,TCIRG1,TCP1,TFRC,TGFBR2,TLR7,TMEM59,TNIP1,TPP2,TRIM14,TRIM25,UBA2,USP8,VIM,WWP2,XPNPEP1,XPO1	162

Table S4

**Cellular Development,Cellular Growth and Proliferation,Hematological System Development and Function,Hematopoiesis,Lymphoid Tissue Structure and Development,Tissue Development**	Hematopoiesis of mononuclear leukocytes	5.9E-06	Increased	2.351	ABCG1,AP3B1,ATP7A,C5AR1,CD36,DNMT1,DOCK2,EEF1D,FASN,FCER1G,FYN,GRB2,HLA-A,HNRNPL,HSP90AA1,ICAM1,IL6ST,ITCH,ITGAM,ITGB2,JAK1,LILRB4,LYN,MAPK3,MFGE8,MSN,NOS2,NOTCH2,PTGS2,PTPRC,PTPRJ,RBPJ,RIPK3,RPS6,SLC3A2,STAT1,TGFBR2,TLR7,TNFRSF1B,TPP2	40
**Cellular Movement,Hematological System Development and Function,Immune Cell Trafficking,Inflammatory Response**	Cell movement of macrophages	5.9E-06		1.411	C5AR1,FLNA,GBA,GNAI3,ICAM1,Irgm1,ITGB2,MAPK3,NDRG1,NOS2,PFN1,PLA2G4A,PLAU,PLEC,PTGS2,PTPRJ,SIRPA,STAT1,TGFBR2,TRPV2,YBX1	21
**Carbohydrate Metabolism, Drug Metabolism, Molecular Transport,Small Molecule Biochemistry**	Efflux of doxorubicin	6.3E-06			ATP7A,MVP,PTGS2	3
**Immune Cell Trafficking,Inflammatory Response**	Margination of neutrophils	6.3E-06			ICAM1,ITGAM,ITGB2	3
**Cancer,Organismal Injury and Abnormalities**	Transitional-cell carcinoma	6.4E-06			ATIC,FERMT3,GART,GNA13,HIST1H1C,HSP90AA1,HSP90AB1,IDH1,JAK1,MCM2,NCSTN,NOS2,NOTCH2,NRAS,NUP93,PI4K2A,PKM,PPP2R1A,PTGS2,SF3B1,TGFBR2,VIM	22
**Cell Morphology, Cellular Movement**	Cell spreading	6.9E-06		0.205	ARAP1,CD36,CD47,FERMT3,FLNA,ICAM1,ITGAM,ITGB2,LYN,MAPK3,PLAU,PTPRA,RAP1B,SIRPA,STX6,VASP,VIM	17
**Hematological System Development and Function**	Hemostasis	7.2E-06		1.896	AP3B1,CAPZA1,CD36,EHD1,FCER1G,FERMT3,FLNA,FYN,GNA13,GNAI2,ICAM1,ITGAM,LYN,MAPK3,MFGE8,NOS2,NPC1,PLAU,PLEK,PTGS2,PTPRJ,RAB5A,VASP	23

Table S4

**Cellular Function and Maintenance,Hematological System Development and Function**	Function of macrophages	7.3E-06			CD36,FCER1G,FYN,ICAM1,ITGAM,LYN,NOS2,PLA2G4A,PLAU,PTGS2,RHBDF2,RIPK3,SIRPA,STAT1,TCIRG1	15
**Cancer,Hematological Disease,Immunological Disease,Organismal Injury and Abnormalities**	T-lymphoblastic leukemia/lymphoma	7.3E-06			DNM2,FYN,HSP90AA1,HSP90AB1,IDH1,ITGAM,JAK1,NRAS,POLD1,PPAT,PTPRC,RPL10,TAX1BP1,TLR7,TSR1	15
**Connective Tissue Disorders,Hereditary Disorder,Organismal Injury and Abnormalities**	Hereditary connective tissue disorder	7.3E-06			ABCA3,ATP7A,CA2,CORO7/CORO7-PAM16,CTSK,DNM2,EIF2S3,FCER1G,FERMT3,FLNA,GNAI3,GNAS,IDH1,ITGAM,MARS,MTHFD1,MYH9,NANS,NCF2,NOTCH2,NRAS,OSGEP,PLEC,PNKP,POLD1,POLR1C,PRIM2,PTGS2,RPS26,SARS,SQSTM1,TCIRG1,TGFBR2,TTC37,UBA1	35
**Cancer,Hematological Disease,Immunological Disease,Organismal Injury and Abnormalities**	High-grade lymphoma	7.5E-06			ADSL,CCT3,CD36,DNMT1,DOCK2,FYN,GNA13,GNAI2,GRB2,HIST1H1C,HNRNPM,HSP90AA1,HSP90AB1,ICAM1,IDH1,JAK1,MYO1G,NOTCH2,POLD1,PPAT,PRIM2,PTPRC,SHMT2,STEAP3,TNIP1,XPO1	26
**Cancer,Organismal Injury and Abnormalities,Respiratory Disease**	Stage IV mixed cell non-small cell lung carcinoma	7.6E-06			ATIC,GART,HSP90AA1,HSP90AB1	4
**Cancer,Organismal Injury and Abnormalities,Respiratory Disease**	EGFR L858R mutation positive EGFR T790M mutation positive non-squamous non-small cell lung cancer	7.6E-06			ATIC,GART,HSP90AA1,HSP90AB1	4
**Cancer,Organismal Injury and Abnormalities,Respiratory Disease**	Stage IIIB locally advanced lung adenocarcinoma	7.6E-06			ATIC,GART,HSP90AA1,HSP90AB1	4

Table S4

**Cancer,Organismal Injury and Abnormalities,Respiratory Disease**	Stage IV EGFR exon 19 deletion positive lung adenocarcinoma	7.6E-06			ATIC,GART,HSP90AA1,HSP90AB1	4
**Cancer,Organismal Injury and Abnormalities,Respiratory Disease**	Stage IIIB EGFR exon 21 L858R activating mutation positive lung adenocarcinoma	7.6E-06			ATIC,GART,HSP90AA1,HSP90AB1	4
**Cancer,Organismal Injury and Abnormalities,Respiratory Disease**	Stage IIIB EGFR exon 19 deletion positive lung adenocarcinoma	7.6E-06			ATIC,GART,HSP90AA1,HSP90AB1	4
**Cancer,Organismal Injury and Abnormalities,Respiratory Disease**	EGFR exon 19 deletion positive EGFR T790M mutation positive non-squamous non-small cell lung cancer	7.6E-06			ATIC,GART,HSP90AA1,HSP90AB1	4
**Cancer,Organismal Injury and Abnormalities,Respiratory Disease**	EGFR exon 19 deletion positive EGFR T790M mutation negative non-squamous non-small cell lung carcinoma	7.6E-06			ATIC,GART,HSP90AA1,HSP90AB1	4
**Cancer,Organismal Injury and Abnormalities,Respiratory Disease**	EGFR exon 19 deletion positive EGFR T790M mutation negative lung adenocarcinoma	7.6E-06			ATIC,GART,HSP90AA1,HSP90AB1	4
**Cancer,Organismal Injury and Abnormalities,Respiratory Disease**	Stage IV EGFR exon 21 L858R activating mutation positive lung adenocarcinoma	7.6E-06			ATIC,GART,HSP90AA1,HSP90AB1	4
**Cancer,Hematological Disease,Organismal Injury and Abnormalities**	Chronic myeloproliferative neoplasm	7.7E-06			CDK1,DAAM1,DNMT1,FCER1G,FYN,IDH1,JAK1,LYN,NCSTN,NOTCH2,NRAS,POLD1,PPAT,PRIM2,PTGS2,RPL3,SF3B1,SIPA1	18

Table S4

**Hereditary Disorder,Neurological Disease,Organismal Injury and Abnormalities**	Hereditary motor neuropathy	8E-06			ATP1A1,ATP7A,CCT5,DCTN1,DNM2,GARS,KIDINS220,MARS,NDRG1,RAB7A,UBA1,USP8,WARS	13
**Cellular Assembly and Organization**	Regulation of mitochondria	8.2E-06			FYN,GNA13,KPNB1,PPP2CA,PPP2R1A,RAN	6
**Inflammatory Response**	Inflammation of absolute anatomical region	8.4E-06		-1.124	ABCA3,ABCG1,ACO2,ALCAM,C5AR1,CA2,CD36,CD47,CTSD,ENO1,FASN,FCER1G,GNAI2,HLA-A,ICAM1,IDE,IL6ST,Irgm1,ITCH,ITGAM,ITGB2,JAK1,KCNN4,LYN,MAPK3,MFGE8,MTA2,MYH9,NOS2,NOTCH2,NPC1,PKM,PLA2G4A,PLAU,POLD1,PPAT,PTGS2,PTPRC,PTPRJ,RBPJ,RIPK3,SF3B1,SQSTM1,STAT1,STUB1,TAX1BP1,TGFBR2,TKT,TLR7,TNFRSF1B,TPI1,TSTA3	52
**Cancer,Organismal Injury and Abnormalities,Reproductive System Disease**	Breast cancer	9.1E-06			ACAA1,AKR1B1,AP3B1,ATP1A1,ATP2C1,CCT3,CDK1,CMAS,CNDP2,CSE1L,CTSD,CTSK,DDX17,DDX39B,DENND4B,DHX29,DKC1,DNM1L,EIF3A,EIF3B,EIF3C,EIF3E,EIF3F,ENO1,FASN,FLNA,FYN,GBA,GNA13,GNAI3,GNAS,HIST1H1C,HLA-A,HNRNPM,HSP90AA1,HSP90AB1,IDH1,ITCH,JAK1,KPNA2,KRT1,LGALS3BP,MAPK3,MCM2,MOV10,MYH9,NANS,NCF2,NCL,NOTCH2,NPC1,NRAS,NUP93,NUS1,PCNA,PFN1,PGK1,PI4K2A,PKM,PLAU,PLEC,PLXNA1,POLD1,POLR2A,PPP2R1A,PRIM2,PTGS2,PTPRC,PTPRJ,RAB31,RAB5C,RANGAP1,RAP1B,RPL4,RPL6,RTCB,SF3B1,SFPQ,SHMT2,SLC20A1,SLC23A2,STAT1,STEAP3,SUPT5H,TCP1,TGFBR2,TNFRSF1B,TPI1,TRIM25,TRPV2,TSG101,UBA1,UBA2,UCHL5,VWA5A,XPO1	96
**Cellular Development,Cellular Growth and Proliferation,Embryonic Development,Hematological System Development and Function,Hematopoiesis,Lymphoid Tissue Structure and Development,Organ Development,Organismal Development,Tissue Development**	Lymphopoiesis	9.2E-06	Increased	2.554	ABCG1,AP3B1,ATP7A,C5AR1,CD36,DNMT1,DOCK2,EEF1D,FCER1G,FYN,GRB2,HLA-A,HNRNPL,HSP90AA1,ICAM1,IL6ST,ITCH,ITGAM,ITGB2,JAK1,LILRB4,LYN,MAPK3,MSN,NOS2,NOTCH2,PTGS2,PTPRC,PTPRJ,RBPJ,RIPK3,RPS6,SLC3A2,STAT1,TGFBR2,TLR7,TNFRSF1B,TPP2	38
**Cancer,Organismal Injury and Abnormalities,Respiratory Disease**	Development of lung carcinoma	9.5E-06			AHNAK,AP3B1,ATIC,CCT7,CDK1,CNDP2,CORO1C,CPD,CRYZ,DDX17,DDX3X,DIS3,EIF3E,ENO1,G3BP1,GART,GNAS,GPNMB,HIST1H1C,HSD17B4,HSP90AA1,HSP90AB1,IDH1,IL6ST,JAK1,KIDINS220,KRT10,LIG1,LYN,MCM2,MSN,MTA2,MYH9,NOTCH2,NRAS,NUP93,PCNA,PFAS,PKM,PLA2G4A,PLEC,POLR2A,PPP1R7,PPP2R1A,PRIM2,PRPF19,PTGS2,PTPRA,PTPRC,RALA,SCFD1,SF3B1,SLCO4A1,SRM,STAT1,TGFBR2,TPI1,TRIM25,TTC37	59

Table S4

**Cellular Function and Maintenance**	Lymphocyte homeostasis	9.7E-06	Increased	2.751	ABCG1,AP3B1,ATP7A,C5AR1,DNMT1,DOCK2,EEF1D,FCER1G,FYN,GRB2,HLA-A,HNRNPL,HSP90AA1,ICAM1,IL6ST,ITCH,ITGB2,JAK1,LILRB4,LYN,MAPK3,NOS2,NOTCH2,PTGS2,PTPRC,RBPJ,RIPK3,RPS6,SLC3A2,STAT1,TGFBR2,TLR7,TNFRSF1B,TPP2	34
**Cell-To-Cell Signaling and Interaction**	Binding of myeloid cells	9.8E-06	Increased	2.489	FERMT3,FYN,ICAM1,Irgm1,ITGAM,ITGB2,LYN,MSN,NOS2,NOTCH2,PLAU,PPP2CA,PTGS2,PTPRC,RAP1B,TGFBR2,TRPV2	17
**Protein Trafficking**	Interaction of protein	9.9E-06			ACACA,ADSL,APEX1,CD36,CRYZ,DNM1L,ENO1,FARSA,FARSB,GFPT1,IDE,KRT1,KRT10,NRAS,PKM,PPAT,SHMT1,SHMT2,STAT1	19
**Cancer,Organismal Injury and Abnormalities,Respiratory Disease**	Respiratory system tumor	1E-05		1.273	ACLY,ADAM8,AHNAK,AKR1B1,AKR1B10,ALCAM,AP3B1,APEX1,ARAP1,ATIC,ATP1A1,ATP5F1B,ATP7A,C5AR1,CCT7,CDK1,CNDP2,CORO1C,CPD,CRYZ,CTSD,DDX17,DDX3X,DIS3,DNAJA1,DNMT1,DOCK2,EDIL3,EEF1G,EIF2S1,EIF3A,EIF3E,ENO1,FASN,FLNA,G3BP1,G6PD,GART,GNAS,GPNMB,HIST1H1C,HLA-A,HSD17B4,HSP90AA1,HSP90AB1,ICAM1,IDH1,IL6ST,Irgm1,JAK1,KIDINS220,KRT1,KRT10,LIG1,LYN,MCM2,MFGE8,MSN,MTA2,MVP,MYH9,MYO1C,MYO1G,NCL,NDRG1,NOS2,NOTCH2,NRAS,NUP93,PCNA,PFAS,PKM,PLA2G4A,PLEC,POLR2A,PPP1R7,PPP2R1A,PPP2R2A,PRIM2,PRPF19,PTGS2,PTPRA,PTPRC,PTPRJ,RACK1,RALA,RBBP7,RNF213,RNPEP,RPL7,RPS27A,RPS6,SCFD1,SF3B1,SHMT2,SLC29A1,SLCO4A1,SQSTM1,SRM,STAT1,TGFBR2,TPI1,TRIM25,TRPV2,TSG101,TTC37,UBA1,VIM,XPNPEP1	109
**Gene Expression**	Expression of RNA	1E-05		1.059	ABCG1,ACTR2,ACTR3,ATP7A,CAND1,CD36,CD47,CDK1,CNOT1,CSE1L,DDX17,DDX21,DDX3X,DNMT1,EIF2A,EIF2S1,EIF2S3,EIF3B,EIF3C,EIF3D,EIF3E,EIF3F,EIF3I,EIF3L,EIF3M,ENO1,FARSB,FLOT2,FYN,GNA13,GNAS,HELZ2,HIST1H1C,HNRNPU,HSPA4,ITGAM,JAK1,KPNA2,LYN,MAPK3,MARS,MCM7,MOV10,MSN,MTA2,MYADM,NAA15,NCBP1,NCL,NOS2,NOTCH2,NRAS,PCNA,PFN1,PHGDH,POLR1C,POLR2A,POLR2B,PPP1CA,PPP2CA,PTPRC,RACK1,RALA,RBBP7,RBPJ,RNMT,RPL10,RPL24,RPL27A,RPL6,RPS14,RPS27A,RPS9,SARS,SFPQ,SHMT1,SIRPA,SQSTM1,STAT1,STRAP,SUPT5H,TGFBR2,TNFRSF1B,TNIP1,TRIM28,TSG101,VASP,VIM,WARS,WDR61,WWP2,XPO1,YBX1	93
**Nucleic Acid Metabolism, Small Molecule Biochemistry**	Synthesis of purine nucleotide	1.1E-05		-1.982	ADSL,ADSSL1,ATP5F1B,CDK1,FASN,G6PD,GMDS,GMPPB,GMPS,MTHFD1,NOS2,PKM,PPAT,TSTA3	14
**Cell-To-Cell Signaling and Interaction, Cellular Function and Maintenance,Inflammatory Response**	Phagocytosis of phagocytes	1.1E-05	Increased	2.224	CD36,CD47,DOCK2,FCER1G,FYN,ICAM1,ITGAM,ITGB2,MFGE8,PLAU,PTPRC,PTPRJ,SIRPA,TRPV2	14
**Cell Morphology**	Morphology of blood cells	1.1E-05			Abcb1b,CD36,CD47,CTSD,FCER1G,FERMT3,FYN,GBA,GNA13,HLA-A,ICAM1,Irgm1,ITGAM,ITGB2,LIG1,LILRB4,LYN,NRAS,PTPRC,PTPRJ,RIPK3,SLC12A4,SLC7A1,STAT1,STEAP3,TFRC,TPP2,TSTA3	28
**Amino Acid Metabolism, Small Molecule Biochemistry**	Synthesis of serine family amino acid	1.1E-05			MTHFD1,PHGDH,PKM,SHMT1,SHMT2	5
**Amino Acid Metabolism, Small Molecule Biochemistry**	Metabolism of amino acid analogs	1.1E-05			AHCY,ATIC,DNMT1,GART,MTHFD1,PHGDH,SHMT1,SHMT2	8

Table S4

**Cell-To-Cell Signaling and Interaction, Hematological System Development and Function**	Activation of blood cells	1.1E-05	Increased	2.207	AHNAK,AP3B1,C5AR1,CD36,CD47,DOCK2,FCER1G,FLNA,FYN,GNA13,GNAI2,GPNMB,HLA-A,HSPA4,ICAM1,ITCH,ITGAM,ITGB2,KCNN4,KIDINS220,LGALS3BP,LYN,MAPK3,NDRG1,NOS2,NOTCH2,NPC1,PLEK,PTGS2,PTPRC,PTPRJ,RAB5B,RAB8B,RBPJ,SIRPA,STAT1,TGFBR2,TLR7,TNFRSF1B,VAMP8,VASP	41
**Cell-To-Cell Signaling and Interaction, Hematological System Development and Function,Immune Cell Trafficking**	Adhesion of lymphocytes	1.2E-05		1.032	CD47,FERMT3,FLOT1,GNAI2,ICAM1,IL6ST,ITGB2,JAK1,MYADM,MYO1G,PLAU,RAP1B	12
**Cell Death and Survival**	Apoptosis of blood cells	1.2E-05	Increased	2.281	ABCG1,ADAM8,CD47,CTSD,FCER1G,FYN,GNAS,HIST1H1C,HSP90AB1,ICAM1,IL6ST,Irgm1,ITGAM,ITGB2,LYN,NOS2,NPC1,PFN1,PLA2G4A,PTPRC,RIPK3,RPS6,SF3B1,STAT1,STUB1,TLR7,TNFRSF1B,TPP2,TRIM28,WWP2	30
**Dermatological Diseases and Conditions, Organismal Injury and Abnormalities**	Keratosis	1.2E-05		-0.447	G6PD,IDH1,KRT1,KRT10,KRT9,MTA2,PGD,PTGS2,RHBDF2,STAT1,TGFBR2,TKT,TLR7	13
**Cell-To-Cell Signaling and Interaction, Cellular Function and Maintenance,Hematological System Development and Function**	Phagocytosis of myeloid cells	1.2E-05	Increased	2.226	CD36,CD47,DOCK2,FCER1G,FYN,ICAM1,ITGAM,ITGB2,MFGE8,PLAU,PTPRC,PTPRJ,SIRPA,TRPV2	14
**Connective Tissue Disorders,Immunological Disease,Inflammatory Disease,Organismal Injury and Abnormalities,Skeletal and Muscular Disorders**	Systemic lupus erythematosus	1.2E-05			DNMT1,GNAS,ICAM1,IL6ST,ITGAM,JAK1,LYN,MTA2,MYH9,NCF2,OASL,PPAT,PPP1CA,PPP2CA,PTGS2,PTPRC,RAB31,RAB5A,RIPK3,STAT1,TLR7,TRIM25	22

Table S4

**Organismal Functions**	Homeostasis of rodents	1.3E-05			CD36,CLUH,GALK1,GNAS,HELZ2,NOS2,PTGS2	7
**Cancer,Gastrointestinal Disease,Organismal Injury and Abnormalities**	Colorectal cancer	1.3E-05		-1	ABCA3,ABCE1,ACLY,ACTA1,ADSL,ADSSL1,AHCY,AKR1B1,AKR1B10,ALCAM,AP3B1,APEX1,ARAP1,ATIC,ATP2B1,ATP7A,CA2,CCT2,CCT4,CCT5,CCT7,CD36,CFL1,CLUH,CMAS,CMIP,CNOT1,COLEC12,COPB1,CORO1C,CORO7/CORO7-PAM16,CPD,CPNE8,CRYZ,CSE1L,DCTN1,DDX17,DDX21,DDX39B,DENND4B,DHX15,DHX29,DIS3,DKC1,DNAJA1,DNAJC13,DNMT1,DOCK2,DPYSL2,EDIL3,EHD1,EIF2S3,EIF3C,EIF3F,EIF3I,EIF3L,EIF3M,ENO1,FASN,FLNA,FYN,G6PD,GALK1,GART,GMPS,GNA13,GNAI2,GNAS,GRB2,HIST1H1C,HK3,HLA-A,HNRNPL,HNRNPM,HSP90AA1,HSP90AB1,HSPA4,ICAM1,IDE,IDH1,IFITM3,IL6ST,IPO5,ITGAM,ITGB2,JAK1,KCNN4,KIDINS220,KPNA2,KPNB1,KRT1,KRT2,LIG1,LILRB4,LRP12,LYN,MAPK3,MARS,MAT2A,MCM2,MCM7,MRI1,MTA2,MYH9,MYO1C,MYO1E,MYOF,NANS,NCL,NDRG1,NOTCH2,NRAS,NUP93,PCNA,PFAS,PGK1,PKM,PLAU,PLEC,PLXNB2,POLD1,PPP1CA,PPP2R1A,PREP,PTGS2,PTPN23,PTPRA,PTPRC,PTPRJ,RAB31,RAB5B,RCC2,RHBDF2,RNF213,RNH1,RNMT,RPL28,RPS6,RTCB,SF3B1,SIPA1,SIRPA,SLC15A3,SLC16A3,SLC20A1,SLC23A2,SLC2A1,SLC38A2,SLC3A2,SLFN13,SMPDL3B,SND1,SNRNP200,SNX2,SPRED1,SQSTM1,SRM,STAT1,STEAP3,STRAP,STUB1,SUPT5H,TAX1BP1,TCIRG1,TCP1,TFRC,TGFBR2,TLR7,TMEM59,TPI1,TPP2,TRIM28,TRPV2,UBA1,VIM,VPS13C,WDR82,XPO1,XPO7	179
**Cancer,Organismal Injury and Abnormalities,Respiratory Disease**	Unresectable malignant pleural mesothelioma	1.4E-05			ATIC,GART,HSP90AA1,HSP90AB1	4
**Cell-To-Cell Signaling and Interaction, Cellular Function and Maintenance,Hematological System Development and Function,Inflammatory Response**	Phagocytosis of blood platelets	1.4E-05		0	CD47,ITGAM,ITGB2,SIRPA	4
**Cancer,Organismal Injury and Abnormalities,Respiratory Disease**	ALK mutation negative EGFR mutation negative nonsquamous non-small cell lung carcinoma	1.4E-05			ATIC,GART,HSP90AA1,HSP90AB1	4
**Cancer,Organismal Injury and Abnormalities,Respiratory Disease**	EGFR L858R activating mutation positive EGFR T790M mutation negative non-small cell lung cancer	1.4E-05			ATIC,GART,HSP90AA1,HSP90AB1	4
**Cell-To-Cell Signaling and Interaction, Hematological System Development and Function**	Binding of mononuclear leukocytes	1.4E-05		1.341	CD47,FERMT3,FLOT1,GNAI2,ICAM1,IL6ST,ITGAM,ITGB2,JAK1,MSN,MYADM,MYO1G,PLAU,RAP1B,TFRC,TGFBR2	16

Table S4

**Cancer,Hematological Disease,Immunological Disease,Organismal Injury and Abnormalities**	Type M3 acute myeloid leukemia	1.4E-05			DNMT1,HK3,IDH1,NRAS,POLD1,PPAT,PRIM2,SF3B1	8
**Connective Tissue Development and Function,Skeletal and Muscular System Development and Function**	Resorption of bone	1.4E-05		1.434	ADAM8,CA2,CD47,CTSK,FCER1G,MFGE8,NOS2,NOTCH2,PTGS2,RAB7A,RBPJ,STAT1,TCIRG1,TFRC,TNFRSF1B	15
**Hematological System Development and Function,Tissue Morphology**	Quantity of myeloid cells	1.5E-05		-0.964	ADAM8,C5AR1,CD36,CD47,FLNA,GBA,GNAI2,GNAS,ICAM1,IL6ST,ITGAM,ITGB2,LILRB4,LYN,NOS2,NPC1,PLAU,PPP2CA,PREP,PTGS2,SIPA1,SIRPA,SLC20A1,SPRED1,TCIRG1,TGFBR2,TNFRSF1B,TSTA3,VASP,YBX1	30
**Cell Morphology,Cellular Movement,Hematological System Development and Function,Immune Cell Trafficking,Inflammatory Response**	Cell spreading of neutrophils	1.5E-05		0.447	FERMT3,ICAM1,ITGAM,ITGB2,LYN	5
**Cellular Movement**	Homing of blood cells	1.5E-05		1.629	ADAM8,C5AR1,CD47,DOCK2,FCER1G,FLOT1,FYN,GNA13,GNAI2,GNAI3,GNAS,ICAM1,ITGAM,ITGB2,JAK1,LYN,MAPK3,PFN1,PLAU,PLEC,PTPRC,PTPRJ,RAP1B,TCIRG1,TRPV2	25
**Cell-To-Cell Signaling and Interaction**	Response of tumor cell lines	1.5E-05		1.969	FCER1G,GRB2,ITGAM,JAK1,LYN,PFN1,RAB31,RALA,VIM	9

Table S4

**Cell-To-Cell Signaling and Interaction, Cellular Assembly and Organization,Hematological System Development and Function,Immune Cell Trafficking**	Cell-cell adhesion of leukocytes	1.6E-05			FERMT3,ICAM1,ITGAM,ITGB2,PTPRC,TNIP1	6
**Amino Acid Metabolism, Small Molecule Biochemistry**	Metabolism of sulfur amino acid	1.6E-05			AHCY,GART,MTHFD1,PHGDH,SHMT1,SHMT2	6
**Cell-To-Cell Signaling and Interaction, Hematological System Development and Function,Immune Cell Trafficking,Inflammatory Response**	Adhesion of macrophages	1.6E-05		0.6	ICAM1,Irgm1,ITGB2,LYN,PLAU,PTGS2	6
**Cellular Function and Maintenance,Connective Tissue Development and Function,Skeletal and Muscular System Development and Function,Tissue Development**	Function of osteoclasts	1.6E-05			CD47,CTSK,FCER1G,IL6ST,NOS2,PTPRC,SLC1A5,TCIRG1	8

Table S4

**Cellular Development,Cellular Growth and Proliferation,Hematological System Development and Function,Hematopoiesis,Lymphoid Tissue Structure and Development,Tissue Development**	Leukopoiesis	1.6E-05	Increased	2.877	ABCG1,AP3B1,ATP7A,C5AR1,CD36,CD47,DNMT1,DOCK2,EEF1D,FASN,FCER1G,FYN,GRB2,HLA-A,HNRNPL,HSP90AA1,ICAM1,IL6ST,ITCH,ITGAM,ITGB2,JAK1,LILRB4,LYN,MAPK3,MFGE8,MSN,NOS2,NOTCH2,PLA2G4A,PTGS2,PTPRC,PTPRJ,RALA,RBPJ,RIPK3,RPS6,SLC3A2,STAT1,TGFBR2,TLR7,TNFRSF1B,TPP2	43
**Cancer,Organismal Injury and Abnormalities,Respiratory Disease**	Lung adenocarcinoma	1.6E-05			AHNAK,AP3B1,ATIC,CCT7,CDK1,CNDP2,CORO1C,CPD,CRYZ,DDX17,DDX3X,DIS3,EIF3E,ENO1,G3BP1,GART,GNAS,GPNMB,HIST1H1C,HSD17B4,HSP90AA1,HSP90AB1,IDH1,IL6ST,JAK1,KIDINS220,KRT10,LIG1,LYN,MCM2,MSN,MTA2,MYH9,NOTCH2,NRAS,NUP93,PCNA,PFAS,PKM,PLA2G4A,PLEC,POLR2A,PPP1R7,PPP2R1A,PRIM2,PRPF19,PTGS2,PTPRA,PTPRC,SCFD1,SF3B1,SLCO4A1,SRM,STAT1,TGFBR2,TPI1,TRIM25,TTC37	58
**Cancer,Hematological Disease,Immunological Disease,Organismal Injury and Abnormalities**	Diffuse lymphoma	1.7E-05			ABCG1,ADSL,AHNAK,CCT3,CD36,CNOT1,DNMT1,DOCK2,EIF2A,FYN,GNA13,GNAI2,GRB2,HIST1H1C,HNRNPM,HNRNPU,HSP90AA1,HSP90AB1,IDH1,JAK1,KRT2,MYO1G,MYOF,NOTCH2,NRAS,POLD1,PPAT,PRIM2,PTPRC,RPSA,SARS,SF3B1,SHMT1,SHMT2,STEAP3,TNIP1,USP8,XPO1	38
**Cellular Assembly and Organization,Cellular Function and Maintenance**	Organization of actin cytoskeleton	1.7E-05		-0.378	ACTR2,ARAP1,ATP2C1,CD47,CFL1,CORO1B,CORO1C,CORO7/CORO7-PAM16,DOCK2,DPYSL2,FLNA,ICAM1,MYH9,PLAU,PLEK,RALA,RAN,RPS6,SIRPA,VASP	20
**Cancer,Cardiovascular Disease,Hematological Disease,Organismal Injury and Abnormalities**	Myelodysplastic syndrome with ring sideroblasts	1.7E-05			DNMT1,IDH1,NRAS,POLD1,PPAT,PRIM2,SF3B1	7
**Cancer,Organismal Injury and Abnormalities**	Stage III cancer	1.8E-05			ATIC,CCT3,FYN,GART,HSP90AA1,HSP90AB1,LYN,NANS,PCNA,POLD1,POLR2A,PRIM2,PTGS2,PTPRC,TGFBR2,TLR7	16

Table S4

**Cellular Movement,Hematological System Development and Function,Immune Cell Trafficking,Inflammatory Response**	Cellular infiltration by phagocytes	1.8E-05		1.353	ADAM8,C5AR1,CD36,EDIL3,FCER1G,GBA,ICAM1,ITGAM,ITGB2,MAPK3,NDRG1,NOS2,PLA2G4A,PLAU,PTGS2,STAT1,TGFBR2,TNFRSF1B,TNIP1,YBX1	20
**Amino Acid Metabolism,Small Molecule Biochemistry**	Metabolism of serine family amino acid	1.9E-05			GART,MTHFD1,PHGDH,PKM,SHMT1,SHMT2	6
**Cardiovascular System Development and Function,Cell-To-Cell Signaling and Interaction**	Binding of vascular endothelial cells	2E-05		1.171	CD36,CD47,FERMT3,ICAM1,ITGAM,ITGB2,KRT1,RACK1,STAT1,STX6,VIM	11
**Infectious Diseases**	Transport of virus	2E-05			DCTN1,KPNB1,RAN,RPS27A,XPO1	5
**Post-Translational Modification**	Aggregation of protein	2E-05		-0.447	NOS2,SQSTM1,STUB1,VAMP8,VASP	5
**Cancer,Organismal Injury and Abnormalities,Respiratory Disease**	Biphasic malignant pleural mesothelioma	2E-05			ATIC,GART,HSP90AA1,HSP90AB1,LYN	5
**Cancer,Organismal Injury and Abnormalities,Respiratory Disease**	Epithelioid malignant pleural mesothelioma	2E-05			ATIC,GART,HSP90AA1,HSP90AB1,LYN	5
**Cancer,Gastrointestinal Disease,Hepatic System Disease,Organismal Injury and Abnormalities**	Hepatitis B virus-related hepatocellular carcinoma	2E-05			ENO1,GALK1,HSPA4,RACK1,RPSA,UCHL5,VIM	7

Table S4

**Cancer,Organismal Injury and Abnormalities,Reproductive System Disease**	Development of genital tumor	2E-05			ABCA3,ABCE1,ACSL4,ACTR2,ACTR3,ADSL,AHNAK,AKR1B1,AP3B1,ATIC,ATP2B1,ATP2C1,ATP7A,CA2,CAND1,CCT5,CCT6A,CD36,CD47,CLUH,CNOT1,COLEC12,COPB1,CORO1C,CSE1L,CTPS1,DCTN1,DDX3X,DIS3,DNAJA1,DNAJC13,DNMT1,ECPAS,EEF1D,EIF2S1,EIF3A,EIF3D,EIF3E,ENO1,FLNA,FLOT1,GART,GFPT1,GMDS,GNA13,GNAS,GNB2,GRB2,HIST1H1C,HIST1H2AJ,HLA-A,HNRNPL,HNRNPM,HNRNPU,HSD17B4,HSP90AA1,HSP90AB1,IDH1,IPO5,ITCH,ITGAM,JAK1,KIDINS220,KPNA2,KRT1,KRT9,LIG1,LILRB4,LYN,MAPK3,MAT2B,MCM2,MCM7,MDH1,MEMO1,MFGE8,MSN,MYH9,MYO1C,MYO1E,MYOF,NAA15,NCBP1,NDRG1,NOP56,NOS2,NOTCH2,NPC1,NRAS,NUP93,OASL,OLA1,PCNA,PDXK,PFAS,PKM,PLD3,PLEC,PLXNA1,PLXNB2,PNKP,POLR1C,PPP1R7,PPP2R1A,PRIM2,PTGS2,PTPRA,PTPRJ,RALA,RBBP7,RIPK3,RNF149,RNPEP,RPF2,RPL14,RPS6,SCFD1,SF3B1,SFPQ,SIRPA,SLC15A3,SLC16A3,SLC20A1,SLC38A2,SLC7A1,SMPDL3B,SND1,SNRNP200,SNX27,STAT1,STOM,SUPT5H,TALDO1,TFRC,TGFBR2,TLR7,TMEM59,TNIP1,TPI1,TPP2,TRIM25,TSR1,TTC37,TTLL12,UBA1,UBA2,USP5,USP8,VAMP8,VIM,VPS13C,WWP2,XPO1,XPO7	154
**Cancer,Organismal Injury and Abnormalities**	Advanced extracranial solid tumor	2.1E-05		1.969	ATIC,C5AR1,CD36,COLEC12,CRYZ,CSE1L,DPYSL2,FLNA,FYN,GART,GPNMB,HSP90AA1,HSP90AB1,ICAM1,JAK1,KIDINS220,LYN,NRAS,PLAU,PLEC,POLD1,PRIM2,PTGS2,PTPRC,PTPRJ,RAB31,RPL7,RPS27A,RPS6,SLC16A3,SQSTM1,STAT1,STEAP3,XPO1	34
**Cell-To-Cell Signaling and Interaction, Hematological System Development and Function**	Binding of lymphocytes	2.2E-05		1.034	CD47,FERMT3,FLOT1,GNAI2,ICAM1,IL6ST,ITGB2,JAK1,MSN,MYADM,MYO1G,PLAU,RAP1B,TFRC	14
**Cancer,Gastrointestinal Disease,Organismal Injury and Abnormalities**	Colon tumor	2.2E-05		0.927	ABCA3,ABCE1,ACLY,ACTA1,ADSL,ADSSL1,AHCY,AKR1B1,AKR1B10,AP3B1,APEX1,ARAP1,ATIC,ATP2B1,ATP7A,CA2,CCT2,CCT4,CCT5,CCT7,CLUH,CMAS,CMIP,CNOT1,COLEC12,COPB1,CORO1C,CORO7/CORO7-PAM16,CPD,CPNE8,DCTN1,DDX17,DDX21,DDX39B,DENND4B,DHX15,DHX29,DIS3,DNAJA1,DNAJC13,DNMT1,DOCK2,EDIL3,EHD1,EIF2S3,EIF3C,EIF3F,EIF3I,EIF3L,EIF3M,FASN,FLNA,FYN,G6PD,GALK1,GART,GMPS,GNA13,GNAS,GRB2,HIST1H1C,HK3,HLA-A,HNRNPL,HNRNPM,HSD17B4,HSPA4,IDE,IDH1,IFITM3,IL6ST,IPO5,ITGAM,ITGB2,JAK1,KCNN4,KIDINS220,KPNA2,KPNB1,KRT1,KRT2,LIG1,LILRB4,LRP12,MAPK3,MARS,MCM2,MCM7,MRI1,MTA2,MYH9,MYO1C,MYO1E,MYOF,NANS,NCL,NDRG1,NOTCH2,NRAS,NUP93,PCNA,PFAS,PGK1,PKM,PLA2G4A,PLAU,PLEC,PLXNB2,POLD1,PPP1CA,PPP2R1A,PREP,PTGS2,PTPN23,PTPRA,PTPRC,PTPRJ,RAB5B,RCC2,RHBDF2,RNF213,RNH1,RNMT,RPL28,RPS6,RTCB,SF3B1,SIPA1,SIRPA,SLC15A3,SLC20A1,SLC23A2,SLC38A2,SLC3A2,SLFN13,SMPDL3B,SND1,SNRNP200,SNX2,SPRED1,SQSTM1,SRM,STAT1,STRAP,STUB1,SUPT5H,TCIRG1,TCP1,TFRC,TGFBR2,TLR7,TMEM59,TPP2,TRIM28,TRPV2,UBA1,VIM,VPS13C,WDR82,XPO1,XPO7	161
**Cancer,Hematological Disease,Organismal Injury and Abnormalities**	Recurrent hematologic cancer	2.2E-05			ATIC,DNMT1,FYN,GART,HSP90AA1,HSP90AB1,IDH1,LYN,POLD1,PPAT,PRIM2,PTGS2,SF3B1	13
**Nucleic Acid Metabolism, Small Molecule Biochemistry**	Metabolism of purine nucleotide	2.3E-05			ACLY,ADSL,ADSSL1,ATP5F1B,ATP7A,G6PD,GMDS,OLA1,PGD,TSTA3	10
**Cell-To-Cell Signaling and Interaction**	Aggregation of cells	2.3E-05	Increased	2.871	AKR1B1,CD36,CD47,FCER1G,FERMT3,FYN,GNA13,GNAI2,GNAS,ICAM1,ITGB2,LYN,NOS2,PLA2G4A,PLEK,PTGS2,RAP1B,SIRPA,TLR7,VASP	20

Table S4

**Cancer,Organismal Injury and Abnormalities,Reproductive System Disease**	Uterine tumor	2.3E-05			ABCA3,ABCE1,ACSL4,ACTR3,ADSL,AHNAK,AKR1B1,AP3B1,ATIC,ATP2B1,ATP2C1,ATP7A,CA2,CAND1,CCT5,CLUH,CNOT1,COLEC12,COPB1,CORO1C,CSE1L,CTPS1,DCTN1,DIS3,DNAJC13,DNMT1,ECPAS,EIF2S1,EIF3A,EIF3D,EIF3E,ENO1,FLNA,GART,GFPT1,GNAS,GNB2,GRB2,HIST1H1C,HIST1H2AJ,HLA-A,HNRNPL,HNRNPU,HSD17B4,HSP90AA1,HSP90AB1,IDH1,IPO5,ITGAM,JAK1,KIDINS220,KPNA2,KRT1,KRT9,LILRB4,LYN,MAPK3,MCM2,MCM7,MDH1,MEMO1,MFGE8,MSN,MYH9,MYO1C,NAA15,NCBP1,NDRG1,NOP56,NOS2,NOTCH2,NRAS,NUP93,OASL,PCNA,PDXK,PFAS,PKM,PLD3,PLEC,PLXNA1,PLXNB2,PPP1R7,PPP2R1A,PTGS2,PTPRA,PTPRJ,RALA,RBBP7,RIPK3,RPF2,RPL14,RPS6,SCFD1,SF3B1,SFPQ,SIRPA,SLC15A3,SLC16A3,SLC20A1,SLC38A2,SLC7A1,SMPDL3B,SND1,SNRNP200,SNX27,STAT1,SUPT5H,TALDO1,TFRC,TGFBR2,TLR7,TMEM59,TNIP1,TPP2,TSR1,TTC37,TTLL12,UBA1,UBA2,USP5,USP8,VAMP8,VIM,VPS13C,WWP2,XPO1,XPO7	128
**Cellular Function and Maintenance**	Engulfment of tumor cell lines	2.4E-05		0.893	CD36,DNM2,GRB2,HSP90AA1,ITGAM,LYN,PFN1,RAB31,RACK1,RALA,SFPQ,VIM	12
**Cancer,Organismal Injury and Abnormalities**	Genitourinary adenocarcinoma	2.4E-05			ABCA3,ABCE1,ACLY,ACSL4,ACTR1A,ACTR2,ADAM8,ADSL,ADSSL1,AHNAK,AKR1B1,AP3B1,APEX1,ARPC2,ATIC,ATP1A1,ATP2B1,ATP2C1,CA2,CAND1,CAPZA1,CCT3,CCT5,CCT6A,CD36,CD47,CLUH,CMIP,CNOT1,COLEC12,COPB1,CORO1C,CPD,CPNE8,CSE1L,CTPS1,CTSD,DCTN1,DDX17,DDX3X,DENND4B,DIS3,DNAJA1,DNAJC13,DNM1L,DNMT1,DOCK2,ECPAS,EDIL3,EEF1D,EEF1G,EIF2S1,EIF3A,EIF3E,EIF3F,EIF3I,ESYT1,FASN,FLNA,FLOT1,G3BP1,G6PD,GART,GFPT1,GMDS,GNA13,GNAI2,GNAS,GNB2,GPNMB,GUSB,HIST1H1C,HK3,HNRNPL,HNRNPM,HNRNPU,HSD17B4,HSP90AA1,HSP90AB1,HSPA4,IDH1,IPO5,ITCH,ITGAM,JAK1,KIDINS220,KPNA2,KRT1,KRT10,KRT2,KRT9,LIG1,LILRB4,LRP12,MAT2B,MCM2,MDH1,MEMO1,MFGE8,MSN,MYADM,MYH9,MYO1C,MYO1E,MYO1G,MYOF,NAA15,NOP56,NOS2,NOTCH2,NPC1,NRAS,NUP93,OLA1,PCNA,PDS5A,PDXK,PKM,PLD3,PLEC,PLEK,PLXNA1,PLXNB2,PNKP,POLD1,POLR1C,POLR2B,PPP1R7,PPP2R1A,PREP,PRIM2,PTGS2,PTPN23,PTPRA,PTPRC,PTPRJ,RAB5B,RALA,RANGAP1,RARS,RBBP7,RCC2,RIPK3,RNF149,RNF213,RNPEP,RPF2,RPL14,RPS27A,RRP9,SCFD1,SEC24B,SF3B1,SIRPA,SLC12A4,SLC16A3,SLC20A1,SLC29A1,SLC2A1,SLC3A2,SLC7A1,SMPDL3B,SND1,SNRNP200,SNX27,STAT1,STEAP3,STOM,STX6,SUPT5H,TALDO1,TGFBR2,TKT,TLR7,TNIP1,TOM1,TPI1,TPP2,TRIM25,TRIM28,TRPV2,TSR1,TTC37,TTLL12,UBA1,UBA2,USP5,USP8,VAMP8,VIM,VPS13C,VWA5A,WWP2,XPO1,XPO7,YBX1	196
**Molecular Transport,Protein Trafficking**	Import of green fluorescent protein	2.5E-05			KPNA2,KPNB1,RAN	3
**Dermatological Diseases and Conditions,Developmental Disorder,Hereditary Disorder,Organismal Injury and Abnormalities**	Epidermolytic hyperkeratosis	2.5E-05			KRT1,KRT10,KRT9	3
**Cellular Assembly and Organization**	Quantity of filaments	2.5E-05		1	ARAP1,CFL1,DNM2,FYN,IDE,ITGB2,NRAS,PLAU,PLEC	9
**Inflammatory Response**	Inflammatory response	2.5E-05		1.34	ACOD1,ADAM8,C5AR1,CD36,CD47,DOCK2,FCER1G,FLOT1,FYN,GNA13,GNAI2,GNAI3,GNAS,HLA-A,ICAM1,IL6ST,ITGAM,ITGB2,KRT1,LGALS3BP,LILRB4,LYN,MAPK3,MFGE8,NCL,NOS2,NPC1,PFN1,PLA2G4A,PLAU,PLEC,PTGS2,PTPRJ,RIPK3,SIRPA,SLC1A5,SMPDL3B,STAT1,TLR7,TNFRSF1B,TNIP1,TRPV2	42

Table S4

**Cellular Assembly and Organization,Cellular Function and Maintenance,Tissue Development**	Polymerization of actin filaments	2.5E-05		-0.707	ACTR3,ARPC2,CFL1,CORO7/CORO7-PAM16,GRB2,ICAM1,MYADM,MYO1C,PFN1,VASP	10
**Cancer,Hematological Disease,Immunological Disease,Organismal Injury and Abnormalities**	Diffuse B-cell lymphoma	2.6E-05			ABCG1,AHNAK,CD36,CNOT1,DNMT1,DOCK2,EIF2A,FYN,GNA13,GRB2,HIST1H1C,HNRNPM,HNRNPU,HSP90AA1,HSP90AB1,IDH1,JAK1,KRT2,MYO1G,MYOF,NOTCH2,NRAS,POLD1,PPAT,PRIM2,PTPRC,RPSA,SARS,SF3B1,SHMT1,SHMT2,STEAP3,TNIP1,USP8,XPO1	35
**Carbohydrate Metabolism,Nucleic Acid Metabolism,Small Molecule Biochemistry**	Synthesis of nucleoside diphosphate sugar	2.6E-05			AMDHD2,GFPT1,GMDS,GMPPB,TSTA3	5
**Amino Acid Metabolism,Small Molecule Biochemistry**	Synthesis of alpha-amino acid	2.7E-05		0	CNDP2,MTHFD1,NOS2,PHGDH,PKM,SHMT1,SHMT2	7
**Connective Tissue Development and Function,Skeletal and Muscular System Development and Function,Tissue Morphology**	Quantity of osteoclasts	2.7E-05		1.012	C5AR1,CD47,CTSK,GNAS,GPNMB,IL6ST,MFGE8,NOS2,NOTCH2,SQSTM1,STAT1,TGFBR2	12
**Hereditary Disorder,Neurological Disease,Organismal Injury and Abnormalities**	Autosomal dominant neuropathy	2.7E-05			DCTN1,DNAJC13,DNM2,GARS,GBA,KIDINS220,MARS,RAB7A,SQSTM1	9
**RNA Post-Transcriptional Modification**	Capping of mRNA	2.8E-05			CMTR1,NCBP1,POLR2A,POLR2B,RNMT,SUPT5H	6

Table S4

**Cancer,Hematological Disease,Immunological Disease,Organismal Injury and Abnormalities**	Chronic myeloid leukemia	2.8E-05			CDK1,DNMT1,FCER1G,FYN,LYN,NCSTN,NOTCH2,NRAS,POLD1,PPAT,PRIM2,RPL3,SF3B1,SIPA1	14
**Cellular Development,Cellular Growth and Proliferation,Connective Tissue Development and Function,Skeletal and Muscular System Development and Function,Tissue Development**	Formation of osteoclasts	2.9E-05		0.79	CD47,FLNA,ICAM1,MAPK3,MFGE8,PLA2G4A,PTGS2,RBPJ,SIRPA,SQSTM1,STAT1,TNFRSF1B	12
**Cell-To-Cell Signaling and Interaction, Cellular Function and Maintenance,Hematological System Development and Function,Inflammatory Response**	Phagocytosis by macrophages	2.9E-05	Increased	2.36	CD36,CD47,DOCK2,FCER1G,FYN,ITGAM,ITGB2,MFGE8,PTPRC,PTPRJ,SIRPA,TRPV2	12
**Cancer,Gastrointestinal Disease,Organismal Injury and Abnormalities**	Colon cancer	2.9E-05			ABCA3,ABCE1,ACLY,ACTA1,ADSL,ADSSL1,AHCY,AKR1B1,AKR1B10,AP3B1,APEX1,ARAP1,ATIC,ATP2B1,ATP7A,CA2,CCT2,CCT4,CCT5,CCT7,CLUH,CMAS,CMIP,CNOT1,COLEC12,COPB1,CORO1C,CORO7/CORO7-PAM16,CPD,CPNE8,DCTN1,DDX17,DDX21,DDX39B,DENND4B,DHX15,DHX29,DIS3,DNAJA1,DNAJC13,DNMT1,DOCK2,EDIL3,EHD1,EIF2S3,EIF3C,EIF3F,EIF3I,EIF3L,EIF3M,FASN,FLNA,FYN,G6PD,GALK1,GART,GMPS,GNA13,GNAS,GRB2,HIST1H1C,HK3,HLA-A,HNRNPL,HNRNPM,HSPA4,IDE,IDH1,IFITM3,IL6ST,IPO5,ITGAM,ITGB2,JAK1,KCNN4,KIDINS220,KPNA2,KPNB1,KRT1,KRT2,LIG1,LILRB4,LRP12,MAPK3,MARS,MCM2,MCM7,MRI1,MTA2,MYH9,MYO1C,MYO1E,MYOF,NANS,NCL,NDRG1,NOTCH2,NRAS,NUP93,PCNA,PFAS,PGK1,PKM,PLAU,PLEC,PLXNB2,POLD1,PPP1CA,PPP2R1A,PREP,PTGS2,PTPN23,PTPRA,PTPRC,PTPRJ,RAB5B,RCC2,RHBDF2,RNF213,RNH1,RNMT,RPL28,RPS6,RTCB,SF3B1,SIPA1,SIRPA,SLC15A3,SLC20A1,SLC23A2,SLC38A2,SLC3A2,SLFN13,SMPDL3B,SND1,SNRNP200,SNX2,SPRED1,SQSTM1,SRM,STAT1,STRAP,STUB1,SUPT5H,TCIRG1,TCP1,TFRC,TGFBR2,TLR7,TMEM59,TPP2,TRIM28,TRPV2,UBA1,VIM,VPS13C,WDR82,XPO1,XPO7	159

Table S4

**Cancer,Gastrointestinal Disease,Hepatic System Disease,Organismal Injury and Abnormalities**	Hepatocellular carcinoma	3E-05			AKR1B10,ATP1A1,ATP1B3,CA2,CNOT1,COPG1,CSE1L,CTSD,DIS3,DOCK2,ENO1,FASN,GALK1,GNAS,HSP90AA1,HSP90AB1,HSPA4,ICAM1,IDH1,IFITM3,IL6ST,IPO7,JAK1,MAT2B,MCM2,NCL,NOS2,NRAS,PFN1,PKM,PLAU,POLD1,PRIM2,PTGS2,RACK1,RALA,RNF213,RNMT,RPSA,SF3B1,TGFBR2,TPI1,UCHL5,VIM	44
**Cancer,Organismal Injury and Abnormalities**	Mucinous neoplasm	3E-05			GNA13,GNAI2,GNAS,HSP90AA1,HSP90AB1,PKM,PPP2R1A,PTGS2,STAT1,TLR7	10
**Cell Morphology,Cellular Assembly and Organization,Cellular Function and Maintenance**	Reorganization of cytoskeleton	3E-05		0.924	ARAP1,ATP2C1,CD47,CFL1,DOCK2,DPYSL2,FLNA,ICAM1,MSN,MYH9,PLAU,PLEK,RALA,RPS6	14
**Cell-To-Cell Signaling and Interaction**	Response of myeloid leukocytes	3.1E-05		0.242	C5AR1,CD36,CD47,DOCK2,FCER1G,ICAM1,ITGAM,ITGB2,LYN,NOS2,PLAU	11
**Post-Translational Modification,Protein Synthesis,Protein Trafficking**	Homotetramerization of protein	3.3E-05			ACACA,CRYZ,DNM1L,GFPT1,IDE,PKM,PPAT,SHMT1,SHMT2	9
**Cancer,Organismal Injury and Abnormalities,Respiratory Disease**	EGFR exon 19 deletion positive EGFR T790M mutation negative non-squamous non-small cell lung cancer	3.4E-05			ATIC,GART,HSP90AA1,HSP90AB1	4
**Cancer,Organismal Injury and Abnormalities,Respiratory Disease**	EGFR L858R mutation positive EGFR T790M negative non-squamous non-small cell lung cancer	3.4E-05			ATIC,GART,HSP90AA1,HSP90AB1	4
**Cancer,Organismal Injury and Abnormalities**	Benign Tumors	3.5E-05		-1.572	ALCAM,ATP1A1,ATP2C1,CA2,CDK1,CLUH,DDX17,DKC1,DNM1L,DNMT1,FLNA,GNAS,GRB2,HSP90AB1,IDH1,IFIT1B,IFITM3,IL6ST,LIG1,LYN,MAPK3,MDH1,MSN,MYH9,NDRG1,NRAS,PCNA,PLD3,PLXNB2,POLD1,POLR1C,PRIM2,PTGS2,SEC24B,SLC29A1,SLC2A1,SLC7A1,SUPT5H,TGFBR2,TMEM59,TNFRSF1B,TTC37,USP8	43

Table S4

**Cancer,Organismal Injury and Abnormalities,Reproductive System Disease**	Cervical cancer	3.5E-05			ACTR3,ATIC,EIF3A,ENO1,GART,GNAS,HIST1H2AJ,HLA-A,HSP90AA1,HSP90AB1,KRT1,KRT9,MCM2,MCM7,NOTCH2,NRAS,PKM,PTGS2,SF3B1,SND1,TFRC,TGFBR2,TLR7,VIM,XPO1	25
**Hematological System Development and Function,Immune Cell Trafficking,Inflammatory Response,Tissue Development**	Accumulation of phagocytes	3.6E-05		-0.078	ABCG1,C5AR1,EDIL3,GNAI2,ITGAM,ITGB2,NOS2,NPC1,PFN1,PLAU,STAT1,TLR7,TNFRSF1B	13
**Cellular Assembly and Organization**	Organization of organelle	3.6E-05			ARAP1,ATP5F1B,ATP7A,CFL1,CLUH,CORO1B,CORO7/CORO7-PAM16,DNAJC13,DNM1L,DPYSL2,FLNA,HSP90AB1,KRT9,MSTO1,MYH9,MYOF,NUP93,PLAU,PLEK,RAB5A,RAB5B,RAB5C,RAB8B,SFPQ,SIRPA,SQSTM1,STX6,TSG101,USP8,VIM,VPS13C	31
**Cellular Movement**	Invasion of breast cancer cell lines	3.6E-05		0.072	AHNAK,CSE1L,CTSK,DNM1L,EIF3E,FERMT3,IFITM3,PTGS2,RAB5A,SDCBP,SIPA1,SNAP23,TCIRG1,TGFBR2,VIM	15
**Cardiovascular System Development and Function**	Development of vasculature	3.7E-05		1.644	ADAM8,AKR1B1,ALCAM,ATP5F1B,ATP7A,C5AR1,CD36,DAAM1,DDX3X,DNM2,EDIL3,FERMT3,FLNA,G6PD,GNA13,HSP90AA1,ICAM1,IL6ST,ITGAM,ITGB2,KRT1,MFGE8,MYH9,MYO1E,MYOF,NCL,NDRG1,NOS2,NOTCH2,NRAS,NUS1,PGK1,PKM,PLA2G4A,PLAU,PLXNB2,PTGS2,PTPRJ,RAP1B,RBPJ,RNF213,RNH1,RPSA,SARS,SLC4A7,SPRED1,STAT1,STX6,TGFBR2,TKT,TNFRSF1B,VIM,WARS	53
**Cancer,Organismal Injury and Abnormalities,Reproductive System Disease**	Female genital tract cancer	3.8E-05			ABCA3,ABCE1,ACSL4,ACTR2,ACTR3,ADSL,AHNAK,AKR1B1,ALCAM,AP3B1,ATIC,ATP2B1,ATP2C1,ATP7A,CA2,CAND1,CCT5,CCT6A,CD36,CD47,CDK1,CLUH,CNOT1,COLEC12,COPB1,CORO1C,CSE1L,CTPS1,DCTN1,DDX3X,DIS3,DNAJA1,DNAJC13,DNMT1,ECPAS,EEF1D,EIF2S1,EIF3A,EIF3D,EIF3E,EIF3F,ENO1,FLNA,FLOT1,GART,GFPT1,GMDS,GNA13,GNAS,GNB2,GRB2,HIST1H1C,HIST1H2AJ,HLA-A,HNRNPL,HNRNPM,HNRNPU,HSD17B4,HSP90AA1,HSP90AB1,IDH1,IPO5,ITCH,ITGAM,JAK1,KIDINS220,KPNA2,KRT1,KRT9,LIG1,LILRB4,MAT2B,MCM2,MCM7,MDH1,MEMO1,MFGE8,MSN,MYH9,MYO1C,MYO1E,MYOF,NAA15,NCBP1,NOP56,NOS2,NOTCH2,NPC1,NRAS,NUP93,OASL,OLA1,PDXK,PFAS,PKM,PLAU,PLEC,PLXNA1,PLXNB2,PNKP,POLR1C,PPP1R7,PPP2R1A,PRIM2,PTGS2,PTPRA,PTPRJ,RALA,RBBP7,RIPK3,RNF149,RNPEP,RPF2,RPL14,RPS6,SCFD1,SF3B1,SFPQ,SHMT2,SIRPA,SLC15A3,SLC16A3,SLC20A1,SLC38A2,SMPDL3B,SND1,SNRNP200,SNX27,STAT1,STOM,SUPT5H,TALDO1,TFRC,TGFBR2,TLR7,TNIP1,TPI1,TPP2,TRIM25,TSG101,TSR1,TTC37,TTLL12,UBA1,UBA2,USP5,USP8,VAMP8,VIM,VPS13C,WWP2,XPO1,XPO7	153
**Cell Morphology,Cellular Movement,Hematological System Development and Function,Immune Cell Trafficking,Inflammatory Response**	Cell spreading of phagocytes	4E-05		0.555	FERMT3,ICAM1,ITGAM,ITGB2,LYN,SIRPA	6

Table S4

**Cancer,Cardiovascular Disease,Hematological Disease,Organismal Injury and Abnormalities**	Refractory anemia	4E-05			DNMT1,GNAS,IDH1,NRAS,POLD1,PPAT,PRIM2,SF3B1	8
**Cardiovascular System Development and Function,Cell-To-Cell Signaling and Interaction**	Adhesion of vascular endothelial cells	4E-05		1.744	CD36,FERMT3,ICAM1,ITGAM,ITGB2,RACK1,STAT1,STX6,VIM	9
**Cancer,Endocrine System Disorders,Organismal Injury and Abnormalities,Reproductive System Disease**	Gonadal tumor	4E-05			ABCA3,ACTR2,AKR1B1,ALCAM,ATIC,CAND1,CCT5,CCT6A,CD36,CD47,CDK1,CLUH,CNOT1,CSE1L,DDX3X,DNAJA1,DNMT1,EEF1D,EIF3C,EIF3F,ENO1,FLOT1,GART,GMDS,GNA13,GNAI2,GNAS,HIST1H1C,HNRNPM,HSP90AA1,HSP90AB1,IDH1,ITCH,KPNA2,KRT1,KRT9,LIG1,LYN,MAT2B,MYH9,MYO1E,MYOF,NOS2,NOTCH2,NPC1,NRAS,OLA1,PKM,PLAU,PLXNA1,PNKP,POLR1C,PPP2CA,PPP2R1A,PRIM2,PTGS2,RAP2C,RNF149,RNPEP,RPF2,SEC24B,SHMT2,SLC16A3,SNRNP200,SNX27,STAT1,STOM,TGFBR2,TPI1,TRIM25,TSG101,TTC37,UBA1,VIM,VPS13C,WWP2,XPO1,XPO7	78
**Infectious Diseases**	Replication of virus	4.1E-05		-1.651	ATP5F1B,DDX3X,DNAJA1,FASN,HNRNPM,HSP90AB1,IFITM3,JAK1,MOV10,MVP,NCL,OASL,STAT1,TGFBR2,TLR7,TSG101,ZC3HAV1	17
**Cancer,Organismal Injury and Abnormalities**	Connective or soft tissue tumor	4.1E-05			ABCA3,ATIC,ATP2B1,ATP7A,CCT8,CLUH,CORO1C,DCTN1,ECPAS,EIF3D,FASN,FLNA,FYN,GALK1,GART,GNAI2,GNAS,GRB2,HSD17B4,HSP90AA1,HSP90AB1,HSPA4,IDH1,KIDINS220,KRT10,LGALS3BP,LYN,MAPK3,MCM7,MDH1,MFGE8,MYH9,MYO1C,NCBP1,NDRG1,NOS2,NOTCH2,NRAS,OASL,PCNA,PFAS,PI4K2A,PKM,PLAU,PLD3,PLEC,PLXNB2,PPP2R1A,PTGS2,PTPRC,RPN1,RPS26,RPS6,SFPQ,SHMT1,SLC15A3,SLC38A2,SLC7A1,STAT1,SUPT5H,TKT,TMEM59,TNFRSF1B,TNIP1,TPP2,TTC37,XPO1	67
**Cardiovascular System Development and Function,Organismal Development**	Angiogenesis	4.2E-05		1.644	ADAM8,AKR1B1,ALCAM,ATP5F1B,ATP7A,C5AR1,CD36,DAAM1,DNM2,EDIL3,FERMT3,FLNA,G6PD,GNA13,HSP90AA1,ICAM1,IL6ST,ITGAM,ITGB2,KRT1,MFGE8,MYH9,MYO1E,MYOF,NCL,NDRG1,NOS2,NRAS,NUS1,PGK1,PKM,PLA2G4A,PLAU,PTGS2,PTPRJ,RAP1B,RBPJ,RNF213,RNH1,RPSA,SARS,SPRED1,STAT1,STX6,TGFBR2,TKT,VIM,WARS	48
**Cellular Movement**	Chemotaxis	4.2E-05	Increased	2.244	ADAM8,C5AR1,CD36,CD47,CORO1B,DOCK2,FCER1G,FLOT1,FYN,GNA13,GNAI2,GNAI3,GNAS,GNB2,GRB2,ICAM1,ITGAM,ITGB2,LYN,MAPK3,NUS1,PFN1,PLAU,PLEC,PTGS2,PTPRC,PTPRJ,RALA,SIRPA,TRPV2	30
**Cellular Movement**	Arrest in movement of cells	4.3E-05			FLNA,GNAI2,ICAM1,ITGAM,ITGB2	5
**Inflammatory Response**	Inflammation of body cavity	4.3E-05		-1.104	ABCA3,ABCG1,ACO2,C5AR1,CD36,CD47,CTSD,ENO1,FASN,GNAI2,HLA-A,ICAM1,IDE,IL6ST,ITCH,ITGAM,ITGB2,JAK1,LYN,MTA2,MYH9,NOS2,NOTCH2,NPC1,PKM,PLA2G4A,PLAU,POLD1,PPAT,PTGS2,PTPRC,PTPRJ,RIPK3,SF3B1,SQSTM1,STAT1,STUB1,TAX1BP1,TGFBR2,TKT,TLR7,TNFRSF1B,TPI1,TSTA3	44
**Antigen Presentation,Inflammatory Response**	Antigen presentation	4.3E-05		0.108	AP3B1,CTSD,FCER1G,HLA-A,HSP90AA1,HSP90AB1,LILRB4,RAB5B,RAB8B	9
**Molecular Transport**	Secretion of molecule	4.4E-05		1.401	ABCG1,ACLY,ACSL4,CA2,CD36,CFL1,DNM1L,DPYSL2,FCER1G,FLOT2,FYN,GNA13,GNAI3,IL6ST,KCNN4,LYN,MYO1C,NOS2,PLA2G4A,PTGS2,PTPRC,RHBDF2,SIRPA,SLC16A3,SLC2A1,SLC38A2,SNAP23,STAT1,STEAP3,TNFRSF1B,VAMP8	31

Table S4

**Cell Signaling,Cell-To-Cell Signaling and Interaction**	Cytokine and chemokine mediated signaling pathway	4.5E-05			CD36,FYN,GRB2,HSP90AA1,ICAM1,IL6ST,ITGAM,ITGB2,JAK1,NOS2,PTGS2,PTPRC,RPS27A,STAT1,TNFRSF1B,VIM	16
**Cancer,Organismal Injury and Abnormalities**	Recurrent cancer	4.5E-05			ATIC,DNMT1,FYN,GART,HSP90AA1,HSP90AB1,IDH1,LYN,POLD1,PPAT,PRIM2,PTGS2,SF3B1,TGFBR2,TLR7,XPO1	16
**Cellular Movement,Hematological System Development and Function,Immune Cell Trafficking,Inflammatory Response**	Infiltration by neutrophils	4.6E-05		1.548	ADAM8,C5AR1,CD36,EDIL3,FCER1G,ICAM1,ITGAM,ITGB2,NOS2,PTGS2,TGFBR2,TNFRSF1B,TNIP1,YBX1	14
**DNA Replication, Recombination, and Repair**	Excision repair	4.6E-05		-0.506	APEX1,EIF3A,HNRNPU,LIG1,PCNA,POLD1,POLR2A,POLR2B,PRPF19,RPS27A,YBX1	11
**Cellular Growth and Proliferation,Organismal Development**	Growth of bacteria	4.7E-05		-1.129	Abcb1b,CTSD,ITGAM,LIG1,NOS2,PNKP,RAN,TFRC,TSG101	9
**Carbohydrate Metabolism**	Synthesis of carbohydrate	4.8E-05		-1.422	AKR1B1,AMDHD2,CD36,CMAS,G6PD,GFPT1,GMDS,GMPPB,ICAM1,IDH1,ITGB2,LYN,NANS,NOS2,PGD,PI4K2A,PI4K2B,PLA2G4A,PLAU,PLEK,PPP1CA,PTPRC,RAB5A,RALA,SLC2A1,TKT,TRPV2,TSTA3	28
**Protein Synthesis**	Quantity of interleukin	5E-05		-1.403	APEX1,C5AR1,GNAI2,IL6ST,Irgm1,ITGB2,KRT1,LGALS3BP,LYN,MTA2,STAT1,WWP2	12
**Amino Acid Metabolism, Small Molecule Biochemistry**	Metabolism of glycine	5.1E-05			GART,PHGDH,SHMT1,SHMT2	4
**Cellular Assembly and Organization,Cellular Function and Maintenance,Inflammatory Response**	Maturation of phagosomes	5.1E-05			MSN,RAB5A,RAB7A,SNAP23	4
**Cancer,Organismal Injury and Abnormalities,Respiratory Disease**	Stage I-IIIA non-small cell lung cancer	5.2E-05			ATIC,GART,HSP90AA1,HSP90AB1,POLR2A,PTGS2,RPL7,RPS27A,RPS6	9

Table S4

**Cell-To-Cell Signaling and Interaction, Cellular Function and Maintenance,Inflammatory Response**	Phagocytosis of tumor cell lines	5.2E-05		1.706	GRB2,ITGAM,LYN,PFN1,RAB31,RALA,VIM	7
**Organismal Development,Organismal Injury and Abnormalities**	Abnormal morphology of abdomen	5.3E-05			ACSL4,APEX1,CD36,CD47,CTSD,DNMT1,DOCK2,FCER1G,FKBP4,FYN,GBA,GNAI2,IL6ST,ITGB2,JAK1,KCNN4,LIG1,LYN,MAPK3,MFGE8,NOS2,NOTCH2,NPC1,NRAS,PLAU,PTGS2,PTPRC,PTPRJ,RIPK3,RPSA,SIPA1,SLC20A1,SLC23A2,SNX27,STAT1,STEAP3,TCIRG1,TLR7,TNFRSF1B,TPP2,TRIM25,TSTA3,YBX1	43
**Cellular Function and Maintenance,Molecular Transport**	Exocytosis by cells	5.3E-05		-0.28	EHD1,GNAI2,GNAI3,LILRB4,MYO1G,RAB5A,RALA,SCAMP2,SNAP23,STAM2,VAMP8	11
**Cell Death and Survival,Cellular Compromise**	Cytotoxicity of cells	5.3E-05	Increased	3.057	DOCK2,FCER1G,FERMT3,HLA-A,ICAM1,ITGAM,ITGB2,LYN,MYH9,NOS2,NOTCH2,NPC1,PTPRC,STAT1,TNFRSF1B	15
**Lymphoid Tissue Structure and Development,Tissue Morphology**	Quantity of lymph follicle	5.3E-05		-0.075	C5AR1,DOCK2,GNA13,GNAI2,IL6ST,KIDINS220,LYN,NOS2,NOTCH2,PTPRJ,RAP1B,STAT1,TCIRG1	13
**Cell-To-Cell Signaling and Interaction, Cellular Function and Maintenance,Inflammatory Response**	Phagocytosis by macrophage cancer cell lines	5.4E-05		1.446	GRB2,ITGAM,PFN1,RAB31,RALA	5
**Cellular Assembly and Organization**	Fusion of endosomes	5.4E-05	Increased	2.144	GNAS,NPC1,RAB5A,RAB7A,VAMP8	5

Table S4

**Cell-To-Cell Signaling and Interaction,Hematological System Development and Function,Immune Cell Trafficking,Inflammatory Response**	Activation of T lymphocytes	5.5E-05		1.285	AHNAK,CD47,DOCK2,FCER1G,FYN,GPNMB,HLA-A,HSPA4,ICAM1,ITCH,ITGAM,ITGB2,KCNN4,LYN,PTPRC,PTPRJ,RBPJ,STAT1,TGFBR2,TLR7,TNFRSF1B,VAMP8	22
**Cell-mediated Immune Response,Cellular Movement,Hematological System Development and Function,Immune Cell Trafficking**	Cell movement of T lymphocytes	5.6E-05		1.1	CD47,FLOT1,FYN,GNAI2,HLA-A,HNRNPL,ICAM1,IL6ST,ITGB2,JAK1,MYH9,PLEC,PTGS2,PTPRA,STAT1,TGFBR2,TNIP1	17
**Cellular Movement,Hematological System Development and Function,Immune Cell Trafficking**	Cell movement of antigen presenting cells	5.7E-05		1.879	ALCAM,C5AR1,FLNA,GBA,GNAI3,ICAM1,Irgm1,ITGB2,MAPK3,NDRG1,NOS2,PFN1,PLA2G4A,PLAU,PLEC,PTGS2,PTPRJ,SIRPA,STAT1,TGFBR2,TNIP1,TRPV2,YBX1	23
**Connective Tissue Development and Function,Tissue Morphology**	Quantity of connective tissue	5.7E-05		-0.48	ACACA,ACTA1,AHNAK,C5AR1,CD36,CD47,CTSK,FASN,GFPT1,GNAS,GPNMB,IL6ST,ITGAM,LIG1,LYN,MAPK3,MFGE8,NOS2,NOTCH2,PDS5A,PFN1,PLA2G4A,PLAU,PTPRC,SIPA1,SLC20A1,SLC7A1,SQSTM1,STAT1,STEAP3,TFRC,TGFBR2,TKT	33
**Cancer,Cardiovascular Disease,Hematological Disease,Organismal Injury and Abnormalities**	Refractory anemia with excess blasts	5.9E-05			DNMT1,IDH1,NRAS,POLD1,PPAT,PRIM2,SF3B1	7
**Cellular Assembly and Organization**	Formation of cytoskeleton	5.9E-05		0.363	ACTR3,ARAP1,ARPC2,CD47,CFL1,CORO1C,CORO7/CORO7-PAM16,DCTN1,DPYSL2,FKBP4,FLNA,FYN,GNA13,GRB2,ICAM1,MYADM,MYO1C,PFN1,PTPRA,SIRPA,TGFBR2,VASP	22

Table S4

**Cell Death and Survival,Cellular Compromise**	Cytotoxicity of leukocytes	5.9E-05	Increased	2.742	DOCK2,FERMT3,HLA-A,ICAM1,ITGAM,ITGB2,LYN,MYH9,NOS2,NOTCH2,NPC1,PTPRC,STAT1	13
**Cell-To-Cell Signaling and Interaction,Inflammatory Response**	Immune response of tumor cell lines	6E-05		1.706	FCER1G,GRB2,ITGAM,LYN,PFN1,RAB31,RALA,VIM	8
**Cancer,Hematological Disease,Immunological Disease,Organismal Injury and Abnormalities**	Secondary acute myeloid leukemia	6E-05			DNMT1,IDH1,LYN,NRAS,POLD1,PPAT,PRIM2,SF3B1	8
**Cell Morphology**	Polarization of leukocytes	6E-05		1.698	CD36,DOCK2,FCER1G,FYN,GNAI3,ITGB2,MSN,MYH9,RBPJ,SIPA1,SIRPA	11
**Cell-To-Cell Signaling and Interaction, Hematological System Development and Function,Inflammatory Response**	Binding of professional phagocytic cells	6.1E-05	Increased	2.182	ALCAM,FERMT3,ICAM1,Irgm1,ITGAM,ITGB2,LYN,MSN,NOS2,NOTCH2,PLAU,PTGS2,PTPRC,TGFBR2,TRPV2	15
**Cancer,Organismal Injury and Abnormalities,Reproductive System Disease**	Malignant neoplasm of male genital organ	6.2E-05			ABCA3,ABCE1,ACLY,ACSL4,ACTR1A,ADSL,ADSSL1,AHNAK,AKR1B1,ALDH9A1,AP3B1,APEX1,ATP1A1,ATP6V0A1,CA2,CAND1,CAPZA1,CCT2,CLUH,CMIP,CNOT1,CPD,CPNE8,CSE1L,CTSD,CTSK,DCTN1,DDX17,DDX3X,DENND4B,DHX29,DNAJC13,DNM1L,DNMT1,DOCK2,ECPAS,EDIL3,EEF1G,EIF2S1,EIF2S3,EIF3A,EIF3C,EIF3F,EIF3I,FASN,FKBP4,FLNA,FYN,G3BP1,GART,GFPT1,GNAI2,GNAS,GPNMB,GUSB,HK3,HSP90AA1,HSP90AB1,HSPA4,IDH1,ITGAM,JAK1,KRT1,KRT10,KRT9,LILRB4,LRP12,MCM2,MCM7,MDH1,MFGE8,MYADM,MYH9,MYO1C,MYO1E,MYO1G,NOP56,NOS2,NOTCH2,NRAS,OLA1,PCNA,PDS5A,PGK1,PKM,PLD3,PLEC,PLEK,PLXNB2,POLD1,POLR2B,PPAT,PPP2CA,PPP2R1A,PRIM2,PTGS2,PTPN23,PTPRC,RANGAP1,RAP2C,RARS,RCC2,RNF213,RNPEP,RPS27A,RRP9,SEC24B,SF3B1,SHMT2,SIRPA,SLC12A4,SLC16A3,SLC29A1,SLC2A1,SLC3A2,SLC7A1,SND1,SNRNP200,STAT1,STEAP3,STOM,STX6,SUPT5H,TGFBR2,TKT,TLR7,TRIM28,TRPV2,TTC37,VIM,VPS13C,VWA5A,WWP2,XPO1,YBX1	135
**Cell-To-Cell Signaling and Interaction, Hematological System Development and Function,Immune Cell Trafficking,Inflammatory Response**	Activation of leukocytes	6.3E-05		1.983	AHNAK,AP3B1,C5AR1,CD36,CD47,DOCK2,FCER1G,FYN,GPNMB,HLA-A,HSPA4,ICAM1,ITCH,ITGAM,ITGB2,KCNN4,KIDINS220,LGALS3BP,LYN,MAPK3,NDRG1,NOS2,NOTCH2,NPC1,PTGS2,PTPRC,PTPRJ,RAB5B,RAB8B,RBPJ,SIRPA,STAT1,TGFBR2,TLR7,TNFRSF1B,VAMP8	36

Table S4

**Cell Cycle**	Interphase	6.3E-05		-0.323	ACTR1A,CDK1,CSE1L,DCTN1,FLNA,GRB2,HSP90AA1,IDH1,MAPK3,MCM2,MCM7,NDRG1,NOTCH2,PCNA,PLA2G4A,POLR2A,PPP1CA,PPP2CA,PPP2R1A,PPP2R2A,PRIM2,PTGS2,RALA,RIPK3,RPS6,STAT1,TGFBR2,TSG101,USP8	29
**Cancer,Endocrine System Disorders,Organismal Injury and Abnormalities,Reproductive System Disease**	Ovarian tumor	6.4E-05			ABCA3,ACTR2,AKR1B1,ALCAM,ATIC,CAND1,CCT5,CCT6A,CD36,CD47,CDK1,CLUH,CNOT1,CSE1L,DDX3X,DNAJA1,DNMT1,EEF1D,EIF3F,ENO1,FLOT1,GART,GMDS,GNA13,GNAI2,GNAS,HIST1H1C,HNRNPM,HSP90AA1,HSP90AB1,IDH1,ITCH,KPNA2,KRT1,KRT9,LIG1,LYN,MAT2B,MYH9,MYO1E,MYOF,NOS2,NOTCH2,NPC1,NRAS,OLA1,PKM,PLAU,PLXNA1,PNKP,POLR1C,PPP2CA,PPP2R1A,PRIM2,PTGS2,RNF149,RNPEP,RPF2,SHMT2,SLC16A3,SNRNP200,SNX27,STAT1,STOM,TGFBR2,TPI1,TRIM25,TSG101,TTC37,UBA1,VIM,VPS13C,WWP2,XPO1,XPO7	75
**Hereditary Disorder,Organismal Injury and Abnormalities,Skeletal and Muscular Disorders**	Autosomal dominant myopathy	6.4E-05			ACLY,ACTA1,AHNAK,CA2,CFL1,COLEC12,DCTN1,DNM2,GARS,KIDINS220,MARS,RAB7A,SDCBP,SQSTM1,TGFBR2	15
**Cell Morphology**	Shape change of lymphocytes	6.4E-05			CD47,DOCK2,KCNN4,MYH9,PTPRA,RAP1B	6
**Cellular Function and Maintenance,Molecular Transport**	Secretory pathway	6.5E-05		-0.16	EHD1,GNAI2,GNAI3,GUSB,LILRB4,MYO1G,RAB31,RAB5A,RALA,SCAMP2,SNAP23,STAM2,STX6,VAMP8	14
**Cell Death and Survival**	Cell death of macrophage cancer cell lines	6.6E-05		1.673	HSP90AB1,ITGAM,MAPK3,MVP,PTPRC,RIPK3,TGFBR2	7
**Cell-To-Cell Signaling and Interaction, Hematological System Development and Function,Inflammatory Response**	Response of neutrophils	6.7E-05		0.9	C5AR1,CD36,CD47,FCER1G,ICAM1,ITGAM,ITGB2,LYN,PLAU	9
**Hematological System Development and Function,Organismal Functions**	Coagulation of blood	6.9E-05		0.478	AP3B1,CAPZA1,CD36,EHD1,FCER1G,FLNA,FYN,GNA13,GNAI2,LYN,MAPK3,MFGE8,NOS2,NPC1,PLEK,PTGS2,PTPRJ,RAB5A,VASP	19

Table S4

**Cancer,Hematological Disease,Immunological Disease,Organismal Injury and Abnormalities**	Recurrent leukemia	6.9E-05			DNMT1,FYN,HSP90AA1,HSP90AB1,IDH1,LYN,POLD1,PPAT,PRIM2,SF3B1	10
**Hematological System Development and Function,Tissue Morphology**	Quantity of granulocytes	6.9E-05		-1.225	ADAM8,C5AR1,CD36,CD47,GNAI2,GNAS,ICAM1,IL6ST,ITGAM,ITGB2,LYN,NOS2,PPP2CA,PREP,PTGS2,SIPA1,SPRED1,TCIRG1,TNFRSF1B,TSTA3,YBX1	21
**Organismal Injury and Abnormalities**	Benign lesion	7E-05		-1.148	AHNAK,ALCAM,ATP1A1,ATP2C1,CA2,CDK1,CLUH,CRYZ,DDX17,DKC1,DNM1L,DNMT1,FLNA,GNAS,GRB2,HSP90AB1,IDH1,IFIT1B,IFITM3,IL6ST,LIG1,LYN,MAPK3,MDH1,MSN,MYH9,NDRG1,NRAS,PCNA,PLAU,PLD3,PLXNB2,POLD1,POLR1C,PRIM2,PTGS2,SEC24B,SLC29A1,SLC2A1,SLC7A1,SUPT5H,TGFBR2,TMEM59,TNFRSF1B,TTC37,USP8	46
**Cellular Assembly and Organization,Tissue Development**	Fibrogenesis	7E-05		1.302	ACTA1,ACTR3,ARAP1,ARPC2,CD47,CFL1,CORO7/CORO7-PAM16,DAAM1,DCTN1,DPYSL2,FKBP4,Fmnl1,FYN,GNA13,GRB2,ICAM1,MYADM,MYO1C,PFN1,PTPRA,SIRPA,TGFBR2,TNFRSF1B,VASP,VIM	25
**Carbohydrate Metabolism, Nucleic Acid Metabolism, Small Molecule Biochemistry**	Pentose shunt of D-glucose	7.2E-05			G6PD,PGD,TALDO1,TKT	4
**Cancer,Organismal Injury and Abnormalities,Tumor Morphology**	Metastatic progressive EGFR L858R mutation positive large cell carcinoma	7.2E-05			ATIC,GART,HSP90AA1,HSP90AB1	4
**Cancer,Organismal Injury and Abnormalities,Respiratory Disease**	Unresectable lung adenocarcinoma	7.2E-05			ATIC,GART,HSP90AA1,HSP90AB1	4
**Cancer,Hematological Disease,Immunological Disease,Organismal Injury and Abnormalities**	Newly diagnosed leukemia	7.2E-05			DNMT1,FYN,IDH1,LYN,NRAS,POLD1,PPAT,PRIM2,SF3B1	9

Table S4

**Cancer,Organismal Injury and Abnormalities**	Cancer of cells	7.4E-05		-1.286	ABCG1,ACO2,ACSL4,ADSL,AHNAK,ALCAM,ALDH9A1,AMDHD2,ARAP1,ATIC,ATP1A1,ATP2C1,ATP7A,CA2,CCT3,CCT7,CD36,CD47,CDK1,CFL1,CMIP,CNOT1,CORO1B,CPD,CSE1L,CTPS1,DAAM1,DDX3X,DENND4B,DHX15,DNAJC13,DNM1L,DNMT1,DOCK2,EDIL3,EEF1G,EIF2A,EIF2S3,EIF3A,EIF3B,FASN,FYN,G3BP1,GART,GNA13,GNAI2,GNAS,GPNMB,GRB2,HIST1H1C,HIST1H2AJ,HLA-A,HNRNPM,HNRNPU,HSP90AA1,HSP90AB1,ICAM1,IDE,IDH1,IPO5,IPO7,ITCH,ITGAM,ITGB2,JAK1,KIDINS220,KPNA2,KPNB1,KRT1,KRT10,KRT2,KRT9,LILRB4,LYN,MAPK3,MARS,MCM2,MPEG1,MSN,MTA2,MYH9,MYO1C,MYO1E,MYO1G,MYOF,NCL,NCSTN,NOS2,NOTCH2,NPC1,NRAS,NUS1,OASL,OLA1,PCNA,PKM,PLAU,PLEC,PLXNB2,POLD1,PPAT,PPP1CA,PPP2CA,PPP2R1A,PRIM2,PRPF19,PTGS2,PTPRA,PTPRC,RALA,RAN,RHBDF2,RIPK3,RNF213,RNH1,RPL10,RPL13,RPL14,RPL4,RPSA,SARS,SDCBP,SF3B1,SFPQ,SHMT1,SHMT2,SIRPA,SLC23A2,SLC2A1,SMPDL3B,SND1,SNRNP200,SNX2,SNX27,SPRED1,STAT1,STEAP3,STRAP,TAX1BP1,TCIRG1,TCP1,TFRC,TGFBR2,TLR7,TMEM59,TNIP1,TPP2,TRIM14,TRIM25,UBA2,USP8,VIM,XPNPEP1,XPO1	154
**Protein Synthesis**	Quantity of interferon	7.4E-05		-1.8	GNAI2,Irgm1,LGALS3BP,LYN,MTA2,STAT1,WWP2	7
**Cancer,Hematological Disease,Immunological Disease,Organismal Injury and Abnormalities**	Diffuse large B-cell lymphoma	7.8E-05			CD36,DNMT1,DOCK2,GNA13,GRB2,HIST1H1C,HNRNPM,HSP90AA1,HSP90AB1,IDH1,MYO1G,NOTCH2,POLD1,PRIM2,PTPRC,SHMT2,TNIP1,XPO1	18
**Cancer,Hematological Disease,Immunological Disease,Organismal Injury and Abnormalities**	Hodgkin lymphoma	7.8E-05			CMIP,CSE1L,HSP90AA1,HSP90AB1,ICAM1,NRAS,POLD1,PRIM2,PTPRC	9
**Immunological Disease**	Abnormal morphology of immune system	7.8E-05			Abcb1b,CD36,CTSD,DOCK2,FCER1G,FYN,GBA,GNAI2,HLA-A,ICAM1,ITGAM,ITGB2,LILRB4,LYN,NOS2,PTPRC,PTPRJ,RIPK3,TNIP1,TPP2	20
**Lymphoid Tissue Structure and Development,Organ Morphology,Tissue Morphology**	Quantity of lymphoid organ	7.9E-05		-1.146	Abcb1b,CTSD,EEF1D,FCER1G,FYN,GBA,GNAI2,ITGB2,JAK1,LYN,MAPK3,MFGE8,NOS2,PTPRC,RIPK3,STAM2,STAT1,STEAP3,TPP2	19
**Cell Death and Survival,Gastrointestinal Disease,Hepatic System Disease,Organismal Injury and Abnormalities**	Cell death of liver cells	8E-05		0.893	DNM1L,IL6ST,ITGB2,MAPK3,NCL,NOS2,NPC1,PTGS2,PTPRC,RIPK3,SLC20A1,STAT1,TLR7,TNFRSF1B,TNIP1	15

Table S4

**Cell-To-Cell Signaling and Interaction, Cellular Function and Maintenance,Hematological System Development and Function,Inflammatory Response**	Phagocytosis of red blood cells	8.2E-05		1.117	CD36,ITGAM,ITGB2,LYN,SIRPA		5
**Cancer,Organismal Injury and Abnormalities**	Liquid tumor	8.3E-05		-1.109	ACSL4,AHNAK,APEX1,ARAP1,ATP1A1,CD36,CDK1,CFL1,CLUH,CPD,DAAM1,DDX3X,DHX15,DIS3,DNAJA1,DNM1L,DNM2,DNMT1,ECPAS,EEF1D,EIF3C,EIF3D,EIF3I,EIF3L,FCER1G,FLNA,FYN,GMDS,GNAS,HK3,HLA-A,HNRNPU,HSP90AA1,HSP90AB1,HSPA4,IDH1,IPO7,ITGAM,ITGB2,JAK1,KRT1,KRT10,LILRB4,LYN,MARS,MCM7,MYH9,MYO1E,NANS,NCSTN,NOTCH2,NRAS,PCNA,PDS5A,POLD1,PPAT,PPP1R7,PRIM2,PTGS2,PTPRA,PTPRC,RACK1,RIPK3,RNPEP,RPL13,RPL28,RPL3,RPL4,RPL6,RPL7,RPS14,RPS27A,RPS6,SARS,SDCBP,SF3B1,SIPA1,SLC2A1,SMU1,SPRED1,SUPT5H,TAX1BP1,TGFBR2,TLR7,TNIP1,TPI1,TRIM25,USP8,XPO1,XPO7		90
**Molecular Transport**	Quantity of metal ion	8.3E-05		0.562	AKR1B1,ATP2B1,C5AR1,EIF2S1,FCER1G,FYN,GNA13,GNAI2,GNAI3,GNAS,ICAM1,ITGAM,LILRB4,LYN,MEMO1,MYH9,NOS2,NPC1,PLAU,PTGS2,PTPRC,PTPRJ,STUB1		23
**Cellular Assembly and Organization**	Transport of endosomes	8.8E-05			EHD1,FLNA,RPS27A,SNX27,SQSTM1,STAM2,TOM1,TSG101		8
**Cardiovascular System Development and Function,Cellular Movement**	Migration of endothelial cells	9.4E-05		1.066	ALCAM,ATP5F1B,CD36,EDIL3,FLNA,G6PD,HSP90AB1,ICAM1,ITGB2,NCL,NUS1,PKM,PLA2G4A,PLAU,PLXNA1,PTGS2,PTPRJ,STX6,TGFBR2,VASP,VIM,WARS		22
**Cell Death and Survival**	Apoptosis of leukocytes	9.4E-05		1.849	ABCG1,ADAM8,CD47,FCER1G,FYN,GNAS,HIST1H1C,HSP90AB1,ICAM1,IL6ST,Irgm1,ITGAM,ITGB2,LYN,NOS2,NPC1,PLA2G4A,PTPRC,RIPK3,RPS6,STAT1,STUB1,TLR7,TNFRSF1B,TPP2,WWP2		26
**Cell Death and Survival**	Cell death of T lymphocytes	9.6E-05		1.375	ADAM8,CD47,CDK1,CTSD,FYN,GNAS,HIST1H1C,ICAM1,Irgm1,NOS2,PTPRC,RIPK3,RPS6,STAT1,STUB1,TGFBR2,TLR7,TNFRSF1B,TPP2,WWP2		20
**Cellular Movement,Reproductive System Development and Function**	Cell movement of gonadal cell lines	9.6E-05		0.849	CD47,DOCK2,FYN,NCL,NUS1,PLAU,RACK1,SIRPA		8
**Cancer,Organismal Injury and Abnormalities,Respiratory Disease**	Stage 3A resectable non-small cell lung cancer	9.9E-05			ATIC,GART,HSP90AA1,HSP90AB1		4
**Lipid Metabolism, Small Molecule Biochemistry**	Incorporation of phospholipid	9.9E-05		0.811	ABCA3,ACLY,ACSL4,NPC1		4

Table S4

**Cancer,Hematological Disease,Organismal Injury and Abnormalities**	Refractory malignant lymphoid neoplasm	1E-04			DNMT1,FYN,HSP90AA1,HSP90AB1,LYN,NRAS,POLD1,PPAT,PRIM2,PTGS2,SF3B1	11
**Cellular Assembly and Organization**	Fusion of lysosome	1E-04		1.982	ATP6V0A1,NPC1,RAB5A,RAB7A,TSG101,VAMP8	6
**Cancer,Hematological Disease,Immunological Disease,Organismal Injury and Abnormalities**	Acute monocytic leukemia (M5)	1E-04			IDH1,NRAS,POLD1,PPAT,PRIM2,SF3B1	6
**Cellular Movement,Hematological System Development and Function,Immune Cell Trafficking**	Homing of leukocytes	0.0001		1.629	ADAM8,C5AR1,CD47,DOCK2,FCER1G,FLOT1,FYN,GNA13,GNAI2,GNAI3,GNAS,ICAM1,ITGAM,ITGB2,JAK1,LYN,MAPK3,PFN1,PLAU,PLEC,PTPRJ,RAP1B,TRPV2	23
**Infectious Diseases**	Replication of RNA virus	0.0001	Decreased	-2.959	DDX3X,DNAJA1,FASN,HNRNPM,HSP90AB1,IFITM3,JAK1,MOV10,MVP,NCL,OASL,TLR7,TSG101,ZC3HAV1	14
**Molecular Transport**	Export of molecule	0.0001		1.69	Abcb1b,ABCG1,ATP7A,CD36,CPSF1,DDX39B,MSN,MVP,NCBP1,NPC1,NUP93,PTGS2,RAN,SLC12A4,SLC1A5,SLC38A2,SLC4A7,XPO1,XPO7	19
**Free Radical Scavenging**	Production of superoxide	0.0001		1.134	AKR1B1,C5AR1,CD36,G6PD,ITGAM,ITGB2,JAK1,NCF2,NOS2,RACK1	10
**Lipid Metabolism, Small Molecule Biochemistry**	Synthesis of lipid	0.0001		0.113	Abcb1b,ABCG1,ACACA,ACLY,ACSL4,AKR1B1,ALDH3B1,ATP1A1,C5AR1,CD36,FASN,FCER1G,FYN,G6PD,GBA,GNAI3,HSD17B4,ITGB2,KPNB1,LYN,MAPK3,NPC1,NUS1,PGD,PI4K2A,PI4K2B,PLA2G4A,PLAU,PPAT,PRPF19,PTGS2,RAB5A,RALA,RAN,TGFBR2,TNFRSF1B,TRPV2,VIM,YBX1	39
**Cellular Assembly and Organization,Tissue Development**	Polymerization of filaments	0.00011		-0.711	ACTR3,ARPC2,CFL1,CORO7/CORO7-PAM16,DCTN1,FKBP4,GRB2,ICAM1,MYADM,MYO1C,PFN1,VASP	12
**Developmental Disorder,Embryonic Development,Organismal Survival**	Death of embryo	0.00011	Increased	2.209	DDX3X,FASN,HNRNPL,PCNA,PFN1,POLD1,PRPF19,RBPJ,RPSA,SF3B1,SNAP23,TKT	12
