# Supplementary material for: Antigen-encapsulating host extracellular vesicles derived from Salmonella-infected cells stimulate pathogen-specific Th1-type responses in vivo
Source: PLoS Pathog. 2021 May 6;17(5):e1009465. doi: 10.1371/journal.ppat.1009465 (PMC8101724; doi:10.1371/journal.ppat.1009465)
Supplement: S5 Table — The exosomal proteins identified by proteomics with a protein level differentially regulated by infection (48 hpi) were analyzed by Ingenuity Pathway Analysis software to identify the canonical functions that these proteins regulate. Identified proteins related to endocytosis are shown in the table. Symbol, Entrez Gene Name, Protein Accession number, Experimental p-value, Fold Change, Protein Type(s), Mapped Entrez Gene ID for Human and Mouse are shown for each protein. (PDF) [file ppat.1009465.s018.pdf]

Table S5

Symbol	Entrez Gene Name	Accession number	Expr p-value	Expr Fold Change	Type(s)	Entrez Gene ID for Human	Entrez Gene ID for Mouse
RAB5A	RAB5A, member RAS oncogene family	Q9CQD1	0.0001	10	enzyme	5868	271457
RAB5B	RAB5B, member RAS oncogene family	P61021	0.003	9	enzyme	5869	19344
FLOT2	flotillin 2	Q60634	0.0049	7.8	other	2319	14252
FLOT1	flotillin 1	G3UYU4	0.00082	7	other	10211	14251
FYN	FYN proto-oncogene, Src family tyrosine kinase	P39688	0.00027	5.5	kinase	2534	14360
RAB5C	RAB5C, member RAS oncogene family	P35278	0.00077	2	enzyme	5878	19345
ITGAM	integrin subunit alpha M	E9Q604	0.025	1.8	transmembrane receptor	3684	16409
ITGB2	integrin subunit beta 2	P11835	0.04	1.6	transmembrane receptor	3689	16414
HLA-A	major histocompatibility complex, class I, A	P01900	0.039	1.5	other	3105	667977|15013|15006|110557|69717|14972|15015|15018|14964|14963|15007
COPG1	coatomer protein complex subunit gamma 1	Q9QZE5	0.0017	-1.667	transporter	22820	54161
COPB1	coatomer protein complex subunit beta 1	Q9JIF7	0.0088	-2	transporter	1315	70349
DNM2	dynamin 2	P39054	0.00036	-2.5	enzyme	1785	13430
ACTA1	actin, alpha 1, skeletal muscle	P68134	0.0013	-2.5	other	58	11459
FLNA	filamin A	Q8BTM8	0.0001	-3.333	other	2316	192176
COPG2	coatomer protein complex subunit gamma 2	Q9QXK3	0.0054	-3.333	transporter	26958	54160
